# N-Heterocyclic carbene-catalyzed deaminative cross-coupling of aldehydes with Katritzky pyridinium salts†

**DOI:** 10.1039/d0sc00225a

**Published:** 2020-02-26

**Authors:** Inwon Kim, Honggu Im, Hyeonyeong Lee, Sungwoo Hong

**Affiliations:** Department of Chemistry, Korea Advanced Institute of Science and Technology (KAIST) Daejeon 34141 Korea hongorg@kaist.ac.kr; Center for Catalytic Hydrocarbon Functionalizations, Institute for Basic Science (IBS) Daejeon 34141 Korea

## Abstract

By employing an N-heterocyclic carbene (NHC) catalyst, we developed a versatile catalytic system that enables deaminative cross-coupling reactions of aldehydes with redox-active pyridinium salts. Katritzky pyridinium salts behave as single-electron oxidants capable of generating alkyl radicals enabled by the redox properties of the enolate form of Breslow intermediates. The resultant alkyl radical undergoes efficient recombination with the NHC-bound aldehyde-derived carbonyl carbon radical for the formation of a C–C bond. The mild and transition metal-free reaction conditions tolerate a broad range of functional groups, and its utility has been further demonstrated by the modification of a series of peptide feedstocks and application to the three-component dicarbofunctionalization of olefins.

## Abstract 2

By employing an N-heterocyclic carbene (NHC) catalyst, we developed a versatile catalytic system that enables deaminative cross-coupling reactions of aldehydes with redox-active pyridinium salts.

## Graphical abstract

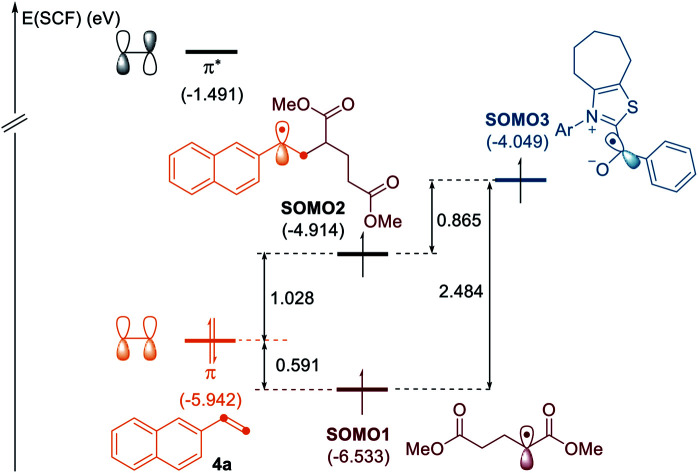

## Introduction

**Scheme 1:**
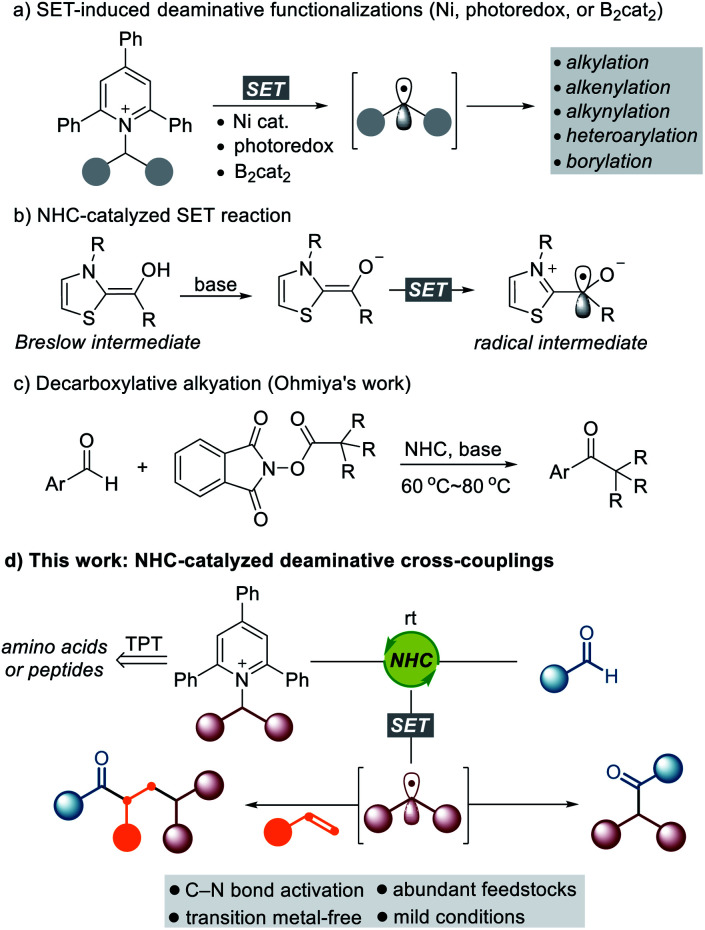
Design plan: NHC-catalyzed deaminative coupling of aldehydes with Katritzky salts.

The utilization of widely available and naturally abundant functionalities is of great interest because it promises a convenient and cost-effective synthetic method to enable the rapid modification of an important class of feedstocks. α-Amino acids and their derivatives are prevalent structural motifs across natural products^1^ and medicinally relevant compounds,^2^ and the development of a general method to exploit them as synthetic intermediates is highly desirable for rapid modification and generation of new chemical entities with broad utility. Recently, Katritzky pyridinium salts, easily prepared from the condensation of primary amines with commercially available pyrylium salts, have emerged as a powerful tool for the generation of alkyl radical species.^3^ Watson,^4^ Glorius,^5^ Aggarwal,^6^ Shi,^7^ Gryko,^8^ Xiao,^9^ Martin,^10^ Rueping,^11^ and Molander^12^ have demonstrated the utility of Katritzky salts to form various types of C–C and C–B bonds *via* deaminative cross-coupling under Ni-catalyzed or photomediated conditions (Scheme 1a). Despite the impressive achievements in this field, a synthetic method that efficiently transforms an amine functionality into a carbonyl group *via* a deaminative radical pathway is still underexplored.

In recent years, NHC-catalyzed radical reactions have shown great potential as a new aspect of reactivity in contrast to what is typically seen for an organocatalyst for the umpolung of aldehydes.^13^ The Fukuzumi group observed a single electron transfer (SET) from the enolate form of Breslow intermediates.^14^ The Studer group disclosed the esterification of aldehydes *via* NHC catalysis using TEMPO in single-electron oxidation.^15^ Since these pioneering studies, important contributions in the arena of NHC-catalyzed SET reactions have been made.^16^ Very recently, the Ohmiya group reported decarboxylative radical couplings of Breslow intermediates with redox-active esters such as *N*-(acyloxy)phthalimides, which demonstrated that merging NHC catalysis and single-electron chemistry has significant potential to form C–C bonds.^17^ Despite the significant advances in NHC-enabled radical reactions, single-electron oxidants that are suitable for effective cross-coupling remain limited. In this context, there is a growing demand for the identification of new types of SET oxidants that enable cross-coupling reactions with NHC-bound aldehyde-derived carbonyl carbon radicals in a predictable and controllable manner.

Inspired by the aforementioned studies on NHC-catalyzed SET reactions, we questioned whether redox-active amines such as Katritzky salts could be directly reduced by the enolate form of the Breslow intermediate. As outlined in Scheme 1d, we imagined that the alkyl radical generated by the SET pathway could be combined with the oxidized enolate form of the Breslow intermediate, which would present a new opportunity for the rapid modification of a series of amino acid-derived and peptidic compounds. This powerful transformation offers a new retrosynthetic disconnection *via* C–N bond cleavage for the synthesis of high-value carbonyl compounds. Room temperature is sufficient for these cross-coupling reactions, and the photomediated process is not required. Moreover, challenging intermolecular three-component dicarbofunctionalization of alkenes can be successfully achieved through a radical relay with complete regioselectivity.

## Results and discussion

To test the viability of this scenario, our investigation was initiated by monitoring the reactivity of Katritzky salt **1a** derived from glutamic acid with aldehyde **2a**, and the results are presented in Table 1. After screening the reaction parameters, we were pleased to find that the desired transformation was feasible to afford the coupling product **3a** in 74% yield in the presence of a catalytic amount of the seven-membered ring fused thiazolium salt **NHC1** and Cs_2_CO_3_ in DMSO at room temperature. Among the solvents screened, DMSO was optimal, and less polar solvents led to significantly lower yields (entries 2–5). A thorough survey of NHC catalysts revealed that the N-substituent and backbone of the NHC precursors were critical in this reaction: NHC precursors bearing an *N*-mesityl group (**NHC2**) or a cyclohexyl group as the backbone (**NHC3**) provided lower yields (entries 8 and 9). The choice of base was critical for the reaction efficiency and the screening of various bases indicated that Cs_2_CO_3_ was most effective. As expected, control experiments verified that the NHC catalyst was indispensable for the successful reaction (entry 7). Comparable reactivity was observed when the reaction occurred under dark conditions, revealing that visible light is not required for this transformation (entry 10). We found that the reaction was completely inhibited in the presence of TEMPO, suggesting that a radical pathway is likely to be operative (entry 11).

**Table 1:** Optimization of NHC-catalyzed deaminative alkylation of aldehydes^a^

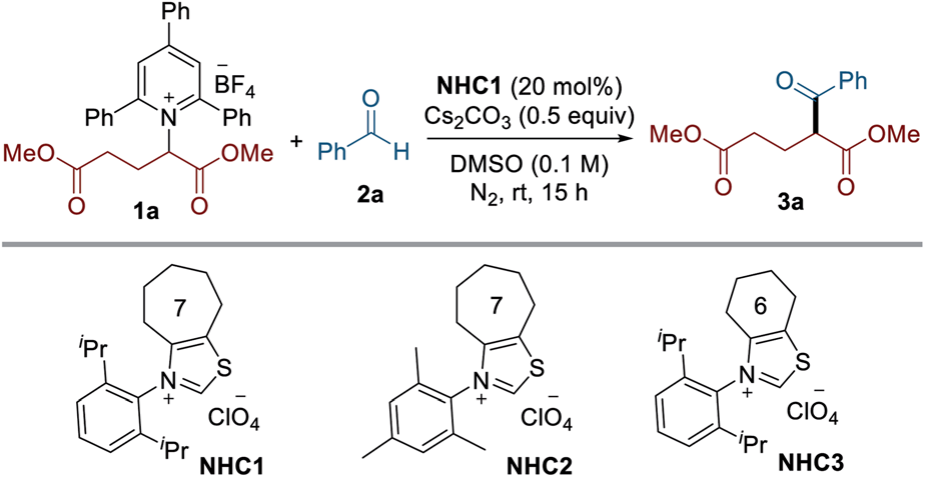		
Entry	Variation from the standard conditions	Yield^b^ (%)
1	None	74
2	MeCN instead of DMSO	36
3	1,2-DCE instead of DMSO	42
4	Toluene instead of DMSO	28
5	THF instead of DMSO	22
6	Water instead of DMSO	17
7	Without NHC catalyst	0
8	**NHC2** instead of **NHC1**	21
9	**NHC3** instead of **NHC1**	42
10	In the dark	72
11	With TEMPO	Trace

a Reaction conditions: **1a** (0.1 mmol), **2a** (1.5 equiv.), **NHC** (20 mol%) and Cs_2_CO_3_ (0.5 equiv.) in solvent (1.5 mL) at rt for 15 h under N_2_.

b Yields were determined by ^1^H NMR spectroscopy.

To examine the versatility and generality of the current methodology, the optimized conditions were then applied to various Katritzky salts derived from natural and unnatural amino acid precursors, as summarized in Table 2. In general, about 10–20% of the Katritzky salts **1** remained unreacted, and deamination products (∼10%) were observed as the major by-products generated from hydrogen atom abstraction (see Scheme S1 for details†). Exploration demonstrated that a series of substrates bearing electron-rich or electron-deficient groups, such as aliphatic, phenyl, thiomethyl, and ester groups, reacted readily under the optimized conditions to afford the desired cross-coupled products **3a–3h**. Pyridinium salts derived from phenylalanine and homophenylalanine also readily participated in the reaction to deliver the corresponding products **3g** and **3h**, respectively. The method was suitable with chloro-substituents to provide the corresponding product **3i**, thus enabling further derivatization. The Katritzky salts containing a phenolic group (tyrosine) and an indole group (tryptophan) were also well tolerated to produce ketone products **3j** and **3o**, respectively. Leucine derivatives bearing a methyl ester group and a 2-naphthyl amide group could be employed in this transformation to afford the desired products **3l** and **3m**. The structure of **3m** was unambiguously confirmed by X-ray crystallographic analysis.^18^ The Cbz-protected amine group was intact under the standard conditions to provide the desired product **3n**, thus enabling post-transformation. An allyl group was compatible with this reaction and produced the corresponding product **3k**. Moreover, the current method can also be extended to Katritzky salts derived from non-amino acids such as 4-amino-1-Boc-piperidine, 4-aminotetrahydropyran, and 2-aminoindane to afford the desired products **3p**, **3q**, and **3r**, respectively. In addition, the current method was suitable for the late-stage modification of biologically relevant molecules such as DOPA and Tamiflu derivatives (**3s** and **3t**). Peptides are one of the most important classes of biomolecules and have gained attention as therapeutic agents.^19^ In this context, site-selective late-stage modification of peptides holds vast potential for chemical biology and drug discovery by expanding the druggable target space. Various Katritzky salts derived from dipeptides were investigated under the optimal reaction conditions. To our delight, the strategy was successfully applied to dipeptides as exemplified by **3u** (Phe–Ala), **3v** (Phe–Phe), **3w** (Met–Gly) , and **3x** (Leu–Gly). The structure of **3y** from the Ala–Phe peptide was assigned by X-ray crystallographic analysis.^18^ Importantly, its excellent performance was further demonstrated by the tolerance of the more complex setting of peptides, such as tri- and tetra-peptides (**3z–3ad**).

**Table 2:** Scope of amine and aldehyde substrates^a^

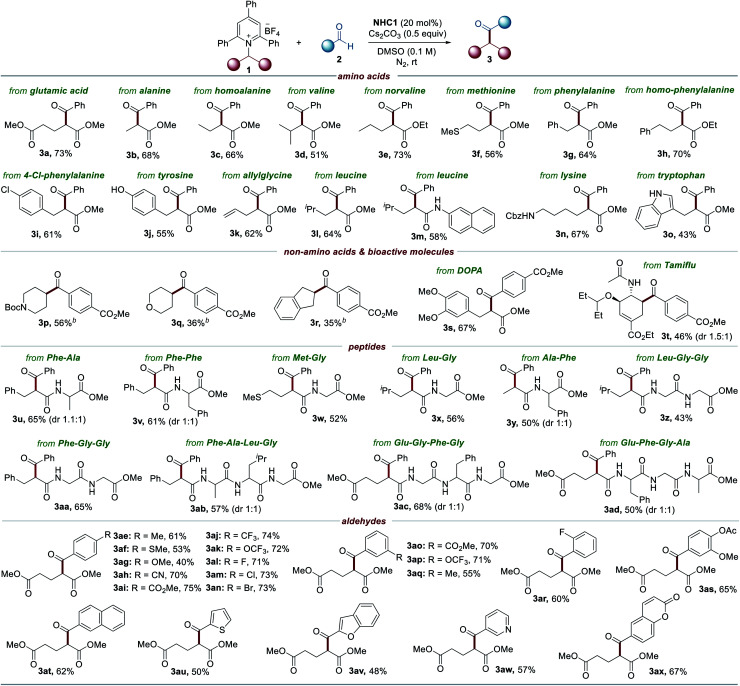

a Reactions were performed by using **1** (0.15 mmol), **2** (1.5 equiv.), **NHC1** (20 mol%), and Cs_2_CO_3_ (0.5 equiv.) in solvent (1.0 mL) at rt under N_2_ for 16–24 h. Yields of isolated products.

b 50 °C.

We subsequently evaluated the scope of aldehydes to extend the generality of this methodology. A series of aldehyde substrates bearing both electron-donating and electron-withdrawing groups on the aryl rings, such as methyl, methylthio, methoxy, cyano, ester, trifluoro, and trifluoromethoxy groups, worked well under the optimized conditions (**3ae–3ak**), as shown in Table 2.

The reaction was compatible with fluoro, chloro, and bromo substituents, thus offering an opportunity for the formation of further C–C or C–heteroatom bonds (**3al–3an**). *meta*- and *ortho*-substituents were tolerated under the standard conditions (**3ao–3ar**). In addition, a vanillin derivative was successfully transformed to provide the desired product **3as**. The scope of the reaction was further extended to other various (hetero)arenes such as naphthalene, thiophene, benzofuran, pyridine, and coumarin, to yield the corresponding products **3at–3ax**.

To further highlight the broad applicability of this reaction, we investigated whether the current NHC catalysis enables the vicinal alkyl carbofunctionalization of alkenes through a radical relay mechanism involving a SET from the enolate form and radical addition of the resultant alkyl radical to an alkene followed by radical–radical coupling. Remarkably, the synthetic utility was further verified by the three-component dicarbofunctionalization of olefins when alkenes were employed as coupling reagents. As highlighted in Table 3, a range of pyridinium salts were successfully reacted with 2-vinyl naphthalene under slightly modified reaction conditions in which DMSO and MeCN were used as cosolvents, leading to the selective formation of the corresponding products (**5a–5e**). Next, we assessed the applicability of this method with respect to the aldehyde scope and the reaction outcome was not significantly affected by the substitution pattern on the aldehyde (**5f–5j**). Additionally, it was possible to expand the scope of the method with vinyl arenes containing both electron-rich and electron-deficient functional groups (**5k–5p**).

**Table 3:** Substrate scope of the three-component reaction^a^

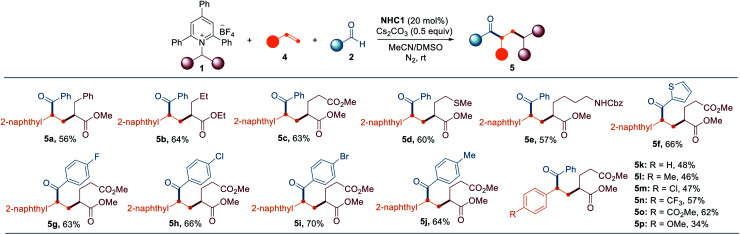

a Reactions were performed by using **1** (0.15 mmol), **2** (1.5 equiv.), **NHC1** (20 mol%), Cs_2_CO_3_ (0.5 equiv.) and DMSO/MeCN (1 : 1) at rt under N_2_ for 16–24 h. Yields of isolated products (dr 1 : 1).

**Fig. 1:**
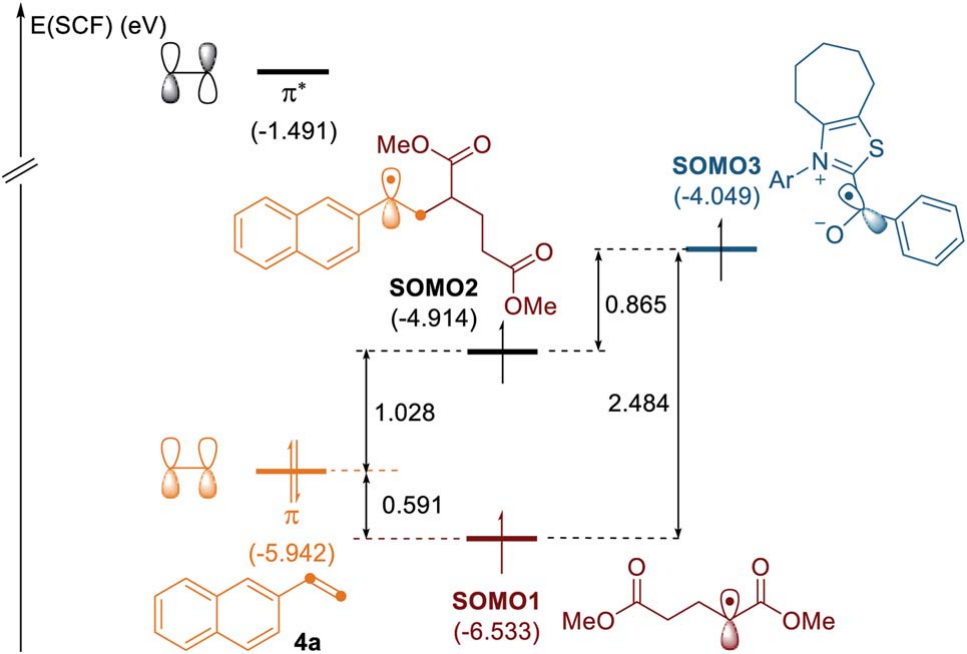
MO diagram of the alkyl radical interactions with alkene and ketyl radicals. Energies are given in eV.

To gain some insights into the selectivity of the radical cascades, we investigated the frontier molecular orbitals (FMOs) by conducting quantum chemical calculations based on density functional theory (DFT), as depicted in Fig. 1.^20^ The singly occupied molecular orbital (SOMO) of the alkyl radical is located at −6.53 eV. While the highest occupied molecular orbital (HOMO) of alkene **4a** is at −5.94 eV, the SOMO of the ketyl radical is found at −4.05 eV. The SOMO of the alkyl radical is closest and well matched with the occupied π orbital of the alkene, thus favoring the radical–olefin interaction. The SOMO energy level of the resultant benzyl radical is located at −4.91 eV, which reacts with the ketyl radical to afford three-component products, which is in good agreement with the experimental observations.

**Scheme 2:**
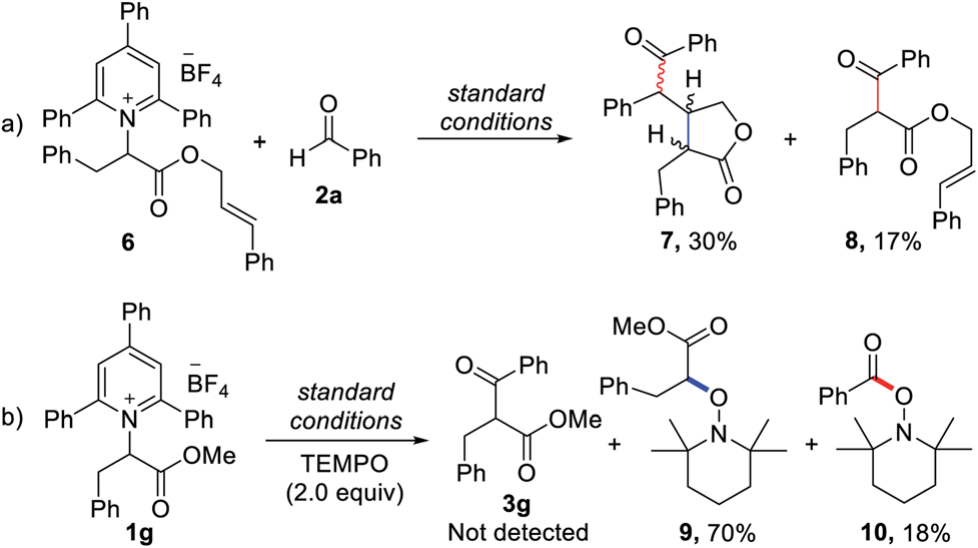
Control experiments.

To elucidate the reaction pathway, we conducted several control experiments (Scheme 2). First, we observed that reactions with a Katritzky salt bearing an internal alkene produced cyclized lactone compound **7** along with the directly coupled product **8** (Scheme 2a). Next, when TEMPO was added under the standard conditions for radical-trapping experiments, a considerable amount of alkyl and ketyl radical-trapped TEMPO adducts **9** and **10** was isolated. Under these conditions, the deaminative cross-coupled product **3g** was not detected by the measurement of HRMS and NMR with unpurified reaction mixtures (Scheme 2b). These two control experiments demonstrated that alkyl radicals from the deaminative process and carbonyl-carbon radicals are present in this reaction system. In addition, we used cyclic voltammetry to measure the pyridinium salt **1a**, and the reduction potential of **1a** was −0.60 V *vs.* SCE (see the ESI†), indicating that the enolate form of the Breslow intermediate (*E*^0^_ox_ = −0.97 V *vs.* the SCE)^14^ may attain sufficient reduction potential for Katritzky salts.

**Fig. 2:**
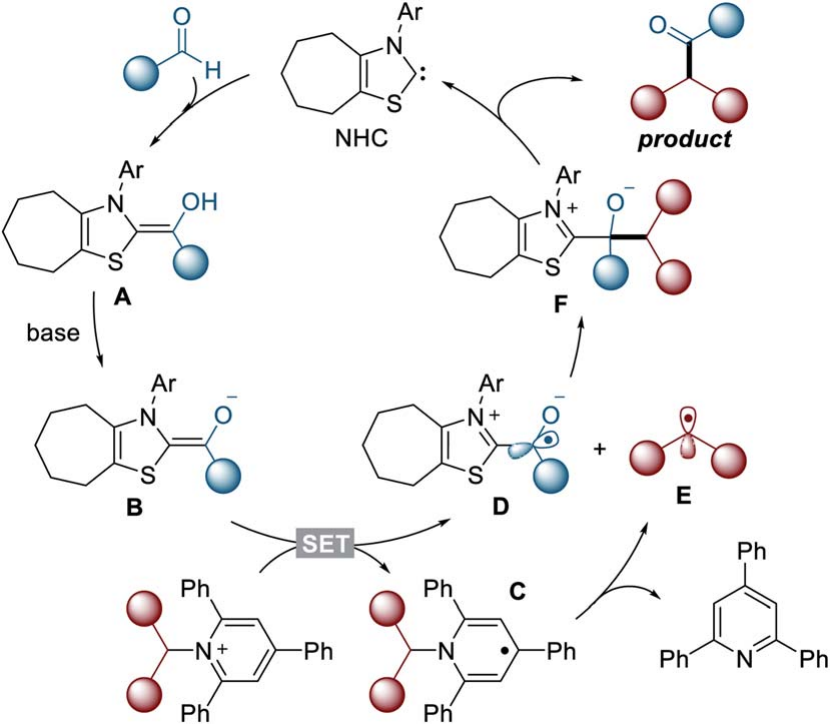
Proposed reaction mechanism.

Based on the above observations, a plausible mechanism for the current reactions is shown in Fig. 2. Initially, the aldehyde reacts with NHC to yield neutral Breslow intermediate **A**. The enolate form **B**, generated after deprotonation, functions as a single-electron reductant, which enables a SET reduction of the Katritzky salt to produce dihydropyridine radical **C** and ketyl radical **D**. Subsequently, dihydropyridine radical **C** undergoes fragmentation to generate alkyl radical **E** and pyridine. The alkyl radical **E** ultimately engages in radical–radical coupling with the carbonyl-carbon radical **D** to form the desired product and regenerates the NHC catalyst.

## Conclusions

In summary, we have reported an NHC-catalyzed deaminative radical–radical coupling strategy between readily available Katritzky salts and aldehydes under mild and metal-free conditions. The Katritzky salt behaves as a single-electron oxidant capable of generating an alkyl radical enabled by the reduction of the enolate form of a Breslow intermediate, offering a new opportunity for the rapid modification of a series of amino compounds. Moreover, the operational ease and broad functional group tolerance allow for the modification of a series of peptide feedstocks. The broad utility of the current versatile platform has been further verified by the application to the three-component dicarbofunctionalization of olefins through a radical relay.

## Conflicts of interest

There are no conflicts to declare.

## Supplementary Material

SC-011-D0SC00225A-s001

SC-011-D0SC00225A-s002
