# Supplementary material for: N-Heterocyclic carbene-catalyzed deaminative cross-coupling of aldehydes with Katritzky pyridinium salts
Source: Chem Sci. 2020 Feb 26;11(12):3192–7. doi: 10.1039/d0sc00225a (PMC8157273; doi:10.1039/d0sc00225a)
Supplement: SC-011-D0SC00225A-s001 [file SC-011-D0SC00225A-s001.pdf]

Electronic Supplementary Material (ESI) for Chemical Science.
This journal is © The Royal Society of Chemistry 2020

Supporting Information

N-Heterocyclic Carbene-Catalyzed Deaminative Cross-Coupling of Aldehydes with Katritzky Pyridinium Salts

*Inwon Kim, Honggu Im, Hyeonyeong Lee and Sungwoo Hong**

Department of Chemistry, Korea Advanced Institute of Science and Technology (KAIST), and Center for Catalytic Hydrocarbon Functionalizations, Institute for Basic Science (IBS), Daejeon, 34141, Korea

I. General Methods and Materials S2

II. Experimental Procedure S2

III. Cyclic Voltammetry S3

IV. Computational Study S7

V. Compound Characterizations S10

Appendix I

Spectral Copies of ^{1}H-, ^{13}C- and ^{19}F-NMR Data Obtained in this Study S49

Appendix II

Crystallographic Data for 1a, 1g, 3m and 3y S153

Appendix III

DFT Calculation Data S175

I. General Methods and Materials.

Unless stated otherwise, reactions were performed in flame-dried glassware. Analytical thin layer chromatography (TLC) was performed on precoated silica gel 60 F^{254} plates and visualization on TLC was achieved by UV light (254 and 365 nm). Flash column chromatography was performed on silica gel (400-630 mesh) or a Combi*Flash*® R_f^+ system with Redi*Sep*® R_f silica columns (230-400 mesh) using a proper eluent. ^{1}H NMR was recorded on Brucker Avance 400 MHz or Agilent Technologies DD2 600 MHz. Chemical shifts were quoted in parts per million (ppm) referenced to the appropriate solvent peak or 0.0 ppm for tetramethylsilane. The following abbreviations were used to describe peak splitting patterns when appropriate: br = broad, s = singlet, d = doublet, t = triplet, q = quartet, m = multiplet, dd = doublet of doublet, td = triplet of doublet, ddd = doublet of doublet of doublet. Coupling constants, *J*, were reported in hertz unit (Hz). ^{13}C NMR was recorded on Brucker Avance 100 MHz or Agilent Technologies DD2 150 MHz and was fully decoupled by broad band proton decoupling. Chemical shifts were reported in ppm referenced to the centerline of a triplet at 77.0 ppm of $CDCl_3$. ^{19}F NMR was recorded on Brucker Avance (375MHz). High-resolution mass spectra were obtained by using EI or FAB method from Korea Basic Science Institute (Daegu) or ESI from KAIST Research Analysis Center (Daejeon). Commercial grade reagents and solvents were used without further purification except as indicated below.

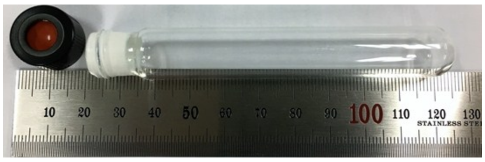

Figure S1. Reaction test tube (12 mL, 15 mm X 100 mm).

II. Experimental Procedure

Representative procedure for deaminative cross-coupling of aldehydes with Katritzky salts (GP1)

Reactions were conducted in a test tube (12 mL) sealed with PTFE/rubber septa. 1-(1,5-dimethoxy-1,5-dioxopentan-2-yl)-2,4,6-triphenylpyridin-1-ium tetrafluoroborate (**1a**) (83.0 mg, 0.15 mmol), benzaldehyde (**2a**) (23.9 mg, 0.225 mmol), 3-(2,6-diisopropylphenyl)-5,6,7,8-tetrahydro-4H-cyclohepta[d]thiazol-3-ium perchlorate (**NHC1**) (12.4 mg, 0.03 mmol), and Cs_2CO_3 (24.4 mg, 0.075 mmol) were combined in dry DMSO (1.5 mL) under N_2 atmosphere. The resulting mixture was stirred at rt. The reaction mixture was monitored by TLC using (EtOAc/hexanes = 1:4) as the mobile phase. After disappearance of starting material, the reaction mixture was diluted and extracted with ethylacetate (3 times) and dried over sodium sulfate. After removal of solvent, the residue was purified by flash chromatography on silica gel (EtOAc/hexanes = 1:4) to give a desired product compound **3a** as colorless oil.

Representative procedure for three-component deaminative cross-coupling of aldehydes with Katritzky salts (GP2)

Reactions were conducted in a test tube (12 mL) sealed with PTFE/rubber septa. 1-(1,5-dimethoxy-1,5-dioxopentan-2-yl)-2,4,6-triphenylpyridin-1-ium tetrafluoroborate (**1a**) (83.0 mg, 0.15 mmol), benzaldehyde (**2a**) (23.9 mg, 0.225 mmol), 3-(2,6-diisopropylphenyl)-5,6,7,8-tetrahydro-4H-cyclohepta[d]thiazol-3-ium perchlorate (**NHC1**) (12.4 mg, 0.03 mmol), 2-vinylnaphthalene (**4a**) (69.4 mg, 0.45 mmol) and Cs_2CO_3 (24.4 mg, 0.075 mmol) were combined in co-solvent system with dry DMSO (0.75 mL) and dry MeCN (0.75 mL) under N_2 atmosphere. The resulting mixture was stirred at rt. The reaction mixture was monitored by TLC using (EtOAc/hexanes = 1:6) as the mobile phase. After disappearance of starting material, the reaction mixture was diluted and extracted with ethylacetate (3 times) and dried over sodium sulfate. After removal of solvent, the residue was purified by flash chromatography on silica gel (EtOAc/hexanes = 1:6) to give a desired product compound **5c** as colorless oil.

Preparation of Katritzky salts (GP3)

The amine hydrochloride (1.0 equiv) was added to a 75 mL sealed tube and ethanol (1.0 M) and triethyl amine (1.2 equiv) were added. The resulting suspension was stirred for 30 min at room temperature. Triphenylpyrylium tetrafluoroborate (1.0 equiv) was added, the tube sealed and stirred for overnight at 92 °C. For removing water-soluble impurities such as TEA salt, reaction mixture was washed with water and the collected organic layer was concentrated. The corresponding salts were recrystallized with CH_2Cl_2 and diethyl ether or purified by flash column chromatography with CH_2Cl_2:acetone (9:1).

Preparation of Katritzky salts (GP4)

Amino acid methyl ester (1.0 equiv), 2,4,6-triphenylpyrylium tetrafluoroborate (1.0 equiv), and activated 4 Å MS (0.5 g per 1.0 mmol) were added to a round-bottomed flask. The flask was closed with a septum. CH_2Cl_2 (0.5 M) was added and then TEA (2.0 equiv) was added. The reaction mixture was stirred for 30 min at room temperature. Acetic acid (2.0 equiv) was added. The mixture was stirred for 5 h at room temperature. The mixture was filtered through a short celite pad. The flask and celite were rinsed with CH_2Cl and concentrated. The product was purified by silica gel chromatography with CH_2Cl_2:acetone (9:1).

III. Cyclic Voltammetry

Cyclic voltammetry was measured by a potentiostat (CH instrument, 600E) with conventional three electrode system (reference electrode: Ag/Ag^+, working electrode: Glassy carbon, counter electrode: Pt wire, supporting electrolyte: 0.1 M NBu_4PF_6 CH_3CN) at 50 mV/sec of scan rate.

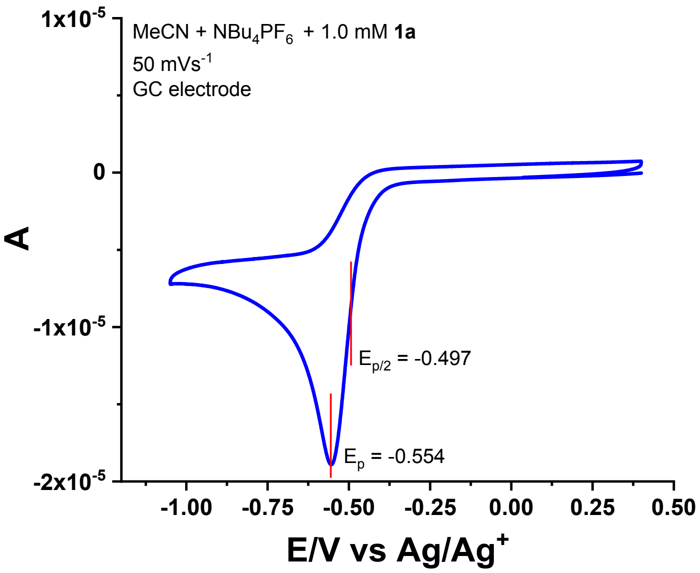

Figure S2. CV of **1a** (1 mM in CH_3CN)

Control experiments

Table S1. Base screening

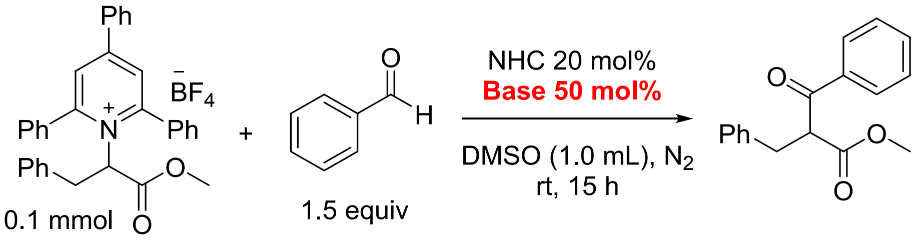

Base	Product
Li_2CO_3	44%
Na_2CO_3	49%
K_2CO_3	53%
Cs_2CO_3	61%
K_3PO_4	33%
DBU	24%
DIPEA	16%
N-methylpiperidien	12%

Table S2. Effect of base equivalent

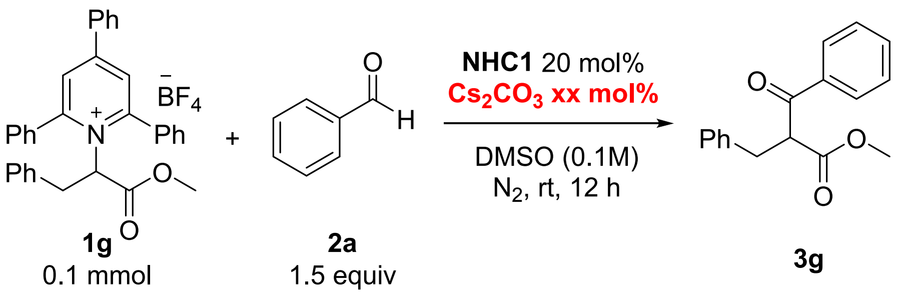

**Base**	0 mol%	20 mol%	30 mol%	40 mol%	50 mol%	100 mol%
**Product yield**	No reaction	18%	26%	45%	64%	53%

Scheme S1. Model reaction and NMR yield of each materials

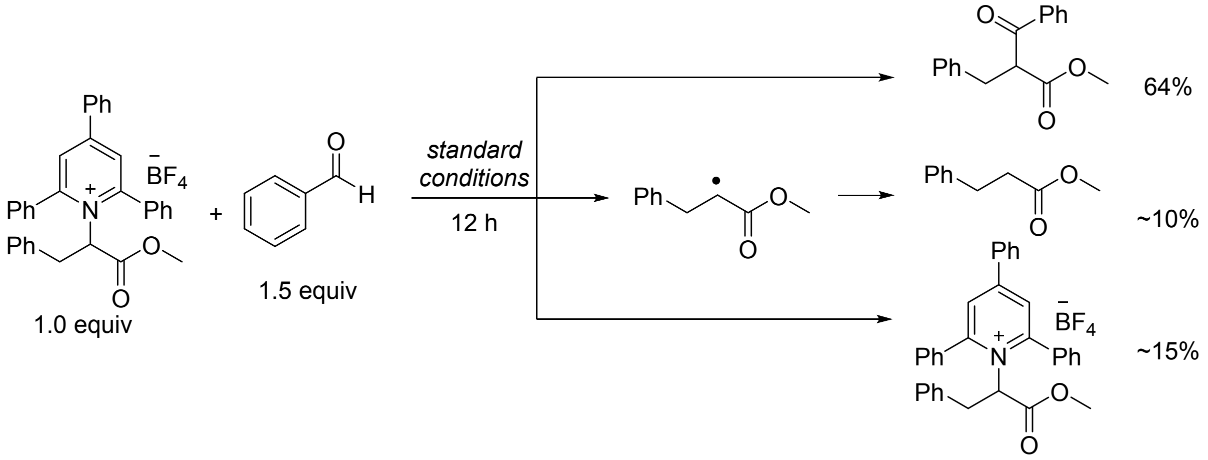

Table S3. Time-course studies for deaminative cross-coupling

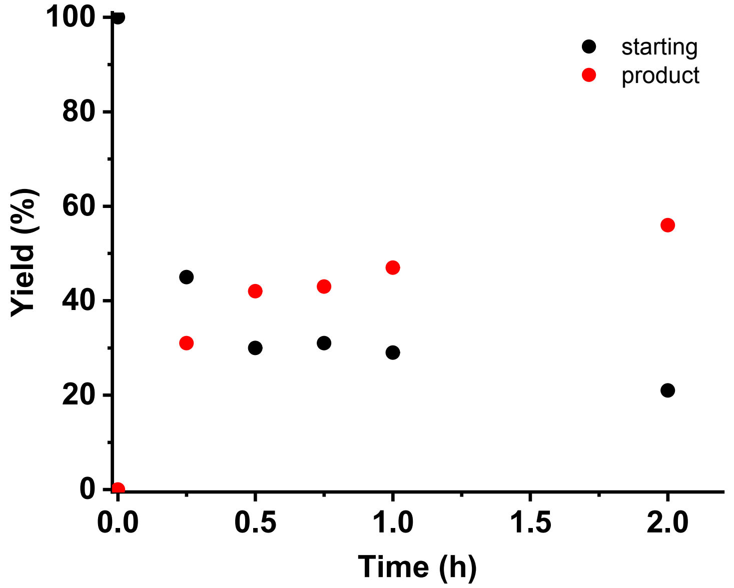

Figure S3. GC-MS analysis of crude reaction mixture

```
File        :D:\MassHunter\GCMS\3\data\KIW-Prof.Hong\200213-1.D
Operator    : KIW
Acquired    : 13 Feb 2020  16:30      using AcqMethod KIW-03.M
Instrument  :    GCMSD
Sample Name: KIW-crude
Misc Info   :
Vial Number: 11
```

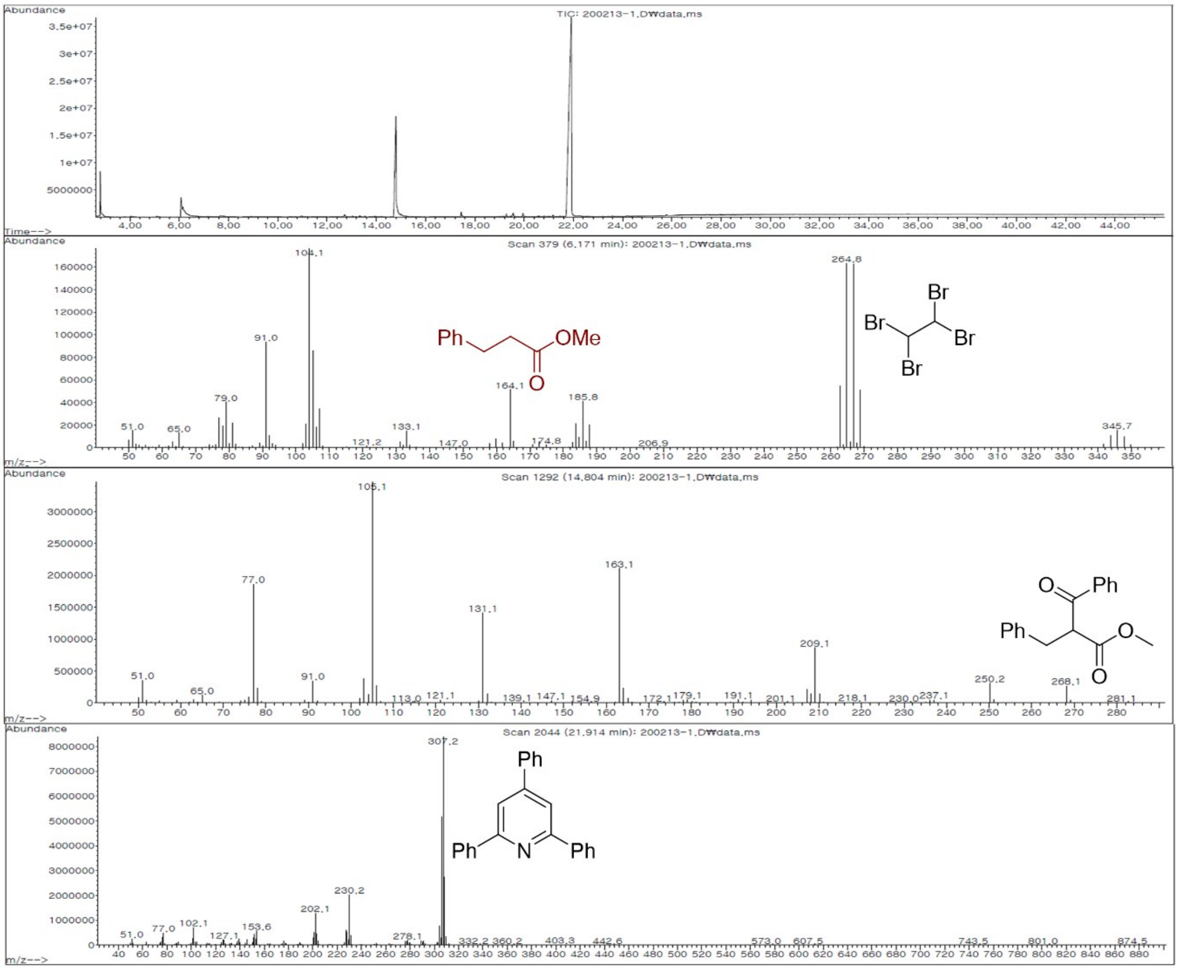


```
File        :D:\MassHunter\GCMS\3\data\KIW-Prof.Hong\200213-2.D
Operator    : KIW
Acquired    : 13 Feb 2020  17:32      using AcqMethod KIW-03.M
Instrument  :    GCMSD
Sample Name: KIW-byproduct
Misc Info   :
Vial Number: 12
```

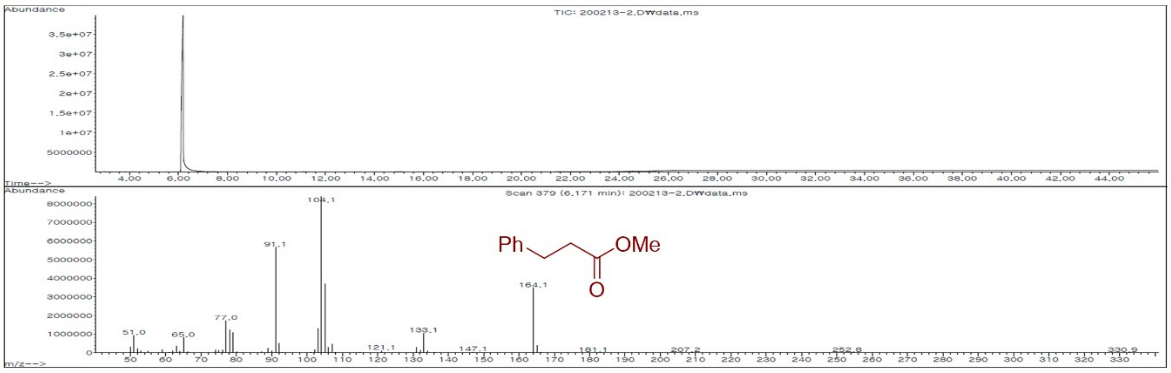

IV. Computational Study

All calculations were conducted using density functional theory (DFT)[S1] as implemented in the Jaguar 9.1 suite[S2] of ab initio quantum chemistry programs with B3LYP-D3 levels of theory.[S3] Geometry optimizations were proceeded using the LACVP** basis set. The energies of the optimized moieties were recalculated with the high quality triple-ζ basis set cc-pVTZ(-f)[sS4] Analytical vibrational frequencies within the harmonic approximation were calculated using the same level of theory as the geometry optimization to confirm proper convergence to well-defined minima or saddle points on the potential energy surface. Solvation energies were calculated using a self-consistent reaction field (SCRF)[S5-S7] approach based on accurate numerical solutions of the Poisson-Boltzmann equation and were performed at the optimized gas-phase geometry with the dielectric constant of ε = 46.48 for dimethylsulfoxide. As is the case for all continuum models, the solvation energies are subject to the empirical parametrization of the atomic radii that are used to generate the solute surface. The quadratic synchronous transit search method (QST) was utilize to locate the corresponding transition states.[S8] The Gibbs free energies in solution phase G(sol) was computed with the following equations:

$$G(sol) = G(gas) + G^{solv} \quad (1)$$

$$G(gas) = H(gas) - TS(gas) \quad (2)$$

$$H(gas) = E(SCF) + ZPE \quad (3)$$

$$\Delta E(SCF) = \Sigma E(SCF) \text{ for products} - \Sigma E(SCF) \text{ for reactants} \quad (4)$$

$$\Delta G(sol) = \Sigma G(sol) \text{ for products} - \Sigma G(sol) \text{ for reactants} \quad (5)$$

G(gas) is the free energy in gas phase; G^{solv} is the free energy of solvation; H(gas) is the enthalpy in gas phase; T is the temperature (298.15K); S(gas) is the entropy in gas phase; E(SCF) is “raw” electronic energy as computed from the SCF procedure which is the self-consistent field energy, and ZPE is the zero point energy. The entropy we refer is specifically vibrational/rotational/translational entropy of the solute(s), and the entropy of the solvent is implicitly comprised in the continuum solvation model.

Figure S4. Energy profile of SET process

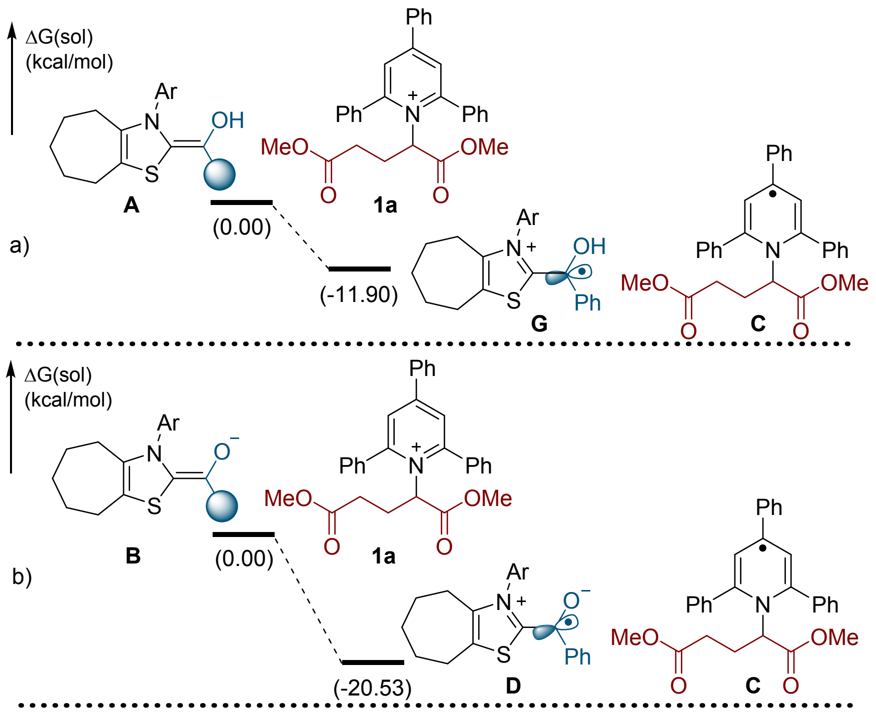

References

[S1] Parr, R. G.; Yang, W., Density Functional Theory of Atoms and Molecules, Oxford University Press, New York, **1989**.

[S2] Bochevarov, A. D.; Harder, E.; Hughes, T. F.; Greenwood, J. R.; Braden, D. A.; Philipp, D. M.; Rinaldo, D.; Halls, M. D.; Zhang, J.; Friesner, R. A., *Int. J. Quantum Chem.* **2013**, *113*, 2110.

[S3] Becke, A. D., *J. Chem. Phys.* **1993**, *98*, 1372.

[S4] Dunning, T. D., *J. Chem. Phys.* **1989**, *90*, 1007.

[S5] Marten, B.; Kim, K.; Cortis, C.; Friesner, R. A.; Murphy, R. B.; Ringnalda, M. N.; Stikoff, D.; Honig, B., *J. Phys. Chem*. **1996**, *100*, 11775.

[S6] Friedrichs, M.; Zhou, R.; Edinger, S. R.; Friesner, R. A., *J. Phys. Chem.* **1999**, *103*, 3057.

[S7] Edinger, S. R.; Cortis, C.; Shenkin, P. S.; Friesner, R. A., *J. Phys. Chem*. **1997**, *101*, 1190.

[S8] Govind, N.; Petersen, M.; King-Smith, D.; Andzelm, J., *Comput. Mater. Sci.* **2003**, *28*, 250.

VIII. Compound Characterizations

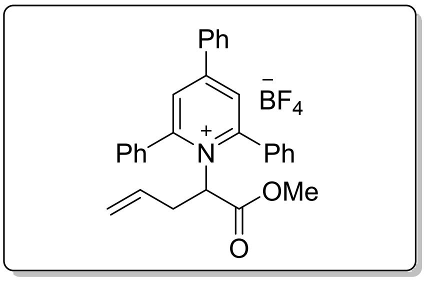

1-(1-methoxy-1-oxopent-4-en-2-yl)-2,4,6-triphenylpyridin-1-ium tetrafluoroborate (1k). Prepared according to **GP3.** 1.0 mmol of amino acid was used for the limiting reagent. Purified by flash chromatography on silica gel (CH_2Cl_2/acetone = 9:1). Ivory solid (281.0 mg, 55%). ^{1}H NMR (600 MHz, Chloroform-*d*) δ 7.94 (s, 2H), 7.86 – 7.81 (m, 2H), 7.78 – 7.48 (m, 13H), 5.46 – 5.32 (m, 2H), 5.04 – 4.87 (m, 2H), 3.68 (s, 3H), 2.95 – 2.78 (m, 1H), 2.52 – 2.32 (m, 1H). ^{13}C NMR (150 MHz, Chloroform-*d*) δ 168.0, 157.0, 133.8, 132.4, 132.0, 131.5, 129.7, 129.3, 129.1, 128.5, 127.8, 119.7, 68.4, 53.6, 53.6, 35.6. ^{19}F NMR (375MHz, Chloroform-*d*) δ -153.05, -153.11. HRMS (FAB) m/z calcd. for $C_{29}H_{26}NO_2^+$ [M-BF_4^-]$^+$: 420.1958, found : 420.1967

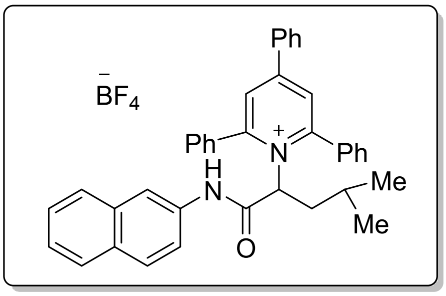

1-(4-methyl-1-(naphthalen-2-ylamino)-1-oxopentan-2-yl)-2,4,6-triphenylpyridin-1-ium tetrafluoroborate (1m). Prepared according to **GP3.** 1.0 mmol of amino acid was used for the limiting reagent. Purified by flash chromatography on silica gel (Methanol/CH_2Cl_2 = 1:20). Off-white solid (468.9 mg, 74%). ^{1}H NMR (400 MHz, Chloroform-*d*) δ 8.87 (s, 1H), 8.19 (d, *J* = 2.0 Hz, 1H), 7.96 (s, 2H), 7.80 (ddd, *J* = 16.0, 8.3, 5.8 Hz, 6H), 7.68 – 7.34 (m, 15H), 5.72 (dd, *J* = 8.3, 4.8 Hz, 1H), 2.30 (dd, *J* = 14.7, 8.2 Hz, 1H), 1.57 – 1.45 (m, 1H), 1.37 – 1.23 (m, 1H), 0.72 (d, *J* = 6.4 Hz, 3H), 0.44 (d, *J* = 6.4 Hz, 3H). ^{13}C NMR (100 MHz, Chloroform-*d*) δ 164.1, 157.5, 156.6, 134.9, 133.7, 133.6, 132.5, 132.4, 131.5, 130.9, 129.7, 129.2, 128.7, 128.4, 128.2, 128.0, 127.5, 126.2, 125.2, 120.3, 117.5, 69.3, 40.7, 26.1, 22.0, 21.0. ^{19}F NMR (375MHz, Chloroform-*d*) δ -150.5, -150.6. HRMS (FAB) m/z calcd. for $C_{39}H_{35}N_2O^+$ [M-BF_4^-]$^+$: 547.2744, found : 547.2750

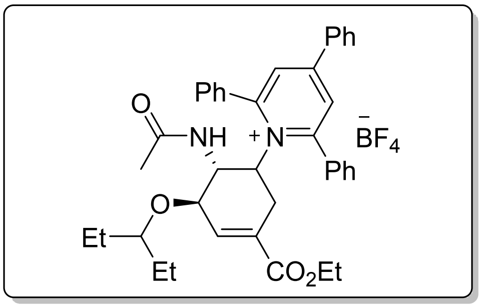

1-((5R,6R)-6-acetamido-3-(ethoxycarbonyl)-5-(pentan-3-yloxy)cyclohex-3-en-1-yl)-2,4,6-triphenylpyridin-1-ium tetrafluoroborate (1t). Prepared according to **GP3.** 1.0 mmol of amino acid was used for the limiting reagent. Purified by flash chromatography on silica gel (CH_2Cl_2/acetone = 9:1). Off-white solid (354.9 mg, 51%). ^{1}H NMR (600 MHz, Chloroform-*d*) δ 8.50 (s, 1H), 7.95 – 7.82 (m, 5H), 7.76 – 7.52 (m, 9H), 7.49 – 7.39 (m, 2H), 7.07 (d, *J* = 7.6 Hz, 1H), 6.45 (q, *J* = 1.8 Hz, 1H), 5.47 (dt, *J* = 11.1, 8.2 Hz, 1H), 4.29 (dt, *J* = 8.3, 2.3 Hz, 1H), 4.15 (q, *J* = 7.1 Hz, 2H), 3.88 (dt, *J* = 11.1, 7.9 Hz, 1H), 3.19 (p, *J* = 5.8 Hz, 1H), 3.04 (dq, *J* = 8.9, 2.1 Hz, 2H), 1.87 (s, 3H), 1.40 – 1.35 (m, 3H), 1.33 – 1.29 (m, 1H), 1.27 (t, *J* = 7.1 Hz, 3H), 0.80 – 0.74 (m, 6H). ^{13}C NMR (150 MHz, Chloroform-*d*) δ 172.5, 165.0, [160.7, 159.2], 155.9, 139.6, [133.25, 133.21], 133.1, 132.8, 132.0, 131.4, 130.2, 129.9, 129.6, 128.3, [126.1, 125.8], 83.0, 73.1, 68.3, 61.1, 55.9, 32.2, 26.0, 25.6, 22.8, 14.1, 9.4, 9.1. ^{19}F NMR (375MHz, Chloroform-*d*) δ -150.6, -150.7. HRMS (FAB) m/z calcd. for $C_{39}H_{43}N_2O_4^+$ $[M-BF_4^-]^+$: 603.3217, found : 603.3225

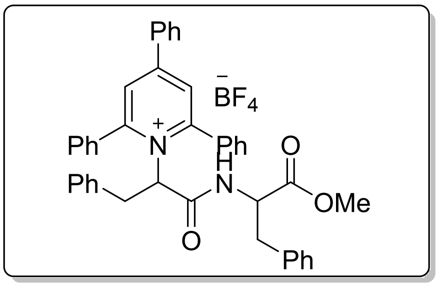

1-(1-((1-methoxy-1-oxo-3-phenylpropan-2-yl)amino)-1-oxo-3-phenylpropan-2-yl)-2,4,6-triphenylpyridin-1-ium tetrafluoroborate (1v). Prepared according to **GP3.** 1.0 mmol of amino acid was used for the limiting reagent. Purified by flash chromatography on silica gel ($MeOH/CH_2Cl_2$ = 1:100). Ivory solid (410.7 mg, 58%). ^{1}H NMR (600 MHz, Chloroform-*d*) δ 7.92 (d, J = 5.5 Hz, 2H), 7.82 (ddd, J = 7.1, 5.2, 1.7 Hz, 2H), 7.78 – 7.45 (m, 13H), 7.32 – 7.21 (m, 2H), 7.14 – 7.05 (m, 2H), 7.04 – 6.93 (m, 2H), 6.84 (d, J = 7.4 Hz, 1H), 6.65 (d, J = 7.4 Hz, 1H), 6.48 (d, J = 7.5 Hz, 1H), 6.40 (d, J = 7.6 Hz, 1H), [6.05 (d, J = 7.7 Hz, 0.4H), 5.92 (d, J = 7.5 Hz, 0.6H)], [5.77 (dd, J = 9.9, 3.1 Hz, 0.6H), 5.65 (dd, J = 10.0, 3.1 Hz, 0.4H)], 4.73 – 4.57 (m, 1H), 3.74 (s, 1.3H), 3.72 – 3.64 (m, 1H), 3.53 (s, 1.7H), [3.01 – 2.88 (m, 1.6H), 2.63 – 2.56 (m, 0.4H)], 2.55 – 2.41 (m, 1H). ^{13}C NMR (150 MHz, Chloroform-*d*) δ [170.7, 170.4], [168.0, 167.5], [156.9, 156.8], [136.2, 135.9], [135.4, 135.2], [134.3, 134.3], [133.3, 133.2], [132.2, 132.2], [131.4, 131.2], [129.8, 129.7], [129.6, 129.5], 129.2, 129.2, 128.8, 128.6, 128.6, 128.4, 128.2, [128.0, 127.8], [127.5, 127.0], [71.7, 71.4], [54.4, 54.1], [52.7, 52.5],

[37.6, 37.3]. ^{19}F NMR (375 MHz, Chloroform-*d*) δ -152.55, -152.60. HRMS (FAB) m/z calcd. for $C_{42}H_{37}N_2O_3^+$ [M-BF_4^-]$^+$: 617.2799, found : 617.2807

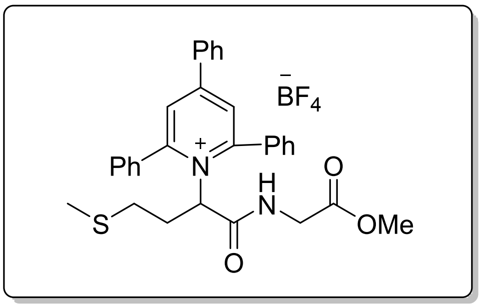

1-(1-((2-methoxy-2-oxoethyl)amino)-4-(methylthio)-1-oxobutan-2-yl)-2,4,6-triphenylpyridin-1-ium tetrafluoroborate (1w). Prepared according to **GP3.** 1.0 mmol of amino acid was used for the limiting reagent. Purified by flash chromatography on silica gel (CH_2Cl_2/acetone = 9:1). Ivory solid (393.7 mg, 66%). ^{1}H NMR (600 MHz, Chloroform-*d*) δ 7.88 (s, 2H), 7.80 – 7.75 (m, 2H), 7.69 (d, *J* = 7.4 Hz, 4H), 7.64 – 7.58 (m, 2H), 7.59 – 7.52 (m, 5H), 7.51 – 7.46 (m, 2H), 7.36 (t, *J* = 5.5 Hz, 1H), 5.63 (t, *J* = 5.8 Hz, 1H), 3.94 (d, *J* = 5.3 Hz, 2H), 3.70 (s, 3H), 2.26 – 2.15 (m, 3H), 1.95 – 1.87 (m, 1H), 1.85 (s, 3H). ^{13}C NMR (150 MHz, Chloroform-*d*) δ 169.1, 167.0, 157.5, 156.7, 133.6, 132.5, 132.4, 131.5, 129.7, 129.4, 129.2, 128.3, 128.0, 69.0, 52.1, 41.7, 31.2, 30.8, 15.2. ^{19}F NMR (375MHz, $CDCl_3$) δ -152.33, -152.38. HRMS (FAB) m/z calcd. for $C_{31}H_{31}N_2O_3S^+$ [M-BF_4^-]$^+$: 511.2050, found : 511.2053

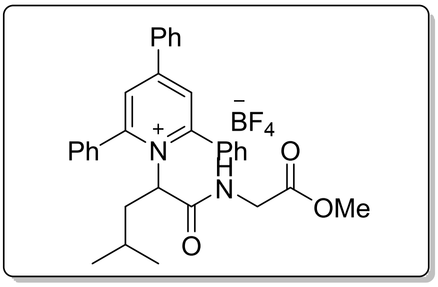

1-(1-((2-methoxy-2-oxoethyl)amino)-4-methyl-1-oxopentan-2-yl)-2,4,6-triphenylpyridin-1-ium tetrafluoroborate (1x). Prepared according to **GP3.** 1.0 mmol of amino acid was used for the limiting reagent. Purified by flash chromatography on silica gel (MeOH/CH_2Cl_2 = 1:100). Off-white solid). Ivory solid (417.3 mg, 72%). ^{1}H NMR (400 MHz, Chloroform-*d*) δ 7.93 (s, 2H), 7.84 – 7.79 (m, 2H), 7.73 – 7.62 (m, 4H), 7.63 – 7.49 (m, 10H), 7.28 (t, J = 5.7 Hz, 1H), 5.56 (t, J = 7.1 Hz, 1H), 4.03 – 3.94 (m, 2H), 3.73 (s, 3H), 1.89 – 1.71 (m, 1H), 1.51 – 1.37 (m, 1H), 1.21 – 1.10 (m, 1H), 0.60 (d, J = 6.5 Hz, 3H), 0.50 (d, J = 6.6 Hz, 3H). ^{13}C NMR (100 MHz, Chloroform-*d*) δ 169.4, 168.0, 156.6, 133.7, 132.8, 132.6, 131.8, 129.9, 129.6, 129.4, 128.5, 128.1, 69.3, 52.3, 42.0, 39.3, 26.0, 21.7, 21.6. ^{19}F NMR (375 MHz, Chloroform-*d*) δ -152.52, -152.58. HRMS (FAB) m/z calcd. for $C_{32}H_{33}N_2O_3^+$ [M-BF_4^-]$^+$: 493.2486, found : 493.2489

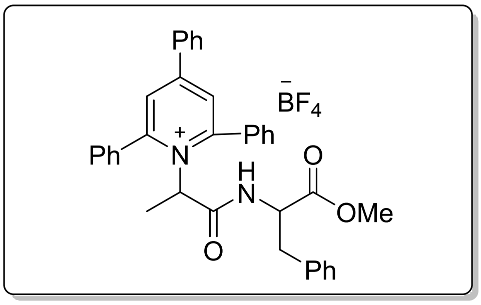

1-(1-((1-methoxy-1-oxo-3-phenylpropan-2-yl)amino)-1-oxopropan-2-yl)-2,4,6-triphenylpyridin-1-ium tetrafluoroborate (1y). Prepared according to **GP3.** 1.0 mmol of amino acid was used for the limiting reagent. Purified by flash chromatography on silica gel (CH_2Cl_2/acetone = 9:1). Yellow solid. (429.2 mg, 68%). ^{1}H NMR (600 MHz, Chloroform-*d*) δ 7.85 (d, *J* = 3.3 Hz, 2H), 7.79 – 7.72 (m, 2H), 7.71 – 7.59 (m, 4H), 7.58 – 7.42 (m, 9H), 7.24 – 7.15 (m, 3H), 7.05 – 6.98 (m, 1H), 6.98 – 6.92 (m, 1H), [6.39 (d, *J* = 7.2 Hz, 0.45H), 6.22 (d, *J* = 7.6 Hz, 0.51H)], [5.55 (q, *J* = 7.2 Hz, 0.56H), 5.47 (q, *J* = 7.1 Hz, 0.46H)], 4.66 – 4.52 (m, 1H), [3.69 (s, 1.45H), 3.67 (s, 1.50H)], 3.12 – 3.01 (m, 1H), 3.00 – 2.91 (m, 1H), [1.37 (d, *J* = 7.2 Hz, 1.51H), 1.34 (d, *J* = 7.2 Hz, 1.47H)]. ^{13}C NMR (150 MHz, Chloroform-*d*) δ [170.8, 170.7], [168.3, 167.8], [157.23, 157.18], [156.3, 156.2], [135.8, 135.6], [133.98, 133.96], [133.0, 132.9], 132.1, [131.1, 131.0], 129.6, 128.99, [129.03, 128.95], [128.7, 128.5], 128.3, 127.6, [127.3, 127.0], [65.8, 65.5], [54.5, 54.2], 52.4, [37.3, 37.1], [17.0, 16.7]. ^{19}F NMR (375MHz, Chloroform-*d*) δ -152.18, -152.23. HRMS (FAB) m/z calcd. for $C_{36}H_{33}N_2O_3^+$ [M-BF_4^-]$^+$: 541.2486, found : 541.2494

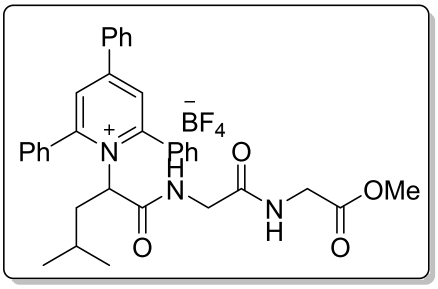

1-(1-((2-((2-methoxy-2-oxoethyl)amino)-2-oxoethyl)amino)-4-methyl-1-oxopentan-2-yl)-2,4,6-triphenylpyridin-1-ium tetrafluoroborate (1z). Prepared according to **GP3.** 1.0 mmol of amino acid was used for the limiting reagent. Purified by flash chromatography on silica gel (CH_2Cl_2/MeOH = 20:1). Ivory solid (602.1 mg, 47%). ^{1}H NMR (600 MHz, Chloroform-*d*) δ 7.93 (s, 2H), 7.87 – 7.77 (m, 2H), 7.65 – 7.52 (m, 12H), 7.15 (t, J = 5.7 Hz, 1H), 7.03 (t, J = 5.4 Hz, 1H), 5.62 (t, J = 7.3 Hz, 1H), 4.06 (dd, J = 16.8, 5.8 Hz, 1H), 4.04 – 3.93 (m, 2H), 3.87 (dd, J = 16.7, 4.8 Hz, 1H), 3.70 (s, 3H), 1.69 – 1.58 (m, 1H), 1.52 – 1.40 (m, 1H), 1.20 – 1.09 (m, 1H), 0.61 (d, J = 6.5 Hz, 3H), 0.53 (s, 3H). ^{13}C NMR (150 MHz, Chloroform-*d*) δ 170.1, 168.6, 168.0, 156.4, 133.5, 132.8, 132.7, 131.9, 130.00, 129.6, 129.5, 129.4, 128.4, 69.4, 52.3, 43.6, 41.3, 38.9, 25.8, 21.9, 21.5. ^{19}F NMR (375 MHz, Chloroform-*d*) δ -152.97, -152.92. HRMS (FAB) m/z calcd. for $C_{34}H_{36}N_3O_4^+$ [M-BF_4^-]$^+$: 550.2700, found : 550.2709

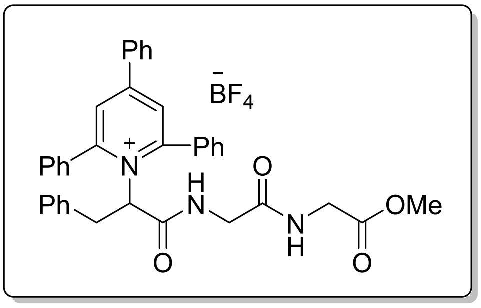

1-(1-((2-((2-methoxy-2-oxoethyl)amino)-2-oxoethyl)amino)-1-oxo-3-phenylpropan-2-yl)-2,4,6-triphenylpyridin-1-ium tetrafluoroborate (1aa). Prepared according to **GP4.** 1.0 mmol of amino acid was used for the limiting reagent. Purified by flash chromatography on silica gel (CH_2Cl_2/acetone = 9:1). Ivory solid (356.5 mg, 53%). ^{1}H NMR (600 MHz, Chloroform-*d*) δ 7.88 (s, 2H), 7.85 – 7.34 (m, 15H), 7.22 – 7.18 (m, 1H), 7.14 (t, *J* = 7.4 Hz, 2H), 7.07 – 6.99 (m, 1H), 6.94 – 6.83 (m, 1H), 6.77 – 6.57 (m, 2H), 5.84 (dd, *J* = 8.2, 6.2 Hz, 1H), 4.04 (dd, *J* = 17.9, 5.7 Hz, 1H), 3.99 – 3.90 (m, 2H), 3.81 (dd, *J* = 16.5, 5.4 Hz, 1H), 3.71 (s, 3H), 3.20 (qd, *J* = 14.8, 7.3 Hz, 2H). ^{13}C NMR (100 MHz, Chloroform-*d*) δ 170.0, 168.1, 167.0, 157.9, 156.3, 134.8, 133.3, 132.7, 132.2, 131.7, 129.9, 129.6, 129.2, 129.2, 128.7, 128.3, 127.8, 127.6, 71.4, 52.2, 43.7, 41.1, 36.2. ^{19}F NMR (375MHz, Chloroform-*d*) δ -151.7, -151.8. HRMS (FAB) m/z calcd. for $C_{37}H_{34}N_3O_4^+$ [M-BF_4^-]$^+$: 584.2544, found : 584.2548

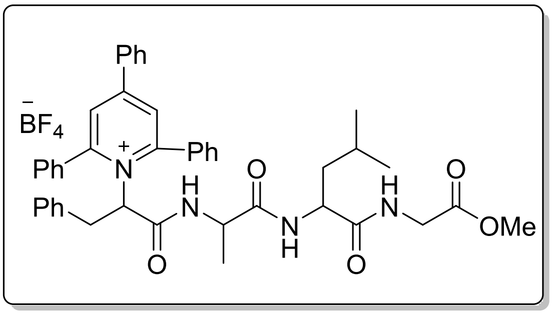

1-(1-((1-((1-((carboxymethyl)amino)-4-methyl-1-oxopentan-2-yl)amino)-1-oxopropan-2-yl)amino)-1-oxo-3-phenylpropan-2-yl)-2,4,6-triphenylpyridin-1-ium tetrafluoroborate (1ab). Prepared according to **GP4.** 1.0 mmol of amino acid was used for the limiting reagent. Purified by flash chromatography on silica gel (CH_2Cl_2/MeOH = 30:1). Ivory solid (505.6 mg, 63%). ^{1}H NMR (600 MHz, Chloroform-*d*) δ 7.90 – 7.84 (m, 2H), 7.80 – 7.31 (m, 15H), 7.18 – 7.06 (m, 3H), 7.05 – 6.65 (m, 5H), 5.96 – 5.54 (m, 1H), 4.47 – 4.35 (m, 1H), 4.32 – 4.17 (m, 1H), 4.00 – 3.79 (m, 2H), 3.64 – 3.62 (m, 3H), 3.34 – 3.20 (m, 1H), 3.15 – 2.83 (m, 1H), 1.77 – 1.66 (m, 2H), 1.66 – 1.57 (m, 1H), 1.40 – 1.25 (m, 3H), 1.02 – 0.78 (m, 6H). ^{13}C NMR (150 MHz, Chloroform-*d*) δ 172.2, 171.3, 169.9, 166.2, 156.4, 135.2, 133.5, 132.5, 131.5, 129.7, 129.5, 129.3, 129.1, 128.5, 128.3, 71.4, 52.2, 52.1, 50.3, 41.1, 40.6, 36.7, 24.8, 22.9, 21.9, 17.7. ^{19}F NMR (375MHz, Chloroform-*d*) δ -150.76, -150.82. HRMS (ESI)

m/z calcd. for $C_{43}H_{45}N_4O_5^+$ $[M-BF_4^-]^+$: 711.3541, found : 711.3544

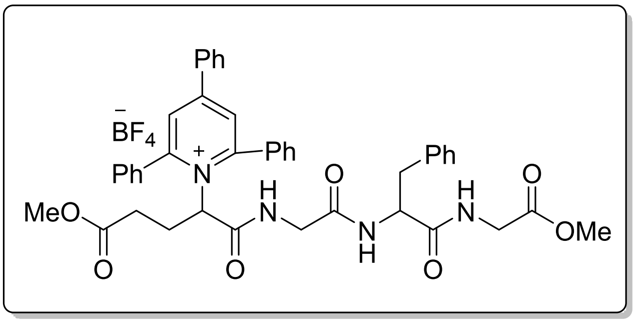

1-(7-benzyl-3,6,9,12,16-pentaoxo-2,17-dioxa-5,8,11-triazaoctadecan-13-yl)-2,4,6-triphenylpyridin-1-ium tetrafluoroborate (1ac). Prepared according to **GP4.** 1.0 mmol of amino acid was used for the limiting reagent. Purified by flash chromatography on silica gel (CH_2Cl_2/MeOH = 30:1). Ivory solid (352.7 mg, 43%). ^{1}H NMR (400 MHz, Chloroform-*d*) δ 7.98 – 7.88 (m, 2H), 7.82 – 7.72 (m, 2H), 7.64 – 7.37 (m, 14H), 7.23 – 7.01 (m, 6H), 6.87 – 6.74 (m, 1H), 5.57 – 5.35 (m, 1H), 4.65 (td, *J* = 8.3, 5.4 Hz, 1H), 3.99 – 3.89 (m, 1H), 3.87 – 3.76 (m, 2H), 3.74 – 3.66 (m, 1H), 3.65 – 3.58 (m, 3H), 3.53 – 3.45 (m, 3H), 3.24 – 3.09 (m, 1H), 3.04 – 2.92 (m, 1H), 2.34 – 2.16 (m, 2H), 2.15 – 2.03 (m, 2H). ^{13}C NMR (100 MHz, Chloroform-*d*) δ 172.5, 171.4, 170.1, 168.2, 166.7, 157.6, 156.6, 137.1, 133.4, 132.7, 132.2, 131.7, 129.9, 129.4, 129.3, 129.3, 128.5, 128.3, 127.8, 126.7, 69.5, 54.7, 52.1, 52.1, 43.6, 41.1, 37.3, 31.2, 26.7. ^{19}F NMR (375MHz, Chloroform-*d*) δ -151.41, -151.46. HRMS (ESI) m/z calcd. for $C_{43}H_{43}N_4O_7^+$ $[M-BF_4^-]^+$: 727.3126, found : 727.3128

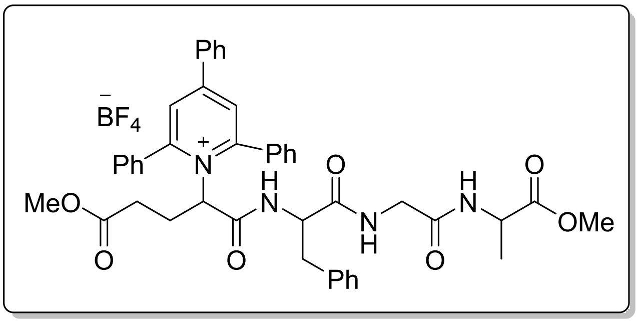

1-(10-benzyl-4-methyl-3,6,9,12,16-pentaoxo-2,17-dioxa-5,8,11-triazaoctadecan-13-yl)-2,4,6-triphenylpyridin-1-ium tetrafluoroborate (1ad). Prepared according to **GP4.** 1.0 mmol of amino acid was used for the limiting reagent. Purified by flash chromatography on silica gel (EtOAc/hexanes/MeOH = 49:49:2). Pale yellow solid (470.6 mg, 57%). ^{1}H NMR (600 MHz, Chloroform-*d*) δ 7.88 (s, 2H), 7.81 – 7.74 (m, 2H), 7.69 – 7.35 (m, 13H), 7.22 – 7.13 (m, 5H), 6.96 (d, J = 7.3 Hz, 1H), 6.70 (t, J = 6.0 Hz, 1H), 5.28 (t, J = 5.5 Hz, 1H), 4.55 – 4.39 (m, 2H), 4.06 (dd, J = 16.8, 6.4 Hz, 1H), 3.70 (dd, J = 16.8, 5.1 Hz, 1H), 3.66 (s, 3H), 3.49 (s, 3H), 3.18 (dd, J = 14.0, 6.3 Hz, 1H), 2.99 (dd, J = 13.8, 8.7 Hz, 1H), 2.25 – 2.11 (m, 2H), 2.10 – 1.96 (m, 0H), 1.29 (d, J = 7.2 Hz, 3H). ^{13}C NMR (100 MHz, Chloroform-*d*) δ 173.0, 170.6, 168.6, 167.0, 157.7, 156.5, 136.8, 133.6, 132.8,

132.6, 131.6, 130.0, 129.5, 129.4, 129.3, 128.6, 128.4, 128.3, 127.0, 70.2, 56.9, 52.4, 52.2, 48.3, 43.3, 37.5, 31.6, 26.7, 17.6. ^{19}F NMR (375MHz, Chloroform-*d*) δ -150.67, -150.72. HRMS (ESI) m/z calcd. for $C_{44}H_{45}N_4O_7^+$ [M-BF_4^-]$^+$: 741.3283, found : 741.3277

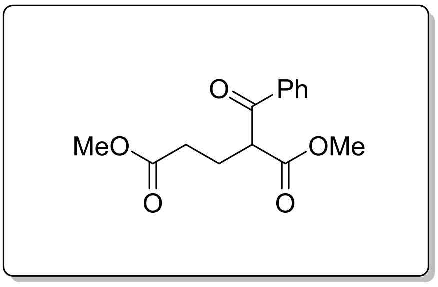

dimethyl 2-benzoylpentanedioate (3a). Prepared according to **GP1.** Purified by flash chromatography on silica gel (EtOAc/hexanes = 1:8). From 1-(1,5-dimethoxy-1,5-dioxopentan-2-yl)-2,4,6-triphenylpyridin-1-ium tetrafluoroborate (82.7 mg, 0.15 mmol), compound **3a** (28.9 mg, 73%) was obtained. Colorless oil. ^{1}H NMR (600 MHz, Chloroform-*d*) δ 8.18 – 7.93 (m, 1H), 7.61 – 7.56 (m, 1H), 7.51 – 7.45 (m, 1H), 4.52 (t, J = 7.1 Hz, 1H), 3.67 (d, J = 9.5 Hz, 3H), 2.56 – 2.37 (m, 1H), 2.35 – 2.25 (m, 1H). ^{13}C NMR (150 MHz, Chloroform-*d*) δ 195.0, 173.3, 170.1, 136.0, 133.8, 128.9, 128.8, 52.7, 52.6, 51.8, 31.3, 24.1. HRMS (EI) m/z calcd. for $C_{14}H_{16}O_5$ [M]$^+$: 264.0998, found : 264.1001

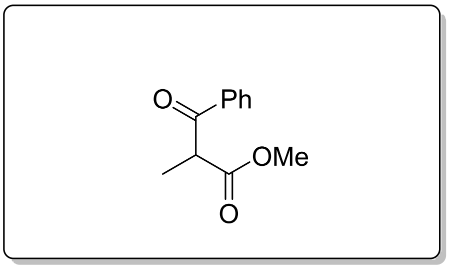

methyl 2-methyl-3-oxo-3-phenylpropanoate (3b). Prepared according to **GP1.** Purified by flash chromatography on silica gel (EtOAc/hexanes = 1:20). From 1-(1-methoxy-1-oxopropan-2-yl)-2,4,6-triphenylpyridin-1-ium tetrafluoroborate (72.2 mg, 0.15 mmol), compound **3b** (16.7 mg, 68%) was obtained. Yellow oil. ^{1}H NMR (400 MHz, Chloroform-*d*) δ 8.00 – 7.92 (m, 2H), 7.61 – 7.55 (m, 1H), 7.51 – 7.44 (m, 2H), 4.41 (q, *J* = 7.1 Hz, 1H), 3.69 (s, 3H), 1.50 (d, *J* = 7.1 Hz, 3H). ^{13}C NMR (100 MHz, Chloroform-*d*) δ 195.8, 171.3, 135.7, 133.5, 128.7, 128.6, 52.5, 48.0, 13.8. HRMS (EI) m/z calcd. for $C_{11}H_{12}O_3$ [M]$^+$: 192.0786, found : 192.0784

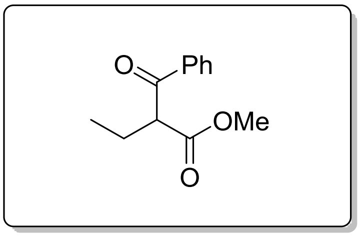

methyl 2-benzoylbutanoate (3c). Prepared according to **GP1.** Purified by flash chromatography on silica gel (EtOAc/hexanes = 1:20). From 1-(1-methoxy-1-oxobutan-2-yl)-2,4,6-triphenylpyridin-1-ium tetrafluoroborate (74.3 mg, 0.15 mmol), compound **3c** (20.3 mg, 66%) was obtained. Colorless oil. ^{1}H NMR (400 MHz, Chloroform-*d*) δ 8.02 – 7.96 (m, 2H), 7.58 (t, *J* = 7.4 Hz, 1H), 7.52 – 7.44 (m, 2H), 4.25 (t, *J* = 7.2 Hz, 1H), 3.68 (s, 3H), 2.14 – 1.89 (m, 2H), 0.99 (t, *J* = 7.4 Hz, 3H. ^{13}C NMR (100 MHz, Chloroform-*d*) δ 195.2, 170.4, 136.2, 133.5, 128.7, 128.5, 55.5, 52.4, 22.5, 12.1. HRMS (EI) m/z calcd. for $C_{12}H_{14}O_3$ $[M]^+$: 206.0943, found : 206.0942

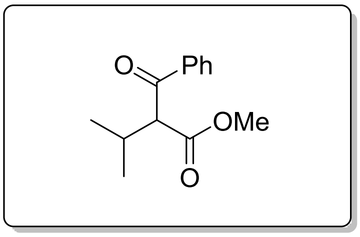

methyl 2-benzoyl-3-methylbutanoate (3d). Prepared according to **GP1.** Purified by flash chromatography on silica gel (EtOAc/hexanes = 1:20). From 1-(1-methoxy-3-methyl-1-oxobutan-2-yl)-2,4,6-triphenylpyridin-1-ium tetrafluoroborate (76.4 mg, 0.15 mmol), compound **3d** (16.7 mg, 51%) was obtained. Colorless oil. ^{1}H NMR (400 MHz, Chloroform-*d*) δ 8.05 – 7.93 (m, 2H), 7.58 (t, *J* = 7.4 Hz, 1H), 7.47 (t, *J* = 7.6 Hz, 2H), 4.12 (d, *J* = 9.4 Hz, 1H), 3.67 (s, 3H), 2.65 (dhept, *J* = 9.5, 6.7 Hz, 1H), 1.04 (d, *J* = 6.7 Hz, 3H), 0.93 (d, *J* = 6.7 Hz, 3H). ^{13}C NMR (100 MHz, Chloroform-*d*) δ 194.8, 169.8, 137.1, 133.7, 128.9, 128.7, 61.5, 52.5, 29.3, 21.1, 20.7. HRMS (EI) m/z calcd. for $C_{13}H_{16}O_3$ $[M]^+$: 220.1099, found : 220.1097

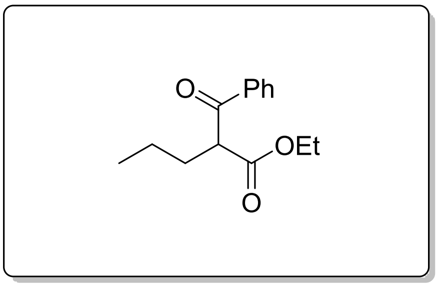

ethyl 2-benzoylpentanoate (3e). Prepared according to **GP1.** Purified by flash chromatography on silica gel (EtOAc/hexanes = 1:20). From 1-(1-ethoxy-1-oxopentan-2-yl)-2,4,6-triphenylpyridin-1-ium tetrafluoroborate (78.5 mg, 0.15 mmol), compound **3e** (25.9 mg, 73%) was obtained. Colorless oil. ^{1}H NMR (400 MHz, Chloroform-*d*) δ 8.04 – 7.93 (m, 2H), 7.63 – 7.54 (m, 1H), 7.51 – 7.42 (m, 2H), 4.30 (t, J = 7.2 Hz, 1H), 4.14 (q, J = 7.4 Hz, 2H), 2.10 – 1.90 (m, 2H), 1.44 – 1.32 (m, 2H), 1.17 (t, J = 7.1 Hz, 3H), 0.95 (t, J = 7.3 Hz, 3H). ^{13}C NMR (150 MHz, Chloroform-*d*) δ 195.4, 170.2, 136.5, 133.5, 128.8, 128.7, 61.4, 54.3, 31.2, 21.0, 14.1, 14.0. HRMS (EI) m/z calcd. for $C_{14}H_{18}O_3$ $[M]^+$: 234.1256, found : 234.1255

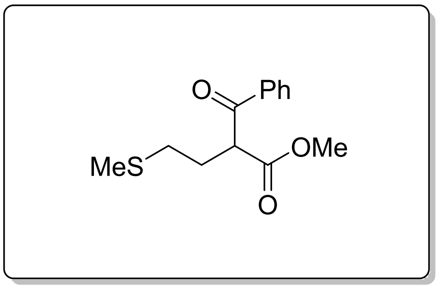

methyl 2-benzoyl-4-(methylthio)butanoate (3f). Prepared according to **GP1.** Purified by flash chromatography on silica gel (EtOAc/hexanes = 1:20). From (S)-1-(1-methoxy-4-(methylthio)-1-oxobutan-2-yl)-2,4,6-triphenylpyridin-1-ium tetrafluoroborate (81.6 mg, 0.15 mmol), compound **3f** (21.1 mg, 56%) was obtained. Colorless oil. ^{1}H NMR (400 MHz, Chloroform-*d*) δ 8.05 – 7.94 (m, 2H), 7.63 – 7.55 (m, 1H), 7.54 – 7.40 (m, 2H), 4.65 (t, J = 6.9 Hz, 1H), 3.68 (s, 3H), 2.64 – 2.47 (m, 2H), 2.37 – 2.22 (m, 2H), 2.07 (s, 3H). ^{13}C NMR (100 MHz, Chloroform-*d*) δ 195.0, 170.3, 136.1, 133.8, 128.9, 128.8, 52.7, 52.2, 32.2, 28.1, 15.3. HRMS (EI) m/z calcd. for $C_{13}H_{16}O_3S$ $[M]^+$: 252.0820, found : 252.0818

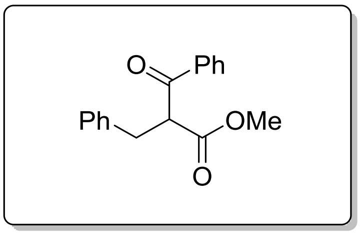

methyl 2-benzyl-3-oxo-3-phenylpropanoate (3g). Prepared according to **GP1.** Purified by flash chromatography on silica gel (EtOAc/hexanes = 1:15). From 1-(1-methoxy-1-oxo-3-phenylpropan-2-yl)-2,4,6-triphenylpyridin-1-ium tetrafluoroborate (83.6 mg, 0.15 mmol), compound **3g** (25.9 mg, 64%) was obtained. Colorless oil. ^{1}H NMR (600 MHz, Chloroform-*d*) δ 7.95 (d, *J* = 7.8 Hz, 2H), 7.56 (t, *J* = 7.2 Hz, 1H), 7.44 (t, *J* = 7.6 Hz, 2H), 7.27 – 7.21 (m, 4H), 7.18 (t, *J* = 7.2 Hz, 1H), 4.66 (t, *J* = 7.3 Hz, 1H), 3.64 (s, 3H), 3.43 – 3.19 (m, 2H). ^{13}C NMR (150 MHz, Chloroform-*d*) δ 194.4, 169.7, 138.3, 136.1, 133.5, 128.8, 128.7, 128.6, 128.5, 126.6, 55.9, 52.5, 34.8. HRMS (EI) m/z calcd. for $C_{17}H_{16}O_3$ $[M]^+$: 268.1099, found : 268.1097

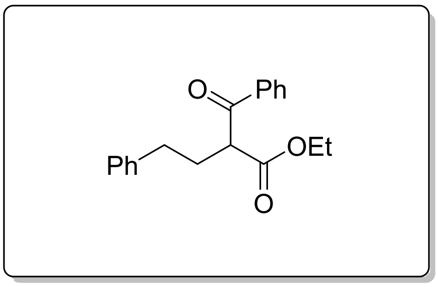

ethyl 2-benzoyl-4-phenylbutanoate (3h). Prepared according to **GP1.** Purified by flash chromatography on silica gel (EtOAc/hexanes = 1:20). From (S)-1-(1-methoxy-1-oxo-4-phenylbutan-2-yl)-2,4,6-triphenylpyridin-1-ium tetrafluoroborate (85.7 mg, 0.15 mmol), compound **3h** (30.9 mg, 70%) was obtained. Pale yellow oil. ^{1}H NMR (600 MHz, Chloroform-*d*) δ 7.93 – 7.87 (m, 2H), 7.60 – 7.53 (m, 1H), 7.49 – 7.42 (m, 2H), 7.35 – 7.27 (m, 2H), 7.24 – 7.12 (m, 3H), 4.30 (t, J = 7.1 Hz, 1H), 4.16 (q, J = 6.9 Hz, 2H), 2.70 (dh, J = 13.9, 7.4 Hz, 2H), 2.47 – 2.26 (m, 2H), 1.18 (t, J = 7.1 Hz, 3H). ^{13}C NMR (150 MHz, Chloroform-*d*) δ 195.2, 167.0, 140.9, 136.3, 128.8, 128.7, 128.7, 128.6, 126.4, 61.5, 53.4, 33.6, 30.6, 14.1. HRMS (EI) m/z calcd. for $C_{19}H_{20}O_3$ $[M]^+$: 296.1412, found : 296.1411

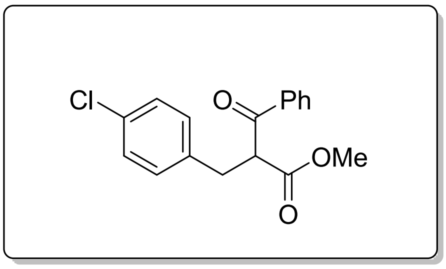

methyl 2-(4-chlorobenzyl)-3-oxo-3-phenylpropanoate (3i). Prepared according to **GP1.** Purified by flash chromatography on silica gel (EtOAc/hexanes = 1:20). From 1-(3-(4-chlorophenyl)-1-methoxy-1-oxopropan-2-yl)-2,4,6-triphenylpyridin-1-ium tetrafluoroborate (88.8 mg, 0.15 mmol), compound **3i** (27.5 mg, 61%) was obtained. Yellow oil. ^{1}H NMR (400 MHz, Chloroform-*d*) δ 7.98 – 7.90 (m, 2H), 7.57 (td, *J* = 7.3, 1.4 Hz, 1H), 7.45 (td, *J* = 7.7, 1.6 Hz, 2H), 7.25 – 7.19 (m, 2H), 7.15 (dd, *J* = 8.4, 1.7 Hz, 2H), 4.61 (td, *J* = 7.4, 1.5 Hz, 1H), 3.64 (d, *J* = 1.6 Hz, 3H), 3.30 (d, *J* = 7.2 Hz, 2H). ^{13}C NMR (100 MHz, Chloroform-*d*) δ 194.0, 169.5, 136.8, 135.9, 133.7, 132.5, 130.3, 128.8, 128.6, 128.6, 55.6, 52.6, 34.1. HRMS (EI) m/z calcd. for $C_{17}H_{15}ClO_3$ $[M]^+$: 302.0710, found : 302.0712

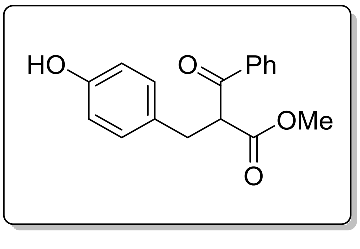

methyl 2-(4-hydroxybenzyl)-3-oxo-3-phenylpropanoate (3j). Prepared according to **GP1.** Purified by flash chromatography on silica gel (EtOAc/hexanes = 1:2). From 1-(3-(4-hydroxyphenyl)-1-methoxy-1-oxopropan-2-yl)-2,4,6-triphenylpyridin-1-ium tetrafluoroborate (86.0 mg, 0.15 mmol), compound **3j** (23.2 mg, 55%) was obtained. Colorless oil. ^{1}H NMR (400 MHz, Methylene Chloride-d_2) δ 7.94 (d, *J* = 7.2 Hz, 2H), 7.59 (t, *J* = 7.4 Hz, 1H), 7.47 (t, *J* = 7.7 Hz, 2H), 7.07 (d, *J* = 8.5 Hz, 2H), 6.72 (d, *J* = 8.5 Hz, 2H), 4.64 (t, *J* = 7.4 Hz, 1H), 3.62 (s, 3H), 3.30 – 3.16 (m, 2H). ^{13}C NMR (100 MHz, Methylene Chloride-d_2) δ 195.4, 170.5, 155.2, 136.7, 134.2, 130.7, 130.6, 129.3, 129.1, 115.8, 56.6, 53.0, 34.6. HRMS (EI) m/z calcd. for $C_{17}H_{16}O_4$ $[M]^+$: 284.1049, found : 284.1050

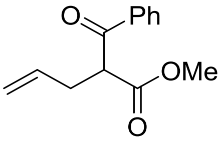

methyl 2-benzoylpent-4-enoate (3k). Prepared according to **GP1.** Purified by flash chromatography on silica gel (EtOAc/hexanes = 1:20). From 1-(1-methoxy-1-oxopent-4-en-2-yl)-2,4,6-triphenylpyridin-1-ium tetrafluoroborate (76.1 mg, 0.15 mmol), compound **3k** (20.2 mg, 62%) was obtained. Colorless oil. ^{1}H NMR (600 MHz, Chloroform-*d*) δ 8.02 – 7.96 (m, 2H), 7.61 – 7.56 (m, 1H), 7.52 – 7.46 (m, 2H), 5.81 (ddt, *J* = 17.1, 10.2, 6.9 Hz, 1H), 5.11 (dd, *J* = 17.0, 1.6 Hz, 1H), 5.04 (dq, *J* = 10.1, 1.3 Hz, 1H), 4.43 (t, *J* = 7.3 Hz, 1H), 3.68 (s, 3H), 2.87 – 2.64 (m, 2H). ^{13}C NMR (150 MHz, Chloroform-*d*) δ 194.4, 169.8, 136.1, 134.4, 133.6, 128.8, 128.6, 117.5, 53.6, 52.5, 33.1. HRMS (EI) m/z calcd. for $C_{13}H_{14}O_3$ $[M]^+$: 218.0943, found : 218.0942

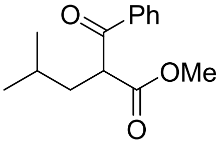

methyl 2-benzoyl-4-methylpentanoate (3l). Prepared according to **GP1.** Purified by flash chromatography on silica gel (EtOAc/hexanes = 1:20). From 1-(1-methoxy-4-methyl-1-oxopentan-2-yl)-2,4,6-triphenylpyridin-1-ium tetrafluoroborate (78.5 mg, 0.15 mmol), compound **3l** (30.0 mg, 64%) was obtained. Yellow oil. ^{1}H NMR (400 MHz, Chloroform-*d*) δ 8.01 – 7.95 (m, 2H), 7.62 – 7.54 (m, 1H), 7.51 – 7.43 (m, 2H), 4.42 (dd, *J* = 7.9, 6.5 Hz, 1H), 3.68 (s, 3H), 1.95 (ddd, *J* = 14.4, 7.9, 6.6 Hz, 1H), 1.85 (ddd, *J* = 13.9, 7.5, 6.5 Hz, 1H), 1.67 – 1.55 (m, 1H), 0.93 (dd, *J* = 12.9, 6.6 Hz, 6H). ^{13}C NMR (100 MHz, Chloroform-*d*) δ 195.2, 170.6, 136.1, 133.5, 128.7, 128.5, 52.4, 52.2, 37.8, 26.3, 22.5, 22.2. HRMS (EI) m/z calcd. for $C_{14}H_{18}O_3$ $[M]^+$: 234.1256, found : 234.1257

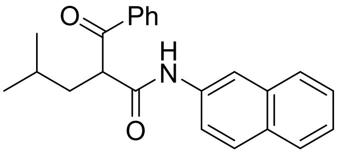

2-benzoyl-4-methyl-N-(naphthalen-2-yl)pentanamide (3m). Prepared according to **GP1.** Purified by

flash chromatography on silica gel (EtOAc/hexanes = 1:5). From 1-(4-methyl-1-(naphthalen-2-ylamino)-1-oxopentan-2-yl)-2,4,6-triphenylpyridin-1-ium tetrafluoroborate (95.2 mg, 0.15 mmol), compound **3m** (30.2 mg, 58%) was obtained. Off-white solid. ^{1}H NMR (400 MHz, Chloroform-*d*) δ 8.65 (s, 1H), 8.23 (d, *J* = 2.2 Hz, 1H), 8.16 – 8.05 (m, 2H), 7.77 (dd, *J* = 8.1, 6.3 Hz, 3H), 7.67 – 7.60 (m, 1H), 7.58 – 7.34 (m, 5H), 4.66 (t, *J* = 7.5 Hz, 1H), 2.01 (t, *J* = 7.3 Hz, 2H), 1.75 (dt, *J* = 13.4, 6.7 Hz, 1H), 1.02 (d, *J* = 6.6 Hz, 3H), 0.95 (d, *J* = 6.6 Hz, 3H). ^{13}C NMR (100 MHz, Chloroform-*d*) δ 200.5, 167.1, 136.3, 135.1, 134.2, 133.7, 130.7, 129.0, 128.7, 128.7, 127.6, 127.5, 126.5, 125.0, 119.8, 116.6, 54.9, 42.3, 26.6, 22.6, 22.3. HRMS (EI) m/z calcd. for $C_{23}H_{23}NO_2$ $[M]^+$: 345.1729, found : 345.1731

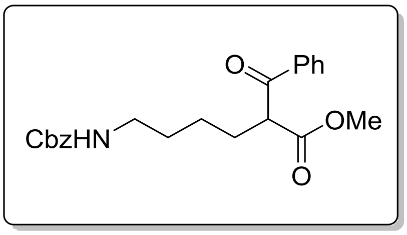

methyl 2-benzoyl-6-(((benzyloxy)carbonyl)amino)hexanoate (3n). Prepared according to **GP1.** Purified by flash chromatography on silica gel (EtOAc/hexanes = 1:2). From 21-(6-(((benzyloxy)carbonyl)amino)-1-methoxy-1-oxohexan-2-yl)-2,4,6-triphenylpyridin-1-ium tetrafluoroborate (100.9 mg, 0.15 mmol), compound **3n** (38.7 mg, 67%) was obtained. Colorless oil. ^{1}H NMR (600 MHz, Chloroform-*d*) δ 7.97 (d, *J* = 7.5 Hz, 2H), 7.58 (t, *J* = 7.4 Hz, 1H), 7.47 (t, *J* = 7.8 Hz, 2H), 7.35 – 7.32 (m, 4H), 7.32 – 7.27 (m, 1H), 5.07 (s, 2H), 4.88 (s, 1H), 4.31 (t, *J* = 7.1 Hz, 1H), 3.66 (s, 3H), 3.18 (q, *J* = 6.7 Hz, 2H), 2.12 – 1.88 (m, *J* = 6.7 Hz, 2H), 1.53 (q, *J* = 7.4 Hz, 2H), 1.37 (d, *J* = 10.2 Hz, 2H). ^{13}C NMR (100 MHz, Chloroform-*d*) δ 194.9, 170.3, 156.3, 136.6, 136.0, 133.5, 128.7, 128.5, 128.4, 128.0, 66.5, 53.8, 52.4, 40.6, 29.7, 28.5, 24.6. HRMS (EI) m/z calcd. for $C_{22}H_{25}NO_5$ $[M]^+$: 383.1733, found : 383.1731

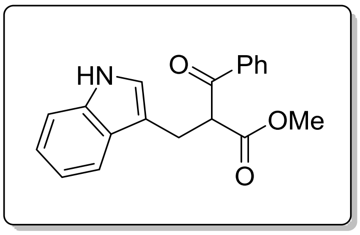

methyl 2-((1H-indol-3-yl)methyl)-3-oxo-3-phenylpropanoate (3o). Prepared according to **GP1.** Purified by flash chromatography on silica gel (EtOAc/hexanes = 1:1). From 1-(3-(1H-indol-3-yl)-1-methoxy-1-oxopropan-2-yl)-2,4,6-triphenylpyridin-1-ium tetrafluoroborate (89.5 mg, 0.15 mmol), compound **3o** (19.7 mg, 43%) was obtained. Brown gum. ^{1}H NMR (400 MHz, Methylene Chloride-d_2) δ 8.13 (s, 1H), 8.05 – 7.81 (m, 2H), 7.61 (dd, *J* = 7.8, 1.2 Hz, 1H), 7.57 (t, *J* = 7.4 Hz, 1H), 7.45 (t, *J* = 7.8 Hz, 2H), 7.35 (dt, *J* = 8.1, 1.0 Hz, 1H), 7.17 (ddd, *J* = 8.2, 7.0, 1.3 Hz, 1H), 7.11 (ddd, *J* = 8.1, 7.1, 1.2 Hz, 1H), 7.02 (d, *J* = 2.3 Hz, 1H), 4.79 (t, *J* = 7.3 Hz, 1H), 3.63 (s, 3H), 3.54 – 3.36 (m, 2H). ^{13}C

NMR (100 MHz, Methylene Chloride-d_2) δ 195.5, 170.6, 136.9, 136.7, 134.1, 129.3, 129.1, 127.7, 123.3, 122.6, 120.0, 119.0, 112.9, 111.7, 55.4, 52.9, 25.2. HRMS (EI) m/z calcd. for $C_{19}H_{17}NO_3$ $[M]^+$: 307.1208, found : 307.1209

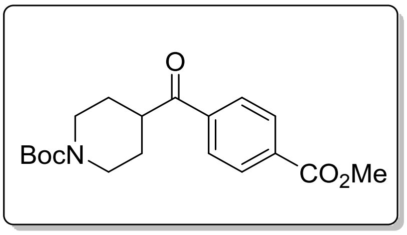

tert-butyl 4-(4-(methoxycarbonyl)benzoyl)piperidine-1-carboxylate (3p). Prepared according to **GP1.** Purified by flash chromatography on silica gel (EtOAc/hexanes = 1:4). From 1-(1-(tert-butoxycarbonyl)piperidin-4-yl)-2,4,6-triphenylpyridin-1-ium tetrafluoroborate (86.7 mg, 0.15 mmol), compound **3p** (29.4 mg, 56%) was obtained. White solid. ^{1}H NMR (600 MHz, Chloroform-*d*) δ 8.13 (d, *J* = 8.5 Hz, 2H), 7.97 (d, *J* = 8.5 Hz, 2H), 4.16 (s, 2H), 3.95 (s, 3H), 3.40 (tt, *J* = 11.1, 3.7 Hz, 1H), 2.91 (s, 2H), 1.94 – 1.79 (m, 2H), 1.78 – 1.63 (m, 2H), 1.46 (s, 9H). ^{13}C NMR (150 MHz, Chloroform-*d*) δ 201.6, 166.1, 154.7, 139.2, 133.9, 130.0, 128.1, 79.7, 52.5, 43.9, 28.4, 28.2. HRMS (EI) m/z calcd. for $C_{19}H_{25}NO_5$ $[M]^+$: 347.1733, found : 347.1735

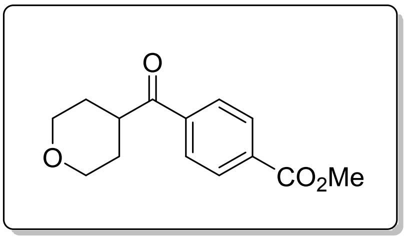

methyl 4-(tetrahydro-2H-pyran-4-carbonyl)benzoate (3q). Prepared according to **GP1.** Purified by flash chromatography on silica gel (EtOAc/hexanes = 1:3). From 2,4,6-triphenyl-1-(tetrahydro-2H-pyran-4-yl)pyridin-1-ium tetrafluoroborate (71.9 mg, 0.15 mmol), compound **3q** (13.6 mg, 36%) was obtained. Ivory solid. ^{1}H NMR (600 MHz, Chloroform-*d*) δ 8.13 (d, *J* = 8.3 Hz, 2H), 7.98 (d, *J* = 8.4 Hz, 2H), 4.11 – 4.02 (m, 2H), 3.95 (s, 3H), 3.56 (td, *J* = 11.6, 2.4 Hz, 2H), 3.49 (tt, *J* = 11.1, 3.9 Hz, 1H), 1.93 – 1.82 (m, 2H), 1.79 (ddt, *J* = 11.1, 4.3, 2.2 Hz, 2H). ^{13}C NMR (150 MHz, Chloroform-*d*) δ 201.3, 166.1, 139.1, 133.8, 129.9, 128.1, 67.2, 52.5, 42.9, 28.9. HRMS (ESI) m/z calcd. for $C_{14}H_{16}NaO_4$ $[M+Na]^+$: 271.0941, found : 271.0895

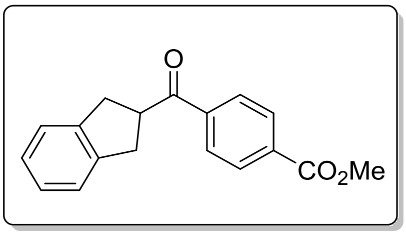

methyl 4-(2,3-dihydro-1H-indene-2-carbonyl)benzoate (3r). Prepared according to **GP1.** Purified by flash chromatography on silica gel (CH_2Cl_2/EtOAc/hexanes = 1:1:6). From 1-(2,3-dihydro-1H-inden-2-yl)-2,4,6-triphenylpyridin-1-ium tetrafluoroborate (76.7 mg, 0.15 mmol), compound **3r** (14.7 mg, 35%) was obtained. White solid. ^{1}H NMR (600 MHz, Chloroform-*d*) δ 8.16 (d, *J* = 8.4 Hz, 2H), 8.06 (d, *J* = 8.4 Hz, 2H), 7.24 – 7.19 (m, 2H), 7.20 – 7.13 (m, 2H), 4.30 (tt, *J* = 9.1, 7.6 Hz, 1H), 3.97 (s, 3H), 3.38 (dd, *J* = 15.9, 7.6 Hz, 2H), 3.29 (dd, *J* = 15.9, 9.0 Hz, 2H). ^{13}C NMR (150 MHz, Chloroform-*d*) δ 200.4, 166.2, 141.3, 139.7, 133.8, 129.9, 128.4, 126.7, 124.4, 52.5, 46.6, 36.1. HRMS (EI) m/z calcd. for $C_{18}H_{16}O_3$ $[M]^+$: 280.1099, found : 280.1101

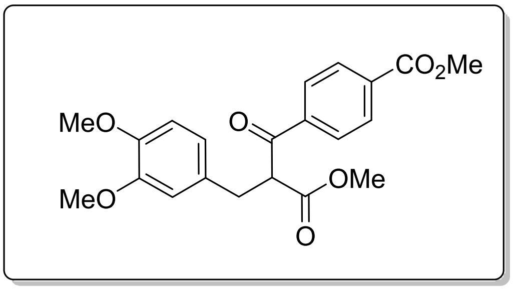

methyl 4-(2-(3,4-dimethoxybenzyl)-3-methoxy-3-oxopropanoyl)benzoate (3s). Prepared according to **GP1.** Purified by flash chromatography on silica gel (EtOAc/hexanes = 1:2). From 1-(3-(3,4-dimethoxyphenyl)-1-methoxy-1-oxopropan-2-yl)-2,4,6-triphenylpyridin-1-ium tetrafluoroborate (92.6 mg, 0.15 mmol), compound **3s** (38.7 mg, 67%) was obtained. Yellow oil. ^{1}H NMR (600 MHz, Chloroform-*d*) δ 8.3 – 8.0 (m, 2H), 8.0 (d, *J* = 8.5 Hz, 2H), 6.7 (d, *J* = 15.6 Hz, 3H), 4.6 (t, *J* = 7.3 Hz, 1H), 3.9 (s, 3H), 3.8 (d, *J* = 2.0 Hz, 6H), 3.6 (s, 3H), 3.3 (d, *J* = 7.3 Hz, 2H). ^{13}C NMR (150 MHz, Chloroform-*d*) δ 194.3, 169.4, 165.9, 148.8, 147.8, 139.4, 134.2, 130.5, 129.8, 128.4, 120.8, 112.2, 111.3, 56.3, 55.8, 55.8, 52.6, 52.4, 34.4. HRMS (ESI) m/z calcd. for $C_{21}H_{22}NaO_7$ $[M+Na]^+$: 409.1258, found : 409.1262

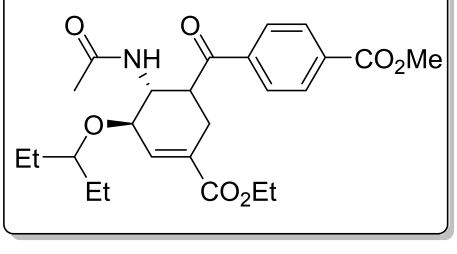

methyl 4-((5R,6R)-6-acetamido-3-(ethoxycarbonyl)-5-(pentan-3-yloxy)cyclohex-3-ene-1-carbonyl)benzoate (3t). Prepared according to **GP1.** Purified by flash chromatography on silica gel (EtOAc/hexanes = 1:1). From 1-((5R,6R)-6-acetamido-3-(ethoxycarbonyl)-5-(pentan-3-yloxy)cyclohex-3-en-1-yl)-2,4,6-triphenylpyridin-1-ium tetrafluoroborate (103.5 mg, 0.15 mmol), compound **3t** (31.5 mg, 46%) was obtained. Light yellow soild. ^{1}H NMR (600 MHz, Chloroform-*d*) δ 8.12 (d, *J* = 8.4 Hz, 2H), 8.06 (d, *J* = 8.5 Hz, 2H), 6.86 (s, 1H), 5.86 (d, *J* = 6.7 Hz, 1H), 4.81 – 4.65 (m,

2H), 4.19 (q, J = 7.0 Hz, 2H), 3.94 (s, 3H), 3.72 (ddd, J = 10.2, 8.0, 6.7 Hz, 1H), 3.31 (p, J = 5.7 Hz, 1H), 2.62 (dd, J = 18.0, 5.5 Hz, 1H), 2.43 (ddt, J = 18.0, 9.7, 2.8 Hz, 1H), 1.80 (s, 3H), 1.57 – 1.46 (m, 2H), 1.46 – 1.38 (m, 2H), 1.27 (t, J = 7.1 Hz, 3H), 0.90 (t, J = 7.4 Hz, 3H), 0.81 (t, J = 7.4 Hz, 3H). ^{13}C NMR (100 MHz, Chloroform-*d*) δ 200.4, 170.7, 166.2, 166.1, 139.6, 137.5, 134.0, 129.9, 129.7, 128.6, 81.8, 72.0, 60.9, 55.0, 52.5, 43.1, 27.9, 26.2, 25.5, 23.6, 14.2, 9.6, 9.2. HRMS (EI) m/z calcd. for $C_{25}H_{33}NO_7$ $[M]^+$: 459.2257, found : 459.2259

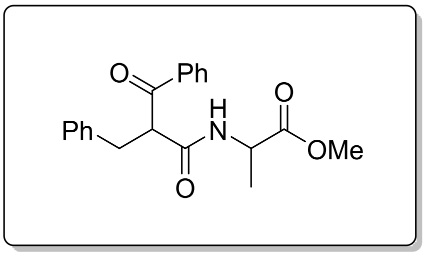

methyl (2-benzyl-3-oxo-3-phenylpropanoyl)alaninate (3u). Prepared according to **GP1.** Purified by flash chromatography on silica gel (EtOAc/hexanes = 1:2). From 1-(1-((1-methoxy-1-oxopropan-2-yl)amino)-1-oxo-3-phenylpropan-2-yl)-2,4,6-triphenylpyridin-1-ium tetrafluoroborate (94.3 mg, 0.15 mmol), compound **3u** (33.2 mg, 65%) was obtained. Ivory solid. ^{1}H NMR (600 MHz, Chloroform-*d*) δ 7.97 – 7.73 (m, 2H), 7.61 – 7.50 (m, 1H), 7.46 – 7.36 (m, 2H), 7.24 – 7.11 (m, 5H), [6.94 (d, J = 7.3 Hz, 0.4H), 6.72 (d, J = 7.6 Hz, 0.54H)], 4.67 – 4.56 (m, 1H), 4.56 – 4.46 (m, 1H), [3.73 (s, 1.7H), 3.65 (s, 1.4H)], 3.43 – 3.34 (m, 1H), 3.33 – 3.21 (m, 1H), [1.36 (d, J = 7.1 Hz, 1.2H), 1.28 (d, J = 7.2 Hz, 1.8H)]. ^{13}C NMR (150 MHz, Chloroform-*d*) δ [198.2, 197.9], [172.9, 172.8], [167.81, 167.79], [137.9, 137.7], 136.4, [133.73, 133.71], 128.9, 128.7, [128.61, 128.56], [128.50, 128.48], [126.8, 126.7], [57.6, 57.2], [52.4, 52.3], [48.3, 48.1], [37.6, 37.4], [18.1, 18.0]. HRMS (EI) m/z calcd. for $C_{20}H_{21}NO_4$ $[M]^+$: 339.1471, found : 339.1473

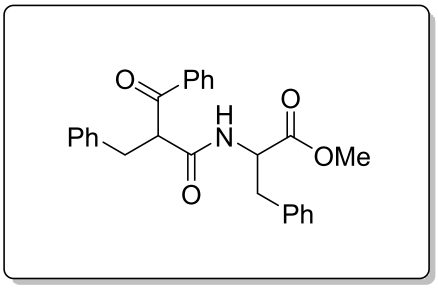

methyl (2-benzyl-3-oxo-3-phenylpropanoyl)phenylalaninate (3v). Prepared according to **GP1.** Purified by flash chromatography on silica gel (EtOAc/hexanes = 1:8). From 1-(1-((1-methoxy-1-oxo-3-phenylpropan-2-yl)amino)-1-oxo-3-phenylpropan-2-yl)-2,4,6-triphenylpyridin-1-ium tetrafluoroborate (105.8 mg, 0.15 mmol), compound **3v** (24.9 mg, 61%) was obtained. Pale yellow oil. ^{1}H NMR (600 MHz, Chloroform-*d*) δ 7.91 – 7.81 (m, 2H), 7.60 – 7.51 (m, 1H), 7.46 – 7.37 (m, 2H), 7.31 – 7.15 (m, 5H), 7.15 – 7.08 (m, 2H), 7.10 – 7.02 (m, 2H), 6.88 – 6.80 (m, 1H), 6.74 (d, J = 7.7 Hz,

0.51H), 6.59 (d, J = 8.2 Hz, 0.5H), 4.86 – 4.76 (m, 1H), 4.59 – 4.52 (m, 1H), 3.71 (s, 1.6H), 3.62 (s, 1.4H), 3.35 (dd, J = 13.8, 8.3 Hz, 0.5H), 3.29 – 3.21 (m, 1H), 3.18 – 3.11 (m, 1H), 3.02 (dd, J = 13.9, 6.9 Hz, 0.5H), 2.99 (dd, J = 5.9, 2.8 Hz, 1H). ^{13}C NMR (150 MHz, Chloroform-*d*) δ [198.1, 197.6], [171.6, 171.5], [168.0, 167.7], [138.2, 137.9], [136.6, 136.6], [135.9, 135.5], [133.9, 133.9], [129.4, 129.3], [129.1, 129.0], 128.9, 128.8, 128.8, 128.7, 128.7, 128.7, 128.6, [127.3, 127.2], 126.9, [58.1,57.4], [53.6, 53.3], [52.5, 52.4], 38.0, [37.7, 37.3]. HRMS (EI) m/z calcd. for $C_{26}H_{25}NO_4$ $[M]^+$: 415.1784, found : 415.1784

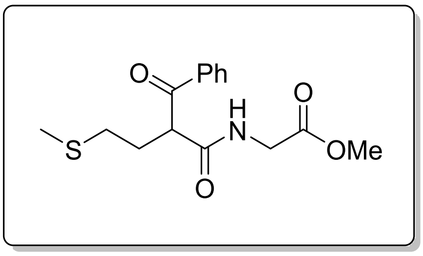

methyl (2-benzoyl-4-(methylthio)butanoyl)glycinate (3w). Prepared according to **GP1.** Purified by flash chromatography on silica gel (EtOAc/hexanes = 1:2). From 1-(1-((2-methoxy-2-oxoethyl)amino)-4-(methylthio)-1-oxobutan-2-yl)-2,4,6-triphenylpyridin-1-ium tetrafluoroborate (89.8 mg, 0.15 mmol), compound **3w** (24.2 mg, 52%) was obtained. Ivory solid. ^{1}H NMR (600 MHz, Chloroform-*d*) δ 8.10 – 7.99 (m, 2H), 7.60 (t, *J* = 7.4 Hz, 1H), 7.48 (t, *J* = 7.8 Hz, 2H), 6.92 (t, *J* = 5.6 Hz, 1H), 4.59 (t, *J* = 7.1 Hz, 1H), 4.07 (dd, *J* = 18.2, 5.7 Hz, 1H), 3.94 (dd, *J* = 18.2, 5.3 Hz, 1H), 3.71 (s, 3H), 2.57 (td, *J* = 7.1, 2.7 Hz, 2H), 2.34 (dq, *J* = 14.5, 7.3 Hz, 1H), 2.27 (dq, *J* = 13.9, 7.0 Hz, 1H), 2.06 (s, 3H). ^{13}C NMR (150 MHz, Chloroform-*d*) δ 198.0, 169.8, 168.8, 136.2, 134.0, 128.9, 128.7, 53.8, 52.3, 41.3, 31.8, 30.7, 15.2. HRMS (EI) m/z calcd. for $C_{15}H_{19}NO_4S$ $[M]^+$: 309.1035, found : 309.1031

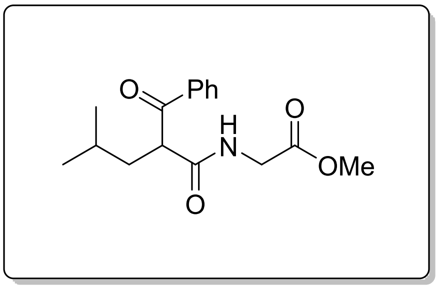

methyl (2-benzoyl-4-methylpentanoyl)glycinate (3x). Prepared according to **GP1.** Purified by flash chromatography on silica gel (EtOAc/hexanes = 1:8). From 1-(1-((2-methoxy-2-oxoethyl)amino)-4-methyl-1-oxopentan-2-yl)-2,4,6-triphenylpyridin-1-ium tetrafluoroborate (84.9 mg, 0.15 mmol), compound **3x** (27.2 mg, 56%) was obtained. Colorless oil. ^{1}H NMR (600 MHz, Chloroform-*d*) δ 8.08 – 7.95 (m, 2H), 7.65 – 7.57 (m, 1H), 7.55 – 7.35 (m, 2H), 6.87 (t, J = 5.6 Hz, 1H), 4.50 (t, J = 7.4 Hz, 1H), 4.08 (dd, J = 18.2, 5.8 Hz, 1H), 3.92 (dd, J = 18.2, 5.1 Hz, 1H), 3.71 (s, 3H), 1.98 – 1.90 (m, 1H), 1.91 – 1.84 (m, 1H), 1.68 – 1.59 (m, 1H), 0.94 (d, J = 6.5 Hz, 3H), 0.91 (d, J = 6.6 Hz, 3H). ^{13}C NMR

(150 MHz, Chloroform-*d*) δ 199.4, 170.0, 169.5, 136.6, 134.1, 129.0, 128.8, 54.1, 52.4, 41.4 (d, J = 2.6 Hz), 26.6, 22.7, 22.5. HRMS (EI) m/z calcd. for $C_{16}H_{21}NO_4$ $[M]^+$: 291.1471, found : 291.1467

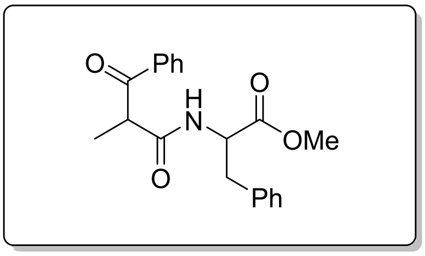

methyl (2-methyl-3-oxo-3-phenylpropanoyl)phenylalaninate (3y). Prepared according to **GP1.** Purified by flash chromatography on silica gel (EtOAc/hexanes = 1:2). From 1-(1-((1-methoxy-1-oxo-3-phenylpropan-2-yl)amino)-1-oxopropan-2-yl)-2,4,6-triphenylpyridin-1-ium tetrafluoroborate (94.3 mg, 0.15 mmol), compound **3y** (25.5 mg, 50%) was obtained. Ivory solid. ^{1}H NMR (600 MHz, Chloroform-*d*) δ 8.09 – 7.84 (m, 2H), 7.66 – 7.56 (m, 1H), 7.51 – 7.43 (m, 2H), 7.30 – 7.19 (m, 2H), 7.16 – 7.05 (m, 2H), 6.99 – 6.92 (m, 1H), [6.88 (d, *J* = 7.8 Hz, 0.5H), 6.70 (d, *J* = 8.1 Hz, 0.5H)], 4.83 (ddt, *J* = 8.1, 6.6, 5.4 Hz, 1H), 4.34 (dq, *J* = 12.1, 7.2 Hz, 1H), [3.71 (s, 1.5H), 3.65 (s, 1.5H)], 3.27 – 2.80 (m, 2H), [1.49 (d, *J* = 7.2 Hz, 1.5H), 1.43 (d, *J* = 7.2 Hz, 1.5H)].^{13}C NMR (150 MHz, Chloroform-*d*) δ [198.8, 198.4], 171.5, [169.6, 169.5], [135.83, 135.77], [135.7, 135.5], [133.8, 133.7], [129.2, 129.1], [128.78, 128.77], 128.6, [128.5, 128.4], [127.1, 127.0], 53.2, [52.30, 52.26], [49.6, 49.3], [37.8, 37.7], [16.5, 16.1]. HRMS (EI) m/z calcd. for $C_{20}H_{21}NO_4$ $[M]^+$: 339.1471, found : 339.1470

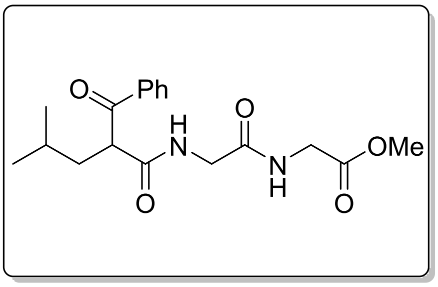

methyl (2-benzoyl-4-methylpentanoyl)glycylglycinate (3z). Prepared according to **GP1.** Purified by flash chromatography on silica gel (EtOAc/hexanes = 1:1). From 1-(1-((2-((2-methoxy-2-oxoethyl)amino)-2-oxoethyl)amino)-4-methyl-1-oxopentan-2-yl)-2,4,6-triphenylpyridin-1-ium tetrafluoroborate (95.7 mg, 0.15 mmol), compound **3z** (22.3 mg, 43%) was obtained. Colorless oil. ^{1}H NMR (600 MHz, Chloroform-*d*) δ 8.04 – 7.97 (m, 2H), 7.64 – 7.56 (m, 1H), 7.51 – 7.43 (m, 2H), 7.15 (t, J = 5.7 Hz, 1H), 6.83 (t, J = 5.4 Hz, 1H), 4.50 (t, J = 7.4 Hz, 1H), 4.05 – 3.88 (m, 4H), 3.71 (s, 3H), 1.97 – 1.82 (m, 2H), 1.69 – 1.57 (m, 2H), 0.95 (d, J = 6.6 Hz, 3H), 0.90 (d, J = 6.6 Hz, 3H). ^{13}C NMR (150 MHz, Chloroform-*d*) δ 199.1, 170.1, 170.1, 169.2, 136.3, 134.0, 129.0, 128.8, 53.9, 52.5, 43.4, 41.3, 40.9, 26.7, 22.7, 22.4. HRMS (EI) m/z calcd. for $C_{18}H_{24}N_2O_5$ $[M]^+$: 348.1685, found : 348.1688

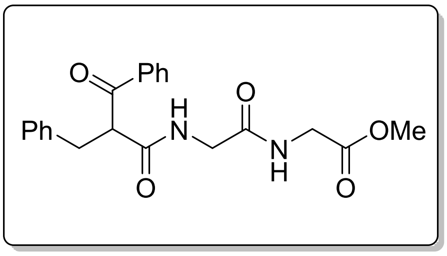

methyl (2-benzyl-3-oxo-3-phenylpropanoyl)glycylglycinate (3aa). Prepared according to **GP1.** Purified by flash chromatography on silica gel (EtOAc/hexanes = 2:1). From 1-(1-((2-((2-methoxy-2-oxoethyl)amino)-2-oxoethyl)amino)-1-oxo-3-phenylpropan-2-yl)-2,4,6-triphenylpyridin-1-ium tetrafluoroborate (100.7 mg, 0.15 mmol), compound **3aa** (37.7 mg, 65%) was obtained. Pale yellow solid. ^{1}H NMR (600 MHz, Chloroform-*d*) δ 7.99 – 7.79 (m, 2H), 7.56 – 7.48 (m, 1H), 7.46 – 7.34 (m, 3H), 7.22 – 7.15 (m, 4H), 7.15 – 7.11 (m, 1H), 6.88 (t, *J* = 5.6 Hz, 1H), 4.70 (t, *J* = 7.4 Hz, 1H), 3.96 – 3.86 (m, 3H), 3.81 (dd, *J* = 16.7, 5.4 Hz, 1H), 3.68 (s, 3H), 3.30 (dd, *J* = 7.4, 2.0 Hz, 2H). ^{13}C NMR (150 MHz, Chloroform-*d*) δ 197.3, 170.0, 169.3, 169.1, 137.9, 136.1, 133.7, 128.8, 128.7, 128.6, 128.5, 126.7, 56.8, 52.2, 43.2, 41.0, 36.7. HRMS (EI) m/z calcd. for $C_{21}H_{22}N_2O_5$ $[M]^+$: 382.4160, found : 382.1528

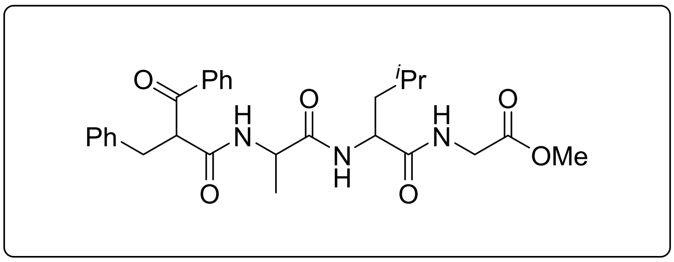

methyl (2-benzyl-3-oxo-3-phenylpropanoyl)alanylleucylglycinate (3ab). Prepared according to **GP1.** Purified by flash chromatography on silica gel (CH_2Cl_2/MeOH = 30:1). From 1-(7-isobutyl-10-methyl-3,6,9,12-tetraoxo-14-phenyl-2-oxa-5,8,11-triazatetradecan-13-yl)-2,4,6-triphenylpyridin-1-ium tetrafluoroborate (119.8 mg, 0.15 mmol), compound **3ab** (43.5 mg, 57%) was obtained. Pale yellow solid. ^{1}H NMR (600 MHz, DMSO-d_6) δ 8.05 – 7.93 (m, 2H), 7.67 – 7.57 (m, 1H), 7.53 – 7.41 (m, 2H), 7.28 – 7.06 (m, 5H), 4.95 – 4.74 (m, 1H), 4.36 – 4.17 (m, 2H), 3.93 – 3.72 (m, 2H), 3.61 (s, 1.5H), 3.61 (s, 1.5H), 3.25 – 3.10 (m, 1H), 3.08 – 2.93 (m, 1H), 1.68 – 1.54 (m, 0.5H), 1.50 – 1.29 (m, 2.5H), 1.07 (d, J = 7.0 Hz, 1.5H), 0.97 (d, J = 6.9 Hz, 1.5H), 0.88 (d, J = 6.6 Hz, 1.5H), 0.85 (d, J = 6.5 Hz, 1.5H), 0.78 (d, J = 6.4 Hz, 1.5H), 0.73 (d, J = 6.4 Hz, 1.5H). ^{13}C NMR (150 MHz, DMSO-d_6 δ 194.8, 194.8, 172.4 – 172.0 (m), 171.5 – 171.2 (m), 170.2 – 169.7 (m), 168.0 – 167.3 (m), [139.3, 138.9], [136.1, 136.0], [133.3, 133.2], [129.0, 128.8], [128.6, 128.6], [128.2, 128.2], [128.1, 128.0], [126.1, 126.0], 55.9 – 55.4 (m), 51.1 – 50.1 (m), 48.3 – 47.2 (m), [41.0, 40.3], [34.6, 34.5], [34.1, 34.1], [24.0, 23.9], [22.9, 22.8], [21.7, 21.6], 18.4 – 17.7 (m). HRMS (ESI) m/z calcd. for $C_{28}H_{35}N_3NaO_6^+$ $[M+Na]^+$: 532.2418, found : 532.2418

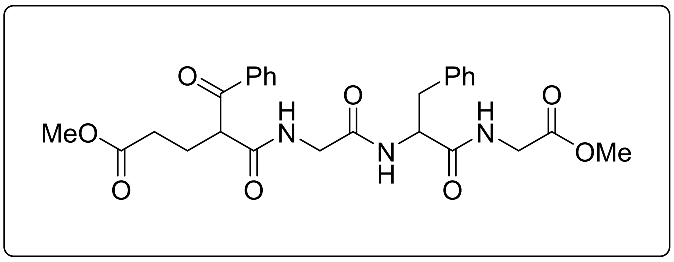

methyl 4-benzoyl-5-((2-((1-((2-methoxy-2-oxoethyl)amino)-1-oxo-3-phenylpropan-2-yl)amino)-2-oxoethyl)amino)-5-oxopentanoate (3ac). Prepared according to **GP1.** Purified by flash chromatography on silica gel (CH_2Cl_2/MeOH = 30:1). From 1-(7-benzyl-3,6,9,12,16-pentaoxo-2,17-dioxa-5,8,11-triazaoctadecan-13-yl)-2,4,6-triphenylpyridin-1-ium tetrafluoroborate (122.2 mg, 0.15 mmol), compound **3ac** (53.5 mg, 68%) was obtained. Pale yellow solid. ^{1}H NMR (600 MHz, Chloroform-*d*) δ 8.12 – 7.89 (m, 2H), 7.64 – 7.52 (m, 2H), 7.49 – 7.42 (m, 2H), 7.36 – 7.28 (m, 1H), 7.24 – 7.09 (m, 6H), 4.81 (q, *J* = 7.5 Hz, 1H), 4.52 (dt, *J* = 8.5, 6.0 Hz, 1H), 4.00 – 3.74 (m, 4H), 3.64 – 3.61 (m, 6H), 3.14 – 3.06 (m, 1H), 3.03 – 2.89 (m, 1H), 2.48 – 2.33 (m, 2H), 2.32 – 2.23 (m, 1H), 2.23 – 2.13 (m, 1H). ^{13}C NMR (150 MHz, Chloroform-*d*) δ [197.6, 197.5], [173.3, 173.2], [171.33, 171.30], [169.9, 169.86], [169.73, 169.69], [169.0, 168.8], [136.6, 136.5], [135.74, 135.69], 133.9, [129.23, 129.20], [128.9, 128.8], [128.74, 128.71], [128.43, 128.41], [126.79, 126.77], [54.39, 54.36], [53.55, 53.52], 52.1, [51.69, 51.65], [43.30, 43.27], [41.10, 41.07], [38.13, 38.07], [31.3, 31.2], 25.6. HRMS (ESI) m/z calcd. for $C_{27}H_{31}N_3NaO_8^+$ $[M+Na]^+$: 548.2003, found : 548.2003

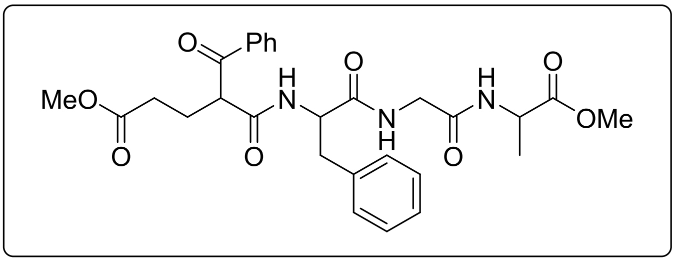

methyl 4-benzoyl-5-((1-((2-((1-methoxy-1-oxopropan-2-yl)amino)-2-oxoethyl)amino)-1-oxo-3-phenylpropan-2-yl)amino)-5-oxopentanoate (3ad). Prepared according to **GP1.** Purified by flash chromatography on silica gel (CH_2Cl_2/MeOH = 30:1). From 1-(10-benzyl-4-methyl-3,6,9,12,16-pentaoxo-2,17-dioxa-5,8,11-triazaoctadecan-13-yl)-2,4,6-triphenylpyridin-1-ium tetrafluoroborate (124.3 mg, 0.15 mmol), compound **3ad** (40.5 mg, 50%) was obtained. Pale yellow solid. ^{1}H NMR (600 MHz, Chloroform-*d)* δ 8.01 – 7.74 (m, 2H), 7.61 – 7.49 (m, 1H), 7.46 – 7.39 (m, 2H), 7.36 (d, *J* = 7.2 Hz, 0.5H), 7.26 – 7.20 (m, 1H), 7.20 – 7.15 (m, 3H), 7.15 – 7.06 (m, 2.5H), 7.02 (dd, *J* = 17.8, 7.3 Hz, 1H), 4.69 – 4.61 (m, 1H), 4.49 (h, *J* = 7.3 Hz, 1H), 4.42 – 4.33 (m, 1H), 3.99 (d, J = 6.0 Hz, 0.5H), 3.96 (d, *J* = 5.9 Hz, 0.5H), 3.82 (dd, *J* = 16.8, 5.4 Hz, 0.5H), 3.76 (dd, *J* = 16.8, 5.2 Hz, 0.5H), 3.67 (s, 1.5H), 3.64 (s, 1.5H), 3.63 (s, 3H), 3.24 – 3.10 (m, 1H), 3.04 – 2.90 (m, 1H), 2.40 – 2.28 (m, 1H), 2.26 – 2.09 (m, 2.5H), 2.01 – 1.92 (m, 0.5H), 1.34 (dd, *J* = 7.3, 2.8 Hz, 3H).^{13}C NMR (150 MHz, Chloroform-*d*) δ 197.7, 173.5, [173.3, 173.3], [171.5, 171.4], [169.5, 169.4], [168.5, 168.4], [136.6, 136.3], [135.9,

135.8], 134.1, [129.2, 129.2], 129.0, 129.0, 128.8, 128.7, [127.1, 127.1], [55.3, 55.3], [53.9, 53.5], [52.5, 52.5], 51.8, 48.2, [43.2, 43.1], [37.7, 37.6], [31.1, 31.0], [26.2, 25.9], [17.9, 17.9]. HRMS (ESI) m/z calcd. for $C_{28}H_{33}N_3NaO_8^+$ [M+Na]$^+$: 562.2160, found : 562.2168

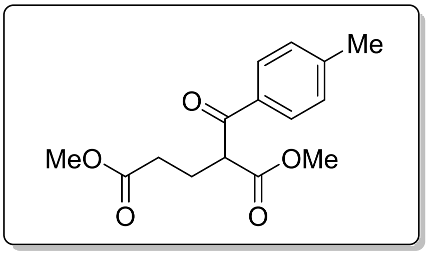

dimethyl 2-(4-methylbenzoyl)pentanedioate (3ae). Prepared according to **GP1.** Purified by flash chromatography on silica gel (EtOAc/hexanes = 1:4). From 1-(1,5-dimethoxy-1,5-dioxopentan-2-yl)-2,4,6-triphenylpyridin-1-ium tetrafluoroborate (83.0 mg, 0.15 mmol). compound **3ae** (23.9 mg, 61%) was obtained. Colorless oil. ^{1}H NMR (400 MHz, Chloroform-*d*) δ 7.94 (d, *J* = 8.0 Hz, 2H), 7.30 (d, *J* = 8.1 Hz, 2H), 4.52 (t, *J* = 7.1 Hz, 1H), 3.70 (s, 3H), 3.69 (s, 3H), 2.53 – 2.44 (m, 2H), 2.43 (s, 3H), 2.35 – 2.25 (m, 2H). ^{13}C NMR (100 MHz, Chloroform-*d*) δ 194.4, 173.2, 170.1, 144.7, 133.3, 129.5, 128.8, 52.5, 52.3, 51.6, 31.2, 24.0, 21.7. HRMS (EI) m/z calcd. for $C_{15}H_{18}O_5$ [M]$^+$: 278.1154, found : 278.1151

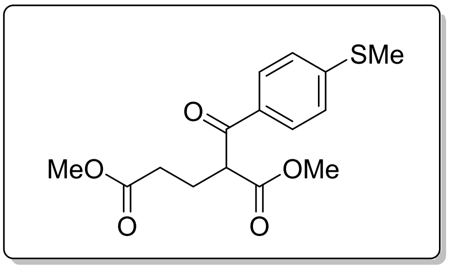

dimethyl 2-(4-(methylthio)benzoyl)pentanedioate (3af). Prepared according to **GP1.** Purified by flash chromatography on silica gel (EtOAc/hexanes = 1:5). From 1-(1,5-dimethoxy-1,5-dioxopentan-2-yl)-2,4,6-triphenylpyridin-1-ium tetrafluoroborate (83.0 mg, 0.15 mmol), compound **3af** (24.6 mg, 53%) was obtained. Yellow oil. ^{1}H NMR (400 MHz, Chloroform-*d*) δ 7.94 (d, *J* = 8.6 Hz, 2H), 7.33 – 7.19 (m, 2H), 4.48 (t, *J* = 7.1 Hz, 1H), 3.68 (d, *J* = 4.9 Hz, 6H), 2.52 (s, 3H), 2.44 (td, *J* = 7.0, 6.4, 4.1 Hz, 2H), 2.28 (q, *J* = 6.9 Hz, 2H). ^{13}C NMR (100 MHz, Chloroform-*d*) δ 193.7, 173.2, 170.1, 147.0, 132.0, 129.1, 125.0, 52.5, 52.3, 51.7, 31.2, 24.0, 14.6. HRMS (EI) m/z calcd. for $C_{15}H_{18}O_5S$ [M]$^+$: 310.0875, found : 310.0875

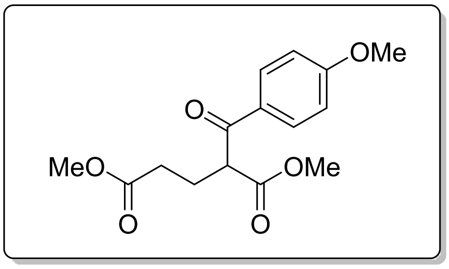

dimethyl 2-(4-methoxybenzoyl)pentanedioate (3ag). Prepared according to **GP1.** Purified by flash chromatography on silica gel (EtOAc/CH_2Cl_2/hexanes = 1:1:4). From 1-(1,5-dimethoxy-1,5-dioxopentan-2-yl)-2,4,6-triphenylpyridin-1-ium tetrafluoroborate (83.0 mg, 0.15 mmol), compound **3ag** (17.5 mg, 40%) was obtained. Colorless oil. ^{1}H NMR (600 MHz, Chloroform-*d*) δ 8.00 (d, *J* = 8.5 Hz, 2H), 6.94 (d, *J* = 8.5 Hz, 2H), 4.48 (t, *J* = 7.2 Hz, 1H), 3.86 (s, 3H), 3.67 (d, *J* = 8.0 Hz, 6H), 2.43 (h, *J* = 10.2, 9.7 Hz, 2H), 2.28 (q, *J* = 7.1 Hz, 2H). ^{13}C NMR (150 MHz, Chloroform-*d*) δ 193.2, 173.2, 170.2, 164.0, 131.1, 128.8, 114.0, 55.5, 52.4, 52.1, 51.6, 31.2, 24.0. HRMS (EI) m/z calcd. for $C_{15}H_{18}O_6$ $[M]^+$: 294.1103, found : 294.1103

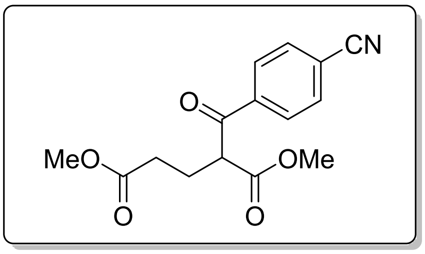

dimethyl 2-(4-cyanobenzoyl)pentanedioate (3ah). Prepared according to **GP1.** Purified by flash chromatography on silica gel (EtOAc/hexanes = 1:4). From 1-(1,5-dimethoxy-1,5-dioxopentan-2-yl)-2,4,6-triphenylpyridin-1-ium tetrafluoroborate (83.0 mg, 0.15 mmol), compound **3ah** (30.1 mg, 70%) was obtained. Colorless oil. ^{1}H NMR (400 MHz, Chloroform-*d*) δ 8.11 (d, *J* = 8.6 Hz, 2H), 7.78 (d, *J* = 8.7 Hz, 2H), 4.52 (t, *J* = 7.1 Hz, 1H), 3.68 (s, 3H), 3.66 (s, 3H), 2.51 – 2.37 (m, 2H), 2.27 (q, *J* = 7.1 Hz, 2H). ^{13}C NMR (100 MHz, Chloroform-*d*) δ 193.7, 173.1, 169.3, 138.7, 132.6, 129.1, 117.7, 116.9, 52.7, 52.6, 51.7, 30.9, 23.6. HRMS (EI) m/z calcd. for $C_{15}H_{15}NO_5$ $[M]^+$: 289.0950, found : 289.0948

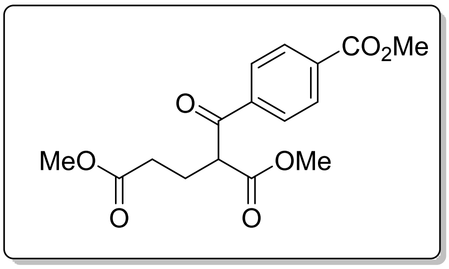

dimethyl 2-(4-(methoxycarbonyl)benzoyl)pentanedioate (3ai). Prepared according to **GP1.** Purified by flash chromatography on silica gel (EtOAc/hexanes = 1:4). From 1-(1,5-dimethoxy-1,5-dioxopentan-2-yl)-2,4,6-triphenylpyridin-1-ium tetrafluoroborate (83.0 mg, 0.15 mmol), compound **3ai**

(36.3 mg, 75%) was obtained. Yellow oil. ^{1}H NMR (400 MHz, Chloroform-*d*) δ 8.16 – 8.09 (m, 2H), 8.05 (d, *J* = 8.5 Hz, 2H), 4.53 (t, *J* = 7.1 Hz, 1H), 3.93 (s, 3H), 3.66 (d, *J* = 5.1 Hz, 6H), 2.48 – 2.39 (m, 2H), 2.28 (q, *J* = 6.8 Hz, 2H). ^{13}C NMR (100 MHz, Chloroform-*d*) δ 194.4, 173.1, 169.6, 166.0, 139.0, 134.3, 129.9, 128.6, 52.7, 52.6, 52.5, 51.7, 31.0, 23.8. HRMS (EI) m/z calcd. for $C_{16}H_{18}O_7$ $[M]^+$: 322.1053, found : 332.1054

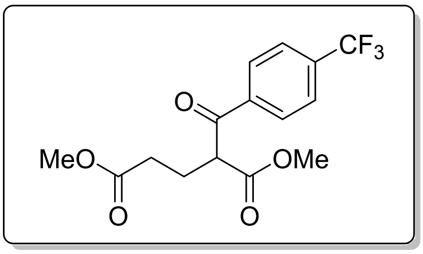

dimethyl 2-(4-(trifluoromethyl)benzoyl)pentanedioate (3aj). Prepared according to **GP1.** Purified by flash chromatography on silica gel (EtOAc/hexanes = 1:4). From 1-(1,5-dimethoxy-1,5-dioxopentan-2-yl)-2,4,6-triphenylpyridin-1-ium tetrafluoroborate (83.0 mg, 0.15 mmol), compound **3aj** (36.8 mg, 74%) was obtained. Colorless oil. ^{1}H NMR (600 MHz, Chloroform-*d*) δ 8.14 (d, *J* = 8.2 Hz, 2H), 7.75 (d, *J* = 8.2 Hz, 2H), 4.55 (t, *J* = 7.1 Hz, 1H), 3.69 (s, 3H), 3.67 (s, 3H), 2.51 – 2.41 (m, 2H), 2.35 – 2.25 (m, 2H). ^{13}C NMR (100 MHz, Chloroform-*d*) δ 194.0, 173.1, 169.6, 138.5, 134.9 (q, *J* = 32.7 Hz), 129.0, 125.9 (q, *J* = 3.8 Hz), 123.4 (q, *J* = 272.8 Hz), 52.7, 52.7, 51.7, 31.0, 23.7. ^{19}F NMR (375MHz, Chloroform-*d*) δ -63.27. HRMS (EI) m/z calcd. for $C_{15}H_{15}F_3O_5$ $[M]^+$: 332.0872, found : 332.0874

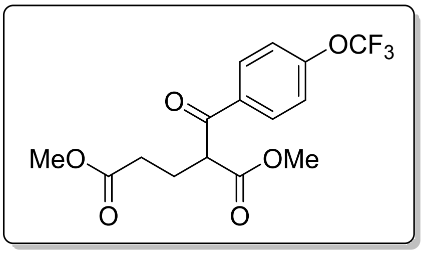

dimethyl 2-(4-(trifluoromethoxy)benzoyl)pentanedioate (3ak). Prepared according to **GP1.** Purified by flash chromatography on silica gel (EtOAc/hexanes = 1:4). From 1-(1,5-dimethoxy-1,5-dioxopentan-2-yl)-2,4,6-triphenylpyridin-1-ium tetrafluoroborate (83.0 mg, 0.15 mmol), compound **3ak** (37.7 mg, 72%) was obtained. Colorless oil. ^{1}H NMR (600 MHz, Chloroform-*d*) δ 8.13 – 8.06 (m, 2H), 7.33 – 7.27 (m, 2H), 4.51 (t, *J* = 7.1 Hz, 1H), 3.69 (s, 3H), 3.67 (s, 3H), 2.49 – 2.40 (m, 2H), 2.28 (q, *J* = 7.1 Hz, 2H). ^{13}C NMR (150 MHz, Chloroform-*d*) δ 193.4, 173.2, 169.7, 153.0, 134.0, 130.8, 121.1, 120.4, 52.6 (d, *J* = 3.1 Hz), 52.5, 51.7 (d, *J* = 3.2 Hz), 31.0, 23.8. ^{19}F NMR (375MHz, Chloroform-*d*) δ -57.61. HRMS (EI) m/z calcd. for $C_{15}H_{15}F_3O_6$ $[M]^+$: 348.0821, found : 348.0825

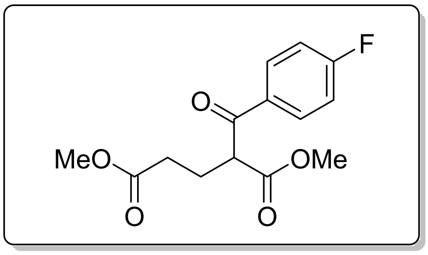

dimethyl 2-(4-fluorobenzoyl)pentanedioate (3al). Prepared according to **GP1.** Purified by flash chromatography on silica gel (EtOAc/hexanes = 1:4). From 1-(1,5-dimethoxy-1,5-dioxopentan-2-yl)-2,4,6-triphenylpyridin-1-ium tetrafluoroborate (83.0 mg, 0.15 mmol), compound **3al** (30.2 mg, 71%) was obtained. Colorless oil. ^{1}H NMR (600 MHz, Chloroform-*d*) δ 8.12 – 7.92 (m, 2H), 7.18 – 7.10 (m, 2H), 4.49 (t, *J* = 7.2 Hz, 1H), 3.68 (s, 3H), 3.66 (s, 3H), 2.49 – 2.38 (m, 2H), 2.31 – 2.20 (m, 2H). ^{13}C NMR (100 MHz, Chloroform-*d*) δ 193.3, 173.2, 169.8, 166.1 (d, *J* = 256.0 Hz), 132.2 (d, *J* = 3.0 Hz), 131.5 (d, *J* = 9.4 Hz), 115.9 (d, *J* = 22.0 Hz), 52.6, 52.4, 51.7, 31.0, 23.9. ^{19}F NMR (375MHz, Chloroform-*d*) δ -103.90. HRMS (EI) m/z calcd. for $C_{14}H_{15}FO_5$ $[M]^+$: 282.0904, found : 282.0903

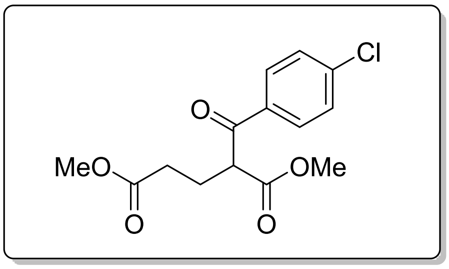

dimethyl 2-(4-chlorobenzoyl)pentanedioate (3am). Prepared according to **GP1.** Purified by flash chromatography on silica gel (EtOAc/hexanes = 1:5). From 1-(1,5-dimethoxy-1,5-dioxopentan-2-yl)-2,4,6-triphenylpyridin-1-ium tetrafluoroborate (83.0 mg, 0.15 mmol), compound **3am** (32.7 mg, 73%) was obtained. Yellow oil. ^{1}H NMR (400 MHz, Chloroform-*d*) δ 8.04 – 7.85 (m, 2H), 7.45 (d, *J* = 8.7 Hz, 2H), 4.48 (t, *J* = 7.1 Hz, 1H), 3.67 (d, *J* = 5.6 Hz, 6H), 2.44 (td, *J* = 7.0, 6.3, 2.5 Hz, 2H), 2.33 – 2.11 (m, 2H). ^{13}C NMR (100 MHz, Chloroform-*d*) δ 193.7, 173.1, 169.7, 140.3, 134.1, 130.1, 129.1, 52.6, 52.4, 51.7, 31.0, 23.8. HRMS (EI) m/z calcd. for $C_{14}H_{15}ClO_5$ $[M]^+$: 298.0608, found : 298.0611

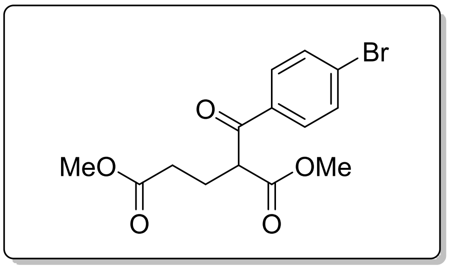

dimethyl 2-(4-bromobenzoyl)pentanedioate (3an). Prepared according to **GP1.** Purified by flash chromatography on silica gel (EtOAc/hexanes = 1:5). From 1-(1,5-dimethoxy-1,5-dioxopentan-2-yl)-2,4,6-triphenylpyridin-1-ium tetrafluoroborate (83.0 mg, 0.15 mmol), compound **3an** (37.5 mg, 73%)

was obtained. Yellow oil. ^{1}H NMR (600 MHz, Chloroform-*d*) δ 7.92 – 7.85 (m, 2H), 7.63 (dd, *J* = 8.6, 1.7 Hz, 2H), 4.48 (t, *J* = 7.1 Hz, 1H), 3.68 (dd, *J* = 8.0, 1.4 Hz, 6H), 2.44 (td, *J* = 6.9, 6.4, 5.0 Hz, 2H), 2.28 (q, *J* = 6.9 Hz, 2H). ^{13}C NMR (150 MHz, Chloroform-*d*) δ 193.9, 173.1, 169.7, 134.6, 132.1, 130.2, 129.1, 52.6 (d, *J* = 2.8 Hz), 52.4, 51.7 (d, *J* = 2.8 Hz), 31.0, 23.8. HRMS (EI) m/z calcd. for $C_{14}H_{15}BrO_5$ $[M]^+$: 342.0103, found : 342.0103

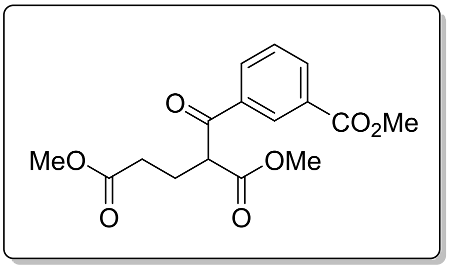

dimethyl 2-(3-(methoxycarbonyl)benzoyl)pentanedioate (3ao). Prepared according to **GP1.** Purified by flash chromatography on silica gel (EtOAc/hexanes = 1:5). From 1-(1,5-dimethoxy-1,5-dioxopentan-2-yl)-2,4,6-triphenylpyridin-1-ium tetrafluoroborate (83.0 mg, 0.15 mmol), compound **3ao** (33.8 mg, 70%) was obtained. Yellow oil. ^{1}H NMR (600 MHz, Chloroform-*d*) δ 8.65 (d, *J* = 1.9 Hz, 1H), 8.25 (dt, *J* = 7.8, 1.5 Hz, 1H), 8.20 (dt, *J* = 7.8, 1.4 Hz, 1H), 7.58 (t, *J* = 7.8 Hz, 1H), 4.56 (t, *J* = 7.1 Hz, 1H), 3.95 (d, *J* = 0.8 Hz, 3H), 3.68 (d, *J* = 7.2 Hz, 6H), 2.52 – 2.38 (m, 2H), 2.30 (q, *J* = 7.1 Hz, 2H). ^{13}C NMR (150 MHz, Chloroform-*d*) δ 194.1, 173.1, 169.7, 166.0, 136.2, 134.4, 132.8, 131.0, 129.7, 129.1, 52.6, 52.4, 52.4, 51.7, 31.1, 23.8. HRMS (EI) m/z calcd. for $C_{16}H_{18}O_7$ $[M]^+$: 322.1053, found : 332.1049

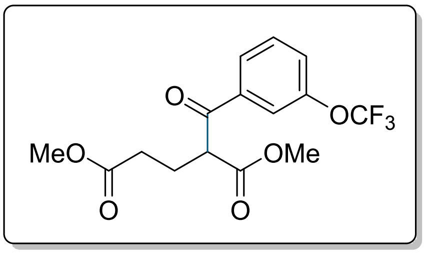

dimethyl 2-(3-(trifluoromethoxy)benzoyl)pentanedioate (3ap). Prepared according to **GP1.** Purified by flash chromatography on silica gel (EtOAc/hexanes = 1:4). From 1-(1,5-dimethoxy-1,5-dioxopentan-2-yl)-2,4,6-triphenylpyridin-1-ium tetrafluoroborate (83.0 mg, 0.15 mmol), compound **3ap** (36.9 mg, 71%) was obtained. Colorless oil. ^{1}H NMR (600 MHz, Chloroform-*d*) δ 7.97 (d, *J* = 7.8 Hz, 1H), 7.86 (s, 1H), 7.54 (t, *J* = 8.0 Hz, 1H), 7.44 (d, *J* = 8.2 Hz, 2H), 4.49 (t, *J* = 7.1 Hz, 1H), 3.69 (s, 3H), 3.67 (s, 3H), 2.50 – 2.40 (m, 2H), 2.30 (q, *J* = 7.1 Hz, 2H). ^{13}C NMR (150 MHz, Chloroform-*d*) δ 193.4, 173.1, 169.6, 149.6, 137.7, 130.4, 127.0, 126.0, 121.0, 120.4 (q, *J* = 258.3 Hz), 52.6, 52.6, 51.7, 31.0, 23.8. ^{19}F NMR (375MHz, Chloroform-*d*) δ -57.94. HRMS (EI) m/z calcd. for $C_{15}H_{15}F_3O_6$ $[M]^+$: 348.0821, found : 348.0823

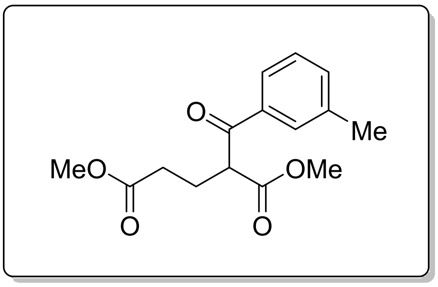

dimethyl 2-(3-methylbenzoyl)pentanedioate (3aq). Prepared according to **GP1.** Purified by flash chromatography on silica gel (EtOAc/hexanes = 1:20). From 1-(1,5-dimethoxy-1,5-dioxopentan-2-yl)-2,4,6-triphenylpyridin-1-ium tetrafluoroborate (83.0 mg, 0.15 mmol), compound **3aq** (23.1 mg, 55%) was obtained. Colorless oil. ^{1}H NMR (600 MHz, Chloroform-*d*) δ 7.85 – 7.77 (m, 2H), 7.44 – 7.33 (m, 2H), 4.51 (t, J = 7.1 Hz, 1H), 3.68 (s, 3H), 3.67 (s, 3H), 2.49 – 2.40 (m, 2H), 2.41 (s, 3H), 2.32 – 2.26 (m, 2H). ^{13}C NMR (150 MHz, Chloroform-*d*) δ 195.2, 170.2, 138.8, 136.0, 134.7, 129.3, 128.8, 126.1, 52.6, 52.6, 51.8, 31.4, 24.2, 21.5. HRMS (EI) m/z calcd. for $C_{15}H_{18}O_5$ $[M]^+$: 278.1154, found : 278.1156

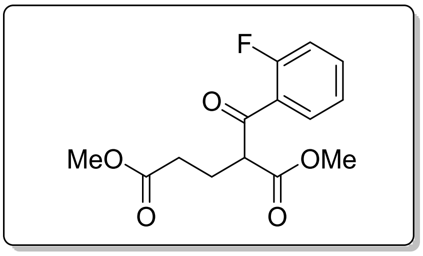

dimethyl 2-(2-fluorobenzoyl)pentanedioate (3ar). Prepared according to **GP1.** Purified by flash chromatography on silica gel (EtOAc/hexanes = 1:4). From 1-(1,5-dimethoxy-1,5-dioxopentan-2-yl)-2,4,6-triphenylpyridin-1-ium tetrafluoroborate (83.0 mg, 0.15 mmol), compound **3ar** (25.3 mg, 60%) was obtained. Colorless oil. ^{1}H NMR (400 MHz, Chloroform-*d*) δ 7.88 (td, *J* = 7.7, 1.9 Hz, 1H), 7.64 – 7.48 (m, 1H), 7.25 (t, *J* = 7.5 Hz, 1H), 7.20 – 7.05 (m, 1H), 4.36 (t, *J* = 6.9 Hz, 1H), 3.70 (s, 3H), 3.66 (s, 3H), 2.46 (t, *J* = 7.4 Hz, 2H), 2.34 – 2.24 (m, 2H). ^{13}C NMR (100 MHz, Chloroform-*d*) δ 193.1 (d, *J* = 3.7 Hz), 173.0, 169.9, 161.6 (d, *J* = 254.3 Hz), 135.2 (d, *J* = 9.2 Hz), 131.2 (d, *J* = 2.4 Hz), 124.8, 124.7 (d, *J* = 3.3 Hz), 116.7 (d, *J* = 23.8 Hz), 56.4 (d, *J* = 6.8 Hz), 52.4, 51.7, 31.6, 23.6. ^{19}F NMR (375MHz, Chloroform-*d*) δ -110.38. HRMS (EI) m/z calcd. for $C_{14}H_{15}FO_5$ $[M]^+$: 282.0904, found : 282.0902

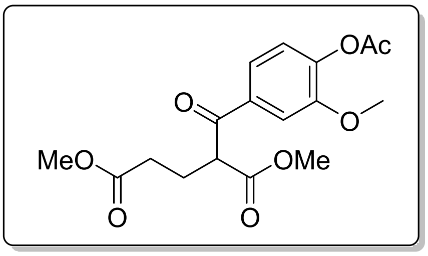

dimethyl 2-(4-acetoxy-3-methoxybenzoyl)pentanedioate (3as). Prepared according to **GP1.** Purified by flash chromatography on silica gel (EtOAc/hexanes = 1:4). From 1-(1,5-dimethoxy-1,5-dioxopentan-2-yl)-2,4,6-triphenylpyridin-1-ium tetrafluoroborate (83.0 mg, 0.15 mmol), compound **3as** (34.5 mg, 65%) was obtained. Colorless oil. ^{1}H NMR (600 MHz, Chloroform-*d*) δ 7.68 (d, *J* = 2.0 Hz, 1H), 7.65 (dd, *J* = 8.2, 2.0 Hz, 1H), 7.14 (d, *J* = 8.2 Hz, 1H), 4.51 (t, *J* = 7.2 Hz, 1H), 3.89 (s, 3H), 3.69 (s, 3H), 3.66 (s, 3H), 2.52 – 2.39 (m, 2H), 2.32 (s, 3H), 2.32 – 2.25 (m, 2H). ^{13}C NMR (150 MHz, Chloroform-*d*) δ 193.66, 173.15, 169.87, 168.31, 151.55, 144.33, 134.47, 123.00, 122.07, 112.22, 56.05, 52.56, 52.39, 51.65, 31.06, 24.02, 20.58. HRMS (EI) m/z calcd. for $C_{17}H_{20}O_8$ $[M]^+$: 352.1158, found : 352.1159

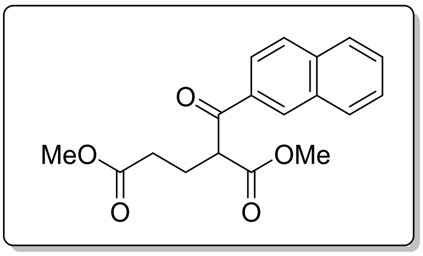

dimethyl 2-(2-naphthoyl)pentanedioate (3at). Prepared according to **GP1.** Purified by flash chromatography on silica gel (EtOAc/hexanes = 1:4). From 1-(1,5-dimethoxy-1,5-dioxopentan-2-yl)-2,4,6-triphenylpyridin-1-ium tetrafluoroborate (83.0 mg, 0.15 mmol), compound **3at** (28.9 mg, 62%) was obtained. Colorless oil. ^{1}H NMR (400 MHz, Chloroform-*d*) δ 8.59 (d, *J* = 1.8 Hz, 1H), 8.06 (dd, *J* = 8.7, 1.8 Hz, 1H), 8.00 (d, *J* = 8.1 Hz, 1H), 7.91 (d, *J* = 8.7 Hz, 1H), 7.88 (d, *J* = 8.0 Hz, 1H), 7.62 (ddd, *J* = 8.3, 6.9, 1.4 Hz, 1H), 7.57 (ddd, *J* = 8.2, 6.9, 1.4 Hz, 1H), 4.71 (t, *J* = 7.1 Hz, 1H), 3.70 (s, 3H), 3.68 (s, 3H), 2.53 – 2.45 (m, 2H), 2.42 – 2.31 (m, 2H). ^{13}C NMR (100 MHz, Chloroform-*d*) δ 194.8, 173.2, 170.1, 135.8, 133.2, 132.5, 130.8, 129.8, 128.9, 128.7, 127.7, 126.9, 124.0, 52.6, 52.5, 51.7, 31.2, 24.1. HRMS (EI) m/z calcd. for $C_{18}H_{18}O_5$ $[M]^+$: 314.1154, found : 314.1156

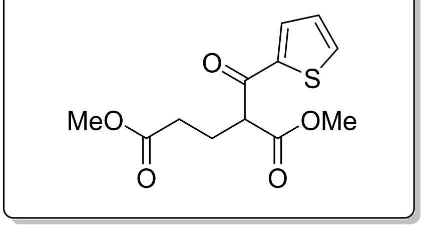

dimethyl 2-(thiophene-2-carbonyl)pentanedioate (3au). Prepared according to **GP1.** Purified by flash chromatography on silica gel (EtOAc/hexanes = 1:4). From 1-(1,5-dimethoxy-1,5-dioxopentan-2-yl)-2,4,6-triphenylpyridin-1-ium tetrafluoroborate (83.0 mg, 0.15 mmol), compound **3au** (20.4 mg, 50%) was obtained. Colorless oil. ^{1}H NMR (600 MHz, Chloroform-*d*) δ 7.87 (dd, *J* = 3.8, 1.1 Hz, 1H), 7.70 (dd, *J* = 4.9, 1.1 Hz, 1H), 7.16 (dd, *J* = 4.9, 3.8 Hz, 1H), 4.37 (t, *J* = 7.2 Hz, 1H), 3.70 (s, 3H), 3.67 (s, 3H), 2.51 – 2.37 (m, 2H), 2.36 – 2.22 (m, 2H). ^{13}C NMR (100 MHz, Chloroform-*d*) δ 187.4, 173.1, 169.6, 143.2, 135.2, 133.4, 128.4, 53.6, 52.6, 51.7, 31.1, 24.1. HRMS (EI) m/z calcd. for $C_{12}H_{14}O_5S$ $[M]^+$: 270.0562, found : 270.0561

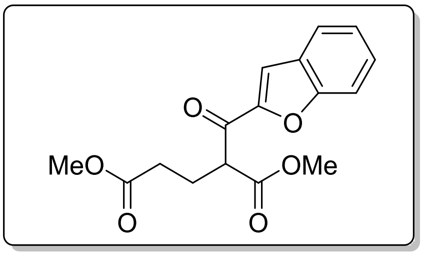

dimethyl 2-(benzofuran-2-carbonyl)pentanedioate (3av). Prepared according to **GP1.** Purified by flash chromatography on silica gel (EtOAc/hexanes = 1:4). From 1-(1,5-dimethoxy-1,5-dioxopentan-2-yl)-2,4,6-triphenylpyridin-1-ium tetrafluoroborate (83.0 mg, 0.15 mmol), compound **3av** (22.2 mg, 48%) was obtained. Colorless oil. ^{1}H NMR (400 MHz, Chloroform-*d*) δ 7.73 (d, *J* = 7.9 Hz, 1H), 7.67 (s, 1H), 7.58 (d, *J* = 8.4 Hz, 1H), 7.50 (t, *J* = 7.8 Hz, 1H), 7.32 (t, *J* = 7.5 Hz, 1H), 4.42 (t, *J* = 7.1 Hz, 1H), 3.71 (s, 3H), 3.67 (s, 3H), 2.47 (dd, *J* = 7.2, 5.7 Hz, 2H), 2.36 (q, *J* = 7.1 Hz, 2H). ^{13}C NMR (100 MHz, Chloroform-*d*) δ 185.3, 173.0, 169.5, 155.9, 151.5, 128.8, 126.9, 124.1, 123.5, 114.7, 112.5, 53.0, 52.7, 51.7, 31.1, 23.5. HRMS (EI) m/z calcd. for $C_{16}H_{16}O_6$ $[M]^+$: 304.0947, found : 304.0946

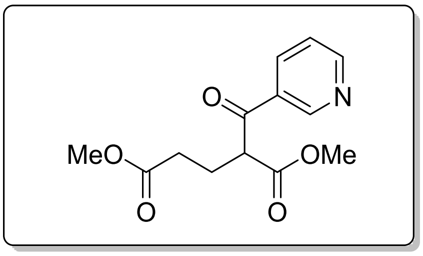

dimethyl 2-nicotinoylpentanedioate (3aw). Prepared according to **GP1.** Purified by flash chromatography on silica gel (EtOAc/hexanes = 1:4). From 1-(1,5-dimethoxy-1,5-dioxopentan-2-yl)-2,4,6-triphenylpyridin-1-ium tetrafluoroborate (83.0 mg, 0.15 mmol), compound **3aw** (23.2 mg, 57%) was obtained. Colorless oil. ^{1}H NMR (400 MHz, Chloroform-*d*) δ 9.22 (s, 1H), 8.80 (d, *J* = 4.0 Hz, 1H), 8.30 (dt, *J* = 8.0, 1.9 Hz, 1H), 7.44 (dd, *J* = 7.9, 4.8 Hz, 1H), 4.51 (t, *J* = 7.1 Hz, 1H), 3.69 (s, 3H), 3.66 (s, 3H), 2.51 – 2.39 (m, 2H), 2.30 (q, *J* = 7.1 Hz, 2H). ^{13}C NMR (100 MHz, Chloroform-*d*) δ 193.8, 173.0, 169.4, 153.9, 150.0, 136.1, 131.2, 123.7, 52.7, 52.7, 51.7, 31.0, 23.6. HRMS (EI) m/z calcd. for $C_{13}H_{15}NO_5$ $[M]^+$: 265.0950, found : 265.0950

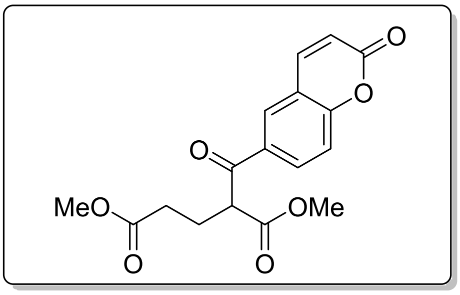

dimethyl 2-(2-oxo-2H-chromene-6-carbonyl)pentanedioate (3ax). Prepared according to **GP1.** Purified by flash chromatography on silica gel (EtOAc/hexanes = 1:1). From 1-(1,5-dimethoxy-1,5-dioxopentan-2-yl)-2,4,6-triphenylpyridin-1-ium tetrafluoroborate (83.0 mg, 0.15 mmol), compound **3ax** (33.7 mg, 67%) was obtained. Colorless oil. ^{1}H NMR (600 MHz, Chloroform-*d*) δ 8.24 (d, *J* = 2.2 Hz, 1H), 8.20 (dd, *J* = 8.7, 2.2 Hz, 1H), 7.79 (d, *J* = 9.6 Hz, 1H), 7.40 (d, *J* = 8.7 Hz, 1H), 6.49 (d, *J* = 9.6 Hz, 1H), 4.56 (t, *J* = 7.1 Hz, 1H), 3.69 (s, 3H), 3.67 (s, 3H), 2.51 – 2.43 (m, 2H), 2.37 – 2.21 (m, 2H). ^{13}C NMR (150 MHz, Chloroform-*d*) δ 193.0, 173.2, 169.6, 159.6, 157.2, 143.1, 132.1, 132.0, 129.3, 118.8, 117.7, 117.5, 52.7, 52.4, 51.7, 30.9, 23.8. HRMS (EI) m/z calcd. for $C_{17}H_{16}O_7$ $[M]^+$: 332.0896, found : 332.0897

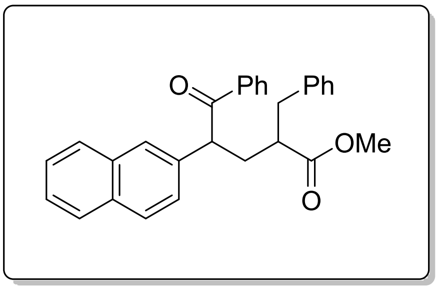

methyl 2-benzyl-4-(naphthalen-2-yl)-5-oxo-5-phenylpentanoate (5a). Prepared according to **GP2.** Purified by flash chromatography on silica gel (EtOAc/hexanes = 1:15). From (S)-1-(1-methoxy-1-oxo-3-phenylpropan-2-yl)-2,4,6-triphenylpyridin-1-ium tetrafluoroborate (83.6 mg, 0.15 mmol), compound **5a** (35.1 mg, 56%) was obtained. Colorless oil. ^{1}H NMR (600 MHz, Chloroform-*d*) δ 8.00 – 7.90 (m, 2H), 7.84 – 7.72 (m, 3H), 7.68 (s, 0.5H), 7.63 (s, 0.5H), 7.51 – 7.31 (m, 6H), 7.30 – 7.24 (m, 1H), 7.23 – 7.14 (m, 3H), 7.05 – 6.90 (m, 1H), 4.81 – 4.63 (m, 1H), 3.64 (s, 1.5H), 3.41 (s, 1.5H), 3.04 – 2.97 (m, 1H), 2.94 – 2.80 (m, 1H), 2.77 – 2.71 (m, 0.5H), 2.63 – 2.49 (m, 1.5H), 2.28 – 2.17 (m, 1H). ^{13}C NMR (100 MHz, Chloroform-*d*) δ [199.2, 199.1], [175.7, 175.7], [138.9, 138.7], [136.8, 136.6], [136.5, 136.0], [133.7, 133.7], [133.1, 133.1], [132.7, 132.6], 129.0, 129.0, 128.9, 128.9, 128.8, 128.6, [128.6, 128.5], [128.0, 127.9], 127.7, [127.6, 127.2], [126.6, 126.5], [126.4, 126.4], [126.3, 126.2], [126.1, 126.1], [51.7, 51.7], [51.6, 51.4], [45.8, 44.7], [39.1, 39.1], [36.6, 35.3]. HRMS (EI) m/z calcd. for $C_{29}H_{26}O_3$ $[M]^+$: 422.1882, found : 422.1884

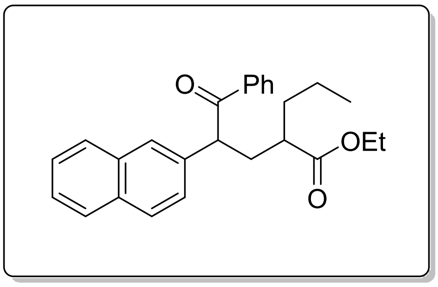

ethyl 4-(naphthalen-2-yl)-5-oxo-5-phenyl-2-propylpentanoate (5b). Prepared according to **GP2.** Purified by flash chromatography on silica gel (EtOAc/hexanes = 1:15). From 1-(1-ethoxy-1-oxopentan-2-yl)-2,4,6-triphenylpyridin-1-ium tetrafluoroborate (78.6 mg, 0.15 mmol), compound **5b** (35.6 mg, 64%) was obtained. Colorless oil. ^{1}H NMR (400 MHz, Chloroform-*d*) δ 8.06 – 7.94 (m, 2H), 7.84 – 7.76 (m, 3H), 7.76 – 7.66 (m, 1H), 7.50 – 7.39 (m, 4H), 7.41 – 7.32 (m, 2H), 4.78 (dd, J = 9.8, 4.7 Hz, 1H), 4.19 (q, J = 7.1 Hz, 1H), 4.08 – 3.91 (m, 1H), 2.65 – 2.40 (m, 1.5H), 2.31 – 2.02 (m, 1.5H), 1.67 (m, 1H), 1.60 – 1.48 (m, 1H), 1.45 – 1.20 (m, 5.5H), 1.10 (t, J = 7.1 Hz, 1.5H), 0.91 (t, J = 7.3 Hz, 1.5H), 0.80 (t, J = 7.3 Hz, 1.5H). ^{13}C NMR (100 MHz, Chloroform-*d*) δ [199.4, 199.3], [176.2, 176.1], [137.1, 137.0], [136.6, 136.3], [133.7, 133.7], [133.1, 133.0], [132.7, 132.6], [129.0, 128.9], [128.9, 128.8], 128.7, [127.9, 127.9], [127.8, 127.7], 127.0, 126.4, [126.3, 126.3], [126.2, 126.0],[60.4, 60.3], [51.8, 51.6], [43.9, 42.8], [37.0, 35.8], [35.3, 35.3], [20.5, 20.4], [14.5, 14.2], [14.1, 14.0]. HRMS (EI) m/z calcd. for $C_{26}H_{28}O_3$ $[M]^+$: 388.2038, found : 388.2036

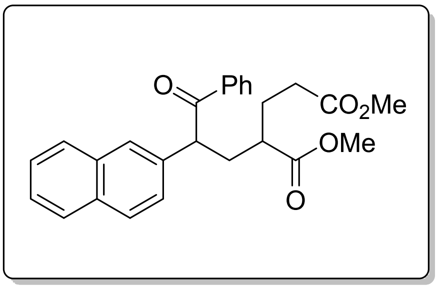

dimethyl 2-(2-(naphthalen-2-yl)-3-oxo-3-phenylpropyl)pentanedioate (5c). Prepared according to **GP2.** Purified by flash chromatography on silica gel (EtOAc/hexanes = 1:20). From 1-(1,5-dimethoxy-1,5-dioxopentan-2-yl)-2,4,6-triphenylpyridin-1-ium tetrafluoroborate (83.0 mg, 0.15 mmol), compound **5c** (39.2 mg, 63%) was obtained. Colorless oil. ^{1}H NMR (400 MHz, Chloroform-*d*) δ 8.02 – 7.93 (m, 2H), 7.83 – 7.74 (m, 3H), 7.74 – 7.68 (m, 1H), 7.51 – 7.40 (m, 4H), 7.40 – 7.32 (m, 2H), 4.86 – 4.75 (m, 1H), 3.69 (s, 1.4H), 3.66 (s, 1.6H), 3.53 (d, J = 1.4 Hz, 3H), 2.60 – 2.46 (m, 1.5H), 2.43 – 2.30 (m, 1.5H), 2.30 – 2.21 (m, 1H), 2.21 – 2.12 (m, 1H), 2.06 – 1.89 (m, 1.5H), 1.88 – 1.75 (m, 0.5H). ^{13}C NMR (100 MHz, Chloroform-*d*) δ [199.1, 199.0], [175.7, 175.6], [173.5, 173.3], [136.8, 136.6], [136.5, 136.1], 133.7, [133.1, 133.1], [132.7, 132.6], [129.1, 128.9], [128.8, 128.7], [127.9, 127.9], [127.8, 127.7], [127.6, 127.2], [126.4, 126.4], [126.2, 126.2], [126.1, 126.1], [51.9, 51.8], 51.7, [51.6, 51.5], [43.0, 42.2], [36.4, 35.7], [31.7, 31.6], [27.7, 27.6]. HRMS (EI) m/z calcd. for $C_{26}H_{26}O_5$ $[M]^+$: 418.1780,

found : 418.1778

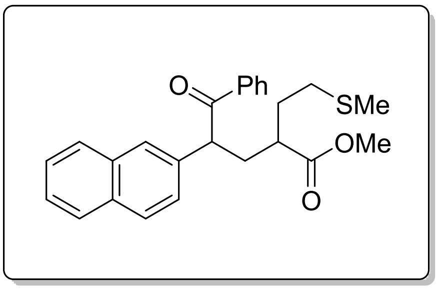

methyl 2-(2-(methylthio)ethyl)-4-(naphthalen-2-yl)-5-oxo-5-phenylpentanoate (5d). Prepared according to **GP2.** Purified by flash chromatography on silica gel (EtOAc/hexanes = 1:15). From 1-(1-methoxy-4-(methylthio)-1-oxobutan-2-yl)-2,4,6-triphenylpyridin-1-ium tetrafluoroborate (81.2 mg, 0.15 mmol), compound **5d** (36.8 mg, 60%) was obtained. Colorless oil. ^{1}H NMR (600 MHz, Methylene Chloride-*d₂*) δ 8.01 – 7.95 (m, 2H), 7.83 – 7.77 (m, 3H), 7.76 – 7.72 (m, 1H), 7.51 – 7.41 (m, 4H), 7.42 – 7.36 (m, 2H), 4.81 (dt, J = 9.1, 5.7 Hz, 1H), 3.67 (s, 1.5H), 3.52 (s, 1.5H), 2.65 – 2.57 (m, 0.5H), 2.56 – 2.44 (m, 2H), 2.43 – 2.34 (m, 1.5H), 2.19 – 2.11 (m, 1H), 2.08 (s, 1.5H), 2.02 – 1.90 (m, 2.5H), 1.89 – 1.80 (m, 0.5H), 1.78 – 1.69 (m, 0.5H). ^{13}C NMR (150 MHz, Methylene Chloride-d_2) δ [199.4, 199.3], [176.1, 176.0], [137.3, 137.3], [137.0, 136.8], [134.2, 134.2], [133.5, 133.5], [133.2, 133.1], [129.4, 129.3], [129.2, 129.2], [129.1, 129.1], [128.3, 128.2], 128.1, 128.1, 128.1, 127.6, 126.9, 126.8, 126.6, 126.5, 52.1, 52.1, 51.9, [43.4, 42.7], [36.8, 36.2], [32.8, 32.7], [32.2, 32.1], [15.7, 15.6]. HRMS (EI) m/z calcd. for $C_{25}H_{26}O_3S$ $[M]^+$: 406.1603, found : 406.1602

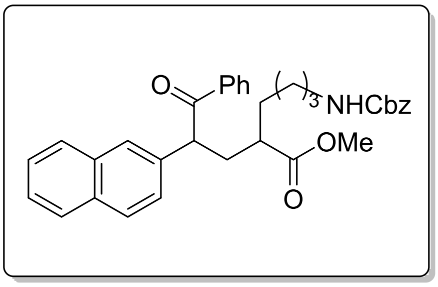

methyl 6-(((benzyloxy)carbonyl)amino)-2-(2-(naphthalen-2-yl)-3-oxo-3-phenylpropyl)hexanoate (5e). Prepared according to **GP2.** Purified by flash chromatography on silica gel (EtOAc/hexanes = 1:8). From 1-(6-(((benzyloxy)carbonyl)amino)-1-methoxy-1-oxohexan-2-yl)-2,4,6-triphenylpyridin-1-ium tetrafluoroborate (101.0 mg, 0.15 mmol), compound **5e** (45.7 mg, 57%) was obtained. Colorless oil. ^{1}H NMR (600 MHz, Acetonitrile-d_3) δ 8.06 – 7.95 (m, 2H), 7.84 – 7.71 (m, 4H), 7.57 – 7.38 (m, 6H), 7.37 – 7.25 (m, 5H), 5.60 (s, 0.6H), 5.51 (s, 0.4H), 5.03 (s, 1.2H), 5.01 (s, 0.8H), 4.89 – 4.78 (m, 1H), 3.62 (s, 1.4H), 3.44 (s, 1.6H), 3.10 – 3.03 (m, 1.2H), 2.98 (q, J = 6.7 Hz, 0.8H), 2.54 – 2.41 (m, 1H), 2.42 – 2.33 (m, 0.6H), 2.24 – 2.18 (m, 0.4H), 2.13 – 2.05 (m, 1H), 1.68 – 1.53 (m, 2H), 1.51 – 1.38 (m, 2H), 1.37 – 1.23 (m, 3H), 1.21 – 1.12 (m, 1H). ^{13}C NMR (150 MHz, Acetonitrile-d_3) δ 199.2, [175.8,

175.7], 156.4, [137.6, 136.9], [136.8, 136.6], 133.6, 133.0, 132.5, 128.9, 128.6, 128.5, 128.4, 127.7, 127.6, 127.5, 127.5, 127.2, 126.4, 126.3, 126.0, 65.7, [51.4, 51.2], [51.0, 50.8], [43.5, 43.0], 40.3, [35.9, 35.3], [32.1, 31.9], 29.4, [24.0, 24.0]. HRMS (EI) m/z calcd. for $C34H_{35}NO_5$ $[M]^+$: 537.2515, found : 537.2513

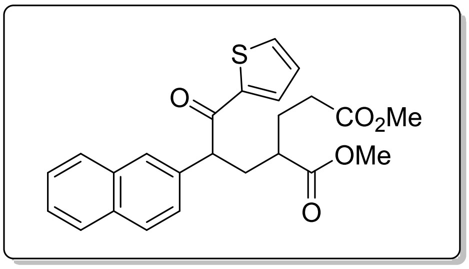

dimethyl 2-(2-(naphthalen-2-yl)-3-oxo-3-(thiophen-2-yl)propyl)pentanedioate (5f). Prepared according to **GP2.** Purified by flash chromatography on silica gel (EtOAc/hexanes = 1:6). 1-(1,5-dimethoxy-1,5-dioxopentan-2-yl)-2,4,6-triphenylpyridin-1-ium tetrafluoroborate (83.0 mg, 0.15 mmol), compound **5f** (42.3 mg, 66%) was obtained. Colorless oil. ^{1}H NMR (600 MHz, Chloroform-*d*) δ 7.83 – 7.69 (m, 5H), 7.54 (ddd, *J* = 4.8, 3.7, 1.1 Hz, 1H), 7.50 – 7.39 (m, 3H), 7.07 – 6.94 (m, 1H), 4.76 – 4.42 (m, 1H), [3.69 (s, 1.5H), 3.66 (s, 1.5H)], [3.55 (s, 1.5H), 3.54 (s, 1.5H)], 2.64 – 2.29 (m, 3H), 2.29 – 2.23 (m, 1H), 2.21 – 2.13 (m, 1H), 2.03 – 1.78 (m, 2H). ^{13}C NMR (150 MHz, Chloroform-*d*) δ [191.8, 191.7], [175.4, 175.3], [173.2, 173.1], [143.8, 143.5], [136.4, 135.9], [133.9, 133.8], 133.5, [132.65, 132.61], [132.58, 132.55], [128.9, 128.8], 128.1, [127.8, 127.7], [127.62, 127.59], [127.4, 127.0], [126.28, 126.26], [126.03, 126.01], [125.9, 125.8], [53.0, 52.8], [51.7, 51.6], [51.54, 51.49], [42.8, 42.0], [35.9, 35.2], [31.5, 31.4], [27.5, 27.4]. HRMS (EI) m/z calcd. for $C_{24}H_{24}O_5S$ $[M]^+$: 424.1344, found : 424.1343

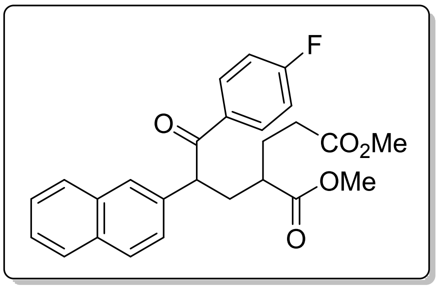

dimethyl 2-(3-(4-fluorophenyl)-2-(naphthalen-2-yl)-3-oxopropyl)pentanedioate (5g). Prepared according to **GP2.** Purified by flash chromatography on silica gel (EtOAc/hexanes = 1:8). From 1-(1,5-dimethoxy-1,5-dioxopentan-2-yl)-2,4,6-triphenylpyridin-1-ium tetrafluoroborate (83.0 mg, 0.15 mmol), compound **5g** (41.2 mg, 63%) was obtained. Colorless oil. ^{1}H NMR (600 MHz, Chloroform-*d*) δ 8.05 – 7.95 (m, 2H), 7.84 – 7.74 (m, 3H), 7.71 (s, 1H), 7.49 – 7.37 (m, 3H), 7.05 – 6.97 (m, 2H), 4.75 (dt, J = 9.0, 5.8 Hz, 1H), 3.69 (s, 1.5H), 3.66 (s, 1.5H), 3.54 (s, 1.5H), 3.54 (s, 1.5H), 2.59 – 2.47 (m, 1.5H), 2.42 – 2.35 (m, 1H), 2.33 – 2.23 (m, 1.5H), 2.16 (m, 1H), 2.05 – 1.90 (m, 1.5H), 1.86 – 1.80 (m,

0.5H). ^{13}C NMR (150 MHz, Chloroform-*d*) δ [197.4, 197.4], [175.6, 175.5], [173.4, 173.2], [165.6 (d, J = 255.1 Hz), 165.6 (d, J = 255.0 Hz)], [136.4, 135.9], [133.7, 133.7], [133.1 (d, J = 3.0 Hz), 132.8 (d, J = 2.9 Hz)], [132.7, 132.6], [131.5 (d, J = 11.1 Hz), 131.4 (d, J = 11.1 Hz)], 129.2, [127.9, 127.8], [127.8, 127.7], [127.5, 127.1], [126.5, 126.5], [126.2, 126.2], [126.1, 125.9], [115.7 (d, J = 21.9 Hz), 115.7 (d, J = 21.9 Hz)], [51.8, 51.7], [51.6, 51.6], [51.6, 51.5], [43.0, 42.1], [36.3, 35.6], [31.7, 31.6], [27.7, 27.6]. ^{19}F NMR (375 MHz, Chloroform-*d*) δ -105.03, -105.12. HRMS (EI) m/z calcd. for $C_{26}H_{25}FO_5$ $[M]^+$: 436.1686, found : 436.1689

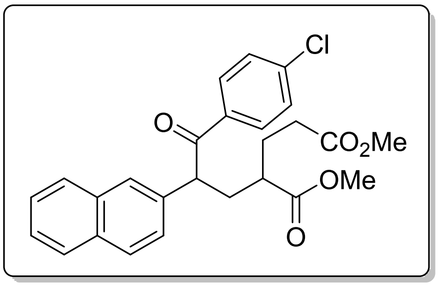

dimethyl 2-(3-(4-chlorophenyl)-2-(naphthalen-2-yl)-3-oxopropyl)pentanedioate (5h). Prepared according to **GP2.** Purified by flash chromatography on silica gel (EtOAc/hexanes = 1:8). From 1-(1,5-dimethoxy-1,5-dioxopentan-2-yl)-2,4,6-triphenylpyridin-1-ium tetrafluoroborate (83.0 mg, 0.15 mmol), compound **5h** (45.2 mg, 66%) was obtained. Colorless oil. ^{1}H NMR (600 MHz, Chloroform-*d*) δ 7.94 – 7.85 (m, 2H), 7.83 – 7.75 (m, 3H), 7.69 (s, 1H), 7.54 – 7.41 (m, 2H), 7.41 – 7.34 (m, 1H), 7.34 – 7.29 (m, 3H), 4.74 (dt, J = 8.7, 5.9 Hz, 1H), 3.69 (s, 1.5H), 3.66 (s, 1.5H), 3.54 (s, 1.5H), 3.54 (s, 1.5H), 2.61 – 2.47 (m, 1.5H), 2.40 – 2.33 (m, 1H), 2.33 – 2.22 (m, 1.5H), 2.21 – 2.13 (m, 1H), 2.08 – 1.90 (m, 1.5H), 1.88 – 1.76 (m, 0.5H). ^{13}C NMR (150 MHz, Chloroform-*d*) δ [197.8, 197.8], [175.6, 175.5], [173.4, 173.2], [139.5, 139.5], [136.3, 135.8], [135.0, 134.8], [133.7, 133.7], [132.7, 132.7], [130.3, 130.2], 129.2, [129.0, 129.0], [127.9, 127.8], [127.8, 127.7], [127.6, 127.2], [126.5, 126.5], [126.3, 126.2], [126.0, 125.9], [51.9, 51.8], [51.7, 51.7], [51.6, 51.6], [43.0, 42.1], [36.3, 35.5], [31.7, 31.6], [27.7, 27.6]. HRMS (EI) m/z calcd. for $C_{28}H_{25}ClO_5$ $[M]^+$: 452.1391, found : 452.1390

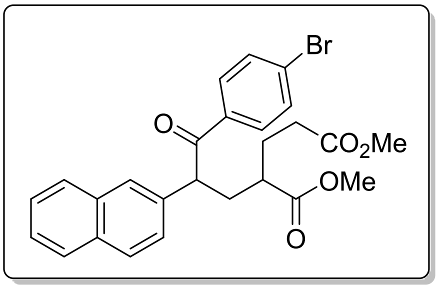

dimethyl 2-(3-(4-bromophenyl)-2-(naphthalen-2-yl)-3-oxopropyl)pentanedioate (5i). Prepared according to **GP2.** Purified by flash chromatography on silica gel (EtOAc/hexanes = 1:8). From 1-(1,5-dimethoxy-1,5-dioxopentan-2-yl)-2,4,6-triphenylpyridin-1-ium tetrafluoroborate (83.0 mg, 0.15

mmol), compound **5i** (52.4 mg, 70%) was obtained. Colorless oil. ^{1}H NMR (600 MHz, Chloroform-*d*) δ 7.86 – 7.79 (m, 2H), 7.80 – 7.75 (m, 3H), 7.69 (s, 1H), 7.54 – 7.46 (m, 2H), 7.47 – 7.41 (m, 2H), 7.40 – 7.35 (m, 1H), 4.73 (dt, J = 8.7, 5.9 Hz, 1H), 3.69 (s, 1.5H), 3.66 (s, 1.5H), 3.54 (s, 1.5H), 3.54 (s, 1.5H), 2.58 – 2.45 (m, 1.5H), 2.43 – 2.33 (m, 1H), 2.33 – 2.22 (m, 1.5H), 2.19 – 2.12 (m, 1H), 2.06 – 1.90 (m, 1.5H), 1.86 – 1.77 (m, 0.5H). ^{13}C NMR (150 MHz, Chloroform-*d*) δ [198.0, 198.0], [175.6, 175.5], [173.4, 173.2], [136.2, 135.7], [135.4, 135.2], 133.7, [132.7, 132.7], [132.0, 131.9], [130.4, 130.3], 129.2, [128.3, 128.3], [127.9, 127.8], [127.8, 127.7], [127.6, 127.2], [126.5, 126.5], [126.3, 126.2], [126.0, 125.9], [51.9, 51.8], 51.7, [51.6, 51.6], [42.9, 42.1], [36.2, 35.5], [31.7, 31.6], [27.7, 27.6]. HRMS (EI) m/z calcd. for $C_{28}H_{25}BrO_5$ $[M]^+$: 496.0885, found : 496.0887

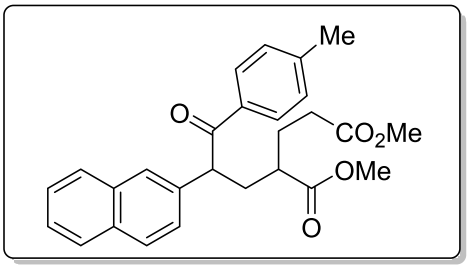

dimethyl 2-(2-(naphthalen-2-yl)-3-oxo-3-(p-tolyl)propyl)pentanedioate (5j). Prepared according to **GP2.** Purified by flash chromatography on silica gel (EtOAc/hexanes = 1:6). 1-(1,5-dimethoxy-1,5-dioxopentan-2-yl)-2,4,6-triphenylpyridin-1-ium tetrafluoroborate (83.0 mg, 0.15 mmol), compound **5j** (41.6mg, 64%) was obtained. Colorless oil. ^{1}H NMR (600 MHz, Chloroform-*d*) δ 7.93 – 7.85 (m, 2H), 7.83 – 7.74 (m, 3H), 7.74 – 7.68 (m, 1H), 7.49 – 7.41 (m, 3H), 7.16 (d, *J* = 8.1 Hz, 2H), 4.93 – 4.66 (m, 1H), [3.69 (s, 1.5H), 3.66 (s, 1.5H)], 3.54 (s, 3H), 2.58 – 2.46 (m, 2H), 2.43 – 2.34 (m, 1H), 2.32 (s, 3H), 2.29 – 2.23 (m, 1H), 2.21 – 2.13 (m, 1H), 2.04 – 1.79 (m, 2H). ^{13}C NMR (150 MHz, Chloroform-*d*) δ [198.5, 198.4], [175.5, 175.4], [173.3, 173.1], [143.8, 143.7], [136.7, 136.2], [134.1, 133.8], 133.6, [132.52, 132.45], [129.19, 129.18], [128.85, 128.83], 128.8, [127.8, 127.7], [127.58, 127.55], [127.4, 127.0], [126.18, 126.17], [126.10, 125.92], [125.91, 125.88], [51.65, 51.59], 51.5, [51.3, 51.2], [42.9, 42.1], [36.2, 35.5], [31.6, 31.5], [27.6, 27.5], 21.5. HRMS (EI) m/z calcd. for $C_{27}H_{28}O_5$ $[M]^+$: 432.1937, found : 432.1939

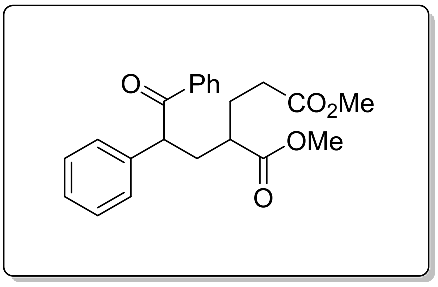

dimethyl 2-(3-oxo-2,3-diphenylpropyl)pentanedioate (5k). Prepared according to **GP2.** Purified by flash chromatography on silica gel (EtOAc/hexanes = 1:8). From 1-(1,5-dimethoxy-1,5-dioxopentan-2-

yl)-2,4,6-triphenylpyridin-1-ium tetrafluoroborate (83.0 mg, 0.15 mmol), compound **5k** (26.5 mg, 48%) was obtained. Colorless oil. ^{1}H NMR (400 MHz, Chloroform-*d*) δ 7.96 – 7.91 (m, 2H), 7.53 – 7.43 (m, 1H), 7.42 – 7.33 (m, 2H), 7.33 – 7.23 (m, 3H), 7.25 – 7.15 (m, 2H), 4.62 (dd, J = 9.0, 5.6 Hz, 1H), 3.67 (s, 1.5H), 3.66 (s, 1.5H), 3.59 (s, 1.5H), 3.55 (s, 1.5H), 2.60 – 2.39 (m, 1.5H), 2.38 – 2.29 (m, 1H), 2.32 – 2.22 (m, 1.5H), 2.16 – 2.02 (m, 1H), 2.01 – 1.88 (m, 1.5H), 1.85 – 1.75 (m, 0.5H). ^{13}C NMR (100 MHz, Chloroform-*d*) δ 199.1, [175.7, 175.6], [173.4, 173.3], [139.1, 138.6], [136.8, 136.5], [133.1, 133.1], 129.2, [128.9, 128.8], 128.7, [128.5, 128.3], [127.5, 127.4], [51.8, 51.8], [51.7, 51.7], [51.5, 51.4], [43.1, 42.2], [36.4, 35.7], [31.7, 31.7], [27.7, 27.6]. HRMS (EI) m/z calcd. for $C_{22}H_{24}O_5$ $[M]^+$: 368.1624, found : 368.1624

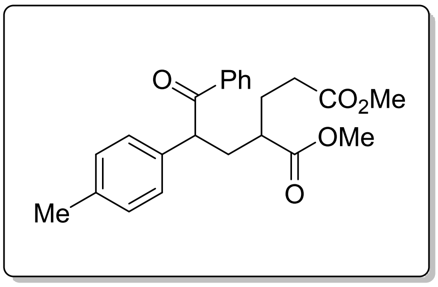

dimethyl 2-(3-oxo-3-phenyl-2-(p-tolyl)propyl)pentanedioate (5l). Prepared according to **GP2.** Purified by flash chromatography on silica gel (EtOAc/hexanes = 1:20). From 1-(1,5-dimethoxy-1,5-dioxopentan-2-yl)-2,4,6-triphenylpyridin-1-ium tetrafluoroborate (83.0 mg, 0.15 mmol), compound **5l** (26.4 mg, 46%) was obtained. Colorless oil. ^{1}H NMR (600 MHz, Chloroform-*d*) δ 7.95 – 7.90 (m, 2H), 7.50 – 7.44 (m, 1H), 7.41 – 7.33 (m, 2H), 7.17 – 7.06 (m, 4H), 4.61 – 4.54 (m, 1H), 3.67 (s, 1.5H), 3.66 (s, 1.5H), 3.60 (s, 1.5H), 3.55 (s, 1.5H), 2.49 – 2.37 (m, 1.5H), 2.36 – 2.29 (m, 2H), 2.29 – 2.24 (m, 3.5H), 2.12 – 1.98 (m, 1H), 2.00 – 1.88 (m, 1.5H), 1.85 – 1.75 (m, 0.5H). ^{13}C NMR (150 MHz, Chloroform-*d*) δ [199.2, 199.2], [175.7, 175.6], [173.5, 173.3], [137.1, 137.1], [136.8, 136.6], [136.1, 135.5], [133.0, 133.0], 129.9, [128.9, 128.8], [128.6, 128.6], [128.4, 128.1], [51.8, 51.8], [51.7, 51.6], [51.1, 51.0], [43.0, 42.2], [36.5, 35.7], [31.8, 31.7], [27.8, 27.6], [21.2, 21.13]. HRMS (EI) m/z calcd. for $C_{23}H_{26}O_5$ $[M]^+$: 382.1780, found : 382.1781

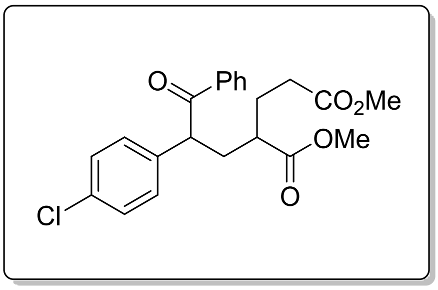

dimethyl 2-(2-(4-chlorophenyl)-3-oxo-3-phenylpropyl)pentanedioate (5m). Prepared according to **GP2.** Purified by flash chromatography on silica gel (EtOAc/hexanes = 1:20). From 1-(1,5-dimethoxy-

1,5-dioxopentan-2-yl)-2,4,6-triphenylpyridin-1-ium tetrafluoroborate (83.0 mg, 0.15 mmol), compound **5m** (28.5 mg, 47%) was obtained. Colorless oil. ^{1}H NMR (400 MHz, Chloroform-*d*) δ 8.00 – 7.90 (m, 2H), 7.58 – 7.51 (m, 1H), 7.50 – 7.39 (m, 2H), 7.34 – 7.21 (m, 3H), 4.65 (dd, J = 9.0, 5.5 Hz, 1H), 3.72 (s, 1.5H), 3.70 (s, 1.5H), 3.64 (s, 1.5H), 3.60 (s, 1.5H), 2.60 – 2.41 (m, 1.5H), 2.40 – 2.34 (m, 1H), 2.35 – 2.26 (m, 1.5H), 2.15 – 2.03 (m, 1H), 2.03 – 1.92 (m, 1.5H), 1.90 – 1.77 (m, 0.5H). ^{13}C NMR (150 MHz, Chloroform-*d*) δ 198.8, [175.5, 175.4], [173.4, 173.2], [137.6, 137.1], [136.5, 136.2], [133.5, 133.4], [133.3, 133.3], [129.9, 129.6], [129.4, 129.4], 128.8, 128.8, 128.7, [51.9, 51.8], [51.8, 51.7], [50.7, 50.6], [43.0, 42.2], [36.3, 35.6], [31.7, 31.6], [27.7, 27.6]. HRMS (EI) m/z calcd. for $C_{22}H_{23}ClO_5$ $[M]^+$: 402.1234, found : 402.1230

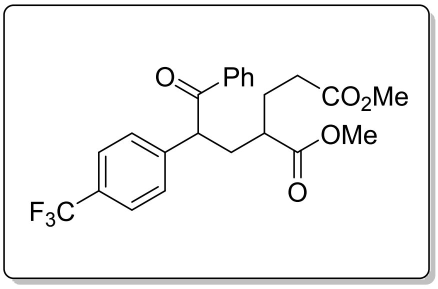

dimethyl 2-(3-oxo-3-phenyl-2-(4-(trifluoromethyl)phenyl)propyl)pentanedioate (5n). Prepared according to **GP2.** Purified by flash chromatography on silica gel (EtOAc/hexanes = 1:8). From 1-(1,5-dimethoxy-1,5-dioxopentan-2-yl)-2,4,6-triphenylpyridin-1-ium tetrafluoroborate (83.0 mg, 0.15 mmol), compound **5n** (37.3 mg, 57%) was obtained. Colorless oil. ^{1}H NMR (600 MHz, Chloroform-*d*) δ 7.95 – 7.88 (m, 2H), 7.61 – 7.48 (m, 3H), 7.46 – 7.37 (m, 4H), 4.72 (ddd, J = 8.4, 5.4, 2.9 Hz, 1H), 3.68 (s, 1.5H), 3.66 (s, 1.5H), 3.59 (s, 1.5H), 3.55 (s, 1.5H), 2.57 – 2.41 (m, 1.5H), 2.40 – 2.31 (m, 1H), 2.31 – 2.19 (m, 1.5H), 2.15 – 2.01 (m, 1H), 2.01 – 1.87 (m, 1.5H), 1.85 – 1.76 (m, 1H). ^{13}C NMR (150 MHz, Chloroform-*d*) δ [198.5, 198.5], [175.4, 175.3], [173.3, 173.2], [143.1, 142.6], [136.4, 136.1], [133.5, 133.5], 129.8 (dd, J = 32.5, 12.1 Hz), 129.0, 128.8, 128.8, 128.7, 126.3 – 125.9 (m), 128.5 – 123.1 (m), [51.9, 51.8], 51.7, [51.2, 51.0], [43.0, 42.2], [36.3, 35.7], [31.6, 31.6], [27.7, 27.6]. ^{19}F NMR (375 MHz, Chloroform-*d*) δ -62.62, -62.63. HRMS (EI) m/z calcd. for $C_{23}H_{23}F_3O_5$ $[M]^+$: 436.1498, found : 436.1501

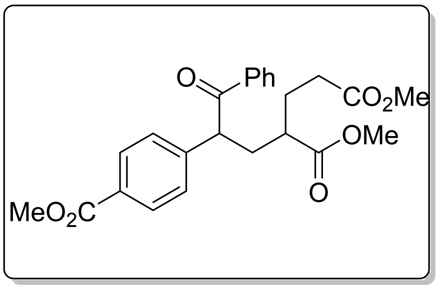

dimethyl 2-(2-(4-(methoxycarbonyl)phenyl)-3-oxo-3-phenylpropyl)pentanedioate (5o). Prepared

according to **GP2.** Purified by flash chromatography on silica gel (EtOAc/hexanes = 1:4). From 1-(1,5-dimethoxy-1,5-dioxopentan-2-yl)-2,4,6-triphenylpyridin-1-ium tetrafluoroborate (83.0 mg, 0.15 mmol), compound **5o** (39.8 mg, 62%) was obtained. Colorless oil. ^{1}H NMR (600 MHz, Chloroform-*d*) δ 7.98 – 7.92 (m, 2H), 7.93 – 7.87 (m, 2H), 7.52 – 7.45 (m, 1H), 7.45 – 7.33 (m, 4H), 4.69 (dt, J = 8.6, 5.3 Hz, 1H), 3.87 (s, 1.5H), 3.86 (s, 1.5H), 3.67 (s, 1.5H), 3.66 (s, 1.5H), 3.59 (s, 1.5H), 3.55 (s, 1.5H), 2.53 – 2.40 (m, 1.5H), 2.39 – 2.30 (m, 1H), 2.30 – 2.20 (m, 1.5H), 2.11 – 2.01 (m, 1H), 2.01 – 1.86 (m, 1.5H), 1.84 – 1.76 (m, 0.5H). ^{13}C NMR (150 MHz, Chloroform-*d*) δ [198.5, 198.5], [175.5, 175.4], [173.4, 173.2], [166.8, 166.8], [136.5, 136.2], [133.4, 133.4], [130.5, 130.5], [129.5, 129.4], 128.8, 128.8, 128.8, 128.6, 128.3, 52.2, 51.9, 51.8, 51.7, [51.5, 51.4], [43.0, 42.2], [36.2, 35.5], [31.7, 31.6], [27.7, 27.6]. HRMS (EI) m/z calcd. for $C_{24}H_{26}O_7$ $[M]^+$: 426.1679, found : 426.1676

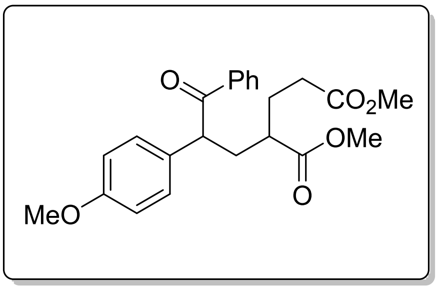

dimethyl 2-(2-(4-methoxyphenyl)-3-oxo-3-phenylpropyl)pentanedioate (5p). Prepared according to **GP2.** Purified by flash chromatography on silica gel (EtOAc/hexanes = 1:4). From 1-(1,5-dimethoxy-1,5-dioxopentan-2-yl)-2,4,6-triphenylpyridin-1-ium tetrafluoroborate (83.0 mg, 0.15 mmol), compound **5p** (20.1 mg, 34%) was obtained. Colorless oil. ^{1}H NMR (600 MHz, Chloroform-*d*) δ 7.95 – 7.89 (m, 2H), 7.50 – 7.44 (m, 1H), 7.41 – 7.34 (m, 2H), 7.22 – 7.14 (m, 2H), 6.90 – 6.78 (m, 2H), 4.56 (dt, J = 9.2, 5.2 Hz, 1H), 3.75 (s, 1.4H), 3.74 (s, 1.6H), 3.67 (s, 1.4H), 3.66 (s, 1.6H), 3.60 (s, 1.4H), 3.55 (s, 1.6H), 2.49 – 2.39 (m, 1.4H), 2.38 – 2.32 (m, 1H), 2.31 – 2.23 (m, 1.6H), 2.09 – 2.00 (m, 1H), 1.99 – 1.87 (m, 1.4H), 1.85 – 1.76 (m, 0.6H). ^{13}C NMR (150 MHz, Chloroform-*d*) δ [199.3, 199.3], [175.7, 175.6], [173.5, 173.3], [159.0, 158.9], [136.9, 136.6], [133.0, 133.0], [131.1, 130.5], [129.6, 129.3], [128.9, 128.8], 128.6, 114.6, 55.4, [51.8, 51.7], [50.6, 50.5], [43.0, 42.2], [36.4, 35.7], [31.8, 31.7], [27.8, 27.6]. HRMS (EI) m/z calcd. for $C_{23}H_{26}O_6$ $[M]^+$: 398.1729, found : 398.1729

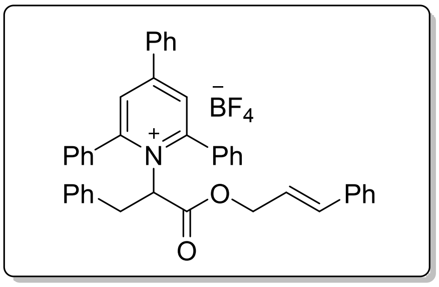

1-(1-(cinnamyloxy)-1-oxo-3-phenylpropan-2-yl)-2,4,6-triphenylpyridin-1-ium tetrafluoroborate

(6). Prepared according to **GP3.** Purified by flash chromatography on silica gel (MeOH/CH_2Cl_2 = 1:100). Yellow solid. ^{1}H NMR (600 MHz, Chloroform-*d*) δ 7.98 (s, 2H), 7.91 – 7.83 (m, 2H), 7.64 – 7.43 (m, 10H), 7.39 – 7.34 (m, 4H), 7.35 – 7.29 (m, 1H), 7.12 – 7.04 (m, 3H), 6.89 – 6.78 (m, 2H), 6.56 (d, J = 15.8 Hz, 1H), 6.11 (dt, J = 15.9, 6.7 Hz, 1H), 5.69 (dd, J = 8.5, 3.3 Hz, 1H), 4.77 – 4.65 (m, 2H), 3.56 (dd, J = 14.4, 3.3 Hz, 1H), 2.82 (dd, J = 14.4, 8.5 Hz, 1H). ^{13}C NMR (100 MHz, Chloroform-*d*) δ 167.8, 157.2, 136.7, 136.6, 135.6, 134.0, 132.6, 132.5, 131.6, 129.8, 129.2, 128.9, 128.9, 128.7, 128.7, 127.3, 126.8, 120.9, 70.7, 67.8, 37.8. ^{19}F NMR (375 MHz, Chloroform-*d*) δ -152.74, -152.80. HRMS (FAB) m/z calcd. for $C_{41}H_{34}NO_2^+$ $[M-BF_4^-]^+$: 572.2584, found : 572.2594

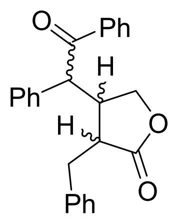

3-benzyl-4-(2-oxo-1,2-diphenylethyl)dihydrofuran-2(3H)-one (7). Prepared according to **GP1.** Purified by flash chromatography on silica gel (EtOAc/hexanes = 1:2). From 1-(1-(cinnamyloxy)-1-oxo-3-phenylpropan-2-yl)-2,4,6-triphenylpyridin-1-ium tetrafluoroborate (65.9 mg, 0.1 mmol), compound **7** (11.0 mg, 30%) was obtained. Yellow solid. ^{1}H NMR (600 MHz, Chloroform-*d*) δ 7.89 – 7.79 (m, 2H), 7.50 – 7.45 (m, 1H), 7.39 – 7.33 (m, 2H), 7.26 – 7.20 (m,35H), 7.20 – 7.14 (m, 3H), 7.13 – 7.07 (m, 2H), 6.94 – 6.79 (m, 2H), 4.48 (d, J = 10.6 Hz, 1H), 4.29 (dd, J = 9.8, 7.5 Hz, 1H), 3.96 (dd, J = 10.0, 4.4 Hz, 1H), 3.20 – 3.12 (m, 1H), 2.83 – 2.78 (m, 1H), 2.74 – 2.68 (m, 1H), 2.52 (dd, J = 13.7, 5.1 Hz, 1H). ^{13}C NMR (100 MHz, Chloroform-*d*) δ 198.2, 178.9, 137.2, 136.1, 136.0, 133.6, 129.7, 129.3, 128.9, 128.8, 128.8, 128.7, 128.2, 126.9, 71.4, 57.3, 44.8, 41.8, 35.9. HRMS (EI) m/z calcd. for $C_{25}H_{22}O_3$ $[M]^+$: 370.1569, found : 370.1572

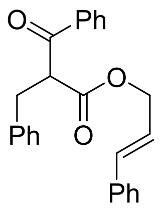

cinnamyl 2-benzyl-3-oxo-3-phenylpropanoate (8). Prepared according to **GP1.** Purified by flash chromatography on silica gel (EtOAc/hexanes = 1:8). From 1-(1-(cinnamyloxy)-1-oxo-3-phenylpropan-2-yl)-2,4,6-triphenylpyridin-1-ium tetrafluoroborate (65.9 mg, 0.1 mmol), compound **8** (6.3 mg, 17%) was obtained. Colorless oil. ^{1}H NMR (600 MHz, Chloroform-*d*) δ 8.05 – 7.94 (m, 2H),

7.60 – 7.52 (m, 1H), 7.49 – 7.39 (m, 2H), 7.33 – 7.22 (m, 8H), 7.20 – 7.14 (m, 1H), 6.49 (d, J = 15.9 Hz, 1H), 6.16 – 5.99 (m, 1H), 4.74 – 4.56 (m, 3H), 3.57 – 3.24 (m, 2H). ^{13}C NMR (150 MHz, Chloroform-*d*) δ 194.5, 169.2, 138.5, 136.3, 136.2, 134.6, 133.7, 129.1, 128.9, 128.8, 128.7, 128.7, 128.3, 126.8, 126.8, 122.5, 66.1, 56.3, 35.0. HRMS (EI) m/z calcd. for $C_{25}H_{22}O_3$ $[M]^+$: 370.1569, found : 370.1566

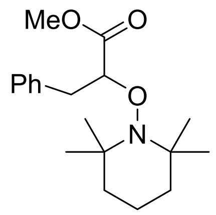

methyl 3-phenyl-2-((2,2,6,6-tetramethylpiperidin-1-yl)oxy)propanoate (9). Prepared according to **GP1** and with 2.0 equiv TEMPO. Purified by flash chromatography on silica gel (EtOAc/hexanes = 1:15). 1-(1-methoxy-1-oxo-3-phenylpropan-2-yl)-2,4,6-triphenylpyridin-1-ium tetrafluoroborate (83.6 mg, 0.15 mmol), compound **11** (33.5 mg, 70%) was obtained. Colorless oil. ^{1}H NMR (600 MHz, Chloroform-*d*) δ 7.25 (t, *J* = 7.4 Hz, 2H), 7.19 (t, *J* = 7.3 Hz, 1H), 7.15 (d, *J* = 7.4 Hz, 2H), 4.45 (dd, *J* = 10.2, 5.5 Hz, 1H), 3.49 (s, 3H), 3.24 (dd, *J* = 13.2, 5.5 Hz, 1H), 2.99 (dd, *J* = 13.2, 10.2 Hz, 1H), 1.59 – 1.35 (m, 5H), 1.34 – 1.27 (m, 1H), 1.23 (s, 3H), 1.13 (s, 6H), 1.01 (s, 3H). ^{13}C NMR (100 MHz, Chloroform-*d*) δ 173.1, 136.0, 129.3, 128.3, 126.6, 86.5, 60.5, 59.4, 51.1, 40.3, 40.2, 38.5, 33.5, 32.8, 20.2, 20.0, 17.0. HRMS (EI) m/z calcd. for $C_{19}H_{29}NO_3$ $[M]^+$: 319.2147, found : 319.2144

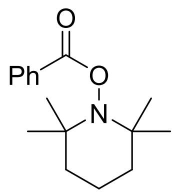

2,2,6,6-tetramethylpiperidin-1-yl benzoate (10). Prepared according to **GP1** and with 2.0 equiv TEMPO. Purified by flash chromatography on silica gel (EtOAc/hexanes = 1:15). 1-(1-methoxy-1-oxo-3-phenylpropan-2-yl)-2,4,6-triphenylpyridin-1-ium tetrafluoroborate (83.6 mg, 0.15 mmol), compound **12** (7.1 mg, 18%) was obtained. Colorless oil. ^{1}H NMR (600 MHz, Chloroform-*d*) δ 8.08 (dd, *J* = 8.3, 1.4 Hz, 2H), 7.62 – 7.53 (m, 1H), 7.49 – 7.42 (m, 2H), 1.78 (tt, *J* = 12.8, 2.5 Hz, 2H), 1.75 – 1.67 (m, 1H), 1.59 (dt, *J* = 12.8, 2.9 Hz, 2H), 1.50 – 1.43 (m, 1H), 1.28 (s, 6H), 1.12 (s, 6H). ^{13}C NMR (100 MHz, Chloroform-*d*) δ 166.4, 132.8, 129.7, 129.6, 128.4, 60.4, 39.0, 32.0, 20.8, 17.0. HRMS (EI) m/z calcd. for $C_{16}H_{23}NO_2$ $[M]^+$: 261.1729, found : 261.1731

Appendix I

Spectral Copies of ^{1}H, ^{13}C and ^{19}F NMR Data Obtained in this Study

1-(1-methoxy-1-oxopent-4-en-2-yl)-2,4,6-triphenylpyridin-1-ium tetrafluoroborate (1k).

600 MHz, ^{1}H NMR in Chloroform-*d*

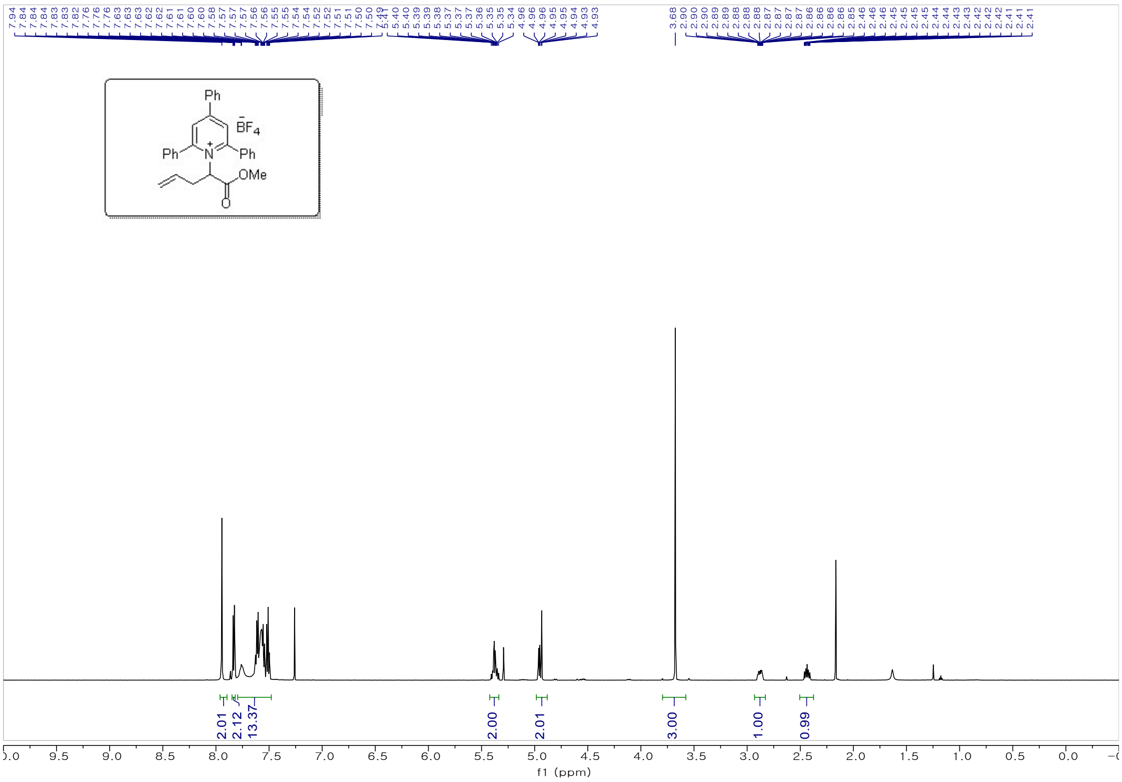

150 MHz, ^{13}C NMR in Chloroform-*d*

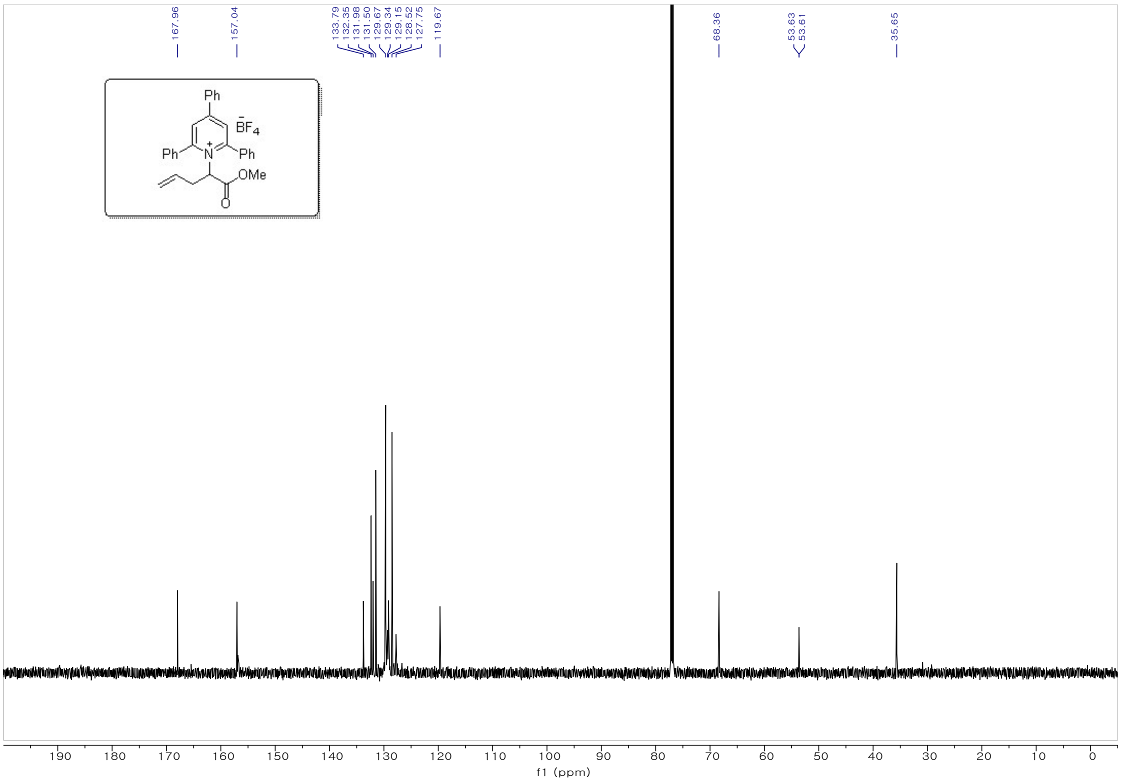

375MHz, ^{19}F NMR in Chloroform-*d*

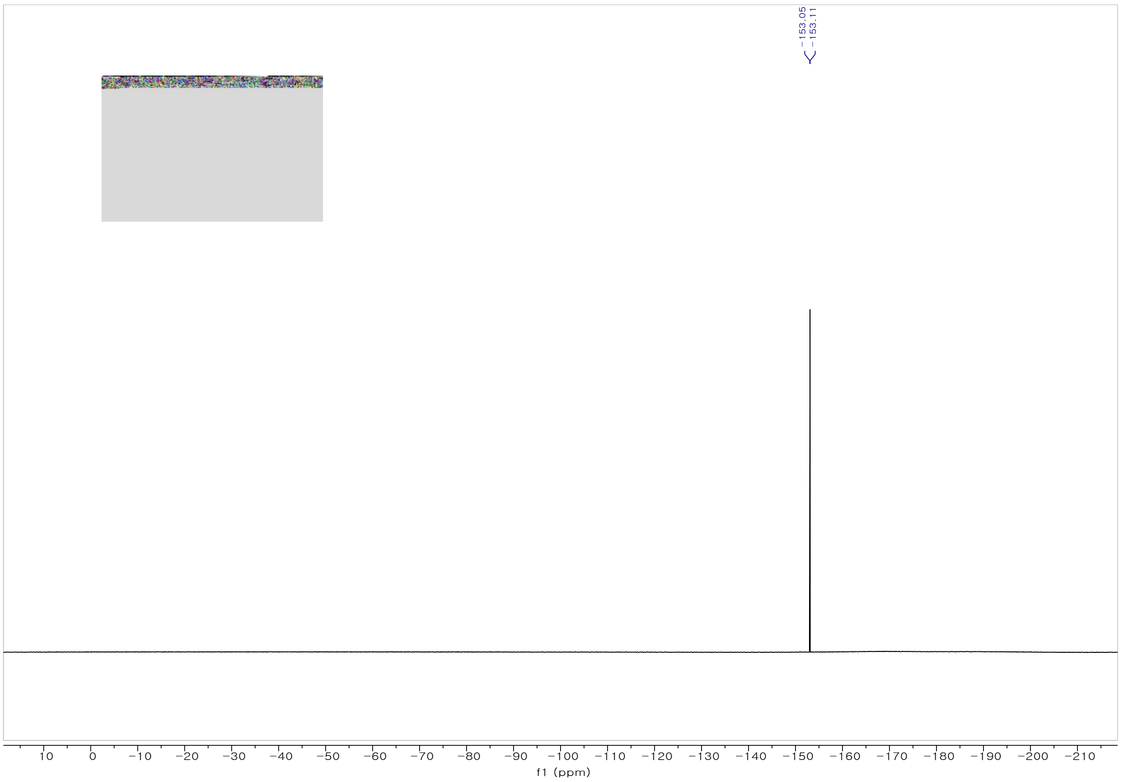

1-(4-methyl-1-(naphthalen-2-ylamino)-1-oxopentan-2-yl)-2,4,6-triphenylpyridin-1-ium tetrafluoroborate (1m).

400 MHz, ^{1}H NMR in Chloroform-d

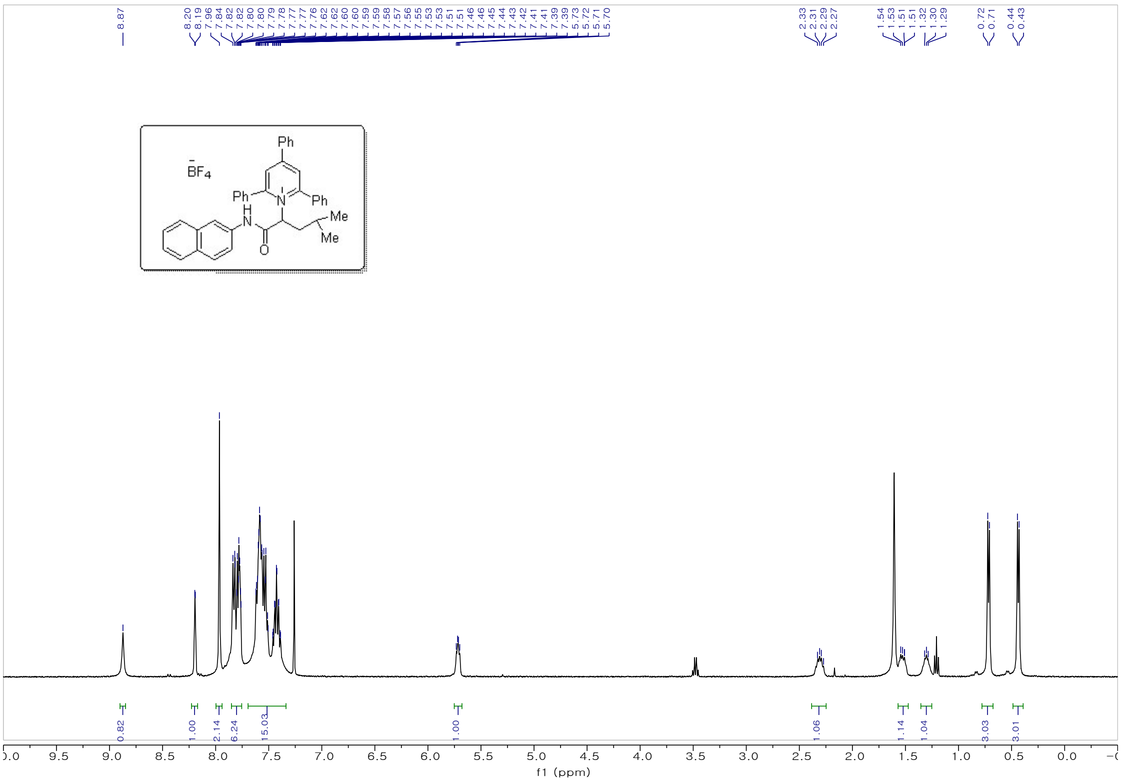

100 MHz, ^{13}C NMR in Chloroform-d

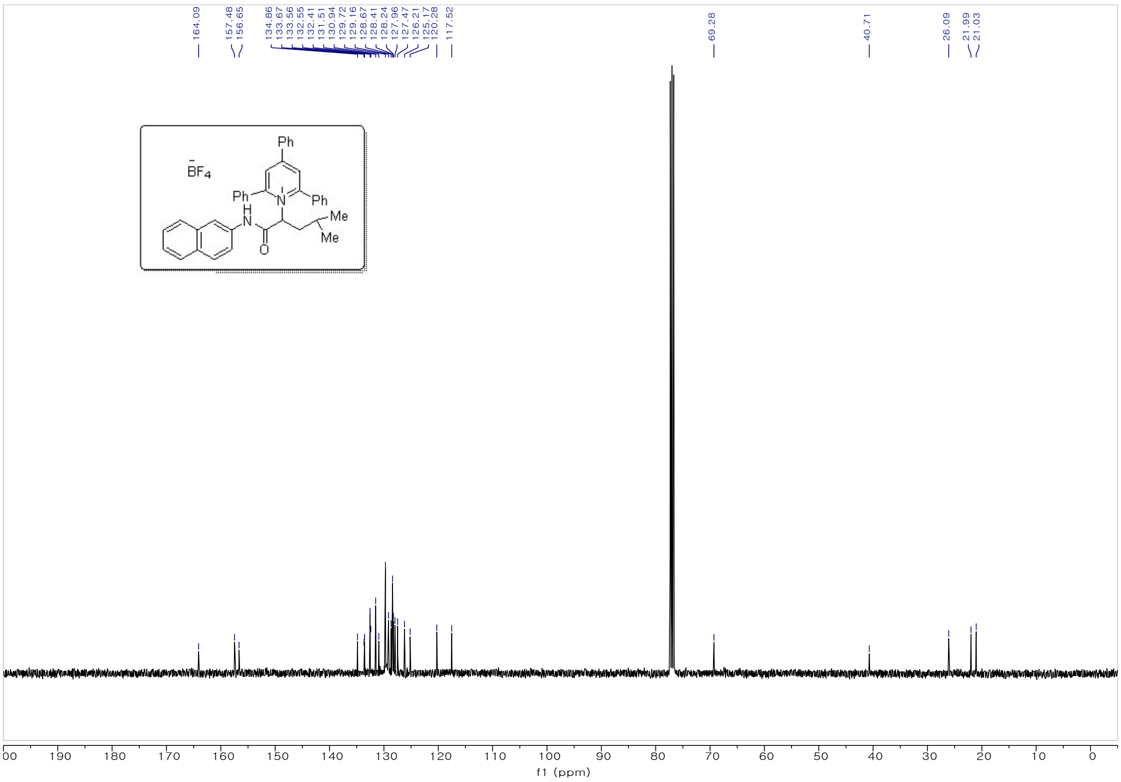

375MHz, ^{19}F NMR in Chloroform-d

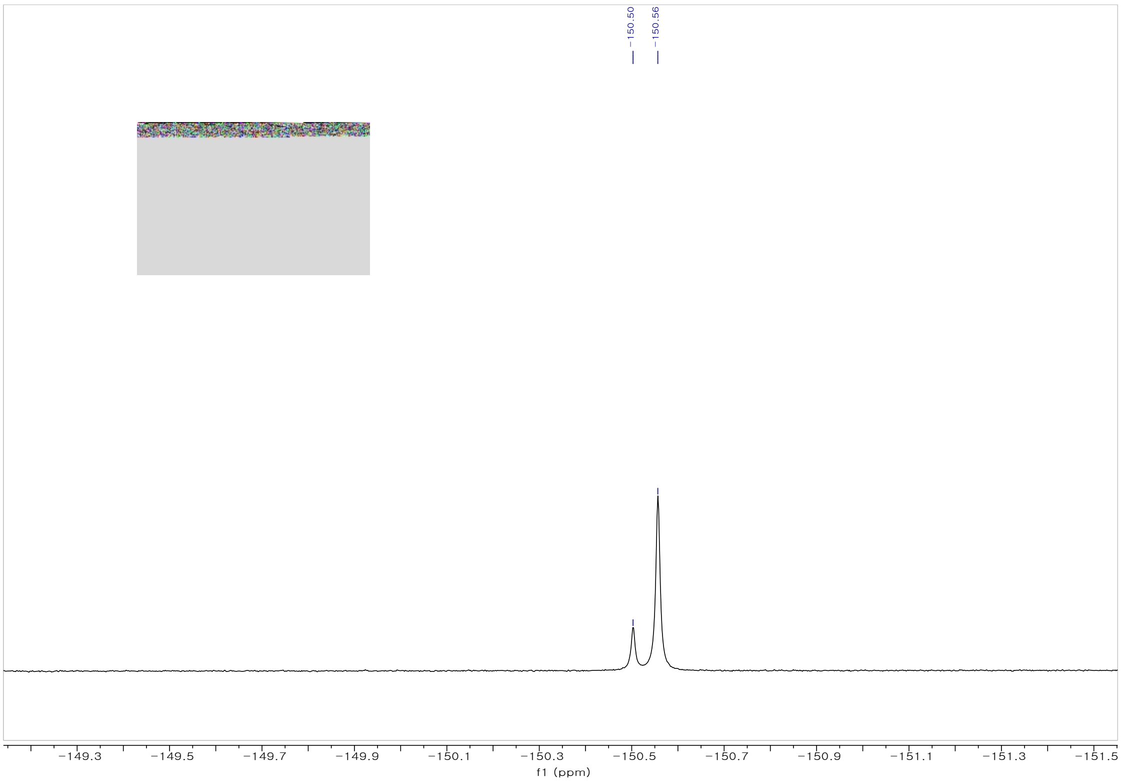

1-((5R,6R)-6-acetamido-3-(ethoxycarbonyl)-5-(pentan-3-yloxy)cyclohex-3-en-1-yl)-2,4,6-triphenylpyridin-1-ium tetrafluoroborate (1t).

600 MHz, ^{1}H NMR in Chloroform-*d*

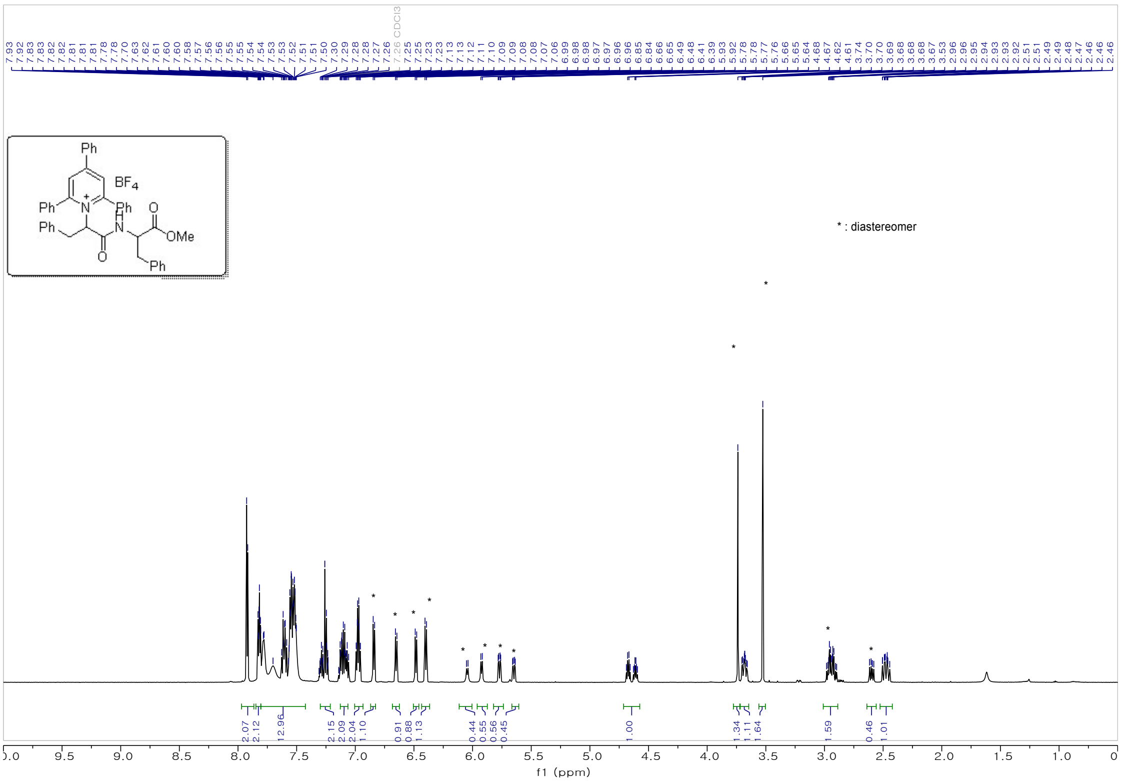

150 MHz, ^{13}C NMR in Chloroform-*d*

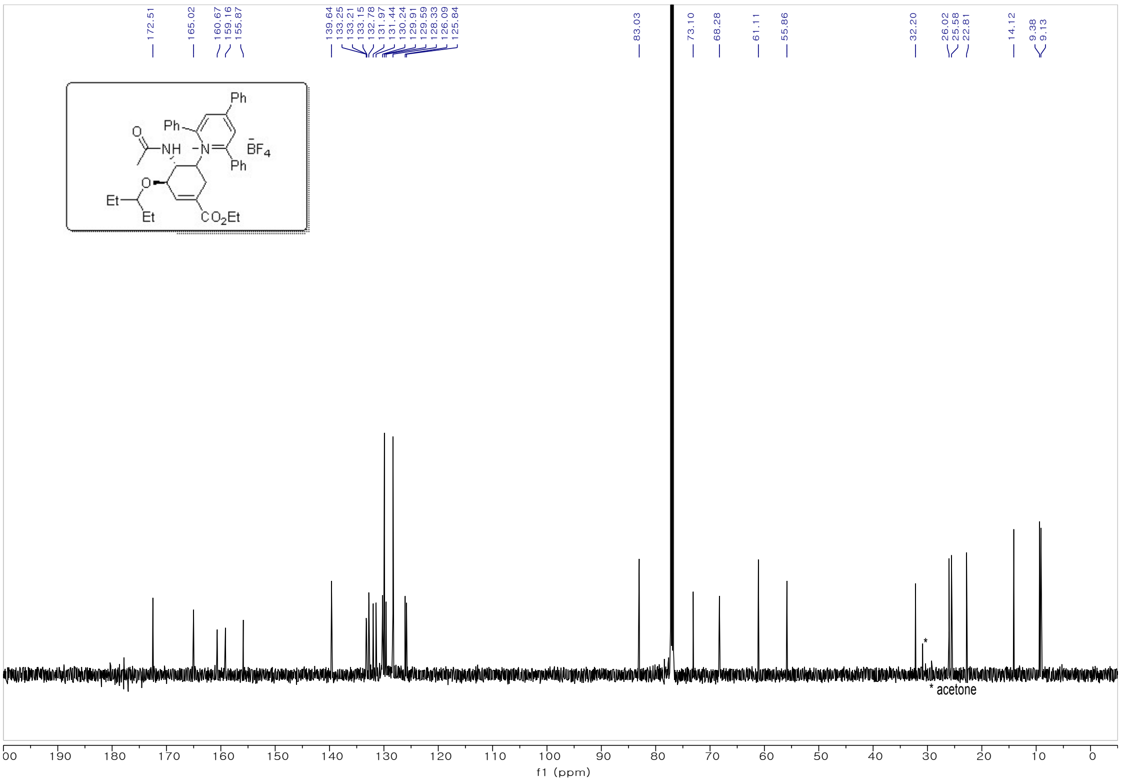

375MHz, ^{19}F NMR in Chloroform-*d*

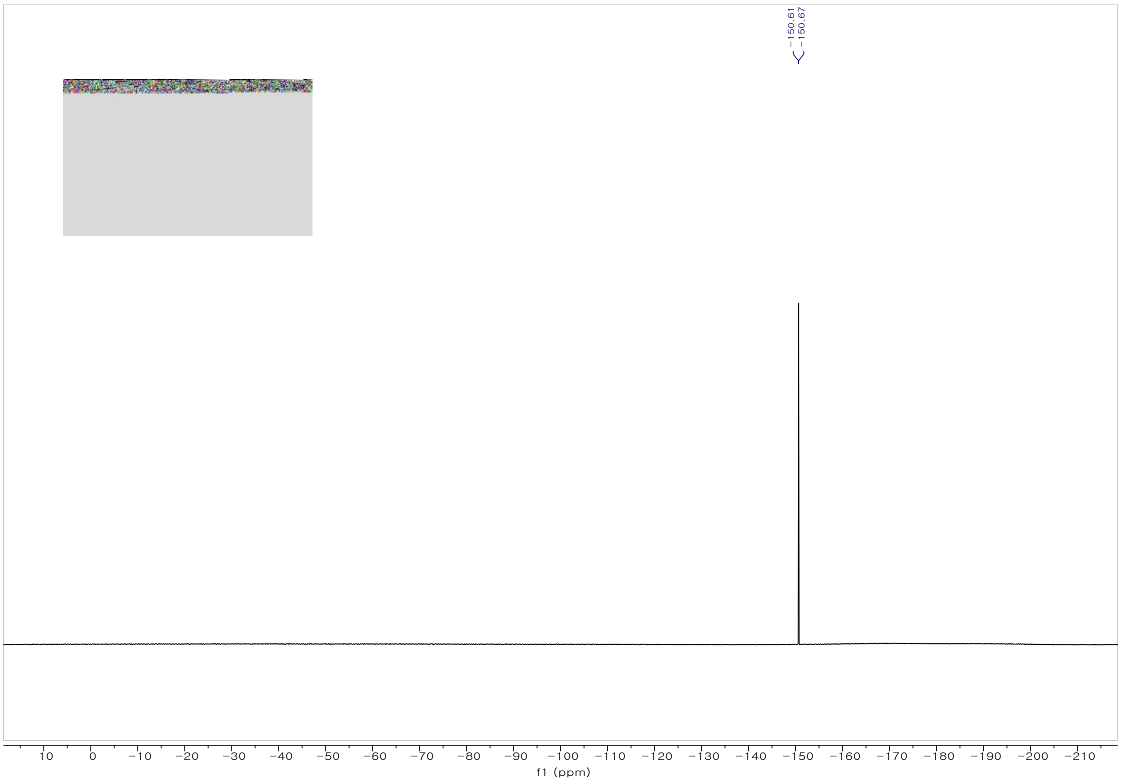

1-(1-((1-methoxy-1-oxo-3-phenylpropan-2-yl)amino)-1-oxo-3-phenylpropan-2-yl)-2,4,6-triphenylpyridin-1-ium tetrafluoroborate (1v).

600 MHz, ^{1}H NMR in Chloroform-*d*

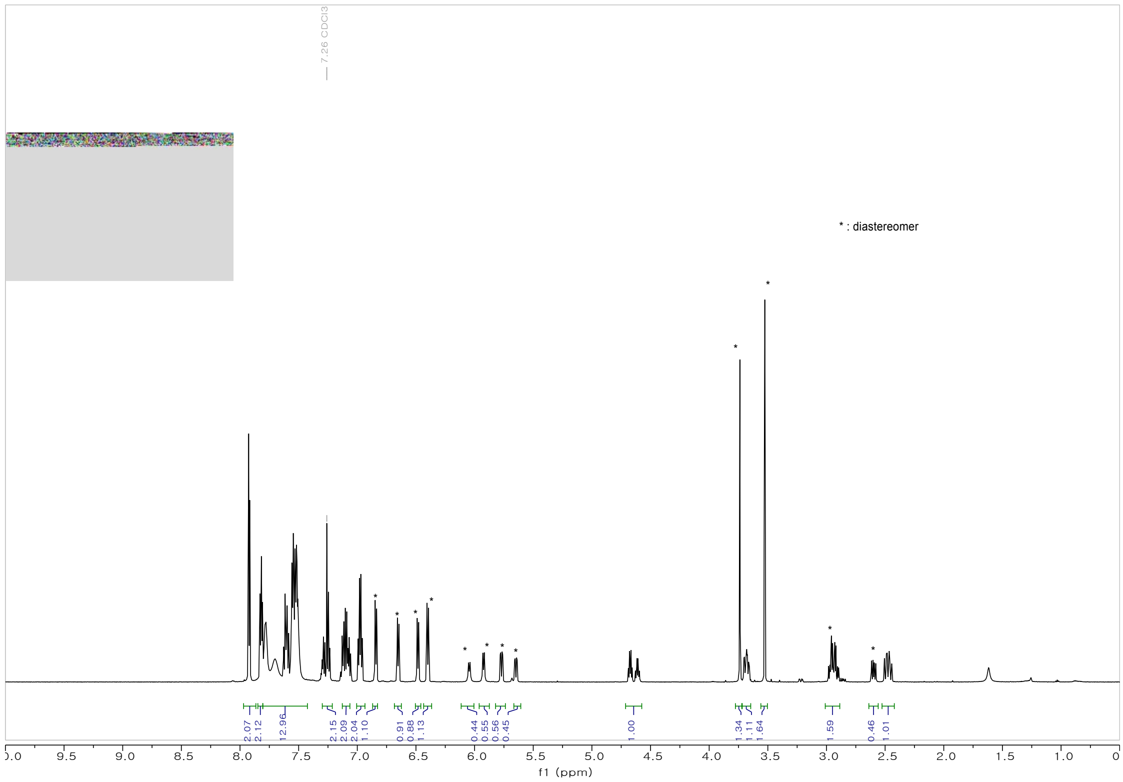

150 MHz, ^{13}C NMR in Chloroform-*d*

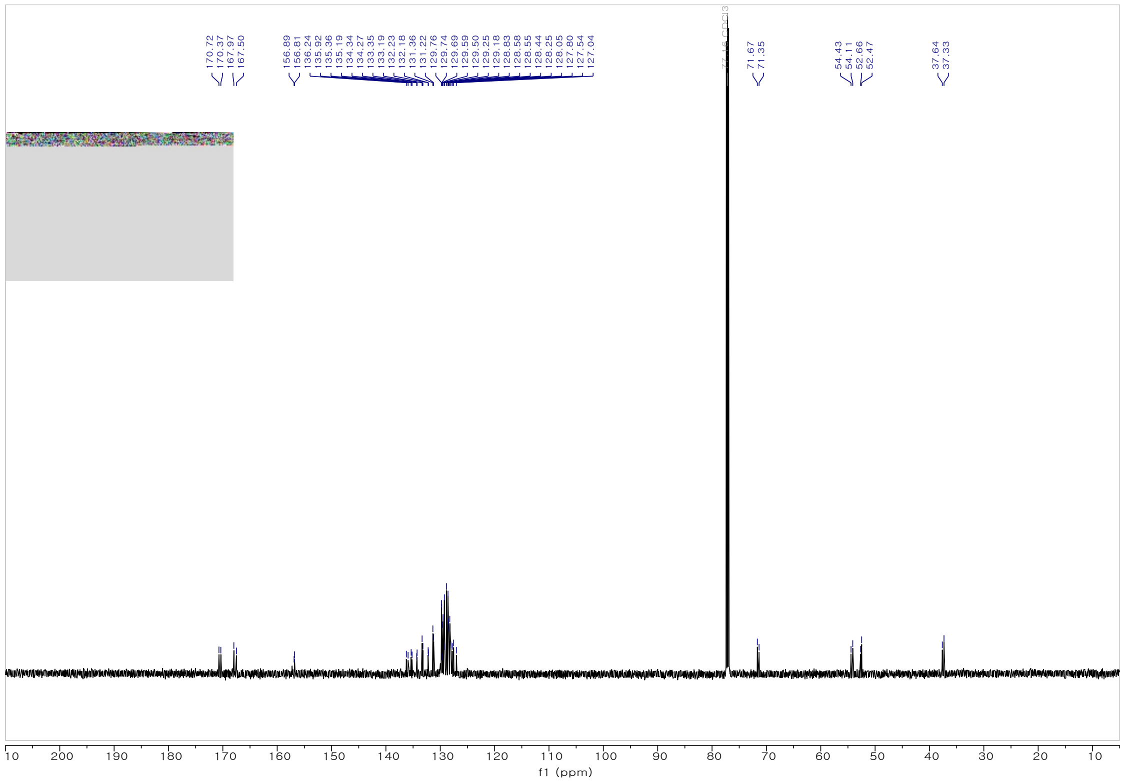

375 MHz, ^{19}F NMR in Chloroform-*d*

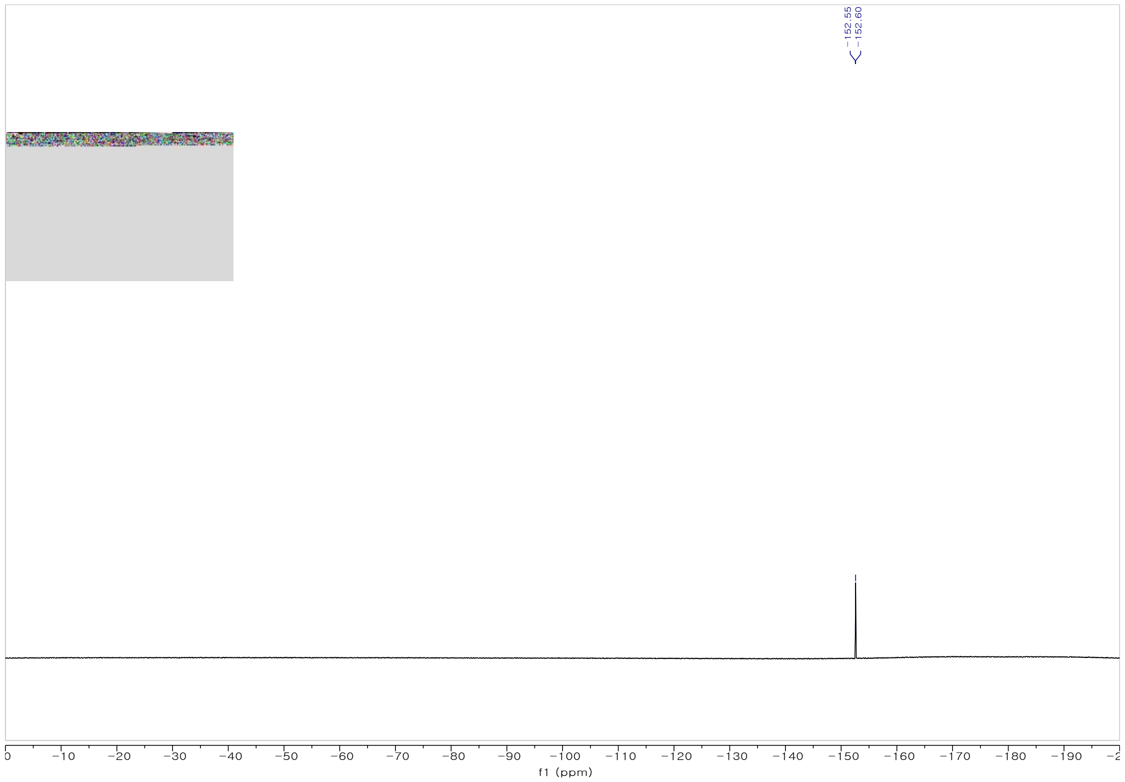

1-(1-((2-methoxy-2-oxoethyl)amino)-4-(methylthio)-1-oxobutan-2-yl)-2,4,6-triphenylpyridin-1-ium tetrafluoroborate (1w).

600 MHz, ^{1}H NMR in Chloroform-*d*

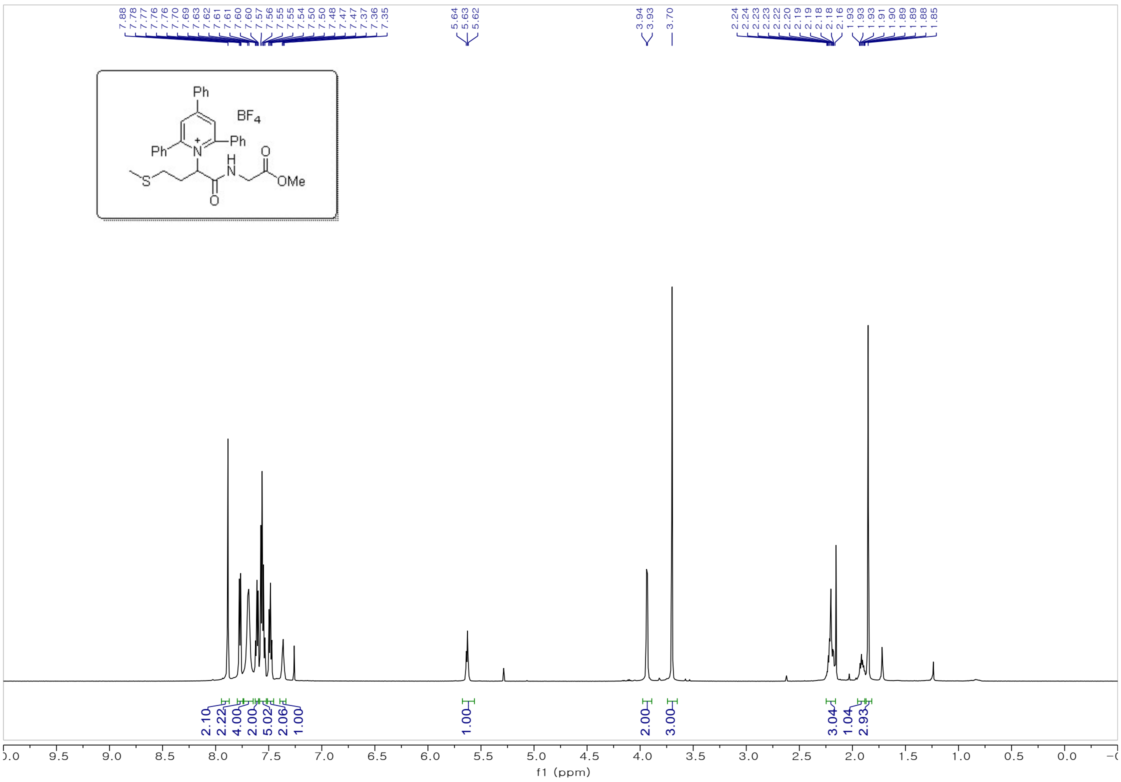

150 MHz, ^{13}C NMR in Chloroform-*d*

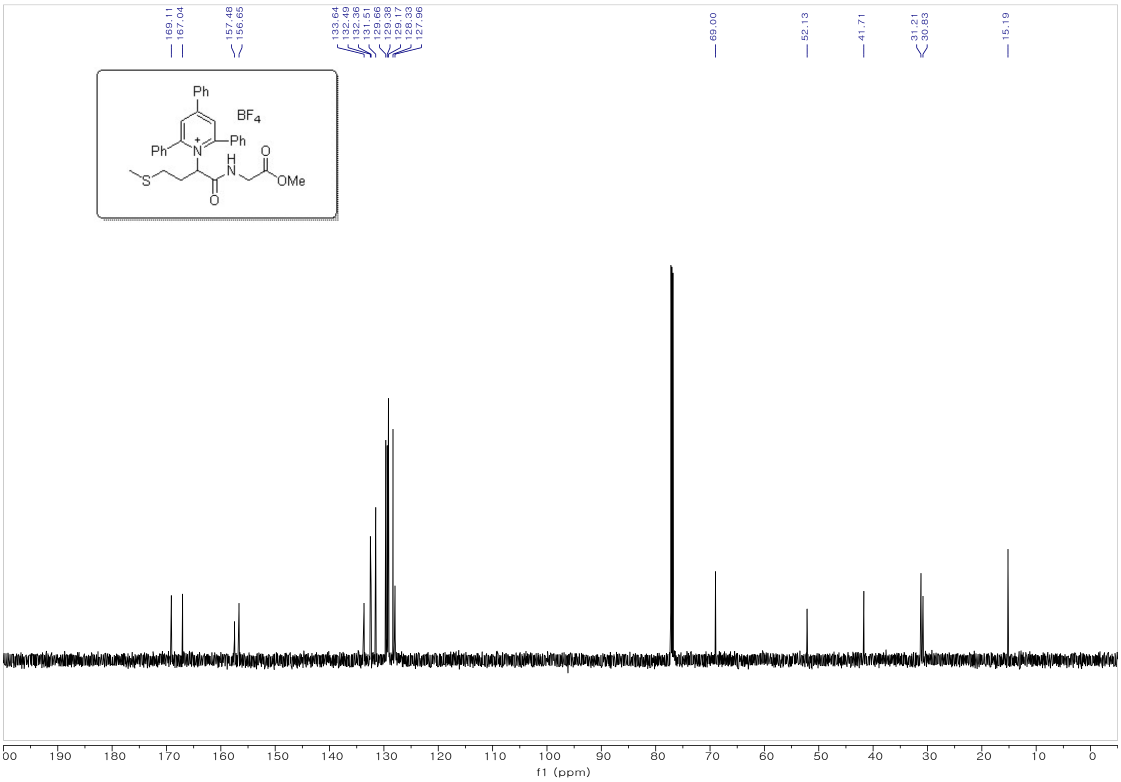

375MHz, ^{19}F NMR in Chloroform-*d*

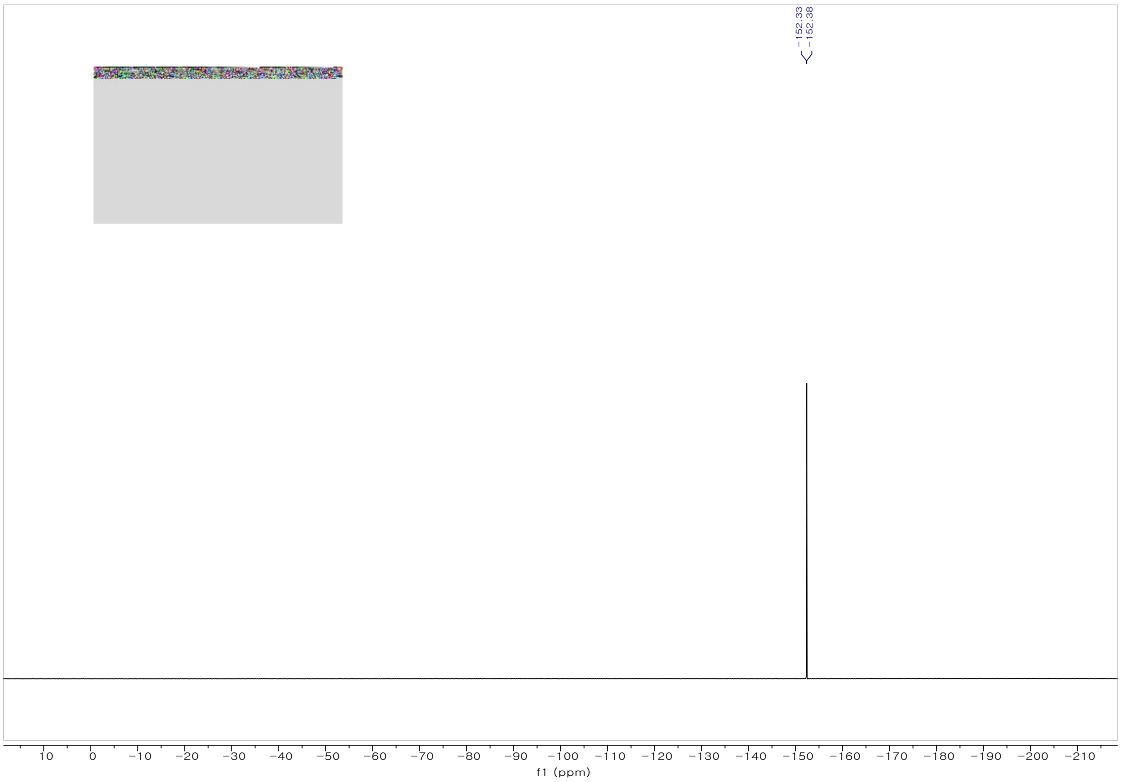

1-(1-((2-methoxy-2-oxoethyl)amino)-4-methyl-1-oxopentan-2-yl)-2,4,6-triphenylpyridin-1-ium tetrafluoroborate (1x).

400 MHz, ^{1}H NMR in Chloroform-*d*

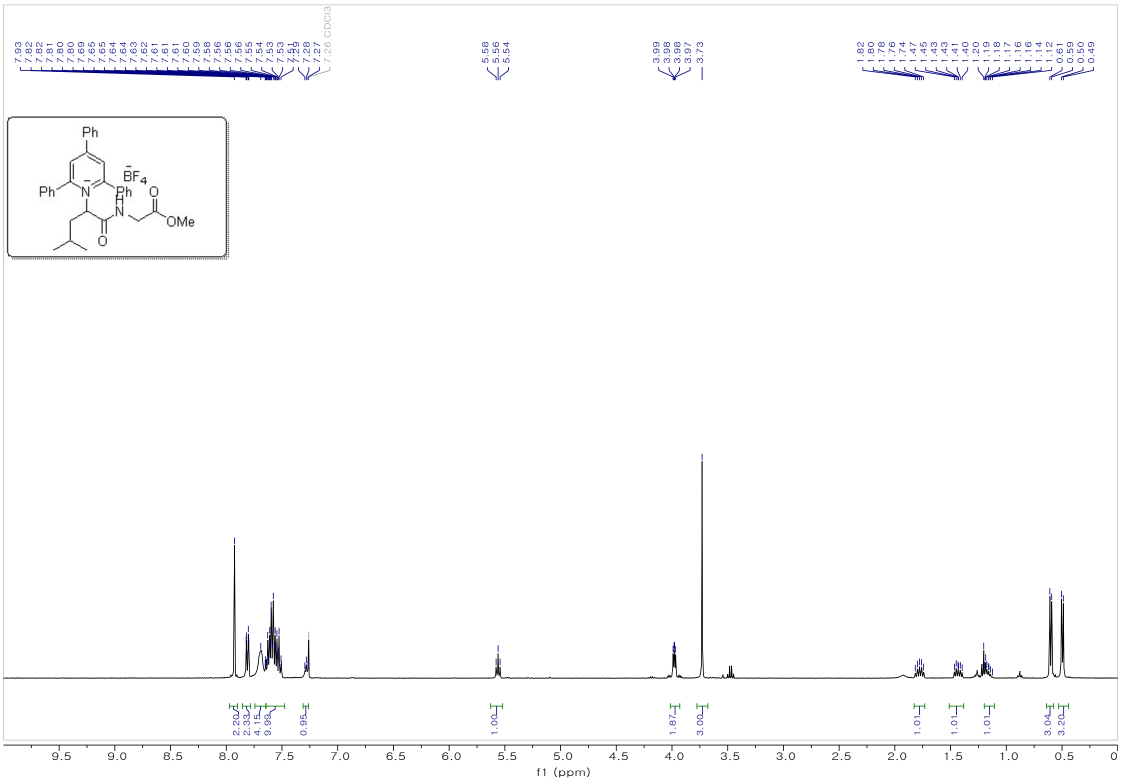

100 MHz, ^{13}C NMR in Chloroform-*d*

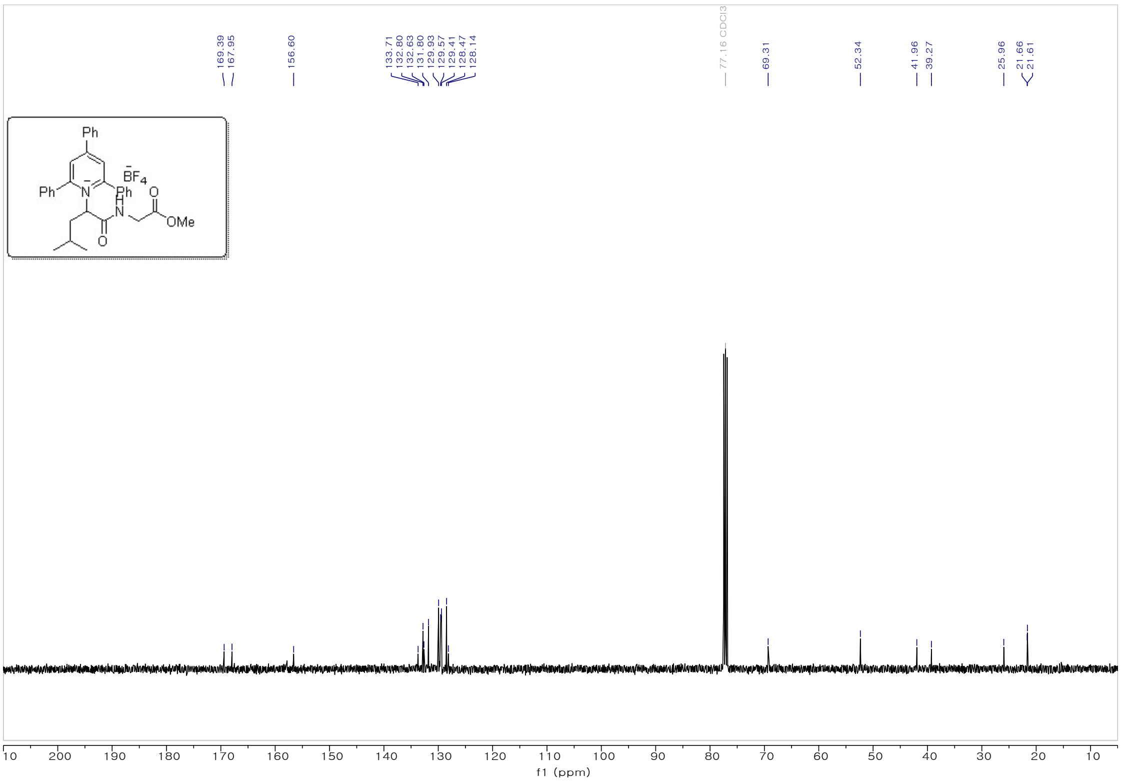

375 MHz, ^{19}F NMR in Chloroform-*d*

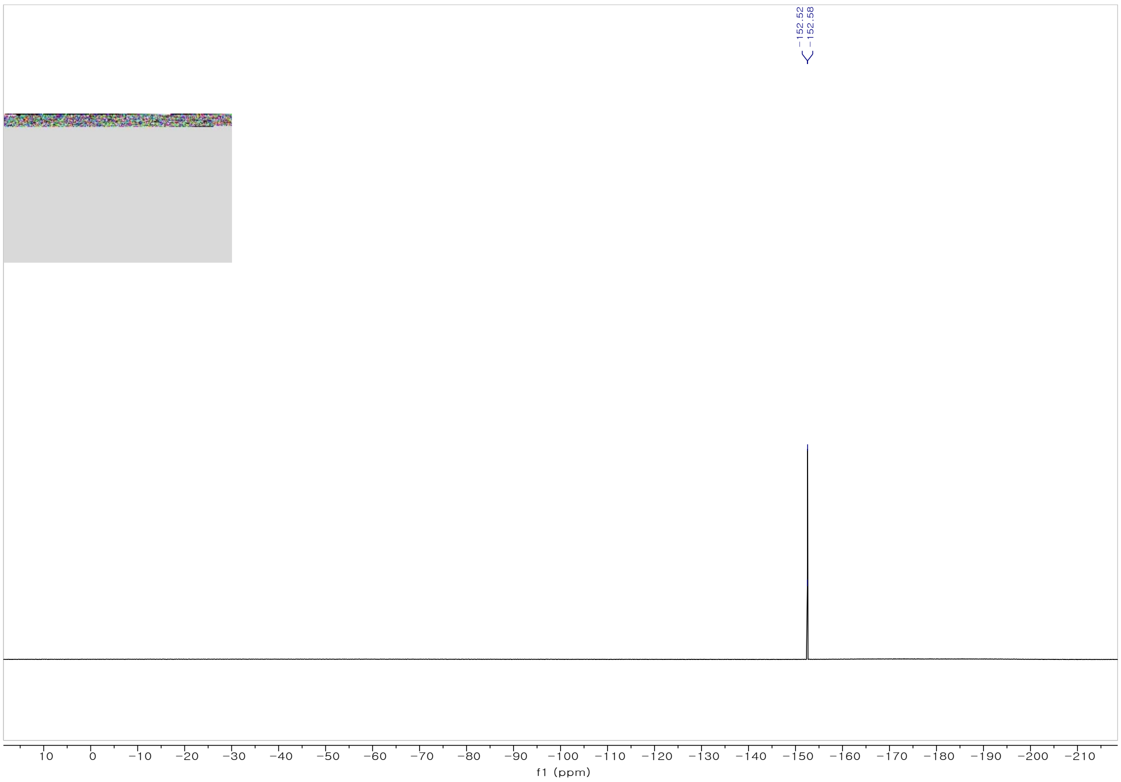

1-(1-((1-methoxy-1-oxo-3-phenylpropan-2-yl)amino)-1-oxopropan-2-yl)-2,4,6-triphenylpyridin-1-ium tetrafluoroborate (1y).

600 MHz, ^{1}H NMR in Chloroform-*d*

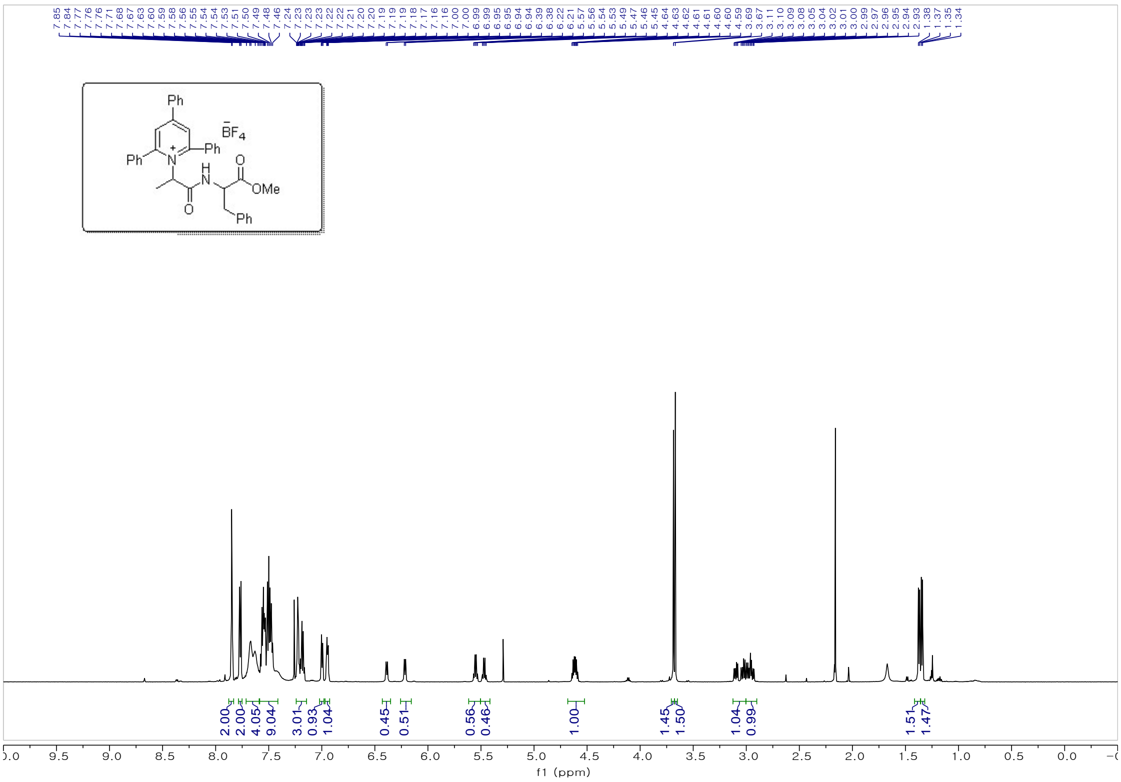

150 MHz, ^{13}C NMR in Chloroform-*d*

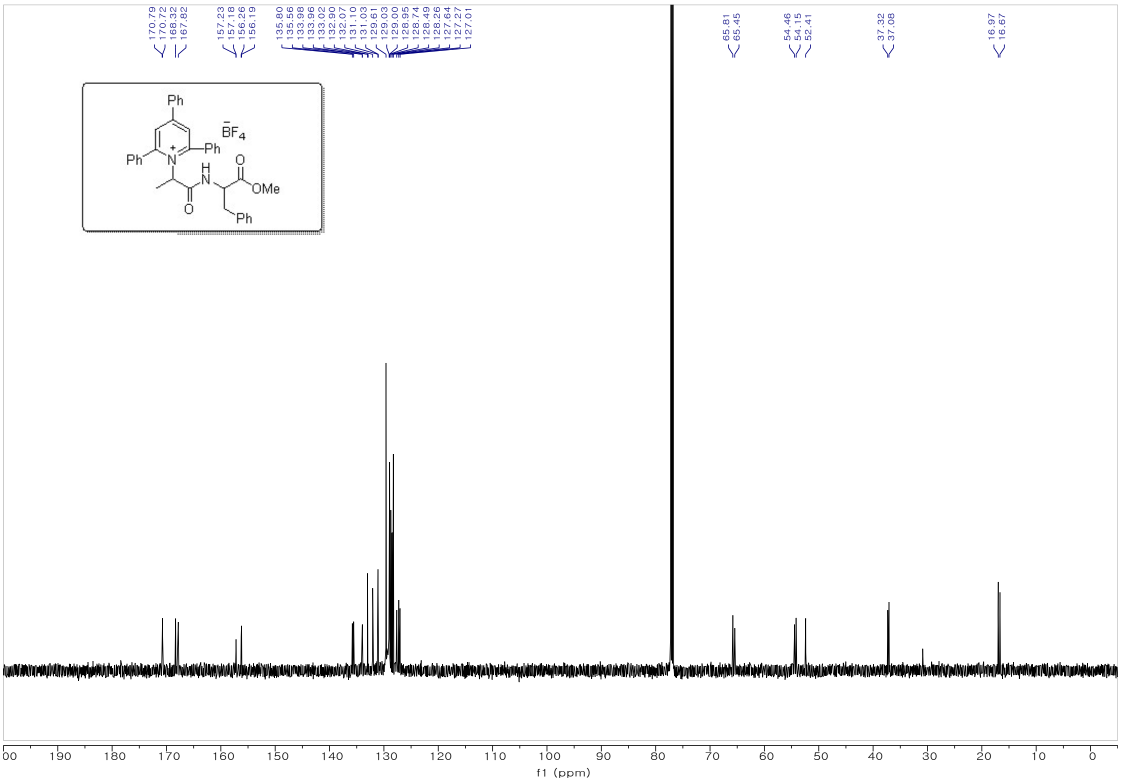

375MHz, ^{19}F NMR in Chloroform-*d*

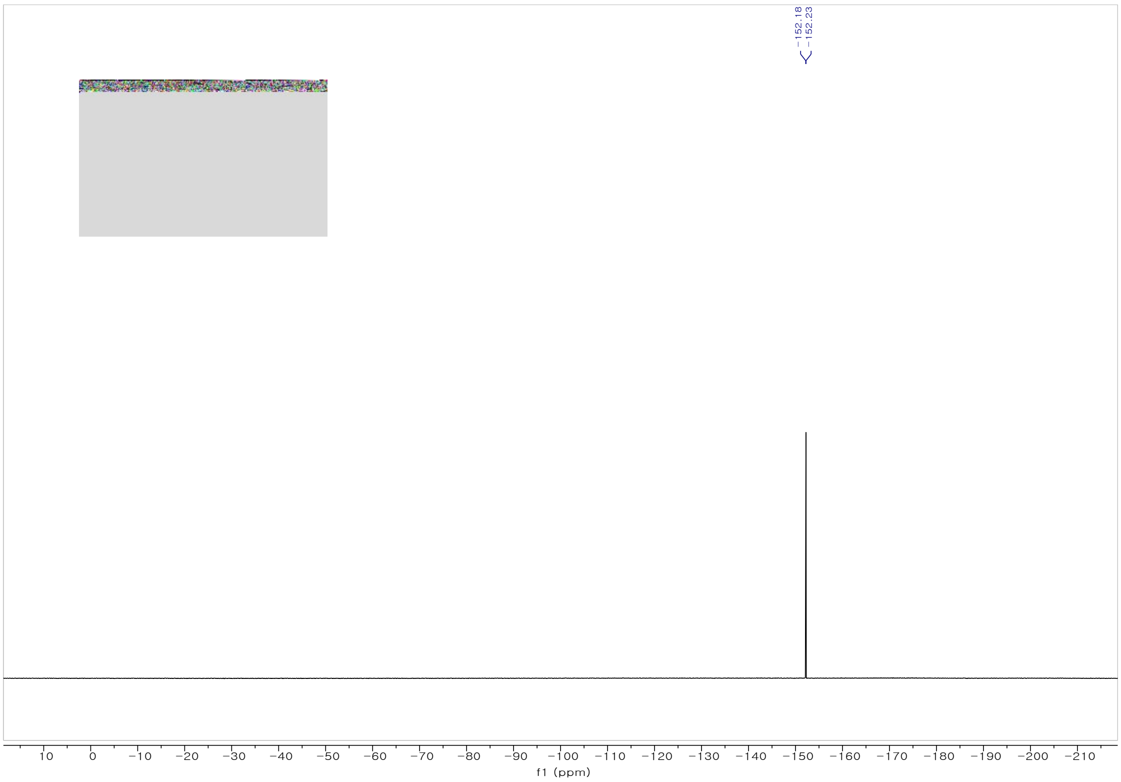

1-(1-((2-((2-methoxy-2-oxoethyl)amino)-2-oxoethyl)amino)-4-methyl-1-oxopentan-2-yl)-2,4,6-triphenylpyridin-1-ium tetrafluoroborate (1z).

600 MHz, ^{1}H NMR in Chloroform-*d*

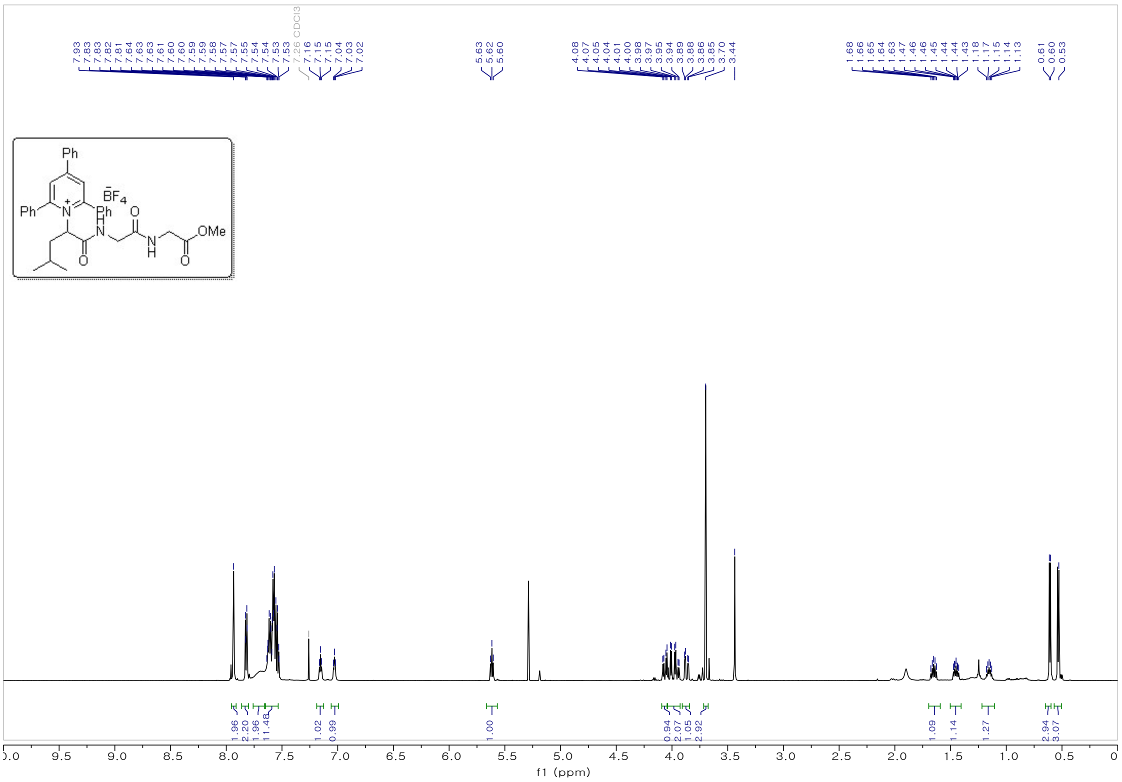

150 MHz, ^{13}C NMR in Chloroform-*d*

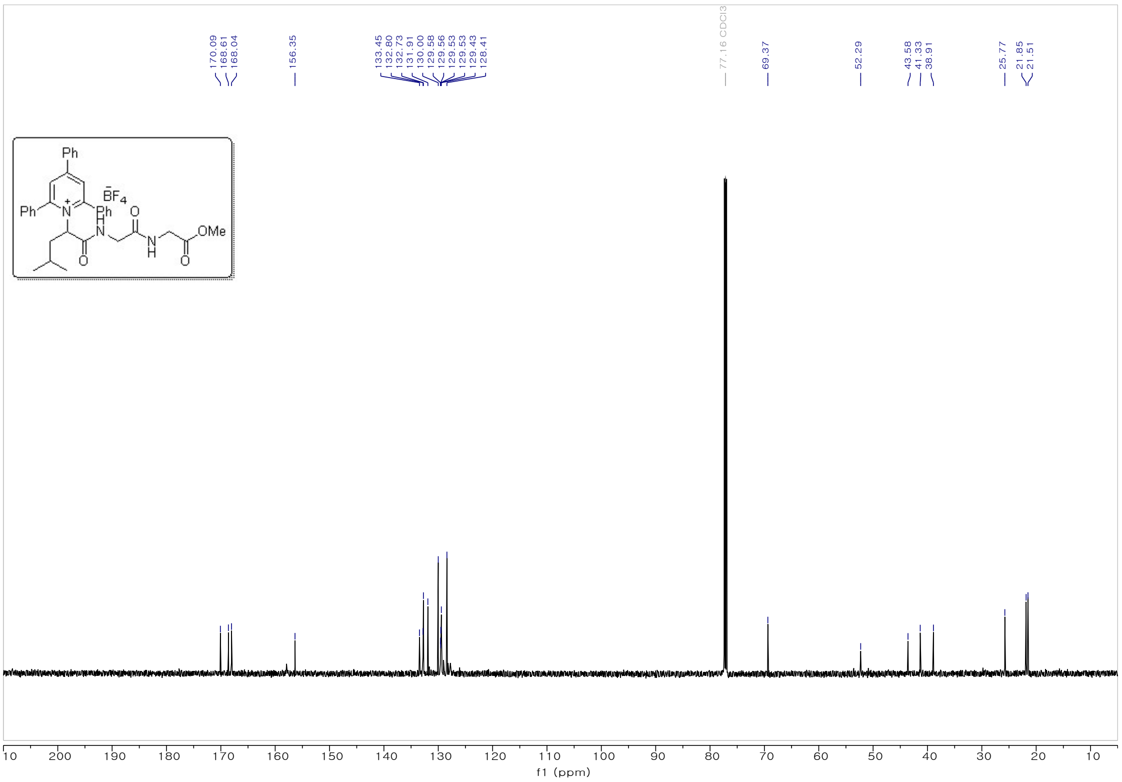

375MHz, ^{19}F NMR in Chloroform-*d*

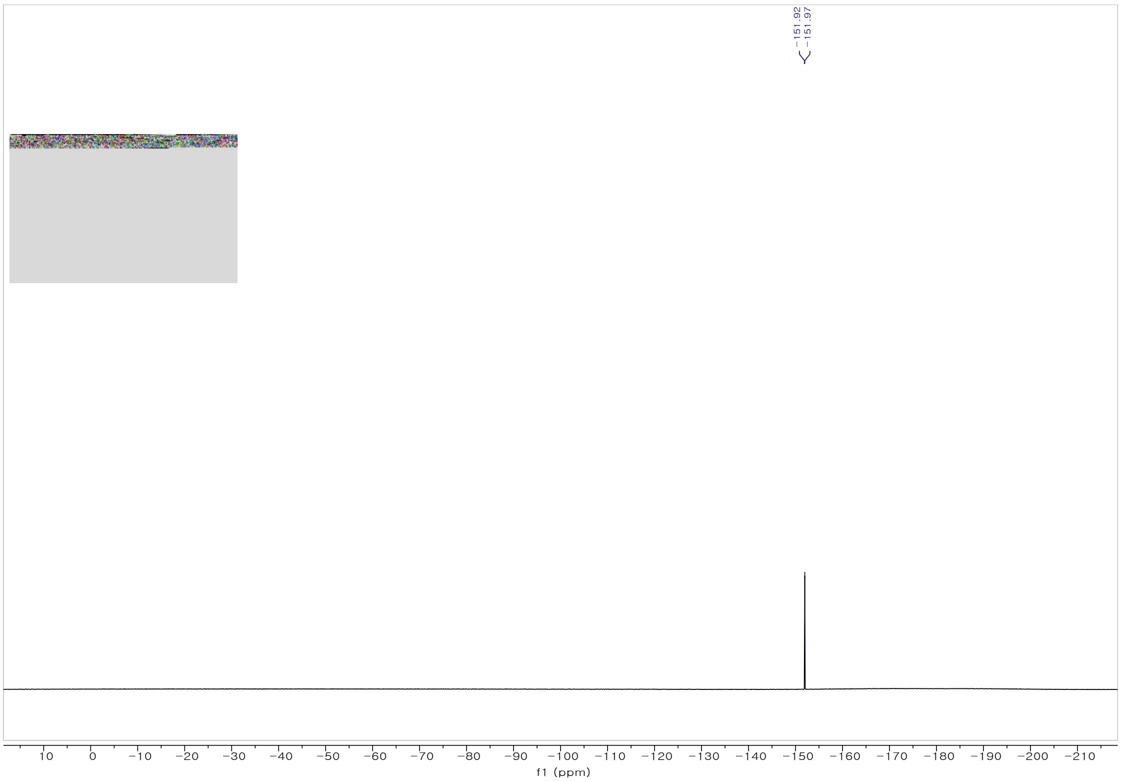

1-(1-((2-((2-methoxy-2-oxoethyl)amino)-2-oxoethyl)amino)-1-oxo-3-phenylpropan-2-yl)-2,4,6-triphenylpyridin-1-ium tetrafluoroborate (1aa).

600 MHz, ^{1}H NMR in Chloroform-*d*

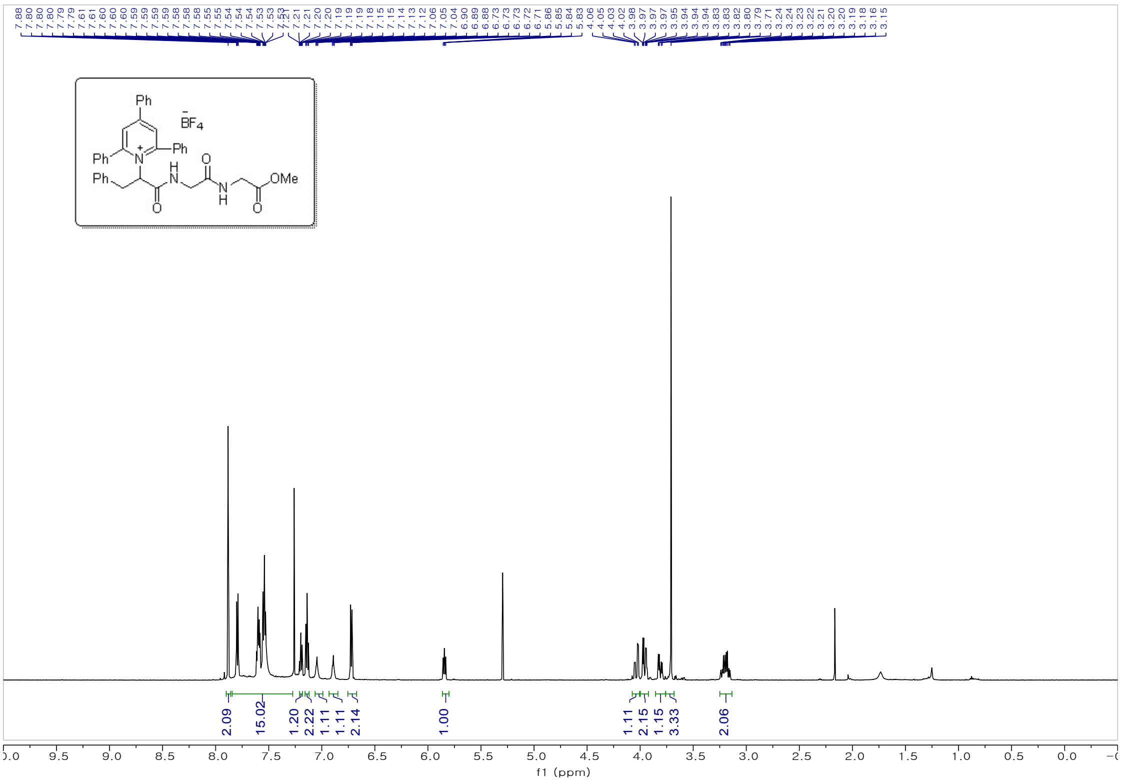

150 MHz, ^{13}C NMR in Chloroform-*d*

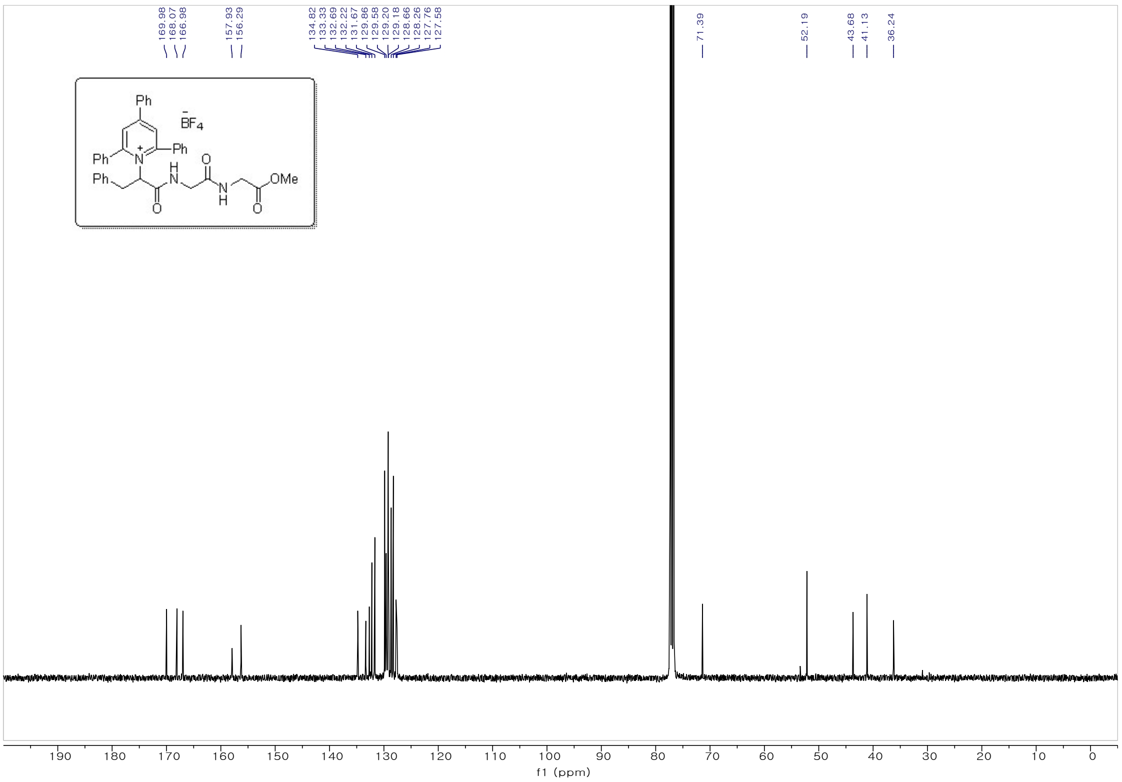

375MHz, ^{19}F NMR in Chloroform-*d*

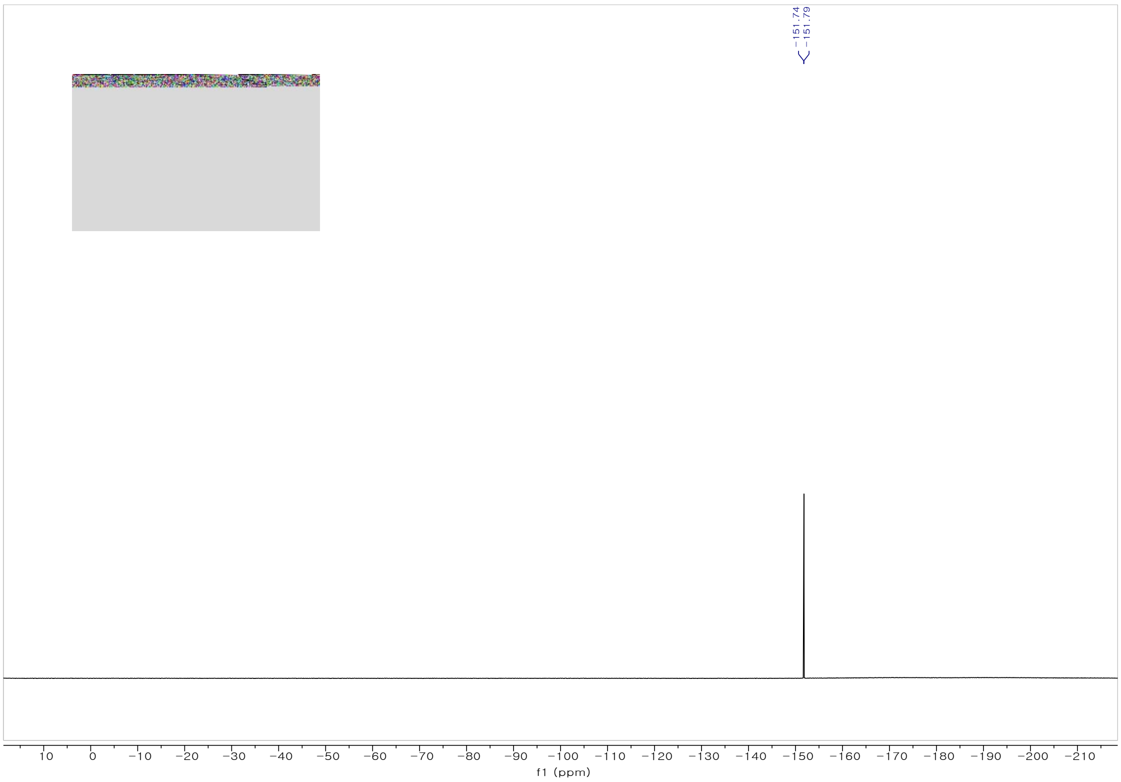

1-(7-isobutyl-10-methyl-3,6,9,12-tetraoxo-14-phenyl-2-oxa-5,8,11-triazatetradecan-13-yl)-2,4,6-triphenylpyridin-1-ium tetrafluoroborate (1ab).

600 MHz, ^{1}H NMR in Chloroform-*d*

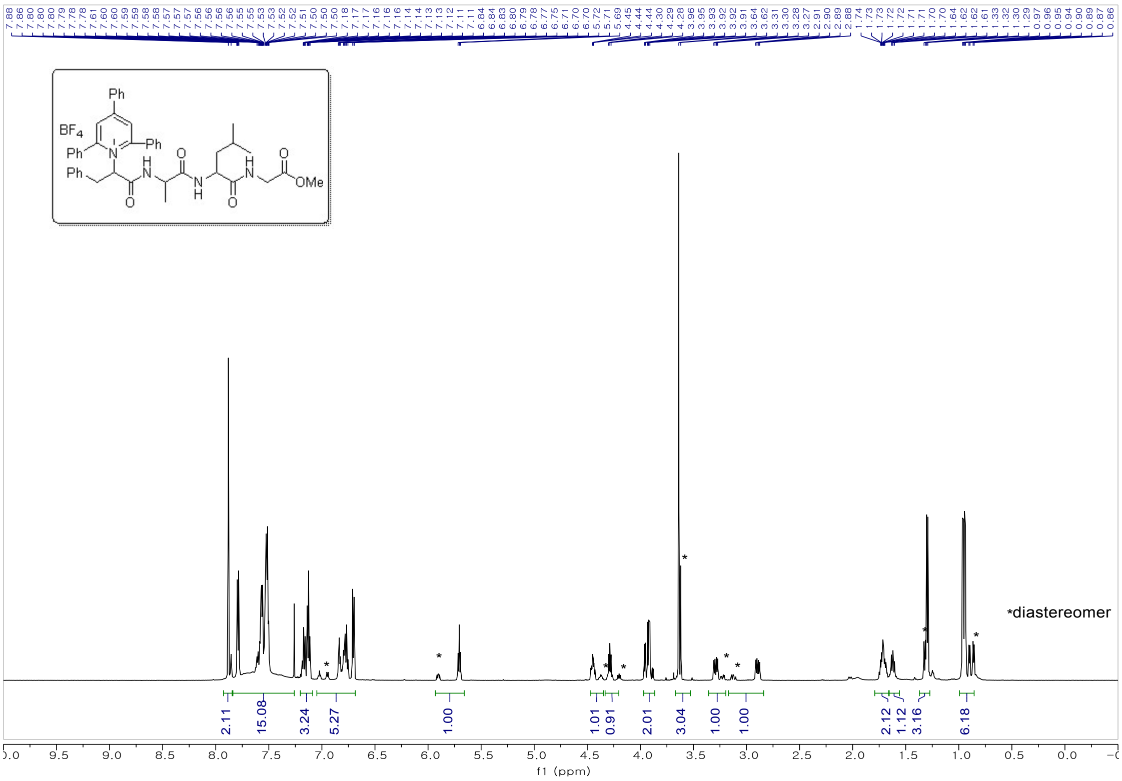

150 MHz, ^{13}C NMR in Chloroform-*d*

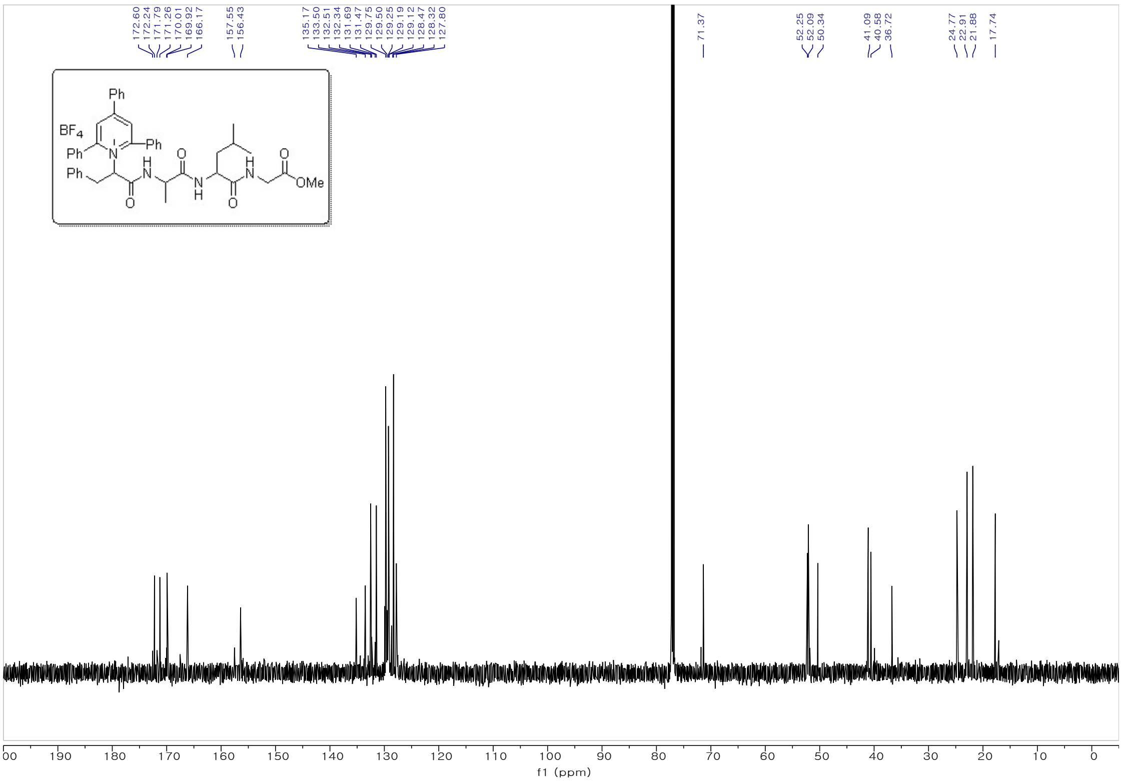

375MHz, ^{19}F NMR in Chloroform-*d*

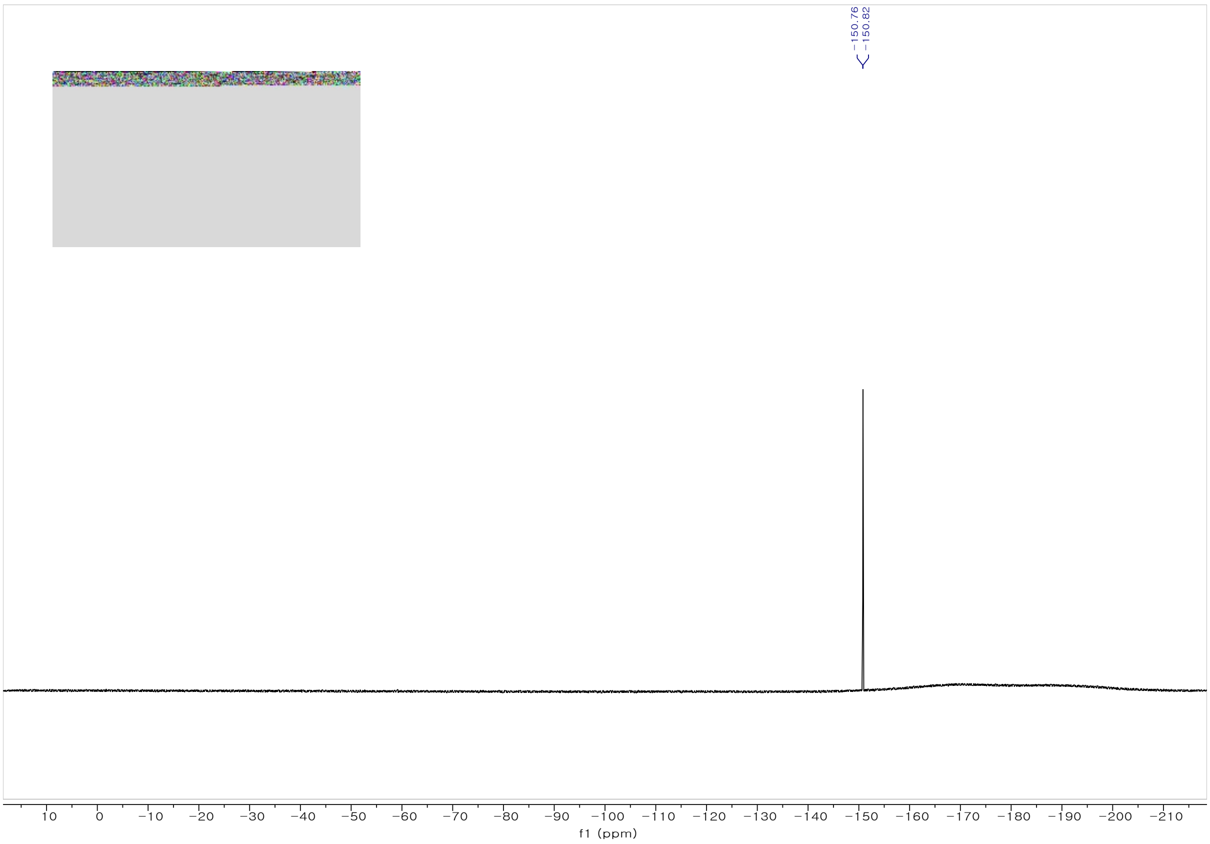

1-(7-benzyl-3,6,9,12,16-pentaoxo-2,17-dioxa-5,8,11-triazaoctadecan-13-yl)-2,4,6-triphenylpyridin-1-ium tetrafluoroborate (1ac).

400 MHz, ^{1}H NMR in Chloroform-*d*

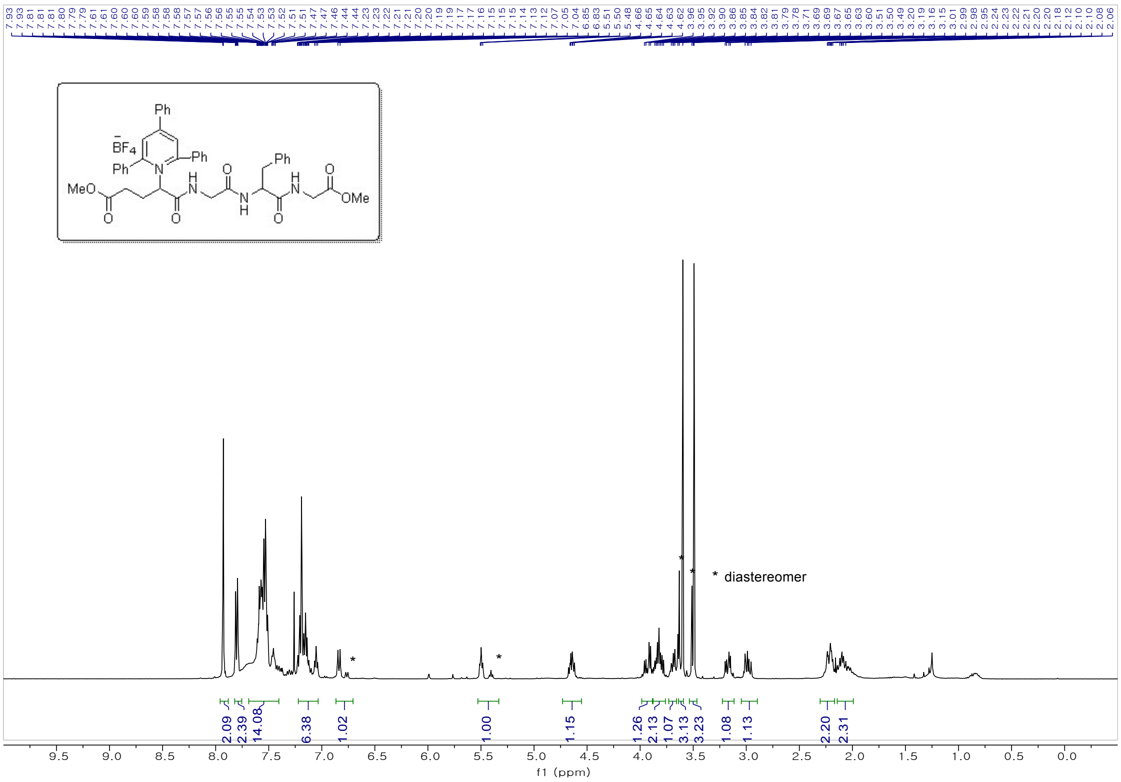

100 MHz, ^{13}C NMR in Chloroform-*d*

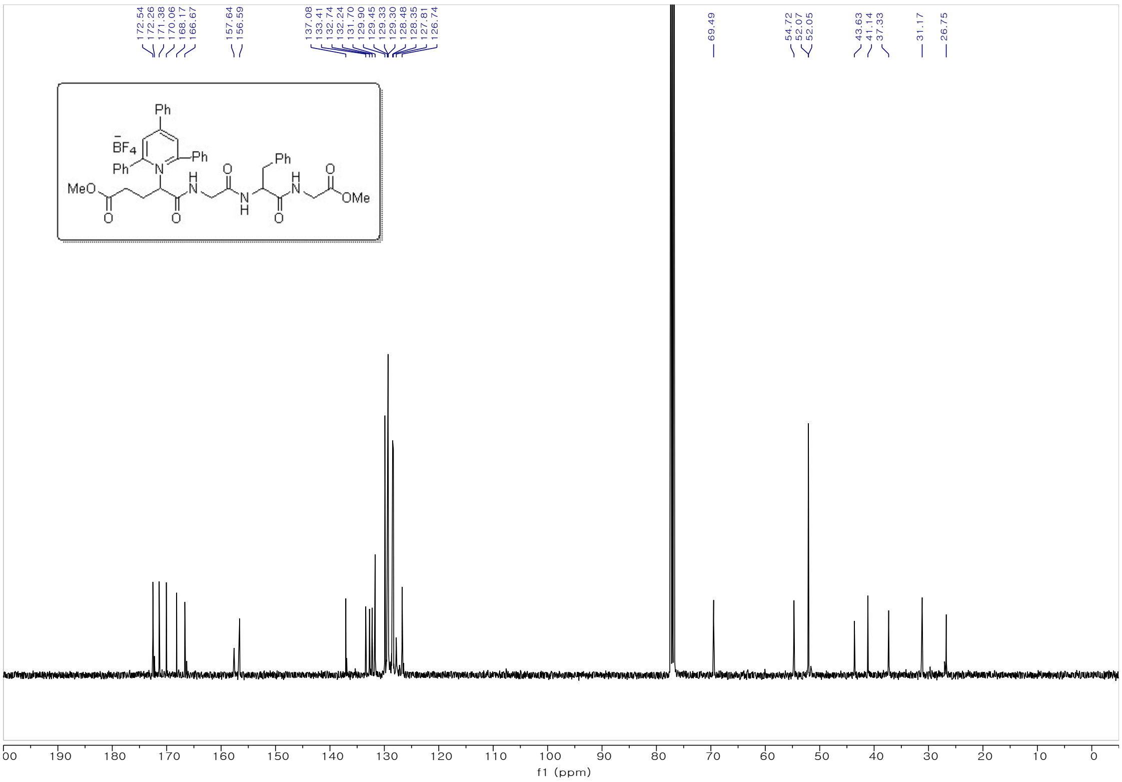

375MHz, ^{19}F NMR in Chloroform-*d*

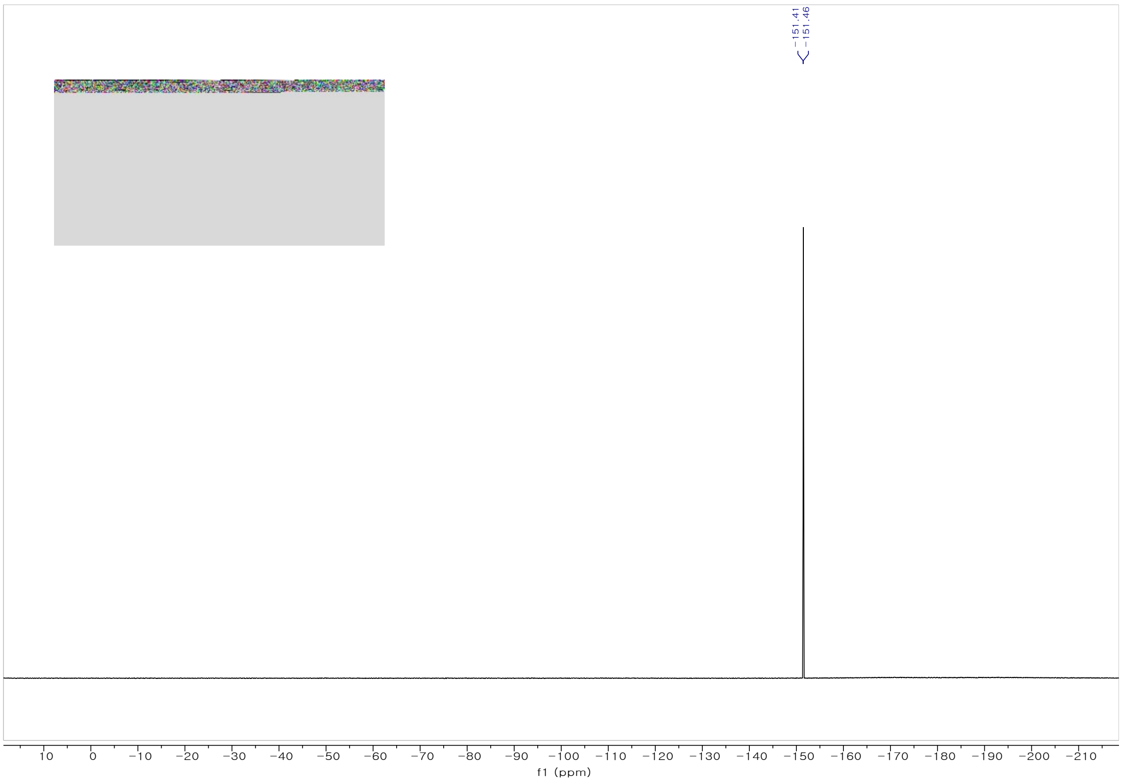

1-(10-benzyl-4-methyl-3,6,9,12,16-pentaoxo-2,17-dioxa-5,8,11-triazaoctadecan-13-yl)-2,4,6-triphenylpyridin-1-ium tetrafluoroborate (1ad).

600 MHz, ^{1}H NMR in Chloroform-*d*

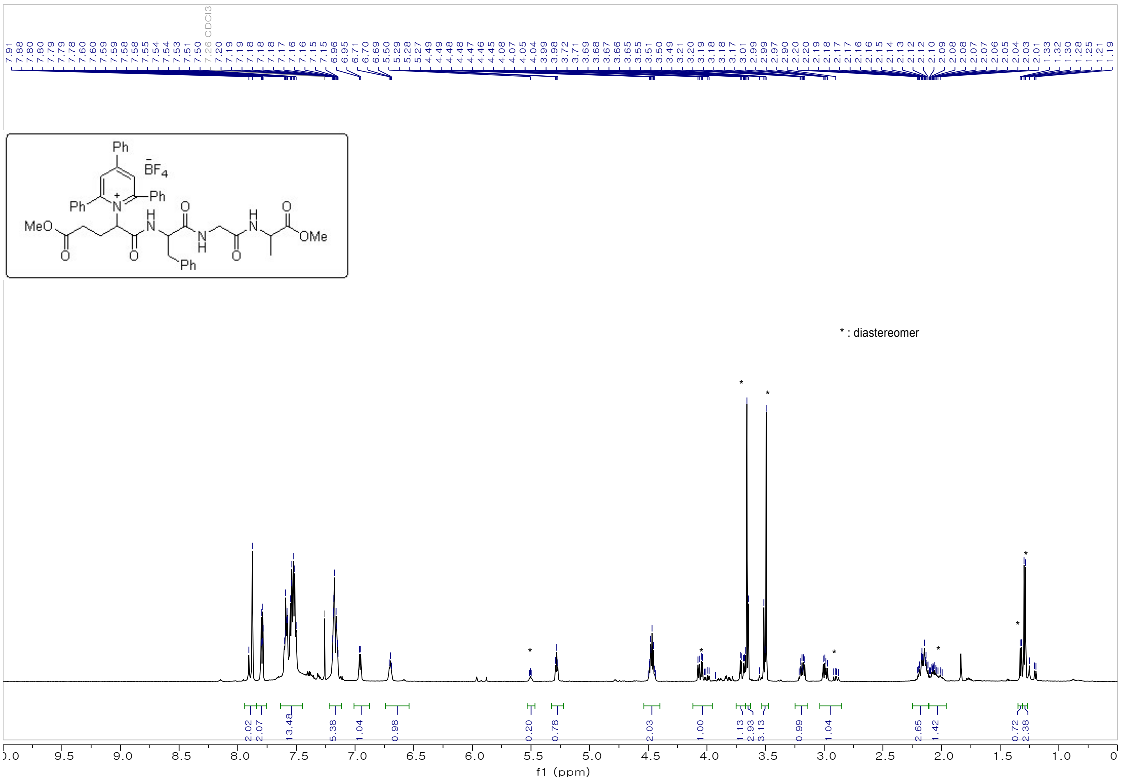

150 MHz, ^{13}C NMR in Chloroform-*d*

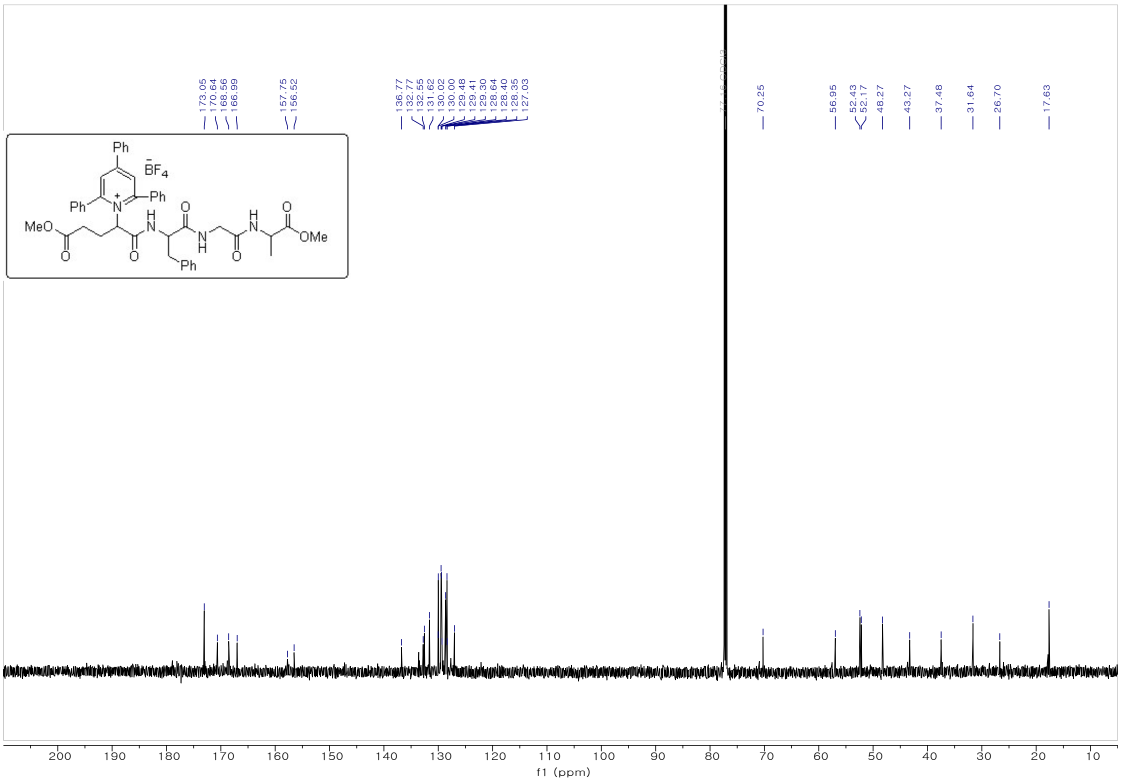

375MHz, ^{19}F NMR in Chloroform-*d*

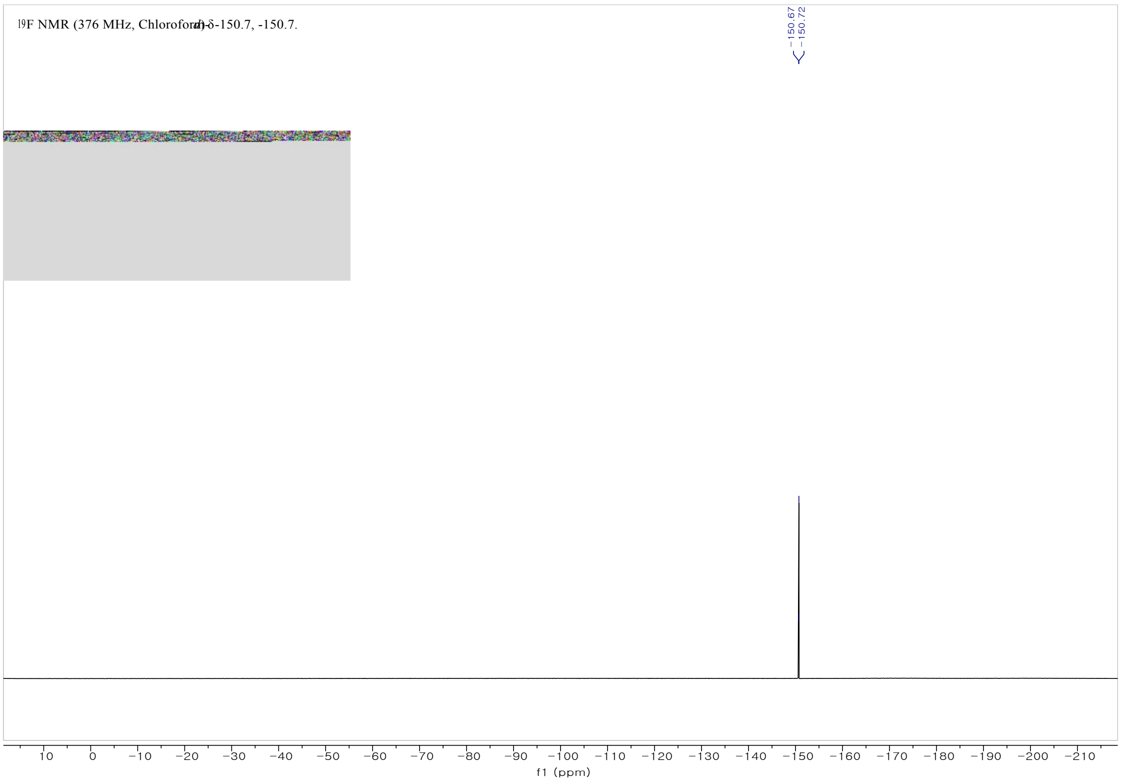

dimethyl 2-benzoylpentanedioate (3a).

600 MHz, ^{1}H NMR in Chloroform-*d*

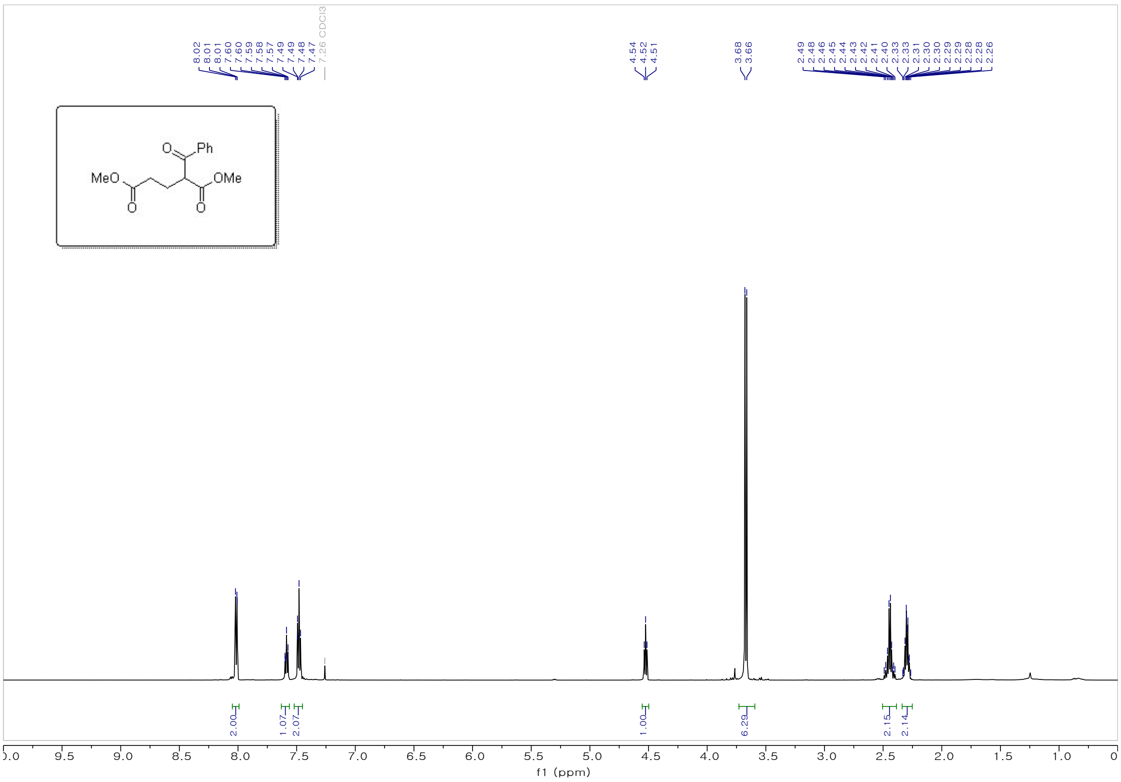

150 MHz, ^{13}C NMR in Chloroform-*d*

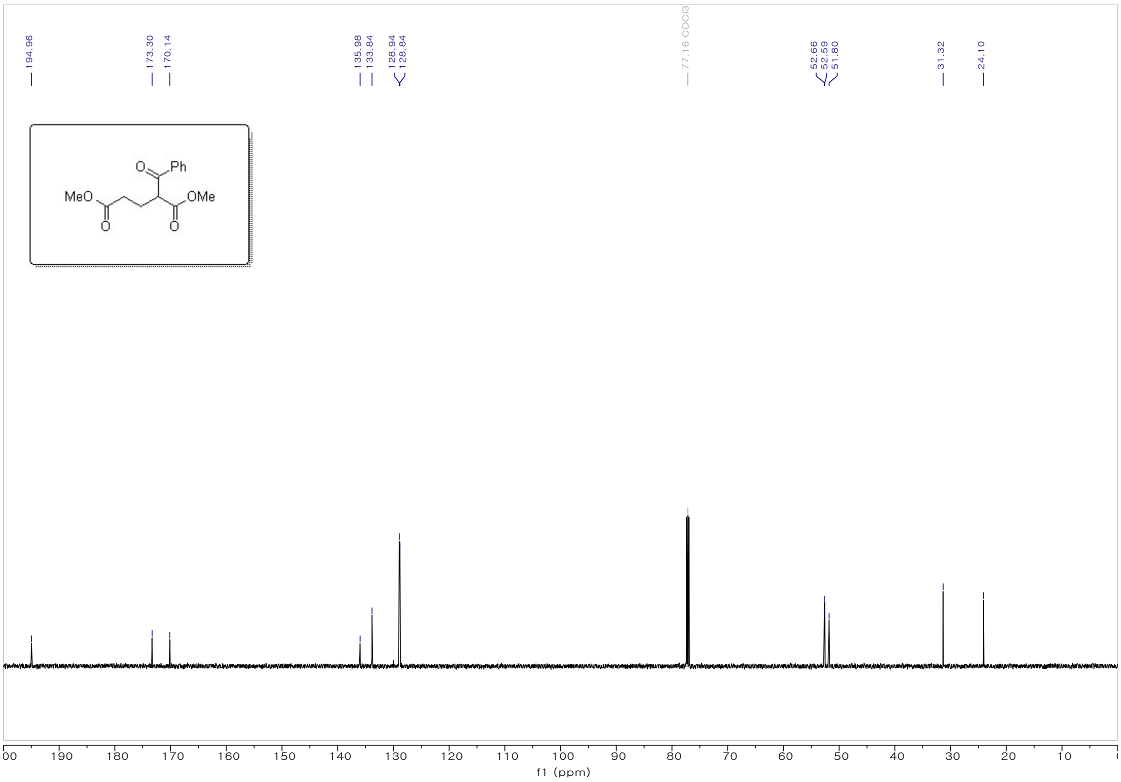

methyl 2-methyl-3-oxo-3-phenylpropanoate (3b).

400 MHz, ^{1}H NMR in Chloroform-*d*

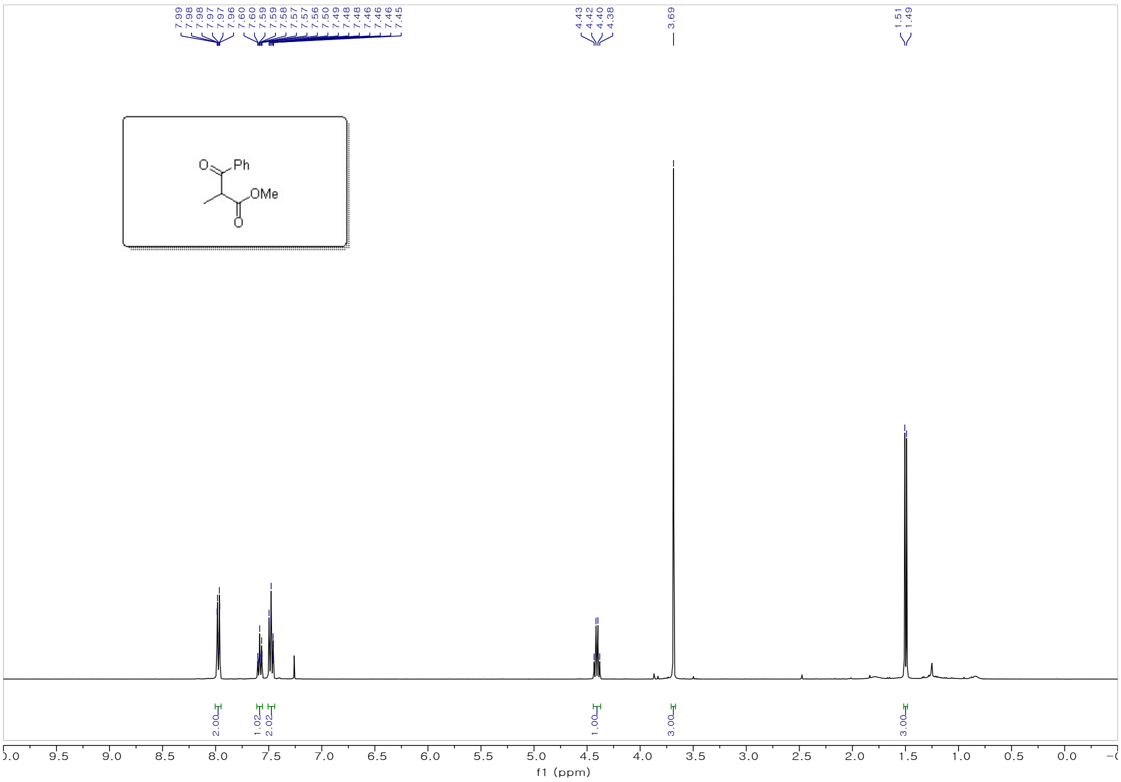

100 MHz, ^{13}C NMR in Chloroform-*d*

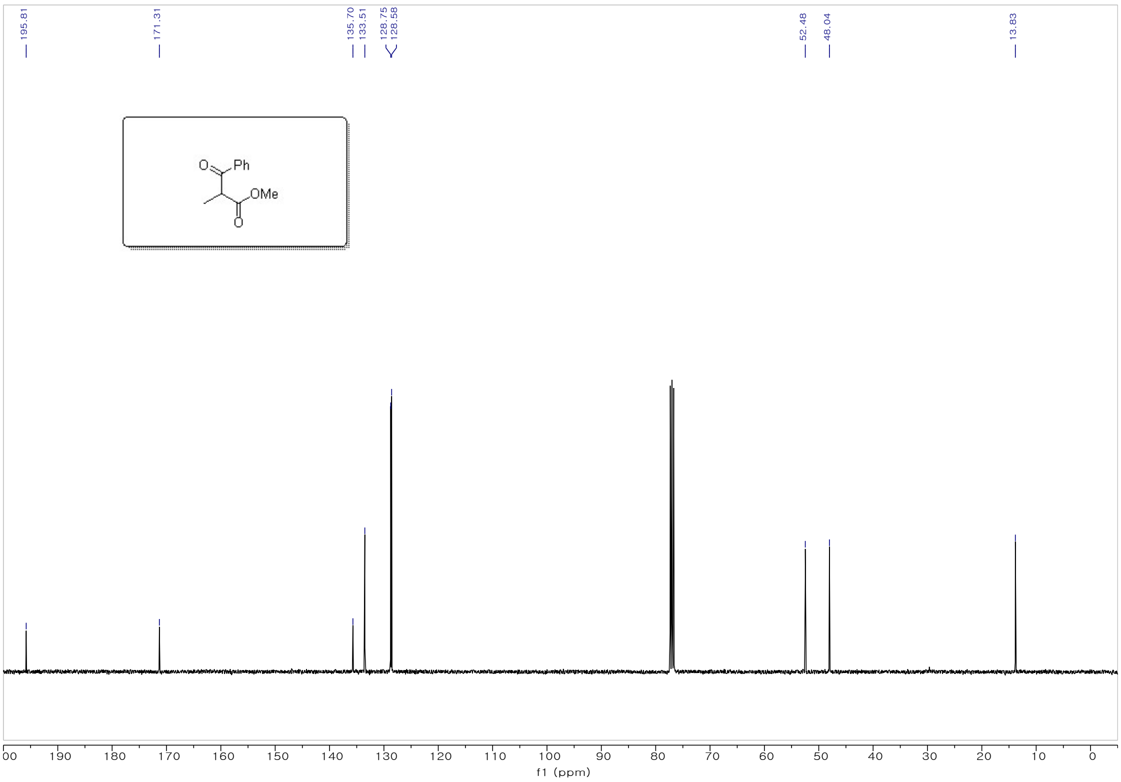

methyl 2-benzoylbutanoate (3c).

400 MHz, ^{1}H NMR in Chloroform-*d*

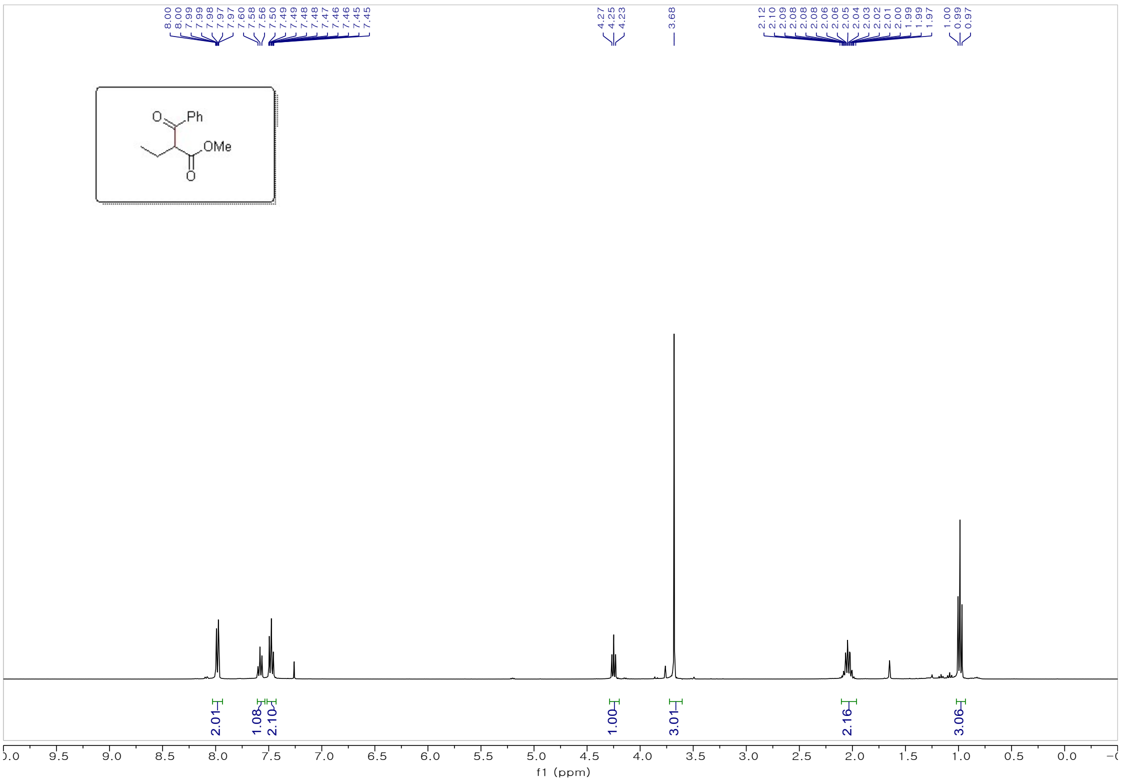

100 MHz, ^{13}C NMR in Chloroform-*d*

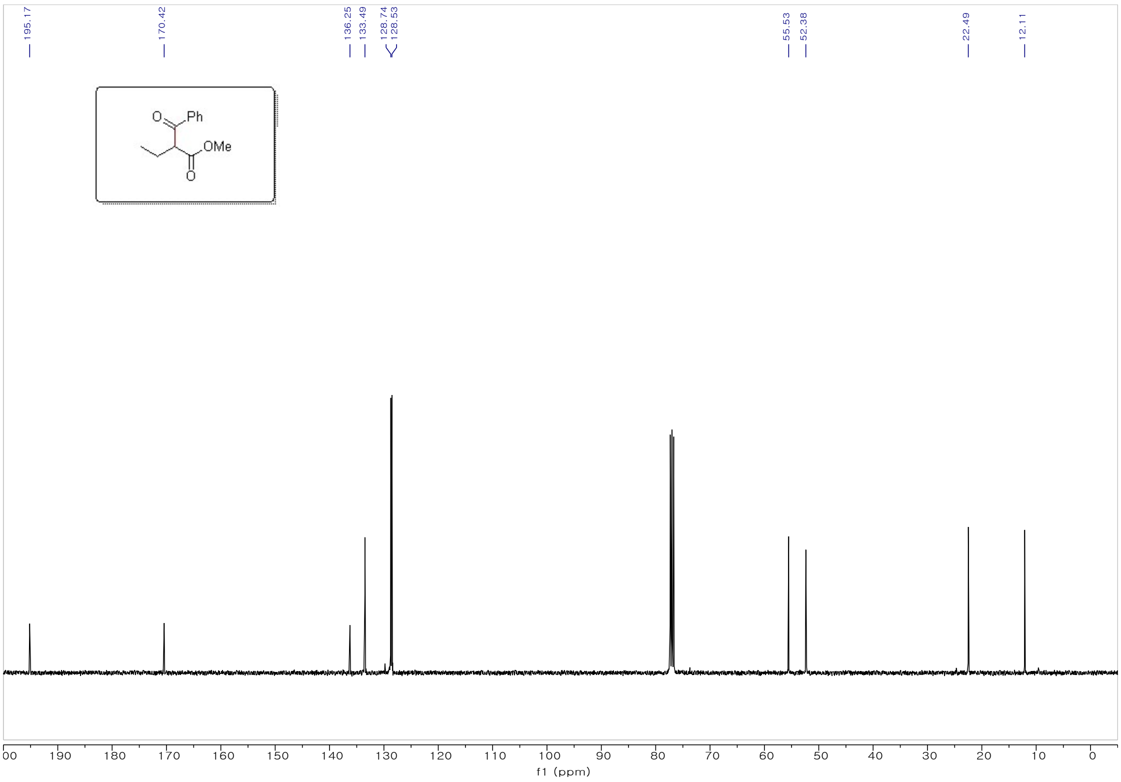

methyl 2-benzoyl-3-methylbutanoate (3d).

400 MHz, ^{1}H NMR in Chloroform-*d*

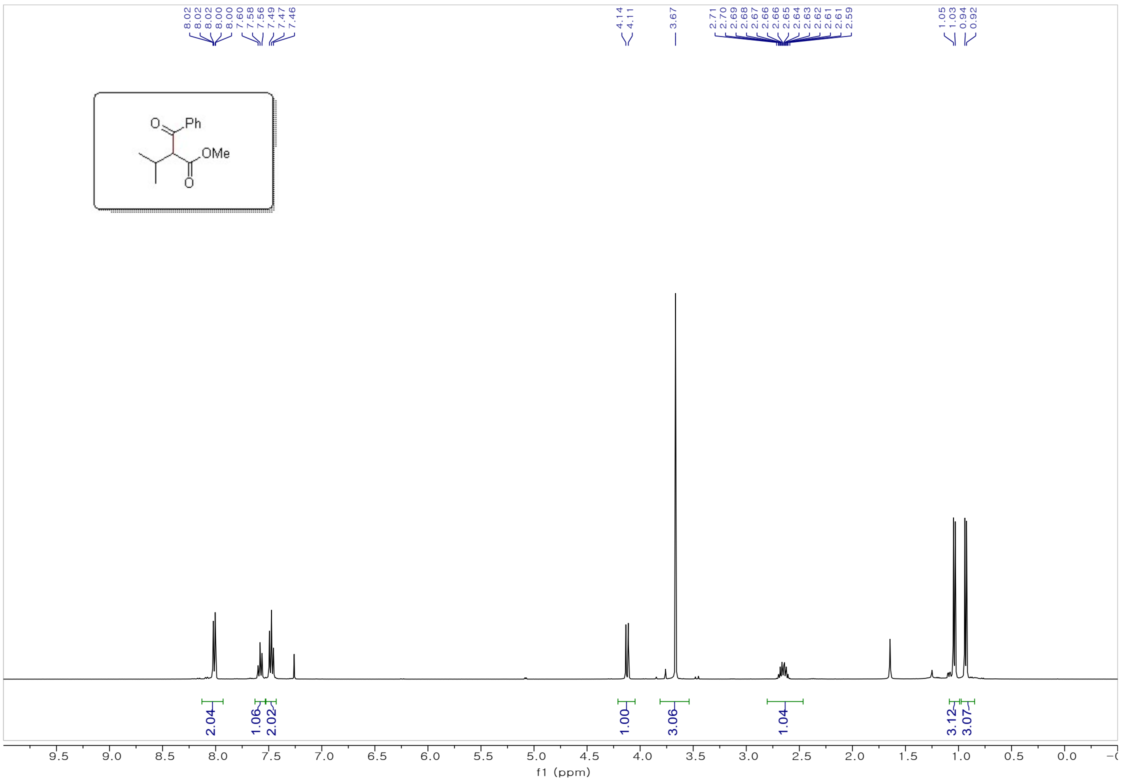

100 MHz, ^{13}C NMR in Chloroform-*d*

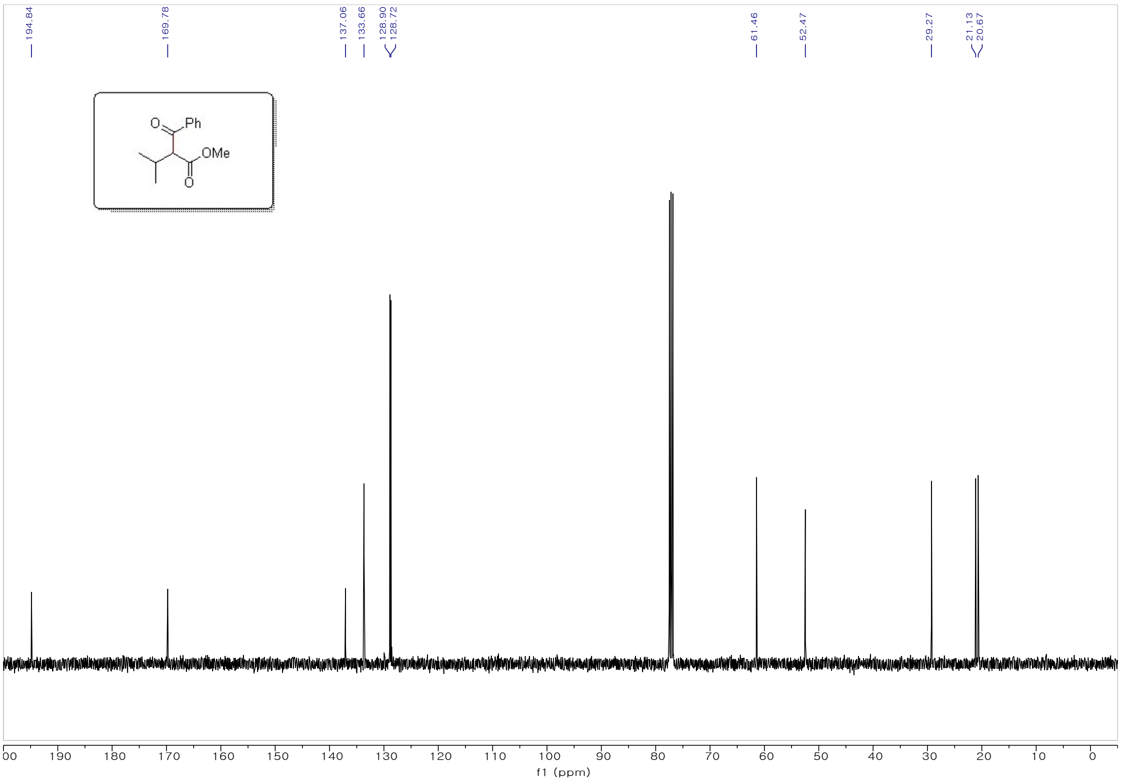

ethyl 2-benzoylpentanoate (3e).

400 MHz, ^{1}H NMR in Chloroform-*d*

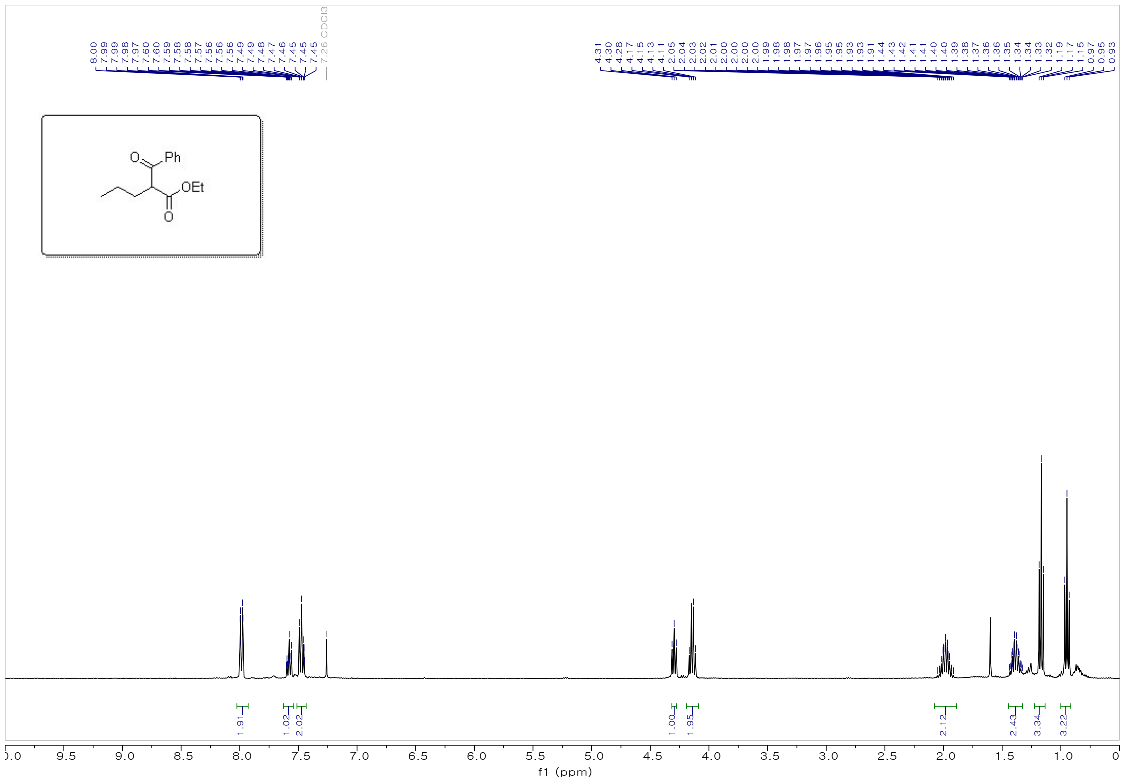

150 MHz, ^{13}C NMR in Chloroform-*d*

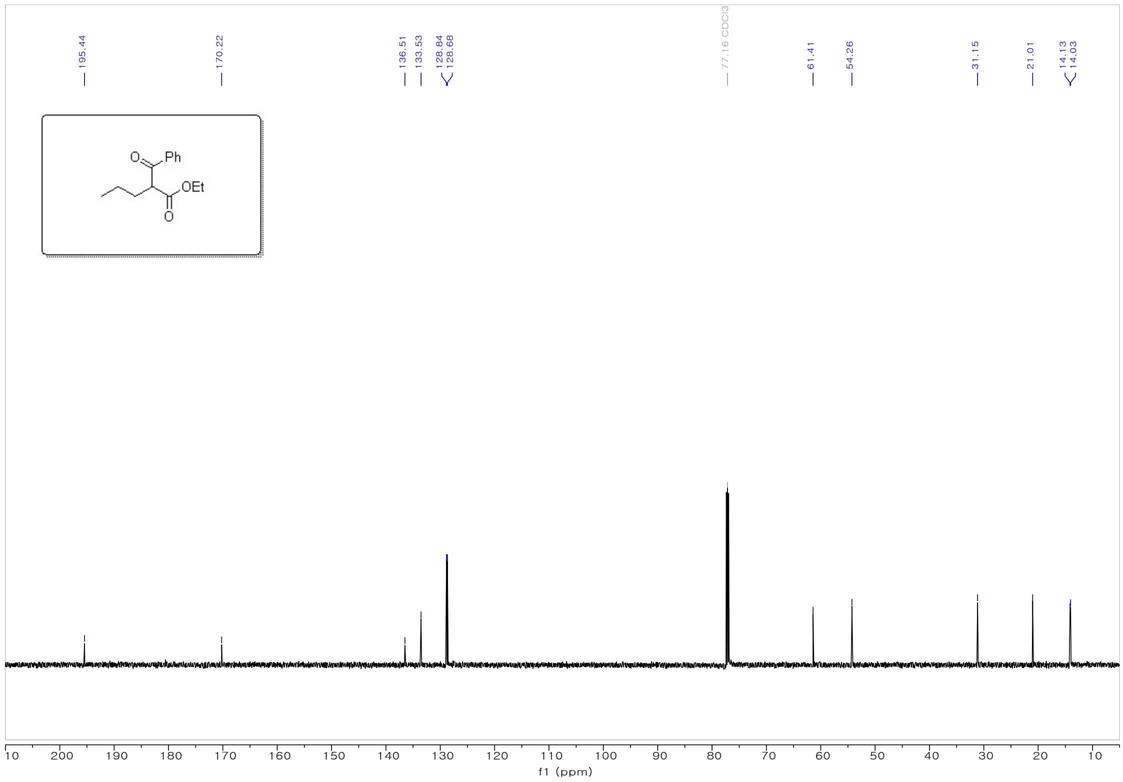

methyl 2-benzoyl-4-(methylthio)butanoate (3f).

400 MHz, ^{1}H NMR in Chloroform-*d*

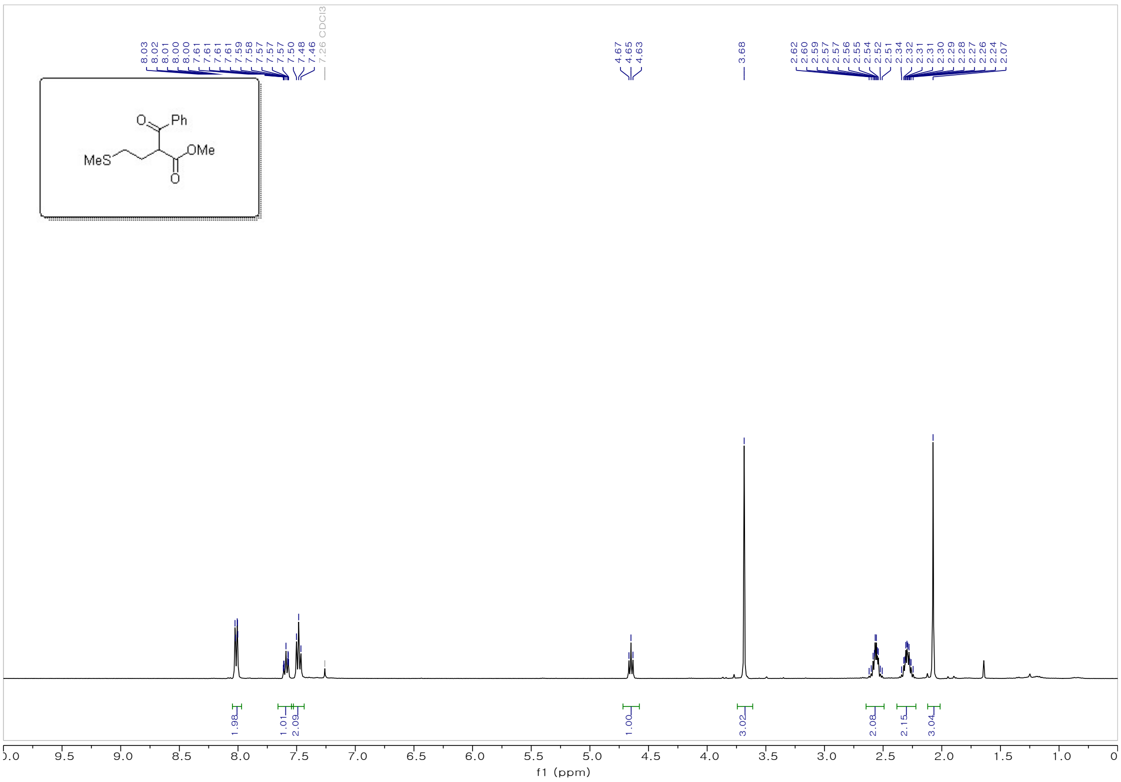

100 MHz, ^{13}C NMR in Chloroform-*d*

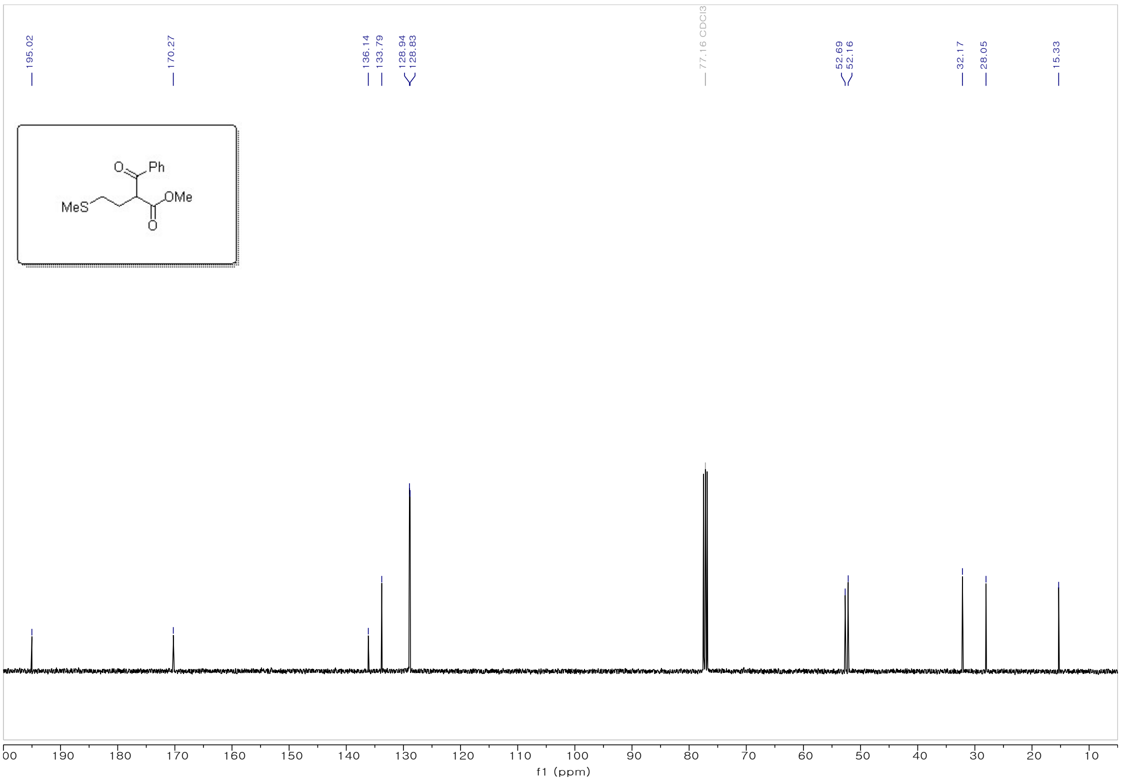

methyl 2-benzyl-3-oxo-3-phenylpropanoate (3g).

600 MHz, ^{1}H NMR in Chloroform-*d*

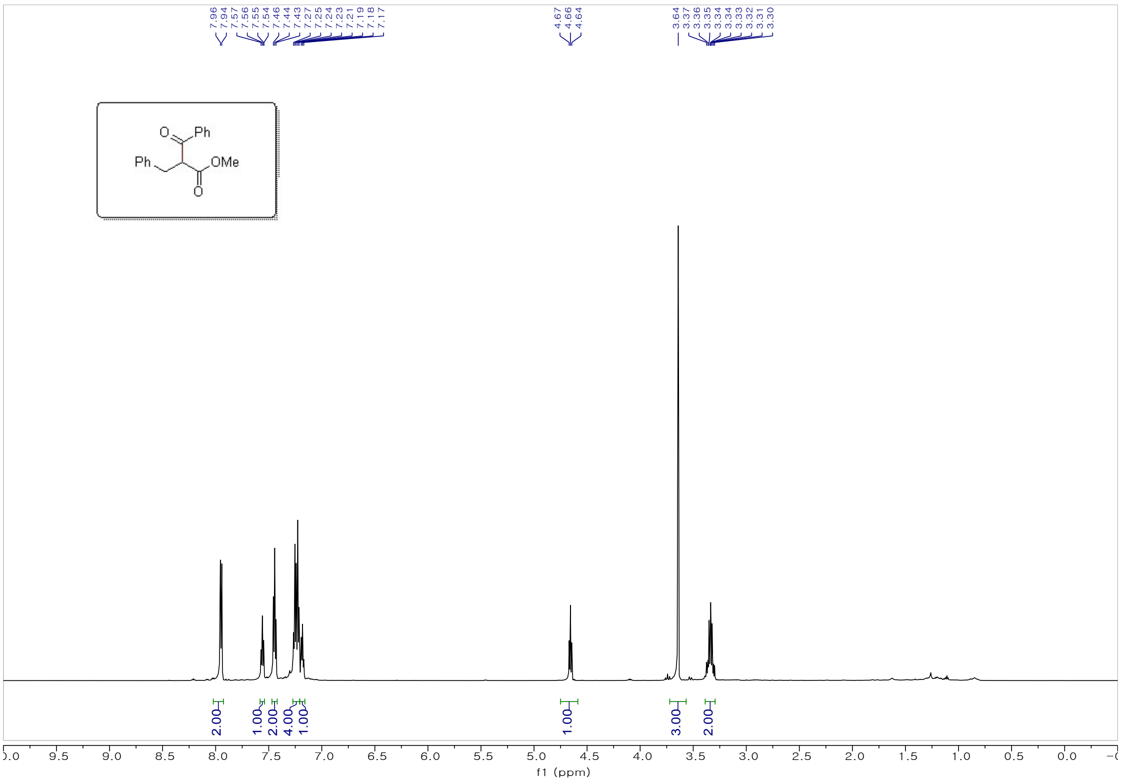

150 MHz, ^{13}C NMR in Chloroform-*d*

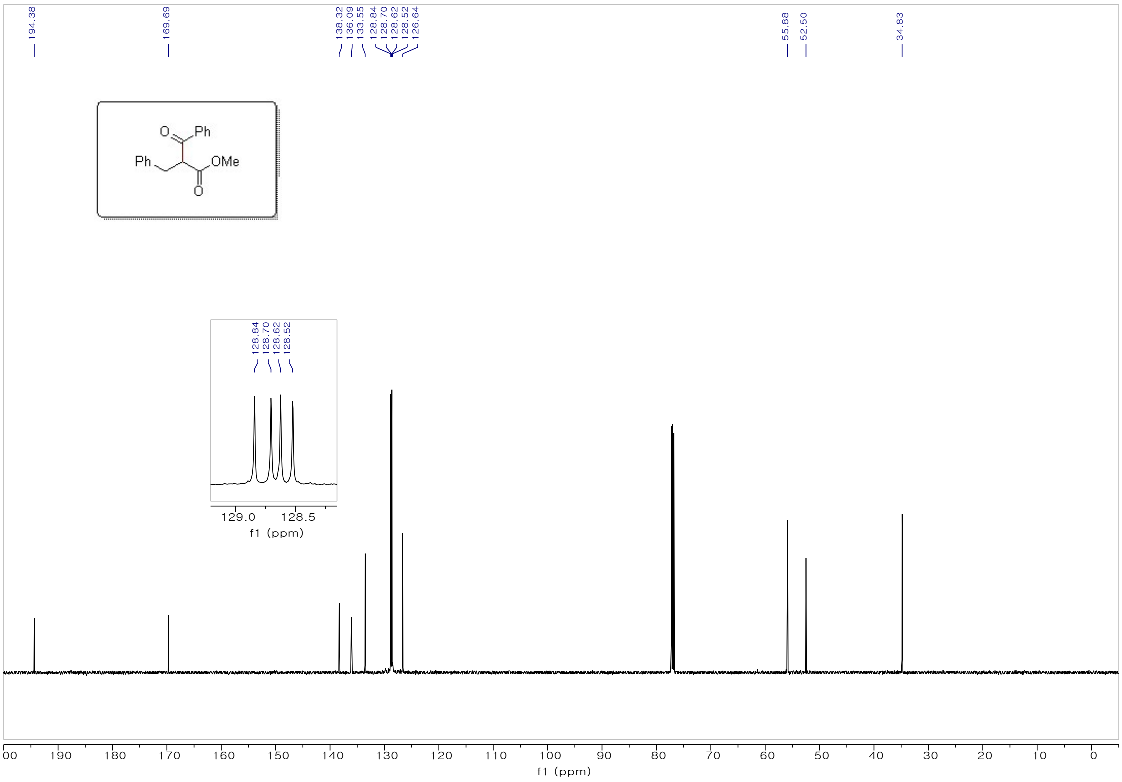

ethyl 2-benzoyl-4-phenylbutanoate (3h).

600 MHz, ^{1}H NMR in Chloroform-*d*

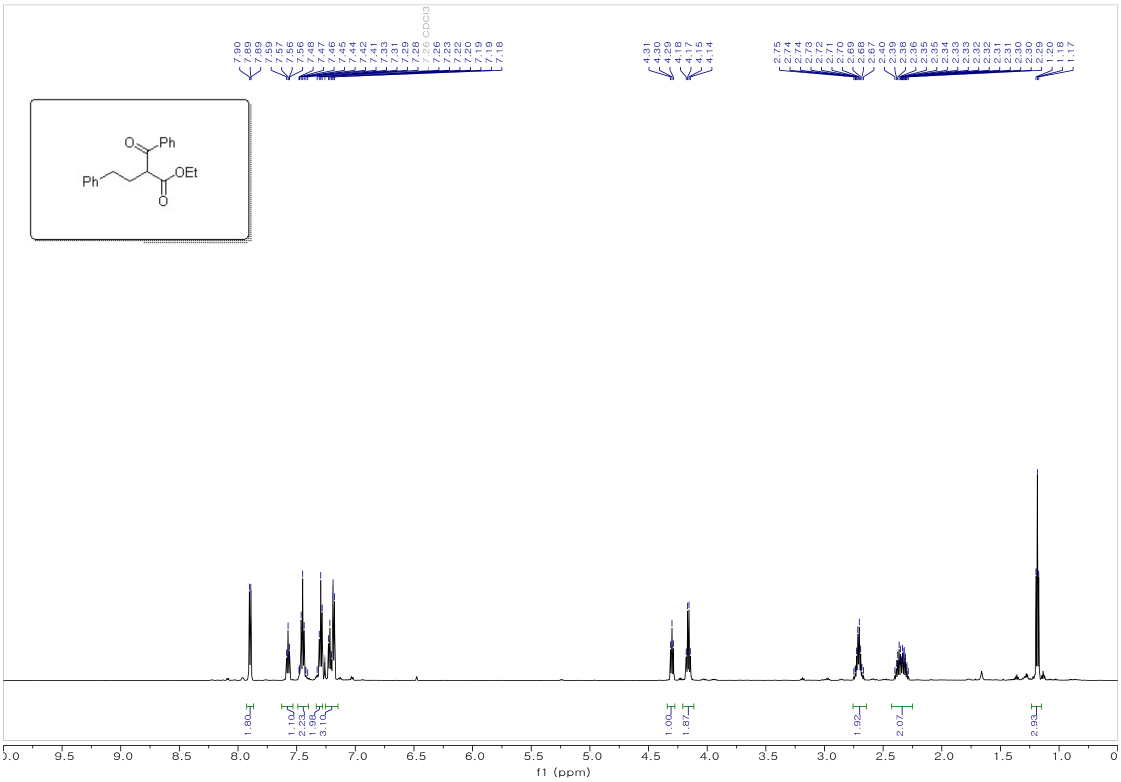

150 MHz, ^{13}C NMR in Chloroform-*d*

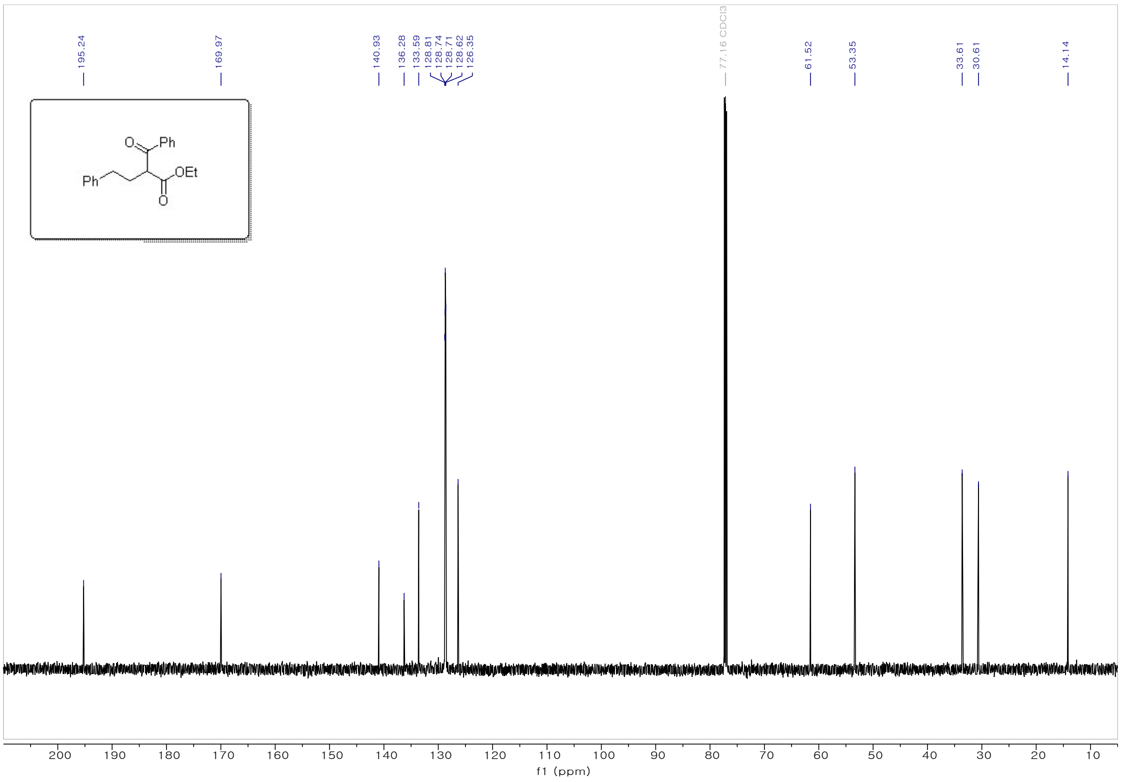

methyl 2-(4-chlorobenzyl)-3-oxo-3-phenylpropanoate (3i).

400 MHz, ^{1}H NMR in Chloroform-d

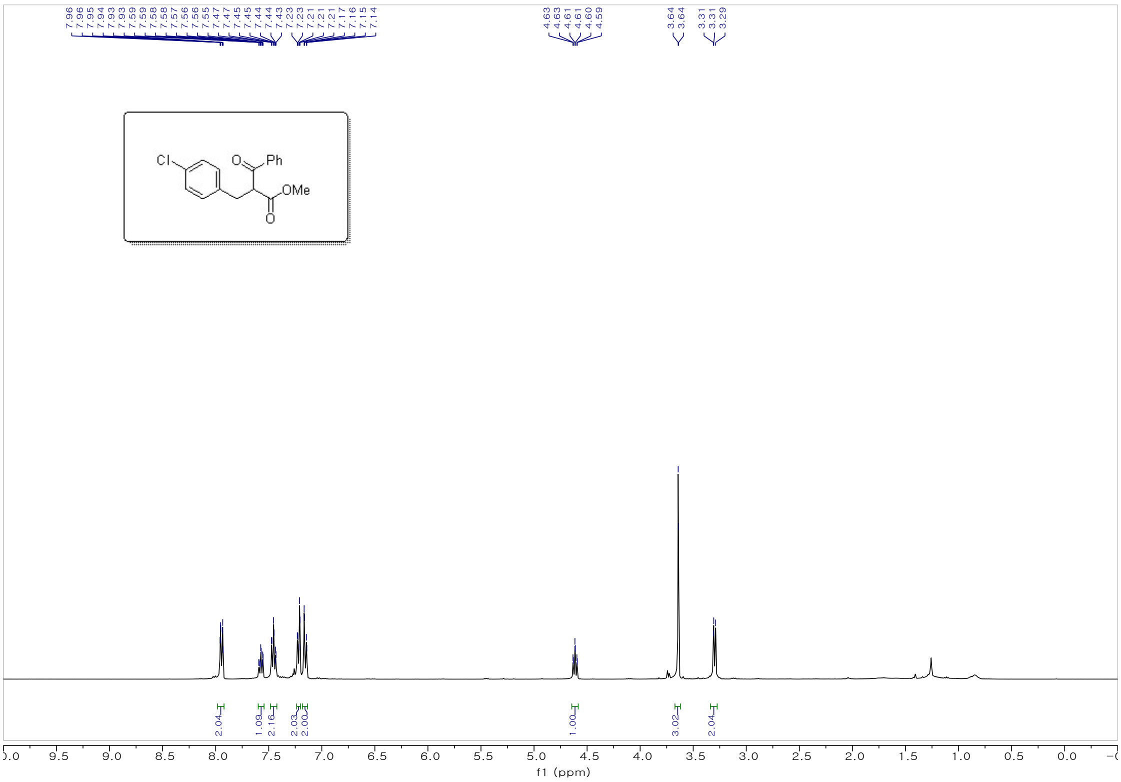

100 MHz, ^{13}C NMR in Chloroform-d

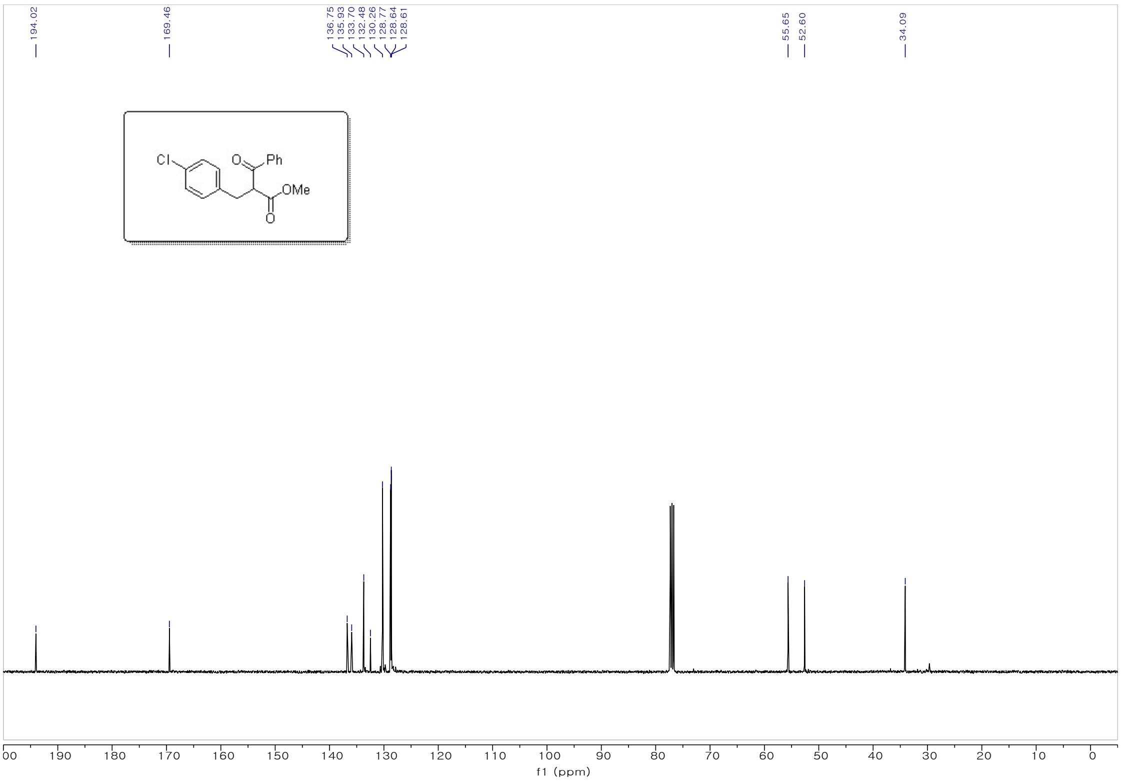

methyl 2-(4-hydroxybenzyl)-3-oxo-3-phenylpropanoate (3j).

400 MHz, ^{1}H NMR in Methylene Chloride-d_2

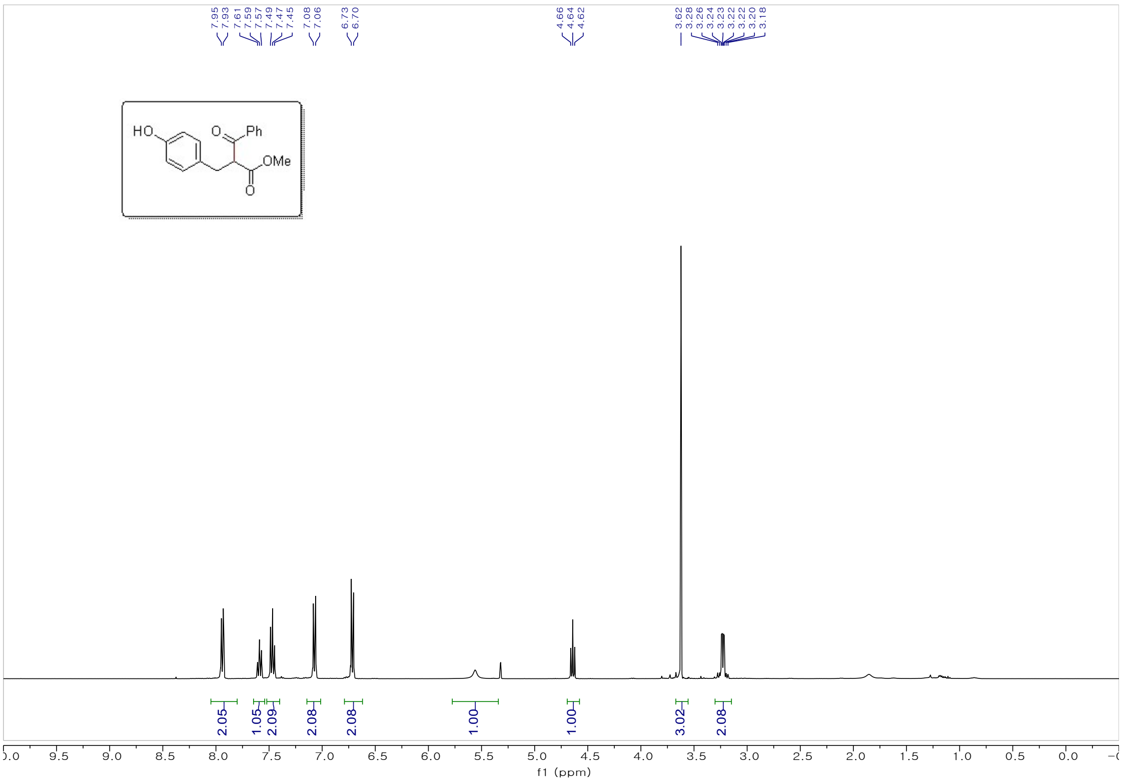

100 MHz, ^{13}C NMR in Methylene Chloride-d_2

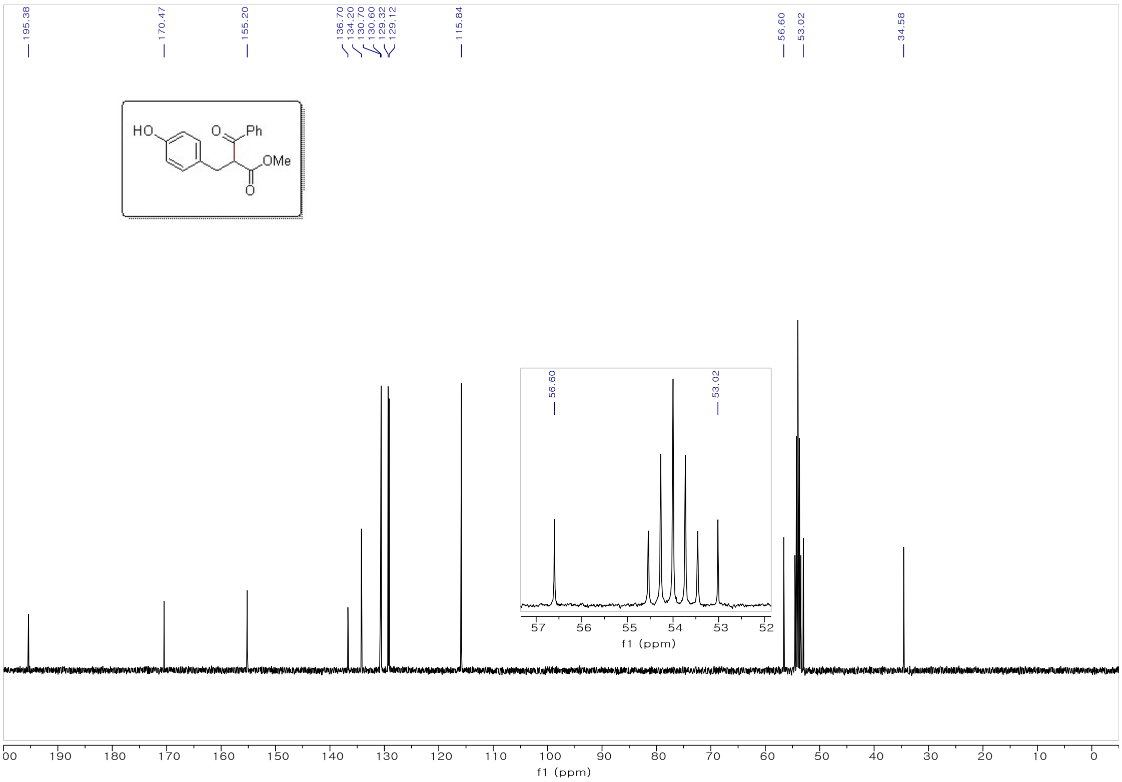

methyl 2-benzoylpent-4-enoate (3k).

600 MHz, ^{1}H NMR in Chloroform-*d*

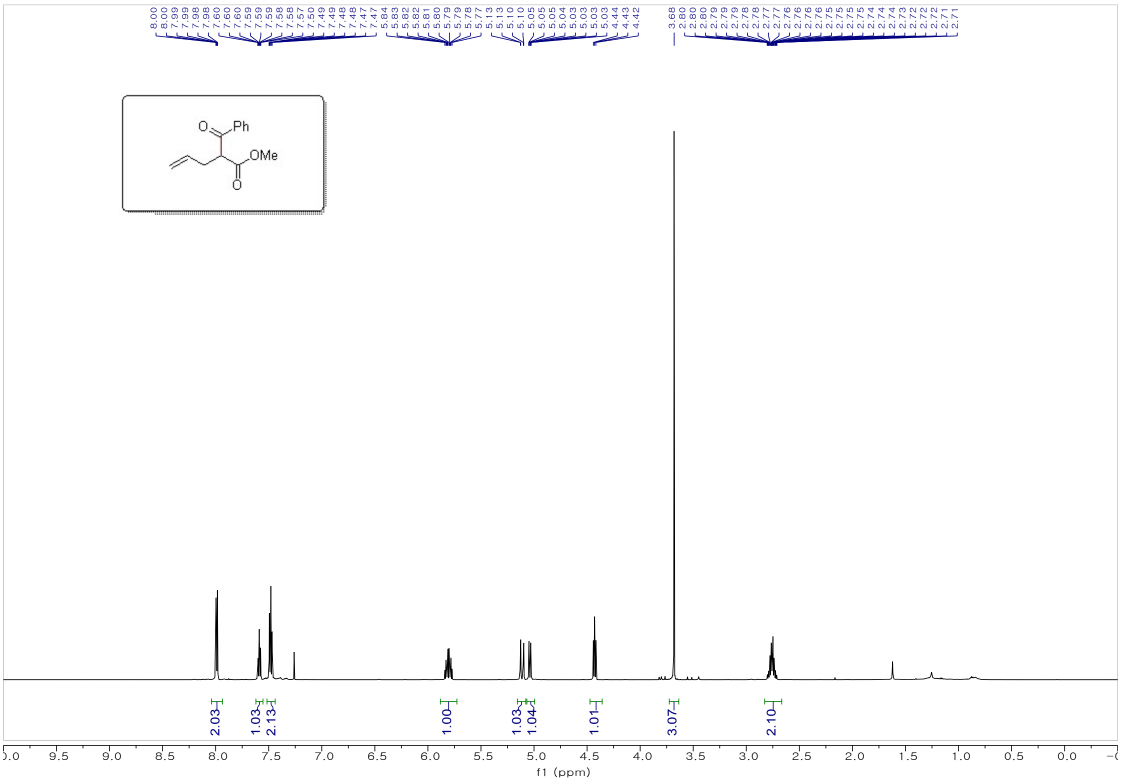

150 MHz, ^{13}C NMR in Chloroform-*d*

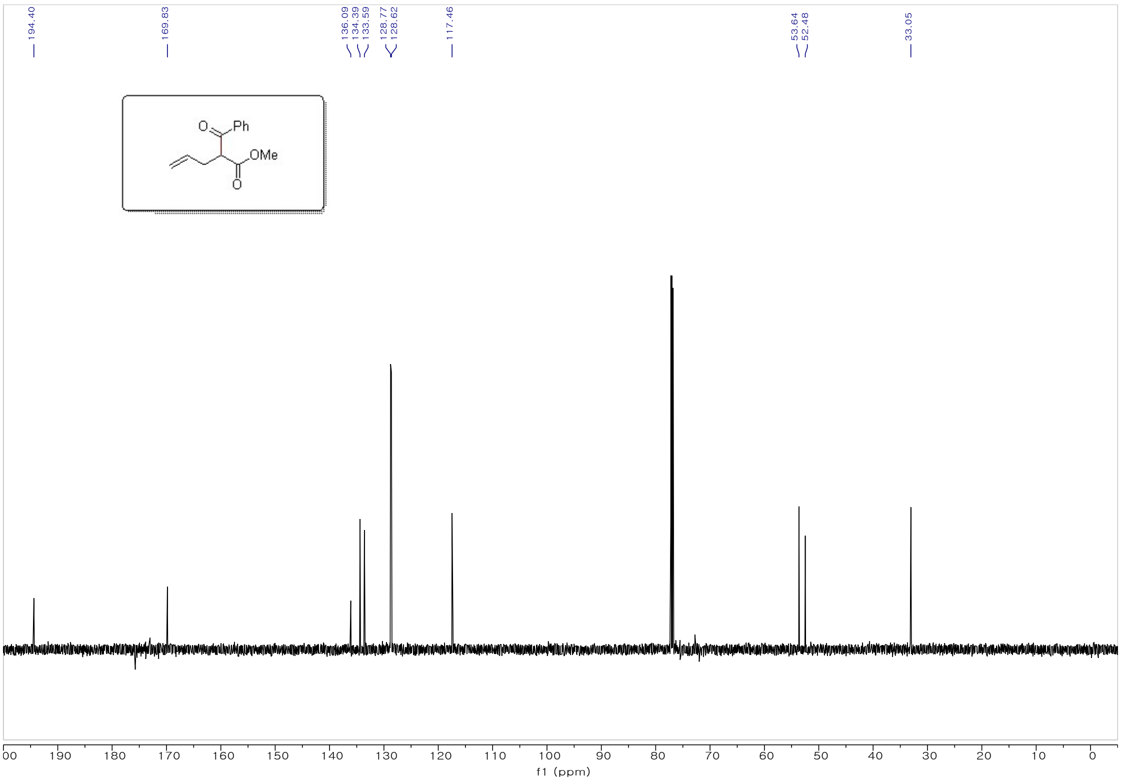

methyl 2-benzoyl-4-methylpentanoate (3l).

400 MHz, ^{1}H NMR in Chloroform-*d*

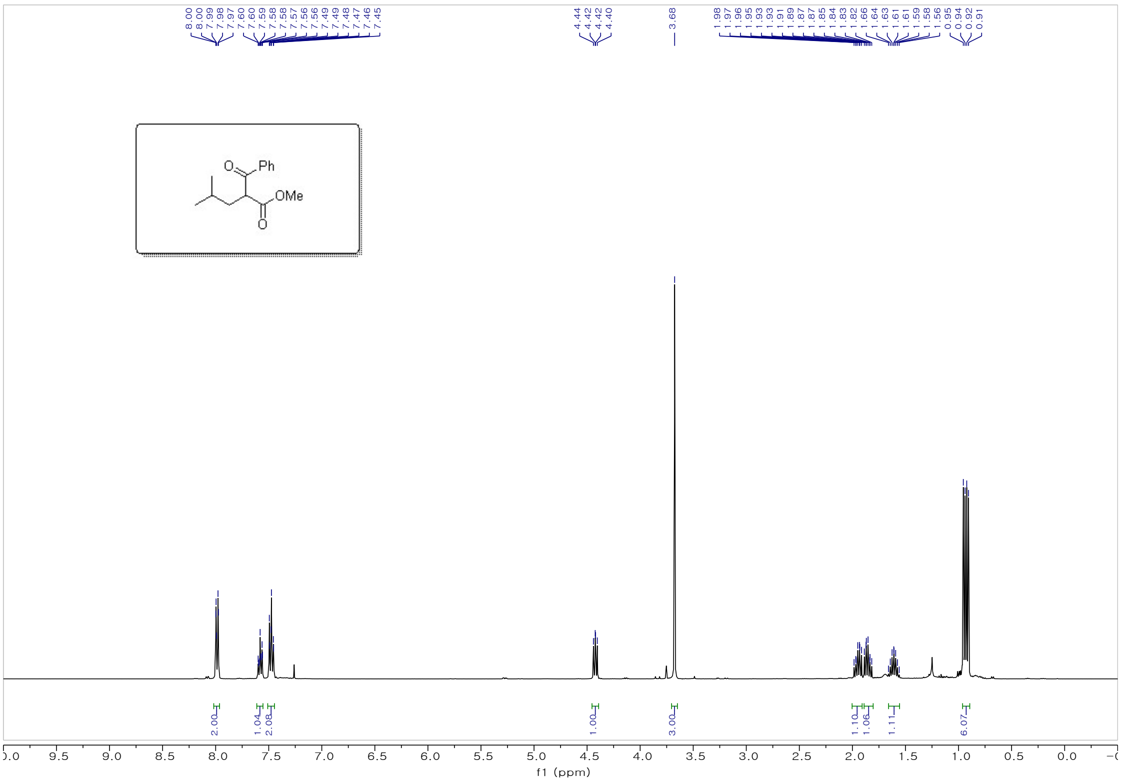

100 MHz, ^{13}C NMR in Chloroform-*d*

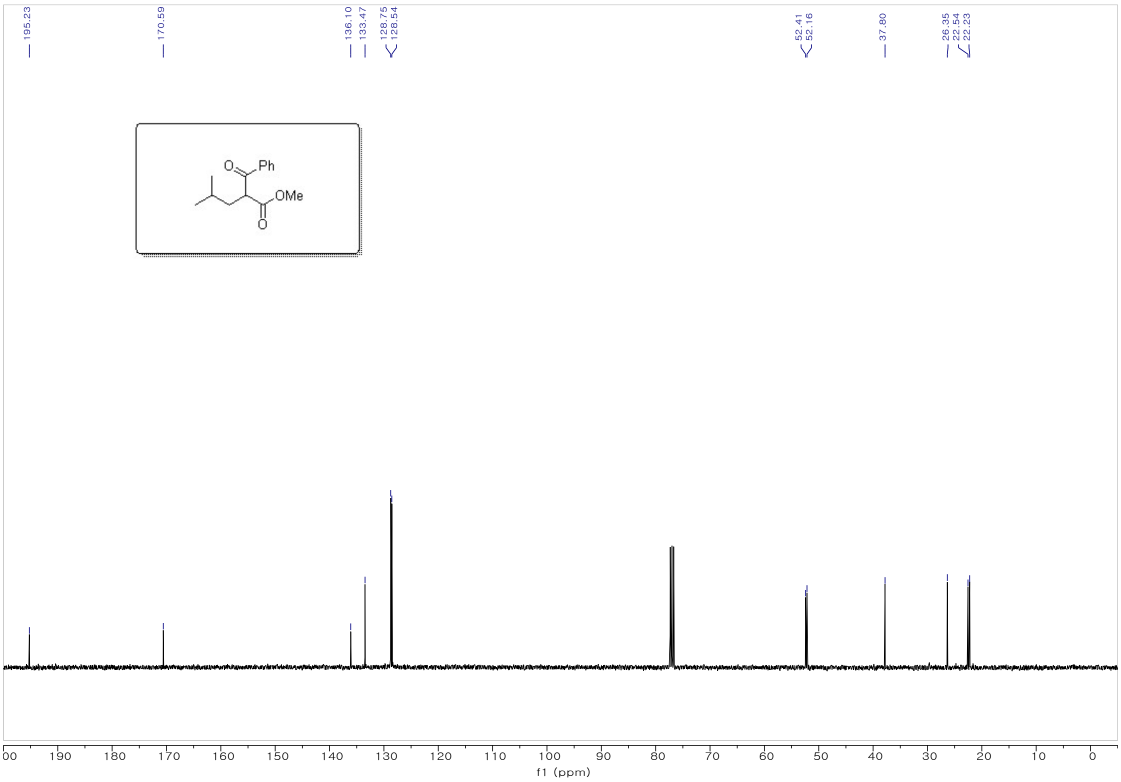

2-benzoyl-4-methyl-N-(naphthalen-2-yl)pentanamide (3m).

400 MHz, ^{1}H NMR in Chloroform-*d*

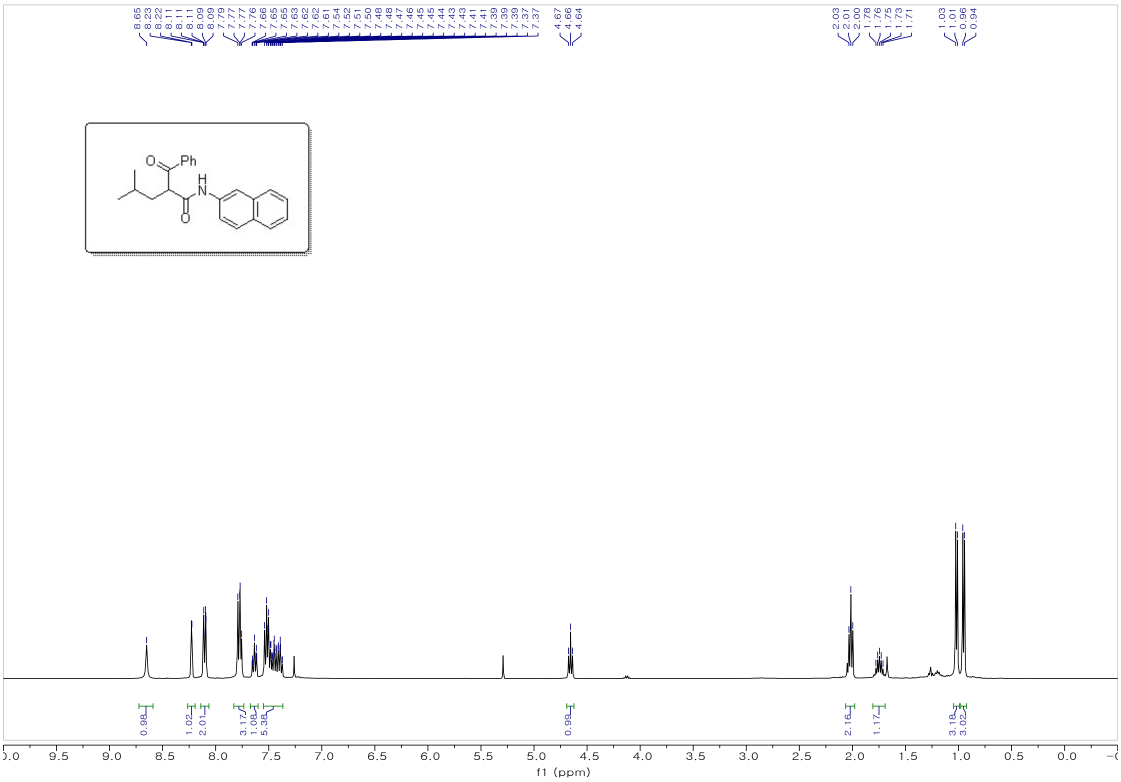

100 MHz, ^{13}C NMR in Chloroform-*d*

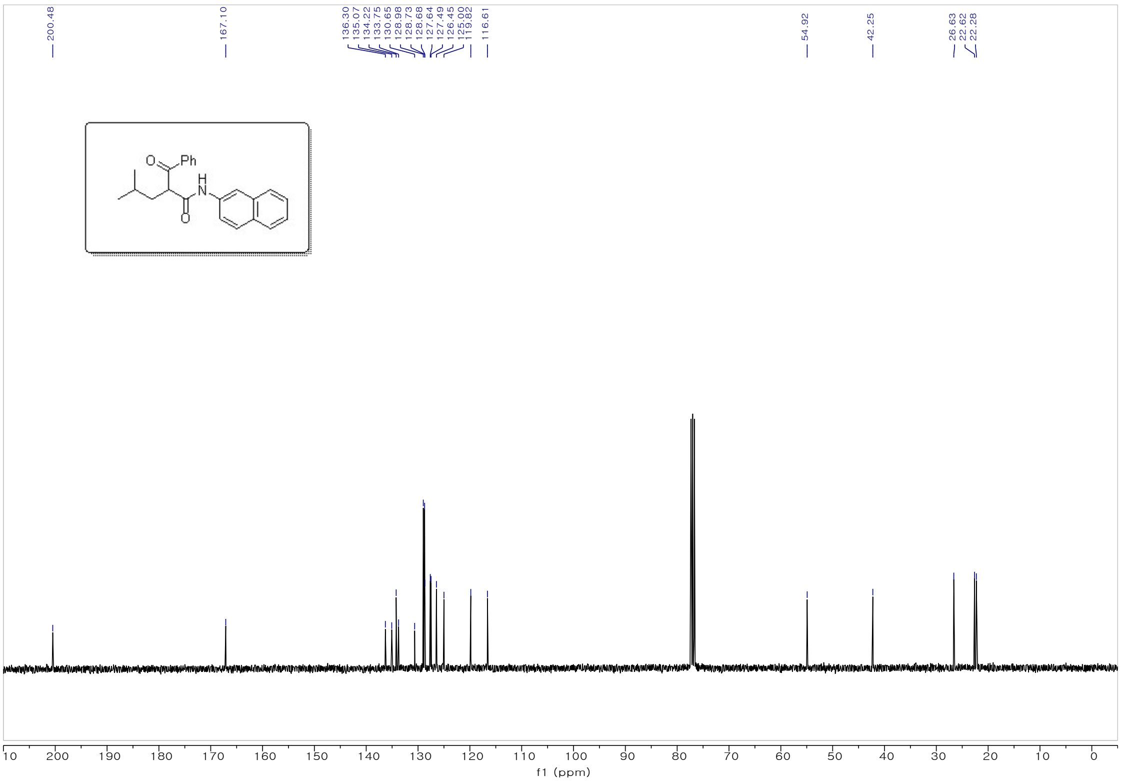

methyl 2-benzoyl-6-(((benzyloxy)carbonyl)amino)hexanoate (3n).

600 MHz, ^{1}H NMR in Chloroform-*d*

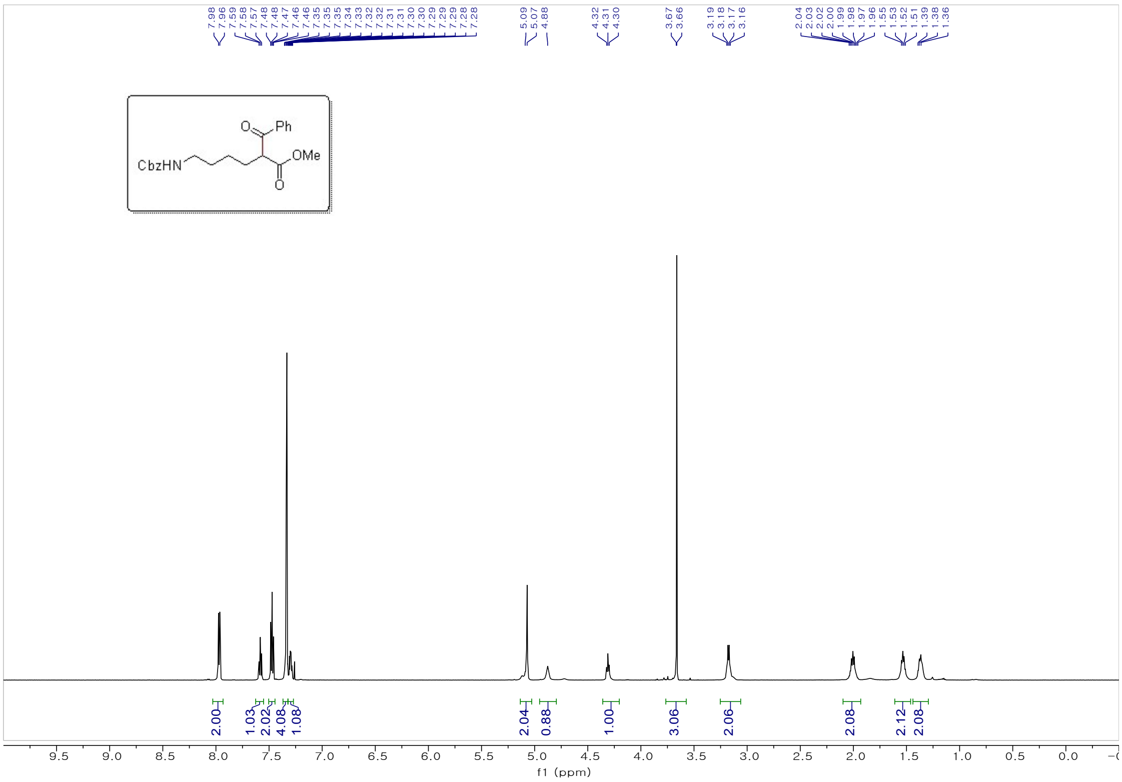

100 MHz, ^{13}C NMR in Chloroform-*d*

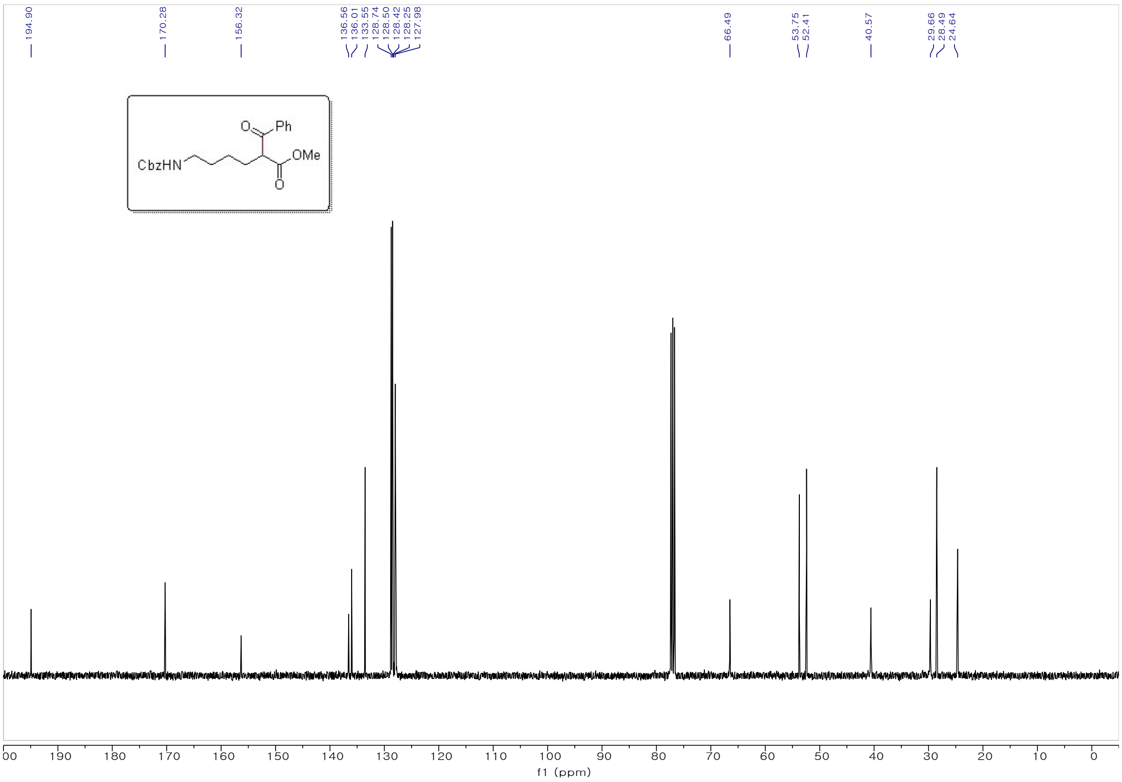

methyl 2-((1H-indol-3-yl)methyl)-3-oxo-3-phenylpropanoate (3o).

400 MHz, ^{1}H NMR in Methylene Chloride-d_2

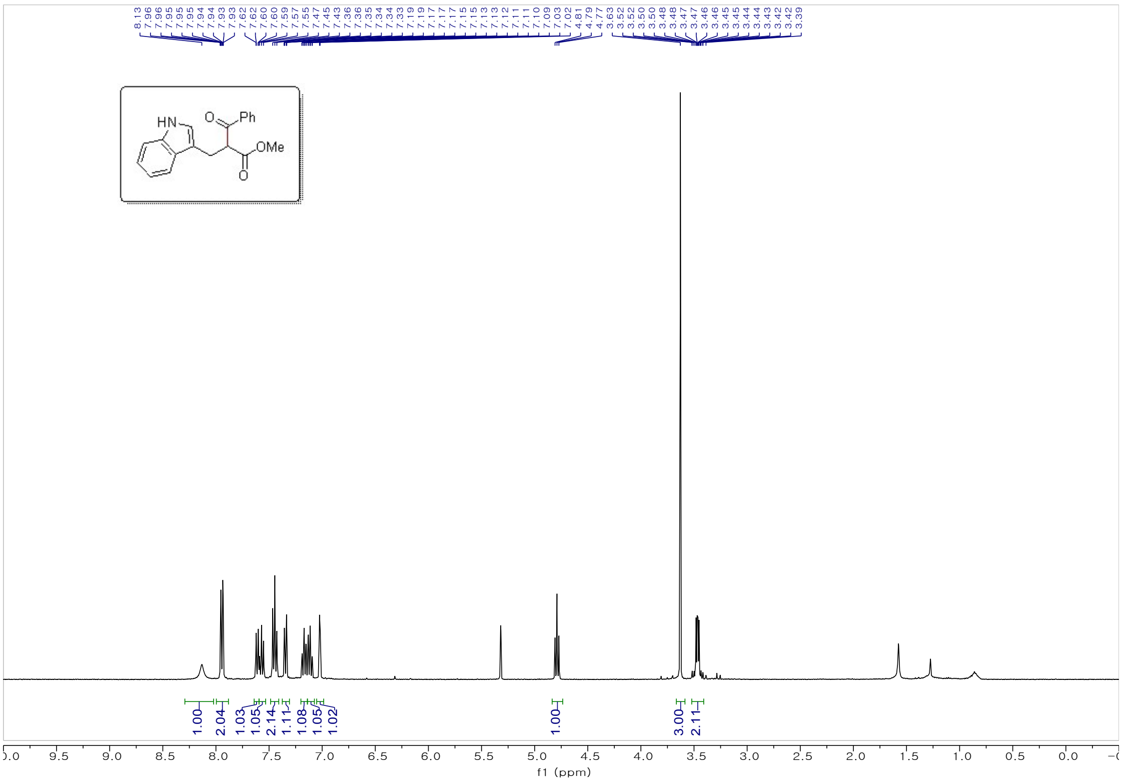

100 MHz, ^{13}C NMR in Methylene Chloride-d_2

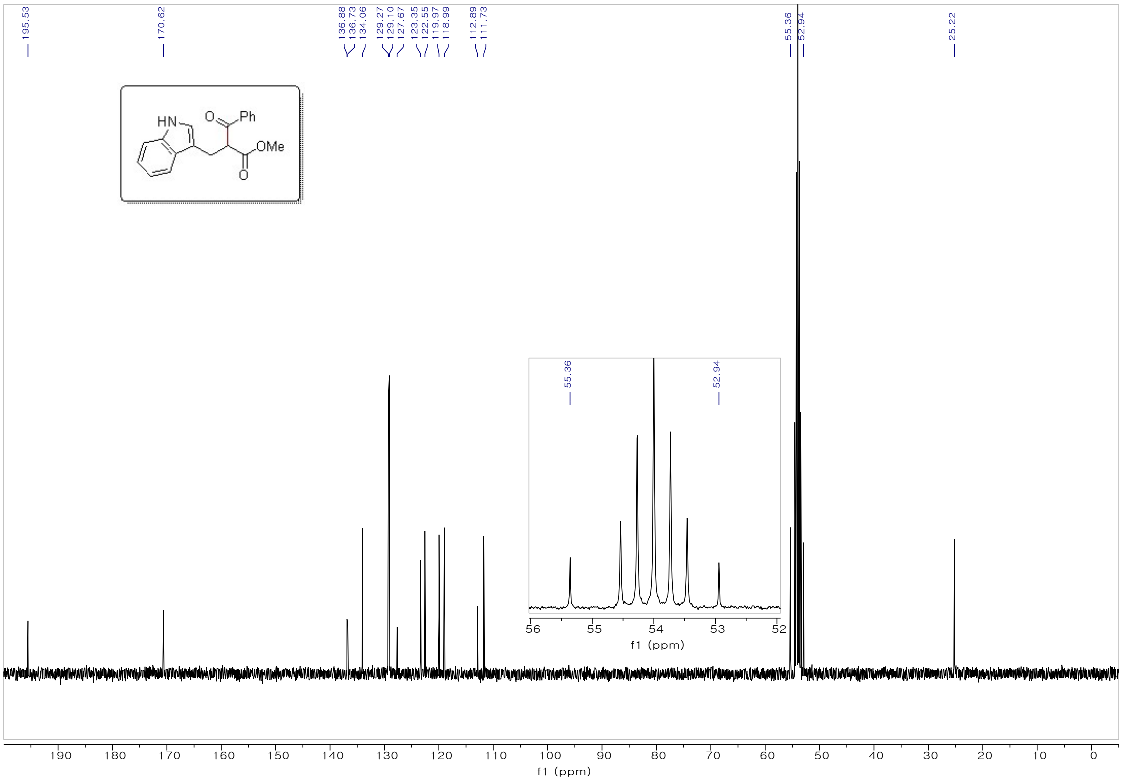

tert-butyl 4-(4-(methoxycarbonyl)benzoyl)piperidine-1-carboxylate (3p).

600 MHz, ^{1}H NMR in Chloroform-*d*

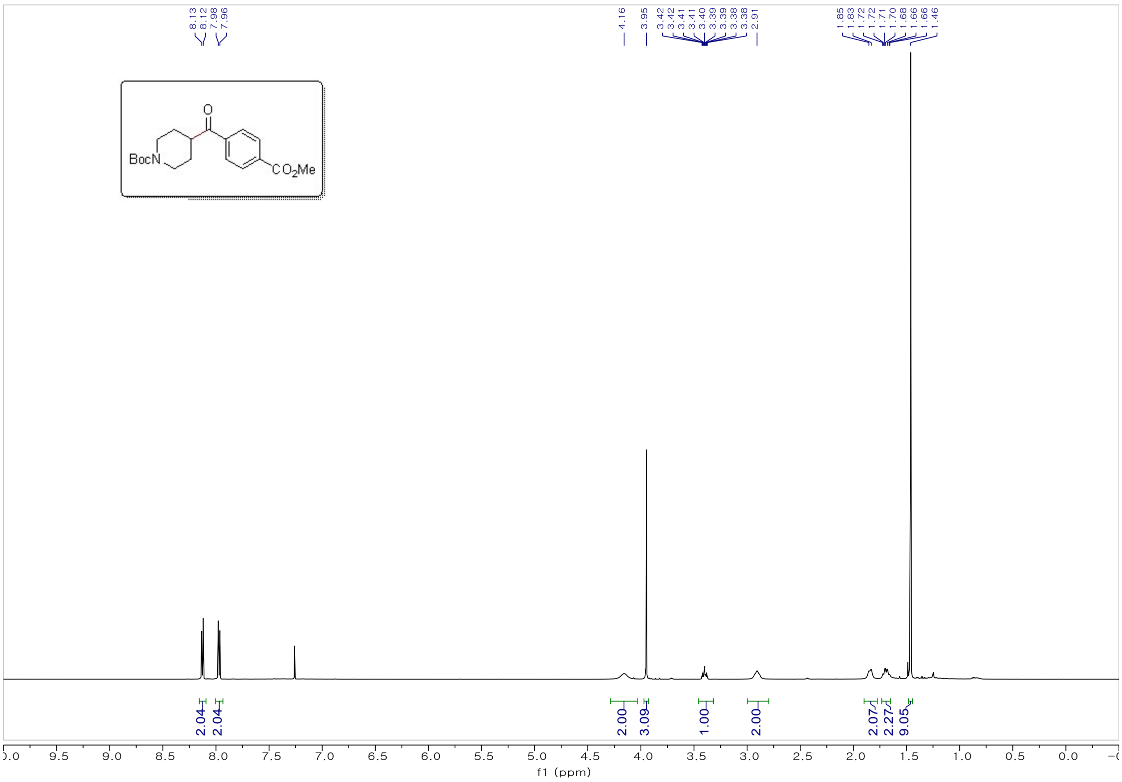

150 MHz, ^{13}C NMR in Chloroform-*d*

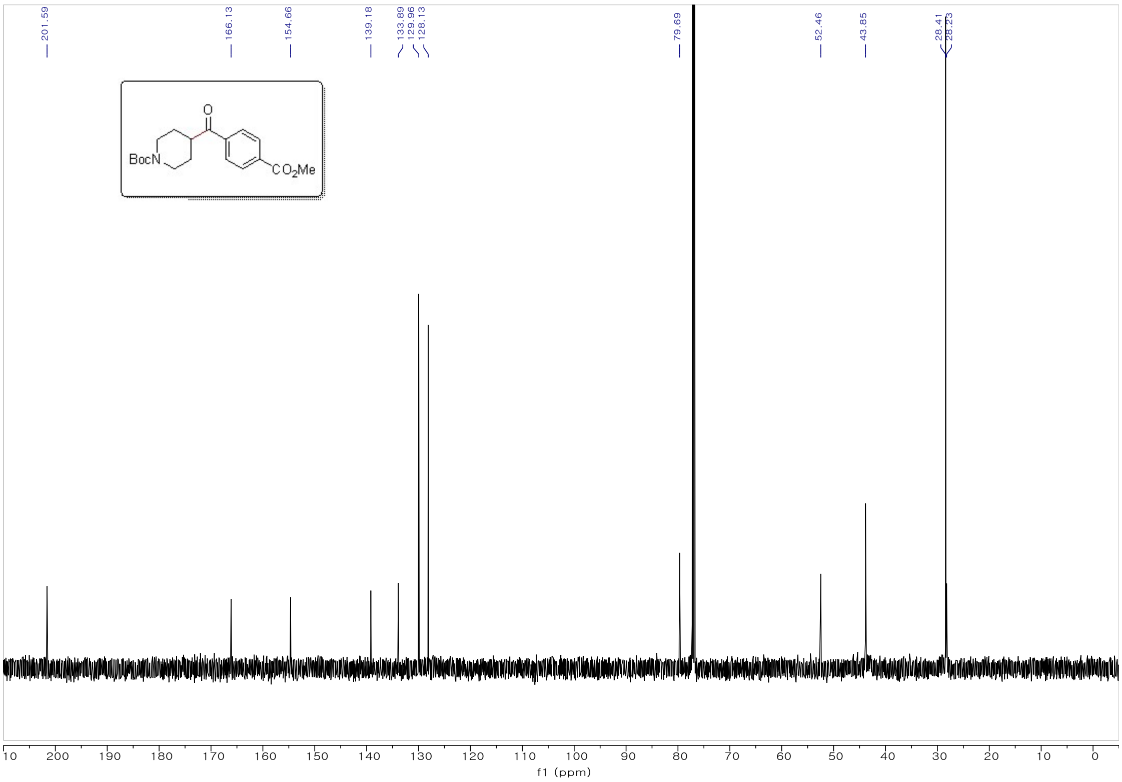

methyl 4-(tetrahydro-2H-pyran-4-carbonyl)benzoate (3q).

600 MHz, ^{1}H NMR in Chloroform-*d*

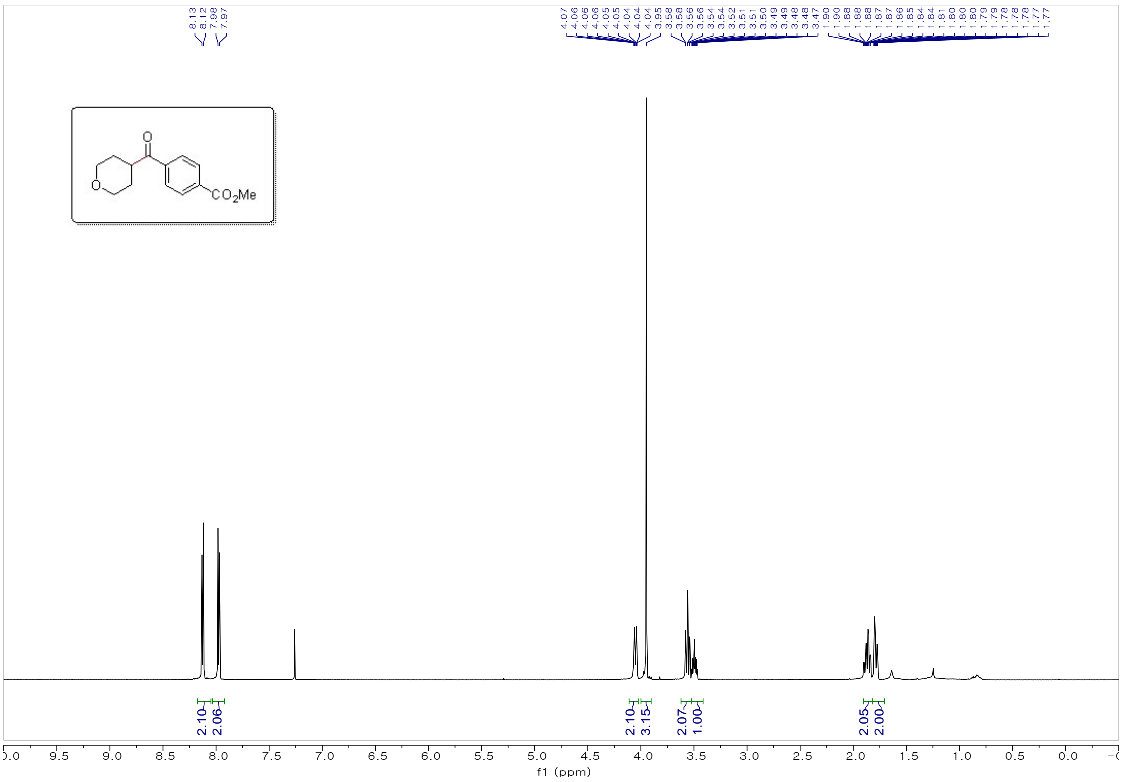

150 MHz, ^{13}C NMR in Chloroform-*d*

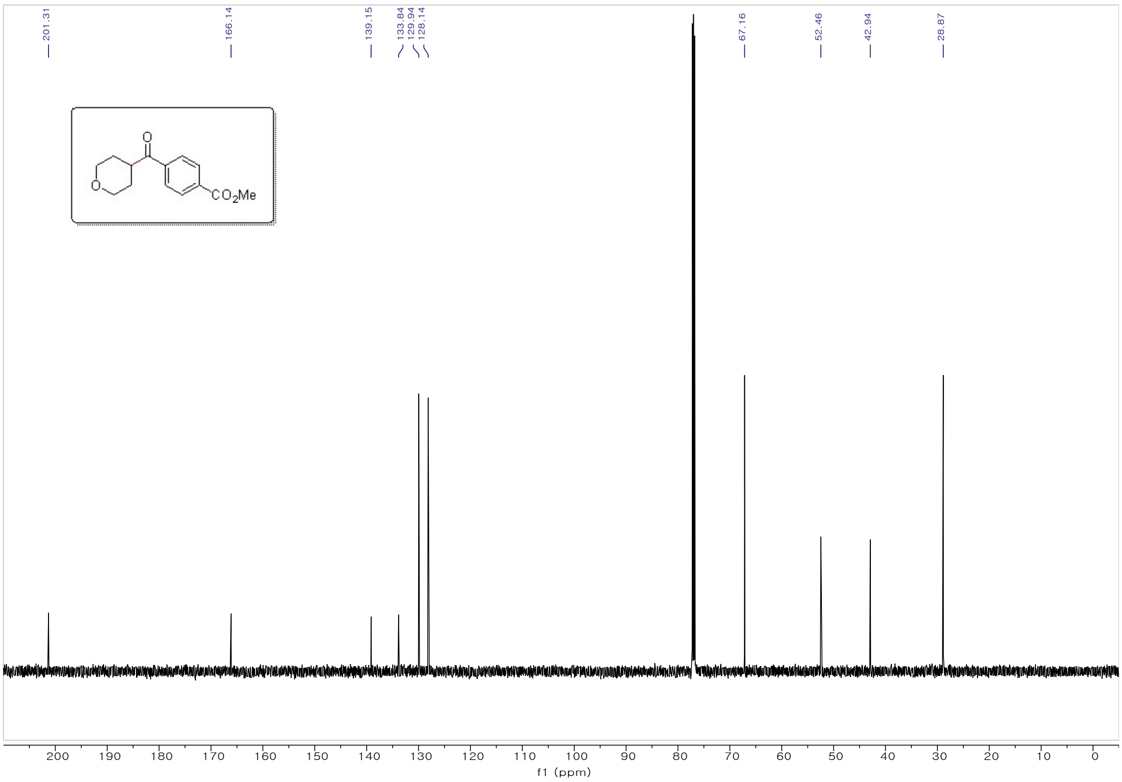

methyl 4-(2,3-dihydro-1H-indene-2-carbonyl)benzoate (3r).

600 MHz, ^{1}H NMR in Chloroform-*d*

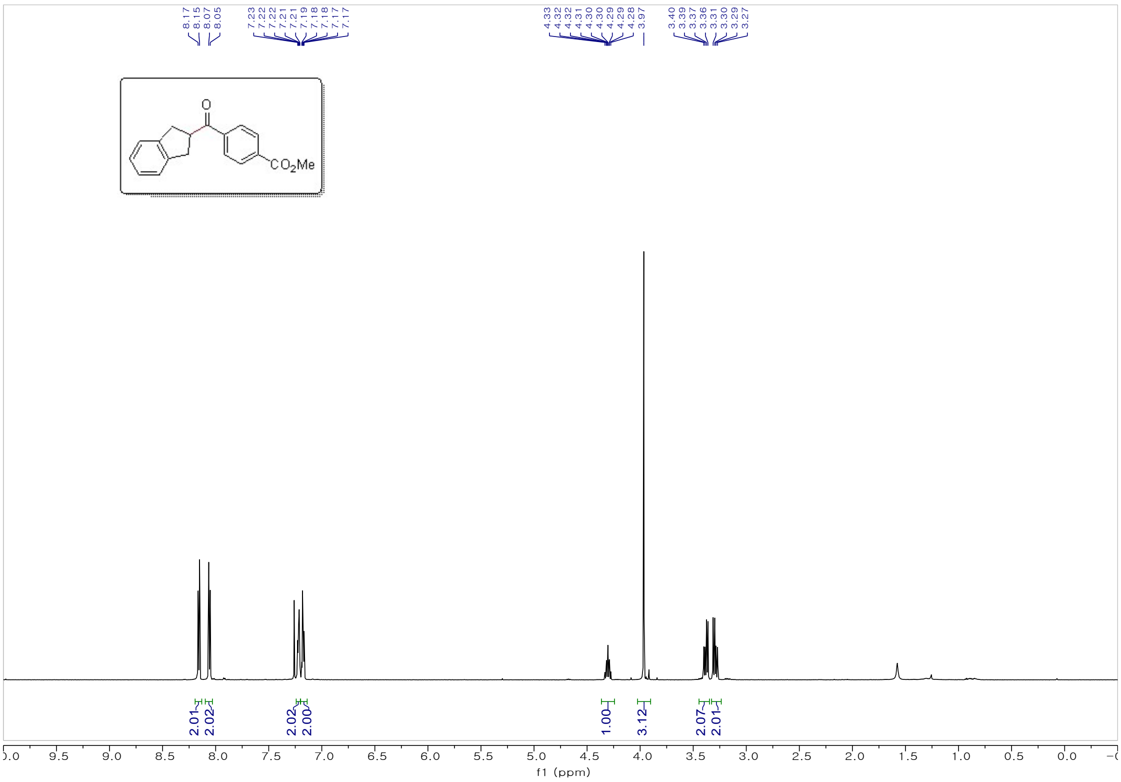

150 MHz, ^{13}C NMR in Chloroform-*d*

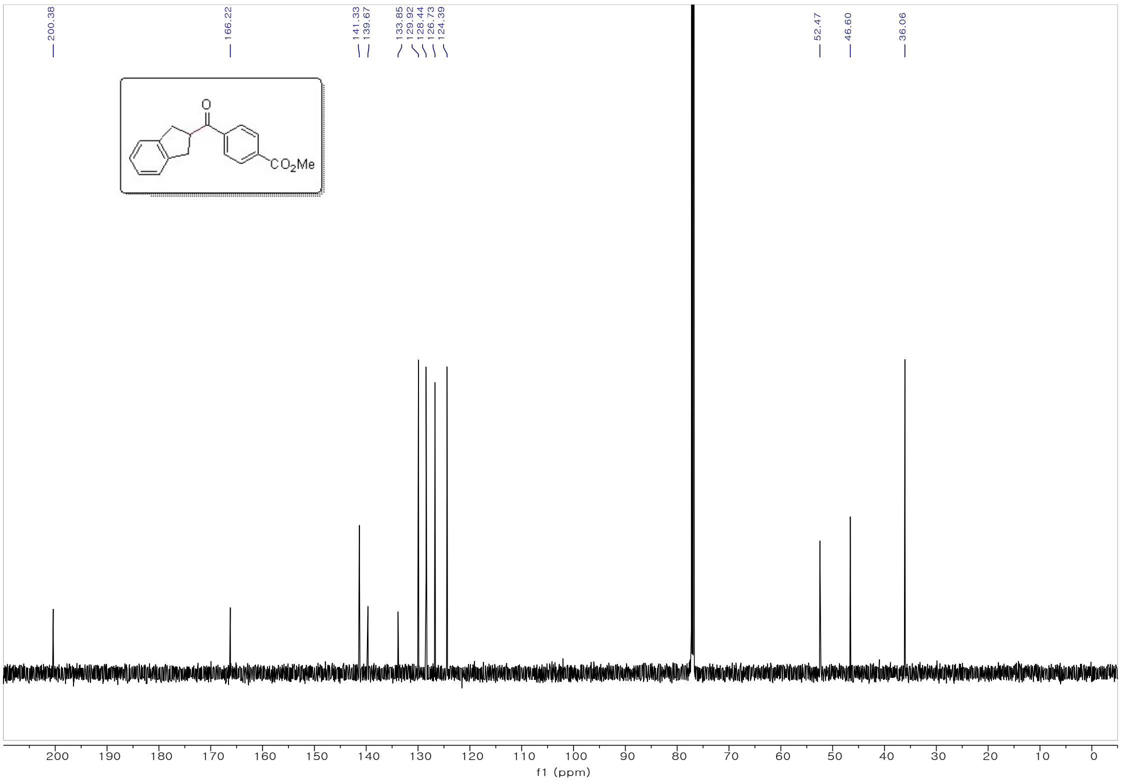

methyl 4-(2-(3,4-dimethoxybenzyl)-3-methoxy-3-oxopropanoyl)benzoate (3s).

600 MHz, ^{1}H NMR in Chloroform-*d*

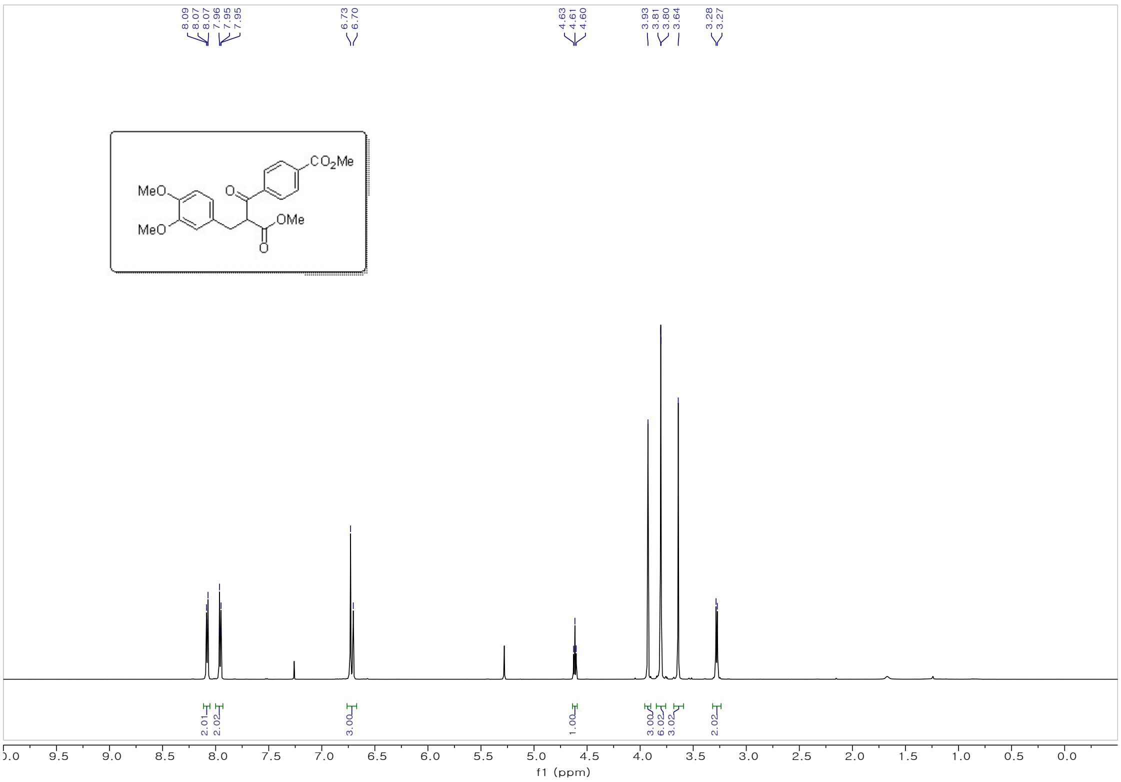

150 MHz, ^{13}C NMR in Chloroform-*d*

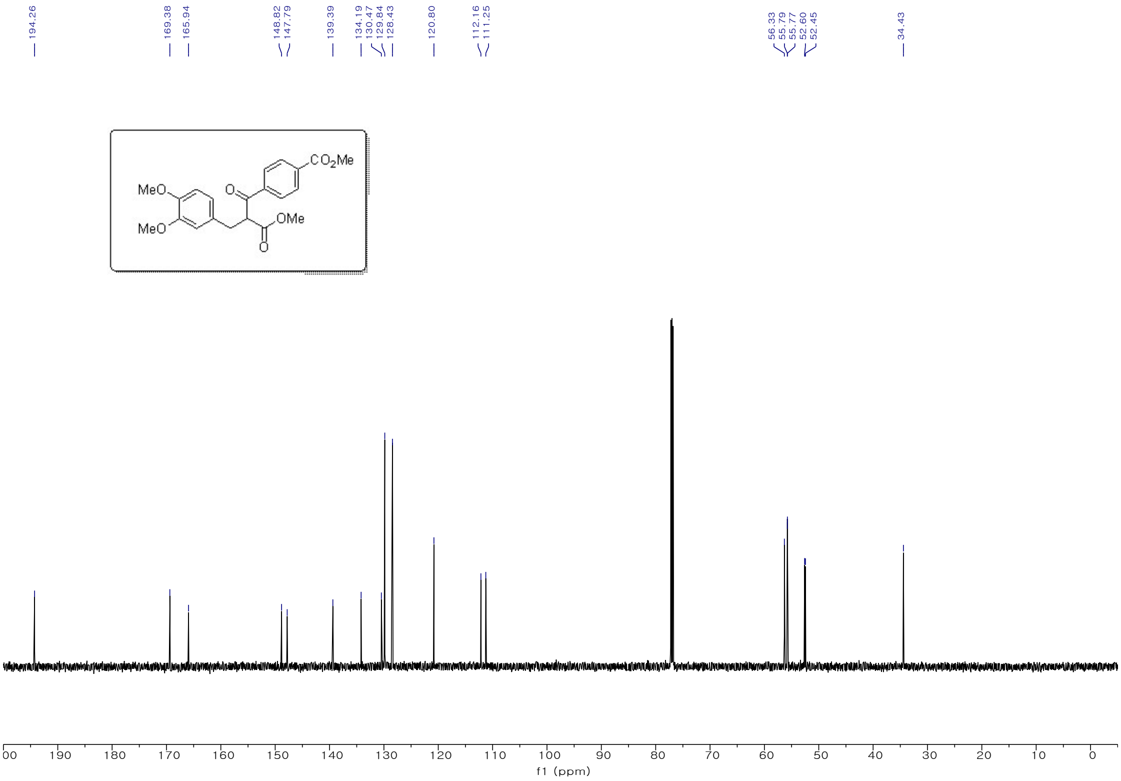

methyl 4-((5R,6R)-6-acetamido-3-(ethoxycarbonyl)-5-(pentan-3-yloxy)cyclohex-3-ene-1-carbonyl)benzoate (3t).

600 MHz, ^{1}H NMR in Chloroform-*d*

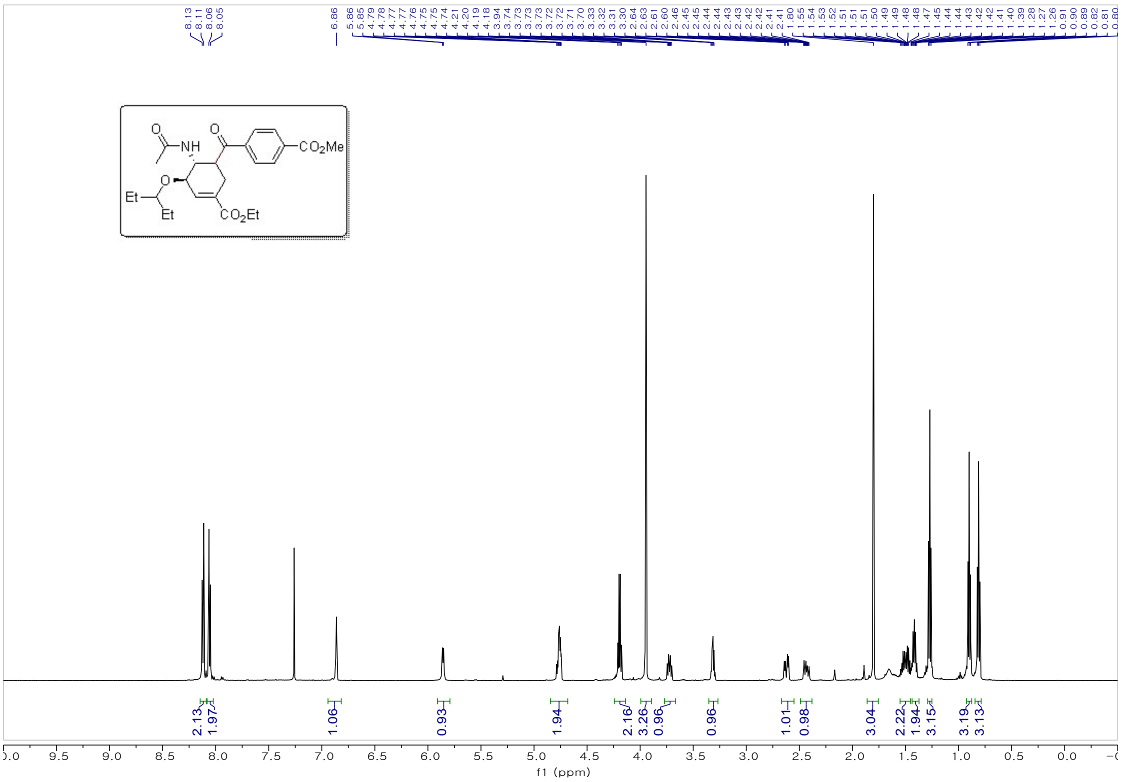

150 MHz, ^{13}C NMR in Chloroform-*d*

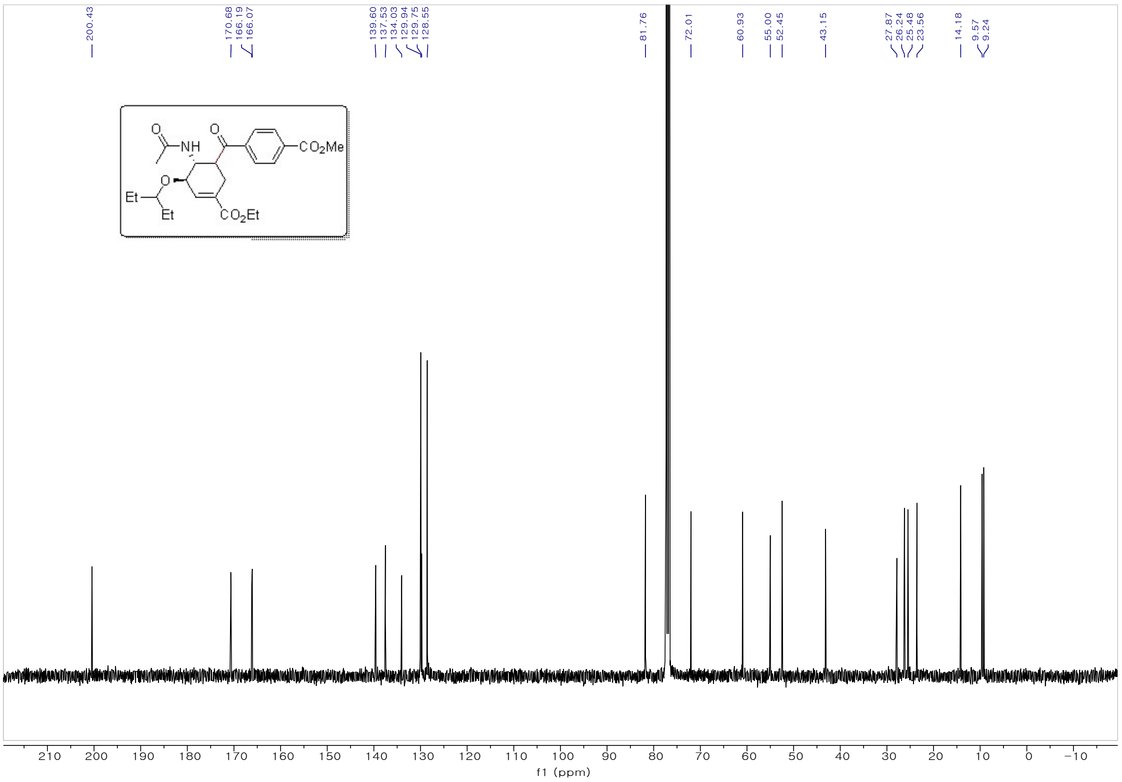

methyl (2-benzyl-3-oxo-3-phenylpropanoyl)alaninate (3u).

600 MHz, ^{1}H NMR in Chloroform-*d*

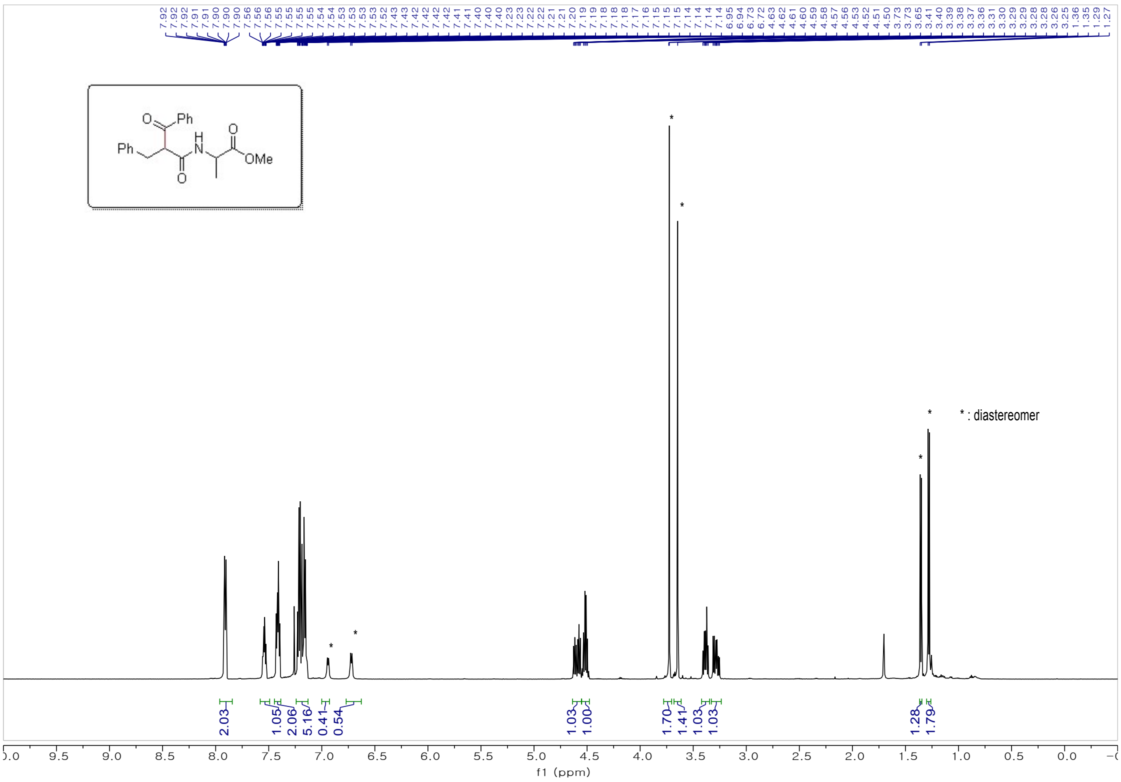

150 MHz, ^{13}C NMR in Chloroform-*d*

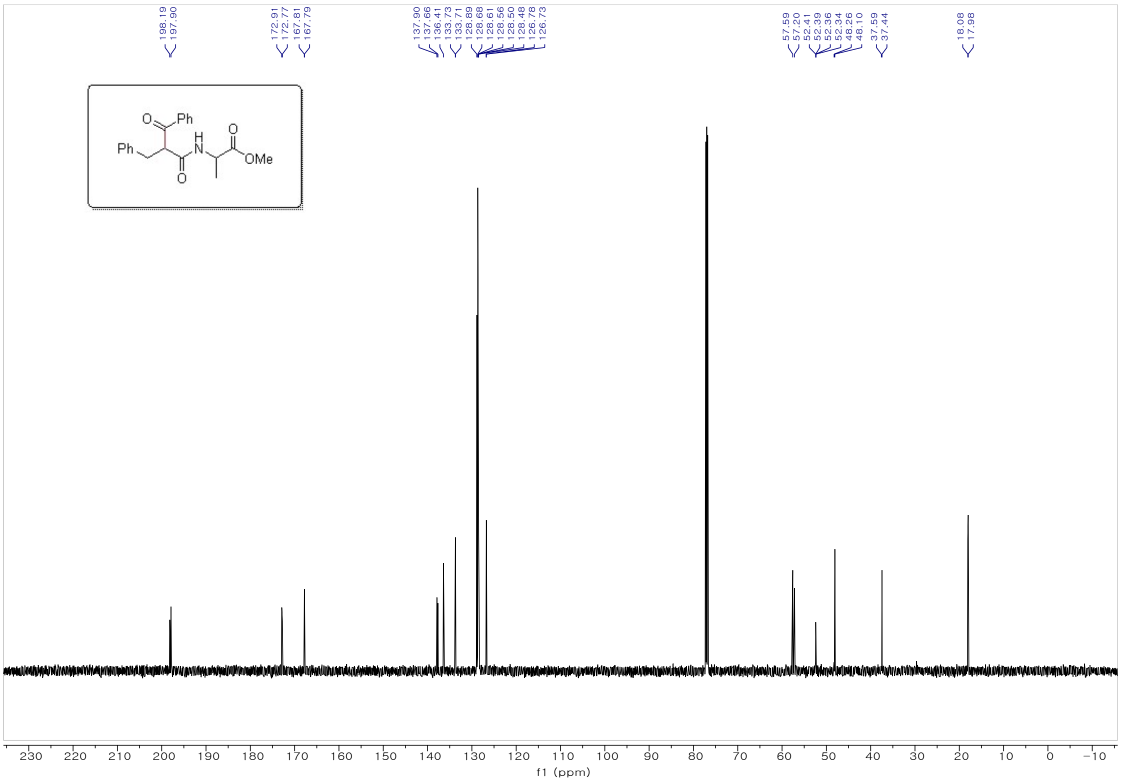

methyl (2-benzyl-3-oxo-3-phenylpropanoyl)phenylalaninate (3v).

600 MHz, ^{1}H NMR in Chloroform-*d*

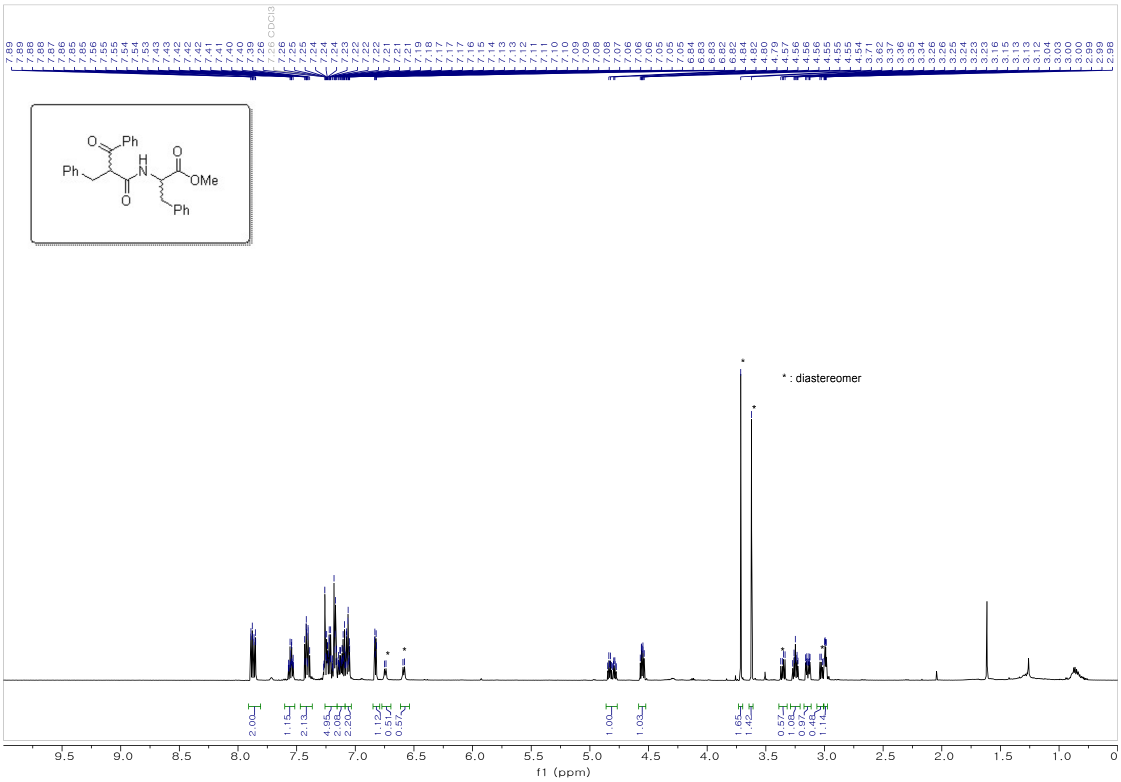

150 MHz, ^{13}C NMR in Chloroform-*d*

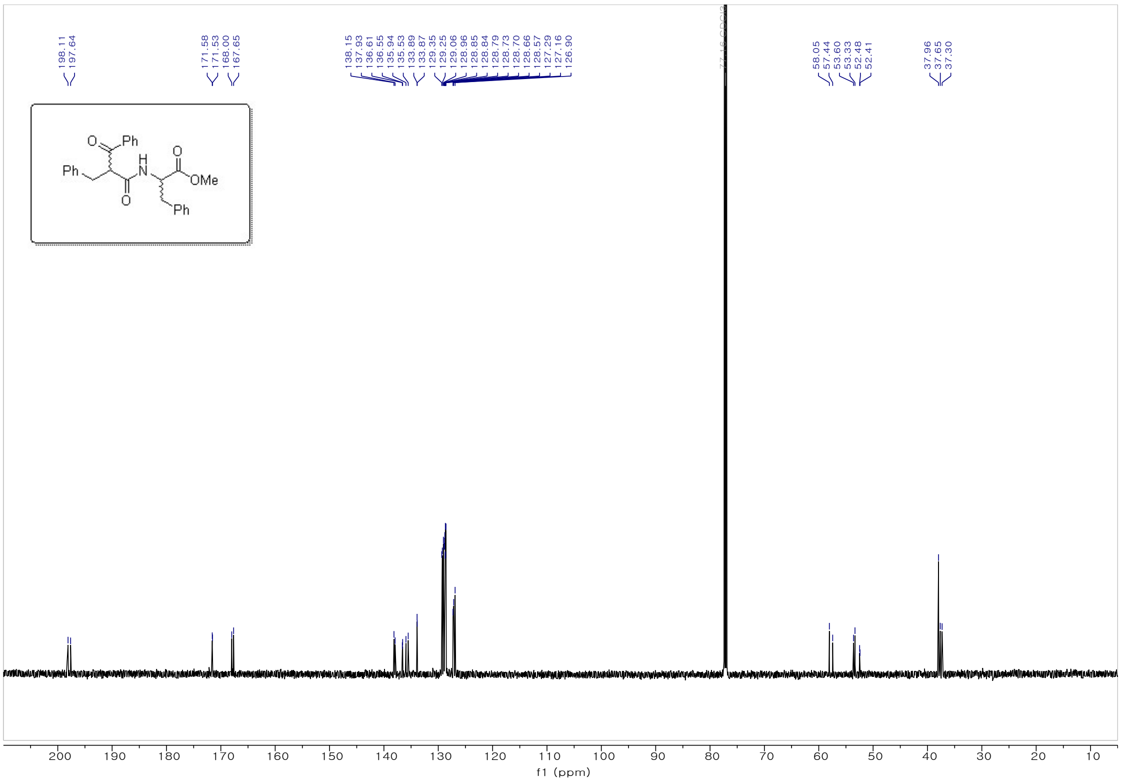

methyl (2-benzoyl-4-(methylthio)butanoyl)glycinate (3w).

600 MHz, ^{1}H NMR in Chloroform-*d*

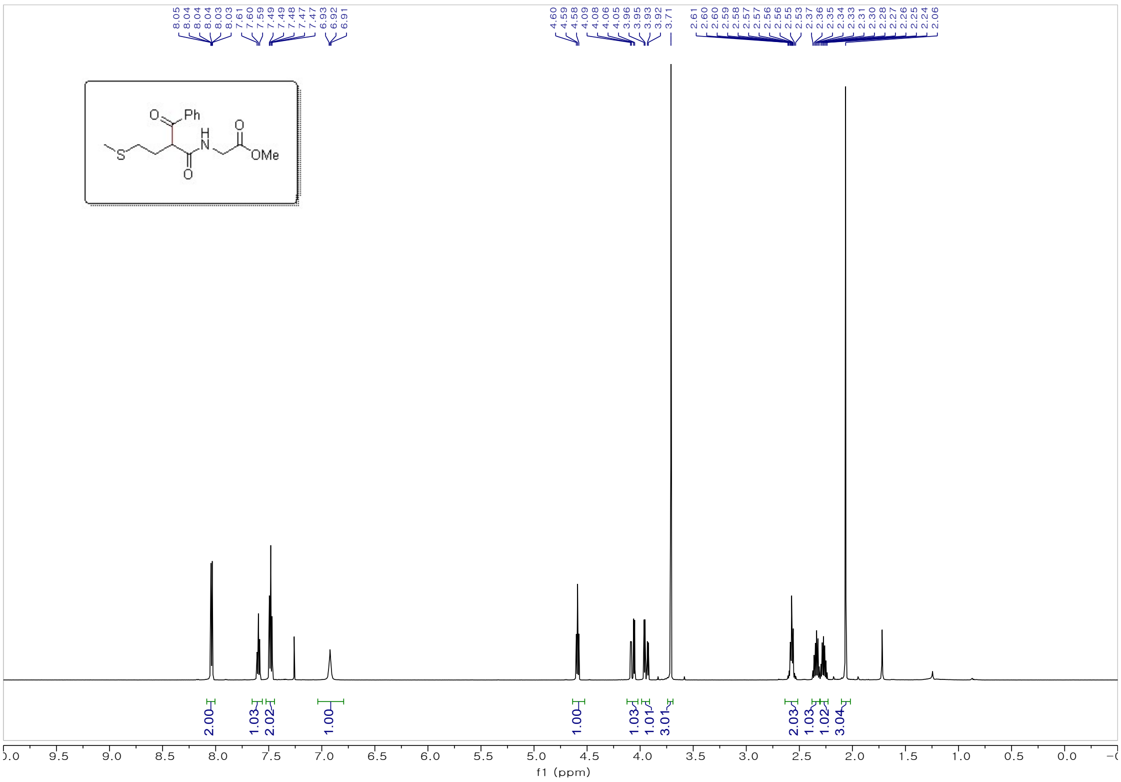

150 MHz, ^{13}C NMR in Chloroform-*d*

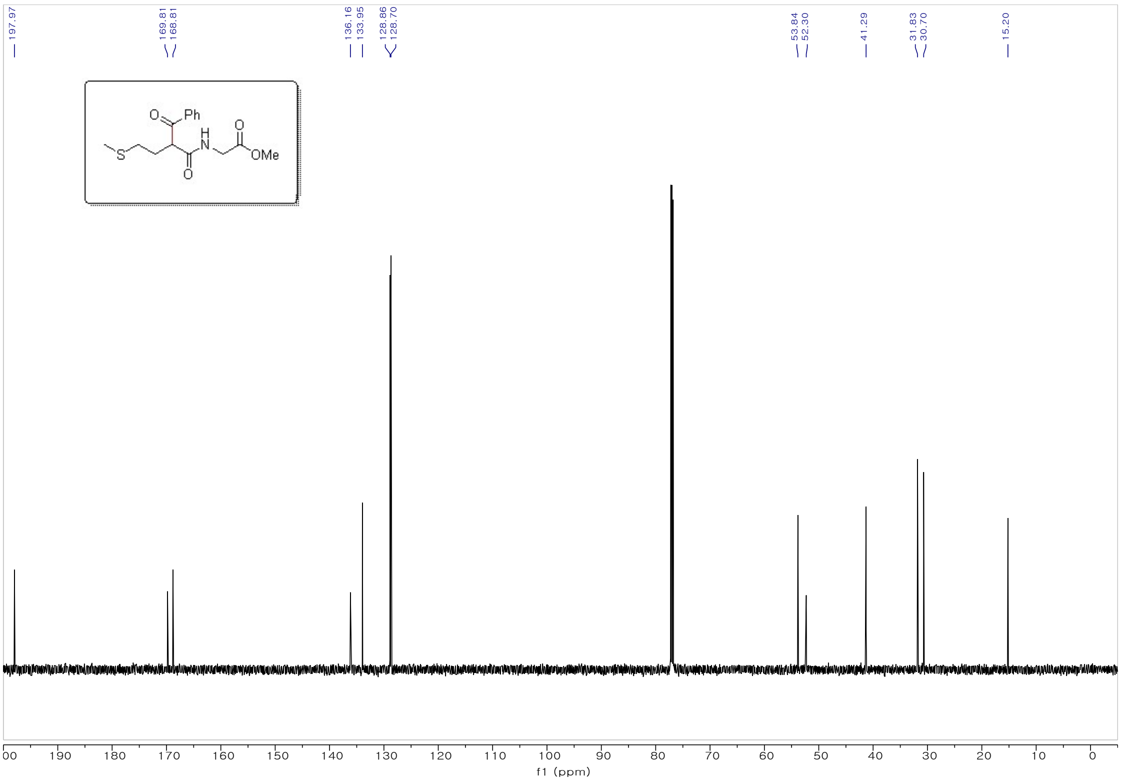

methyl (2-benzoyl-4-methylpentanoyl)glycinate (3x).

600 MHz, ^{1}H NMR in Chloroform-*d*

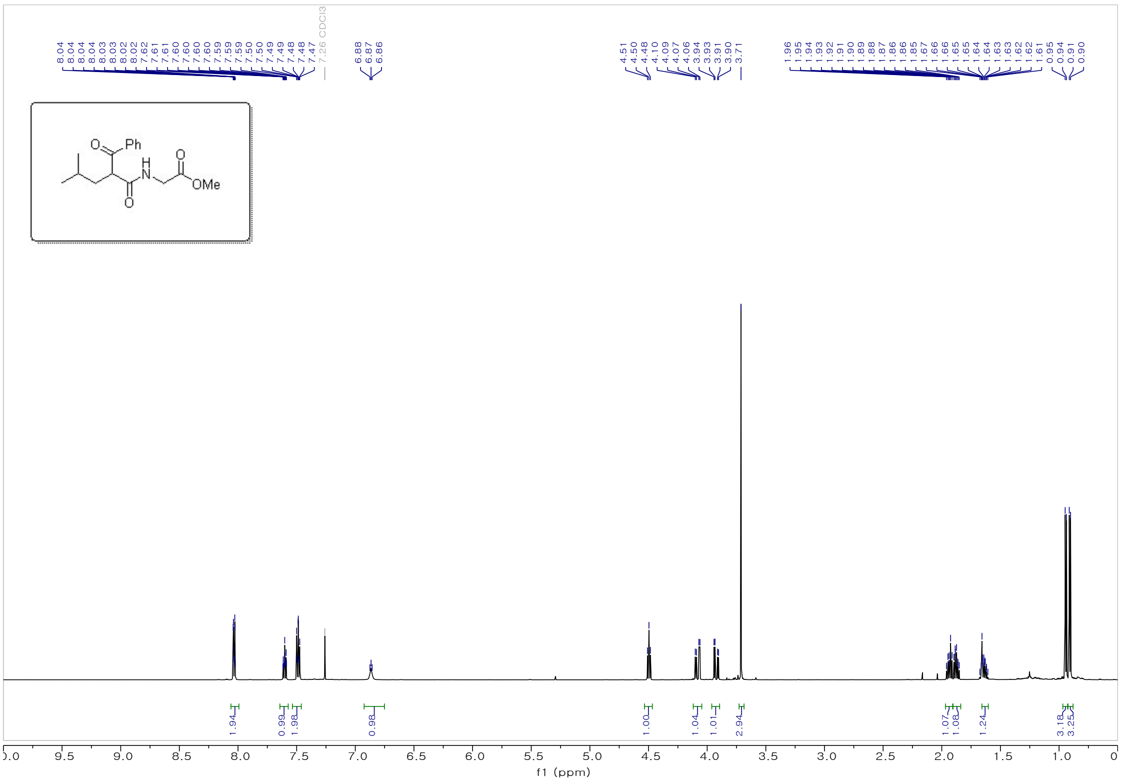

150 MHz, ^{13}C NMR in Chloroform-*d*

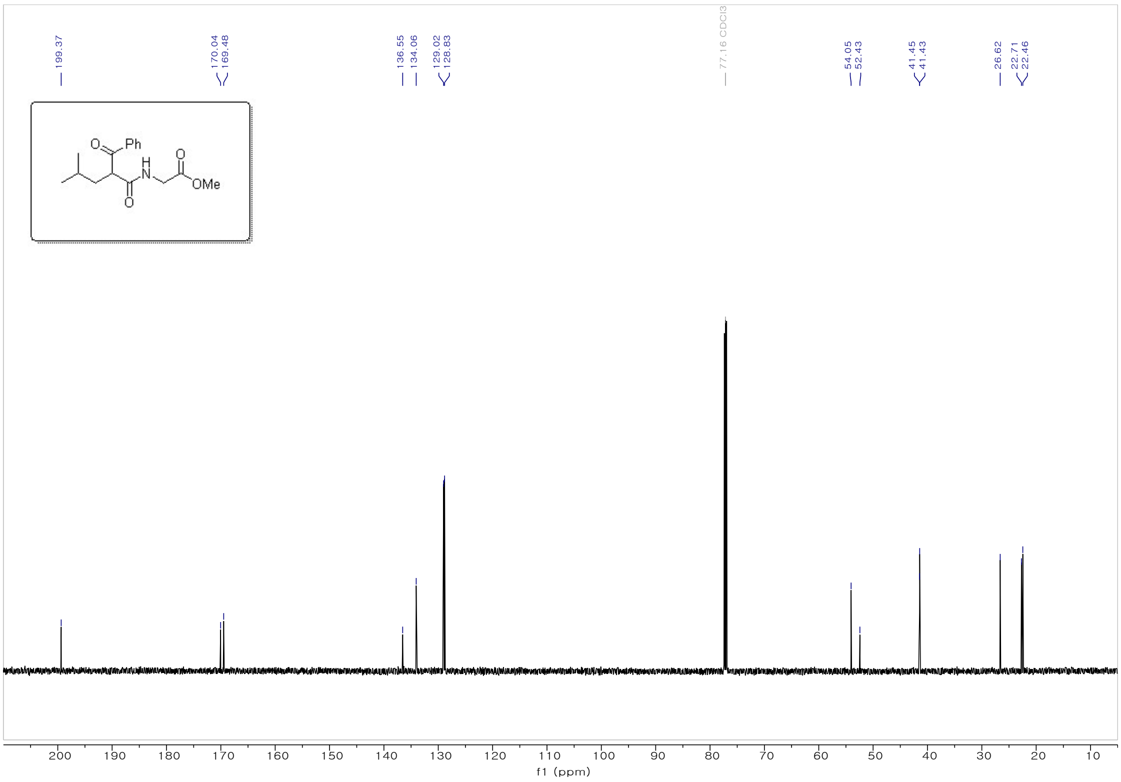

methyl (2-methyl-3-oxo-3-phenylpropanoyl)phenylalaninate (3y).

600 MHz, ^{1}H NMR in Chloroform-*d*

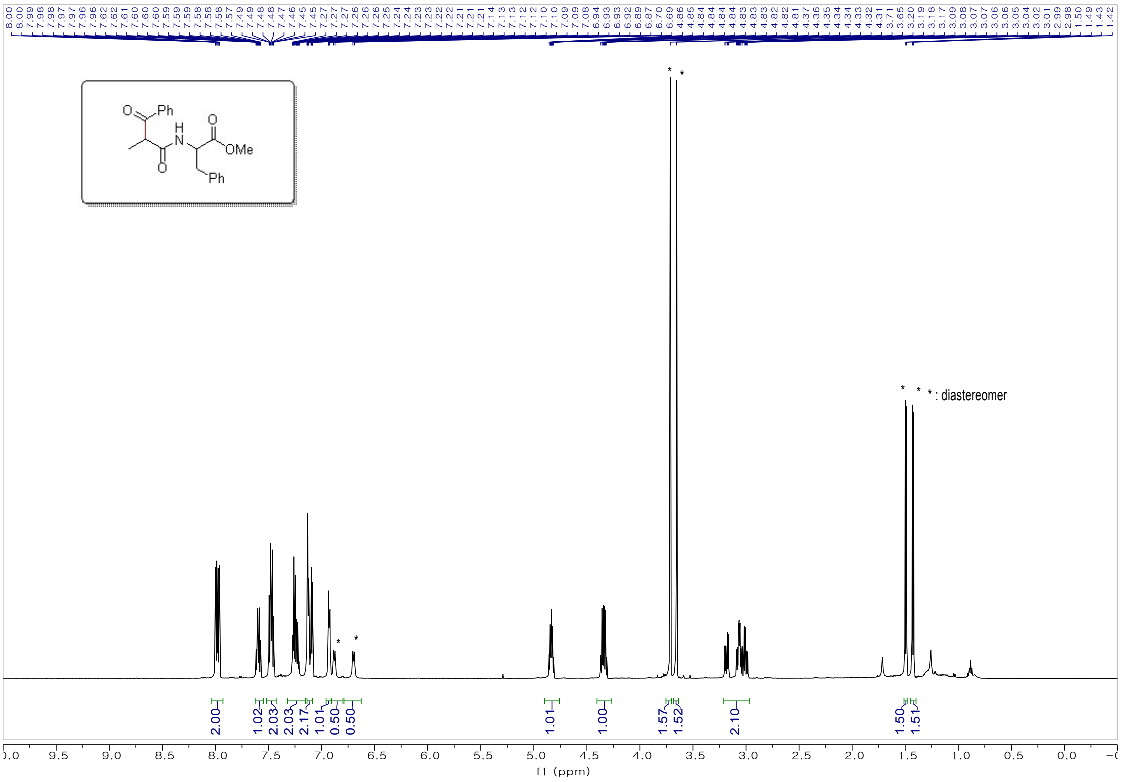

150 MHz, ^{13}C NMR in Chloroform-*d*

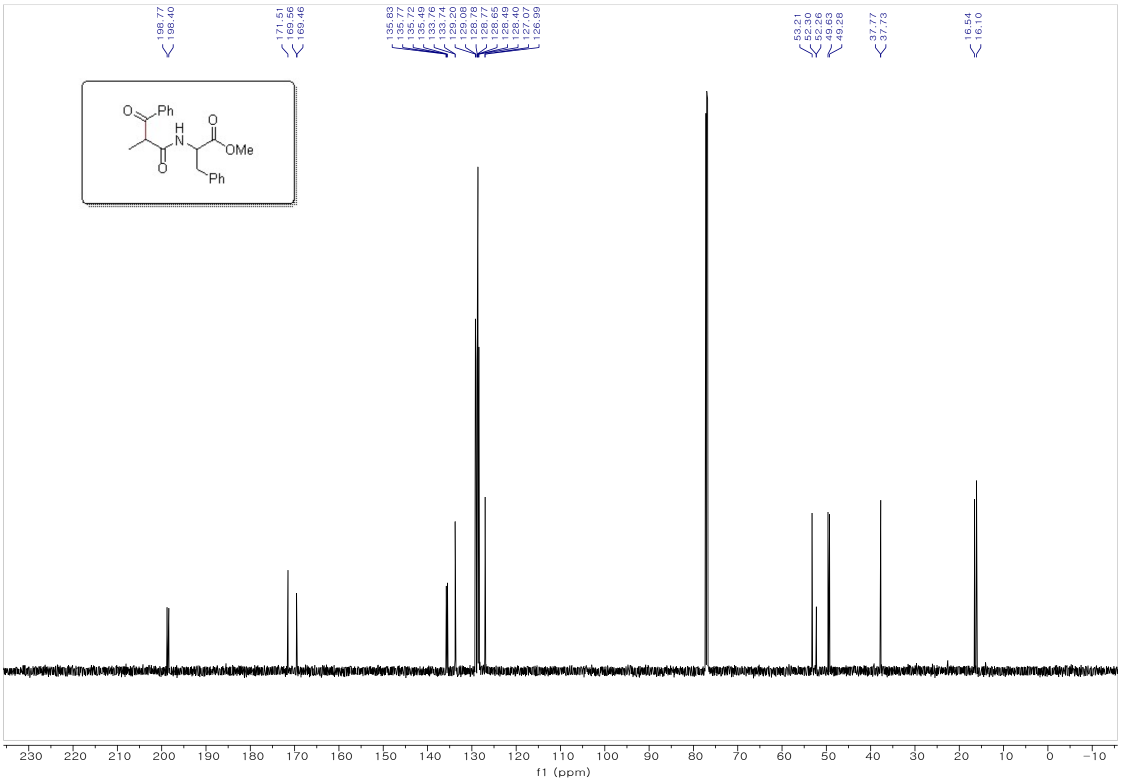

methyl (2-benzoyl-4-methylpentanoyl)glycylglycinate (3z).

600 MHz, ^{1}H NMR in Chloroform-*d*

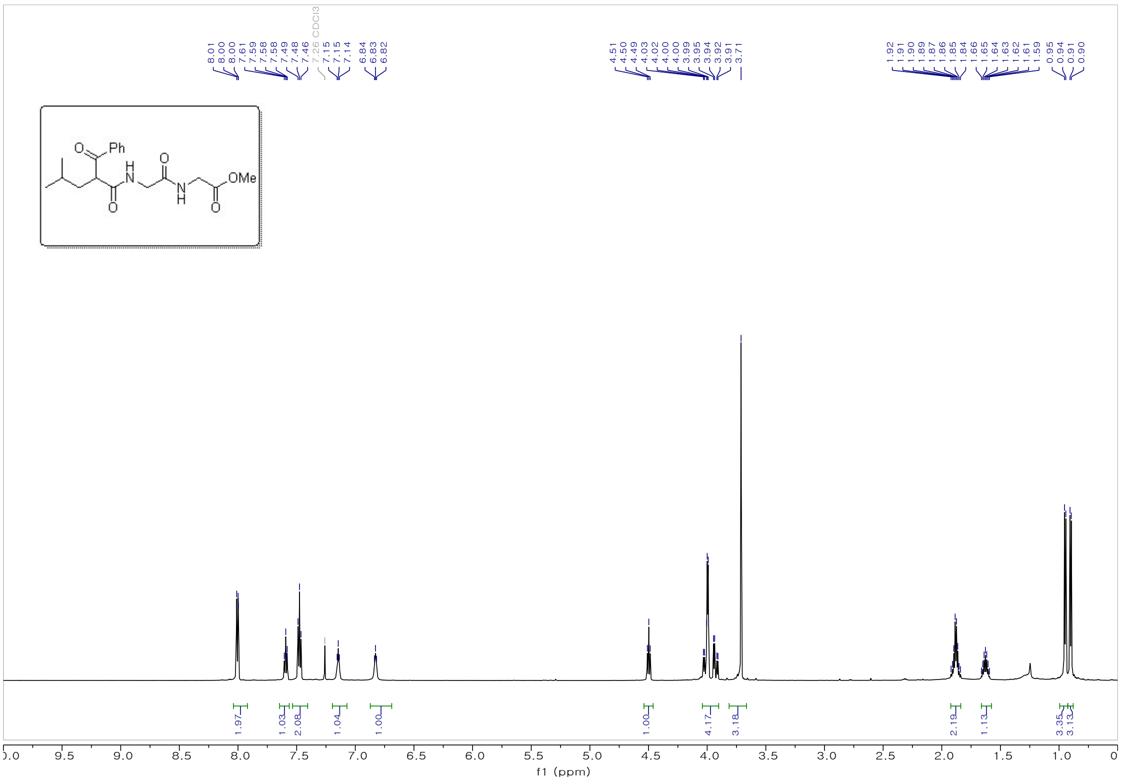

150 MHz, ^{13}C NMR in Chloroform-*d*

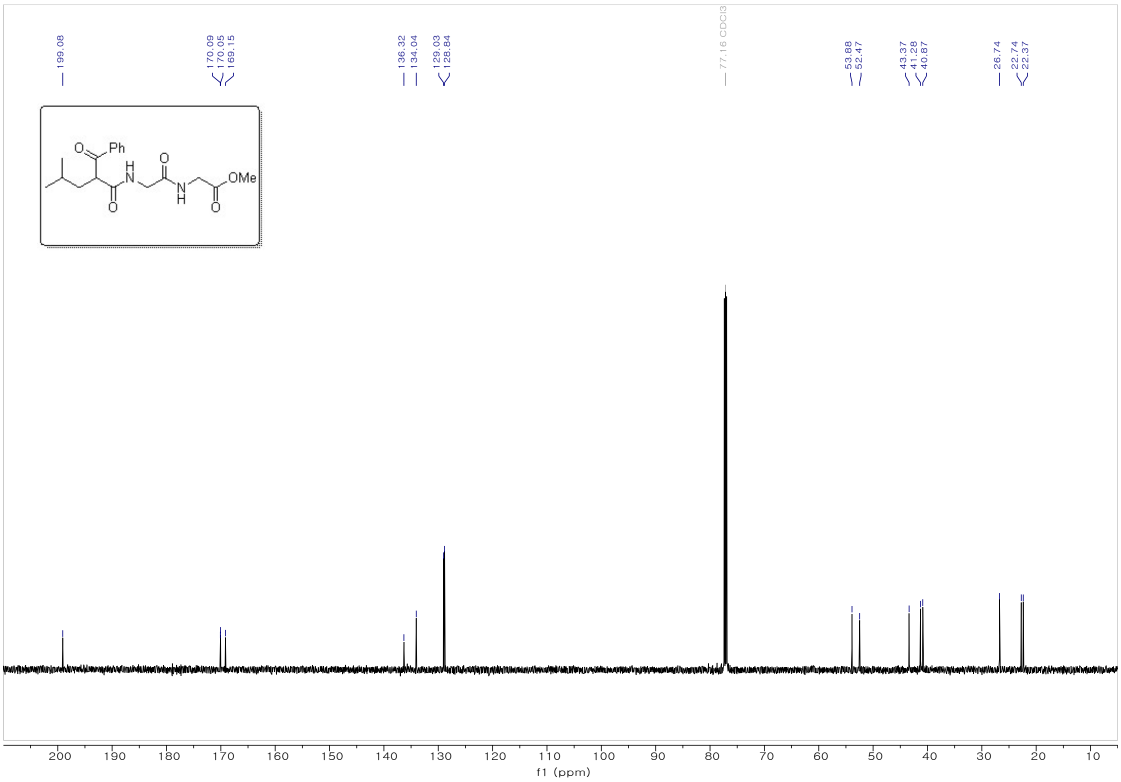

methyl (2-benzyl-3-oxo-3-phenylpropanoyl)glycylglycinate (3aa).

600 MHz, ^{1}H NMR in Chloroform-*d*

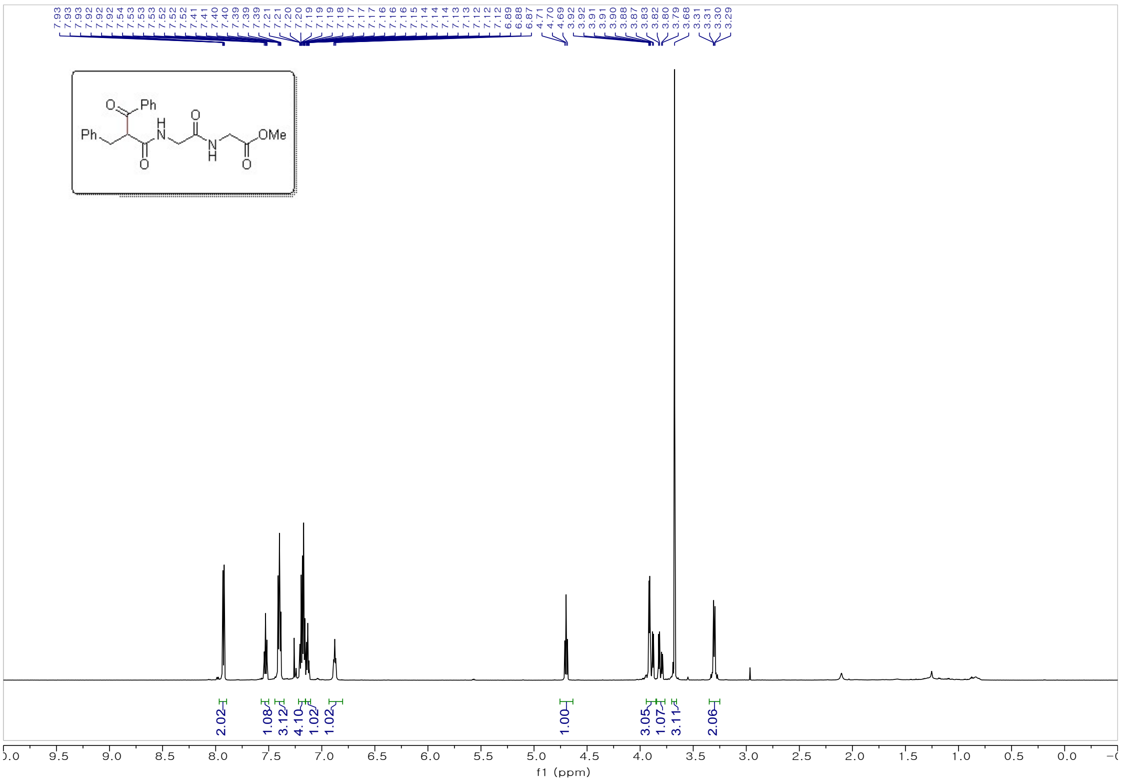

150 MHz, ^{13}C NMR in Chloroform-*d*

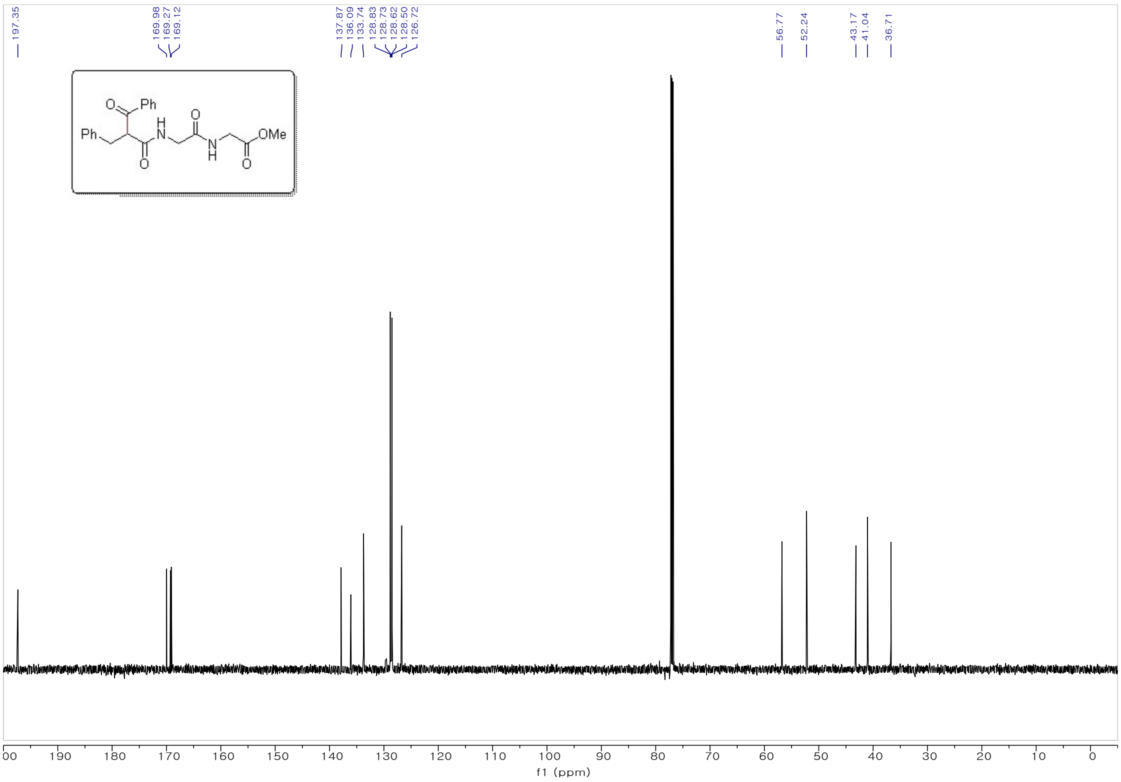

methyl (2-benzyl-3z-oxo-3-phenylpropanoyl)alanylleucylglycinate (3ab).

600 MHz, ^{1}H NMR in Chloroform-*d*

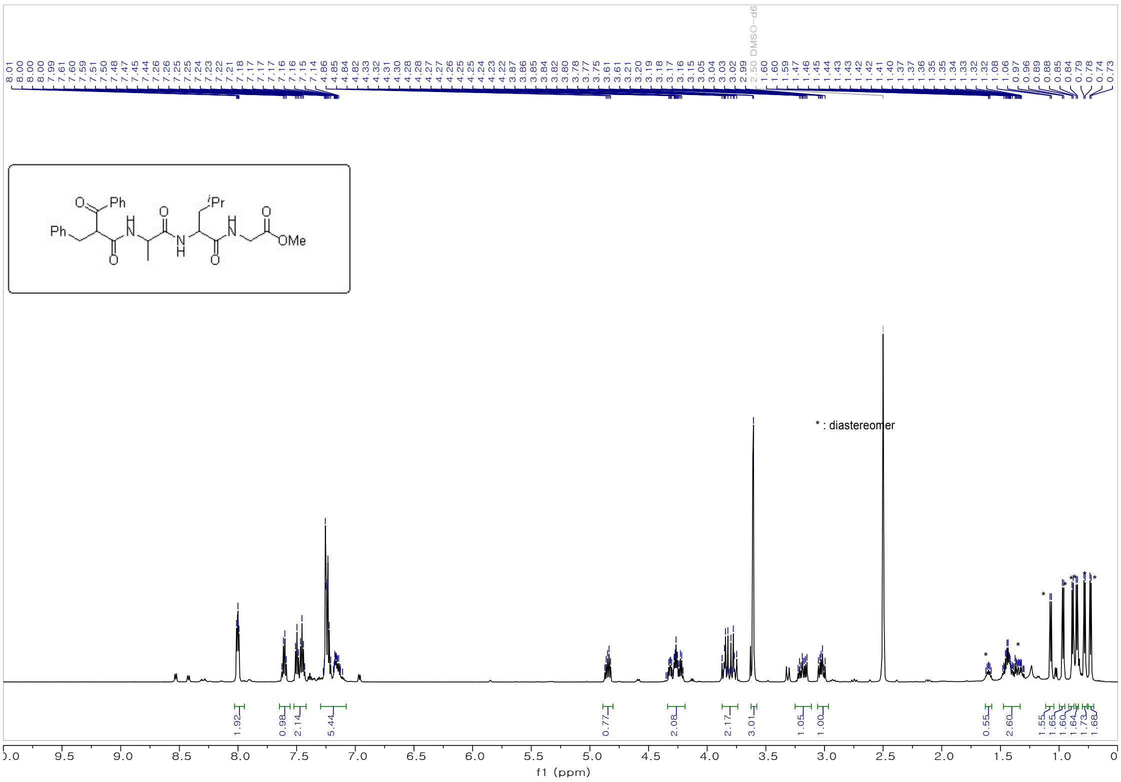

150 MHz, ^{13}C NMR in Chloroform-*d*

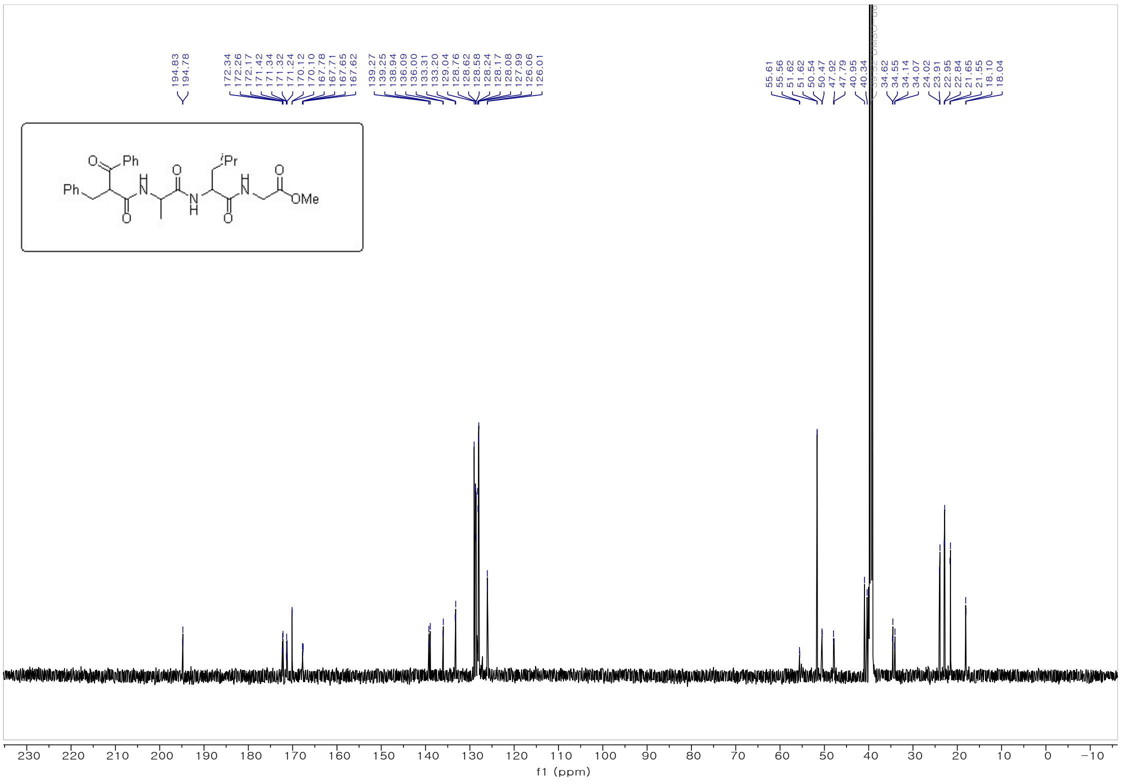

methyl 4-benzoyl-5-((2-((1-((2-methoxy-2-oxoethyl)amino)-1-oxo-3-phenylpropan-2-yl)amino)-2-oxoethyl)amino)-5-oxopentanoate (3ac).

600 MHz, ^{1}H NMR in Chloroform-*d*

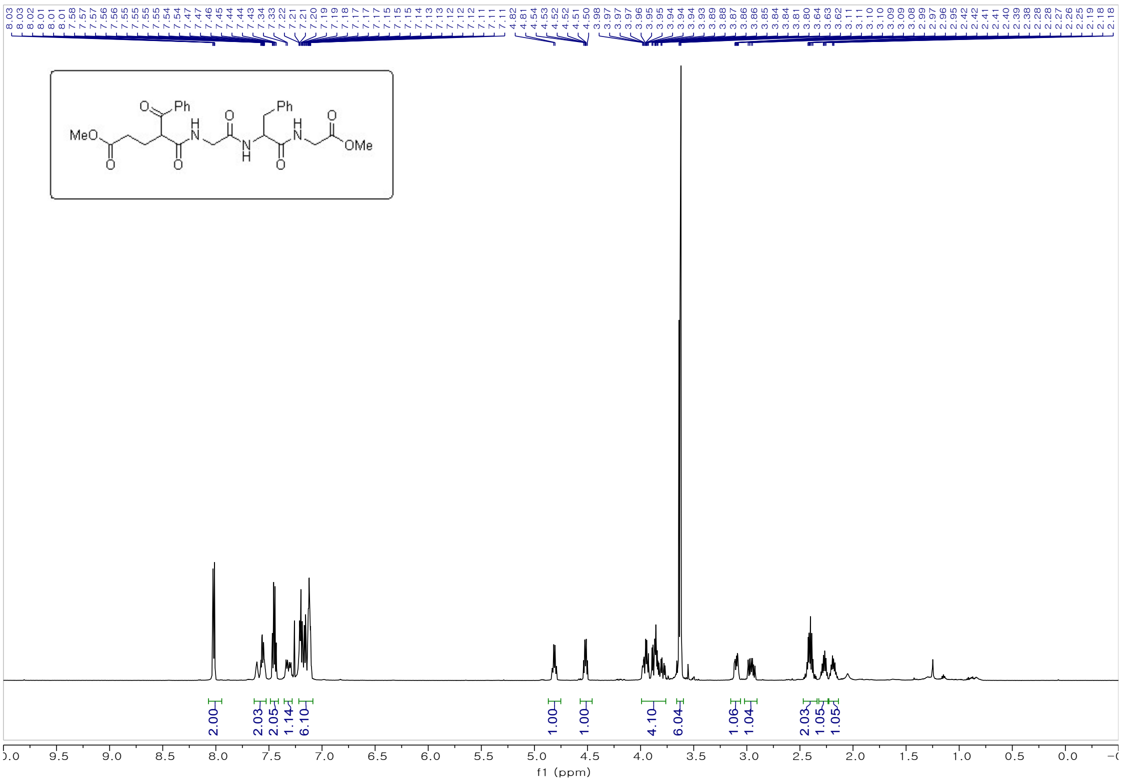

150 MHz, ^{13}C NMR in Chloroform-*d*

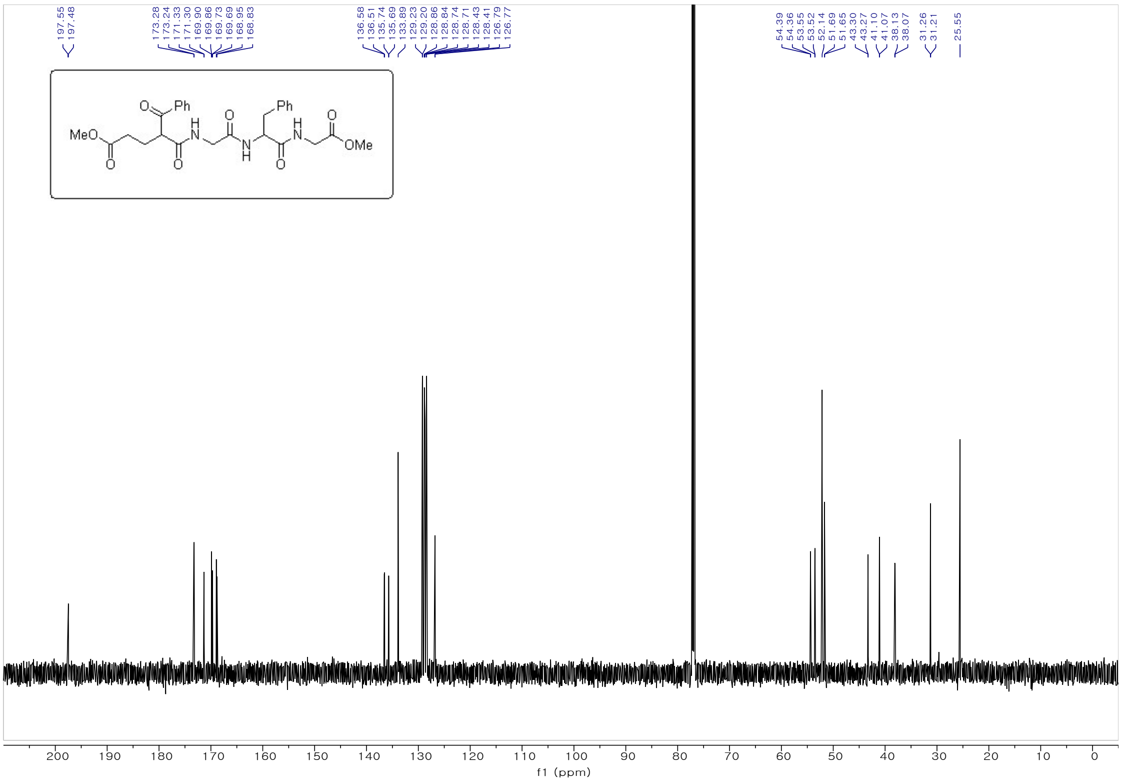

methyl 4-benzoyl-5-((1-((2-((1-methoxy-1-oxopropan-2-yl)amino)-2-oxoethyl)amino)-1-oxo-3-phenylpropan-2-yl)amino)-5-oxopentanoate (3ad).

600 MHz, ^{1}H NMR in Chloroform-*d*

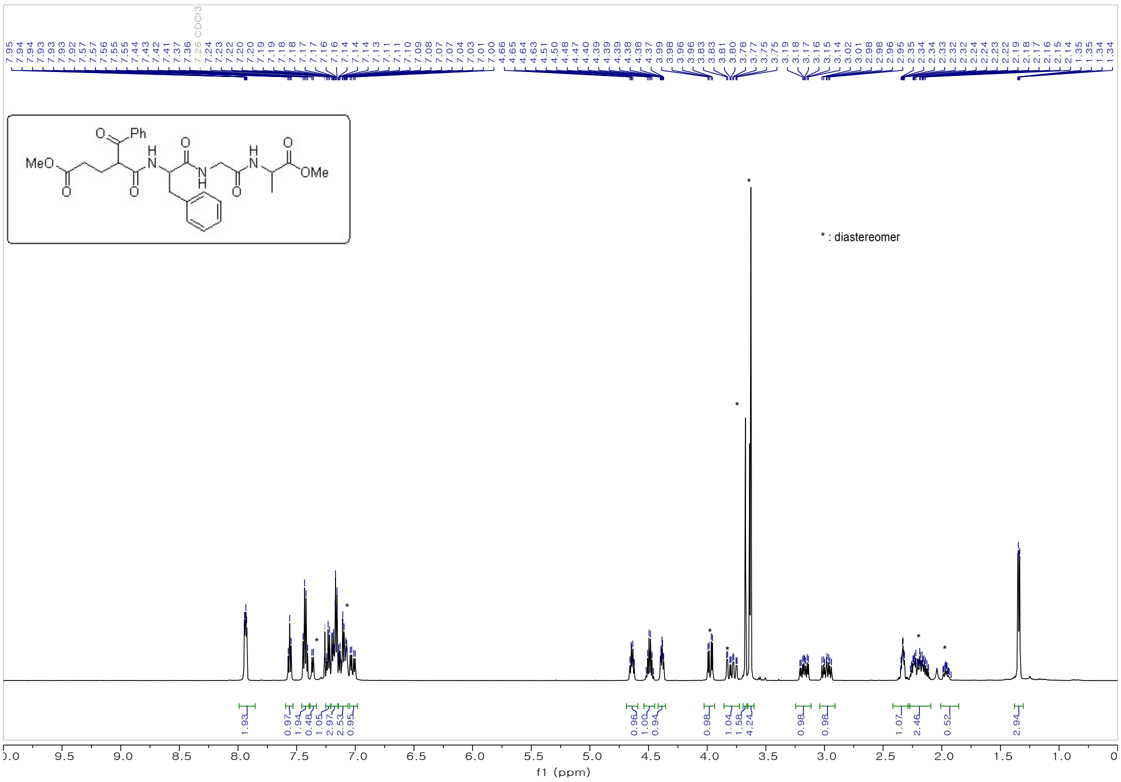

150 MHz, ^{13}C NMR in Chloroform-*d*

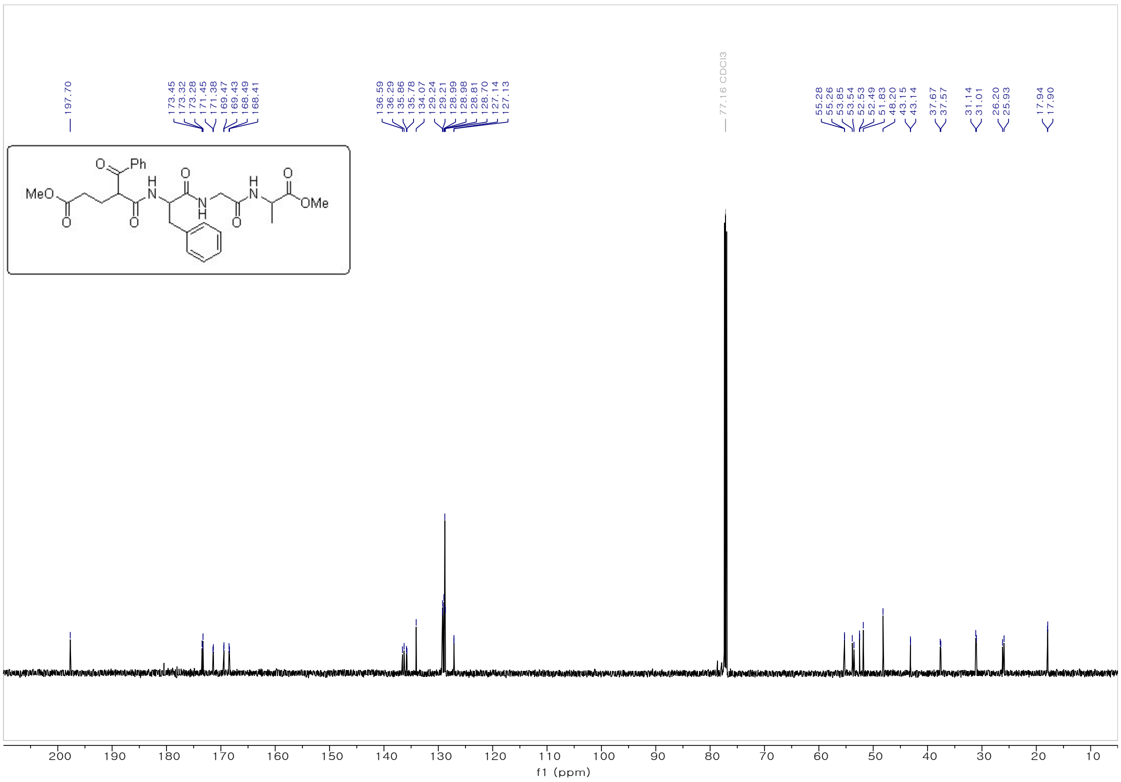

dimethyl 2-(4-methylbenzoyl)pentanedioate (3ae).

400 MHz, ^{1}H NMR in Chloroform-*d*

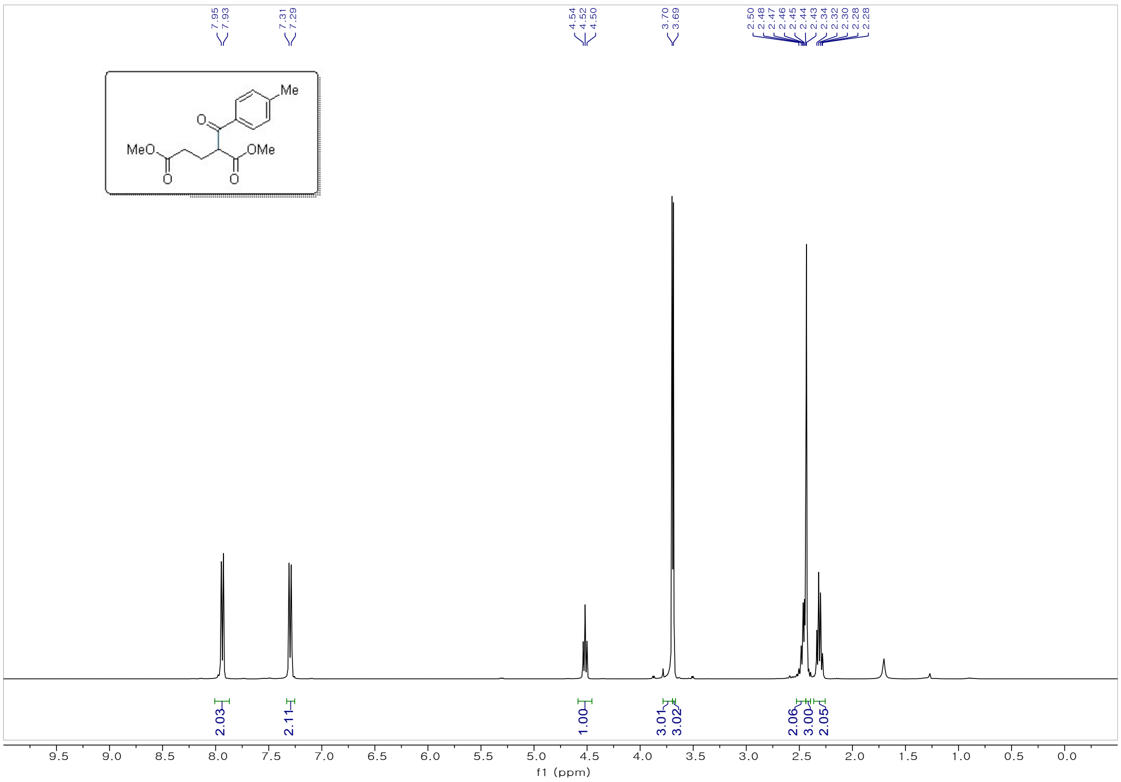

100 MHz, ^{13}C NMR in Chloroform-*d*

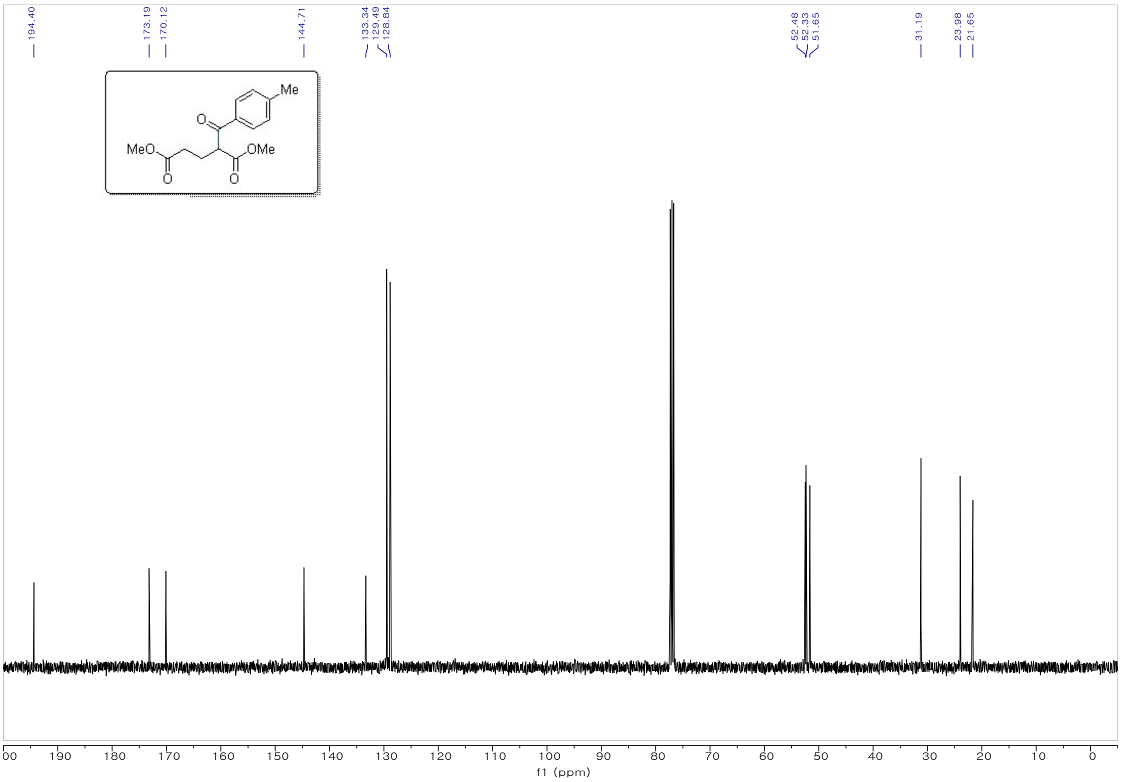

dimethyl 2-(4-(methylthio)benzoyl)pentanedioate (3af).

400 MHz, ^{1}H NMR in Chloroform-*d*

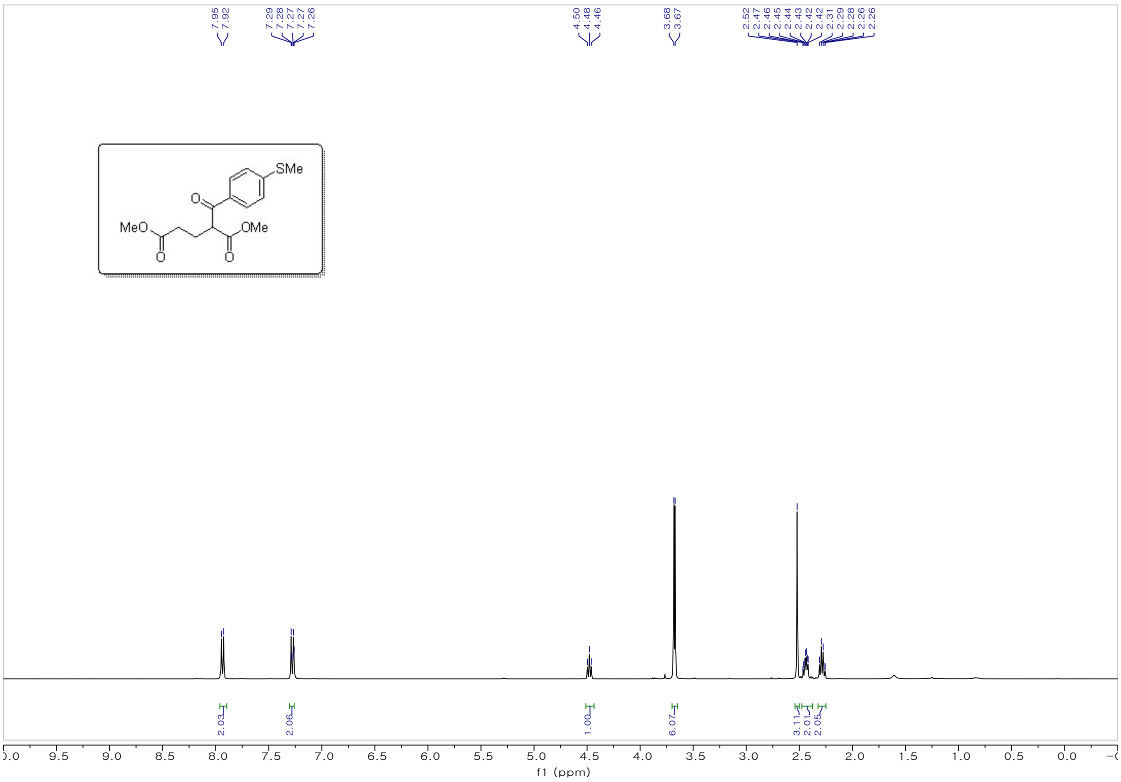

100 MHz, ^{13}C NMR in Chloroform-*d*

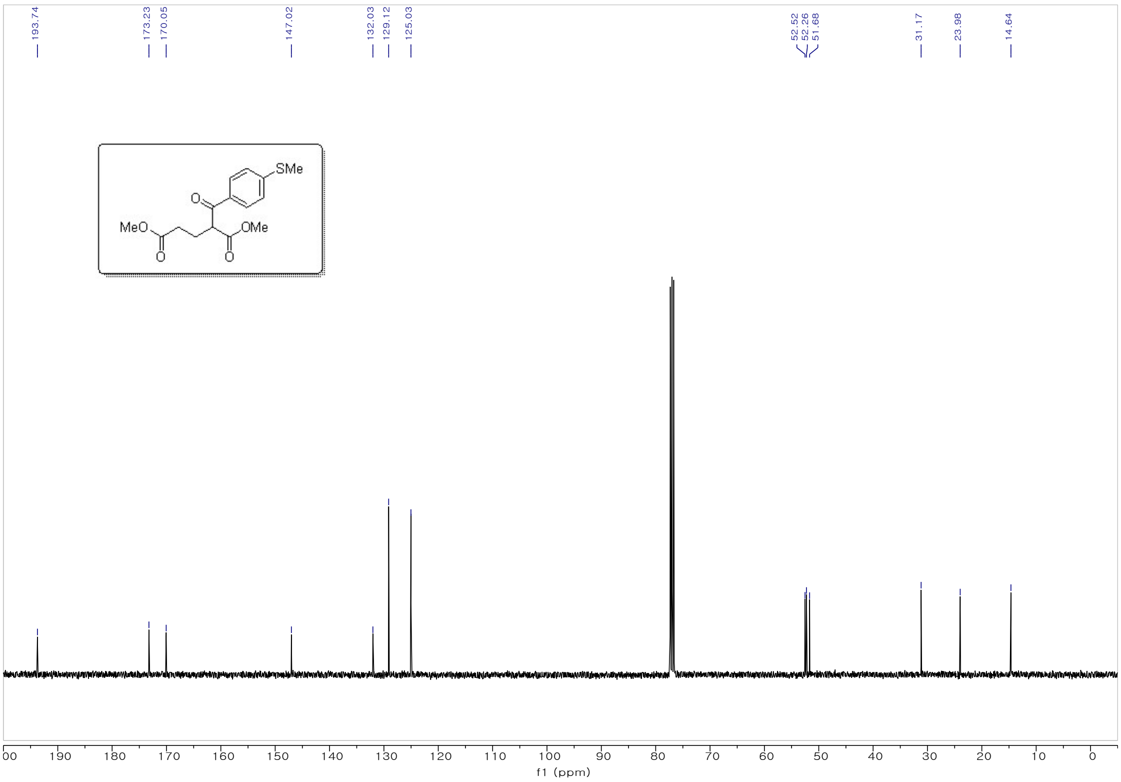

dimethyl 2-(4-methoxybenzoyl)pentanedioate (3ag).

600 MHz, ^{1}H NMR in Chloroform-*d*

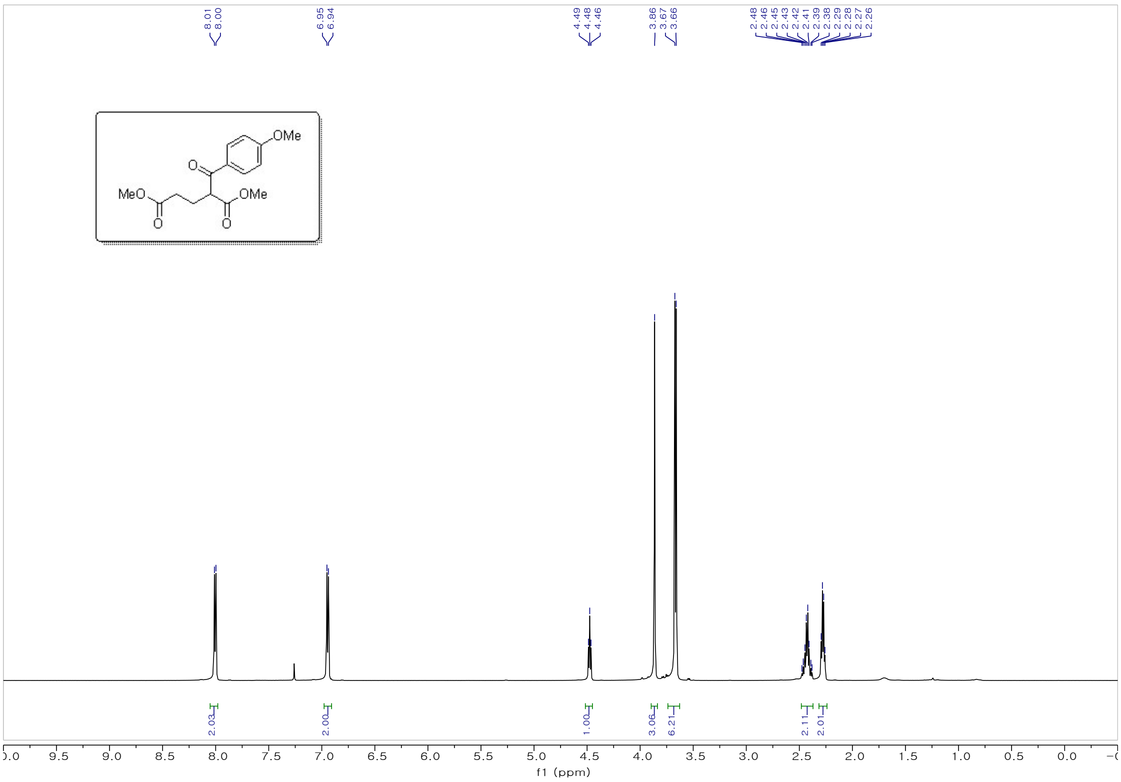

150 MHz, ^{13}C NMR in Chloroform-*d*

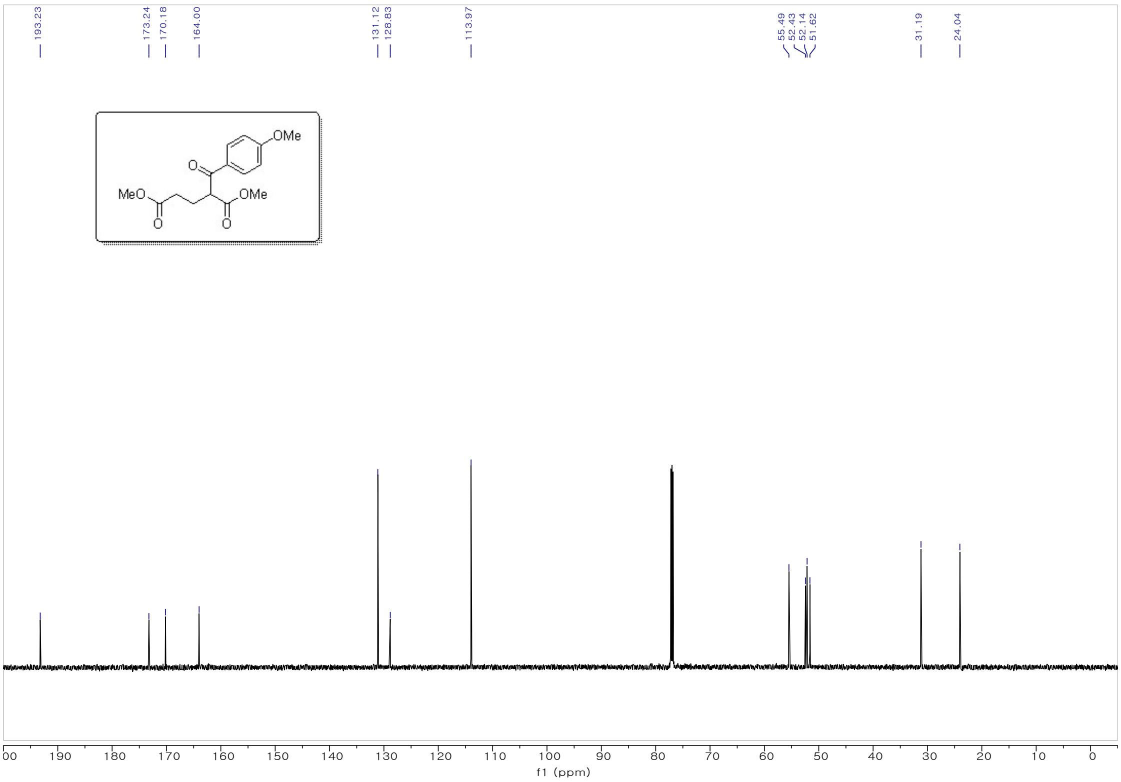

dimethyl 2-(4-cyanobenzoyl)pentanedioate (3ah).

400 MHz, ^{1}H NMR in Chloroform-*d*

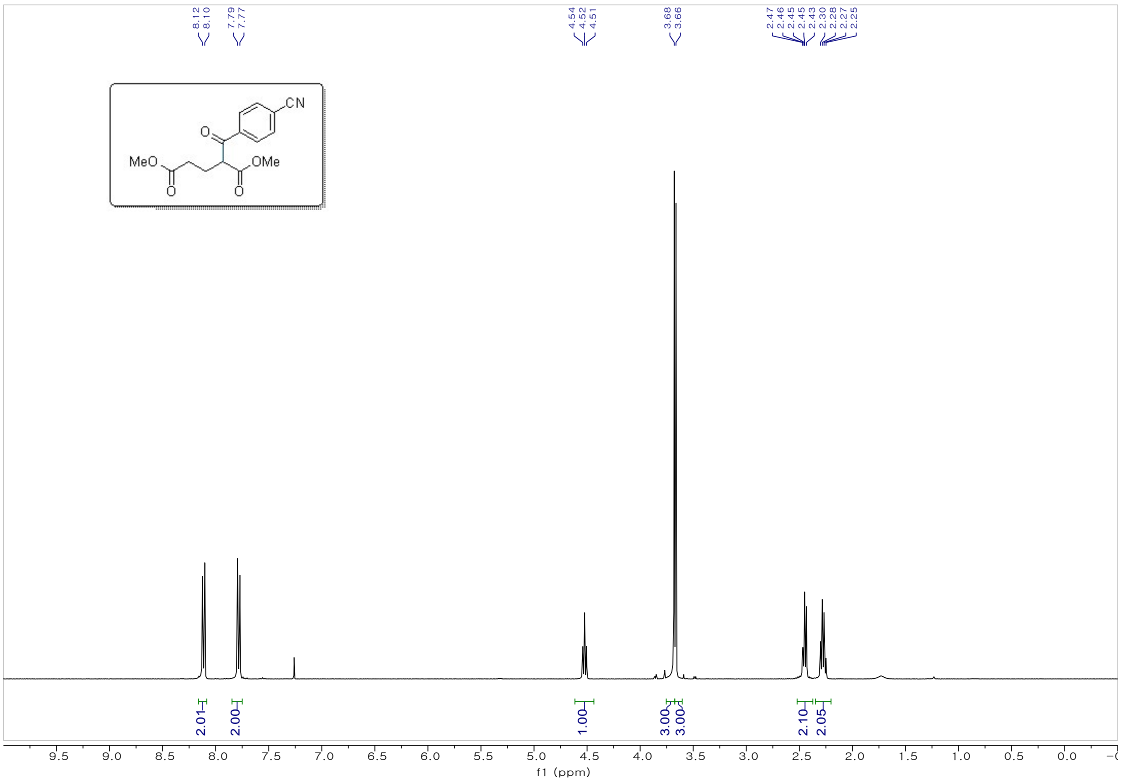

100 MHz, ^{13}C NMR in Chloroform-*d*

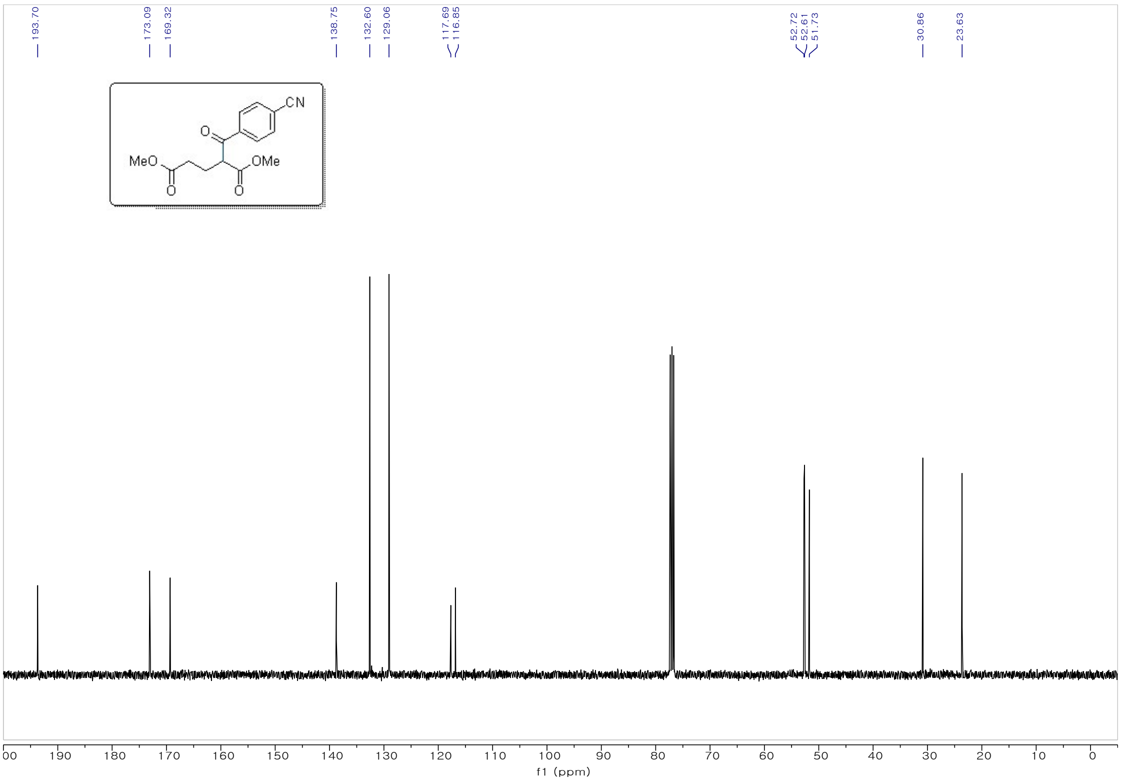

dimethyl 2-(4-(methoxycarbonyl)benzoyl)pentanedioate (3ai).

400 MHz, ^{1}H NMR in Chloroform-*d*

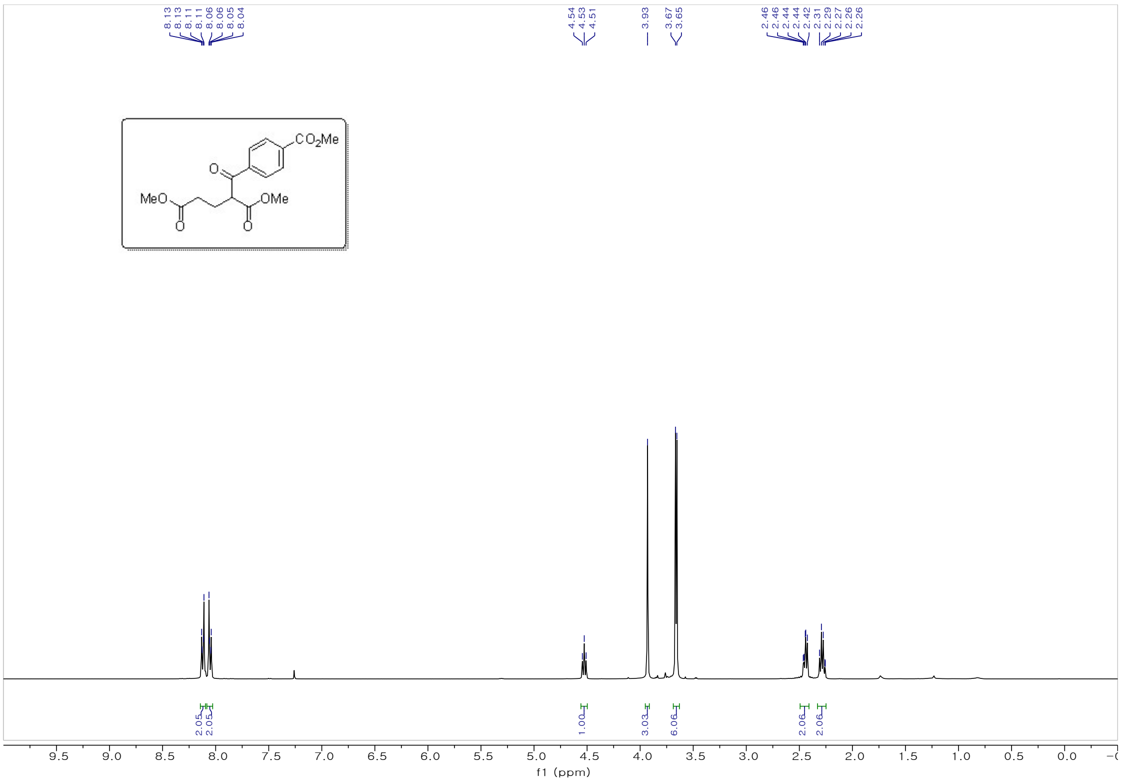

100 MHz, ^{13}C NMR in Chloroform-*d*

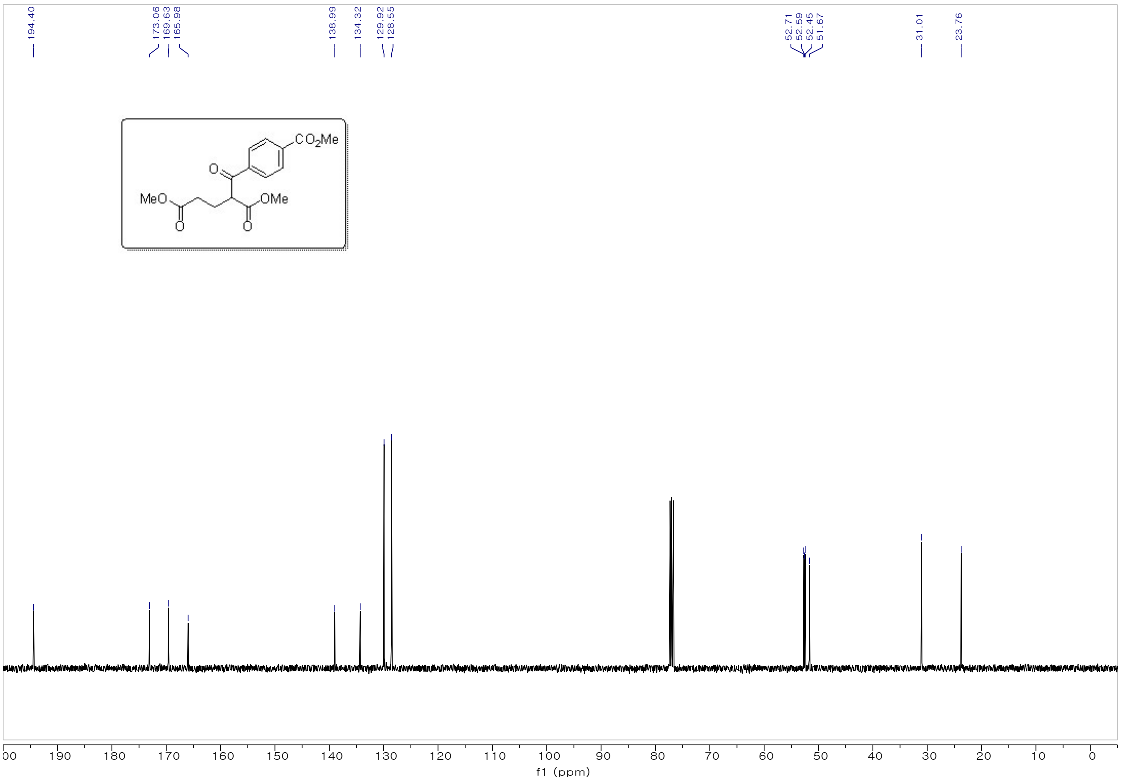

dimethyl 2-(4-(trifluoromethyl)benzoyl)pentanedioate (3aj).

600 MHz, ^{1}H NMR in Chloroform-*d*

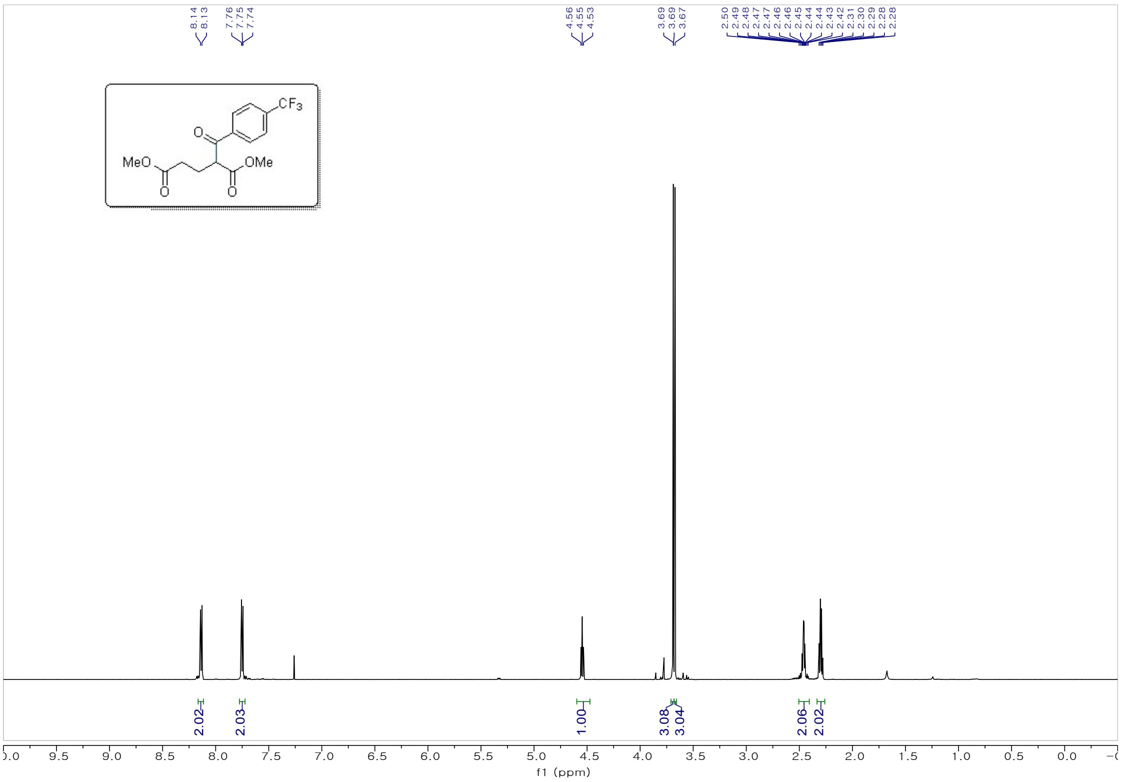

100 MHz, ^{13}C NMR in Chloroform-*d*

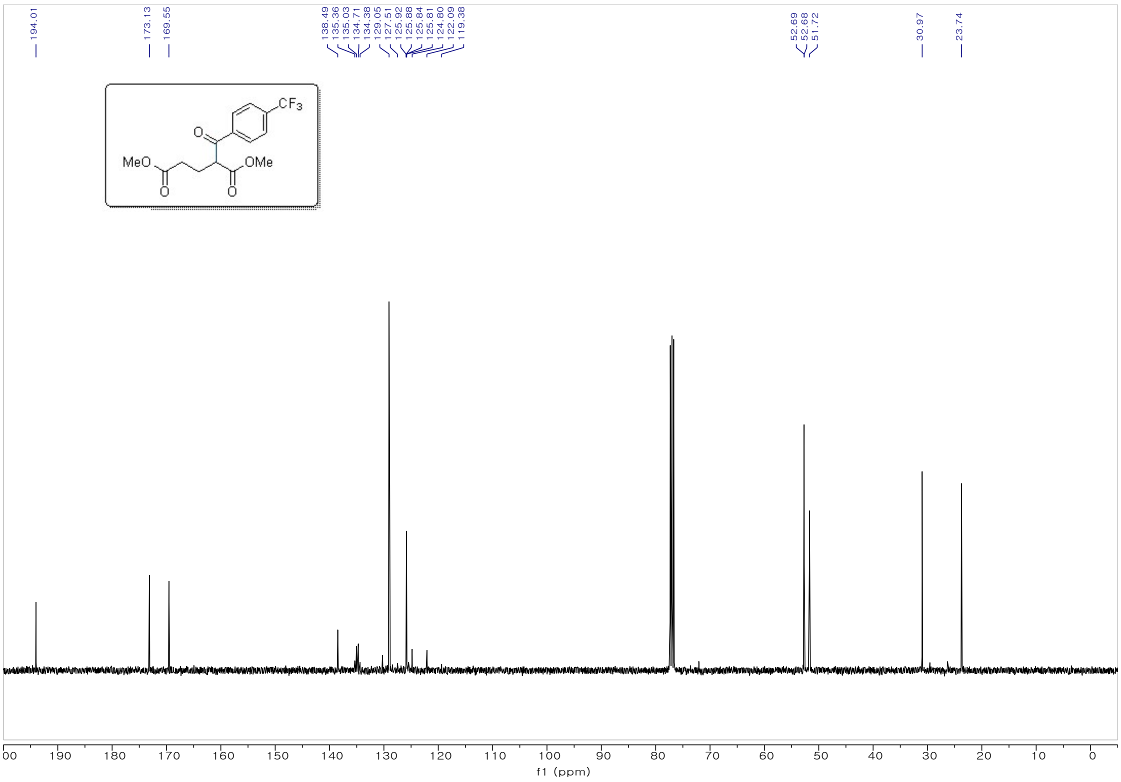

375MHz, ^{19}F NMR in Chloroform-*d*

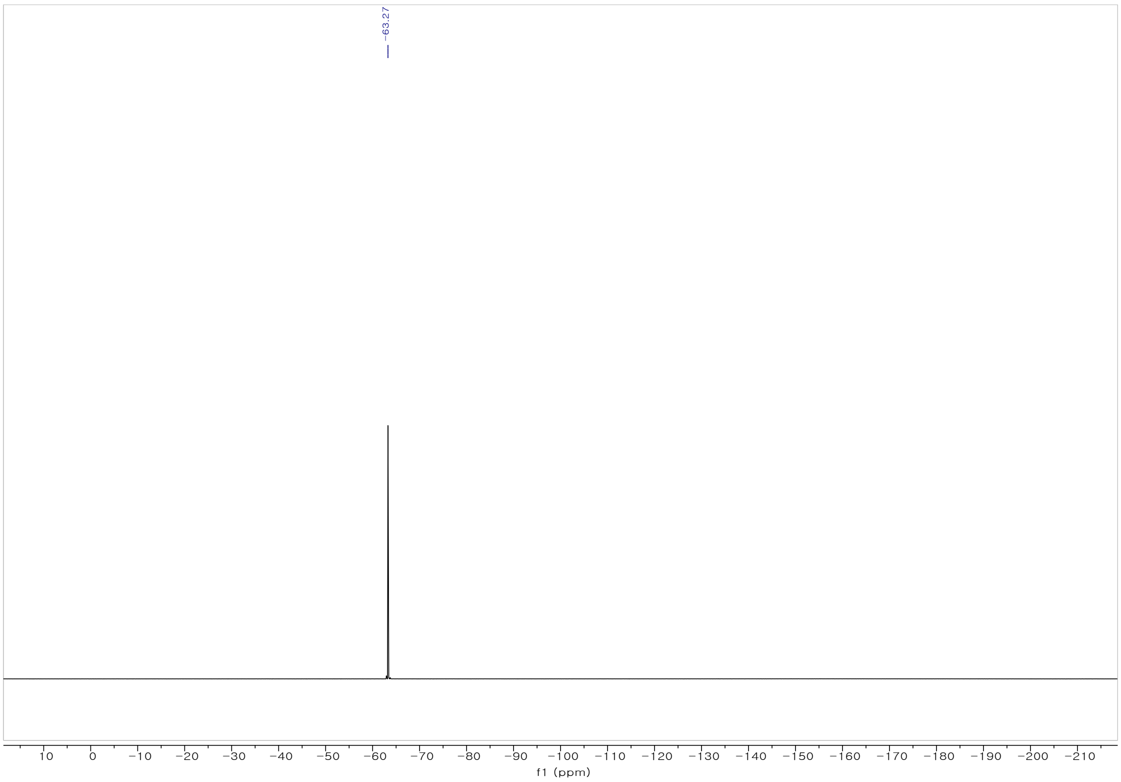

dimethyl 2-(4-(trifluoromethoxy)benzoyl)pentanedioate (3ak).

600 MHz, ^{1}H NMR in Chloroform-*d*

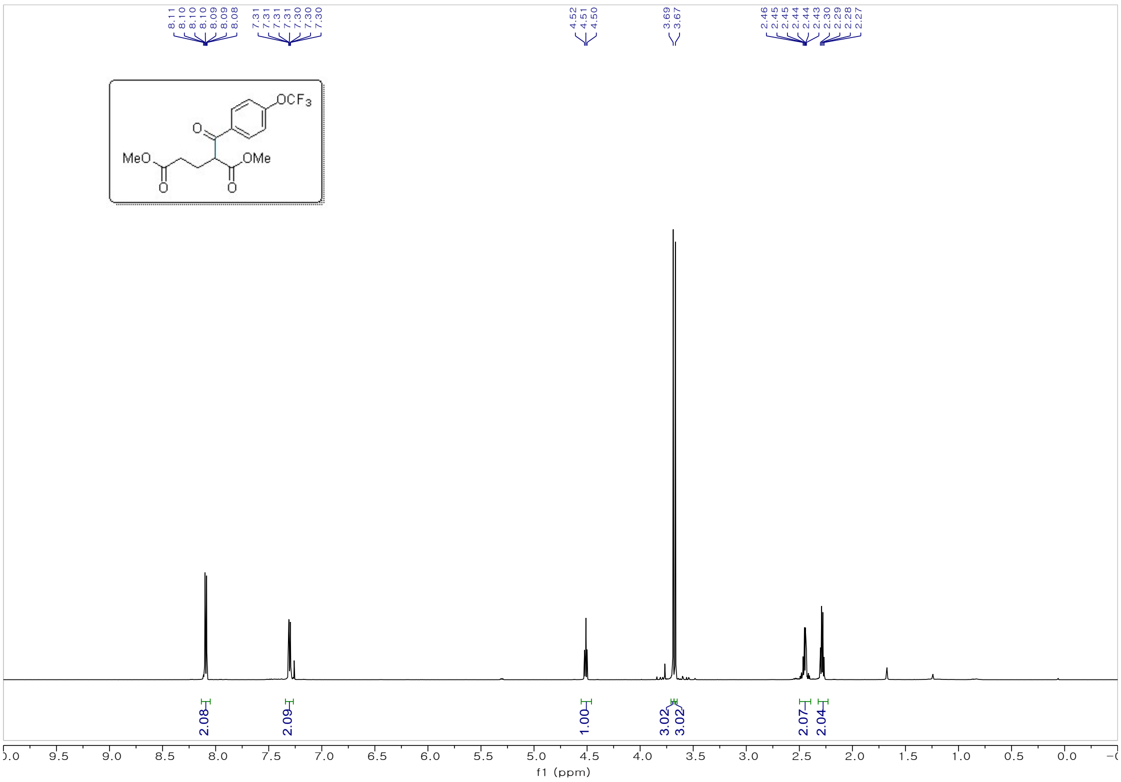

150 MHz, ^{13}C NMR in Chloroform-*d*

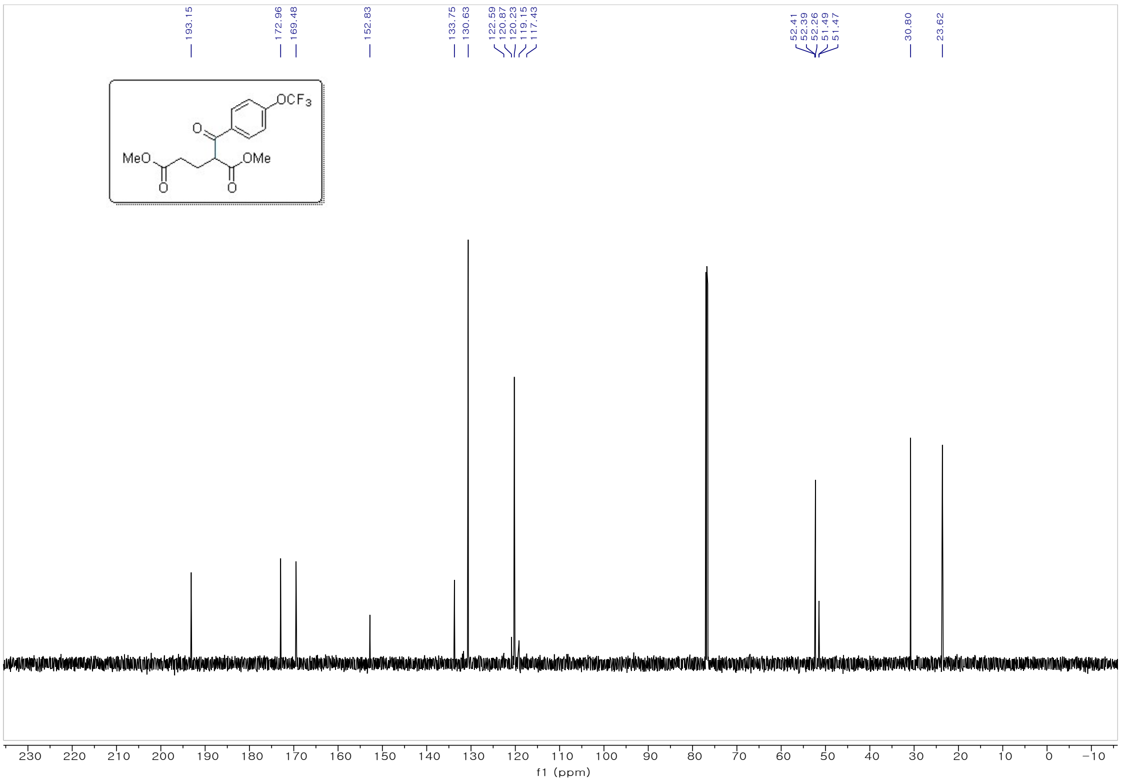

375MHz, ^{19}F NMR in Chloroform-*d*

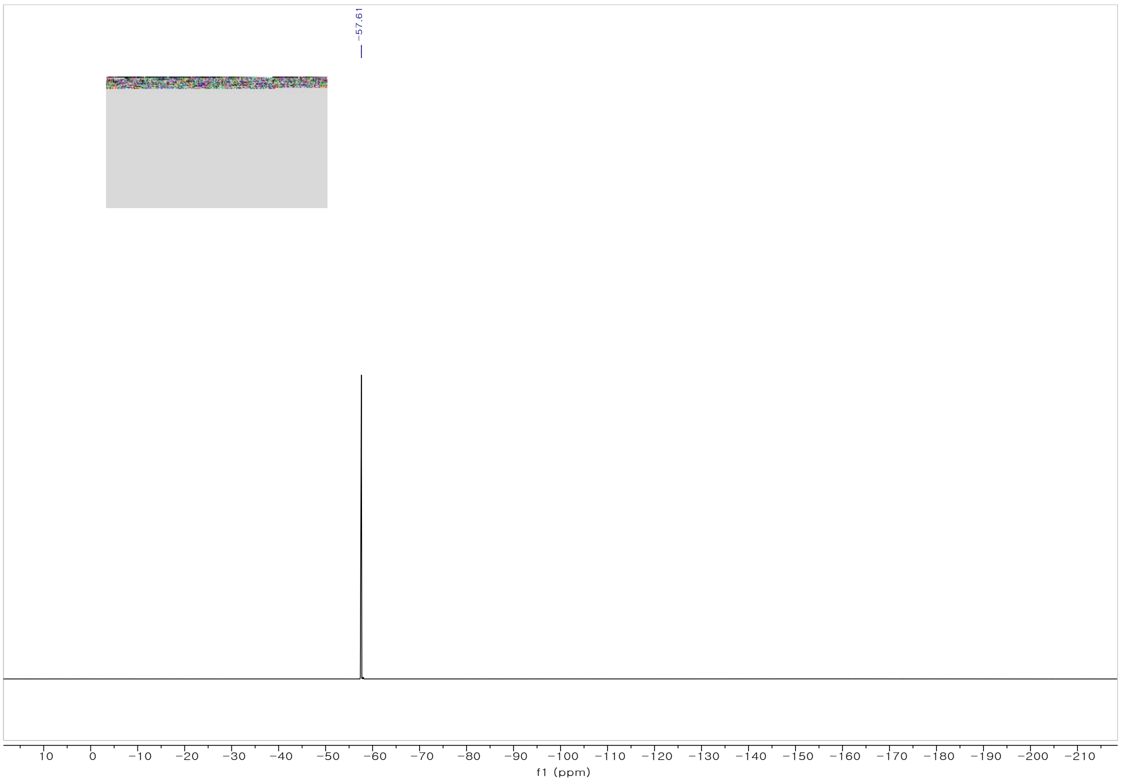

dimethyl 2-(4-fluorobenzoyl)pentanedioate (3al).

600 MHz, ^{1}H NMR in Chloroform-*d*

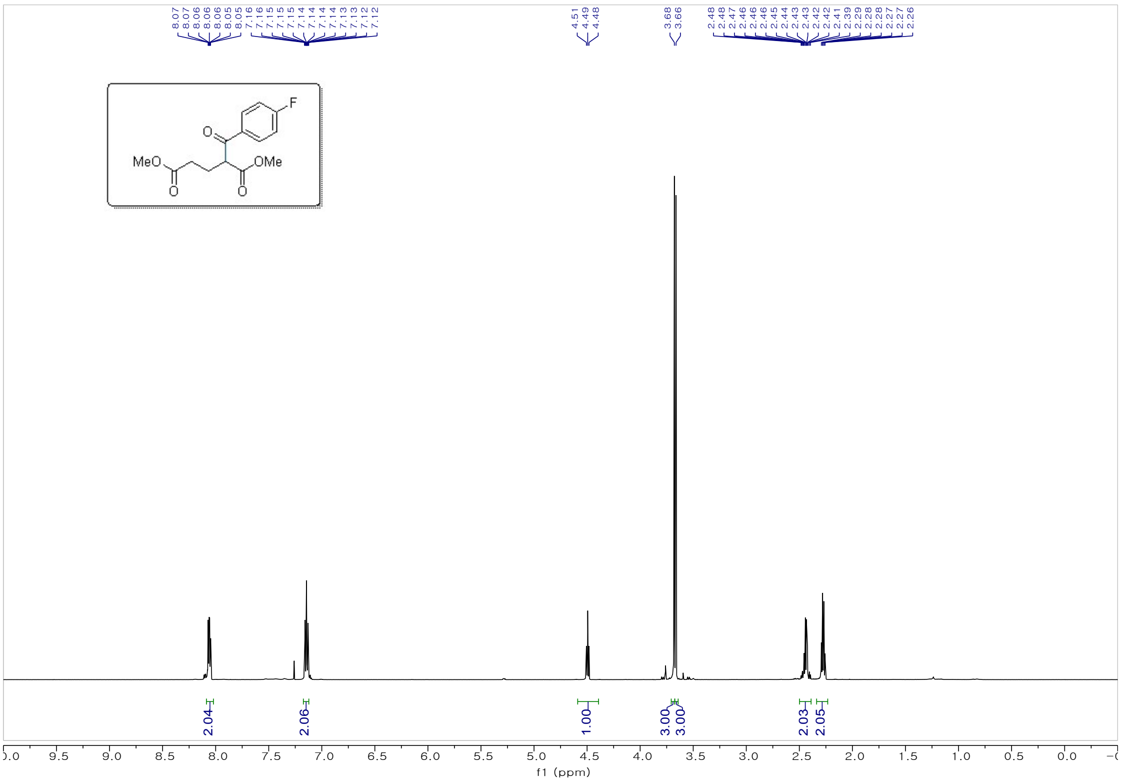

150 MHz, ^{13}C NMR in Chloroform-*d*

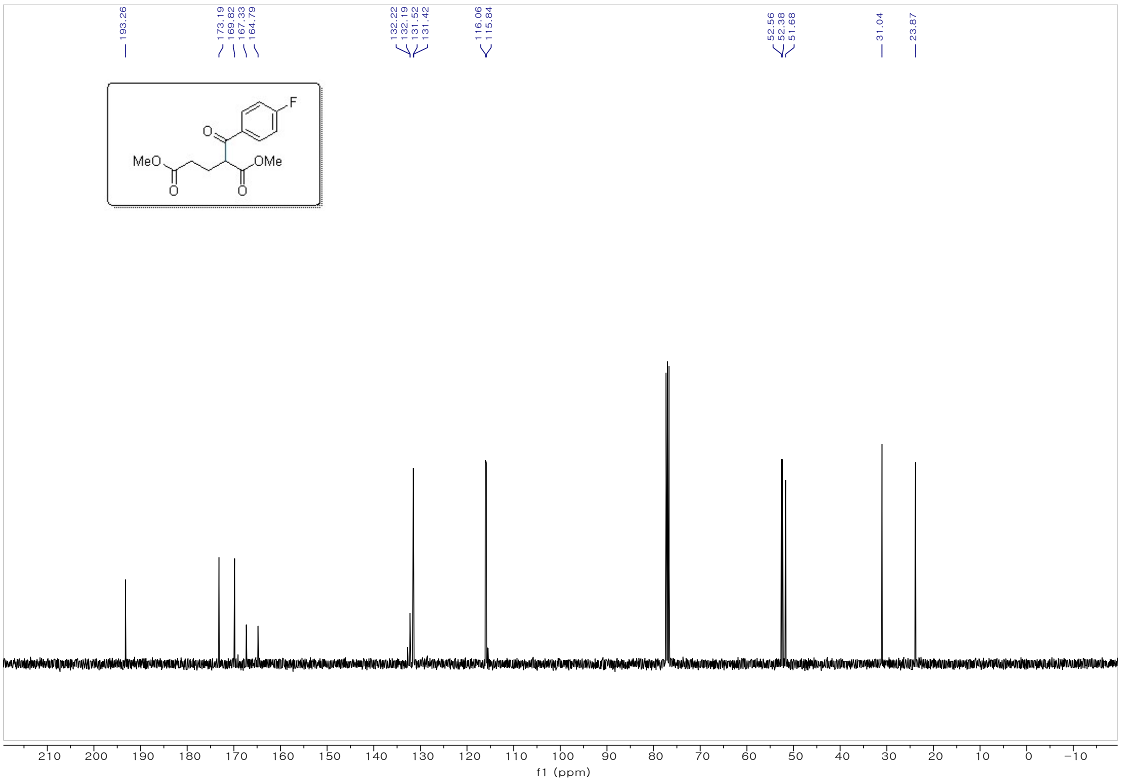

375MHz, ^{19}F NMR in Chloroform-*d*

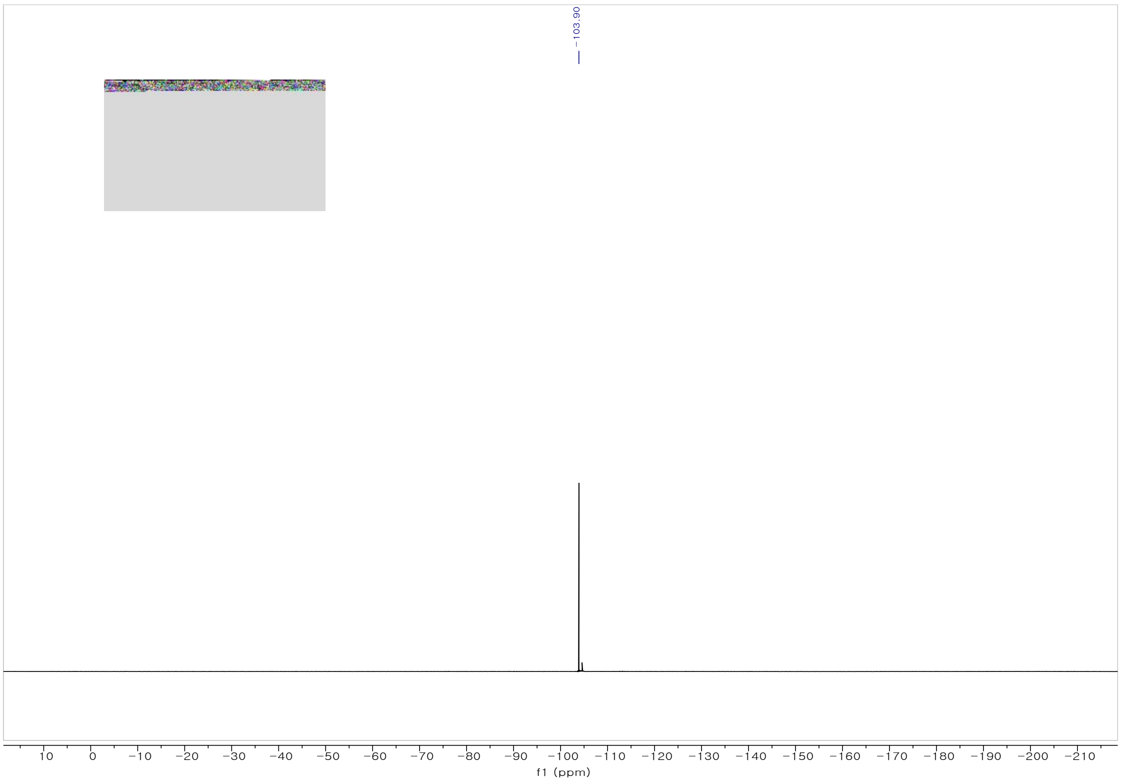

dimethyl 2-(4-chlorobenzoyl)pentanedioate (3am).

400 MHz, ^1H NMR in Chloroform-*d*

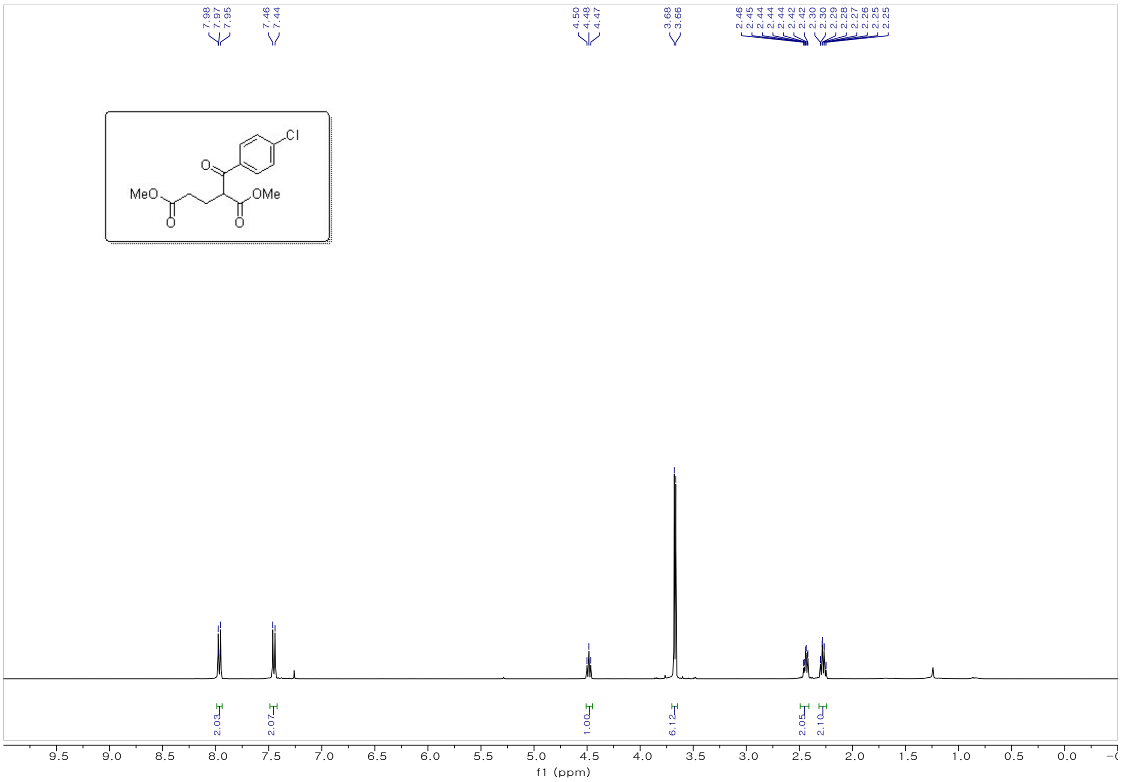

100 MHz, ^{13}C NMR in Chloroform-*d*

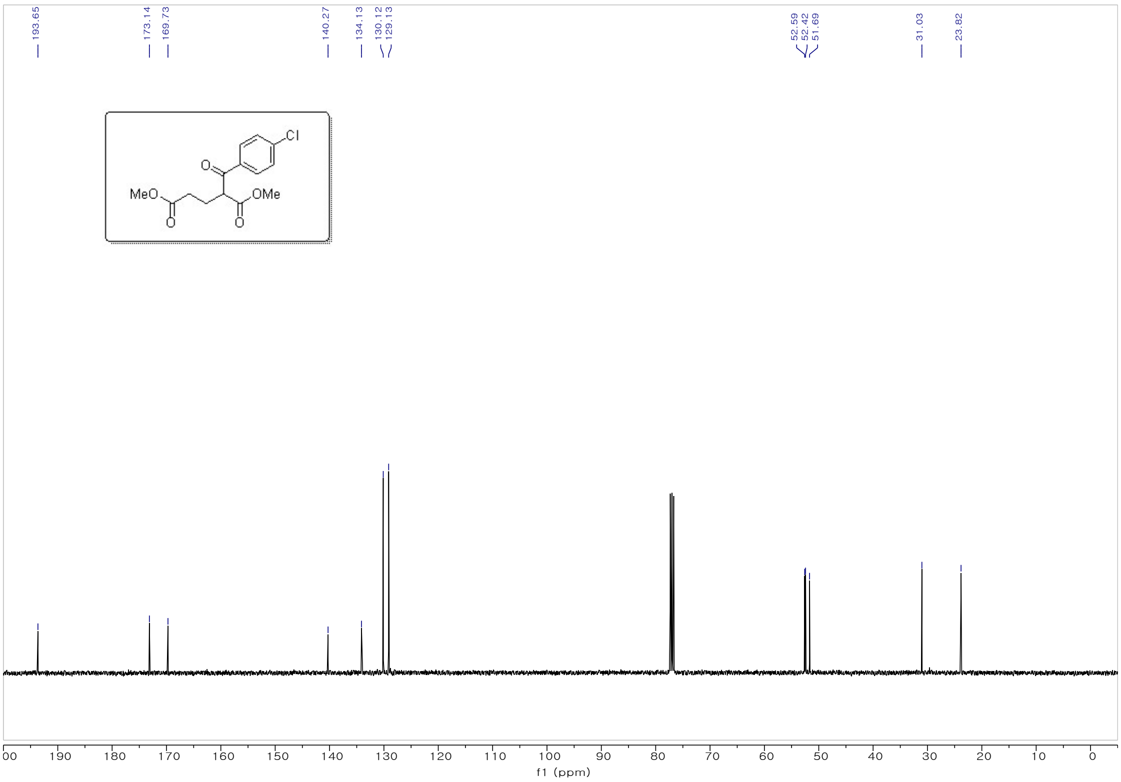

dimethyl 2-(4-bromobenzoyl)pentanedioate (3an).

600 MHz, ^{1}H NMR in Chloroform-*d*

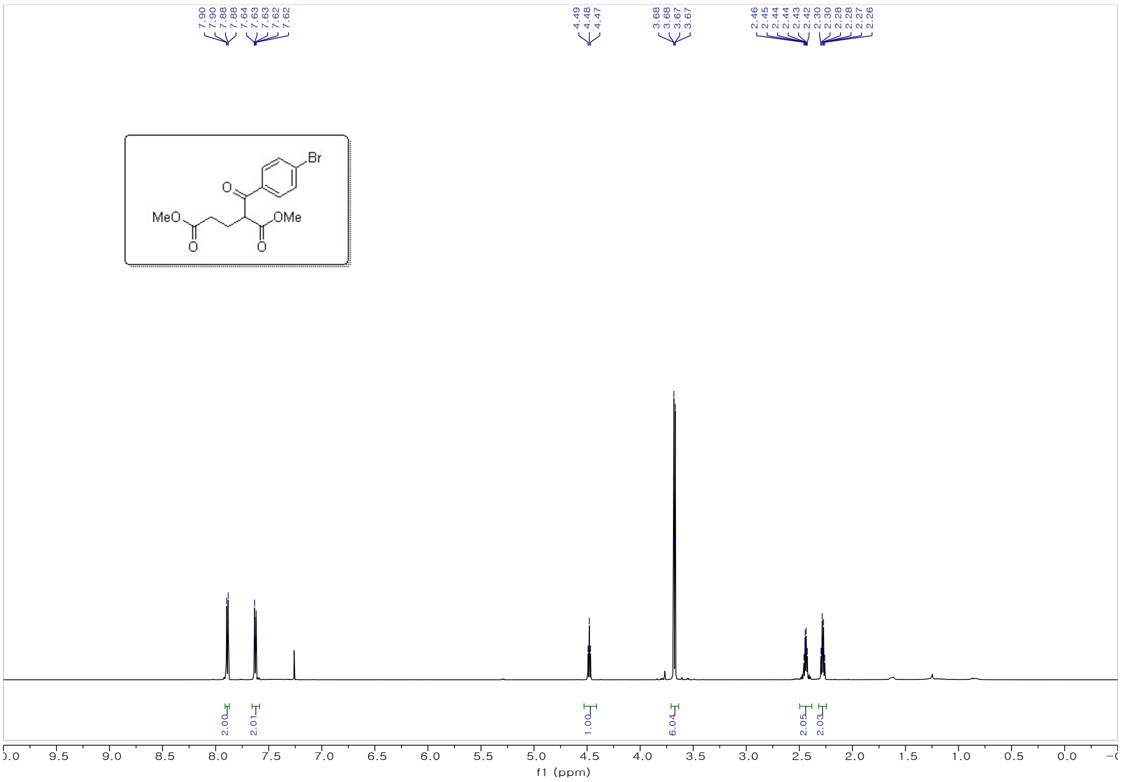

150 MHz, ^{13}C NMR in Chloroform-*d*

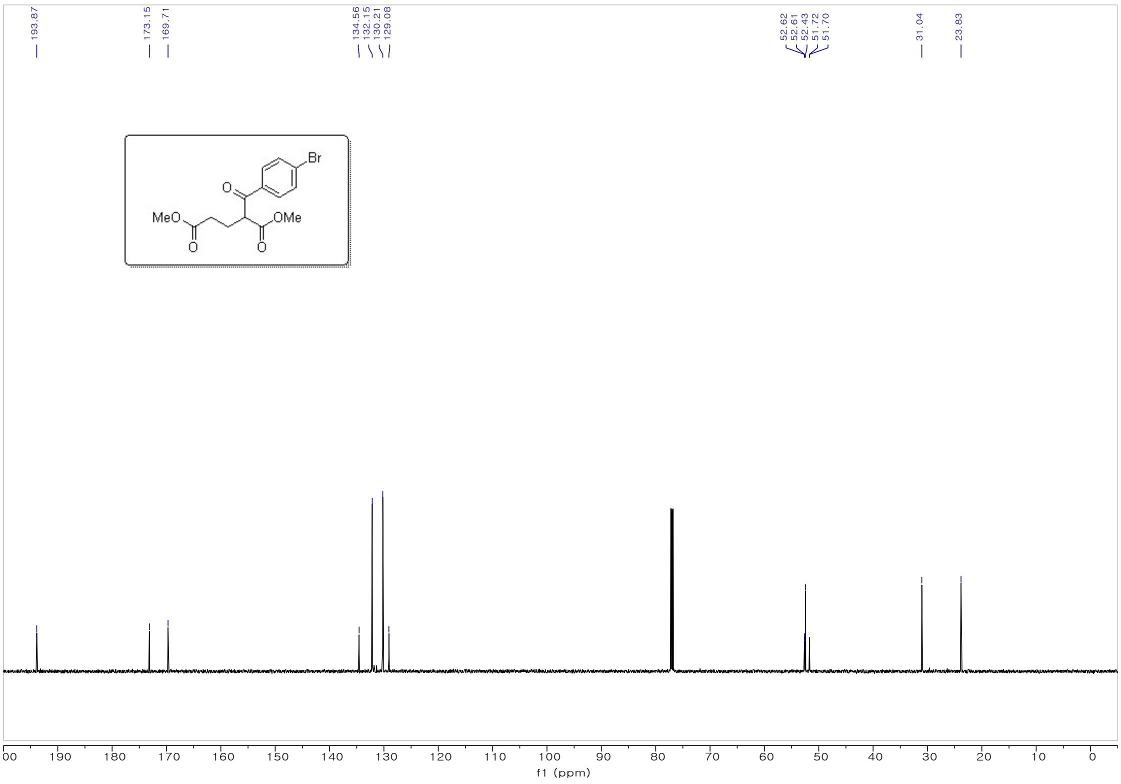

dimethyl 2-(3-(methoxycarbonyl)benzoyl)pentanedioate (3ao).

600 MHz, ^{1}H NMR in Chloroform-*d*

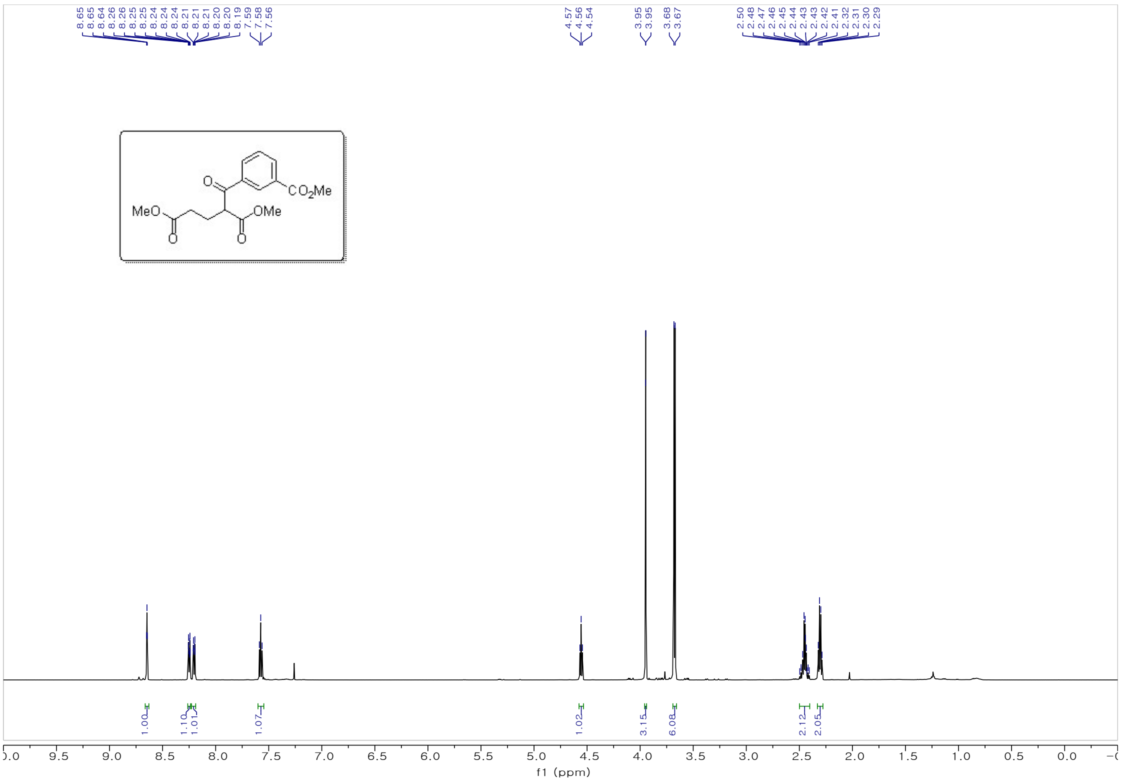

150 MHz, ^{13}C NMR in Chloroform-*d*

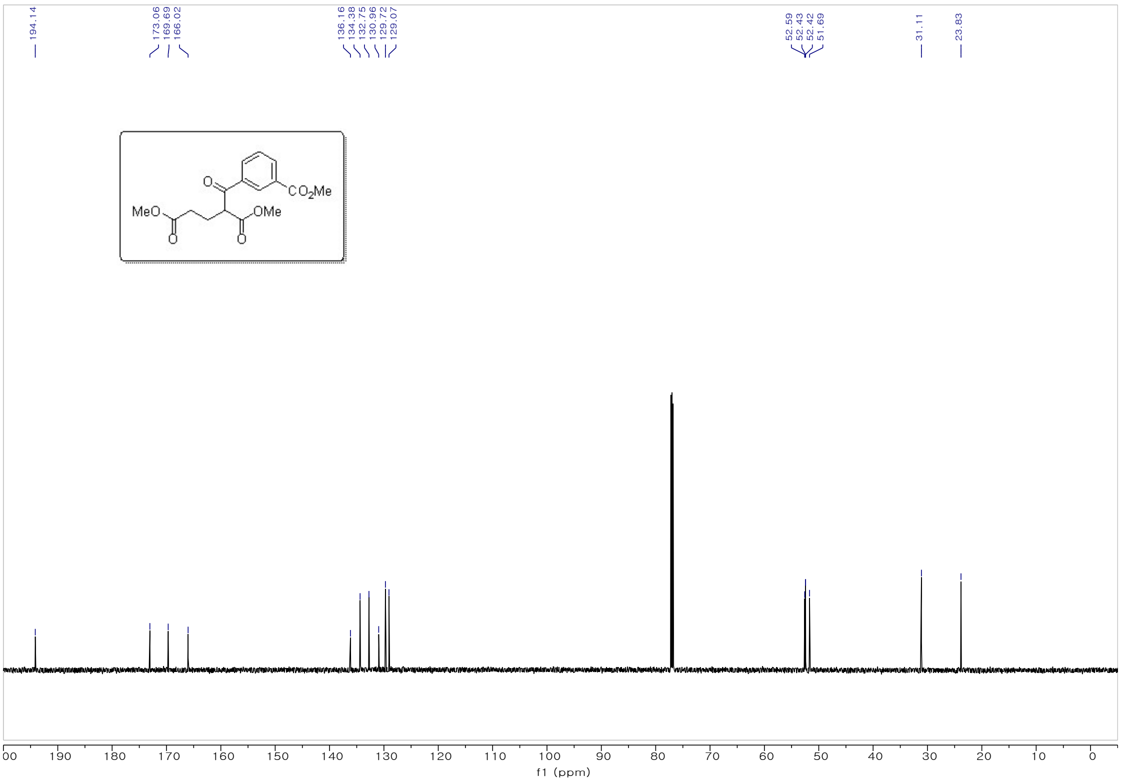

dimethyl 2-(3-(trifluoromethoxy)benzoyl)pentanedioate (3ap).

600 MHz, ^{1}H NMR in Chloroform-*d*

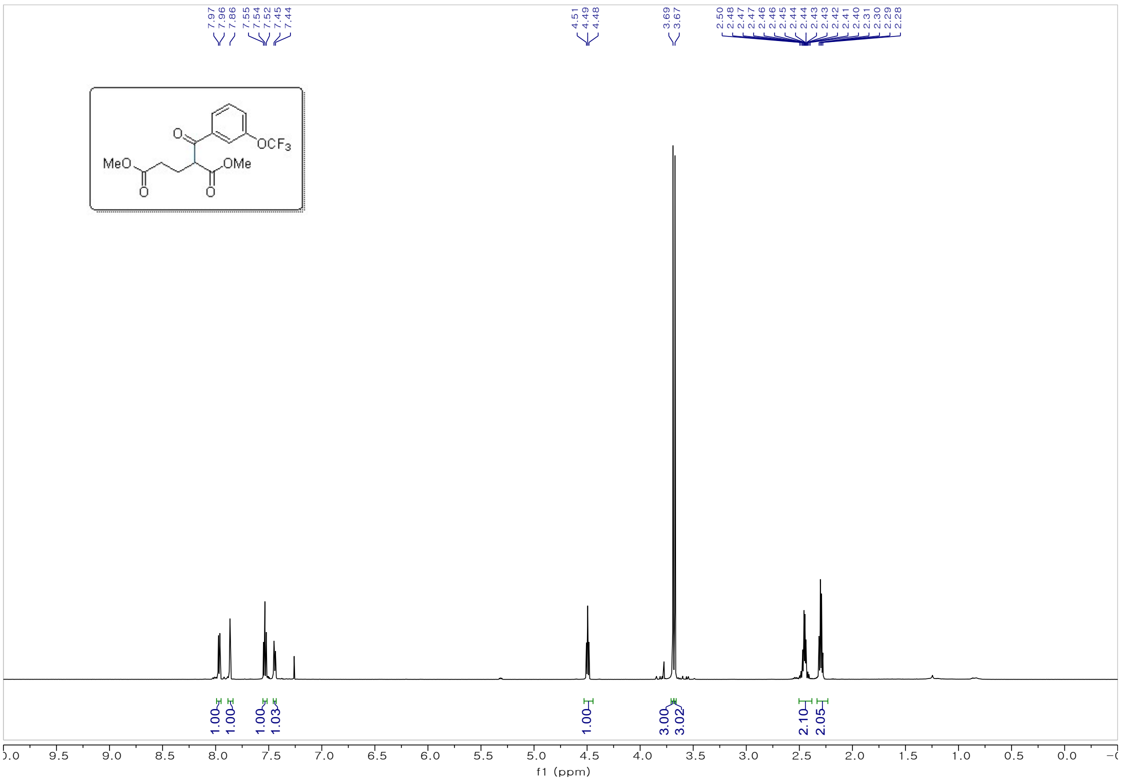

150 MHz, ^{13}C NMR in Chloroform-*d*

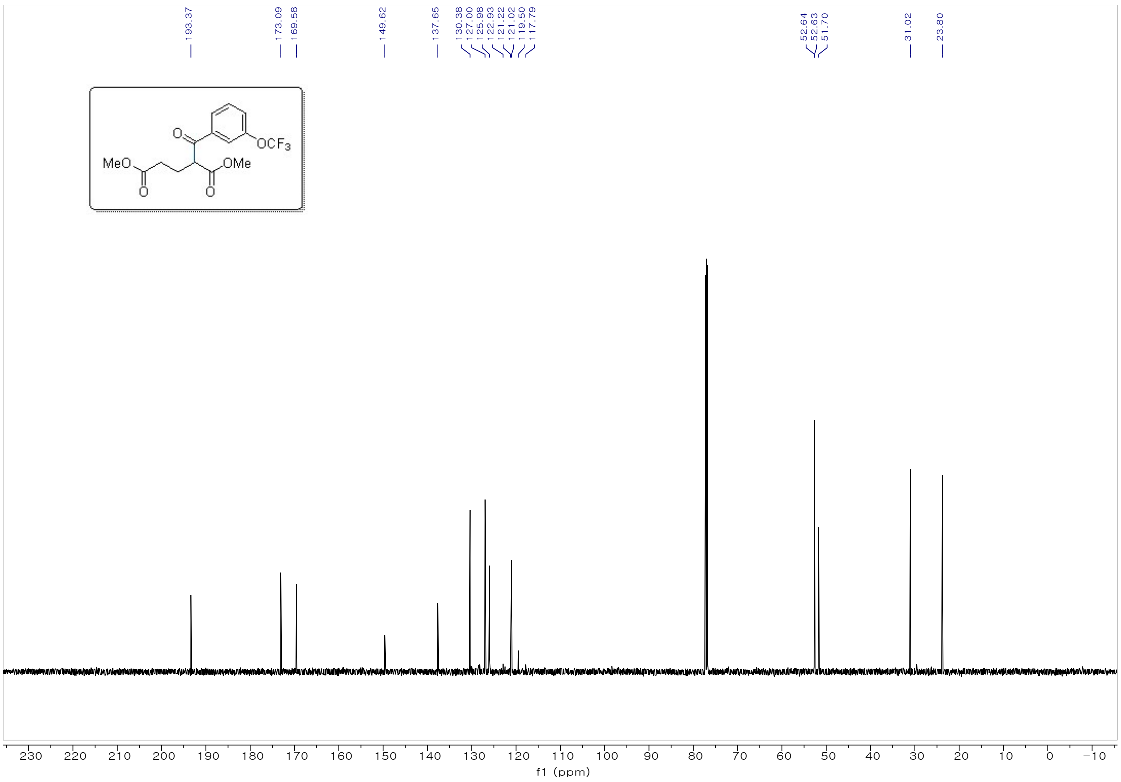

375MHz, ^{19}F NMR in Chloroform-*d*

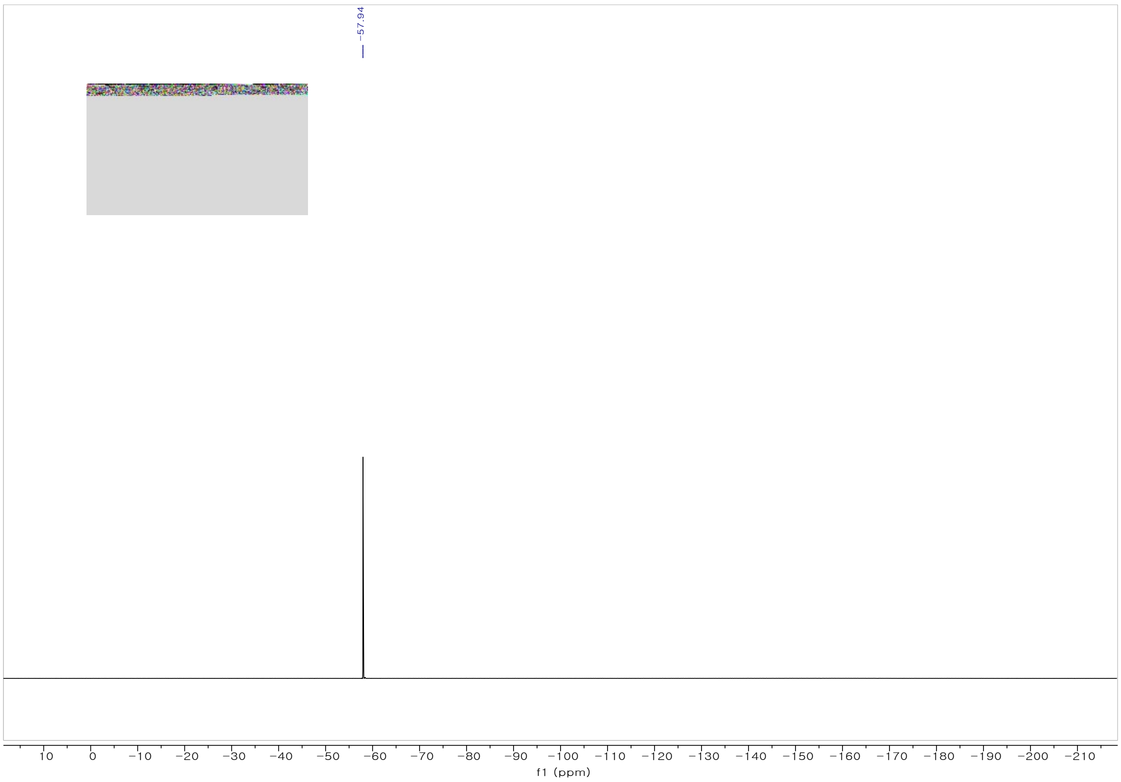

dimethyl 2-(3-methylbenzoyl)pentanedioate (3aq).

600 MHz, ^{1}H NMR in Chloroform-*d*

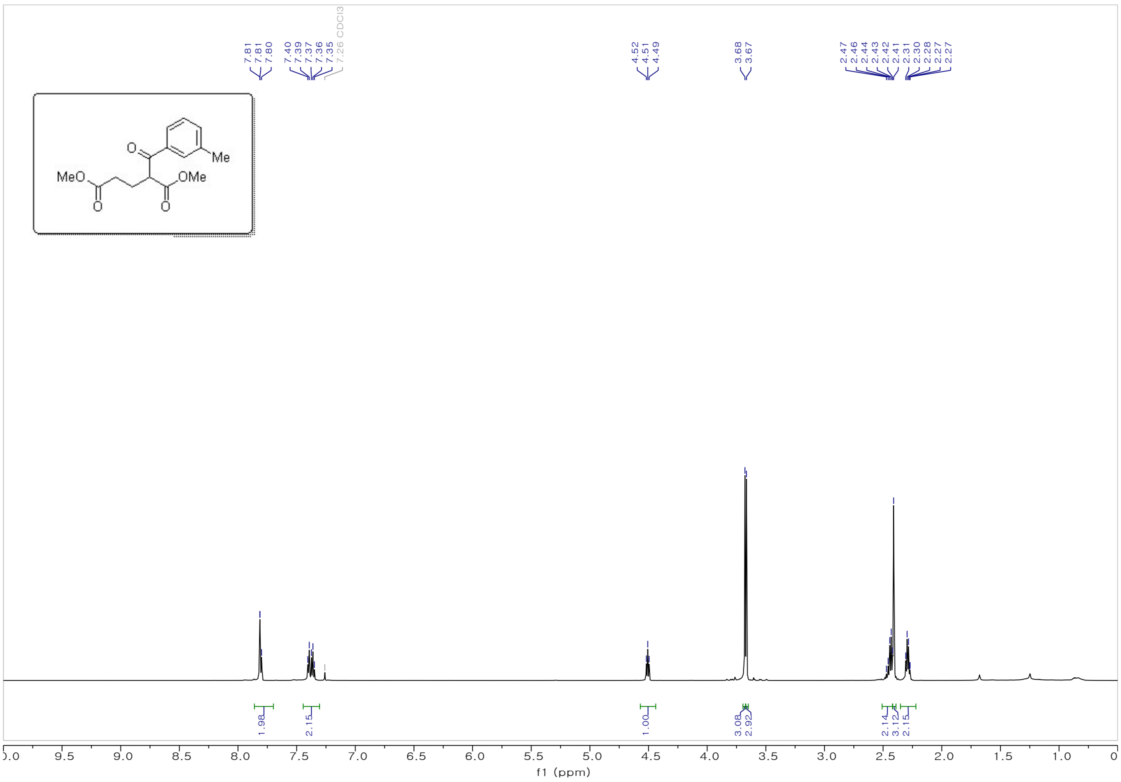

150 MHz, ^{13}C NMR in Chloroform-*d*

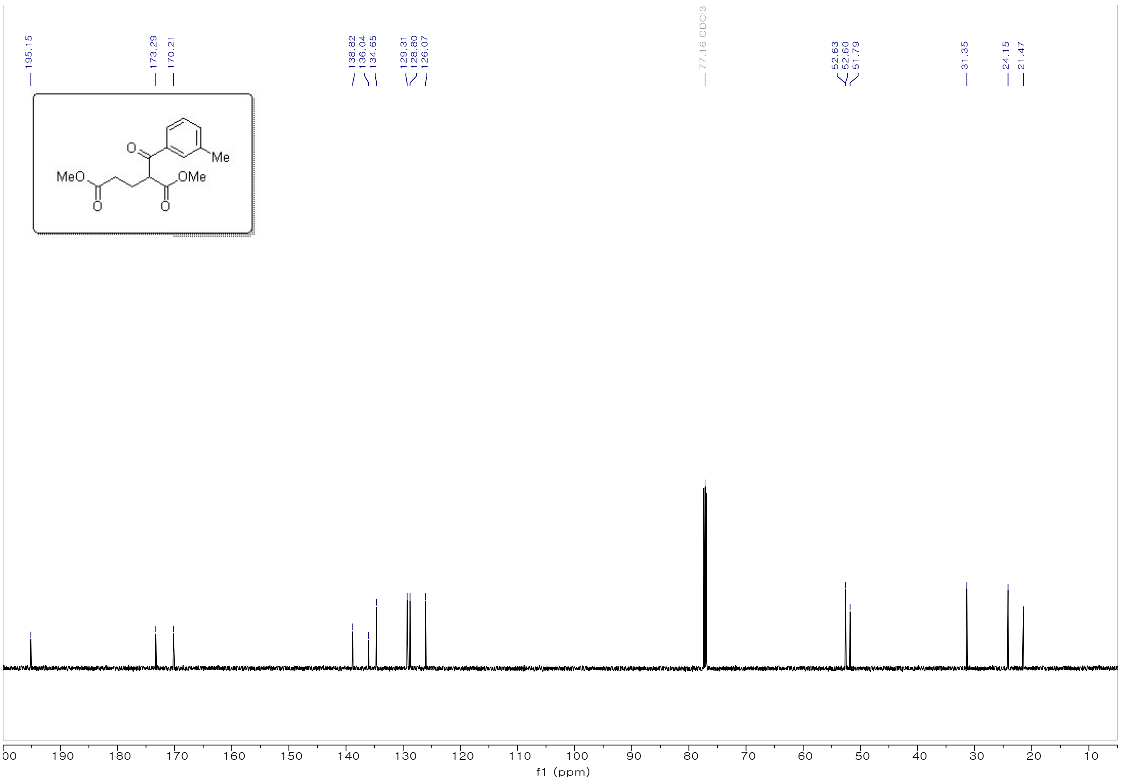

dimethyl 2-(2-fluorobenzoyl)pentanedioate (3ar).

400 MHz, ^{1}H NMR in Chloroform-*d*

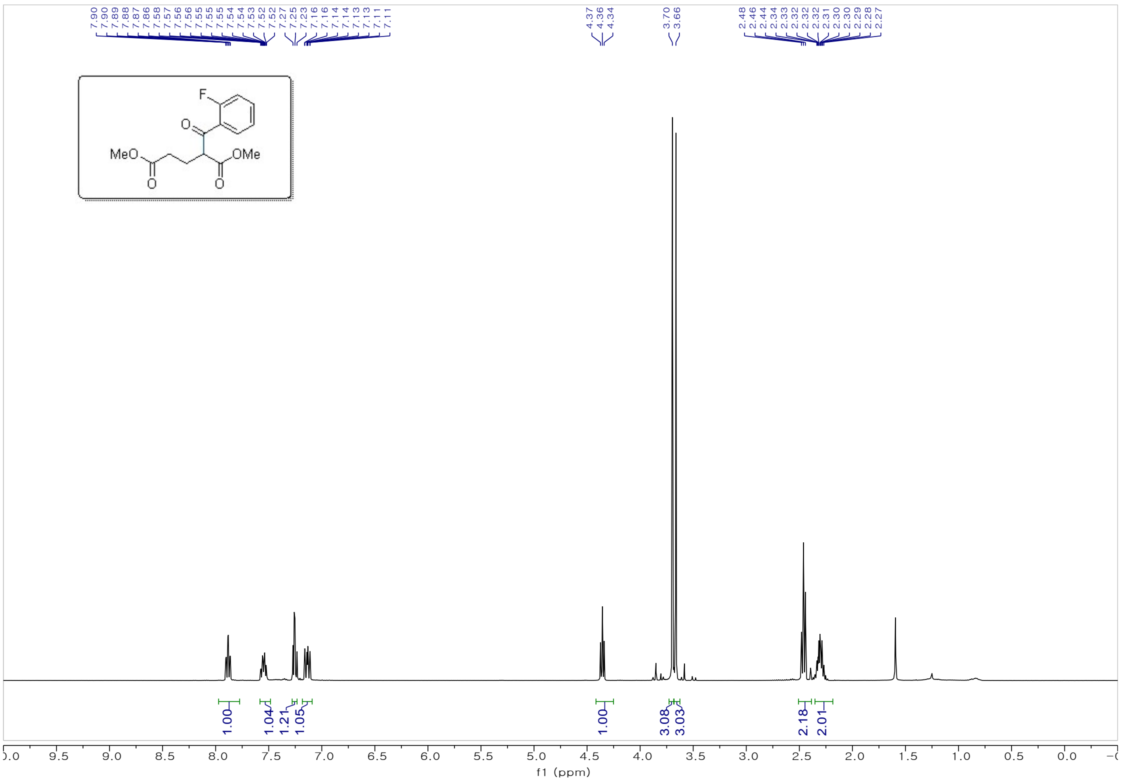

100 MHz, ^{13}C NMR in Chloroform-*d*

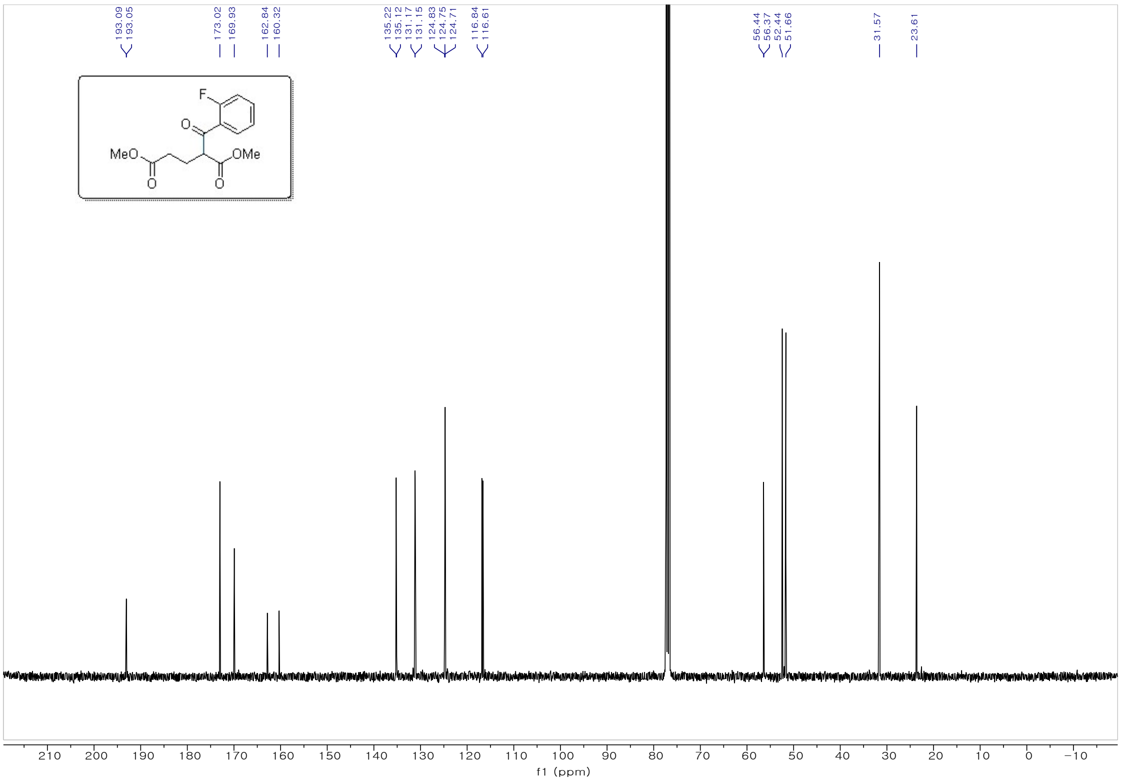

375MHz, ^{19}F NMR in Chloroform-*d*

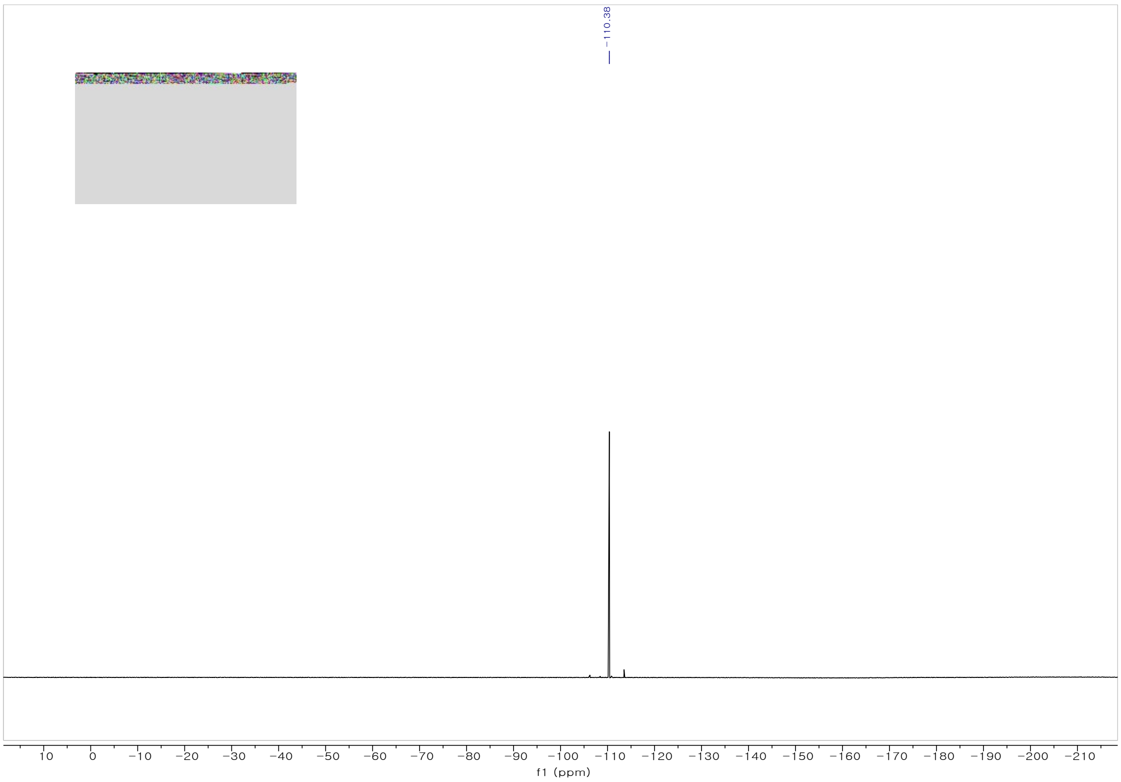

dimethyl 2-(4-acetoxy-3-methoxybenzoyl)pentanedioate (3as).

600 MHz, ¹H NMR in Chloroform-*d*

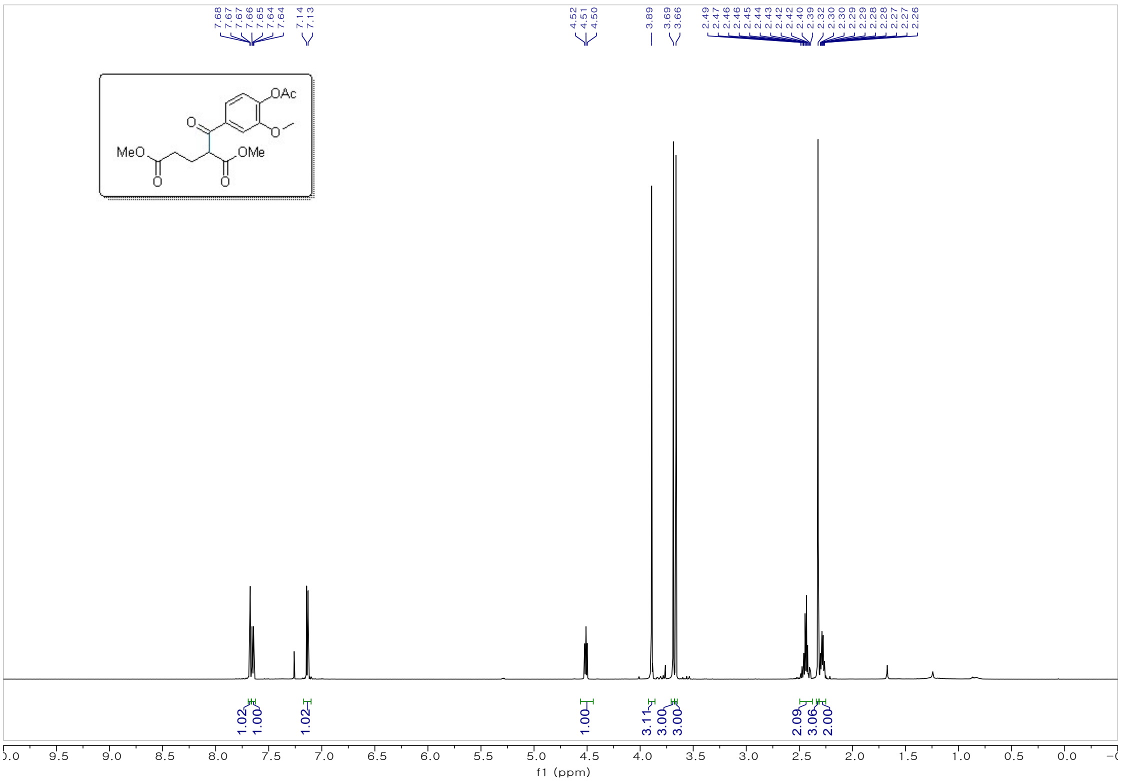

150 MHz, ¹³C NMR in Chloroform-*d*

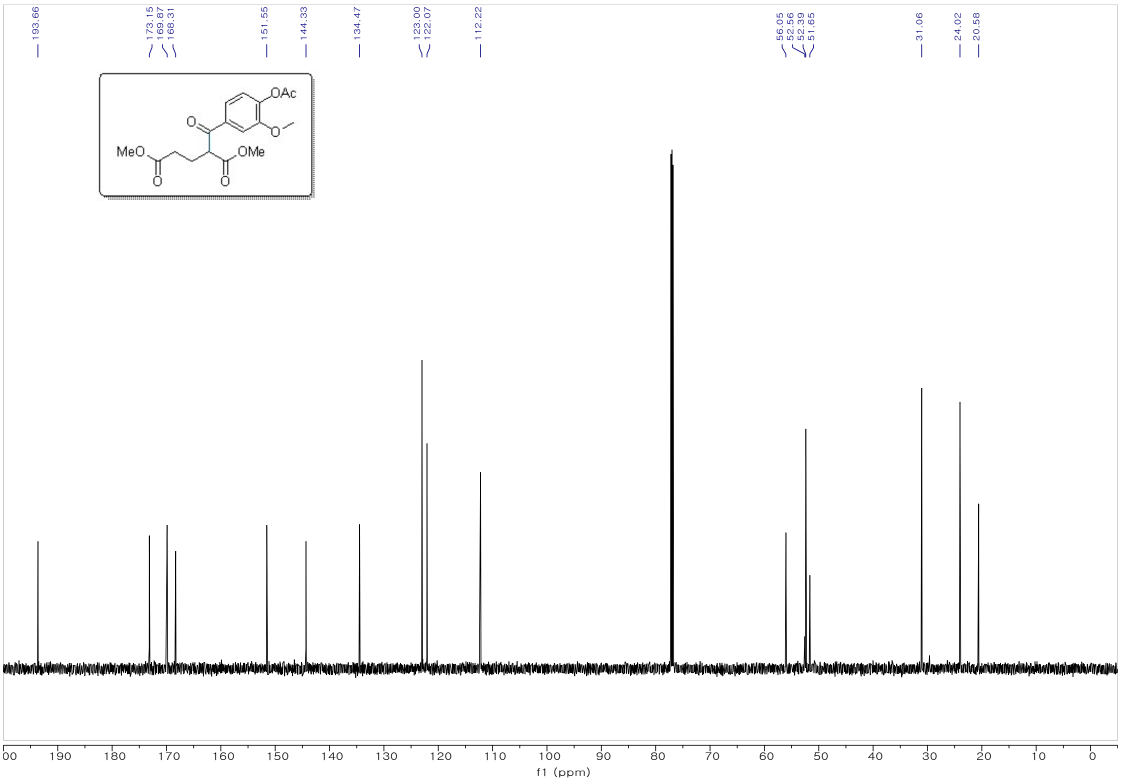

dimethyl 2-(2-naphthoyl)pentanedioate (3at).

400 MHz, ^{1}H NMR in Chloroform-*d*

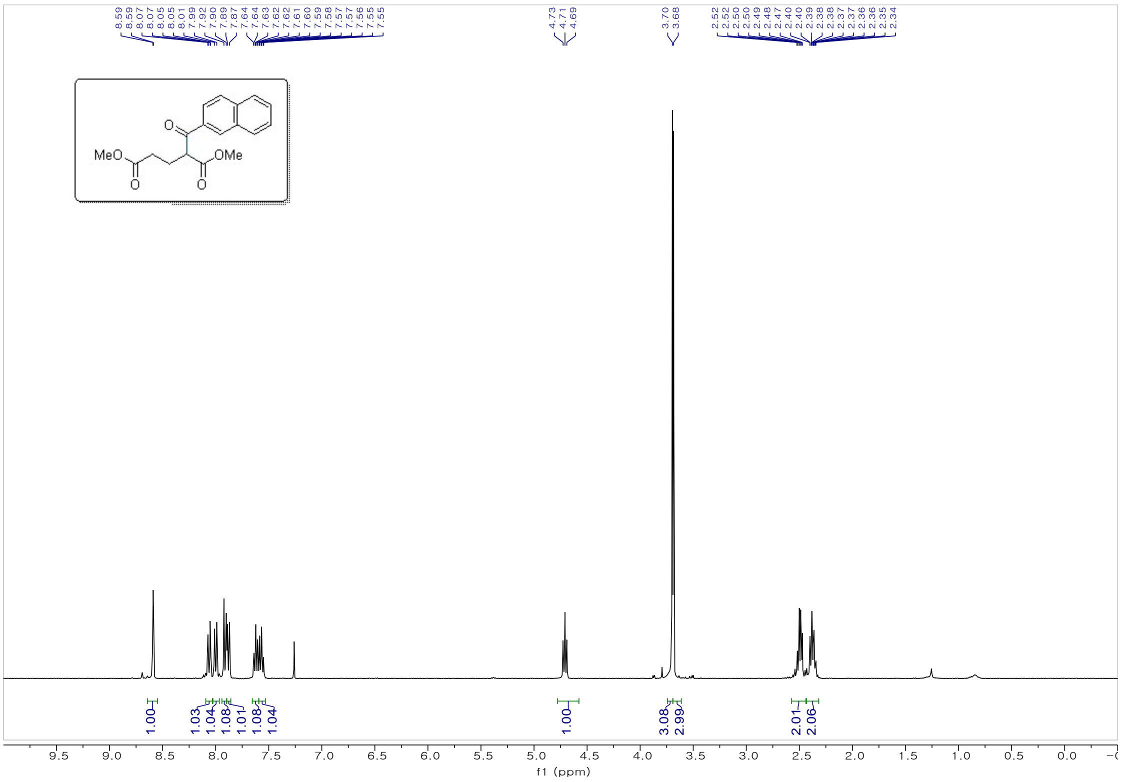

100 MHz, ^{13}C NMR in Chloroform-*d*

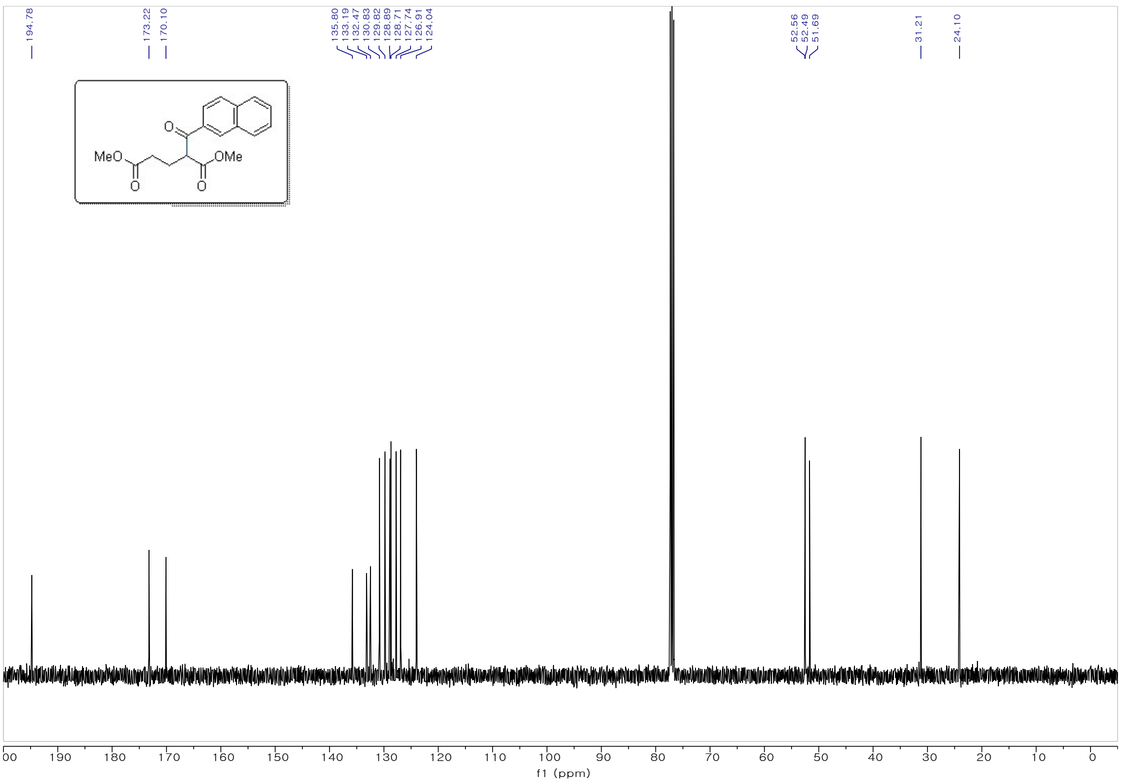

dimethyl 2-(thiophene-2-carbonyl)pentanedioate (3au).

600 MHz, ^{1}H NMR in Chloroform-*d*

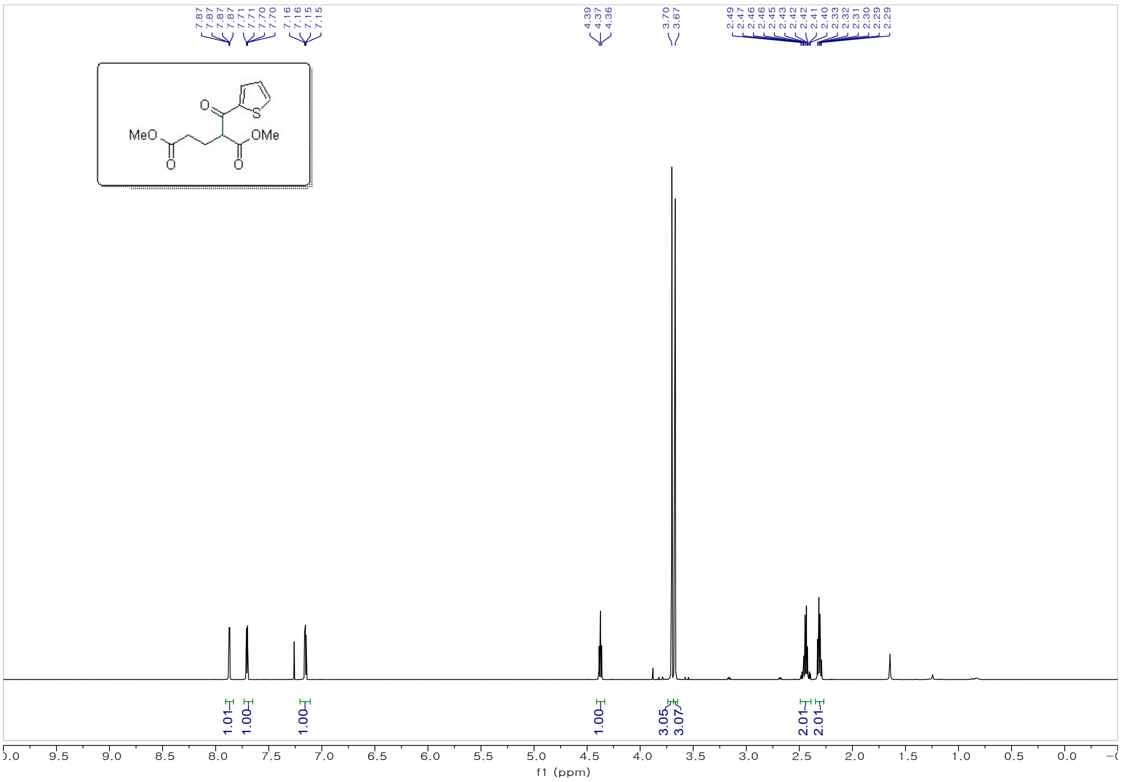

100 MHz, ^{13}C NMR in Chloroform-*d*

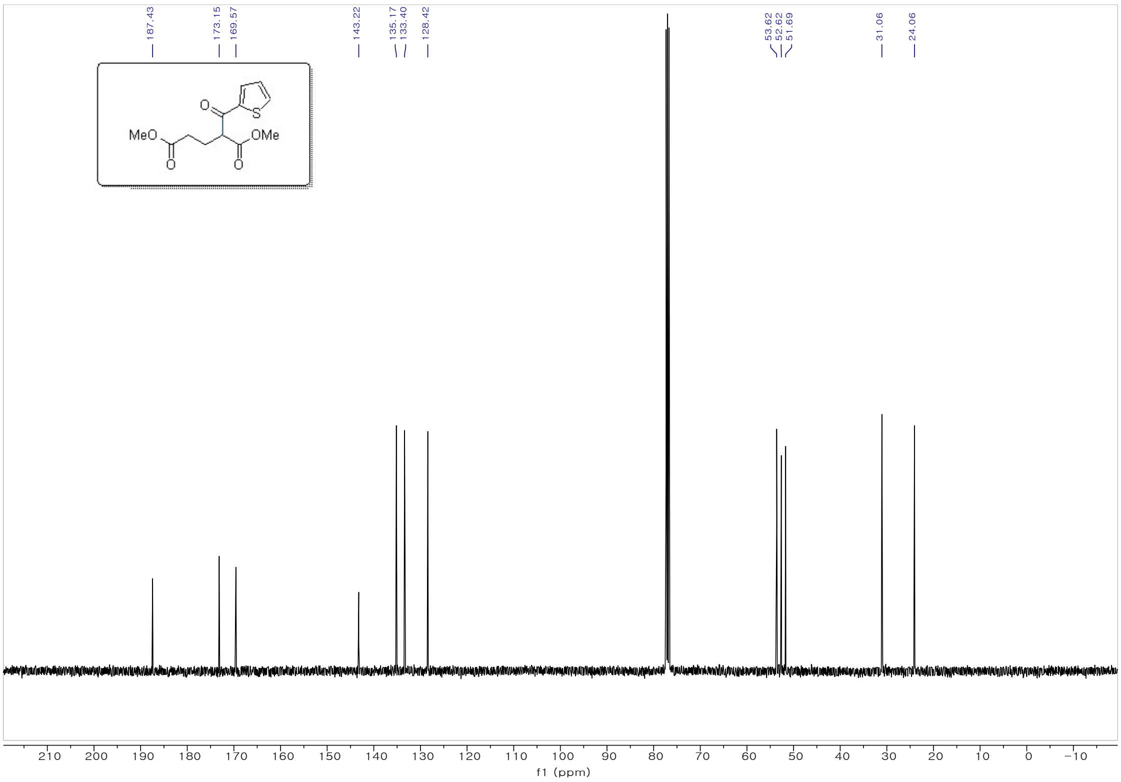

dimethyl 2-(benzofuran-2-carbonyl)pentanedioate (3av).

400 MHz, ^{1}H NMR in Chloroform-*d*

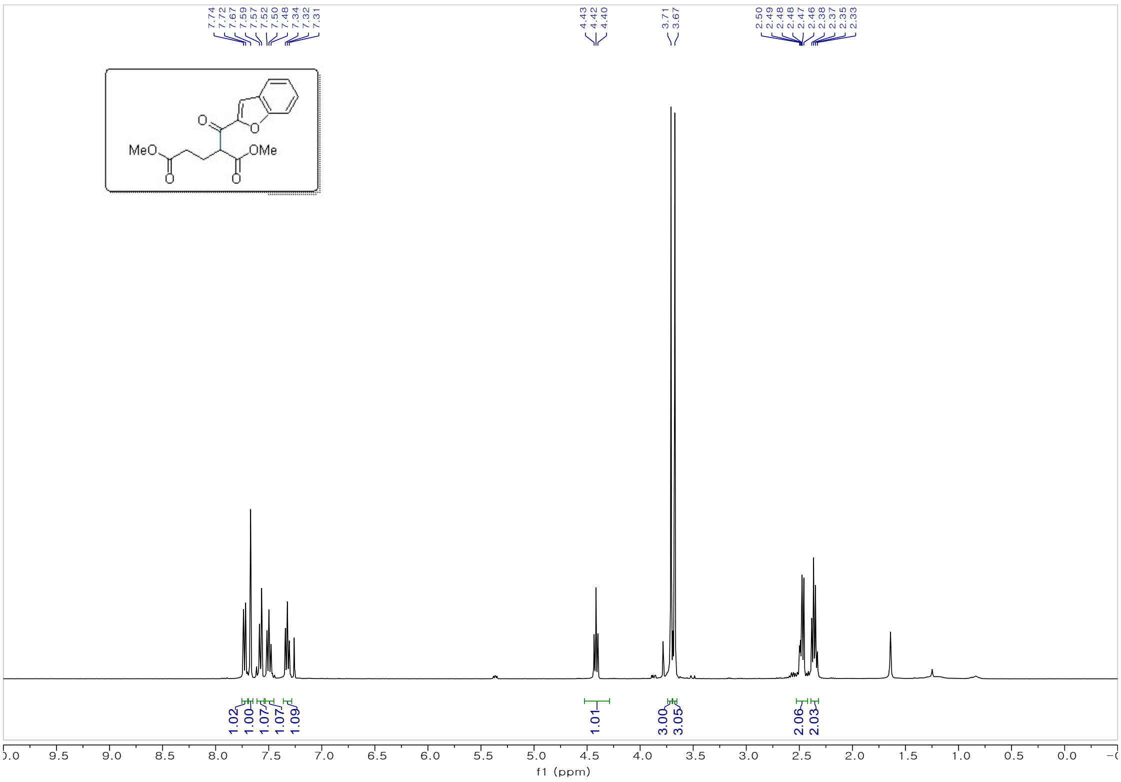

100 MHz, ^{13}C NMR in Chloroform-*d*

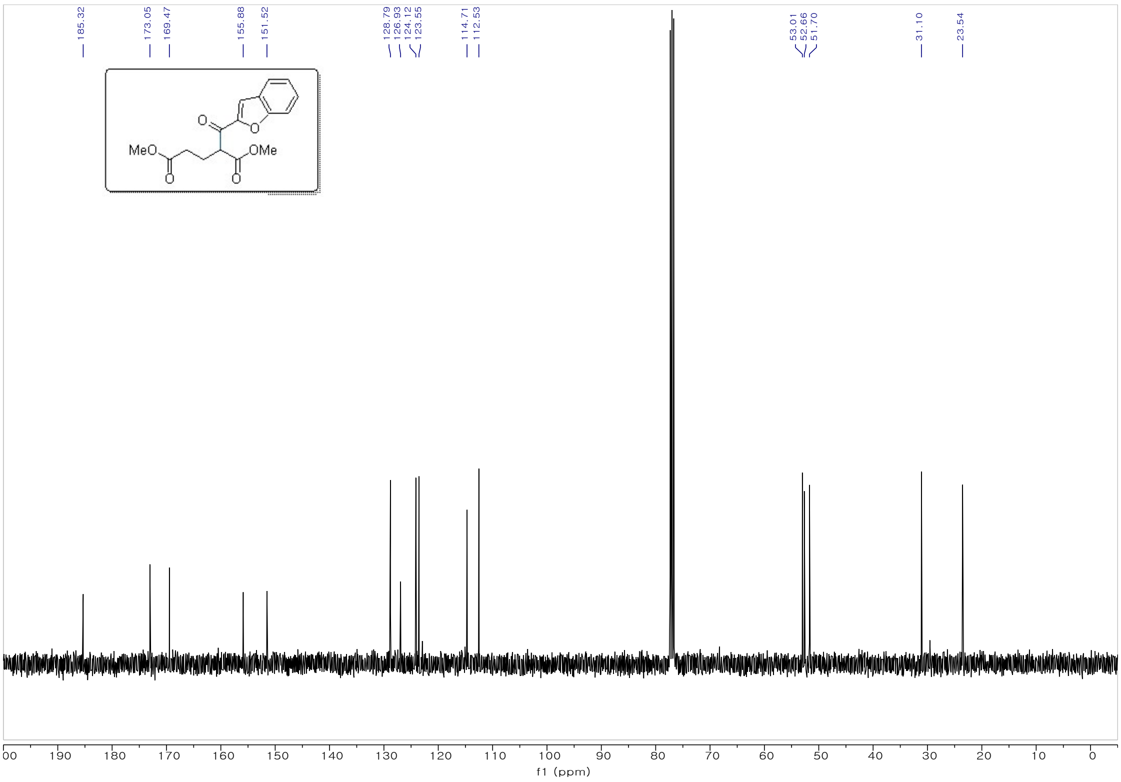

dimethyl 2-nicotinoylpentanedioate (3aw).

400 MHz, ^{1}H NMR in Chloroform-*d*

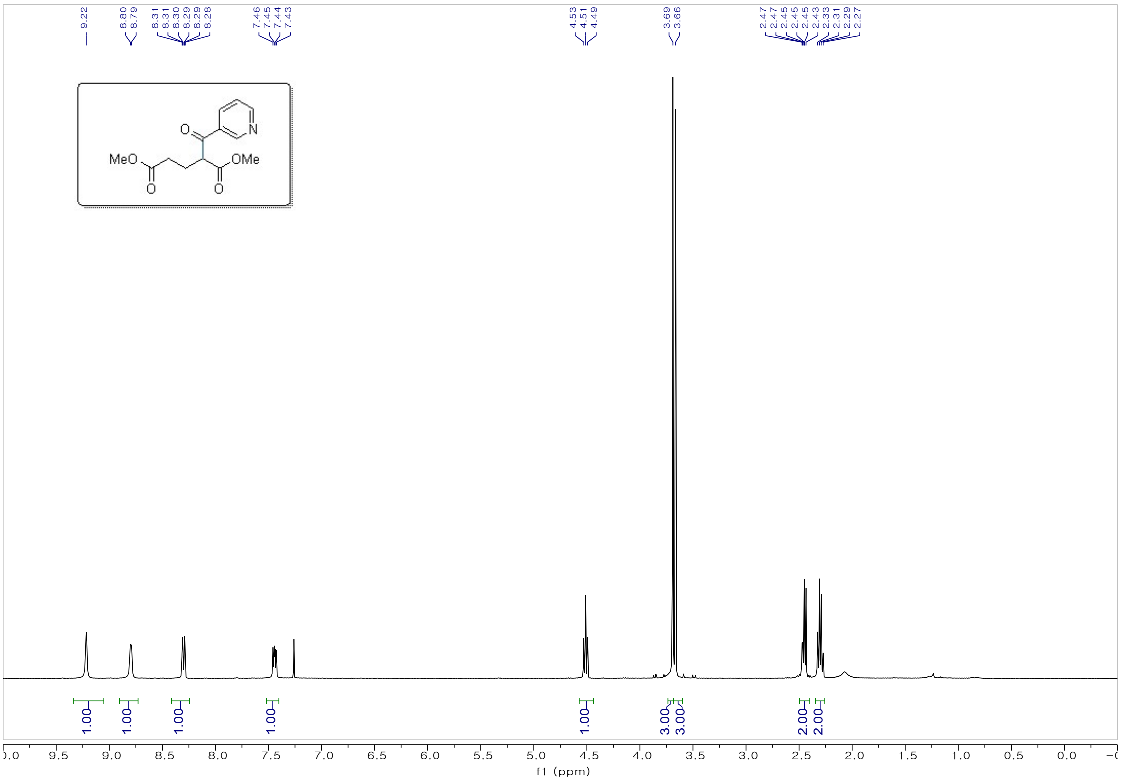

100 MHz, ^{13}C NMR in Chloroform-*d*

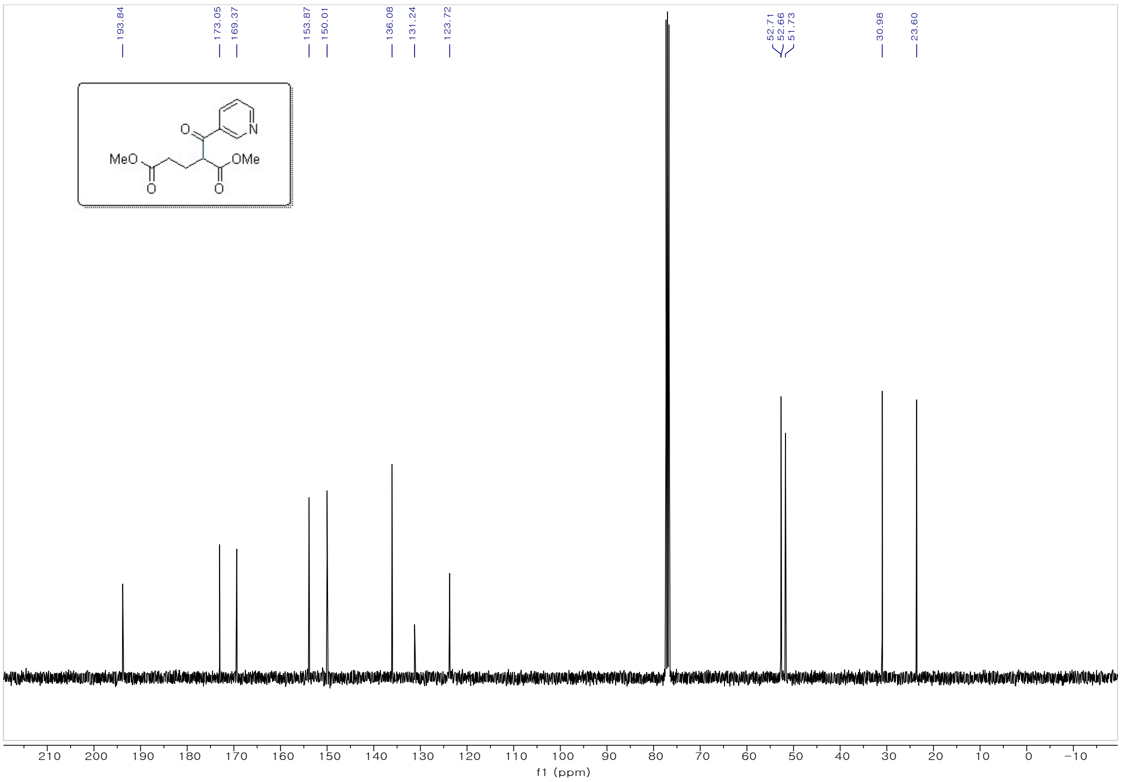

dimethyl 2-(2-oxo-2H-chromene-6-carbonyl)pentanedioate (3ax).

600 MHz, ^{1}H NMR in Chloroform-*d*

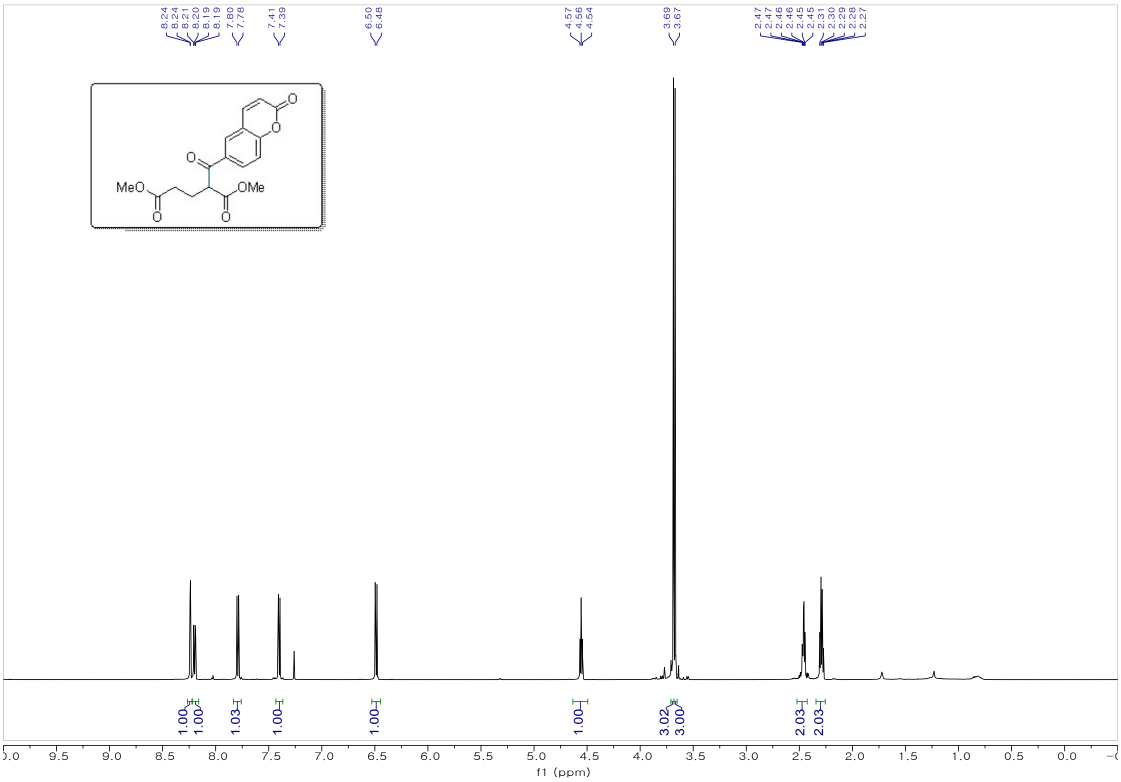

150 MHz, ^{13}C NMR in Chloroform-*d*

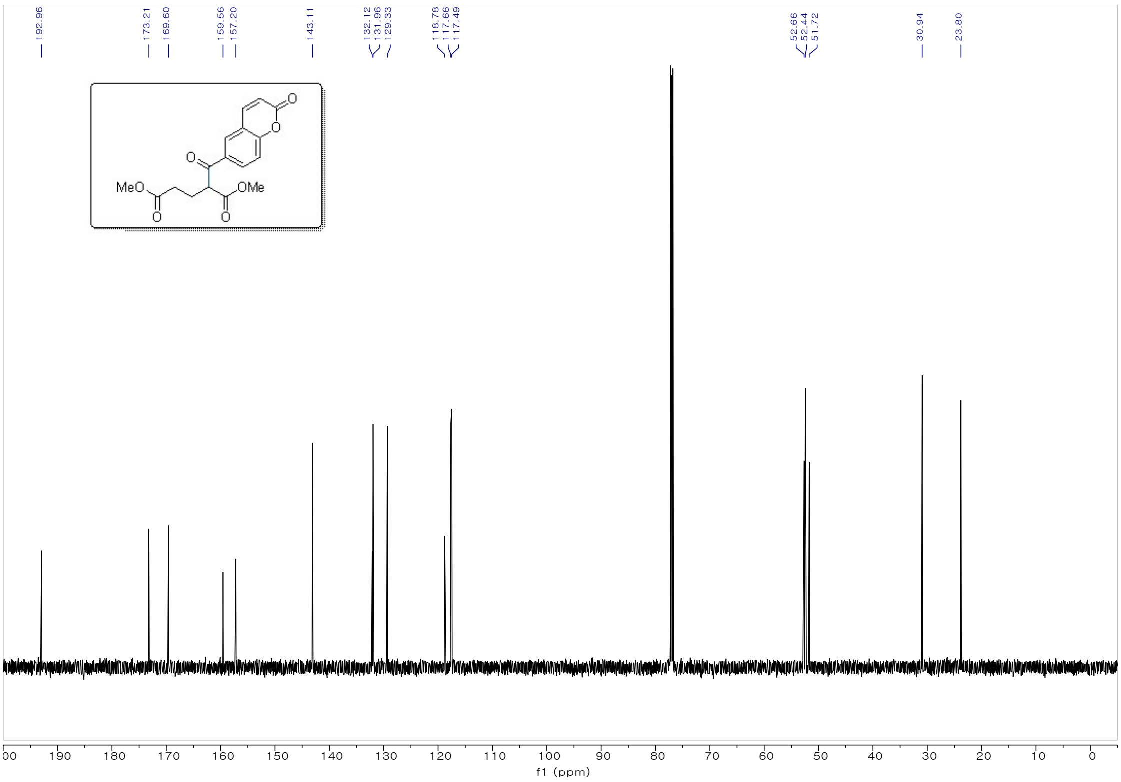

methyl 2-benzyl-4-(naphthalen-2-yl)-5-oxo-5-phenylpentanoate (5a).

600 MHz, ^{1}H NMR in Chloroform-*d*

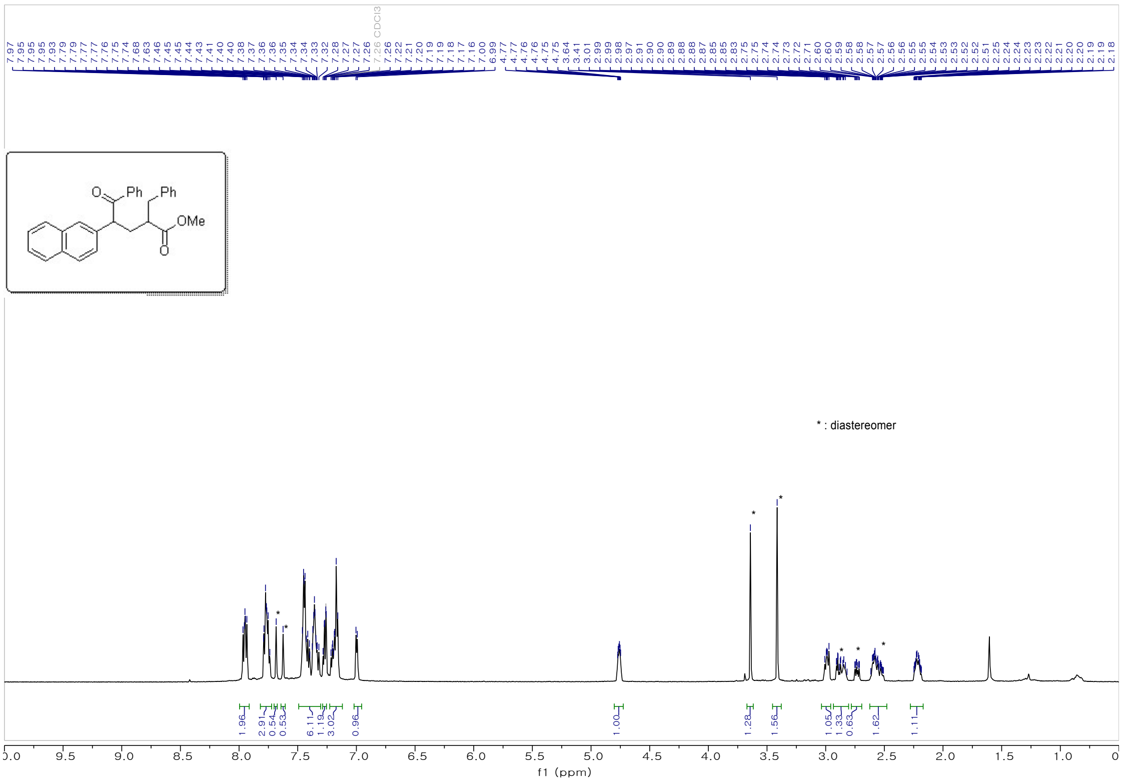

100 MHz, ^{13}C NMR in Chloroform-*d*

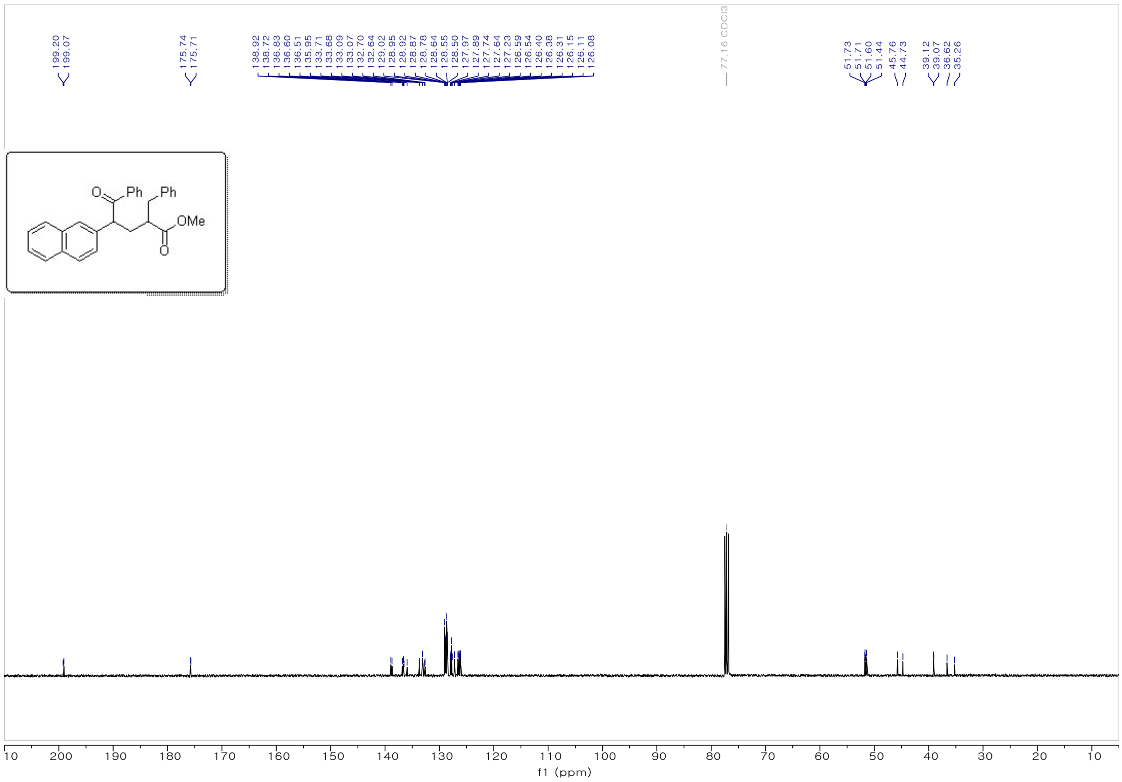

ethyl 4-(naphthalen-2-yl)-5-oxo-5-phenyl-2-propylpentanoate (5b).

400 MHz, ^{1}H NMR in Chloroform-*d*

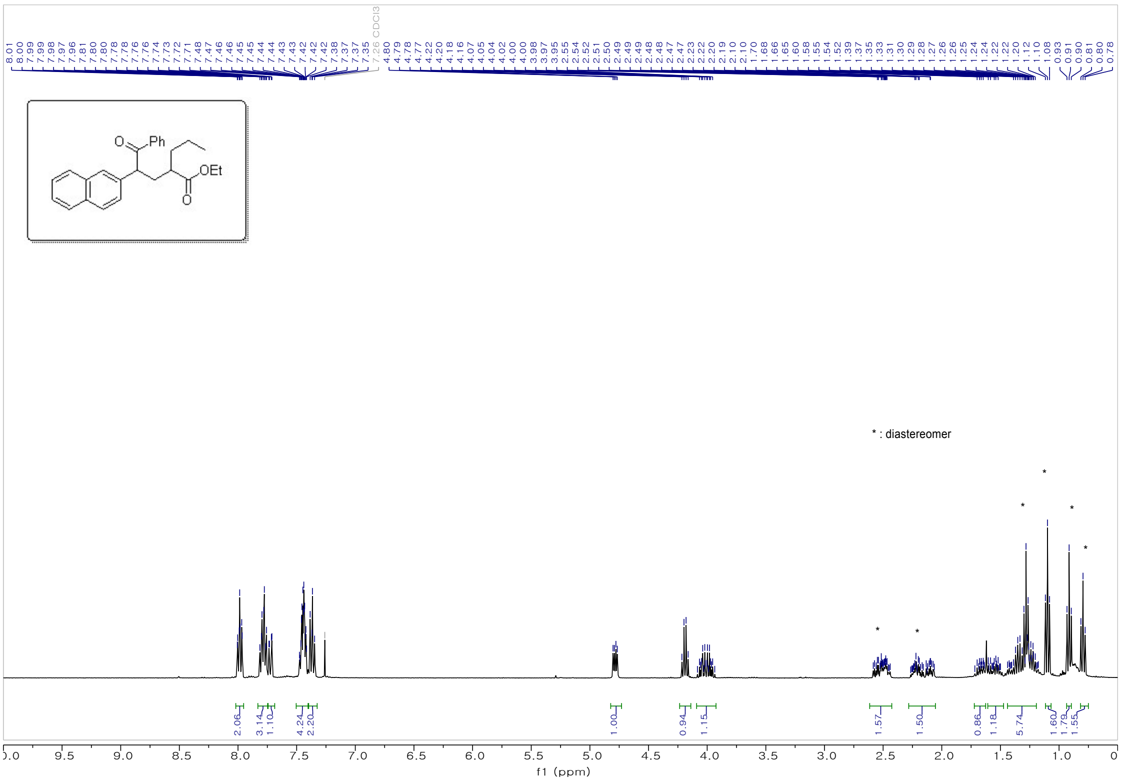

100 MHz, ^{13}C NMR in Chloroform-*d*

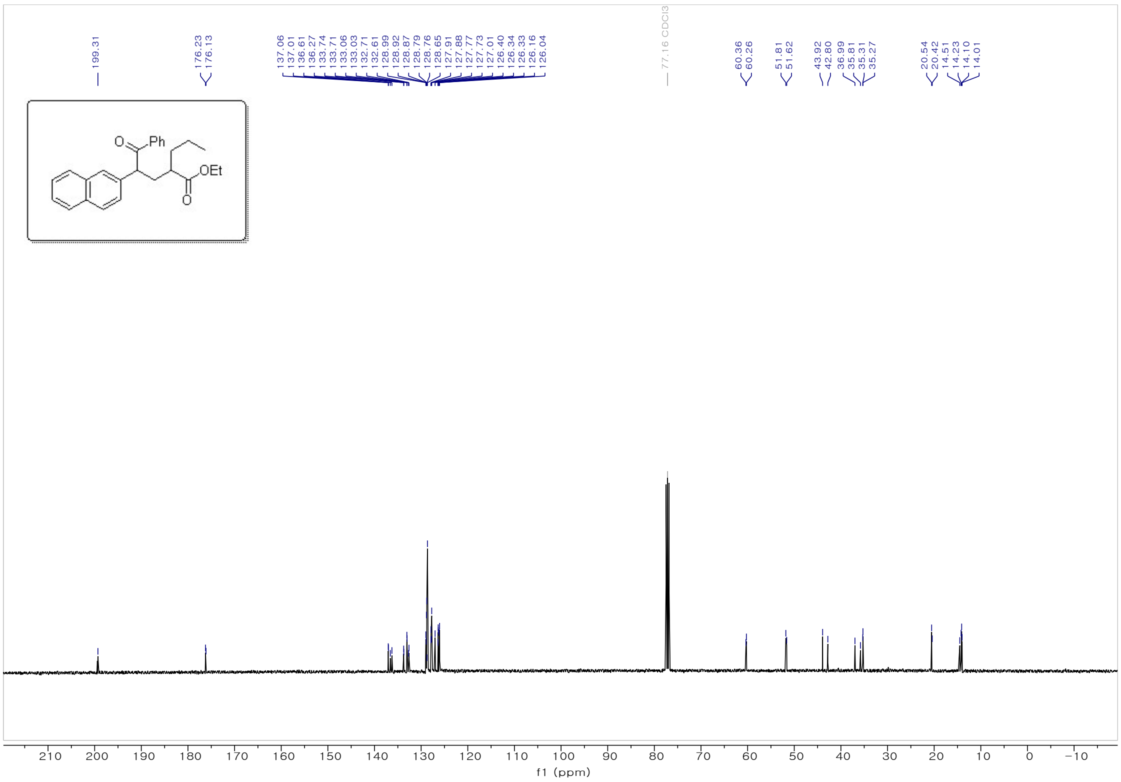

dimethyl 2-(2-(naphthalen-2-yl)-3-oxo-3-phenylpropyl)pentanedioate (5c).

400 MHz, ^{1}H NMR in Chloroform-*d*

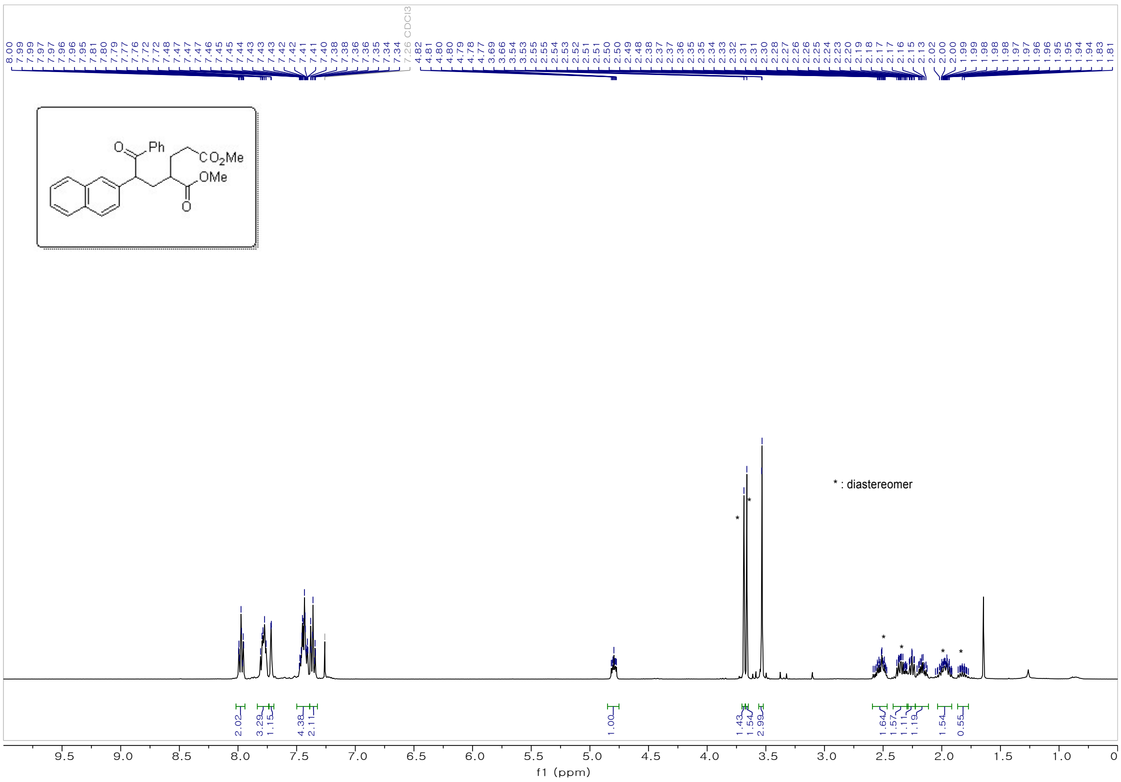

100 MHz, ^{13}C NMR in Chloroform-*d*

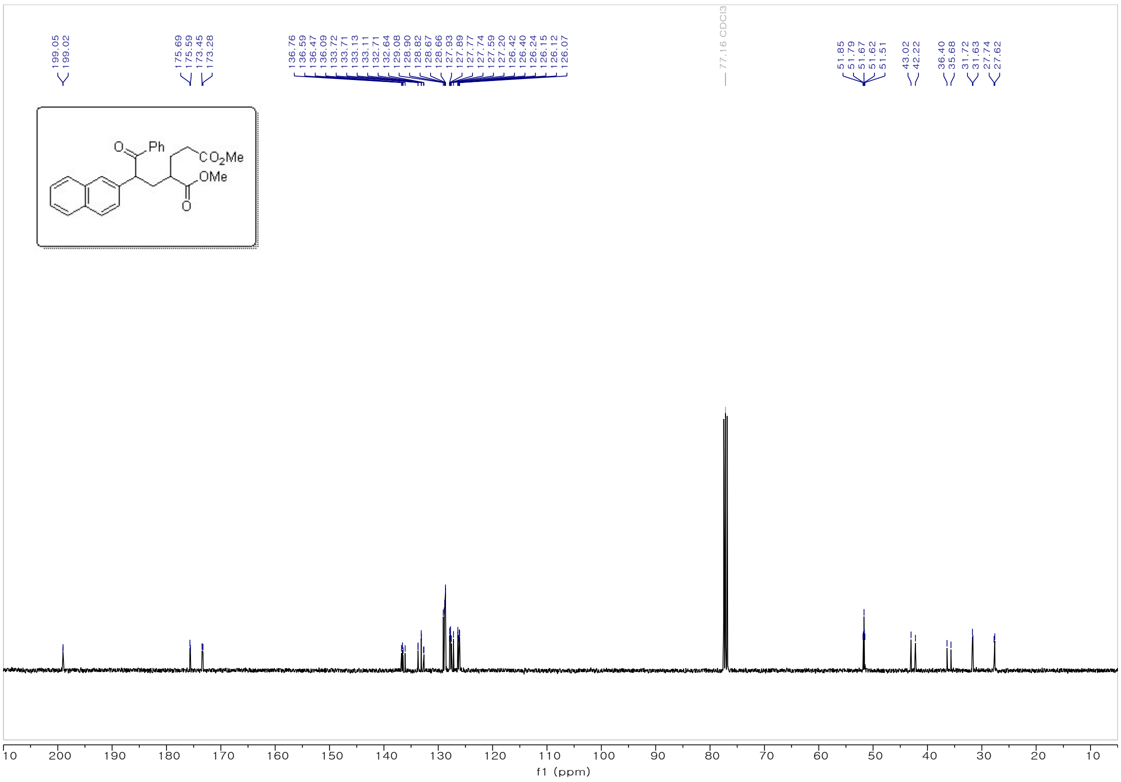

methyl 2-(2-(methylthio)ethyl)-4-(naphthalen-2-yl)-5-oxo-5-phenylpentanoate (5d).

600 MHz, ^{1}H NMR in Methylene Chloride-d_2

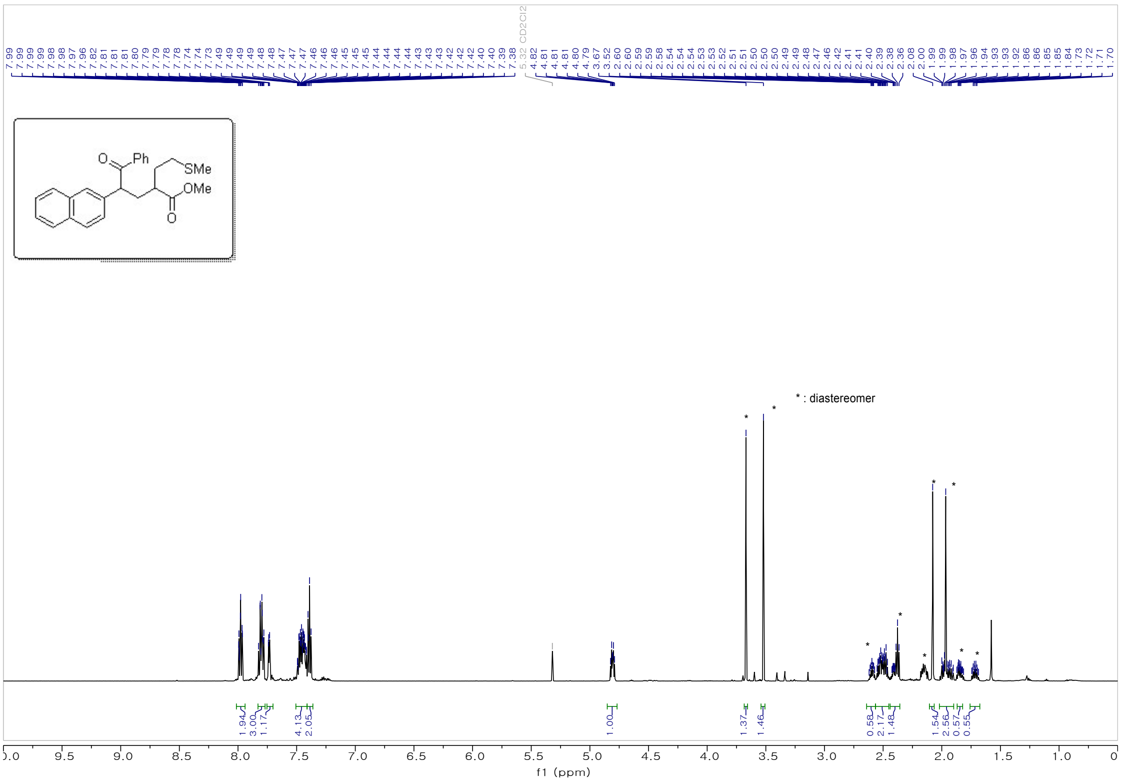

150 MHz, ^{13}C NMR in Methylene Chloride-d_2

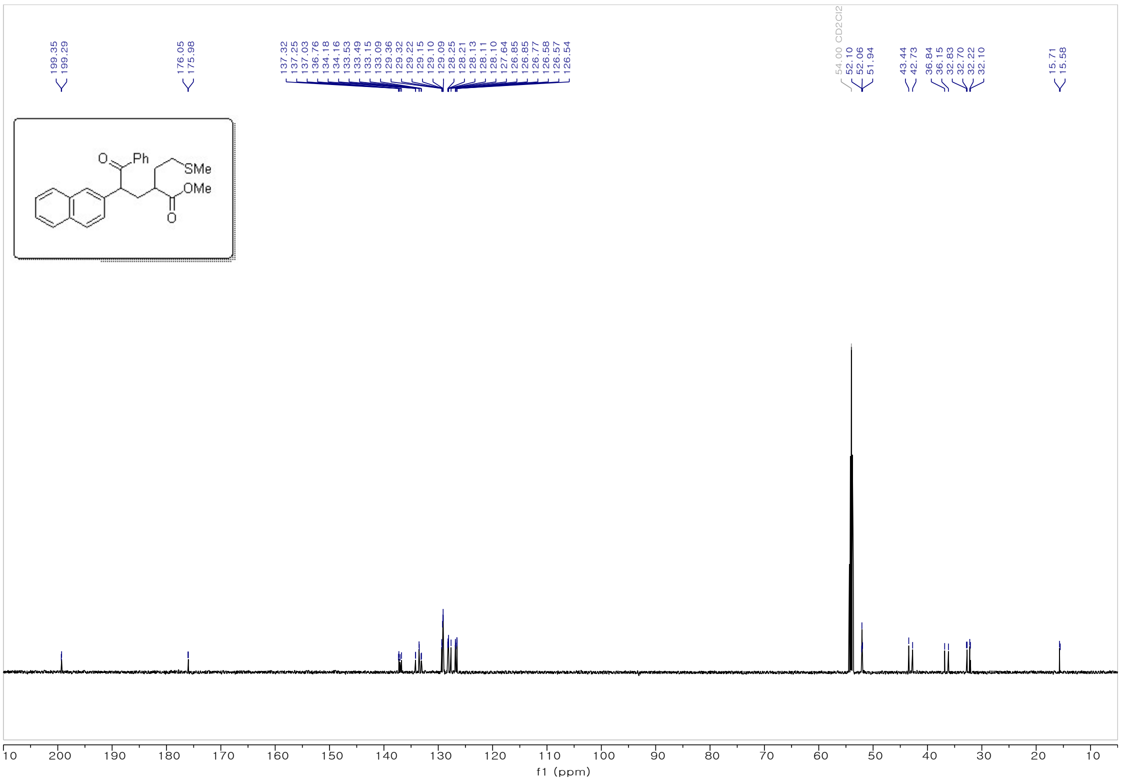

methyl 6-(((benzyloxy)carbonyl)amino)-2-(2-(naphthalen-2-yl)-3-oxo-3-phenylpropyl)hexanoate (5e).

600 MHz, ^{1}H NMR in Acetonitrile-d_3

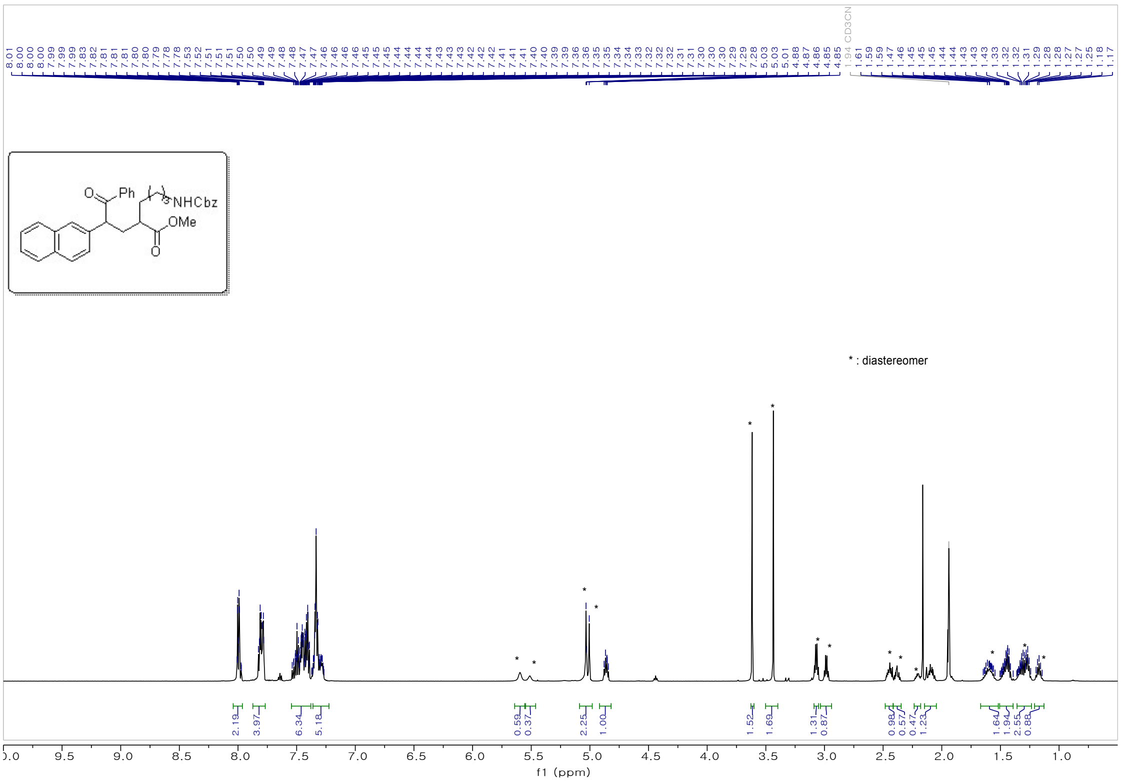

150 MHz, ^{13}C NMR in Acetonitrile-d_3

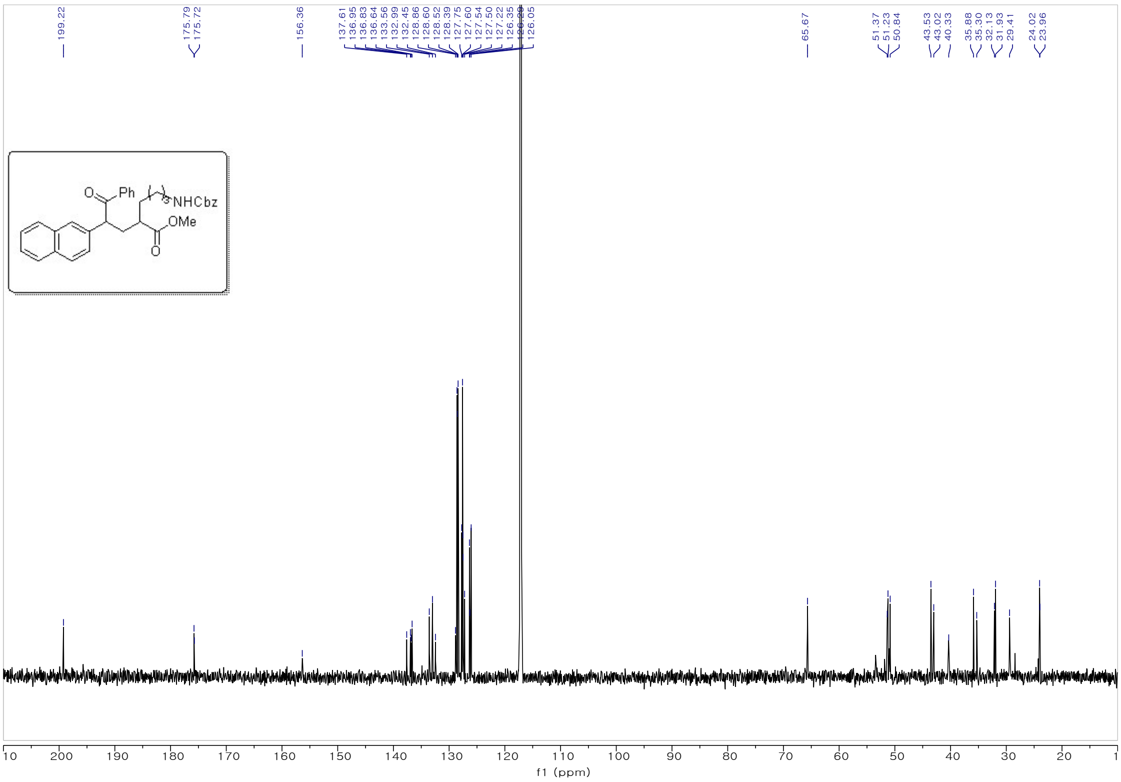

dimethyl 2-(2-(naphthalen-2-yl)-3-oxo-3-(thiophen-2-yl)propyl)pentanedioate (5f).

600 MHz, ^{1}H NMR in Chloroform-*d*

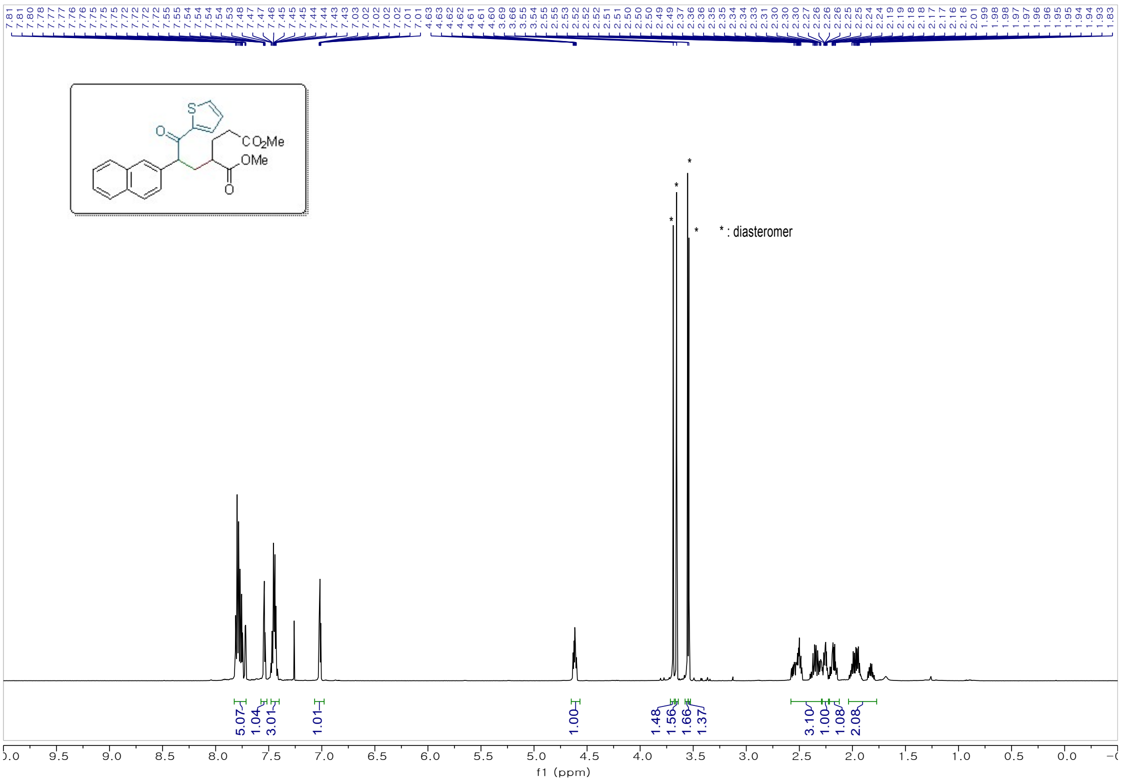

150 MHz, ^{13}C NMR in Chloroform-*d*

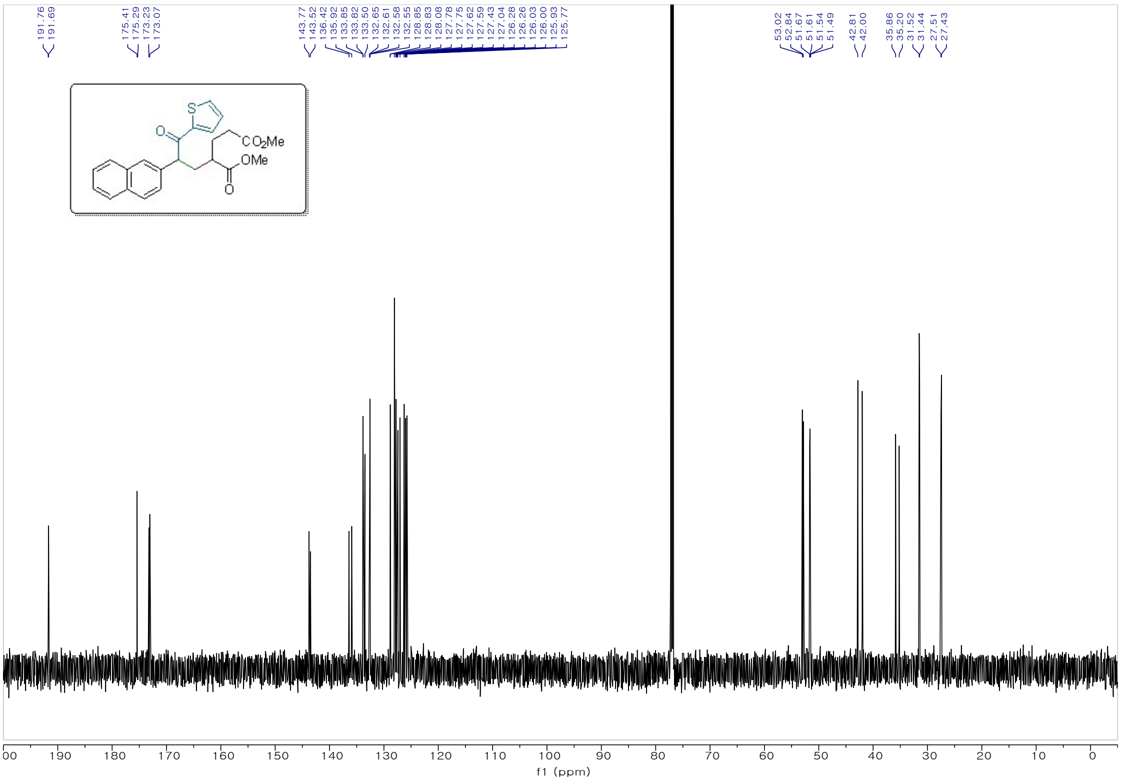

dimethyl 2-(3-(4-fluorophenyl)-2-(naphthalen-2-yl)-3-oxopropyl)pentanedioate (5g).

600 MHz, ^{1}H NMR in Chloroform-*d*

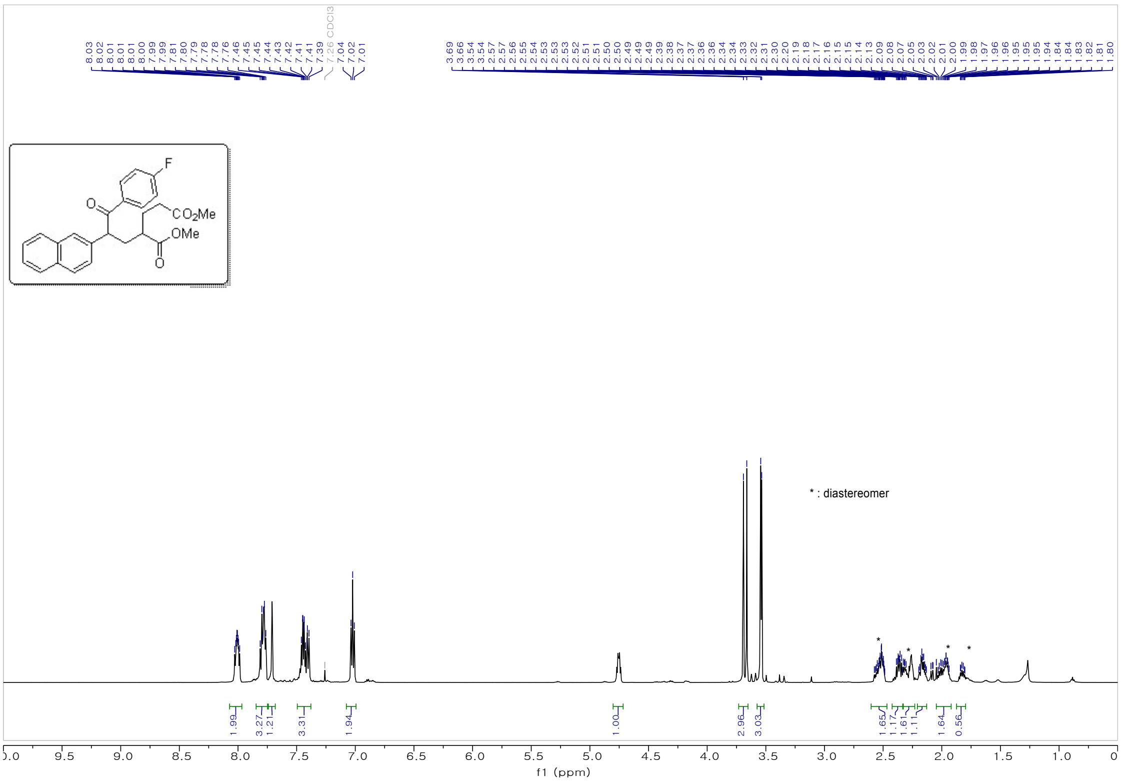

150 MHz, ^{13}C NMR in Chloroform-*d*

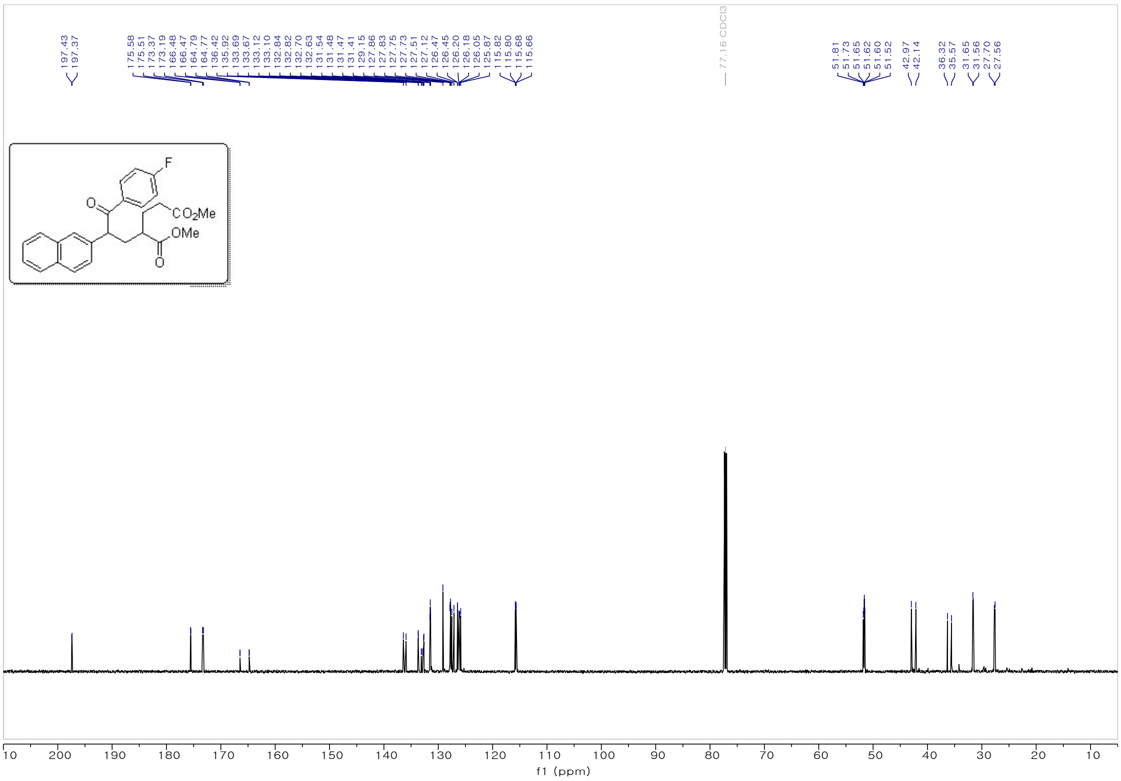

375 MHz, ^{19}F NMR in Chloroform-*d*

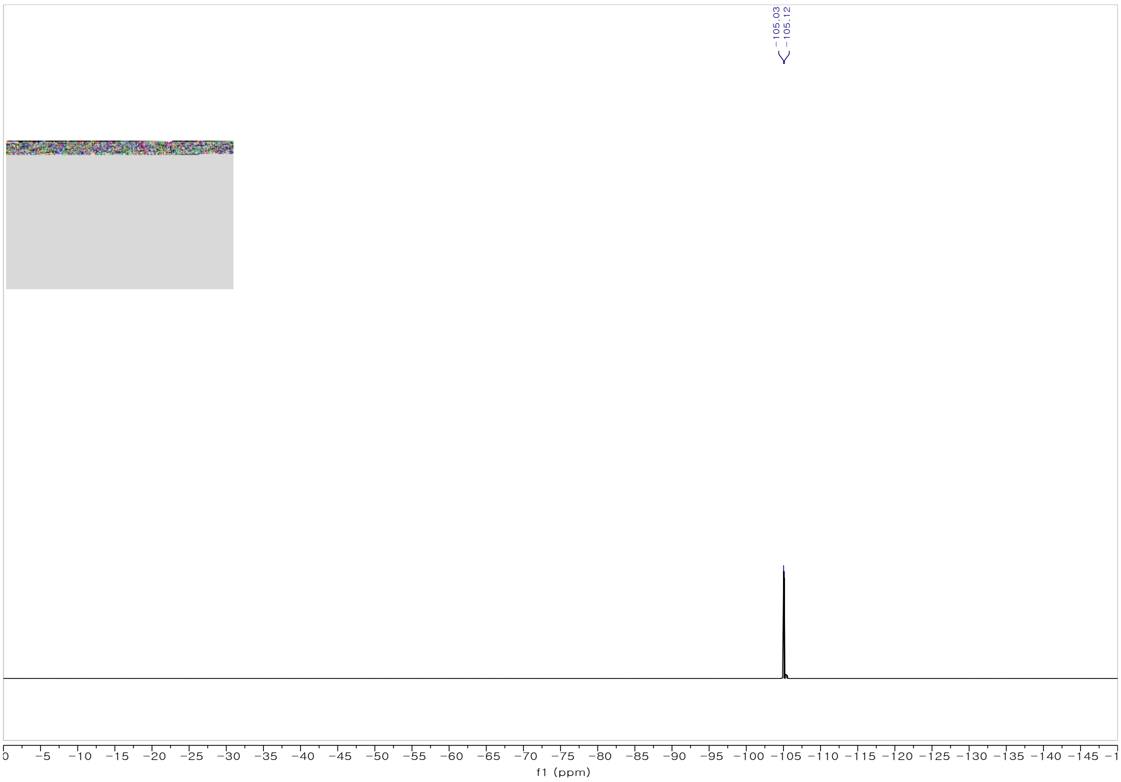

dimethyl 2-(3-(4-chlorophenyl)-2-(naphthalen-2-yl)-3-oxopropyl)pentanedioate (5h).

600 MHz, ^{1}H NMR in Chloroform-*d*

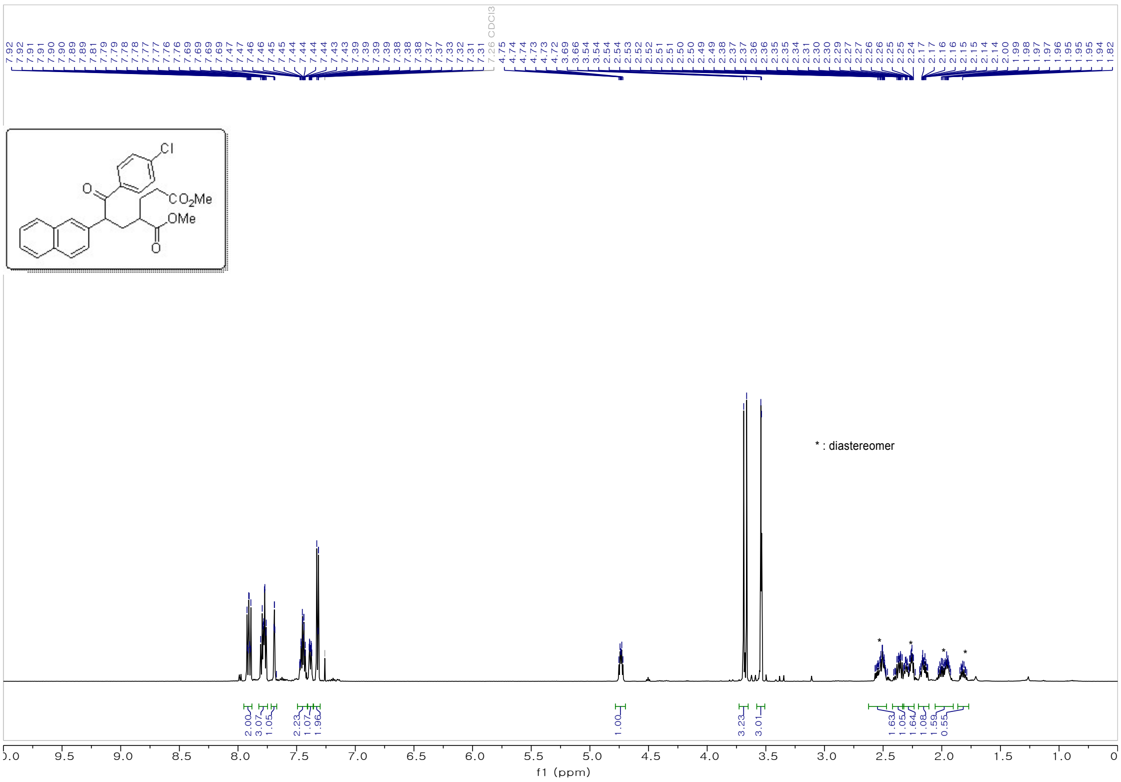

150 MHz, ^{13}C NMR in Chloroform-*d*

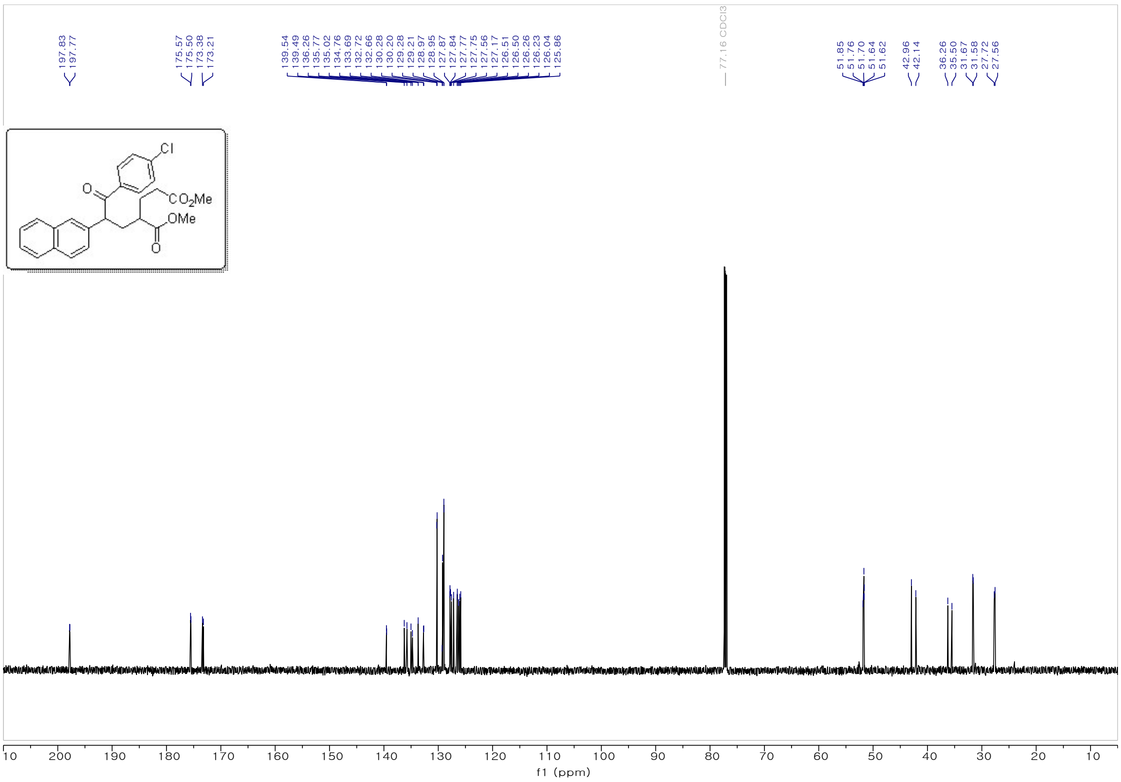

dimethyl 2-(3-(4-bromophenyl)-2-(naphthalen-2-yl)-3-oxopropyl)pentanedioate (5i).

600 MHz, ^{1}H NMR in Chloroform-*d*

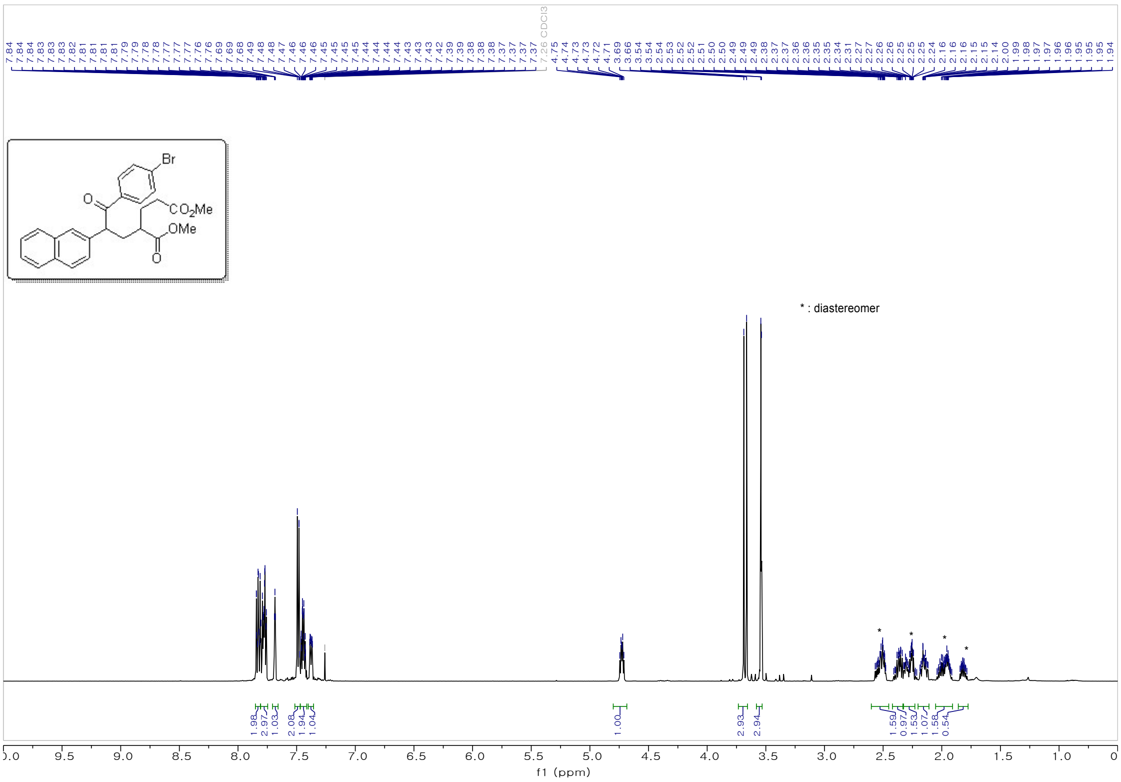

150 MHz, ^{13}C NMR in Chloroform-*d*

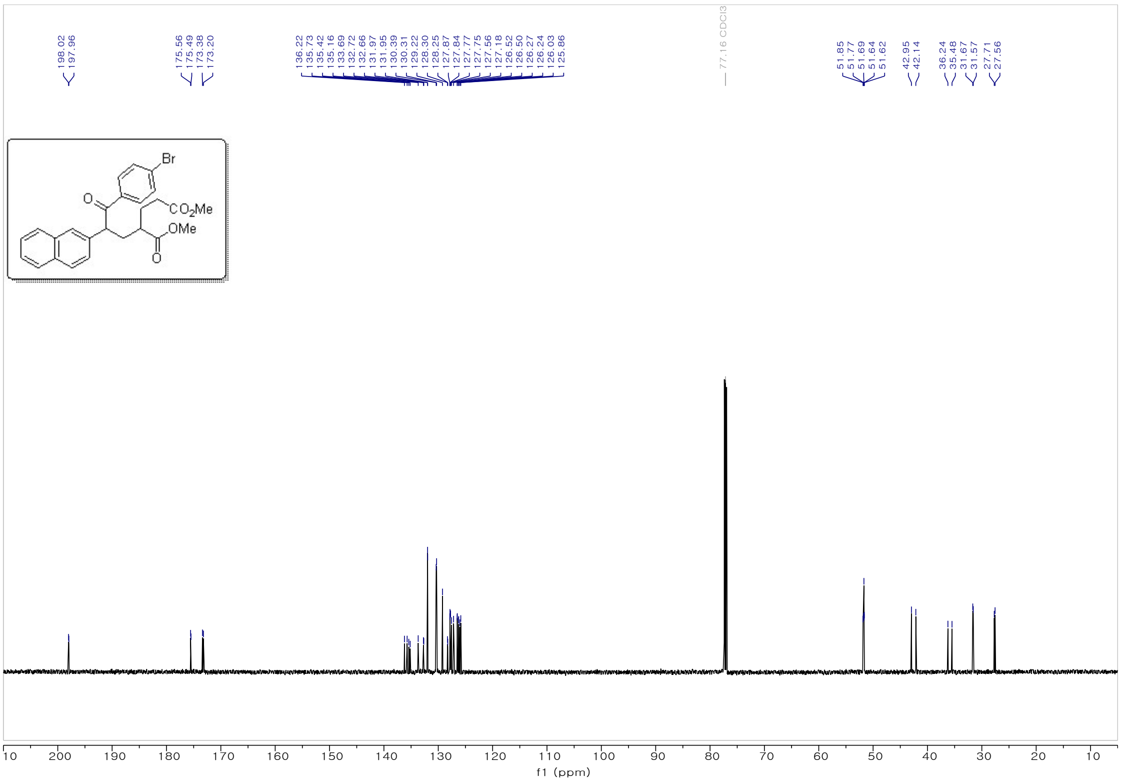

dimethyl 2-(2-(naphthalen-2-yl)-3-oxo-3-(p-tolyl)propyl)pentanedioate (5j).

600 MHz, ^{1}H NMR in Chloroform-*d*

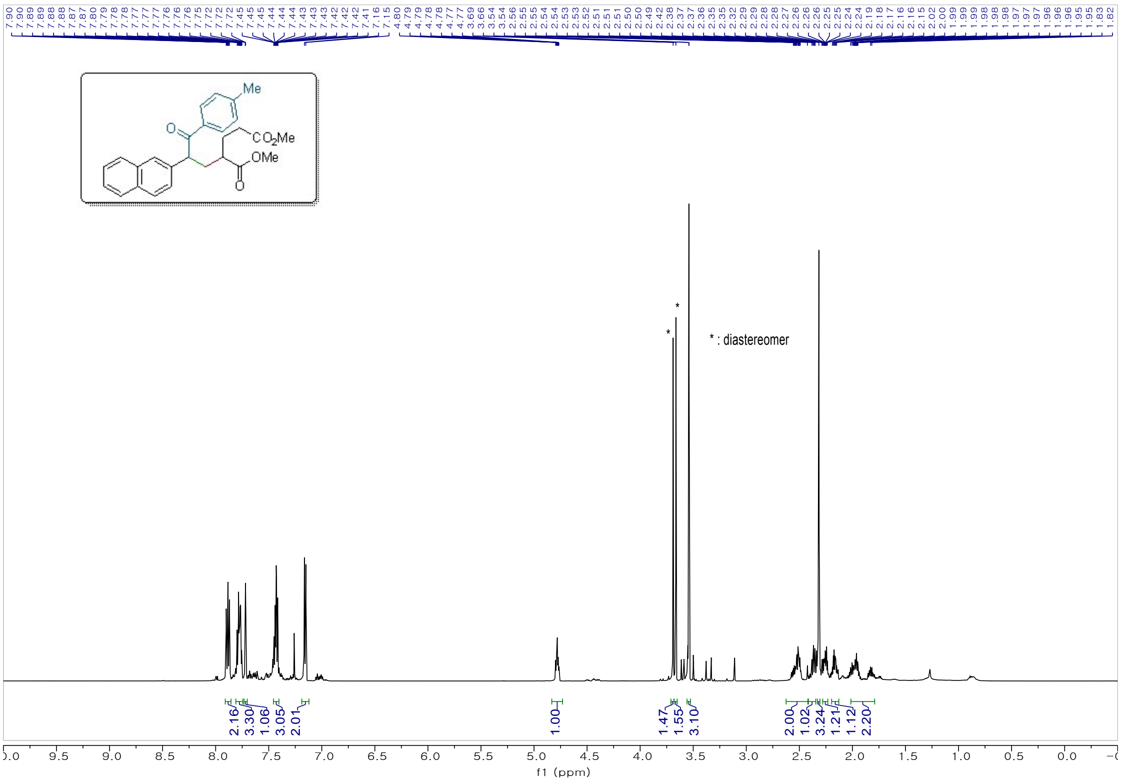

150 MHz, ^{13}C NMR in Chloroform-*d*

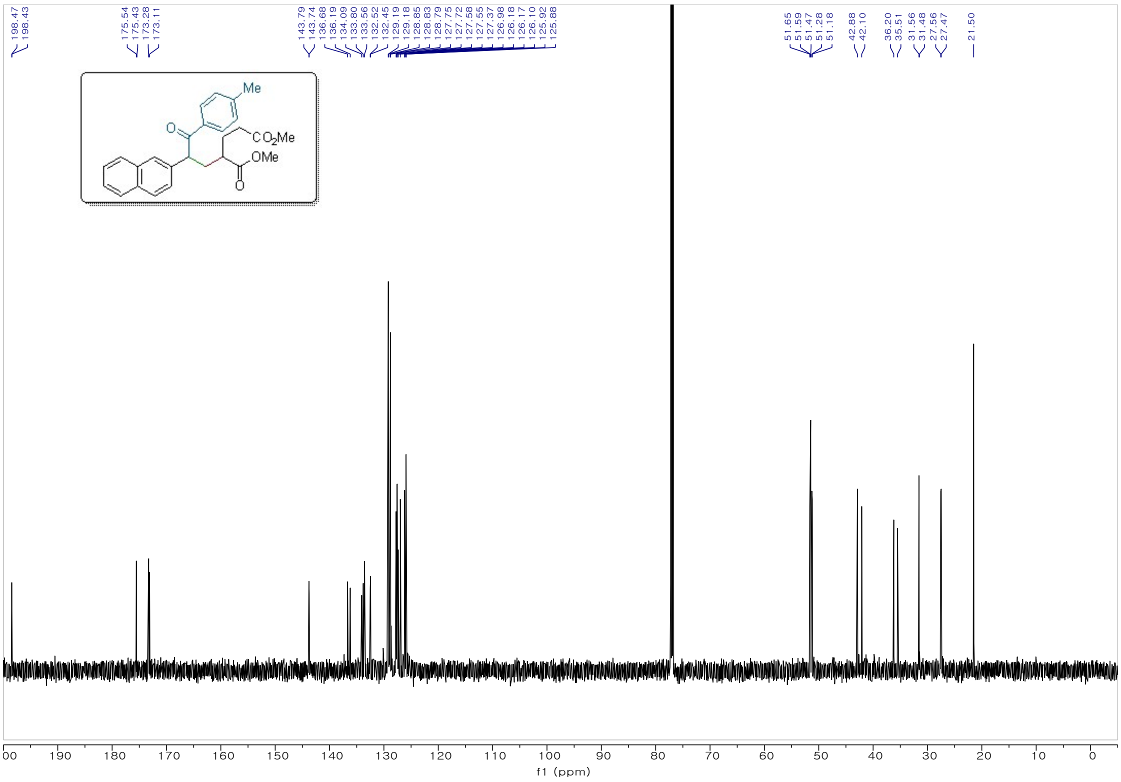

dimethyl 2-(3-oxo-2,3-diphenylpropyl)pentanedioate (5k).

400 MHz, ^{1}H NMR in Chloroform-*d*

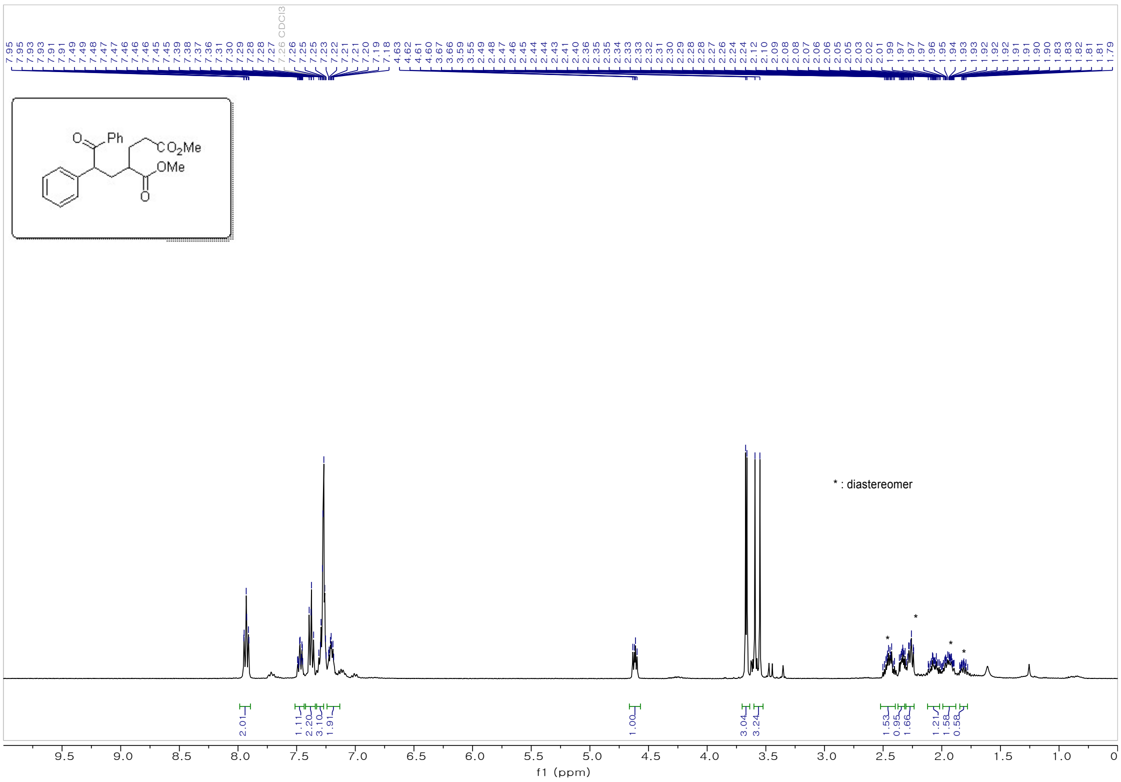

100 MHz, ^{13}C NMR in Chloroform-*d*

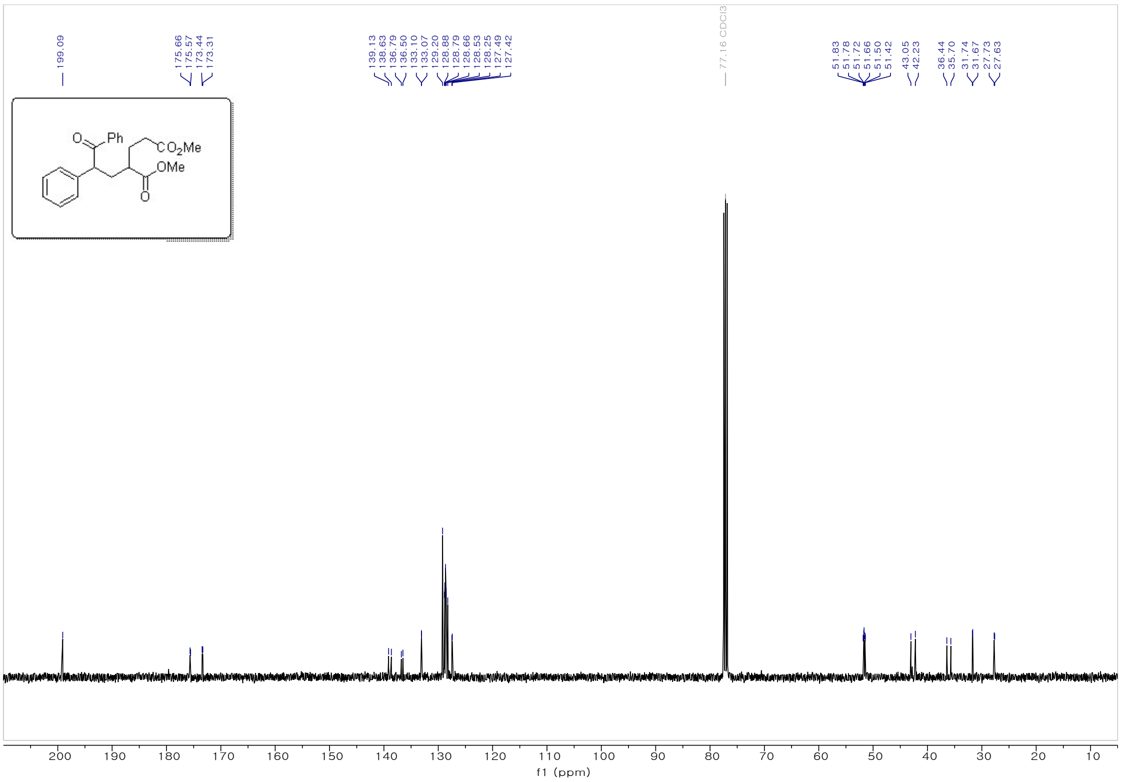

dimethyl 2-(3-oxo-3-phenyl-2-(p-tolyl)propyl)pentanedioate (5l).

600 MHz, ^{1}H NMR in Chloroform-*d*

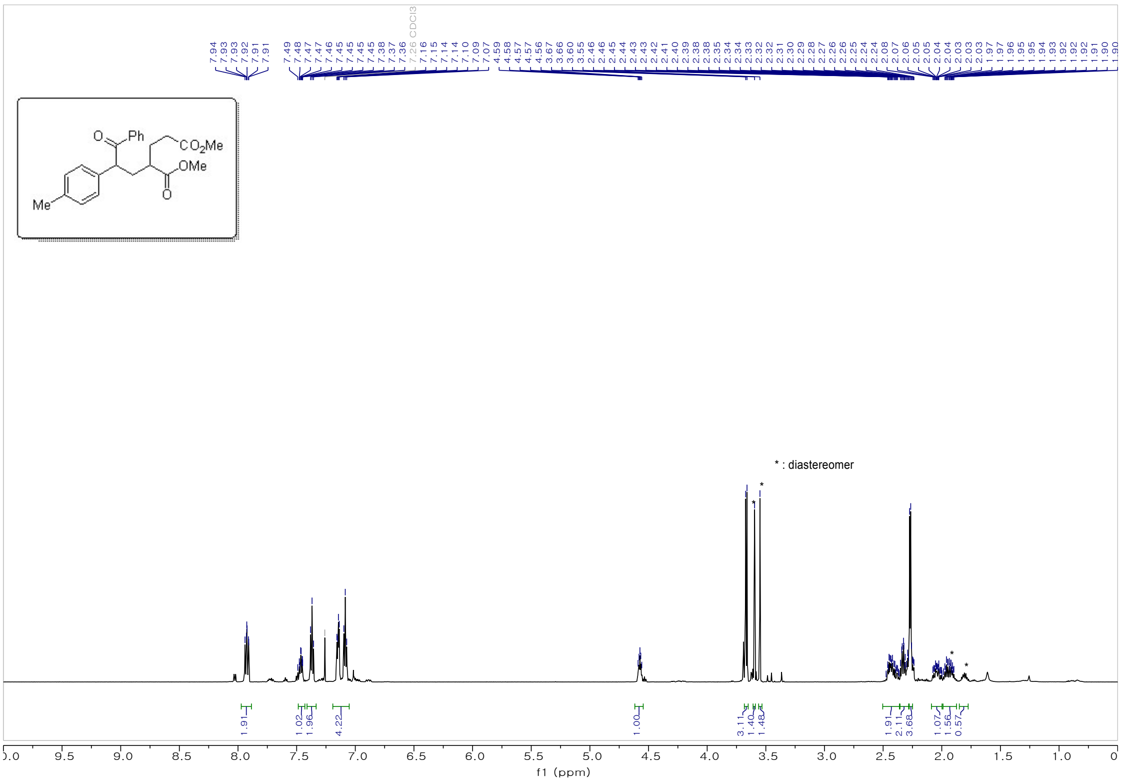

150 MHz, ^{13}C NMR in Chloroform-*d*

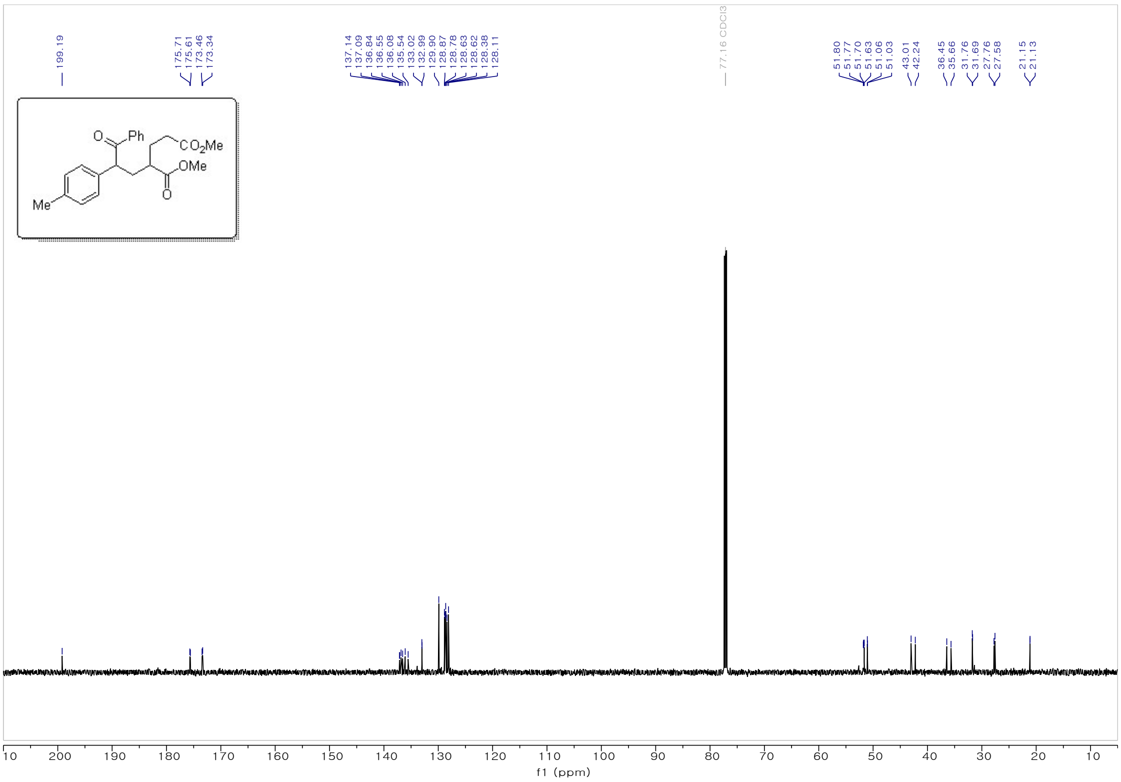

dimethyl 2-(2-(4-chlorophenyl)-3-oxo-3-phenylpropyl)pentanedioate (5m).

400 MHz, ^{1}H NMR in Chloroform-*d*

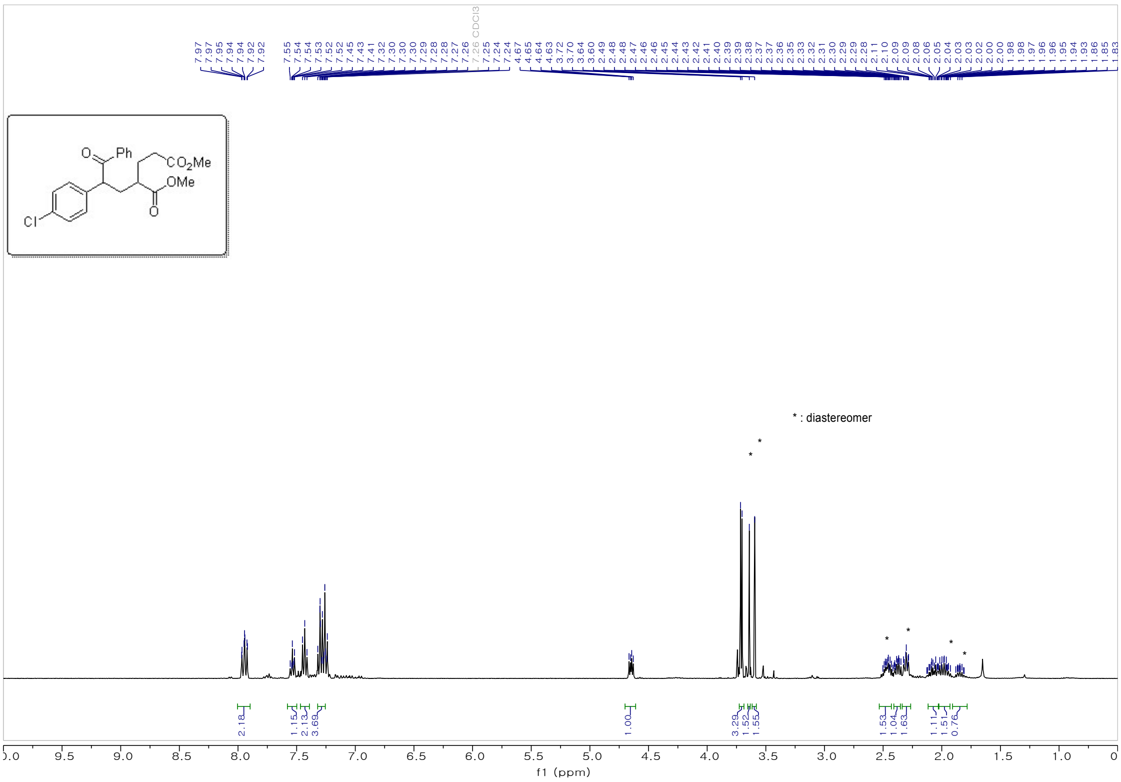

150 MHz, ^{13}C NMR in Chloroform-*d*

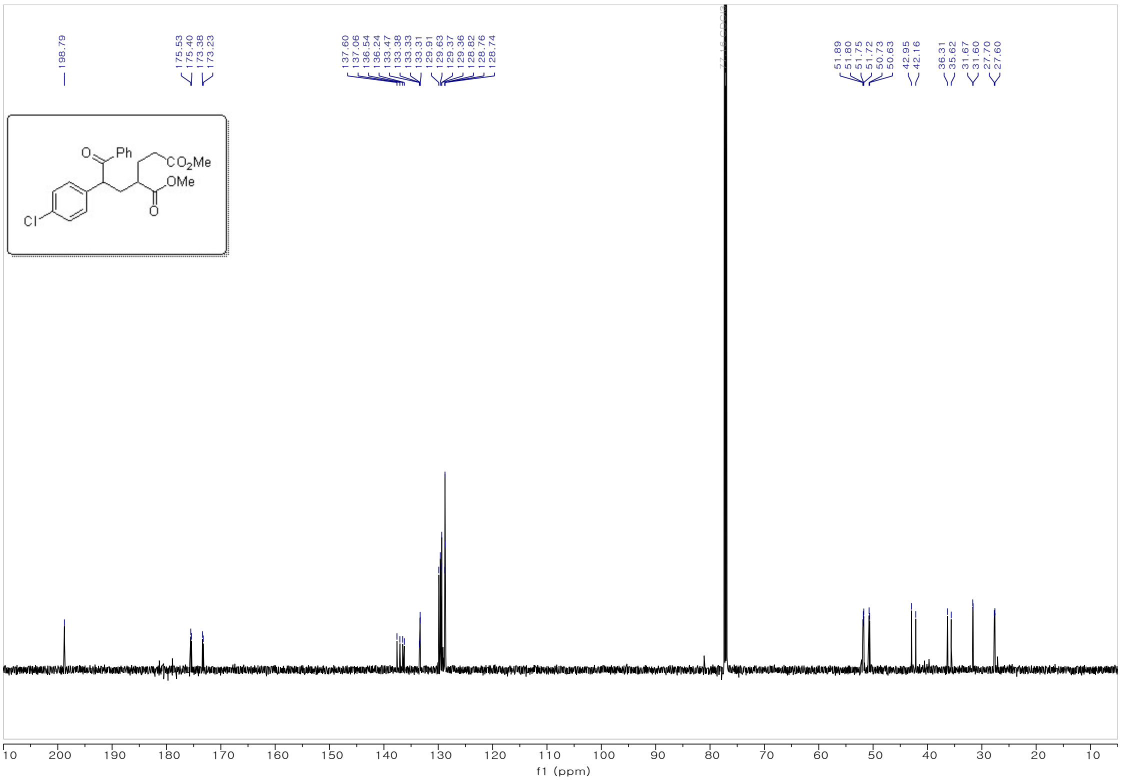

dimethyl 2-(3-oxo-3-phenyl-2-(4-(trifluoromethyl)phenyl)propyl)pentanedioate (5n).

600 MHz, ^{1}H NMR in Chloroform-*d*

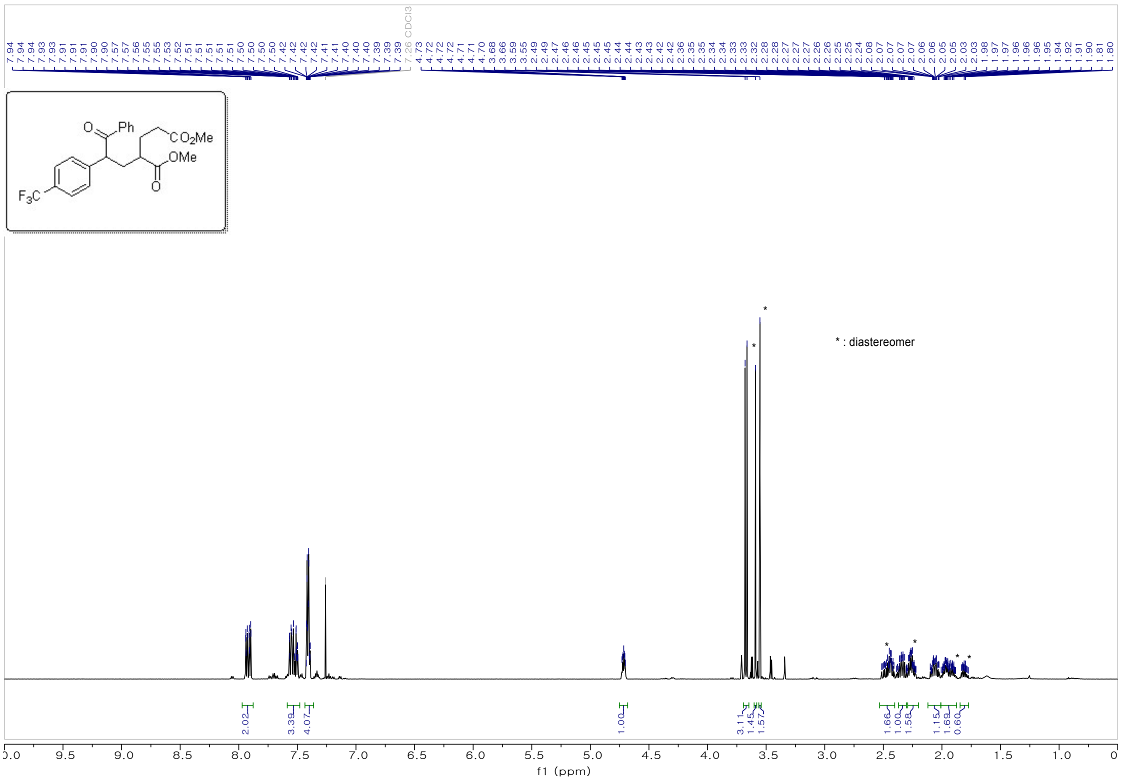

150 MHz, ^{13}C NMR in Chloroform-*d*

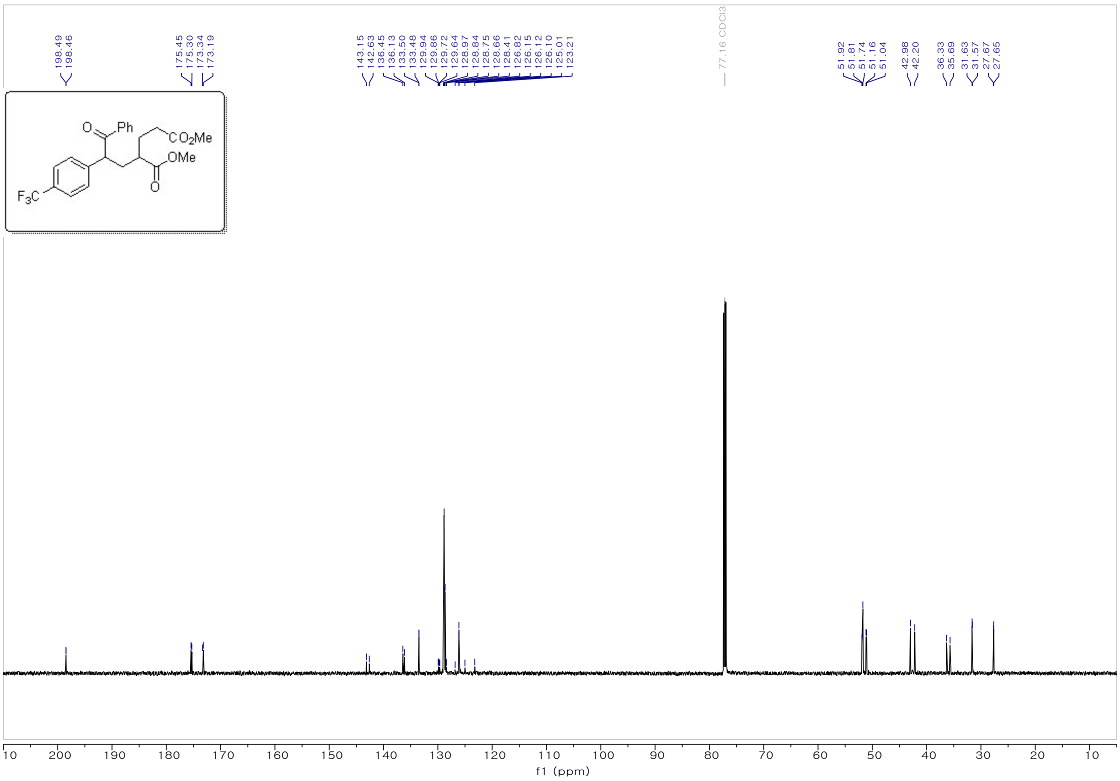

375 MHz, ^{19}F NMR in Chloroform-*d*

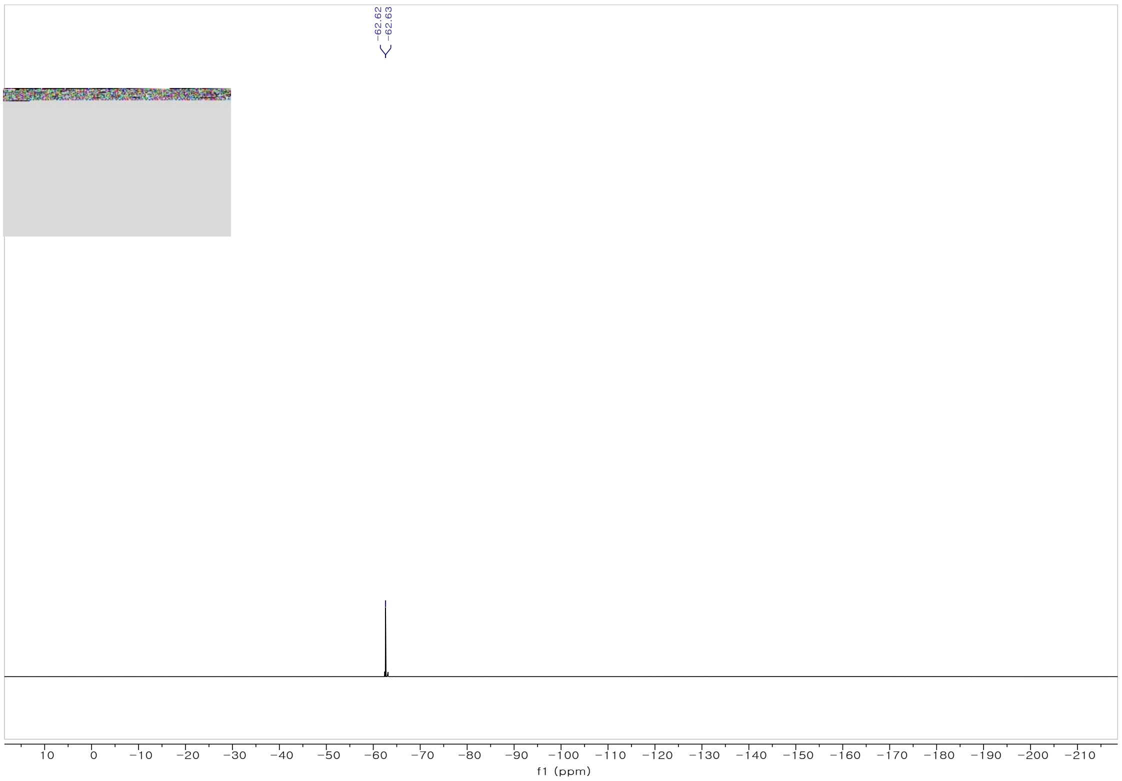

dimethyl 2-(2-(4-(methoxycarbonyl)phenyl)-3-oxo-3-phenylpropyl)pentanedioate (5o).

600 MHz, ^{1}H NMR in Chloroform-*d*

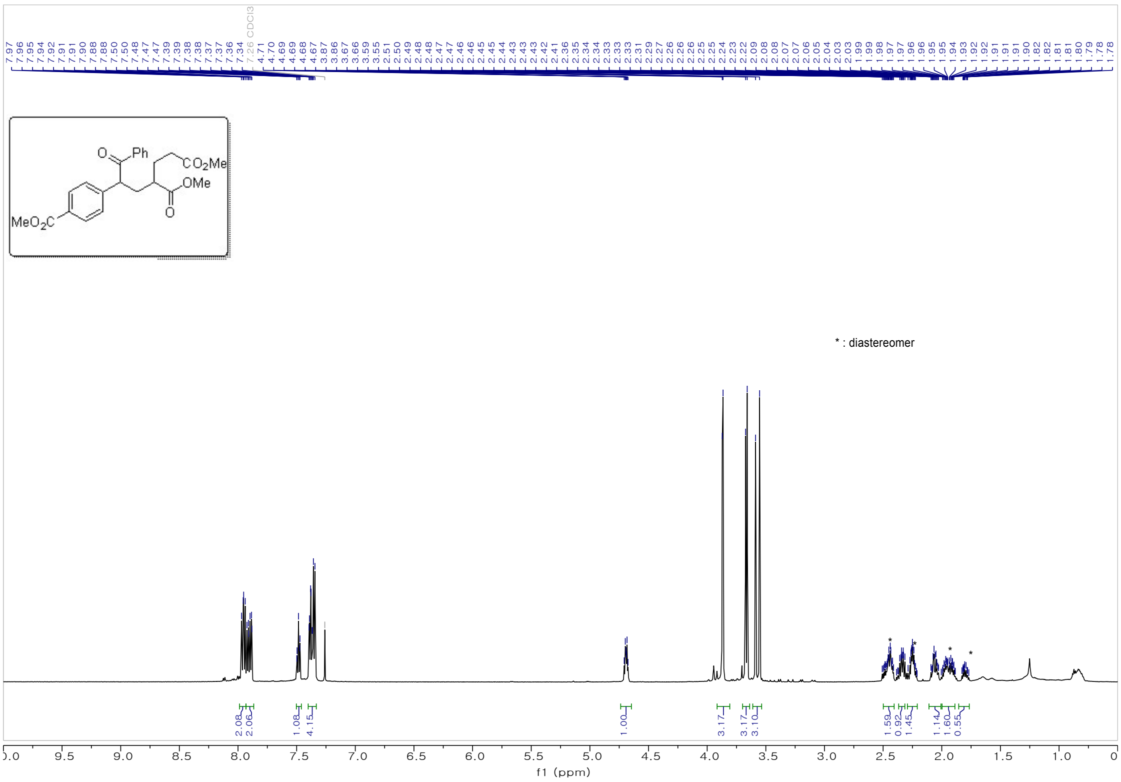

150 MHz, ^{13}C NMR in Chloroform-*d*

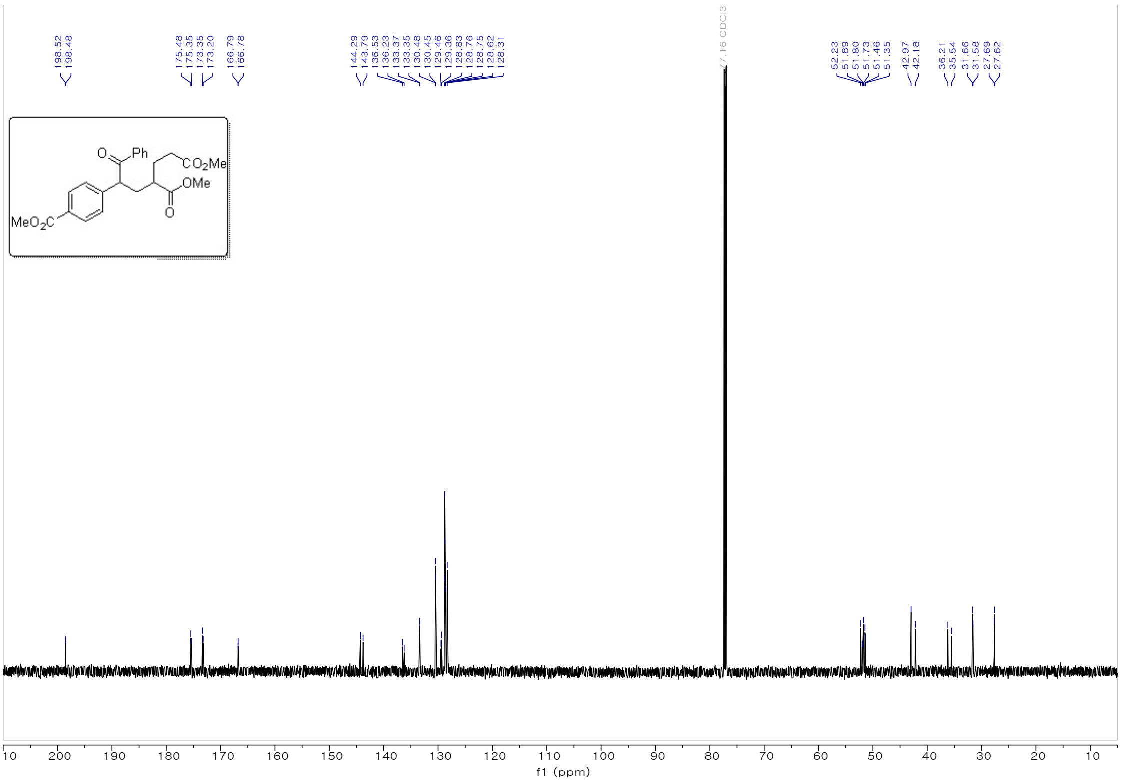

dimethyl 2-(2-(4-methoxyphenyl)-3-oxo-3-phenylpropyl)pentanedioate (5p).

600 MHz, ^{1}H NMR in Chloroform-*d*

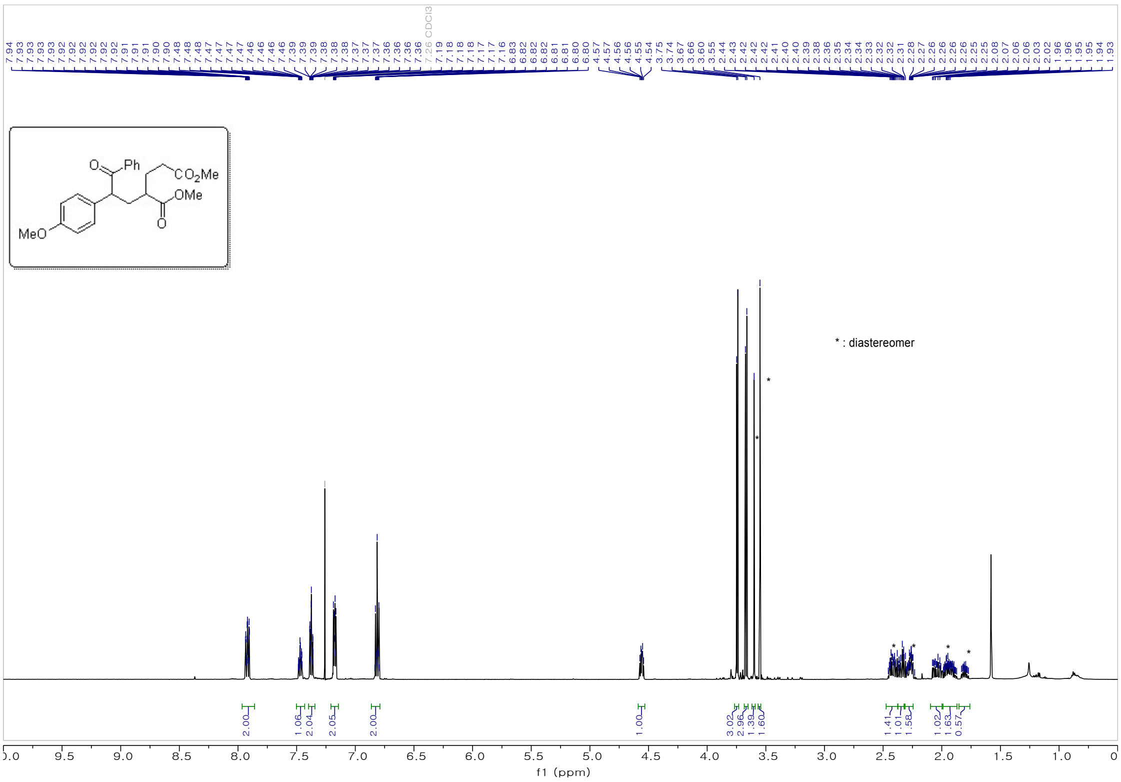

150 MHz, ^{13}C NMR in Chloroform-*d*

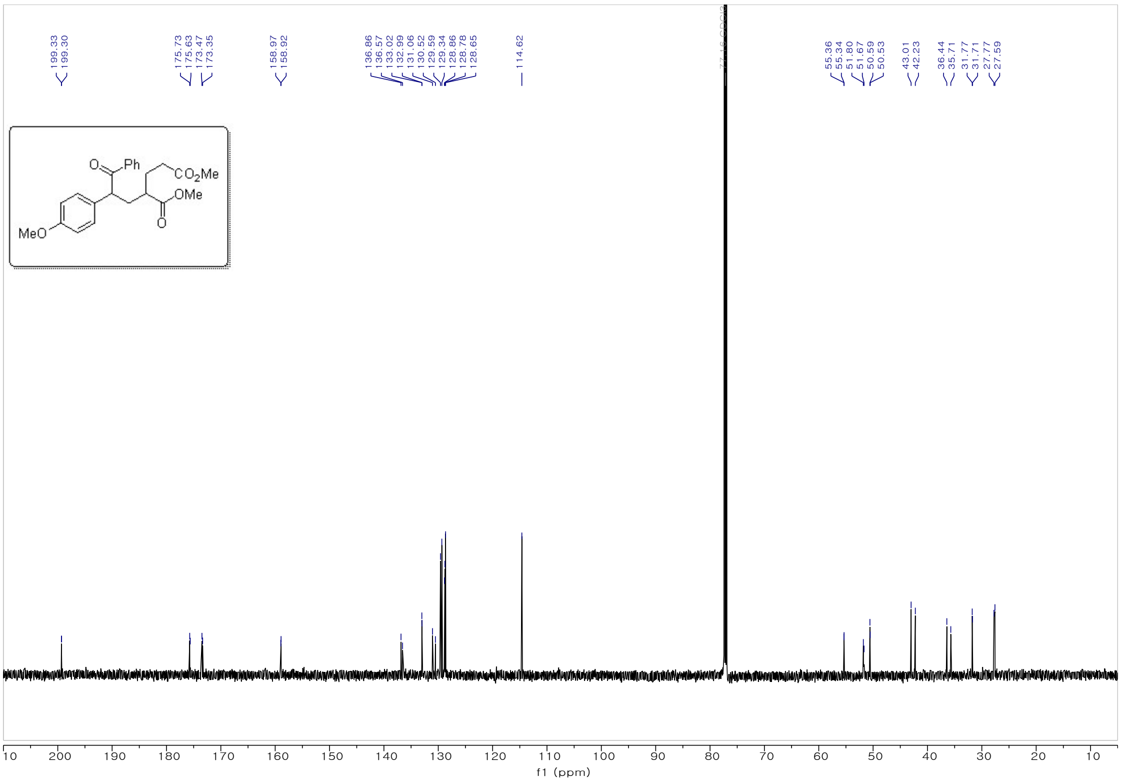

1-(1-(cinnamyloxy)-1-oxo-3-phenylpropan-2-yl)-2,4,6-triphenylpyridin-1-ium tetrafluoroborate (6).

600 MHz, ^{1}H NMR in Chloroform-*d*

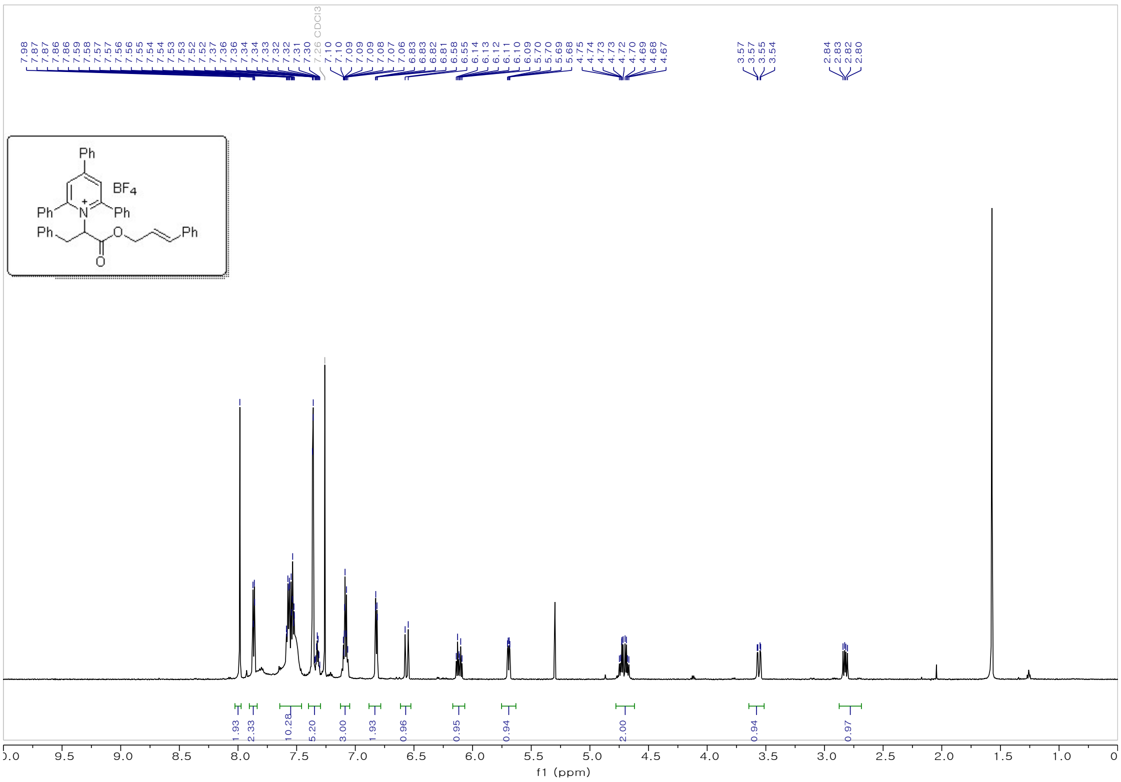

100 MHz, ^{13}C NMR in Chloroform-*d*

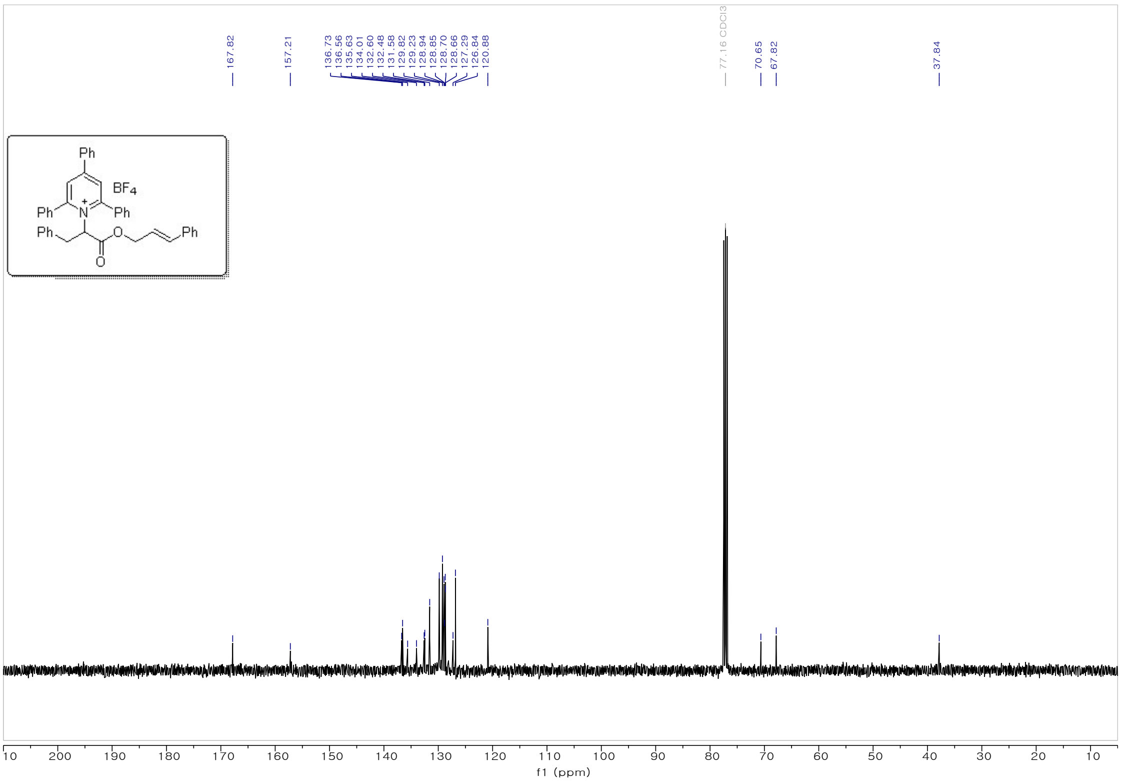

375 MHz, ^{19}F NMR in Chloroform-*d*

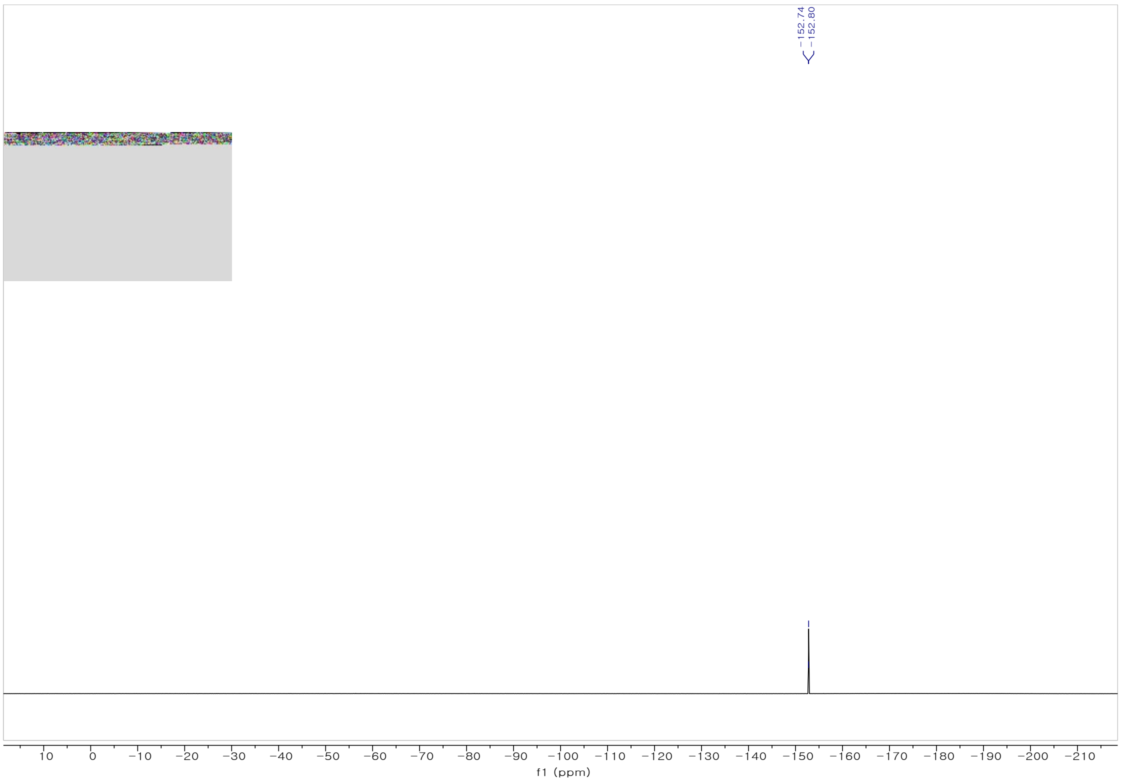

3-benzyl-4-(2-oxo-1,2-diphenylethyl)dihydrofuran-2(3H)-one (7).

600 MHz, ^{1}H NMR in Chloroform-*d*

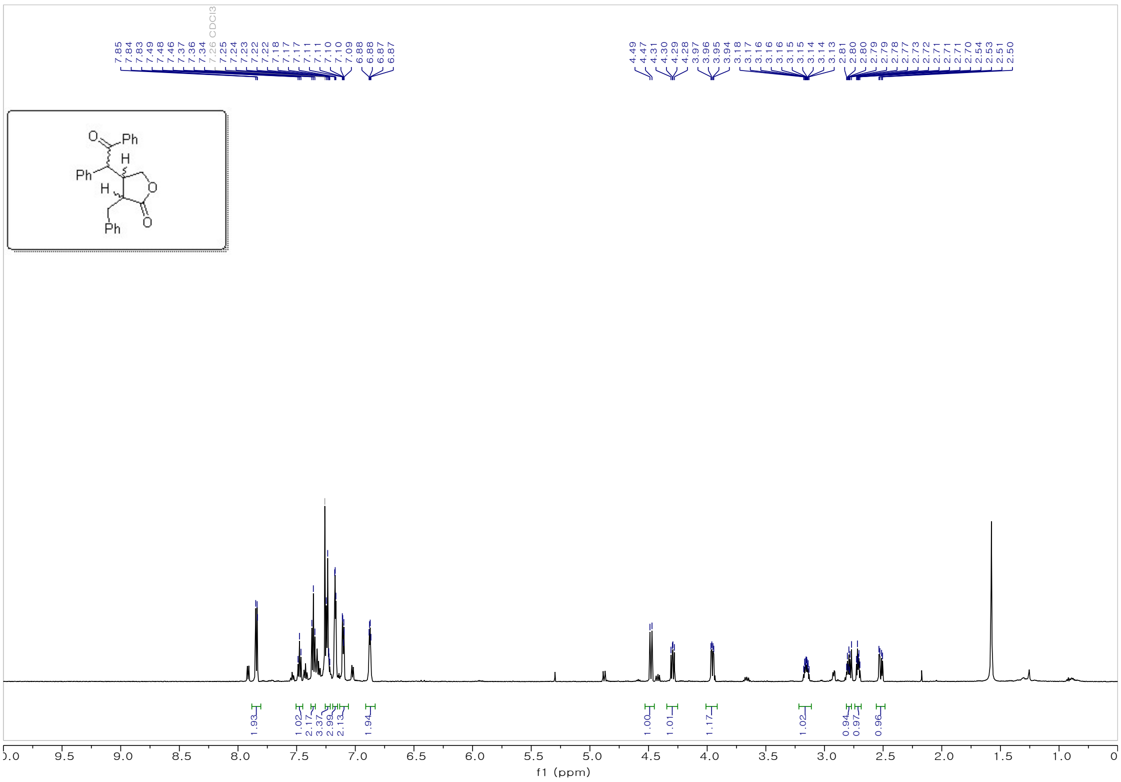

100 MHz, ^{13}C NMR in Chloroform-*d*

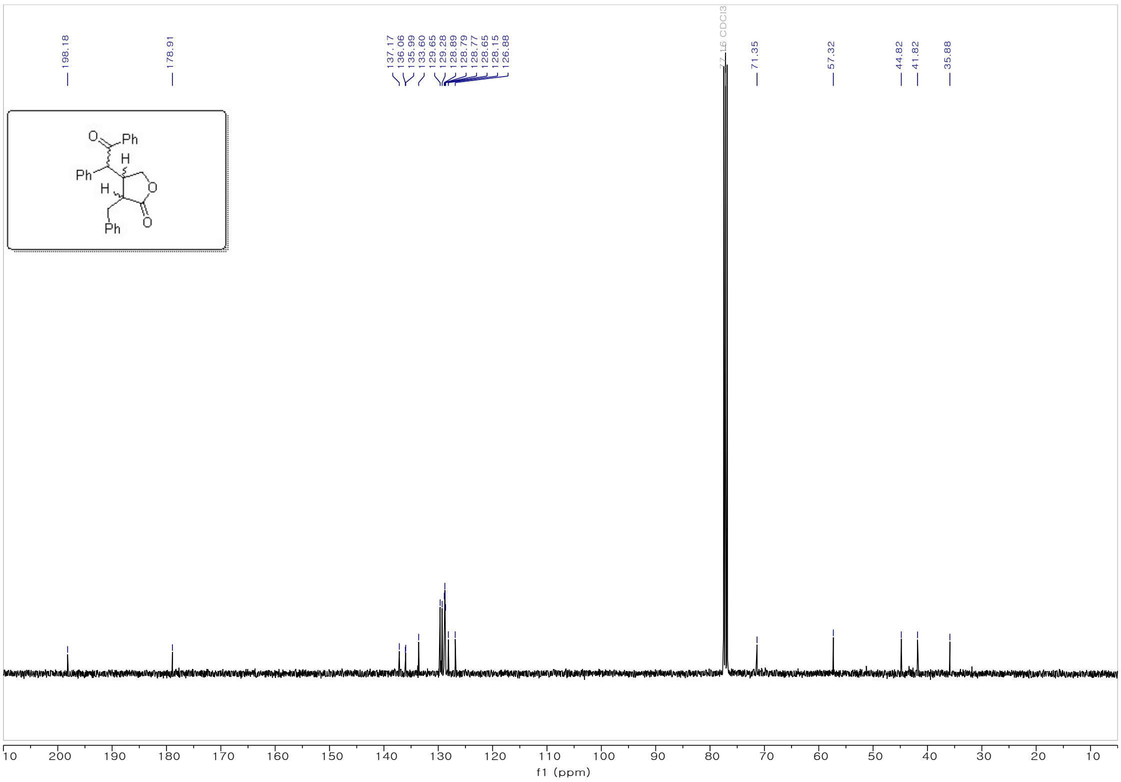

cinnamyl 2-benzyl-3-oxo-3-phenylpropanoate (8).

600 MHz, ^{1}H NMR in Chloroform-*d*

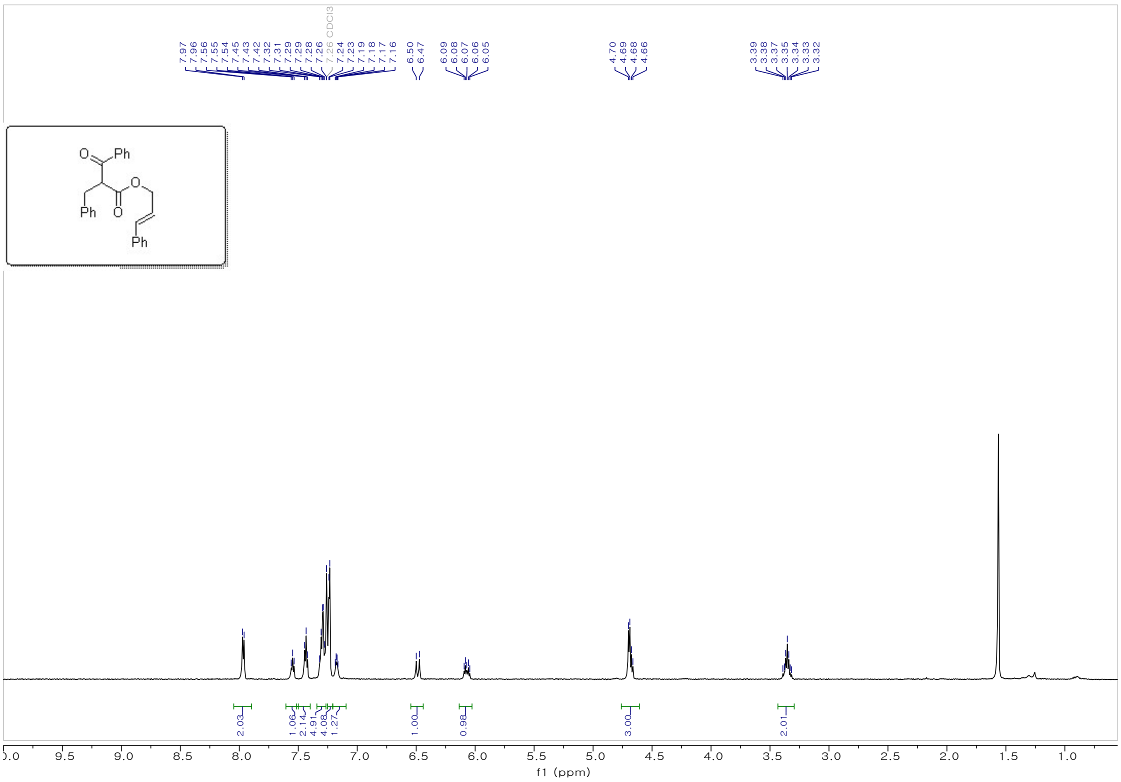

150 MHz, ^{13}C NMR in Chloroform-*d*

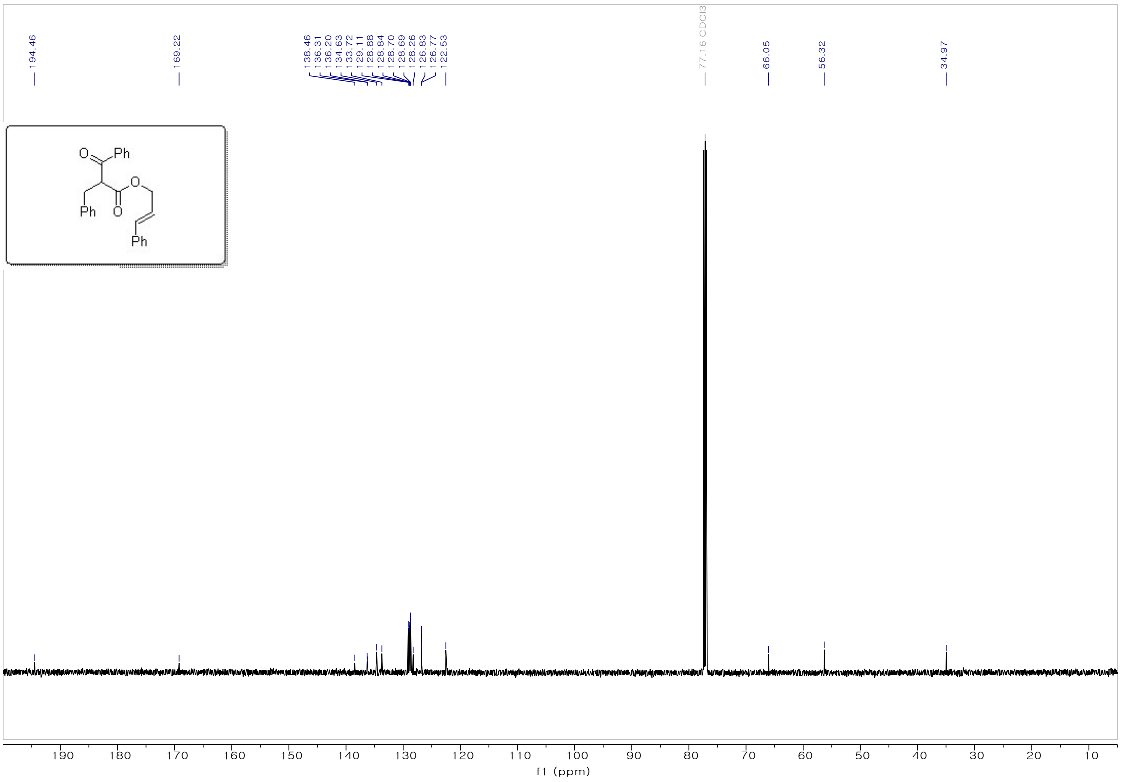

methyl 3-phenyl-2-((2,2,6,6-tetramethylpiperidin-1-yl)oxy)propanoate (9).

600 MHz, ^{1}H NMR in Chloroform-*d*

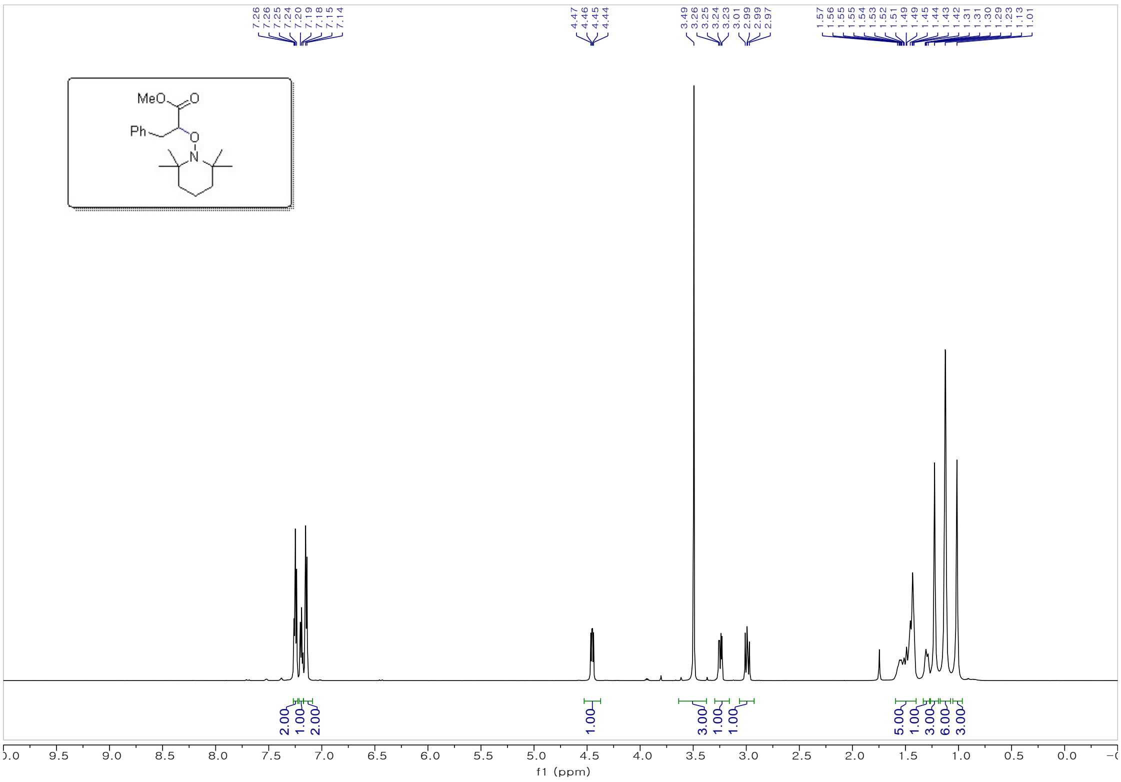

100 MHz, ^{13}C NMR in Chloroform-*d*

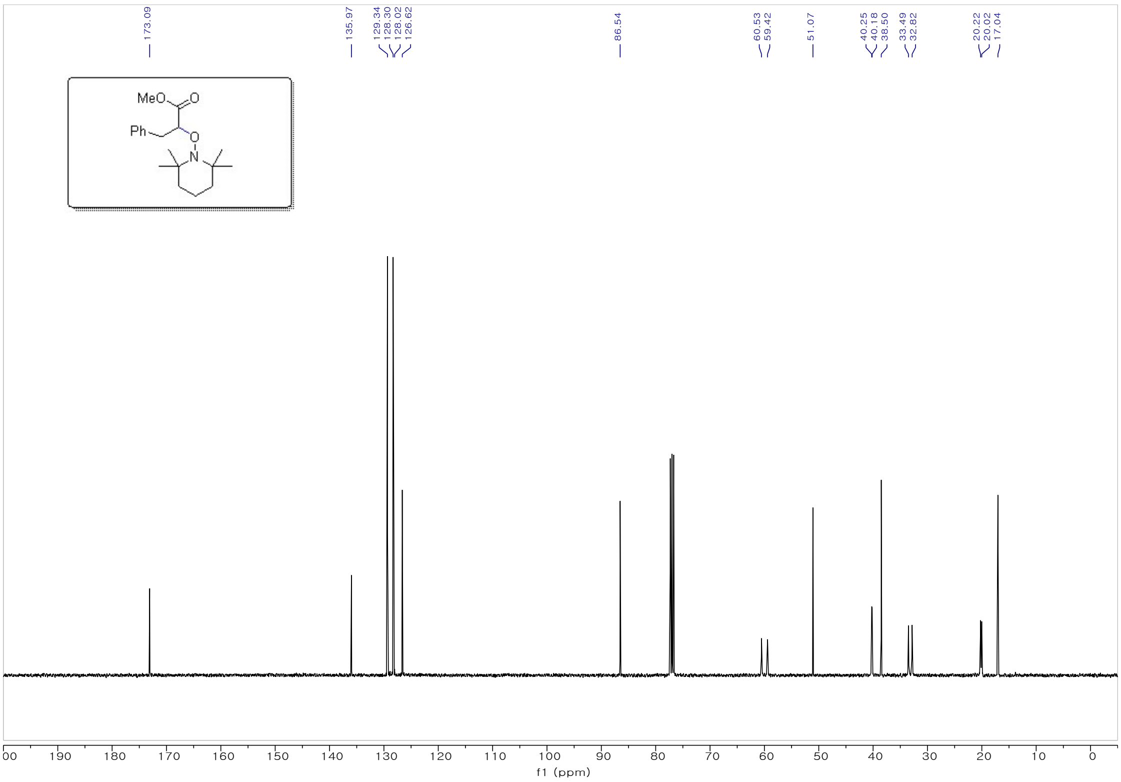

2,2,6,6-tetramethylpiperidin-1-yl benzoate (10).

600 MHz, ^{1}H NMR in Chloroform-*d*

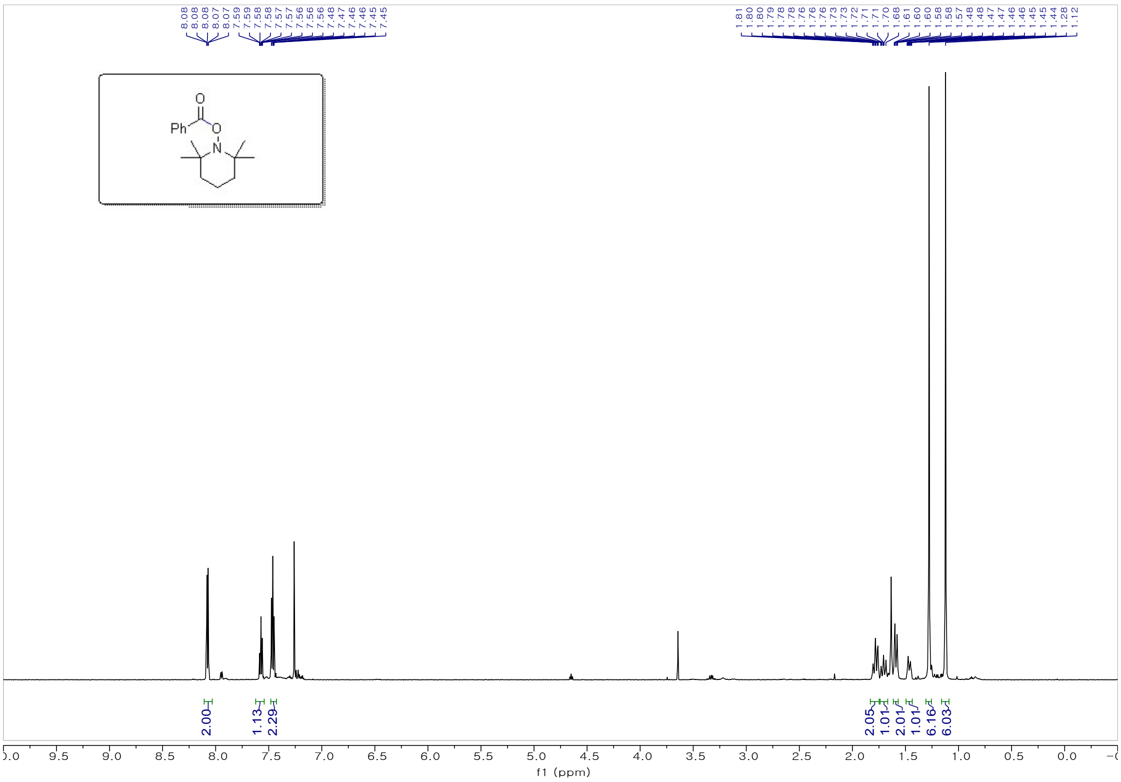

100 MHz, ^{13}C NMR in Chloroform-*d*

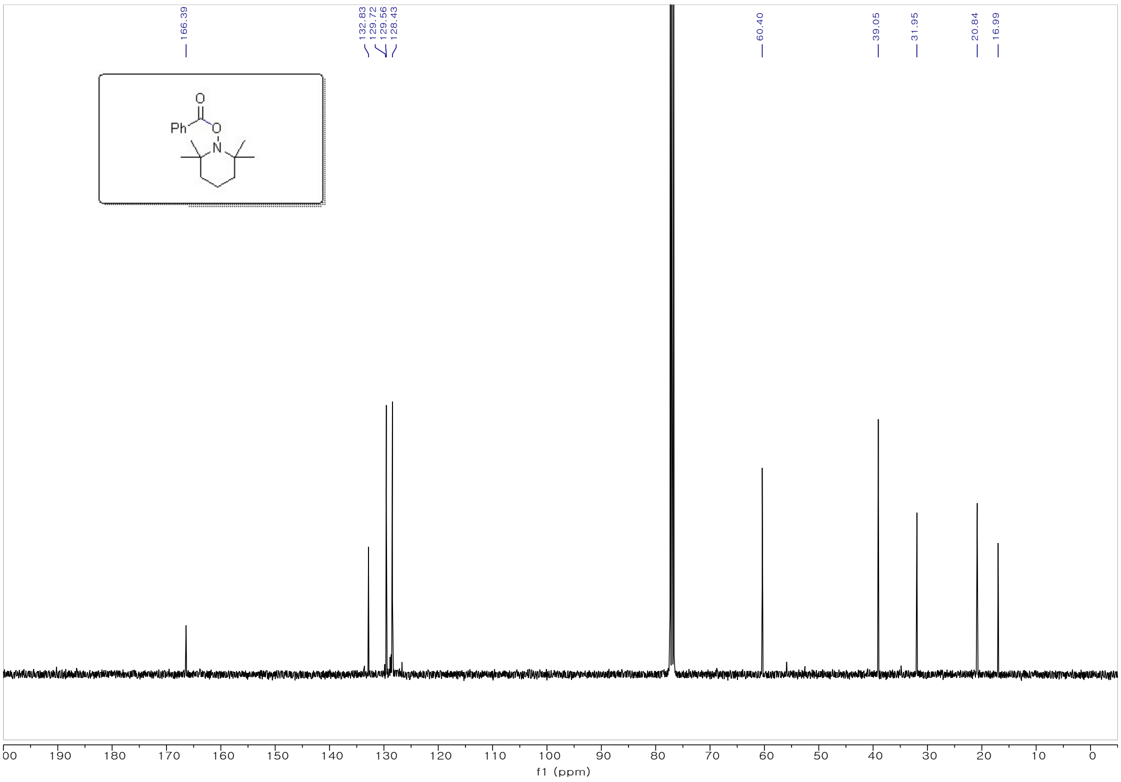

Appendix II

Crystallographic Data for 1a, 1g, 3m and 3y

Crystallographic Data for 1a (CCDC : 1964487)

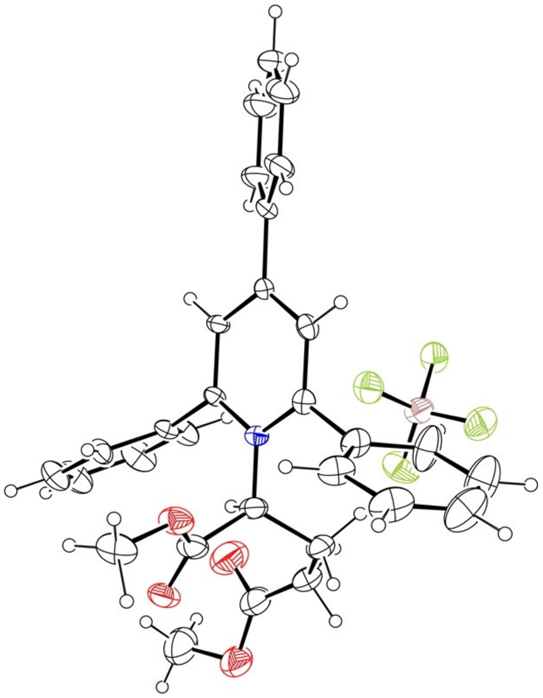

ORTEP representation (30% probability) of the crystal structure of **1a**

Table 1. Crystal data and structure refinement for **1a**.

Empirical formula	$C_{60}H_{56}N_2O_8B_2F_8$	
Formula weight	1106.68	
Temperature	153(2) K	
Wavelength	0.71073 Å	
Crystal system	Monoclinic	
Space group	*Pn*	
Unit cell dimensions	a = 12.5252(9) Å	α = 90°
	b = 16.8071(12) Å	β = 90.786(2)°
	c = 12.9207(11) Å	γ = 90°
Volume	2719.7(4) $Å^3$	
Z	2	
Density (calculated)	1.351 Mg/m^3	
Absorption coefficient	0.107 mm^{-1}	
F(000)	1152	
Crystal size	0.187 x 0.152 x 0.142 mm^3	
Theta range for data collection	2.892 to 29.192°.	
Index ranges	−17<=h<=15, −22<=k<=23, −17<=l<=17	
Reflections collected	64121	
Independent reflections	13233 [R(int) = 0.0958]	
Completeness to theta = 25.242°	99.4 %	
Absorption correction	Semi-empirical from equivalents	
Max. and min. transmission	0.7458 and 0.6797	
Refinement method	Full-matrix least-squares on F^2	
Data / restraints / parameters	13233 / 1756 / 976	
Goodness-of-fit on F^2	1.036	
Final R indices [I>2sigma(I)]	R1 = 0.0998, wR2 = 0.2440	
R indices (all data)	R1 = 0.1604, wR2 = 0.2992	
Absolute structure parameter	0.0(5)	
Largest diff. peak and hole	0.563 and −0.432 e·$Å^{-3}$	

Table 2. Atomic coordinates (x 10^4) and equivalent isotropic displacement parameters ($Å^2$ x 10^3) for **1a**. U(eq) is defined as one third of the trace of the orthogonalized U^{ij} tensor.

	x	y	z	U(eq)
N(1A)	7869(2)	-303(2)	3860(3)	29(1)
C(2A)	8404(3)	-181(2)	4796(3)	28(1)
C(3A)	9347(3)	-588(3)	5013(3)	31(1)
C(4A)	9755(3)	-1118(3)	4293(3)	31(1)
C(5A)	9220(3)	-1241(2)	3357(3)	32(1)
C(6A)	8277(3)	-833(3)	3140(2)	32(1)
C(7A)	10778(3)	-1557(3)	4547(4)	40(2)

C(8A)	11570(5)	-1171(3)	5120(5)	68(2)
C(9A)	12523(4)	-1559(4)	5353(5)	82(3)
C(10A)	12684(4)	-2334(4)	5012(6)	78(3)
C(11A)	11893(5)	-2720(3)	4439(6)	72(2)
C(12A)	10940(4)	-2331(3)	4206(5)	55(2)
C(13A)	7990(4)	359(3)	5580(3)	32(1)
C(14A)	7192(4)	111(2)	6244(4)	40(2)
C(15A)	6874(5)	602(3)	7050(4)	57(2)
C(16A)	7355(5)	1340(3)	7191(5)	68(2)
C(17A)	8154(5)	1588(3)	6526(5)	65(2)
C(18A)	8471(4)	1097(3)	5721(4)	48(2)
C(19A)	7772(5)	-941(4)	2114(3)	45(2)
C(20A)	6940(5)	-1476(4)	1954(4)	54(2)
C(21A)	6560(5)	-1623(4)	957(5)	72(3)
C(22A)	7011(7)	-1235(5)	119(4)	91(3)
C(23A)	7843(7)	-700(5)	279(4)	97(3)
C(24A)	8223(5)	-553(4)	1277(4)	75(3)
C(25A)	6990(30)	170(40)	3680(50)	43(3)
C(26A)	6980(50)	740(30)	2750(40)	50(4)
C(27A)	6870(70)	1580(30)	3140(40)	60(4)
C(28A)	6550(40)	2090(20)	4000(30)	75(4)
O(29A)	7200(50)	2210(40)	4750(40)	105(9)
O(30A)	5700(40)	2540(30)	4100(40)	84(4)
C(31A)	5810(80)	2790(50)	5170(40)	107(9)
C(32A)	5950(30)	-130(20)	4140(40)	51(3)
O(33A)	5330(30)	380(30)	4490(40)	63(9)
O(34A)	5860(50)	-890(30)	4120(50)	57(4)
C(35A)	4940(50)	-1340(30)	4510(70)	65(8)
C(25C)	6901(6)	208(6)	3635(9)	34(2)
C(26C)	7209(8)	940(6)	3001(10)	54(2)
C(27C)	6362(10)	1559(7)	2848(9)	60(2)
C(28C)	5832(9)	1852(6)	3799(8)	64(2)
O(29C)	6130(11)	1725(7)	4660(8)	115(4)
O(30C)	5085(7)	2357(5)	3594(6)	75(2)
C(31C)	4524(15)	2729(10)	4405(11)	107(5)
C(32C)	5941(7)	-273(5)	3320(8)	52(2)
O(33C)	5343(6)	-76(6)	2640(8)	83(3)
O(34C)	5810(9)	-891(6)	3932(9)	62(2)
C(35C)	4837(11)	-1371(8)	3770(15)	93(4)
B(36A)	10065(7)	1388(5)	2490(7)	51(2)
F(1E)	9800(16)	723(9)	3077(13)	63(4)
F(2E)	9975(11)	1187(10)	1416(8)	76(4)
F(3E)	9420(12)	2023(7)	2670(17)	79(4)
F(4E)	11155(11)	1540(20)	2680(20)	56(5)
F(5E)	9720(20)	673(11)	2636(19)	60(5)
F(6E)	9570(19)	1824(14)	1813(18)	92(5)
F(7E)	9782(13)	1827(10)	3517(14)	64(4)
F(8E)	11140(17)	1450(30)	2460(30)	61(6)
F(9E)	9780(30)	1330(30)	3580(30)	70(6)
F(10E)	9810(30)	720(20)	1990(40)	64(6)
F(11E)	9240(40)	1980(30)	2160(40)	67(6)
F(12E)	11000(40)	1650(40)	2380(50)	65(6)
N(1B)	1878(2)	5299(2)	6202(2)	25(1)
C(2B)	1523(3)	5810(2)	6970(2)	26(1)
C(3B)	603(3)	6258(2)	6808(3)	30(1)
C(4B)	39(3)	6197(2)	5878(3)	28(1)
C(5B)	395(3)	5687(2)	5109(3)	28(1)
C(6B)	1314(3)	5238(2)	5271(2)	25(1)
C(7B)	-917(3)	6717(3)	5699(4)	34(1)
C(8B)	-1031(4)	7408(3)	6279(4)	47(2)
C(9B)	-1929(4)	7884(2)	6140(4)	54(2)
C(10B)	-2712(4)	7671(3)	5420(5)	58(2)
C(11B)	-2599(4)	6980(3)	4840(4)	56(2)
C(12B)	-1701(4)	6503(3)	4979(4)	49(2)
C(13B)	2087(4)	5861(3)	7975(3)	33(1)
C(14B)	1795(4)	5371(3)	8790(4)	48(2)
C(15B)	2268(5)	5469(3)	9761(3)	63(2)
C(16B)	3033(5)	6058(4)	9917(3)	60(2)
C(17B)	3326(4)	6548(3)	9102(4)	55(2)
C(18B)	2853(4)	6450(3)	8131(3)	39(2)
C(19B)	1704(3)	4726(3)	4446(3)	26(1)
C(20B)	1159(4)	4022(3)	4241(4)	45(2)
C(21B)	1424(5)	3566(3)	3384(4)	59(2)
C(22B)	2234(5)	3814(3)	2734(4)	58(2)
C(23B)	2779(4)	4518(3)	2940(3)	46(2)
C(24B)	2514(3)	4974(2)	3796(3)	33(1)
C(25B)	2837(15)	4787(11)	6389(17)	28(2)
C(26B)	2666(8)	3910(5)	6313(8)	34(2)
C(27B)	3189(8)	3420(7)	7174(9)	41(2)
C(28B)	4341(8)	3604(6)	7313(8)	46(2)
O(29B)	4706(7)	4180(5)	7802(6)	54(2)
O(30B)	4934(11)	3072(8)	6777(9)	67(3)
C(31B)	6089(15)	3273(15)	6685(16)	87(6)
C(32B)	3842(7)	5105(6)	5888(8)	35(2)
O(33B)	4502(6)	4675(5)	5515(6)	45(2)
O(34B)	3885(17)	5900(11)	5866(11)	38(3)

C(35B)	4908(15)	6230(11)	5528(14)	58(4)
C(25D)	2830(40)	4810(30)	6430(40)	30(3)
C(26D)	2620(20)	4087(13)	7058(19)	37(3)
C(27D)	3060(20)	3328(14)	6560(20)	49(3)
C(28D)	4250(20)	3450(13)	6470(20)	64(3)
O(29D)	4580(20)	3775(15)	5653(19)	77(5)
O(30D)	5020(30)	3210(20)	7130(20)	69(4)
C(31D)	6130(30)	3500(30)	7070(40)	80(8)
C(32D)	3836(17)	5254(14)	6665(19)	32(3)
O(33D)	4485(15)	5036(11)	7279(15)	47(4)
O(34D)	3930(40)	5840(30)	6090(30)	36(4)
C(35D)	4900(40)	6350(30)	5980(30)	49(7)
B(36B)	-202(8)	3563(6)	7439(8)	46(2)
F(1F)	-63(19)	4364(10)	7086(16)	52(4)
F(2F)	0(20)	3067(12)	6592(18)	61(4)
F(3F)	690(13)	3382(15)	8094(12)	72(4)
F(4F)	-1166(16)	3438(18)	7779(19)	53(5)
F(5F)	60(20)	4339(14)	7440(30)	59(5)
F(6F)	-130(20)	3292(17)	6393(16)	53(5)
F(7F)	390(20)	3044(16)	7965(16)	66(5)
F(8F)	-1340(20)	3490(20)	7520(20)	49(5)
F(9F)	0(60)	4170(40)	6760(50)	55(6)
F(10F)	270(40)	2990(30)	7090(50)	56(6)
F(11F)	-40(40)	3680(30)	8690(40)	57(5)
F(12F)	-1000(60)	3340(50)	8060(50)	50(5)

Table 3. Bond lengths [Å] and angles [°] for **1a**.

N(1A)-C(25A)	1.38(5)
N(1A)-C(2A)	1.3900
N(1A)-C(6A)	1.3900
N(1A)-C(25C)	1.511(9)
C(2A)-C(3A)	1.3900
C(2A)-C(13A)	1.460(4)
C(3A)-C(4A)	1.3900
C(3A)-H(3A)	0.9500
C(4A)-C(5A)	1.3900
C(4A)-C(7A)	1.511(4)
C(5A)-C(6A)	1.3900
C(5A)-H(5A)	0.9500
C(6A)-C(19A)	1.473(5)
C(7A)-C(8A)	1.3900
C(7A)-C(12A)	1.3900
C(8A)-C(9A)	1.3900
C(8A)-H(8A)	0.9500
C(9A)-C(10A)	1.3900
C(9A)-H(9A)	0.9500
C(10A)-C(11A)	1.3900
C(10A)-H(10A)	0.9500
C(11A)-C(12A)	1.3900
C(11A)-H(11A)	0.9500
C(12A)-H(12A)	0.9500
C(13A)-C(14A)	1.3900
C(13A)-C(18A)	1.3900
C(14A)-C(15A)	1.3900
C(14A)-H(14A)	0.9500
C(15A)-C(16A)	1.3900
C(15A)-H(15A)	0.9500
C(16A)-C(17A)	1.3900
C(16A)-H(16A)	0.9500
C(17A)-C(18A)	1.3900
C(17A)-H(17A)	0.9500
C(18A)-H(18A)	0.9500
C(19A)-C(20A)	1.3900
C(19A)-C(24A)	1.3900
C(20A)-C(21A)	1.3900
C(20A)-H(20A)	0.9500
C(21A)-C(22A)	1.3900
C(21A)-H(21A)	0.9500
C(22A)-C(23A)	1.3900
C(22A)-H(22A)	0.9500
C(23A)-C(24A)	1.3900
C(23A)-H(23A)	0.9500
C(24A)-H(24A)	0.9500
C(25A)-C(32A)	1.520(12)
C(25A)-C(26A)	1.529(13)
C(25A)-H(25A)	1.0000
C(26A)-C(27A)	1.50(3)
C(26A)-H(26A)	0.9900
C(26A)-H(26B)	0.9900
C(27A)-C(28A)	1.48(3)
C(27A)-H(27A)	0.9900
C(27A)-H(27B)	0.9900
C(28A)-O(29A)	1.27(3)
C(28A)-O(30A)	1.30(3)
O(30A)-C(31A)	1.45(3)
C(31A)-H(31A)	0.9800
C(31A)-H(31B)	0.9800
C(31A)-H(31C)	0.9800
C(32A)-O(33A)	1.24(2)
C(32A)-O(34A)	1.29(3)
O(34A)-C(35A)	1.46(3)
C(35A)-H(35A)	0.9800
C(35A)-H(35B)	0.9800
C(35A)-H(35C)	0.9800
C(25C)-C(32C)	1.501(9)
C(25C)-C(26C)	1.531(9)
C(25C)-H(25C)	1.0000
C(26C)-C(27C)	1.497(14)
C(26C)-H(26C)	0.9900
C(26C)-H(26D)	0.9900
C(27C)-C(28C)	1.490(14)
C(27C)-H(27C)	0.9900
C(27C)-H(27D)	0.9900
C(28C)-O(29C)	1.188(12)
C(28C)-O(30C)	1.287(11)
O(30C)-C(31C)	1.414(13)
C(31C)-H(31D)	0.9800
C(31C)-H(31E)	0.9800
C(31C)-H(31F)	0.9800
C(32C)-O(33C)	1.193(11)
C(32C)-O(34C)	1.318(12)
O(34C)-C(35C)	1.474(12)
C(35C)-H(35L)	0.9800
C(35C)-H(35M)	0.9800
C(35C)-H(35N)	0.9800
B(36A)-F(12E)	1.26(4)
B(36A)-F(6E)	1.292(19)
B(36A)-F(5E)	1.29(2)
B(36A)-F(10E)	1.33(4)
B(36A)-F(8E)	1.35(2)
B(36A)-F(3E)	1.360(11)
B(36A)-F(1E)	1.394(11)
B(36A)-F(4E)	1.406(12)
B(36A)-F(2E)	1.431(11)
B(36A)-F(9E)	1.45(4)
B(36A)-F(11E)	1.49(4)
B(36A)-F(7E)	1.564(17)
N(1B)-C(2B)	1.3900
N(1B)-C(6B)	1.3900
N(1B)-C(25D)	1.47(5)
N(1B)-C(25B)	1.49(2)
C(2B)-C(3B)	1.3900
C(2B)-C(13B)	1.472(4)
C(3B)-C(4B)	1.3900
C(3B)-H(3B)	0.9500
C(4B)-C(5B)	1.3900
C(4B)-C(7B)	1.499(4)
C(5B)-C(6B)	1.3900
C(5B)-H(5B)	0.9500
C(6B)-C(19B)	1.459(4)
C(7B)-C(8B)	1.3900
C(7B)-C(12B)	1.3900
C(8B)-C(9B)	1.3900
C(8B)-H(8B)	0.9500
C(9B)-C(10B)	1.3900

C(9B)-H(9B)	0.9500
C(10B)-C(11B)	1.3900
C(10B)-H(10B)	0.9500
C(11B)-C(12B)	1.3900
C(11B)-H(11B)	0.9500
C(12B)-H(12B)	0.9500
C(13B)-C(14B)	1.3900
C(13B)-C(18B)	1.3900
C(14B)-C(15B)	1.3900
C(14B)-H(14B)	0.9500
C(15B)-C(16B)	1.3900
C(15B)-H(15B)	0.9500
C(16B)-C(17B)	1.3900
C(16B)-H(16B)	0.9500
C(17B)-C(18B)	1.3900
C(17B)-H(17B)	0.9500
C(18B)-H(18B)	0.9500
C(19B)-C(20B)	1.3900
C(19B)-C(24B)	1.3900
C(20B)-C(21B)	1.3900
C(20B)-H(20B)	0.9500
C(21B)-C(22B)	1.3900
C(21B)-H(21B)	0.9500
C(22B)-C(23B)	1.3900
C(22B)-H(22B)	0.9500
C(23B)-C(24B)	1.3900
C(23B)-H(23B)	0.9500
C(24B)-H(24B)	0.9500
C(25B)-C(26B)	1.493(18)
C(25B)-C(32B)	1.520(18)
C(25B)-H(25B)	1.0000
C(26B)-C(27B)	1.524(12)
C(26B)-H(26E)	0.9900
C(26B)-H(26F)	0.9900
C(27B)-C(28B)	1.484(14)
C(27B)-H(27E)	0.9900
C(27B)-H(27F)	0.9900
C(28B)-O(29B)	1.240(13)
C(28B)-O(30B)	1.359(14)
O(30B)-C(31B)	1.491(17)
C(31B)-H(31G)	0.9800
C(31B)-H(31H)	0.9800
C(31B)-H(31I)	0.9800
C(32B)-O(33B)	1.204(12)
C(32B)-O(34B)	1.34(2)
O(34B)-C(35B)	1.47(2)
C(35B)-H(35D)	0.9800
C(35B)-H(35E)	0.9800
C(35B)-H(35F)	0.9800
C(25D)-C(26D)	1.49(3)
C(25D)-C(32D)	1.49(3)
C(25D)-H(25D)	1.0000
C(26D)-C(27D)	1.53(2)
C(26D)-H(26G)	0.9900
C(26D)-H(26H)	0.9900
C(27D)-C(28D)	1.52(2)
C(27D)-H(27G)	0.9900
C(27D)-H(27H)	0.9900
C(28D)-O(29D)	1.26(2)
C(28D)-O(30D)	1.34(2)
O(30D)-C(31D)	1.47(3)
C(31D)-H(31J)	0.9800
C(31D)-H(31K)	0.9800
C(31D)-H(31L)	0.9800
C(32D)-O(33D)	1.19(3)
C(32D)-O(34D)	1.23(5)
O(34D)-C(35D)	1.49(6)
C(35D)-H(35G)	0.9800
C(35D)-H(35H)	0.9800
C(35D)-H(35I)	0.9800
B(36B)-F(10F)	1.21(6)
B(36B)-F(4F)	1.31(2)
B(36B)-F(7F)	1.328(19)
B(36B)-F(5F)	1.34(2)
B(36B)-F(12F)	1.35(8)
B(36B)-F(9F)	1.37(6)
B(36B)-F(2F)	1.40(2)
B(36B)-F(3F)	1.424(16)
B(36B)-F(6F)	1.43(2)
B(36B)-F(1F)	1.434(19)
B(36B)-F(8F)	1.44(3)
B(36B)-F(11F)	1.63(5)
F(11F)-F(12F)	1.54(8)

C(25A)-N(1A)-C(2A)	116(3)
C(25A)-N(1A)-C(6A)	124(3)
C(2A)-N(1A)-C(6A)	120.0
C(2A)-N(1A)-C(25C)	117.3(5)
C(6A)-N(1A)-C(25C)	122.5(5)
C(3A)-C(2A)-N(1A)	120.0
C(3A)-C(2A)-C(13A)	118.4(3)
N(1A)-C(2A)-C(13A)	121.6(3)
C(4A)-C(3A)-C(2A)	120.0
C(4A)-C(3A)-H(3A)	120.0
C(2A)-C(3A)-H(3A)	120.0
C(5A)-C(4A)-C(3A)	120.0
C(5A)-C(4A)-C(7A)	120.9(3)
C(3A)-C(4A)-C(7A)	119.1(3)
C(4A)-C(5A)-C(6A)	120.0
C(4A)-C(5A)-H(5A)	120.0
C(6A)-C(5A)-H(5A)	120.0
C(5A)-C(6A)-N(1A)	120.0
C(5A)-C(6A)-C(19A)	118.3(4)
N(1A)-C(6A)-C(19A)	121.6(4)
C(8A)-C(7A)-C(12A)	120.0
C(8A)-C(7A)-C(4A)	119.0(4)
C(12A)-C(7A)-C(4A)	121.0(4)
C(9A)-C(8A)-C(7A)	120.0
C(9A)-C(8A)-H(8A)	120.0
C(7A)-C(8A)-H(8A)	120.0
C(8A)-C(9A)-C(10A)	120.0
C(8A)-C(9A)-H(9A)	120.0
C(10A)-C(9A)-H(9A)	120.0
C(9A)-C(10A)-C(11A)	120.0
C(9A)-C(10A)-H(10A)	120.0
C(11A)-C(10A)-H(10A)	120.0
C(12A)-C(11A)-C(10A)	120.0
C(12A)-C(11A)-H(11A)	120.0
C(10A)-C(11A)-H(11A)	120.0
C(11A)-C(12A)-C(7A)	120.0
C(11A)-C(12A)-H(12A)	120.0
C(7A)-C(12A)-H(12A)	120.0
C(14A)-C(13A)-C(18A)	120.0
C(14A)-C(13A)-C(2A)	120.6(3)
C(18A)-C(13A)-C(2A)	119.2(3)
C(15A)-C(14A)-C(13A)	120.0
C(15A)-C(14A)-H(14A)	120.0
C(13A)-C(14A)-H(14A)	120.0
C(14A)-C(15A)-C(16A)	120.0
C(14A)-C(15A)-H(15A)	120.0
C(16A)-C(15A)-H(15A)	120.0
C(15A)-C(16A)-C(17A)	120.0
C(15A)-C(16A)-H(16A)	120.0
C(17A)-C(16A)-H(16A)	120.0
C(18A)-C(17A)-C(16A)	120.0
C(18A)-C(17A)-H(17A)	120.0
C(16A)-C(17A)-H(17A)	120.0
C(17A)-C(18A)-C(13A)	120.0
C(17A)-C(18A)-H(18A)	120.0
C(13A)-C(18A)-H(18A)	120.0
C(20A)-C(19A)-C(24A)	120.0
C(20A)-C(19A)-C(6A)	121.7(4)
C(24A)-C(19A)-C(6A)	118.0(4)
C(21A)-C(20A)-C(19A)	120.0
C(21A)-C(20A)-H(20A)	120.0
C(19A)-C(20A)-H(20A)	120.0
C(20A)-C(21A)-C(22A)	120.0
C(20A)-C(21A)-H(21A)	120.0
C(22A)-C(21A)-H(21A)	120.0
C(23A)-C(22A)-C(21A)	120.0
C(23A)-C(22A)-H(22A)	120.0
C(21A)-C(22A)-H(22A)	120.0
C(22A)-C(23A)-C(24A)	120.0
C(22A)-C(23A)-H(23A)	120.0
C(24A)-C(23A)-H(23A)	120.0
C(23A)-C(24A)-C(19A)	120.0
C(23A)-C(24A)-H(24A)	120.0
C(19A)-C(24A)-H(24A)	120.0
N(1A)-C(25A)-C(32A)	115(3)
N(1A)-C(25A)-C(26A)	119(3)
C(32A)-C(25A)-C(26A)	121.1(16)
N(1A)-C(25A)-H(25A)	97.0
C(32A)-C(25A)-H(25A)	97.0
C(26A)-C(25A)-H(25A)	97.0
C(27A)-C(26A)-C(25A)	109(5)
C(27A)-C(26A)-H(26A)	109.9
C(25A)-C(26A)-H(26A)	109.9
C(27A)-C(26A)-H(26B)	109.9
C(25A)-C(26A)-H(26B)	109.9
H(26A)-C(26A)-H(26B)	108.3
C(28A)-C(27A)-C(26A)	146(4)
C(28A)-C(27A)-H(27A)	100.4

C(26A)-C(27A)-H(27A)	100.4
C(28A)-C(27A)-H(27B)	100.4
C(26A)-C(27A)-H(27B)	100.4
H(27A)-C(27A)-H(27B)	104.3
O(29A)-C(28A)-O(30A)	111(4)
O(29A)-C(28A)-C(27A)	119(5)
O(30A)-C(28A)-C(27A)	130(5)
C(28A)-O(30A)-C(31A)	101(3)
O(30A)-C(31A)-H(31A)	109.5
O(30A)-C(31A)-H(31B)	109.5
H(31A)-C(31A)-H(31B)	109.5
O(30A)-C(31A)-H(31C)	109.5
H(31A)-C(31A)-H(31C)	109.5
H(31B)-C(31A)-H(31C)	109.5
O(33A)-C(32A)-O(34A)	129(3)
O(33A)-C(32A)-C(25A)	117(2)
O(34A)-C(32A)-C(25A)	114(2)
C(32A)-O(34A)-C(35A)	125(3)
O(34A)-C(35A)-H(35A)	109.5
O(34A)-C(35A)-H(35B)	109.5
H(35A)-C(35A)-H(35B)	109.5
O(34A)-C(35A)-H(35C)	109.5
H(35A)-C(35A)-H(35C)	109.5
H(35B)-C(35A)-H(35C)	109.5
C(32C)-C(25C)-N(1A)	112.6(7)
C(32C)-C(25C)-C(26C)	119.7(7)
N(1A)-C(25C)-C(26C)	110.7(7)
C(32C)-C(25C)-H(25C)	104.0
N(1A)-C(25C)-H(25C)	104.0
C(26C)-C(25C)-H(25C)	104.0
C(27C)-C(26C)-C(25C)	116.5(9)
C(27C)-C(26C)-H(26C)	108.2
C(25C)-C(26C)-H(26C)	108.2
C(27C)-C(26C)-H(26D)	108.2
C(25C)-C(26C)-H(26D)	108.2
H(26C)-C(26C)-H(26D)	107.3
C(28C)-C(27C)-C(26C)	116.4(10)
C(28C)-C(27C)-H(27C)	108.2
C(26C)-C(27C)-H(27C)	108.2
C(28C)-C(27C)-H(27D)	108.2
C(26C)-C(27C)-H(27D)	108.2
H(27C)-C(27C)-H(27D)	107.4
O(29C)-C(28C)-O(30C)	122.0(10)
O(29C)-C(28C)-C(27C)	125.1(10)
O(30C)-C(28C)-C(27C)	112.3(9)
C(28C)-O(30C)-C(31C)	120.4(9)
O(30C)-C(31C)-H(31D)	109.5
O(30C)-C(31C)-H(31E)	109.5
H(31D)-C(31C)-H(31E)	109.5
O(30C)-C(31C)-H(31F)	109.5
H(31D)-C(31C)-H(31F)	109.5
H(31E)-C(31C)-H(31F)	109.5
O(33C)-C(32C)-O(34C)	125.4(9)
O(33C)-C(32C)-C(25C)	122.9(8)
O(34C)-C(32C)-C(25C)	111.5(8)
C(32C)-O(34C)-C(35C)	117.1(11)
O(34C)-C(35C)-H(35L)	109.5
O(34C)-C(35C)-H(35M)	109.5
H(35L)-C(35C)-H(35M)	109.5
O(34C)-C(35C)-H(35N)	109.5
H(35L)-C(35C)-H(35N)	109.5
H(35M)-C(35C)-H(35N)	109.5
F(6E)-B(36A)-F(5E)	117.9(17)
F(12E)-B(36A)-F(10E)	117(3)
F(6E)-B(36A)-F(8E)	114(2)
F(5E)-B(36A)-F(8E)	114(2)
F(3E)-B(36A)-F(1E)	112.9(13)
F(3E)-B(36A)-F(4E)	114.0(15)
F(1E)-B(36A)-F(4E)	106.6(15)
F(3E)-B(36A)-F(2E)	108.2(12)
F(1E)-B(36A)-F(2E)	108.8(11)
F(4E)-B(36A)-F(2E)	106.0(13)
F(12E)-B(36A)-F(9E)	112(3)
F(10E)-B(36A)-F(9E)	111(3)
F(12E)-B(36A)-F(11E)	112(3)
F(10E)-B(36A)-F(11E)	105(3)
F(9E)-B(36A)-F(11E)	98(2)
F(6E)-B(36A)-F(7E)	101.2(15)
F(5E)-B(36A)-F(7E)	103.5(13)
F(8E)-B(36A)-F(7E)	102.7(18)
C(2B)-N(1B)-C(6B)	120.0
C(2B)-N(1B)-C(25D)	118(2)
C(6B)-N(1B)-C(25D)	122(2)
C(2B)-N(1B)-C(25B)	120.3(9)
C(6B)-N(1B)-C(25B)	119.6(9)
C(3B)-C(2B)-N(1B)	120.0
C(3B)-C(2B)-C(13B)	119.2(3)
N(1B)-C(2B)-C(13B)	120.8(3)
C(2B)-C(3B)-C(4B)	120.0
C(2B)-C(3B)-H(3B)	120.0
C(4B)-C(3B)-H(3B)	120.0
C(3B)-C(4B)-C(5B)	120.0
C(3B)-C(4B)-C(7B)	119.1(3)
C(5B)-C(4B)-C(7B)	120.9(3)
C(4B)-C(5B)-C(6B)	120.0
C(4B)-C(5B)-H(5B)	120.0
C(6B)-C(5B)-H(5B)	120.0
C(5B)-C(6B)-N(1B)	120.0
C(5B)-C(6B)-C(19B)	119.6(3)
N(1B)-C(6B)-C(19B)	120.3(3)
C(8B)-C(7B)-C(12B)	120.0
C(8B)-C(7B)-C(4B)	119.4(3)
C(12B)-C(7B)-C(4B)	120.5(3)
C(7B)-C(8B)-C(9B)	120.0
C(7B)-C(8B)-H(8B)	120.0
C(9B)-C(8B)-H(8B)	120.0
C(10B)-C(9B)-C(8B)	120.0
C(10B)-C(9B)-H(9B)	120.0
C(8B)-C(9B)-H(9B)	120.0
C(11B)-C(10B)-C(9B)	120.0
C(11B)-C(10B)-H(10B)	120.0
C(9B)-C(10B)-H(10B)	120.0
C(10B)-C(11B)-C(12B)	120.0
C(10B)-C(11B)-H(11B)	120.0
C(12B)-C(11B)-H(11B)	120.0
C(11B)-C(12B)-C(7B)	120.0
C(11B)-C(12B)-H(12B)	120.0
C(7B)-C(12B)-H(12B)	120.0
C(14B)-C(13B)-C(18B)	120.0
C(14B)-C(13B)-C(2B)	120.3(3)
C(18B)-C(13B)-C(2B)	119.5(3)
C(15B)-C(14B)-C(13B)	120.0
C(15B)-C(14B)-H(14B)	120.0
C(13B)-C(14B)-H(14B)	120.0
C(14B)-C(15B)-C(16B)	120.0
C(14B)-C(15B)-H(15B)	120.0
C(16B)-C(15B)-H(15B)	120.0
C(15B)-C(16B)-C(17B)	120.0
C(15B)-C(16B)-H(16B)	120.0
C(17B)-C(16B)-H(16B)	120.0
C(18B)-C(17B)-C(16B)	120.0
C(18B)-C(17B)-H(17B)	120.0
C(16B)-C(17B)-H(17B)	120.0
C(17B)-C(18B)-C(13B)	120.0
C(17B)-C(18B)-H(18B)	120.0
C(13B)-C(18B)-H(18B)	120.0
C(20B)-C(19B)-C(24B)	120.0
C(20B)-C(19B)-C(6B)	118.2(3)
C(24B)-C(19B)-C(6B)	121.3(3)
C(21B)-C(20B)-C(19B)	120.0
C(21B)-C(20B)-H(20B)	120.0
C(19B)-C(20B)-H(20B)	120.0
C(20B)-C(21B)-C(22B)	120.0
C(20B)-C(21B)-H(21B)	120.0
C(22B)-C(21B)-H(21B)	120.0
C(23B)-C(22B)-C(21B)	120.0
C(23B)-C(22B)-H(22B)	120.0
C(21B)-C(22B)-H(22B)	120.0
C(22B)-C(23B)-C(24B)	120.0
C(22B)-C(23B)-H(23B)	120.0
C(24B)-C(23B)-H(23B)	120.0
C(23B)-C(24B)-C(19B)	120.0
C(23B)-C(24B)-H(24B)	120.0
C(19B)-C(24B)-H(24B)	120.0
C(26B)-C(25B)-N(1B)	116.4(11)
C(26B)-C(25B)-C(32B)	116.0(17)
N(1B)-C(25B)-C(32B)	113.4(10)
C(26B)-C(25B)-H(25B)	102.8
N(1B)-C(25B)-H(25B)	102.8
C(32B)-C(25B)-H(25B)	102.8
C(25B)-C(26B)-C(27B)	115.2(11)
C(25B)-C(26B)-H(26E)	108.5
C(27B)-C(26B)-H(26E)	108.5
C(25B)-C(26B)-H(26F)	108.5
C(27B)-C(26B)-H(26F)	108.5
H(26E)-C(26B)-H(26F)	107.5
C(28B)-C(27B)-C(26B)	112.5(8)
C(28B)-C(27B)-H(27E)	109.1
C(26B)-C(27B)-H(27E)	109.1
C(28B)-C(27B)-H(27F)	109.1
C(26B)-C(27B)-H(27F)	109.1
H(27E)-C(27B)-H(27F)	107.8

O(29B)-C(28B)-O(30B)	124.9(12)	O(30D)-C(28D)-C(27D)	127(3)
O(29B)-C(28B)-C(27B)	125.2(10)	C(28D)-O(30D)-C(31D)	122(3)
O(30B)-C(28B)-C(27B)	109.8(11)	O(30D)-C(31D)-H(31J)	109.5
C(28B)-O(30B)-C(31B)	115.4(14)	O(30D)-C(31D)-H(31K)	109.5
O(30B)-C(31B)-H(31G)	109.5	H(31J)-C(31D)-H(31K)	109.5
O(30B)-C(31B)-H(31H)	109.5	O(30D)-C(31D)-H(31L)	109.5
H(31G)-C(31B)-H(31H)	109.5	H(31J)-C(31D)-H(31L)	109.5
O(30B)-C(31B)-H(31I)	109.5	H(31K)-C(31D)-H(31L)	109.5
H(31G)-C(31B)-H(31I)	109.5	O(33D)-C(32D)-O(34D)	125(3)
H(31H)-C(31B)-H(31I)	109.5	O(33D)-C(32D)-C(25D)	123(3)
O(33B)-C(32B)-O(34B)	124.3(12)	O(34D)-C(32D)-C(25D)	111(3)
O(33B)-C(32B)-C(25B)	122.4(12)	C(32D)-O(34D)-C(35D)	127(4)
O(34B)-C(32B)-C(25B)	113.2(14)	O(34D)-C(35D)-H(35G)	109.5
C(32B)-O(34B)-C(35B)	114.8(17)	O(34D)-C(35D)-H(35H)	109.5
O(34B)-C(35B)-H(35D)	109.5	H(35G)-C(35D)-H(35H)	109.5
O(34B)-C(35B)-H(35E)	109.5	O(34D)-C(35D)-H(35I)	109.5
H(35D)-C(35B)-H(35E)	109.5	H(35G)-C(35D)-H(35I)	109.5
O(34B)-C(35B)-H(35F)	109.5	H(35H)-C(35D)-H(35I)	109.5
H(35D)-C(35B)-H(35F)	109.5	F(7F)-B(36B)-F(5F)	120.2(16)
H(35E)-C(35B)-H(35F)	109.5	F(10F)-B(36B)-F(12F)	112(5)
N(1B)-C(25D)-C(26D)	115(3)	F(10F)-B(36B)-F(9F)	105(4)
N(1B)-C(25D)-C(32D)	116(3)	F(12F)-B(36B)-F(9F)	137(5)
C(26D)-C(25D)-C(32D)	117(3)	F(4F)-B(36B)-F(2F)	110.1(16)
N(1B)-C(25D)-H(25D)	101.6	F(4F)-B(36B)-F(3F)	119.1(14)
C(26D)-C(25D)-H(25D)	101.6	F(2F)-B(36B)-F(3F)	101.0(13)
C(32D)-C(25D)-H(25D)	101.6	F(7F)-B(36B)-F(6F)	103.5(14)
C(25D)-C(26D)-C(27D)	113(3)	F(5F)-B(36B)-F(6F)	107.1(15)
C(25D)-C(26D)-H(26G)	109.0	F(4F)-B(36B)-F(1F)	112.0(16)
C(27D)-C(26D)-H(26G)	109.0	F(2F)-B(36B)-F(1F)	106.6(11)
C(25D)-C(26D)-H(26H)	109.0	F(3F)-B(36B)-F(1F)	107.0(12)
C(27D)-C(26D)-H(26H)	109.0	F(7F)-B(36B)-F(8F)	117.1(18)
H(26G)-C(26D)-H(26H)	107.8	F(5F)-B(36B)-F(8F)	108.7(19)
C(28D)-C(27D)-C(26D)	106.4(18)	F(6F)-B(36B)-F(8F)	97.0(16)
C(28D)-C(27D)-H(27G)	110.5	F(10F)-B(36B)-F(11F)	114(3)
C(26D)-C(27D)-H(27G)	110.5	F(12F)-B(36B)-F(11F)	61(3)
C(28D)-C(27D)-H(27H)	110.5	F(9F)-B(36B)-F(11F)	122(3)
C(26D)-C(27D)-H(27H)	110.5	F(12F)-F(11F)-B(36B)	50(3)
H(27G)-C(27D)-H(27H)	108.6	B(36B)-F(12F)-F(11F)	68(4)
O(29D)-C(28D)-O(30D)	115(3)		
O(29D)-C(28D)-C(27D)	117(2)		

Symmetry transformations used to generate equivalent atoms:

Table 4. Anisotropic displacement parameters (Å^2 x 10^3) for **1a**. The anisotropic displacement factor exponent takes the form: $-2\pi^2$[$h^2a^{*2}U^{11}$ + ... + 2 h k a* b* U^{12}]

	U^{11}	U^{22}	U^{33}	U^{23}	U^{13}	U^{12}
N(1A)	24(2)	26(2)	36(3)	1(2)	-2(2)	1(2)
C(2A)	20(3)	32(3)	31(3)	-3(2)	1(2)	1(2)
C(3A)	23(3)	37(3)	32(3)	-2(2)	-1(2)	8(2)
C(4A)	28(3)	36(3)	30(3)	8(2)	7(2)	4(2)
C(5A)	35(3)	28(3)	32(3)	-6(2)	7(2)	-4(2)
C(6A)	34(3)	31(3)	32(3)	-1(2)	1(2)	-6(2)
C(7A)	33(3)	48(4)	39(3)	10(3)	15(3)	17(3)
C(8A)	53(4)	106(6)	45(4)	-16(4)	-13(3)	39(4)
C(9A)	53(5)	134(7)	59(5)	-6(5)	-9(4)	44(5)
C(10A)	54(5)	98(6)	83(6)	39(5)	20(4)	40(5)
C(11A)	64(5)	52(4)	102(6)	34(4)	35(4)	28(4)
C(12A)	41(4)	42(4)	83(5)	20(4)	27(4)	13(3)
C(13A)	25(3)	31(3)	39(3)	-6(3)	2(2)	7(2)
C(14A)	39(4)	36(3)	46(4)	0(3)	15(3)	5(3)
C(15A)	51(5)	60(4)	61(5)	-13(4)	20(4)	7(4)
C(16A)	61(5)	67(5)	77(6)	-38(4)	16(4)	5(4)
C(17A)	57(5)	54(4)	83(6)	-35(4)	7(4)	-5(4)
C(18A)	37(4)	42(4)	66(5)	-14(3)	8(3)	-5(3)
C(19A)	48(4)	49(4)	39(3)	-11(3)	-11(3)	-7(3)
C(20A)	52(5)	45(4)	64(5)	-10(4)	-17(4)	-2(3)
C(21A)	71(6)	62(5)	82(6)	-21(4)	-36(5)	-10(4)
C(22A)	125(8)	85(7)	61(5)	-14(5)	-42(5)	-12(6)
C(23A)	147(9)	97(7)	46(5)	-13(5)	-22(5)	-34(6)
C(24A)	110(7)	83(6)	33(4)	-3(4)	-10(4)	-38(5)
C(25A)	31(6)	43(6)	54(6)	10(6)	-4(5)	4(5)
C(26A)	46(7)	50(6)	55(7)	18(6)	-2(6)	7(6)
C(27A)	63(7)	58(6)	58(7)	20(6)	-3(7)	19(7)
C(28A)	85(7)	79(7)	60(7)	14(6)	0(6)	31(7)
O(29A)	120(17)	120(19)	75(15)	-3(15)	-16(14)	41(17)
O(30A)	99(8)	87(8)	65(8)	10(8)	5(7)	39(7)
C(31A)	138(17)	115(17)	69(13)	5(14)	13(15)	47(16)
C(32A)	32(6)	52(6)	68(7)	10(6)	-3(6)	-1(6)
O(33A)	41(16)	68(16)	80(20)	19(17)	9(15)	16(15)
O(34A)	35(8)	56(7)	80(9)	8(8)	-5(8)	-7(7)
C(35A)	33(14)	65(14)	95(17)	-9(16)	-14(15)	-23(12)
C(25C)	22(3)	37(3)	43(4)	9(3)	-6(3)	3(3)
C(26C)	49(5)	50(4)	64(5)	21(4)	1(4)	7(4)
C(27C)	63(5)	57(5)	59(5)	18(4)	5(4)	15(4)
C(28C)	75(5)	66(5)	51(4)	9(4)	-11(4)	33(4)

O(29C)	160(9)	121(8)	64(5)	2(5)	-4(5)	69(7)
O(30C)	85(5)	88(5)	51(4)	18(4)	10(4)	45(4)
C(31C)	144(11)	108(10)	69(7)	15(7)	36(8)	59(9)
C(32C)	30(4)	54(4)	71(5)	4(4)	-10(4)	2(3)
O(33C)	46(4)	81(5)	121(7)	1(5)	-49(4)	13(4)
O(34C)	39(4)	60(4)	86(6)	10(4)	-1(4)	-17(3)
C(35C)	49(6)	97(9)	132(11)	-6(9)	-3(7)	-34(6)
B(36A)	43(4)	50(4)	60(5)	10(4)	-5(4)	-7(3)
F(1E)	58(7)	55(6)	76(9)	15(7)	11(8)	-12(5)
F(2E)	71(7)	102(8)	54(6)	-5(6)	-18(5)	-4(6)
F(3E)	79(7)	51(6)	108(10)	-12(7)	13(8)	15(5)
F(4E)	39(6)	79(10)	49(10)	-6(7)	-6(5)	-21(6)
F(5E)	63(8)	46(6)	72(11)	-3(7)	11(10)	-21(6)
F(6E)	89(9)	96(9)	91(9)	25(8)	-22(8)	13(8)
F(7E)	62(8)	50(8)	82(8)	-16(7)	21(7)	2(7)
F(8E)	41(7)	81(13)	60(13)	-4(10)	16(7)	-17(7)
F(9E)	66(11)	70(12)	74(9)	8(10)	22(9)	-3(12)
F(10E)	62(11)	56(9)	75(12)	5(10)	-14(12)	1(9)
F(11E)	66(11)	68(11)	67(12)	-13(11)	9(11)	22(10)
F(12E)	51(9)	77(13)	66(13)	7(12)	8(10)	-12(9)
N(1B)	21(2)	30(2)	23(2)	-7(2)	0(2)	1(2)
C(2B)	27(3)	28(3)	23(3)	-7(2)	3(2)	-5(2)
C(3B)	27(3)	28(3)	35(3)	-4(2)	8(2)	1(2)
C(4B)	24(3)	24(3)	38(3)	-3(2)	7(2)	3(2)
C(5B)	27(3)	33(3)	23(3)	-3(2)	0(2)	3(2)
C(6B)	26(3)	23(3)	25(3)	-2(2)	2(2)	3(2)
C(7B)	23(3)	33(3)	47(3)	11(3)	8(2)	5(2)
C(8B)	39(4)	34(3)	68(5)	6(3)	16(3)	11(3)
C(9B)	46(4)	37(4)	80(5)	17(3)	25(4)	12(3)
C(10B)	51(4)	50(4)	73(5)	27(4)	17(4)	23(4)
C(11B)	39(4)	70(5)	59(5)	14(4)	0(3)	15(4)
C(12B)	33(3)	61(4)	53(4)	4(3)	4(3)	17(3)
C(13B)	32(3)	36(3)	31(3)	-7(3)	-3(2)	-4(3)
C(14B)	60(4)	50(4)	34(3)	-7(3)	-2(3)	-12(3)
C(15B)	67(5)	83(6)	37(4)	1(4)	-7(4)	-6(4)
C(16B)	55(5)	79(5)	45(4)	-23(4)	-18(3)	-3(4)
C(17B)	42(4)	66(5)	57(4)	-25(4)	-13(3)	-5(4)
C(18B)	30(3)	48(4)	40(3)	-14(3)	-3(3)	-12(3)
C(19B)	24(3)	32(3)	23(3)	-6(2)	-4(2)	3(2)
C(20B)	41(4)	47(4)	47(4)	-20(3)	6(3)	-7(3)
C(21B)	53(5)	66(5)	58(5)	-39(4)	7(4)	-15(4)
C(22B)	50(4)	77(5)	48(4)	-33(4)	6(3)	5(4)
C(23B)	36(4)	63(4)	38(4)	-15(3)	9(3)	10(3)
C(24B)	32(3)	36(3)	31(3)	-5(3)	-1(2)	6(3)
C(25B)	27(2)	29(2)	28(2)	0(1)	0(1)	1(1)
C(26B)	31(4)	33(4)	38(4)	5(3)	-5(3)	4(3)
C(27B)	41(4)	42(4)	39(4)	14(4)	-10(4)	1(4)
C(28B)	42(4)	55(5)	43(4)	18(4)	-1(4)	10(4)
O(29B)	45(4)	77(5)	40(4)	7(4)	-14(3)	-11(4)
O(30B)	61(5)	73(6)	69(6)	18(5)	10(5)	27(4)
C(31B)	55(7)	113(13)	92(12)	40(10)	12(8)	26(8)
C(32B)	25(3)	35(4)	45(4)	8(3)	-1(3)	6(3)
O(33B)	38(4)	46(4)	50(4)	5(3)	8(3)	7(3)
O(34B)	28(4)	36(4)	49(6)	9(4)	3(5)	-1(3)
C(35B)	39(6)	47(8)	88(11)	9(8)	18(9)	-5(5)
C(25D)	29(3)	30(3)	30(3)	0(1)	0(1)	0(1)
C(26D)	37(6)	38(5)	37(6)	13(5)	-1(5)	-1(5)
C(27D)	51(6)	44(6)	51(6)	14(6)	-1(6)	7(6)
C(28D)	57(6)	67(7)	69(7)	14(6)	1(6)	16(6)
O(29D)	70(11)	71(11)	91(11)	14(9)	18(9)	16(9)
O(30D)	58(6)	75(8)	74(8)	17(7)	1(7)	15(7)
C(31D)	54(10)	91(16)	94(17)	14(15)	9(13)	23(12)
C(32D)	25(5)	35(5)	37(6)	-7(5)	-5(5)	1(5)
O(33D)	44(4)	47(5)	48(4)	-2(3)	-5(3)	1(3)
O(34D)	24(6)	38(7)	47(8)	-4(6)	-2(7)	-4(6)
C(35D)	33(10)	41(12)	72(16)	-6(12)	3(13)	-10(9)
B(36B)	47(4)	44(4)	47(4)	5(3)	-16(3)	-3(3)
F(1F)	69(8)	40(6)	47(10)	2(6)	-1(8)	-12(6)
F(2F)	79(9)	55(8)	49(8)	-1(7)	2(7)	2(8)
F(3F)	66(7)	84(10)	64(7)	16(7)	-35(6)	-4(7)
F(4F)	42(8)	62(9)	55(13)	2(10)	9(8)	-3(8)
F(5F)	64(8)	42(6)	71(12)	2(8)	-15(10)	-12(6)
F(6F)	58(8)	64(11)	36(7)	6(7)	-7(6)	17(8)
F(7F)	70(9)	62(9)	67(8)	-2(7)	-27(7)	13(7)
F(8F)	43(7)	54(9)	50(11)	0(9)	-4(7)	1(7)
F(9F)	62(12)	52(11)	52(11)	8(10)	-5(12)	-11(11)
F(10F)	57(12)	56(10)	56(11)	7(10)	-16(11)	11(10)
F(11F)	54(9)	64(9)	52(7)	6(8)	-24(8)	-4(8)
F(12F)	45(9)	56(9)	48(9)	8(9)	-13(7)	-2(8)

Table 5. Hydrogen coordinates (x 10^4) and isotropic displacement parameters ($Å^2$ x 10^3) for **1a**.

	x	y	z	U(eq)
H(3A)	9712	-504	5652	37

H(5A)	9499	-1603	2865	38
H(8A)	11459	-641	5353	81
H(9A)	13064	-1295	5745	99
H(10A)	13336	-2599	5171	94
H(11A)	12004	-3249	4206	87
H(12A)	10399	-2595	3815	66
H(14A)	6863	-393	6148	48
H(15A)	6329	433	7503	69
H(16A)	7139	1676	7741	82
H(17A)	8482	2092	6623	78
H(18A)	9016	1266	5267	58
H(20A)	6631	-1741	2526	65
H(21A)	5991	-1989	847	87
H(22A)	6751	-1336	-562	109
H(23A)	8152	-435	-293	116
H(24A)	8792	-187	1386	90
H(25A)	7150	580	4225	51
H(26A)	7655	682	2369	61
H(26B)	6380	609	2281	61
H(27A)	7607	1775	3024	72
H(27B)	6449	1812	2561	72
H(31A)	5208	3134	5349	161
H(31B)	5807	2319	5623	161
H(31C)	6480	3079	5272	161
H(35A)	5066	-1907	4409	97
H(35B)	4853	-1225	5246	97
H(35C)	4296	-1177	4126	97
H(25C)	6710	430	4326	41
H(26C)	7833	1195	3344	65
H(26D)	7441	758	2311	65
H(27C)	6685	2021	2493	72
H(27D)	5807	1338	2378	72
H(31D)	3988	3092	4111	160
H(31E)	4169	2323	4822	160
H(31F)	5026	3029	4842	160
H(35L)	4830	-1811	4268	139
H(35M)	4206	-1036	3870	139
H(35N)	4827	-1586	3065	139
H(3B)	360	6607	7334	36
H(5B)	10	5645	4473	33
H(8B)	-495	7554	6771	56
H(9B)	-2006	8356	6536	65
H(10B)	-3326	7996	5325	69
H(11B)	-3134	6834	4348	67
H(12B)	-1624	6032	4582	59
H(14B)	1272	4969	8683	58
H(15B)	2068	5135	10318	75
H(16B)	3356	6125	10581	72
H(17B)	3849	6950	9209	66
H(18B)	3053	6784	7574	47
H(20B)	606	3853	4685	54
H(21B)	1052	3085	3244	71
H(22B)	2415	3502	2149	70
H(23B)	3333	4687	2495	55
H(24B)	2887	5455	3937	40
H(25B)	2988	4867	7144	34
H(26E)	2943	3724	5642	41
H(26F)	1888	3804	6314	41
H(27E)	2817	3524	7831	49
H(27F)	3107	2847	7011	49
H(31G)	6449	2857	6288	130
H(31H)	6163	3784	6328	130
H(31I)	6415	3309	7377	130
H(35D)	4872	6812	5534	87
H(35E)	5480	6052	5999	87
H(35F)	5057	6045	4825	87
H(25D)	2982	4580	5739	36
H(26G)	1841	4027	7148	45
H(26H)	2951	4154	7753	45
H(27G)	2910	2861	7006	59
H(27H)	2725	3240	5875	59
H(31J)	6559	3253	7616	119
H(31K)	6422	3360	6391	119
H(31L)	6138	4079	7150	119
H(35G)	4771	6864	6302	73
H(35H)	5049	6421	5243	73
H(35I)	5511	6088	6320	73

Crystallographic Data for 1g (CCDC : 1964495)

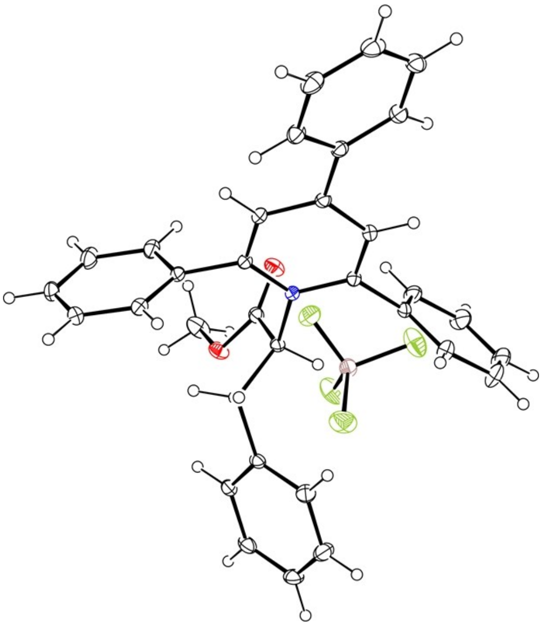

ORTEP representation (30% probability) of the crystal structure of **1g**

Table 1. Crystal data and structure refinement for **1g**.

Empirical formula	$C_{33}H_{28}NO_2BF_4$	
Formula weight	557.37	
Temperature	153(2) K	
Wavelength	0.71073 Å	
Crystal system	Monoclinic	
Space group	$P2_1/n$	
Unit cell dimensions	a = 11.0604(6) Å	α = 90°
	b = 16.6780(9) Å	β = 100.639(2)°
	c = 15.1704(8) Å	γ = 90°
Volume	2750.3(3) $Å^3$	
Z	4	
Density (calculated)	1.346 Mg/m^3	
Absorption coefficient	0.101 mm^{-1}	
F(000)	1160	
Crystal size	0.321 x 0.178 x 0.142 mm^3	
Theta range for data collection	2.993 to 28.278°.	
Index ranges	−14<=h<=14, −22<=k<=22, −20<=l<=20	
Reflections collected	46862	
Independent reflections	6750 [R(int) = 0.0360]	
Completeness to theta = 25.242°	98.9 %	
Absorption correction	Semi-empirical from equivalents	
Max. and min. transmission	0.7457 and 0.7034	
Refinement method	Full-matrix least-squares on F^2	
Data / restraints / parameters	6750 / 0 / 417	
Goodness-of-fit on F^2	1.030	
Final R indices [I>2sigma(I)]	R1 = 0.0418, wR2 = 0.0963	
R indices (all data)	R1 = 0.0531, wR2 = 0.1035	
Largest diff. peak and hole	0.344 and −0.202 e·$Å^{-3}$	

Table 2. Atomic coordinates (x 10^4) and equivalent isotropic displacement parameters ($Å^2$ x 10^3) for **1g**. U(eq) is defined as one third of the trace of the orthogonalized U^{ij} tensor.

	x	y	z	U(eq)
N(1)	5046(1)	3839(1)	3535(1)	18(1)
C(2)	5390(1)	3094(1)	3873(1)	18(1)
C(3)	6558(1)	2978(1)	4361(1)	20(1)
C(4)	7406(1)	3605(1)	4537(1)	19(1)
C(5)	7002(1)	4358(1)	4213(1)	20(1)
C(6)	5837(1)	4475(1)	3718(1)	19(1)
C(7)	8659(1)	3472(1)	5055(1)	21(1)
C(8)	9216(1)	2718(1)	5067(1)	26(1)
C(9)	10396(1)	2597(1)	5554(1)	33(1)
C(10)	11030(1)	3222(1)	6035(1)	34(1)
C(11)	10485(1)	3971(1)	6036(1)	32(1)

C(12)	9305(1)	4097(1)	5547(1)	26(1)
C(13)	4528(1)	2399(1)	3727(1)	20(1)
C(14)	3606(1)	2308(1)	4232(1)	26(1)
C(15)	2871(1)	1625(1)	4118(1)	33(1)
C(16)	3062(1)	1039(1)	3516(1)	35(1)
C(17)	3999(2)	1119(1)	3033(1)	34(1)
C(18)	4743(1)	1795(1)	3141(1)	27(1)
C(19)	5457(1)	5299(1)	3396(1)	20(1)
C(20)	4627(1)	5734(1)	3791(1)	29(1)
C(21)	4352(2)	6521(1)	3529(1)	36(1)
C(22)	4911(2)	6872(1)	2884(1)	39(1)
C(23)	5735(2)	6442(1)	2491(1)	39(1)
C(24)	6017(1)	5653(1)	2742(1)	30(1)
C(25)	3827(1)	3960(1)	2931(1)	19(1)
C(26)	3603(1)	3403(1)	2102(1)	21(1)
C(27)	2961(1)	3835(1)	1264(1)	21(1)
C(28)	1691(1)	3779(1)	976(1)	24(1)
C(29)	1123(1)	4164(1)	191(1)	29(1)
C(30)	1812(1)	4603(1)	-310(1)	32(1)
C(31)	3074(1)	4664(1)	-27(1)	33(1)
C(32)	3646(1)	4284(1)	758(1)	27(1)
C(33)	2837(1)	3981(1)	3515(1)	21(1)
O(34)	3021(1)	4235(1)	4271(1)	30(1)
O(35)	1767(1)	3720(1)	3072(1)	28(1)
C(36)	754(1)	3727(1)	3564(1)	39(1)
B(37)	7637(15)	3707(8)	2101(10)	23(2)
F(38)	7943(5)	3138(4)	2735(4)	31(1)
F(39)	6407(6)	3871(5)	1931(4)	41(1)
F(40)	7989(6)	3469(5)	1286(5)	44(1)
F(41)	8288(10)	4423(6)	2452(8)	42(2)
B(37B)	7790(20)	3771(14)	2005(15)	25(3)
F(38B)	7762(16)	3119(7)	2627(10)	67(3)
F(39B)	6528(15)	3967(9)	1729(17)	72(3)
F(40B)	8310(20)	3520(9)	1362(10)	68(3)
F(41B)	8346(16)	4419(9)	2356(12)	43(3)

Table 3. Bond lengths [Å] and angles [°] for **1g**.

N(1)-C(6)	1.3708(14)
N(1)-C(2)	1.3711(14)
N(1)-C(25)	1.4975(14)
C(2)-C(3)	1.3790(16)
C(2)-C(13)	1.4903(16)
C(3)-C(4)	1.3969(16)
C(3)-H(3)	0.9500
C(4)-C(5)	1.3924(16)
C(4)-C(7)	1.4790(16)
C(5)-C(6)	1.3810(16)
C(5)-H(5)	0.9500
C(6)-C(19)	1.4924(16)
C(7)-C(8)	1.3980(17)
C(7)-C(12)	1.3989(17)
C(8)-C(9)	1.3909(18)
C(8)-H(8)	0.9500
C(9)-C(10)	1.387(2)
C(9)-H(9)	0.9500
C(10)-C(11)	1.388(2)
C(10)-H(10)	0.9500
C(11)-C(12)	1.3931(18)
C(11)-H(11)	0.9500
C(12)-H(12)	0.9500
C(13)-C(14)	1.3918(17)
C(13)-C(18)	1.3932(17)
C(14)-C(15)	1.3927(18)
C(14)-H(14)	0.9500
C(15)-C(16)	1.380(2)
C(15)-H(15)	0.9500
C(16)-C(17)	1.383(2)
C(16)-H(16)	0.9500
C(17)-C(18)	1.3870(18)
C(17)-H(17)	0.9500
C(18)-H(18)	0.9500
C(19)-C(20)	1.3898(18)
C(19)-C(24)	1.3933(17)
C(20)-C(21)	1.3894(18)
C(20)-H(20)	0.9500
C(21)-C(22)	1.380(2)
C(21)-H(21)	0.9500
C(22)-C(23)	1.379(2)
C(22)-H(22)	0.9500
C(23)-C(24)	1.3895(19)
C(23)-H(23)	0.9500
C(24)-H(24)	0.9500
C(25)-C(33)	1.5307(17)
C(25)-C(26)	1.5471(16)
C(25)-H(25)	1.0000
C(26)-C(27)	1.5191(16)
C(26)-H(26A)	0.9900
C(26)-H(26B)	0.9900
C(27)-C(32)	1.3929(18)
C(27)-C(28)	1.3952(17)
C(28)-C(29)	1.3951(17)
C(28)-H(28)	0.9500
C(29)-C(30)	1.381(2)
C(29)-H(29)	0.9500
C(30)-C(31)	1.386(2)
C(30)-H(30)	0.9500
C(31)-C(32)	1.3927(18)
C(31)-H(31)	0.9500
C(32)-H(32)	0.9500
C(33)-O(34)	1.2028(15)
C(33)-O(35)	1.3229(14)
O(35)-C(36)	1.4575(16)
C(36)-H(36A)	0.9800
C(36)-H(36B)	0.9800
C(36)-H(36C)	0.9800
B(37)-F(38)	1.348(17)
B(37)-F(39)	1.365(18)
B(37)-F(40)	1.420(16)
B(37)-F(41)	1.445(18)
B(37B)-F(40B)	1.29(3)
B(37B)-F(41B)	1.31(3)
B(37B)-F(39B)	1.42(3)
B(37B)-F(38B)	1.44(3)
C(6)-N(1)-C(2)	120.25(10)
C(6)-N(1)-C(25)	119.16(9)
C(2)-N(1)-C(25)	120.54(9)
N(1)-C(2)-C(3)	119.71(10)
N(1)-C(2)-C(13)	121.53(10)
C(3)-C(2)-C(13)	118.76(10)
C(2)-C(3)-C(4)	121.80(11)
C(2)-C(3)-H(3)	119.1
C(4)-C(3)-H(3)	119.1
C(5)-C(4)-C(3)	116.62(10)
C(5)-C(4)-C(7)	122.10(10)
C(3)-C(4)-C(7)	121.27(10)
C(6)-C(5)-C(4)	121.72(11)
C(6)-C(5)-H(5)	119.1
C(4)-C(5)-H(5)	119.1
N(1)-C(6)-C(5)	119.81(10)
N(1)-C(6)-C(19)	121.12(10)
C(5)-C(6)-C(19)	119.07(10)
C(8)-C(7)-C(12)	118.91(11)

C(8)-C(7)-C(4)	120.60(11)
C(12)-C(7)-C(4)	120.49(11)
C(9)-C(8)-C(7)	120.35(12)
C(9)-C(8)-H(8)	119.8
C(7)-C(8)-H(8)	119.8
C(10)-C(9)-C(8)	120.24(13)
C(10)-C(9)-H(9)	119.9
C(8)-C(9)-H(9)	119.9
C(9)-C(10)-C(11)	120.07(12)
C(9)-C(10)-H(10)	120.0
C(11)-C(10)-H(10)	120.0
C(10)-C(11)-C(12)	119.91(13)
C(10)-C(11)-H(11)	120.0
C(12)-C(11)-H(11)	120.0
C(11)-C(12)-C(7)	120.53(13)
C(11)-C(12)-H(12)	119.7
C(7)-C(12)-H(12)	119.7
C(14)-C(13)-C(18)	120.08(11)
C(14)-C(13)-C(2)	121.09(11)
C(18)-C(13)-C(2)	118.49(11)
C(13)-C(14)-C(15)	119.45(12)
C(13)-C(14)-H(14)	120.3
C(15)-C(14)-H(14)	120.3
C(16)-C(15)-C(14)	120.25(13)
C(16)-C(15)-H(15)	119.9
C(14)-C(15)-H(15)	119.9
C(15)-C(16)-C(17)	120.27(12)
C(15)-C(16)-H(16)	119.9
C(17)-C(16)-H(16)	119.9
C(16)-C(17)-C(18)	120.17(13)
C(16)-C(17)-H(17)	119.9
C(18)-C(17)-H(17)	119.9
C(17)-C(18)-C(13)	119.72(13)
C(17)-C(18)-H(18)	120.1
C(13)-C(18)-H(18)	120.1
C(20)-C(19)-C(24)	120.17(11)
C(20)-C(19)-C(6)	120.46(11)
C(24)-C(19)-C(6)	119.18(11)
C(21)-C(20)-C(19)	119.77(12)
C(21)-C(20)-H(20)	120.1
C(19)-C(20)-H(20)	120.1
C(22)-C(21)-C(20)	120.07(13)
C(22)-C(21)-H(21)	120.0
C(20)-C(21)-H(21)	120.0
C(23)-C(22)-C(21)	120.21(13)
C(23)-C(22)-H(22)	119.9
C(21)-C(22)-H(22)	119.9
C(22)-C(23)-C(24)	120.52(13)
C(22)-C(23)-H(23)	119.7
C(24)-C(23)-H(23)	119.7
C(23)-C(24)-C(19)	119.25(13)
C(23)-C(24)-H(24)	120.4
C(19)-C(24)-H(24)	120.4
N(1)-C(25)-C(33)	107.95(9)
N(1)-C(25)-C(26)	114.01(9)
C(33)-C(25)-C(26)	117.40(10)
N(1)-C(25)-H(25)	105.5
C(33)-C(25)-H(25)	105.5
C(26)-C(25)-H(25)	105.5
C(27)-C(26)-C(25)	112.03(9)
C(27)-C(26)-H(26A)	109.2
C(25)-C(26)-H(26A)	109.2
C(27)-C(26)-H(26B)	109.2
C(25)-C(26)-H(26B)	109.2
H(26A)-C(26)-H(26B)	107.9
C(32)-C(27)-C(28)	118.81(11)
C(32)-C(27)-C(26)	119.90(11)
C(28)-C(27)-C(26)	121.27(11)
C(29)-C(28)-C(27)	120.35(12)
C(29)-C(28)-H(28)	119.8
C(27)-C(28)-H(28)	119.8
C(30)-C(29)-C(28)	120.37(12)
C(30)-C(29)-H(29)	119.8
C(28)-C(29)-H(29)	119.8
C(29)-C(30)-C(31)	119.70(12)
C(29)-C(30)-H(30)	120.2
C(31)-C(30)-H(30)	120.2
C(30)-C(31)-C(32)	120.25(13)
C(30)-C(31)-H(31)	119.9
C(32)-C(31)-H(31)	119.9
C(31)-C(32)-C(27)	120.51(12)
C(31)-C(32)-H(32)	119.7
C(27)-C(32)-H(32)	119.7
O(34)-C(33)-O(35)	125.37(11)
O(34)-C(33)-C(25)	123.09(11)
O(35)-C(33)-C(25)	111.49(10)
C(33)-O(35)-C(36)	115.88(10)
O(35)-C(36)-H(36A)	109.5
O(35)-C(36)-H(36B)	109.5
H(36A)-C(36)-H(36B)	109.5
O(35)-C(36)-H(36C)	109.5
H(36A)-C(36)-H(36C)	109.5
H(36B)-C(36)-H(36C)	109.5
F(38)-B(37)-F(39)	112.7(12)
F(38)-B(37)-F(40)	110.5(10)
F(39)-B(37)-F(40)	108.4(10)
F(38)-B(37)-F(41)	106.1(10)
F(39)-B(37)-F(41)	108.4(10)
F(40)-B(37)-F(41)	110.7(12)
F(40B)-B(37B)-F(41B)	109(2)
F(40B)-B(37B)-F(39B)	113.8(17)
F(41B)-B(37B)-F(39B)	107.2(17)
F(40B)-B(37B)-F(38B)	108.3(17)
F(41B)-B(37B)-F(38B)	114.9(18)
F(39B)-B(37B)-F(38B)	103.3(17)

Symmetry transformations used to generate equivalent atoms:

Table 4. Anisotropic displacement parameters (Å^2 x 10^3) for **1g**. The anisotropic displacement factor exponent takes the form: $-2\pi^2[h^2a^{*2}U^{11} + ... + 2 h k a^* b^* U^{12}]$

	U^{11}	U^{22}	U^{33}	U^{23}	U^{13}	U^{12}
N(1)	19(1)	16(1)	17(1)	1(1)	1(1)	-1(1)
C(2)	22(1)	16(1)	16(1)	1(1)	4(1)	-1(1)
C(3)	22(1)	17(1)	20(1)	3(1)	3(1)	0(1)
C(4)	20(1)	21(1)	16(1)	1(1)	4(1)	-1(1)
C(5)	22(1)	18(1)	19(1)	0(1)	3(1)	-3(1)
C(6)	22(1)	16(1)	18(1)	0(1)	4(1)	-1(1)
C(7)	19(1)	24(1)	20(1)	5(1)	3(1)	-2(1)
C(8)	24(1)	24(1)	29(1)	5(1)	3(1)	0(1)
C(9)	26(1)	34(1)	38(1)	12(1)	5(1)	6(1)
C(10)	20(1)	49(1)	31(1)	13(1)	0(1)	0(1)
C(11)	25(1)	41(1)	28(1)	3(1)	-1(1)	-9(1)
C(12)	24(1)	28(1)	26(1)	2(1)	2(1)	-3(1)
C(13)	21(1)	17(1)	20(1)	4(1)	-2(1)	-2(1)
C(14)	26(1)	21(1)	30(1)	4(1)	5(1)	-1(1)
C(15)	27(1)	30(1)	41(1)	9(1)	5(1)	-6(1)
C(16)	38(1)	25(1)	35(1)	6(1)	-8(1)	-14(1)
C(17)	52(1)	21(1)	25(1)	-2(1)	-1(1)	-8(1)
C(18)	37(1)	22(1)	22(1)	1(1)	4(1)	-3(1)
C(19)	23(1)	14(1)	21(1)	1(1)	-2(1)	-2(1)
C(20)	41(1)	21(1)	24(1)	2(1)	8(1)	3(1)
C(21)	53(1)	23(1)	33(1)	0(1)	9(1)	12(1)
C(22)	56(1)	18(1)	42(1)	8(1)	7(1)	6(1)
C(23)	49(1)	25(1)	44(1)	14(1)	15(1)	-1(1)
C(24)	32(1)	23(1)	36(1)	6(1)	10(1)	0(1)

C(25)	19(1)	17(1)	18(1)	1(1)	-2(1)	1(1)
C(26)	25(1)	19(1)	18(1)	-1(1)	0(1)	2(1)
C(27)	26(1)	17(1)	17(1)	-3(1)	0(1)	3(1)
C(28)	27(1)	21(1)	22(1)	-2(1)	-1(1)	-2(1)
C(29)	29(1)	26(1)	28(1)	-4(1)	-8(1)	2(1)
C(30)	44(1)	28(1)	20(1)	3(1)	-5(1)	6(1)
C(31)	41(1)	32(1)	26(1)	7(1)	8(1)	1(1)
C(32)	25(1)	29(1)	26(1)	1(1)	4(1)	3(1)
C(33)	22(1)	18(1)	23(1)	0(1)	1(1)	1(1)
O(34)	29(1)	36(1)	25(1)	-9(1)	4(1)	-3(1)
O(35)	20(1)	38(1)	25(1)	-6(1)	2(1)	-2(1)
C(36)	22(1)	57(1)	39(1)	-15(1)	9(1)	-5(1)
B(37)	25(3)	24(3)	21(3)	-3(3)	10(2)	-4(2)
F(38)	38(2)	28(2)	27(2)	4(1)	9(1)	4(1)
F(39)	22(1)	46(3)	51(2)	-15(2)	-1(1)	2(1)
F(40)	48(2)	60(2)	28(2)	-7(1)	16(1)	-8(1)
F(41)	28(2)	33(3)	61(4)	-13(2)	-1(2)	2(2)
B(37B)	20(5)	28(4)	28(5)	12(3)	6(4)	2(4)
F(38B)	116(7)	42(3)	40(3)	5(2)	9(4)	0(4)
F(39B)	45(4)	37(2)	115(8)	-6(5)	-33(4)	2(3)
F(40B)	107(9)	49(3)	60(4)	2(3)	45(5)	9(5)
F(41B)	44(5)	36(5)	44(3)	11(3)	-5(3)	-13(4)

Table 5. Hydrogen coordinates (x 10^4) and isotropic displacement parameters ($Å^2$ x 10^3) for **1g**.

	x	y	z	U(eq)
H(3)	6793	2458	4584	24
H(5)	7542	4804	4336	24
H(8)	8785	2287	4740	31
H(9)	10769	2083	5557	39
H(10)	11838	3137	6363	41
H(11)	10916	4398	6370	38
H(12)	8936	4611	5547	32
H(14)	3480	2709	4650	31
H(15)	2235	1561	4457	40
H(16)	2547	579	3433	41
H(17)	4135	711	2626	41
H(18)	5396	1846	2816	32
H(20)	4249	5493	4239	34
H(21)	3779	6818	3794	43
H(22)	4726	7412	2710	47
H(23)	6114	6687	2046	46
H(24)	6584	5357	2470	36
H(25)	3854	4516	2687	22
H(26A)	4402	3192	1998	26
H(26B)	3094	2941	2222	26
H(28)	1210	3478	1315	29
H(29)	257	4125	1	35
H(30)	1423	4861	-845	38
H(31)	3550	4966	-370	39
H(32)	4511	4332	949	32
H(36A)	87	3385	3257	58
H(36B)	451	4277	3595	58
H(36C)	1042	3524	4173	58

Crystallographic Data for 3m (CCDC : 1964489)

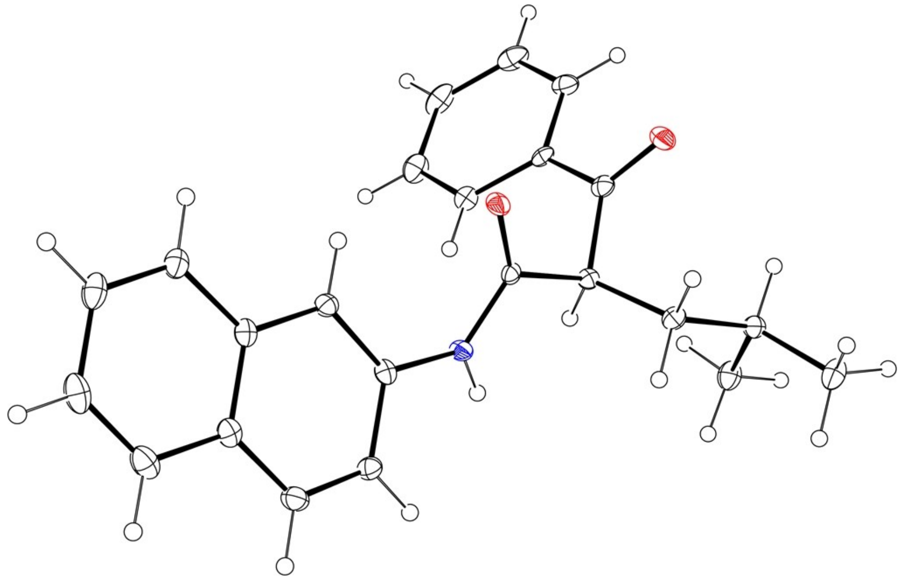

ORTEP representation (30% probability) of the crystal structure of **3m**

Table 1. Crystal data and structure refinement for **3m**.

Empirical formula	$C_{23}H_{23}NO_2$	
Formula weight	345.42	
Temperature	153(2) K	
Wavelength	0.71073 Å	
Crystal system	Monoclinic	
Space group	$P2_1/c$	
Unit cell dimensions	a = 9.3351(6) Å	α = 90°
	b = 20.6304(13) Å	β = 105.289(2)°
	c = 9.7275(6) Å	γ = 90°
Volume	1807.1(2) $Å^3$	
Z	4	
Density (calculated)	1.270 Mg/m^3	
Absorption coefficient	0.080 mm^{-1}	
F(000)	736	
Crystal size	0.254 x 0.083 x 0.068 mm^3	
Theta range for data collection	2.468 to 26.422°.	
Index ranges	−11<=h<=11, −25<=k<=25, −12<=l<=11	
Reflections collected	34021	
Independent reflections	3634 [R(int) = 0.0734]	
Completeness to theta = 25.242°	98.4 %	
Absorption correction	Semi-empirical from equivalents	
Max. and min. transmission	0.7454 and 0.7067	
Refinement method	Full-matrix least-squares on F^2	
Data / restraints / parameters	3634 / 0 / 240	
Goodness-of-fit on F^2	1.202	
Final R indices [I>2sigma(I)]	R1 = 0.0841, wR2 = 0.1306	
R indices (all data)	R1 = 0.1079, wR2 = 0.1379	
Largest diff. peak and hole	0.250 and −0.256 e·Å^{-3}	

Table 2. Atomic coordinates (x 10^4) and equivalent isotropic displacement parameters ($Å^2$ x 10^3) for **3m**. U(eq) is defined as one third of the trace of the orthogonalized U^{ij} tensor.

	x	y	z	U(eq)
C(1)	4942(3)	3840(1)	4332(3)	19(1)
C(2)	4693(3)	3415(1)	5322(3)	18(1)
C(3)	5552(3)	3445(1)	6750(3)	22(1)
C(4)	6652(3)	3890(1)	7153(3)	25(1)
C(5)	6955(3)	4336(1)	6170(3)	21(1)
C(6)	6071(3)	4315(1)	4740(3)	18(1)
C(7)	6339(3)	4781(1)	3763(3)	24(1)
C(8)	7433(3)	5226(1)	4169(3)	29(1)
C(9)	8338(3)	5239(2)	5580(4)	32(1)
C(10)	8096(3)	4805(1)	6554(3)	30(1)
N(11)	3577(2)	2934(1)	5008(2)	18(1)
C(12)	2987(3)	2639(1)	3756(3)	16(1)
O(13)	3318(2)	2766(1)	2645(2)	22(1)
C(14)	1891(3)	2101(1)	3827(3)	17(1)

C(15)	761(3)	2067(1)	2378(3)	20(1)
O(16)	899(2)	1671(1)	1495(2)	27(1)
C(17)	-491(3)	2542(1)	2059(3)	17(1)
C(18)	-489(3)	3091(1)	2897(3)	22(1)
C(19)	-1645(3)	3528(2)	2551(3)	30(1)
C(20)	-2820(3)	3417(2)	1374(4)	33(1)
C(21)	-2845(3)	2875(2)	540(3)	32(1)
C(22)	-1677(3)	2438(1)	874(3)	25(1)
C(23)	2745(3)	1465(1)	4238(3)	20(1)
C(24)	1805(3)	874(1)	4356(3)	21(1)
C(25)	2827(3)	297(1)	4874(3)	30(1)
C(26)	801(3)	992(1)	5339(3)	28(1)

Table 3. Bond lengths [Å] and angles [°] for **3m**.

C(1)-C(2)	1.367(4)
C(1)-C(6)	1.417(4)
C(1)-H(1)	0.9500
C(2)-C(3)	1.410(4)
C(2)-N(11)	1.413(3)
C(3)-C(4)	1.356(4)
C(3)-H(3)	0.9500
C(4)-C(5)	1.408(4)
C(4)-H(4)	0.9500
C(5)-C(10)	1.415(4)
C(5)-C(6)	1.419(4)
C(6)-C(7)	1.419(4)
C(7)-C(8)	1.353(4)
C(7)-H(7)	0.9500
C(8)-C(9)	1.410(4)
C(8)-H(8)	0.9500
C(9)-C(10)	1.365(4)
C(9)-H(9)	0.9500
C(10)-H(10)	0.9500
N(11)-C(12)	1.343(3)
N(11)-H(11)	0.84(3)
C(12)-O(13)	1.228(3)
C(12)-C(14)	1.524(3)
C(14)-C(15)	1.523(4)
C(14)-C(23)	1.532(4)
C(14)-H(14)	1.0000
C(15)-O(16)	1.218(3)
C(15)-C(17)	1.494(4)
C(17)-C(22)	1.389(4)
C(17)-C(18)	1.394(4)
C(18)-C(19)	1.378(4)
C(18)-H(18)	0.9500
C(19)-C(20)	1.380(4)
C(19)-H(19)	0.9500
C(20)-C(21)	1.378(5)
C(20)-H(20)	0.9500
C(21)-C(22)	1.386(4)
C(21)-H(21)	0.9500
C(22)-H(22)	0.9500
C(23)-C(24)	1.523(4)
C(23)-H(23A)	0.9900
C(23)-H(23B)	0.9900
C(24)-C(26)	1.525(4)
C(24)-C(25)	1.526(4)
C(24)-H(24)	1.0000
C(25)-H(25A)	0.9800
C(25)-H(25B)	0.9800
C(25)-H(25C)	0.9800
C(26)-H(26A)	0.9800
C(26)-H(26B)	0.9800
C(26)-H(26C)	0.9800
C(2)-C(1)-C(6)	119.9(2)
C(2)-C(1)-H(1)	120.1
C(6)-C(1)-H(1)	120.1
C(1)-C(2)-C(3)	120.5(2)
C(1)-C(2)-N(11)	123.4(2)
C(3)-C(2)-N(11)	116.1(2)
C(4)-C(3)-C(2)	120.3(3)
C(4)-C(3)-H(3)	119.8
C(2)-C(3)-H(3)	119.8
C(3)-C(4)-C(5)	121.2(3)
C(3)-C(4)-H(4)	119.4
C(5)-C(4)-H(4)	119.4
C(4)-C(5)-C(10)	122.7(3)
C(4)-C(5)-C(6)	118.5(2)
C(10)-C(5)-C(6)	118.8(3)
C(1)-C(6)-C(5)	119.5(2)
C(1)-C(6)-C(7)	122.0(2)
C(5)-C(6)-C(7)	118.5(2)
C(8)-C(7)-C(6)	121.2(3)
C(8)-C(7)-H(7)	119.4
C(6)-C(7)-H(7)	119.4
C(7)-C(8)-C(9)	120.6(3)
C(7)-C(8)-H(8)	119.7
C(9)-C(8)-H(8)	119.7
C(10)-C(9)-C(8)	119.8(3)
C(10)-C(9)-H(9)	120.1
C(8)-C(9)-H(9)	120.1
C(9)-C(10)-C(5)	121.1(3)
C(9)-C(10)-H(10)	119.5
C(5)-C(10)-H(10)	119.5
C(12)-N(11)-C(2)	128.1(2)
C(12)-N(11)-H(11)	117(2)
C(2)-N(11)-H(11)	115(2)
O(13)-C(12)-N(11)	124.3(2)
O(13)-C(12)-C(14)	121.5(2)
N(11)-C(12)-C(14)	114.1(2)
C(15)-C(14)-C(12)	107.5(2)
C(15)-C(14)-C(23)	112.9(2)
C(12)-C(14)-C(23)	109.1(2)
C(15)-C(14)-H(14)	109.1
C(12)-C(14)-H(14)	109.1
C(23)-C(14)-H(14)	109.1
O(16)-C(15)-C(17)	121.1(2)
O(16)-C(15)-C(14)	120.5(2)
C(17)-C(15)-C(14)	118.4(2)
C(22)-C(17)-C(18)	119.3(2)
C(22)-C(17)-C(15)	118.7(2)
C(18)-C(17)-C(15)	122.0(2)
C(19)-C(18)-C(17)	120.5(3)
C(19)-C(18)-H(18)	119.7
C(17)-C(18)-H(18)	119.7
C(18)-C(19)-C(20)	119.6(3)
C(18)-C(19)-H(19)	120.2
C(20)-C(19)-H(19)	120.2
C(21)-C(20)-C(19)	120.6(3)
C(21)-C(20)-H(20)	119.7
C(19)-C(20)-H(20)	119.7
C(20)-C(21)-C(22)	120.0(3)
C(20)-C(21)-H(21)	120.0
C(22)-C(21)-H(21)	120.0
C(21)-C(22)-C(17)	119.9(3)
C(21)-C(22)-H(22)	120.0
C(17)-C(22)-H(22)	120.0
C(24)-C(23)-C(14)	115.7(2)
C(24)-C(23)-H(23A)	108.3
C(14)-C(23)-H(23A)	108.3
C(24)-C(23)-H(23B)	108.3
C(14)-C(23)-H(23B)	108.3
H(23A)-C(23)-H(23B)	107.4
C(23)-C(24)-C(26)	112.4(2)
C(23)-C(24)-C(25)	109.1(2)
C(26)-C(24)-C(25)	110.6(2)
C(23)-C(24)-H(24)	108.2
C(26)-C(24)-H(24)	108.2
C(25)-C(24)-H(24)	108.2
C(24)-C(25)-H(25A)	109.5
C(24)-C(25)-H(25B)	109.5
H(25A)-C(25)-H(25B)	109.5
C(24)-C(25)-H(25C)	109.5
H(25A)-C(25)-H(25C)	109.5
H(25B)-C(25)-H(25C)	109.5
C(24)-C(26)-H(26A)	109.5
C(24)-C(26)-H(26B)	109.5
H(26A)-C(26)-H(26B)	109.5
C(24)-C(26)-H(26C)	109.5
H(26A)-C(26)-H(26C)	109.5
H(26B)-C(26)-H(26C)	109.5

Symmetry transformations used to generate equivalent atoms:

Table 4. Anisotropic displacement parameters (Å^2 x 10^3) for **3m**. The anisotropic displacement factor exponent takes the form: $-2\pi^2$[$h^2a^{*2}U^{11}$ + ... + 2 h k a* b* U^{12}]

	U^{11}	U^{22}	U^{33}	U^{23}	U^{13}	U^{12}
C(1)	21(1)	20(1)	18(1)	-1(1)	7(1)	2(1)
C(2)	17(1)	18(1)	22(1)	-4(1)	9(1)	-1(1)
C(3)	26(2)	22(1)	18(1)	2(1)	7(1)	0(1)
C(4)	26(2)	26(2)	21(2)	-2(1)	1(1)	2(1)
C(5)	18(1)	19(1)	28(2)	-3(1)	7(1)	4(1)
C(6)	16(1)	16(1)	26(1)	-3(1)	11(1)	4(1)
C(7)	29(2)	19(1)	28(2)	-1(1)	15(1)	0(1)
C(8)	31(2)	20(2)	42(2)	1(1)	20(2)	-3(1)
C(9)	24(2)	25(2)	49(2)	-9(2)	13(2)	-7(1)
C(10)	23(2)	28(2)	35(2)	-9(1)	2(1)	0(1)
N(11)	21(1)	22(1)	14(1)	0(1)	7(1)	-6(1)
C(12)	17(1)	13(1)	18(1)	1(1)	5(1)	3(1)
O(13)	31(1)	22(1)	17(1)	-4(1)	13(1)	-6(1)
C(14)	17(1)	18(1)	17(1)	0(1)	6(1)	1(1)
C(15)	20(1)	21(1)	20(1)	4(1)	7(1)	-4(1)
O(16)	34(1)	26(1)	20(1)	-5(1)	5(1)	4(1)
C(17)	16(1)	19(1)	18(1)	7(1)	7(1)	-2(1)
C(18)	21(2)	23(2)	25(2)	2(1)	7(1)	1(1)
C(19)	28(2)	27(2)	39(2)	4(1)	13(1)	6(1)
C(20)	21(2)	33(2)	48(2)	17(2)	12(2)	8(1)
C(21)	21(2)	41(2)	30(2)	13(1)	2(1)	-3(1)
C(22)	25(2)	30(2)	19(1)	4(1)	5(1)	-4(1)
C(23)	18(1)	21(1)	21(1)	3(1)	6(1)	0(1)
C(24)	22(2)	20(1)	20(1)	1(1)	4(1)	-3(1)
C(25)	34(2)	21(2)	38(2)	6(1)	12(1)	5(1)
C(26)	28(2)	23(2)	35(2)	3(1)	13(1)	-1(1)

Table 5. Hydrogen coordinates (x 10^4) and isotropic displacement parameters (Å^2 x 10^3) for **3m**.

	x	y	z	U(eq)
H(1)	4360	3816	3372	23
H(3)	5358	3152	7433	26
H(4)	7228	3901	8116	30
H(7)	5740	4779	2808	29
H(8)	7592	5533	3497	35
H(9)	9114	5549	5853	39
H(10)	8704	4818	7506	35
H(11)	3290(30)	2801(14)	5710(30)	22
H(14)	1367	2212	4570	20
H(18)	315	3164	3713	27
H(19)	-1633	3903	3121	36
H(20)	-3618	3718	1136	40
H(21)	-3663	2801	-263	38
H(22)	-1688	2067	292	30
H(23A)	3298	1372	3521	24
H(23B)	3485	1529	5166	24
H(24)	1163	771	3383	25
H(25A)	2235	-101	4771	45
H(25B)	3356	359	5878	45
H(25C)	3547	262	4304	45
H(26A)	240	597	5402	41
H(26B)	109	1345	4957	41
H(26C)	1409	1110	6290	41

Table 6. Hydrogen bonds for **3m** [Å and °].

D-H...A	d(D-H)	d(H...A)	d(D...A)	<(DHA)
N(11)-H(11)...O(13)#1	0.84(3)	2.21(3)	3.008(3)	158(3)

Symmetry transformations used to generate equivalent atoms:
#1 x,-y+1/2,z+1/2

Crystallographic Data for 3y (CCDC : 1964493)

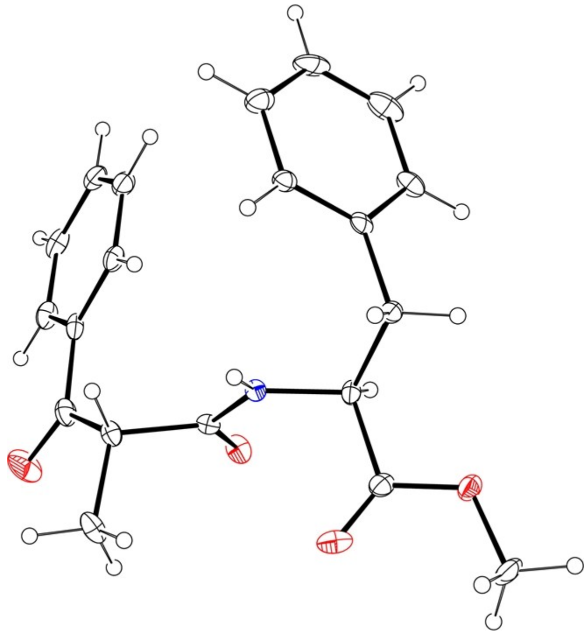

ORTEP representation (30% probability) of the crystal structure of **3y**

Table 1. Crystal data and structure refinement for **3y**.

Empirical formula	$C_{20}H_{21}NO_4$	
Formula weight	339.38	
Temperature	153(2) K	
Wavelength	0.71073 Å	
Crystal system	Monoclinic	
Space group	$P2_1/c$	
Unit cell dimensions	a = 9.2314(5) Å	α = 90°
	b = 19.6394(11) Å	β = 93.605(2)°
	c = 19.9121(10) Å	γ = 90°
Volume	3602.9(3) Å^3	
Z	8	
Density (calculated)	1.251 Mg/m^3	
Absorption coefficient	0.087 mm^{-1}	
F(000)	1440	
Crystal size	0.314 x 0.091 x 0.036 mm^3	
Theta range for data collection	2.594 to 26.999°.	
Index ranges	−11<=h<=11, −25<=k<=25, −25<=l<=25	
Reflections collected	49715	
Independent reflections	7787 [R(int) = 0.0995]	
Completeness to theta = 25.242°	99.2 %	
Absorption correction	Semi-empirical from equivalents	
Max. and min. transmission	0.7456 and 0.6480	
Refinement method	Full-matrix least-squares on F^2	
Data / restraints / parameters	7787 / 144 / 499	
Goodness-of-fit on F^2	1.144	
Final R indices [I>2sigma(I)]	R1 = 0.0892, wR2 = 0.1397	
R indices (all data)	R1 = 0.1420, wR2 = 0.1553	
Largest diff. peak and hole	0.271 and −0.308 e·Å^{-3}	

Table 2. Atomic coordinates (x 10^4) and equivalent isotropic displacement parameters (Å^2 x 10^3) for **3y**. U(eq) is defined as one third of the trace of the orthogonalized U^{ij} tensor.

	x	y	z	U(eq)
C(1)	6413(3)	5817(2)	2845(2)	23(1)
C(2)	5837(4)	5252(2)	3140(2)	27(1)
C(3)	4650(4)	4921(2)	2829(2)	28(1)
C(4)	4054(4)	5154(2)	2215(2)	30(1)
C(5)	4638(3)	5715(2)	1917(2)	27(1)
C(6)	5813(3)	6062(2)	2230(2)	23(1)
C(7)	6351(4)	6690(2)	1905(2)	26(1)
O(8)	5901(3)	6848(1)	1339(1)	46(1)
C(9)	7452(3)	7138(2)	2293(1)	22(1)
C(10)	7913(4)	7744(2)	1882(2)	33(1)

C(11)	6794(3)	7410(2)	2922(1)	18(1)
O(12)	5520(2)	7597(1)	2896(1)	26(1)
N(13)	7679(3)	7453(1)	3471(1)	16(1)
C(14)	7306(3)	7815(2)	4071(1)	19(1)
C(15A)	7740(13)	8581(7)	3973(7)	24(1)
O(16A)	8313(9)	8810(5)	3512(5)	34(2)
O(17A)	7539(9)	8924(3)	4525(3)	26(1)
C(18A)	7965(12)	9634(3)	4504(4)	35(2)
C(15B)	7520(30)	8534(15)	4024(14)	26(2)
O(16B)	7845(17)	8821(11)	3495(11)	34(3)
O(17B)	7028(15)	8873(6)	4574(6)	28(2)
C(18B)	7280(20)	9607(7)	4613(8)	37(3)
C(19)	8089(3)	7509(2)	4702(1)	22(1)
C(20)	7750(3)	6765(2)	4807(1)	20(1)
C(21)	6631(3)	6581(2)	5205(2)	27(1)
C(22)	6318(4)	5905(2)	5310(2)	40(1)
C(23)	7117(4)	5403(2)	5025(2)	39(1)
C(24)	8238(4)	5578(2)	4625(2)	33(1)
C(25)	8548(3)	6258(2)	4520(2)	22(1)
C(26)	-46(4)	5623(2)	2073(2)	34(1)
C(27)	-609(4)	5663(2)	1420(2)	48(1)
C(28)	-195(4)	6187(2)	1010(2)	42(1)
C(29)	788(4)	6663(2)	1257(2)	34(1)
C(30)	1358(3)	6620(2)	1914(2)	24(1)
C(31)	959(3)	6097(1)	2330(2)	22(1)
C(32)	1542(4)	6013(2)	3040(2)	25(1)
O(33)	1148(3)	5551(1)	3385(1)	41(1)
C(34)	2651(3)	6527(1)	3326(1)	18(1)
C(35)	3315(4)	6322(2)	4018(2)	27(1)
C(36)	1903(3)	7209(1)	3394(1)	16(1)
O(37)	681(2)	7240(1)	3609(1)	28(1)
N(38)	2641(3)	7758(1)	3231(1)	16(1)
C(39)	2114(3)	8439(1)	3341(1)	20(1)
C(40)	2462(4)	8685(2)	4055(2)	25(1)
O(41)	1851(3)	9152(1)	4300(1)	59(1)
O(42)	3532(3)	8345(1)	4370(1)	36(1)
C(43)	3952(5)	8569(2)	5048(2)	51(1)
C(44)	2774(4)	8931(2)	2848(1)	24(1)
C(45)	2323(3)	8769(1)	2120(2)	20(1)
C(46)	3242(3)	8414(2)	1724(2)	26(1)
C(47)	2831(4)	8283(2)	1056(2)	31(1)
C(48)	1501(4)	8501(2)	779(2)	30(1)
C(49)	570(4)	8842(2)	1170(2)	28(1)
C(50)	980(3)	8975(2)	1839(2)	25(1)

Table 3. Bond lengths [Å] and angles [°] for **3y**.

C(1)-C(2)	1.379(4)
C(1)-C(6)	1.397(4)
C(1)-H(1)	0.9500
C(2)-C(3)	1.386(4)
C(2)-H(2)	0.9500
C(3)-C(4)	1.386(5)
C(3)-H(3)	0.9500
C(4)-C(5)	1.377(5)
C(4)-H(4)	0.9500
C(5)-C(6)	1.393(4)
C(5)-H(5)	0.9500
C(6)-C(7)	1.492(4)
C(7)-O(8)	1.217(4)
C(7)-C(9)	1.519(4)
C(9)-C(10)	1.520(4)
C(9)-C(11)	1.522(4)
C(9)-H(9)	1.0000
C(10)-H(10A)	0.9800
C(10)-H(10B)	0.9800
C(10)-H(10C)	0.9800
C(11)-O(12)	1.231(3)
C(11)-N(13)	1.324(4)
N(13)-C(14)	1.451(4)
N(13)-H(13)	0.83(3)
C(14)-C(15B)	1.43(3)
C(14)-C(19)	1.532(4)
C(14)-C(15A)	1.571(14)
C(14)-H(14)	1.0000
C(15A)-O(16A)	1.177(15)
C(15A)-O(17A)	1.313(14)
O(17A)-C(18A)	1.448(8)
C(18A)-H(18A)	0.9800
C(18A)-H(18B)	0.9800
C(18A)-H(18C)	0.9800
C(15B)-O(16B)	1.25(3)
C(15B)-O(17B)	1.38(3)
O(17B)-C(18B)	1.461(17)
C(18B)-H(18D)	0.9800
C(18B)-H(18E)	0.9800
C(18B)-H(18F)	0.9800
C(19)-C(20)	1.511(4)
C(19)-H(19A)	0.9900
C(19)-H(19B)	0.9900
C(20)-C(25)	1.385(4)
C(20)-C(21)	1.388(4)
C(21)-C(22)	1.378(5)
C(21)-H(21)	0.9500
C(22)-C(23)	1.374(5)
C(22)-H(22)	0.9500
C(23)-C(24)	1.388(5)
C(23)-H(23)	0.9500
C(24)-C(25)	1.383(4)
C(24)-H(24)	0.9500
C(25)-H(25)	0.9500
C(26)-C(27)	1.372(5)
C(26)-C(31)	1.388(4)
C(26)-H(26)	0.9500
C(27)-C(28)	1.381(5)
C(27)-H(27)	0.9500
C(28)-C(29)	1.373(5)
C(28)-H(28)	0.9500
C(29)-C(30)	1.382(4)
C(29)-H(29)	0.9500
C(30)-C(31)	1.384(4)
C(30)-H(30)	0.9500
C(31)-C(32)	1.491(4)
C(32)-O(33)	1.208(4)
C(32)-C(34)	1.522(4)
C(34)-C(36)	1.516(4)
C(34)-C(35)	1.528(4)
C(34)-H(34)	1.0000
C(35)-H(35A)	0.9800
C(35)-H(35B)	0.9800
C(35)-H(35C)	0.9800
C(36)-O(37)	1.233(3)
C(36)-N(38)	1.328(4)
N(38)-C(39)	1.445(4)
N(38)-H(38)	0.83(3)

C(39)-C(40)	1.517(4)
C(39)-C(44)	1.531(4)
C(39)-H(39)	1.0000
C(40)-O(41)	1.195(4)
C(40)-O(42)	1.319(4)
O(42)-C(43)	1.449(4)
C(43)-H(43A)	0.9800
C(43)-H(43B)	0.9800
C(43)-H(43C)	0.9800
C(44)-C(45)	1.515(4)
C(44)-H(44A)	0.9900
C(44)-H(44B)	0.9900
C(45)-C(46)	1.382(4)
C(45)-C(50)	1.389(4)
C(46)-C(47)	1.385(4)
C(46)-H(46)	0.9500
C(47)-C(48)	1.382(5)
C(47)-H(47)	0.9500
C(48)-C(49)	1.370(5)
C(48)-H(48)	0.9500
C(49)-C(50)	1.386(4)
C(49)-H(49)	0.9500
C(50)-H(50)	0.9500
C(2)-C(1)-C(6)	120.6(3)
C(2)-C(1)-H(1)	119.7
C(6)-C(1)-H(1)	119.7
C(1)-C(2)-C(3)	120.1(3)
C(1)-C(2)-H(2)	119.9
C(3)-C(2)-H(2)	119.9
C(4)-C(3)-C(2)	119.9(3)
C(4)-C(3)-H(3)	120.1
C(2)-C(3)-H(3)	120.1
C(5)-C(4)-C(3)	120.0(3)
C(5)-C(4)-H(4)	120.0
C(3)-C(4)-H(4)	120.0
C(4)-C(5)-C(6)	120.9(3)
C(4)-C(5)-H(5)	119.5
C(6)-C(5)-H(5)	119.5
C(5)-C(6)-C(1)	118.5(3)
C(5)-C(6)-C(7)	118.8(3)
C(1)-C(6)-C(7)	122.6(3)
O(8)-C(7)-C(6)	120.6(3)
O(8)-C(7)-C(9)	120.1(3)
C(6)-C(7)-C(9)	119.2(3)
C(7)-C(9)-C(10)	112.4(3)
C(7)-C(9)-C(11)	109.5(2)
C(10)-C(9)-C(11)	107.9(3)
C(7)-C(9)-H(9)	109.0
C(10)-C(9)-H(9)	109.0
C(11)-C(9)-H(9)	109.0
C(9)-C(10)-H(10A)	109.5
C(9)-C(10)-H(10B)	109.5
H(10A)-C(10)-H(10B)	109.5
C(9)-C(10)-H(10C)	109.5
H(10A)-C(10)-H(10C)	109.5
H(10B)-C(10)-H(10C)	109.5
O(12)-C(11)-N(13)	123.6(3)
O(12)-C(11)-C(9)	120.0(3)
N(13)-C(11)-C(9)	116.3(3)
C(11)-N(13)-C(14)	123.0(2)
C(11)-N(13)-H(13)	122(2)
C(14)-N(13)-H(13)	113(2)
C(15B)-C(14)-N(13)	113.0(11)
C(15B)-C(14)-C(19)	112.4(11)
N(13)-C(14)-C(19)	111.0(2)
N(13)-C(14)-C(15A)	106.9(5)
C(19)-C(14)-C(15A)	111.5(5)
N(13)-C(14)-H(14)	109.1
C(19)-C(14)-H(14)	109.1
C(15A)-C(14)-H(14)	109.1
O(16A)-C(15A)-O(17A)	123.4(11)
O(16A)-C(15A)-C(14)	126.4(12)
O(17A)-C(15A)-C(14)	109.7(8)
C(15A)-O(17A)-C(18A)	114.7(6)
O(17A)-C(18A)-H(18A)	109.5
O(17A)-C(18A)-H(18B)	109.5
H(18A)-C(18A)-H(18B)	109.5
O(17A)-C(18A)-H(18C)	109.5
H(18A)-C(18A)-H(18C)	109.5
H(18B)-C(18A)-H(18C)	109.5
O(16B)-C(15B)-O(17B)	124(2)
O(16B)-C(15B)-C(14)	123(2)
O(17B)-C(15B)-C(14)	111.8(16)
C(15B)-O(17B)-C(18B)	117.3(13)
O(17B)-C(18B)-H(18D)	109.5
O(17B)-C(18B)-H(18E)	109.5
H(18D)-C(18B)-H(18E)	109.5
O(17B)-C(18B)-H(18F)	109.5
H(18D)-C(18B)-H(18F)	109.5
H(18E)-C(18B)-H(18F)	109.5
C(20)-C(19)-C(14)	113.7(2)
C(20)-C(19)-H(19A)	108.8
C(14)-C(19)-H(19A)	108.8
C(20)-C(19)-H(19B)	108.8
C(14)-C(19)-H(19B)	108.8
H(19A)-C(19)-H(19B)	107.7
C(25)-C(20)-C(21)	118.8(3)
C(25)-C(20)-C(19)	121.2(3)
C(21)-C(20)-C(19)	120.0(3)
C(22)-C(21)-C(20)	120.6(3)
C(22)-C(21)-H(21)	119.7
C(20)-C(21)-H(21)	119.7
C(23)-C(22)-C(21)	120.3(3)
C(23)-C(22)-H(22)	119.8
C(21)-C(22)-H(22)	119.8
C(22)-C(23)-C(24)	119.9(3)
C(22)-C(23)-H(23)	120.0
C(24)-C(23)-H(23)	120.0
C(25)-C(24)-C(23)	119.6(3)
C(25)-C(24)-H(24)	120.2
C(23)-C(24)-H(24)	120.2
C(24)-C(25)-C(20)	120.8(3)
C(24)-C(25)-H(25)	119.6
C(20)-C(25)-H(25)	119.6
C(27)-C(26)-C(31)	121.0(3)
C(27)-C(26)-H(26)	119.5
C(31)-C(26)-H(26)	119.5
C(26)-C(27)-C(28)	120.0(3)
C(26)-C(27)-H(27)	120.0
C(28)-C(27)-H(27)	120.0
C(29)-C(28)-C(27)	119.8(3)
C(29)-C(28)-H(28)	120.1
C(27)-C(28)-H(28)	120.1
C(28)-C(29)-C(30)	120.0(3)
C(28)-C(29)-H(29)	120.0
C(30)-C(29)-H(29)	120.0
C(29)-C(30)-C(31)	120.8(3)
C(29)-C(30)-H(30)	119.6
C(31)-C(30)-H(30)	119.6
C(30)-C(31)-C(26)	118.3(3)
C(30)-C(31)-C(32)	123.7(3)
C(26)-C(31)-C(32)	118.0(3)
O(33)-C(32)-C(31)	121.2(3)
O(33)-C(32)-C(34)	120.4(3)
C(31)-C(32)-C(34)	118.4(3)
C(36)-C(34)-C(32)	108.6(2)
C(36)-C(34)-C(35)	108.2(2)
C(32)-C(34)-C(35)	112.6(3)
C(36)-C(34)-H(34)	109.1
C(32)-C(34)-H(34)	109.1
C(35)-C(34)-H(34)	109.1
C(34)-C(35)-H(35A)	109.5
C(34)-C(35)-H(35B)	109.5
H(35A)-C(35)-H(35B)	109.5
C(34)-C(35)-H(35C)	109.5
H(35A)-C(35)-H(35C)	109.5
H(35B)-C(35)-H(35C)	109.5
O(37)-C(36)-N(38)	122.7(3)
O(37)-C(36)-C(34)	120.4(3)
N(38)-C(36)-C(34)	116.9(2)
C(36)-N(38)-C(39)	122.1(2)
C(36)-N(38)-H(38)	115(2)
C(39)-N(38)-H(38)	122(2)
N(38)-C(39)-C(40)	112.6(2)
N(38)-C(39)-C(44)	109.6(2)
C(40)-C(39)-C(44)	109.3(2)
N(38)-C(39)-H(39)	108.4
C(40)-C(39)-H(39)	108.4
C(44)-C(39)-H(39)	108.4
O(41)-C(40)-O(42)	123.6(3)
O(41)-C(40)-C(39)	123.3(3)
O(42)-C(40)-C(39)	113.1(3)
C(40)-O(42)-C(43)	116.0(3)
O(42)-C(43)-H(43A)	109.5
O(42)-C(43)-H(43B)	109.5
H(43A)-C(43)-H(43B)	109.5
O(42)-C(43)-H(43C)	109.5
H(43A)-C(43)-H(43C)	109.5
H(43B)-C(43)-H(43C)	109.5
C(45)-C(44)-C(39)	112.6(2)
C(45)-C(44)-H(44A)	109.1

C(39)-C(44)-H(44A)	109.1
C(45)-C(44)-H(44B)	109.1
C(39)-C(44)-H(44B)	109.1
H(44A)-C(44)-H(44B)	107.8
C(46)-C(45)-C(50)	118.8(3)
C(46)-C(45)-C(44)	120.6(3)
C(50)-C(45)-C(44)	120.6(3)
C(45)-C(46)-C(47)	120.1(3)
C(45)-C(46)-H(46)	120.0
C(47)-C(46)-H(46)	120.0
C(48)-C(47)-C(46)	120.5(3)
C(48)-C(47)-H(47)	119.7
C(46)-C(47)-H(47)	119.7
C(49)-C(48)-C(47)	119.9(3)
C(49)-C(48)-H(48)	120.0
C(47)-C(48)-H(48)	120.0
C(48)-C(49)-C(50)	119.6(3)
C(48)-C(49)-H(49)	120.2
C(50)-C(49)-H(49)	120.2
C(49)-C(50)-C(45)	121.0(3)
C(49)-C(50)-H(50)	119.5
C(45)-C(50)-H(50)	119.5

Symmetry transformations used to generate equivalent atoms:

Table 4. Anisotropic displacement parameters (Å^2 x 10^3) for **3y**. The anisotropic displacement factor exponent takes the form: $-2\pi^2$[$h^2a^{*2}U^{11}$ + ... + 2 h k a* b* U^{12}]

	U^{11}	U^{22}	U^{33}	U^{23}	U^{13}	U^{12}
C(1)	24(2)	22(2)	23(2)	-8(1)	-4(1)	2(1)
C(2)	32(2)	22(2)	27(2)	-4(1)	-2(2)	4(2)
C(3)	32(2)	19(2)	34(2)	-5(1)	5(2)	2(2)
C(4)	24(2)	26(2)	41(2)	-15(2)	-1(2)	2(2)
C(5)	23(2)	31(2)	25(2)	-9(1)	-6(1)	4(2)
C(6)	21(2)	28(2)	18(2)	-8(1)	1(1)	6(1)
C(7)	27(2)	35(2)	17(2)	-4(1)	-2(1)	3(2)
O(8)	55(2)	60(2)	21(1)	5(1)	-16(1)	-13(1)
C(9)	19(2)	29(2)	16(2)	-1(1)	1(1)	1(1)
C(10)	34(2)	46(2)	21(2)	3(2)	9(2)	-5(2)
C(11)	13(2)	20(2)	20(2)	4(1)	0(1)	-1(1)
O(12)	11(1)	42(1)	24(1)	2(1)	-2(1)	6(1)
N(13)	10(1)	21(1)	17(1)	0(1)	-1(1)	3(1)
C(14)	19(2)	22(2)	17(2)	-4(1)	-1(1)	4(1)
C(15A)	27(3)	21(2)	22(2)	0(2)	-8(2)	3(2)
O(16A)	34(4)	22(2)	44(2)	10(2)	-2(3)	-3(3)
O(17A)	31(3)	20(2)	26(2)	-5(1)	-4(2)	2(2)
C(18A)	53(4)	14(2)	37(3)	-7(2)	-7(3)	3(3)
C(15B)	28(3)	23(3)	25(3)	1(2)	-6(3)	2(3)
O(16B)	35(5)	27(4)	39(4)	7(3)	-2(5)	2(5)
O(17B)	32(3)	22(2)	30(2)	-5(2)	-2(3)	1(3)
C(18B)	45(5)	25(4)	41(4)	-1(4)	-4(4)	6(5)
C(19)	26(2)	20(2)	18(2)	-1(1)	-5(1)	4(1)
C(20)	21(2)	24(2)	13(1)	2(1)	-6(1)	-2(1)
C(21)	21(2)	38(2)	22(2)	7(1)	-1(1)	-2(2)
C(22)	33(2)	54(3)	32(2)	16(2)	-2(2)	-17(2)
C(23)	54(3)	22(2)	39(2)	13(2)	-13(2)	-18(2)
C(24)	45(2)	21(2)	31(2)	0(1)	-9(2)	-1(2)
C(25)	26(2)	20(2)	19(2)	2(1)	-3(1)	-2(1)
C(26)	36(2)	17(2)	49(2)	1(2)	-7(2)	-7(2)
C(27)	53(3)	23(2)	63(3)	-5(2)	-28(2)	-9(2)
C(28)	56(3)	29(2)	39(2)	-5(2)	-24(2)	5(2)
C(29)	48(2)	24(2)	28(2)	0(1)	-6(2)	-2(2)
C(30)	23(2)	21(2)	29(2)	-3(1)	-1(1)	-4(1)
C(31)	21(2)	13(1)	32(2)	-5(1)	1(1)	2(1)
C(32)	29(2)	18(2)	30(2)	1(1)	6(2)	-1(1)
O(33)	57(2)	29(1)	39(2)	10(1)	7(1)	-19(1)
C(34)	19(2)	16(1)	21(2)	2(1)	4(1)	4(1)
C(35)	34(2)	24(2)	24(2)	4(1)	0(2)	12(2)
C(36)	13(2)	19(2)	16(1)	-1(1)	0(1)	3(1)
O(37)	13(1)	28(1)	42(1)	8(1)	10(1)	2(1)
N(38)	11(1)	18(1)	20(1)	-1(1)	3(1)	1(1)
C(39)	21(2)	17(2)	21(2)	-1(1)	-3(1)	1(1)
C(40)	31(2)	17(2)	26(2)	1(1)	-1(2)	3(1)
O(41)	92(2)	46(2)	36(2)	-19(1)	-15(2)	38(2)
O(42)	39(2)	48(2)	19(1)	-8(1)	-10(1)	18(1)
C(43)	63(3)	70(3)	19(2)	-13(2)	-14(2)	13(2)
C(44)	31(2)	18(2)	23(2)	0(1)	-2(1)	-4(1)
C(45)	24(2)	12(1)	23(2)	5(1)	-3(1)	-4(1)
C(46)	19(2)	28(2)	30(2)	5(1)	-1(1)	0(1)
C(47)	30(2)	36(2)	27(2)	-4(2)	6(2)	-6(2)
C(48)	35(2)	36(2)	19(2)	5(1)	-1(2)	-10(2)
C(49)	26(2)	29(2)	28(2)	8(1)	-7(2)	0(2)
C(50)	27(2)	20(2)	28(2)	-1(1)	2(1)	-2(1)

Table 5. Hydrogen coordinates (x 10^4) and isotropic displacement parameters (Å^2 x 10^3) for **3y**.

	x	y	z	U(eq)
H(1)	7225	6043	3061	28
H(2)	6254	5088	3558	32
H(3)	4245	4536	3036	34
H(4)	3243	4927	2000	36
H(5)	4235	5868	1493	32
H(9)	8328	6859	2430	26
H(10A)	8638	8011	2149	50
H(10B)	7066	8030	1762	50
H(10C)	8332	7582	1472	50
H(13)	8560(30)	7361(15)	3469(15)	19
H(14)	6234	7786	4113	23
H(18A)	8971	9666	4376	53
H(18B)	7886	9838	4949	53
H(18C)	7326	9876	4173	53
H(18D)	7164	9764	5074	56
H(18E)	6569	9841	4305	56
H(18F)	8260	9710	4485	56
H(19A)	9149	7560	4668	26

H(19B)	7815	7769	5100	26
H(21)	6076	6924	5406	32
H(22)	5545	5785	5580	48
H(23)	6903	4938	5101	46
H(24)	8790	5233	4425	39
H(25)	9317	6377	4247	26
H(26)	-348	5266	2353	41
H(27)	-1284	5331	1250	57
H(28)	-588	6216	559	51
H(29)	1077	7023	976	40
H(30)	2032	6953	2083	29
H(34)	3442	6576	3008	22
H(35A)	3994	6675	4187	41
H(35B)	3833	5889	3982	41
H(35C)	2542	6269	4330	41
H(38)	3490(30)	7686(15)	3131(15)	19
H(39)	1036	8440	3252	24
H(43A)	4689	8260	5251	77
H(43B)	3101	8569	5318	77
H(43C)	4352	9031	5034	77
H(44A)	2468	9401	2949	29
H(44B)	3845	8911	2912	29
H(46)	4156	8261	1911	31
H(47)	3469	8041	785	37
H(48)	1232	8414	319	36
H(49)	-351	8987	983	34
H(50)	332	9211	2109	30

Table 6. Hydrogen bonds for **3y** [Å and °].

D-H...A	d(D-H)	d(H...A)	d(D...A)	<(DHA)
N(13)-H(13)...O(37)#1	0.83(3)	1.98(3)	2.799(3)	170(3)
N(38)-H(38)...O(12)	0.83(3)	1.97(3)	2.798(3)	175(3)

Symmetry transformations used to generate equivalent atoms:
#1 x+1,y,z

Appendix III

DFT Calculation Data

DFT-optimized structure's energy components

Table 1. Computed energy components for optimized structures

	E(SCF)/(eV)	ZPE/(kcal/mol)	S(gas)/(cal/mol ·K)	G(solv)/(kcal/mol)
	B3LYP-D3 cc-pVTZ(-f)	B3LYP-D3 LACVP**	B3LYP-D3 LACVP**	B3LYP-D3 LACVP**
**1a**	-41254.461	324.56	197.381	-45.6
**4a**	-12611.136	113.222	94.529	-5.04
**SOMO1**	-15631.706	111.310	116.349	-10.66
**SOMO2**	-28243.914	227.976	160.630	-14.22
**SOMO3**	-42905.930	333.682	183.772	-10.07
**NHC2**	-39695.613	278.345	175.638	-10.7
**A**	-42922.277	340.605	187.047	-8.62
**B**	-42906.863	331.659	183.371	-49.29
**C**	-41259.223	322.826	202.881	-15.59
**G**	-42916.961	341.879	184.553	-38.15

Table 2. Cartesian coordinates of the optimized geometries

The cartesian coordinates of optimized geometries are given below in the standard XYZ format, and units are in Å.

==============================

1a

==============================

C	2.380456448	-3.932318449	-2.666105032
C	1.346148133	-2.985240698	-2.724421263
C	0.506202579	-2.741958380	-1.652178884
N	0.683227301	-3.426302433	-0.467227787
C	1.640153646	-4.419026852	-0.389408112
C	2.474519014	-4.659194469	-1.472528696
H	1.185467720	-2.394947529	-3.617389679
H	3.185084105	-5.469717503	-1.376757741
C	1.820352435	-5.250087261	0.824339390
C	0.782620370	-6.036120892	1.349028111
C	3.099278927	-5.318954945	1.399098396
C	1.023926973	-6.860478401	2.445599794
C	3.331207275	-6.138930798	2.498777866
C	2.293308020	-6.910138130	3.024660587
H	-0.197198123	-6.007696629	0.888265193
H	3.900568724	-4.707257271	0.997208834
H	0.220212355	-7.472148418	2.842964411
H	4.318655014	-6.172833920	2.947865725
H	2.474381447	-7.551146030	3.881634235
C	3.289796352	-4.159977913	-3.797966480
C	4.612762928	-4.584746838	-3.574029684
C	2.859974623	-3.943514109	-5.120558262
C	5.481882095	-4.778525352	-4.642934799
C	3.728760958	-4.149743557	-6.187301636
C	5.041904449	-4.563747406	-5.951409340
H	4.974859238	-4.726499557	-2.560377121
H	1.834191084	-3.649843693	-5.320093632
H	6.504467964	-5.090866089	-4.456702232
H	3.380858183	-3.992954016	-7.203328133

H	5.720320225	-4.718499184	-6.784608841
C	-0.543756902	-1.704673529	-1.821467519
C	-1.905859947	-2.031651258	-1.825844169
C	-0.145380095	-0.386173010	-2.095423222
C	-2.858106852	-1.038511395	-2.051106691
C	-1.101769090	0.602531910	-2.312051058
C	-2.460290432	0.278676361	-2.282500744
H	-2.212666035	-3.059853792	-1.681895375
H	0.911618233	-0.137386665	-2.115136147
H	-3.912047386	-1.297389507	-2.054325819
H	-0.787206888	1.623081207	-2.505955219
H	-3.205508709	1.049505949	-2.451031685
O	-2.274697781	-2.754800081	1.539542317
O	-1.752649426	-4.486021042	0.179497555
C	-3.120747089	-4.951542377	0.293321103
H	-3.357603550	-5.169107914	1.336670399
H	-3.808107615	-4.186968327	-0.075966142
H	-3.172760963	-5.851648331	-0.317234814
C	0.000540215	-2.978530169	0.795006454
C	-1.475307703	-3.362633467	0.871492863
C	0.209107906	-1.491478801	1.143299818
H	-0.098863780	-1.407724023	2.188222647
H	-0.464947164	-0.858526647	0.568724811
H	0.491751492	-3.544245243	1.587692142
C	1.648542881	-1.000628591	0.992560387
H	1.696621776	0.070740335	1.215870619
H	2.021193743	-1.097420812	-0.034133077
C	2.624112844	-1.706784010	1.916580677
O	2.322189331	-2.566102028	2.721499920
O	3.875531673	-1.262511611	1.726714730
C	4.883378029	-1.827928185	2.593117476
H	4.676833630	-1.567682147	3.633871078
H	4.901957512	-2.916446447	2.499174595
H	5.827361107	-1.392842293	2.267326593

==============================

4a

==============================

C	-1.547783136	0.929518998	-8.121999741
H	-1.359217525	0.344300300	-7.226773262
H	-2.150201321	1.822901011	-7.993658543
C	-1.078883767	0.588040233	-9.328943253
H	-1.327427626	1.229674101	-10.174752235
C	-0.252578318	-0.576955736	-9.668971062
C	0.025780460	-0.844342887	-10.999440193
C	0.284940392	-1.451562881	-8.676194191
C	0.810972452	-1.957174540	-11.395571709
C	1.048451900	-2.535653114	-9.024786949
C	1.336570859	-2.827914476	-10.387535095
H	-0.368939400	-0.188266069	-11.772818565
H	0.091822468	-1.250677466	-7.627440929
H	1.450331688	-3.189405918	-8.254711151
C	2.381081343	-4.186661720	-12.110918999
C	1.861781478	-3.326858282	-13.109066010
C	2.123214483	-3.940465689	-10.780004501
H	2.071801901	-3.531075478	-14.155060768
H	2.520577908	-4.597834110	-10.010589600
C	1.095085382	-2.237831831	-12.758792877
H	0.695649445	-1.576434851	-13.523627281
H	2.984648705	-5.042219162	-12.400009155

==============================

SOMO1

==============================

C	-0.781884670	1.195428371	-6.480188847
O	-1.283671021	0.294526994	-5.842049122
O	0.188518286	2.001420975	-5.996165276
C	0.623250961	1.699537754	-4.657907009
H	-0.209122956	1.786294818	-3.954081774
H	1.022223115	0.683061481	-4.602375507
H	1.399309993	2.429427147	-4.426513672
C	-1.129745960	1.561129212	-7.907838345
H	-1.170386791	2.651275396	-8.000090599
H	-0.315057725	1.219035029	-8.556103706
C	-2.447296858	0.889609814	-8.354624748
H	-2.317816019	-0.192078605	-8.227312088
H	-3.261645079	1.196086168	-7.692473888
C	-2.786519289	1.196530104	-9.773283958
H	-3.632558823	1.823855400	-10.033963203
C	-1.960611105	0.680409670	-10.847537041
O	-0.956418037	-0.005404373	-10.689236641
O	-2.423545837	1.049679995	-12.071739197

C	-1.646950483	0.573606849	-13.179882050
H	-0.620869577	0.949461401	-13.124608040
H	-1.614251256	-0.519856155	-13.189660072
H	-2.144441843	0.949728131	-14.074352264

===============================

SOMO2

===============================

C	-5.080355644	1.073748589	-7.195501804
O	-6.238286018	1.372361302	-7.001888752
O	-4.389935493	0.213405132	-6.410408497
C	-5.133462906	-0.345462143	-5.313919544
H	-5.473824978	0.443916112	-4.638116837
H	-6.006795883	-0.892198265	-5.679743290
H	-4.445195675	-1.018177509	-4.801885605
C	-4.213285446	1.577800155	-8.329958916
H	-4.780616283	2.348296881	-8.858128548
H	-3.320486069	2.039081573	-7.894628525
C	-3.812314272	0.443111151	-9.290677071
H	-3.261407614	-0.326063246	-8.738495827
H	-4.721930504	-0.024502851	-9.683140755
C	-2.959216356	0.939728379	-10.480590820
H	-3.494055748	1.762826681	-10.967813492
C	-2.843738317	-0.182836458	-11.502376556
O	-1.866899133	-0.879205406	-11.681588173
O	-4.004668713	-0.341171443	-12.175744057
C	-4.018732548	-1.414483190	-13.133206367
H	-3.270266056	-1.241177320	-13.911238670
H	-3.803144455	-2.369422674	-12.646525383
H	-5.021686077	-1.416880012	-13.560326576
C	-1.547423363	1.412357807	-10.061644554
H	-1.646241784	2.123273134	-9.233159065
H	-0.997985959	0.545009792	-9.683798790
C	-0.795051336	2.038606644	-11.195562363
H	-0.085105844	1.413037300	-11.727972984
C	-1.036458015	3.337849855	-11.684977531
C	-1.977931976	4.216898441	-11.106175423
C	-0.310518235	3.815428019	-12.839277267
C	-2.223829269	5.504806042	-11.630156517
C	-0.529070199	5.057966232	-13.358118057
C	-1.490878820	5.946768761	-12.782523155
H	-2.543418884	3.906430006	-10.232956886
H	0.422080725	3.154662371	-13.295140266
H	0.028817879	5.393092155	-14.229176521
C	-2.682963133	8.065258026	-12.726612091
C	-3.406813622	7.634542465	-11.590975761
C	-1.743398309	7.233771801	-13.307080269
H	-4.143617630	8.294105530	-11.141298294
H	-1.185782075	7.562944412	-14.180618286
C	-3.182247400	6.384978771	-11.055025101
H	-3.739123583	6.053356647	-10.182009697
H	-2.868426800	9.051599503	-13.141784668

===============================

SOMO3

===============================

C	-5.447345734	0.146011278	-4.570544243
C	-4.574685574	-0.828788877	-5.325400829
C	-3.383074760	-0.557877541	-5.917572975
C	-4.651411533	1.259048820	-3.866927147
C	-2.665009975	0.760186791	-6.059837818
C	-4.207404613	2.407617092	-4.789348602
C	-3.627241135	1.961028576	-6.143487453
H	-6.187108517	0.585239470	-5.249921322
H	-1.950855851	0.902854681	-5.235767841
H	-3.778056622	0.810074508	-3.381365776
H	-3.463653326	3.011840105	-4.254549503
H	-6.025550842	-0.414854854	-3.834301472
H	-5.266089916	1.673620105	-3.059758663
H	-2.061046839	0.723961711	-6.974277020
H	-5.059990406	3.071466208	-4.981647968
H	-3.104871750	2.809180737	-6.600746155
H	-4.440697670	1.696318030	-6.829971790
N	-4.967326641	-2.169808626	-5.447514057
S	-2.692573786	-2.009822369	-6.647021294
C	-4.076698303	-2.976984024	-6.150449753
C	-6.199019432	-2.647735596	-4.853818893
C	-7.385663986	-2.597136021	-5.617163181
C	-6.185191154	-3.084347486	-3.509197712
C	-8.576984406	-2.972985506	-4.982115269
C	-7.412204266	-3.444263458	-2.938036203
C	-8.600274086	-3.385705709	-3.656333208

H	-9.502696991	-2.947351217	-5.550549507
H	-7.427974224	-3.785080910	-1.906352997
H	-9.537883759	-3.670829535	-3.187435627
C	-7.500452995	-2.234626532	-7.103644371
H	-8.574286461	-2.314552069	-7.312944412
C	-7.113554478	-0.791968942	-7.472739220
H	-6.038470268	-0.618133962	-7.380510807
H	-7.387315273	-0.596896887	-8.515724182
H	-7.642273903	-0.064751409	-6.848271370
C	-6.811111450	-3.240614176	-8.046800613
H	-5.735057354	-3.063126087	-8.103028297
H	-6.957275867	-4.269465446	-7.711838245
H	-7.218450069	-3.131420135	-9.058215141
C	-4.951499939	-3.254190207	-2.612209558
H	-5.366206646	-3.599475622	-1.657110810
C	-4.177340031	-1.965109706	-2.285358906
H	-3.450306654	-2.169938087	-1.491559267
H	-3.619622231	-1.593863010	-3.148028612
H	-4.841235638	-1.169735551	-1.931753874
C	-3.984528542	-4.360184669	-3.077312708
H	-4.525856018	-5.246585369	-3.412541151
H	-3.357635260	-4.023375034	-3.905618906
H	-3.321510077	-4.634797096	-2.248876095
C	-4.265693665	-4.381387234	-6.361008644
O	-5.236346245	-4.989904881	-5.848522663
C	-3.256979942	-5.160409451	-7.149495602
C	-2.967363834	-6.458173275	-6.698378086
C	-2.655681372	-4.706305027	-8.332065582
C	-2.064021826	-7.263258934	-7.384737968
C	-1.758576155	-5.519377232	-9.027121544
C	-1.451950908	-6.794289589	-8.551192284
H	-3.472588778	-6.815709591	-5.807144165
H	-2.911512852	-3.730872631	-8.733447075
H	-1.839061737	-8.260165215	-7.015278816
H	-1.307741284	-5.158548355	-9.947478294
H	-0.749701917	-7.424043655	-9.090327263

==============================

NHC2

==============================

C	-5.336446285	0.124344900	-4.380138397
C	-4.497069836	-0.822394133	-5.205069065
C	-3.427489281	-0.509661555	-5.980499268
C	-4.549186707	1.346234322	-3.874851942
C	-2.834060669	0.841014028	-6.288422108
C	-4.328120708	2.455018520	-4.918933868
C	-3.889180422	1.963965297	-6.310660362
H	-6.218376637	0.443530351	-4.953014851
H	-2.032208443	1.086724877	-5.577196121
H	-3.582971096	0.995465636	-3.492794991
H	-3.576626301	3.151007175	-4.524951458
H	-5.732137203	-0.431343198	-3.525983810
H	-5.077778816	1.775489330	-3.016206503
H	-2.352002859	0.785424054	-7.271800995
H	-5.251967907	3.036231041	-5.033637524
H	-3.496971369	2.816371441	-6.877394676
H	-4.759130955	1.599207640	-6.870238304
N	-4.802451611	-2.190022230	-5.191281319
S	-2.750945330	-1.955392599	-6.740593433
C	-3.961366177	-2.977905750	-5.970984459
C	-6.103916645	-2.648283482	-4.757176876
C	-7.160953999	-2.611644030	-5.675409794
C	-6.277927399	-3.086622715	-3.436632156
C	-8.428388596	-3.004709005	-5.232850552
C	-7.561434269	-3.461797714	-3.038926363
C	-8.648687363	-3.427696705	-3.920295715
H	-9.259164810	-2.984075308	-5.934685230
H	-7.714111328	-3.806463957	-2.018541574
C	-3.998935223	-4.411527157	-5.959679604
O	-4.641962528	-5.031603336	-5.078967571
C	-3.230630159	-5.180591106	-6.990106583
C	-2.741091728	-6.442307949	-6.615237713
C	-3.035816193	-4.745624065	-8.309312820
C	-2.041939497	-7.230775833	-7.523431778
C	-2.341811180	-5.540860176	-9.222458839
C	-1.835639954	-6.780626774	-8.831139565
H	-2.931316137	-6.783580303	-5.603031635
H	-3.454333067	-3.799357891	-8.637826920
H	-1.660192251	-8.200498581	-7.215671539
H	-2.208296537	-5.195085526	-10.243787766
H	-1.293114424	-7.397663593	-9.541929245
C	-10.024696350	-3.838085890	-3.452719688

H	-10.729347229	-3.907345057	-4.286328316
H	-9.998829842	-4.812153816	-2.951703787
H	-10.430847168	-3.117304802	-2.732528925
C	-5.098910809	-3.201873302	-2.508882046
H	-5.415001392	-3.455331802	-1.493644953
H	-4.439732552	-3.990512371	-2.886990309
H	-4.513963699	-2.276425838	-2.469240904
C	-6.928766727	-2.185510397	-7.103916645
H	-6.277033806	-2.901331902	-7.618123531
H	-7.870819569	-2.123406887	-7.654567242
H	-6.431234360	-1.211249828	-7.162201881

==============================

A

==============================

C	-5.479611874	0.180343658	-4.712356567
C	-4.586470127	-0.817132473	-5.412141323
C	-3.378407717	-0.566507936	-5.954752445
C	-4.696487427	1.302662134	-4.006421089
C	-2.649683952	0.740347743	-6.131162643
C	-4.210088730	2.426896572	-4.937956333
C	-3.598043919	1.946045041	-6.265455246
H	-6.197235107	0.616353035	-5.418445110
H	-1.938422680	0.907233834	-5.308210373
H	-3.842244625	0.851409554	-3.489743948
H	-3.472929001	3.030594587	-4.393319607
H	-6.086104393	-0.355498344	-3.977704287
H	-5.329547405	1.742489100	-3.226788282
H	-2.039704800	0.670001090	-7.040333271
H	-5.047137737	3.099478006	-5.166264534
H	-3.058019400	2.781188488	-6.726760864
H	-4.395292759	1.671586275	-6.966977119
N	-4.976166248	-2.173994303	-5.476953983
S	-2.615664005	-2.054210424	-6.559380531
C	-4.055538177	-3.027589798	-6.138863564
C	-6.200574875	-2.628515244	-4.875676632
C	-7.409177780	-2.568531990	-5.612335205
C	-6.173172474	-3.106629610	-3.532968283
C	-8.586537361	-2.986256599	-4.970820427
C	-7.385415077	-3.510262251	-2.958956480
C	-8.583879471	-3.448027849	-3.662984848
H	-9.520116806	-2.956797123	-5.526572704
H	-7.382924557	-3.883032799	-1.938568711
H	-9.509292603	-3.770739794	-3.194453716
C	-7.605253696	-2.100678205	-7.062838554
H	-8.345598221	-2.806597710	-7.467207432
C	-8.271110535	-0.706266046	-7.097651005
H	-7.570682526	0.071884282	-6.774333477
H	-8.584624290	-0.467029631	-8.119658470
H	-9.153172493	-0.656383455	-6.448195934
C	-6.411691666	-2.148109913	-8.028635979
H	-5.665902615	-1.377109766	-7.813557148
H	-5.915523052	-3.120891809	-8.011331558
H	-6.780846119	-1.975159287	-9.045495033
C	-4.925659657	-3.274111748	-2.654081345
H	-5.331615925	-3.482925177	-1.656244278
C	-4.033408165	-2.032189608	-2.491206169
H	-3.312237024	-2.212780237	-1.686617255
H	-3.467404604	-1.804611802	-3.395809412
H	-4.619999409	-1.149546027	-2.217916727
C	-4.080989361	-4.506978989	-3.037921667
H	-4.704365253	-5.397972107	-3.162456512
H	-3.531245947	-4.344701290	-3.966643572
H	-3.351629734	-4.711514473	-2.246752739
C	-4.205136299	-4.355930805	-6.409981251
O	-5.319702625	-5.089524269	-6.018768311
H	-5.815653801	-4.615275860	-5.339477539
C	-3.212633133	-5.215980053	-7.073616505
C	-3.099836349	-6.557576656	-6.654815197
C	-2.397995710	-4.788905144	-8.138421059
C	-2.181650877	-7.418090820	-7.248165607
C	-1.478027105	-5.653869152	-8.728178024
C	-1.357934475	-6.971400261	-8.284721375
H	-3.745959520	-6.911190033	-5.859670639
H	-2.503671646	-3.784524918	-8.531276703
H	-2.108081818	-8.445025444	-6.899525642
H	-0.864708960	-5.297963619	-9.551814079
H	-0.641075194	-7.644140244	-8.746886253

==============================

B

==============================

C	-5.504149437	0.136705548	-4.582100391

C	-4.617226124	-0.801116884	-5.376431942
C	-3.447727442	-0.461554945	-5.970197678
C	-4.725609303	1.254503131	-3.865970850
C	-2.797120094	0.885434806	-6.130231857
C	-4.343029976	2.445963383	-4.761452675
C	-3.797539234	2.055530310	-6.145914078
H	-6.278858662	0.573705852	-5.224456310
H	-2.028438091	1.063257337	-5.358444214
H	-3.822202682	0.817448795	-3.427648544
H	-3.595736027	3.054095507	-4.232573509
H	-6.045426369	-0.455305904	-3.841647863
H	-5.324536800	1.628624916	-3.025269032
H	-2.253449440	0.884483755	-7.085466862
H	-5.219438553	3.094002962	-4.902554512
H	-3.325639963	2.938232899	-6.598216057
H	-4.628413677	1.776266575	-6.805116177
N	-4.965719700	-2.147627831	-5.494133949
S	-2.645478964	-1.883148193	-6.694913387
C	-4.010738373	-2.962600231	-6.209508419
C	-6.156824112	-2.665178061	-4.875360966
C	-7.383719921	-2.594512224	-5.580754757
C	-6.119990349	-3.098411798	-3.526024103
C	-8.559354782	-2.940188885	-4.903738022
C	-7.330543041	-3.432423830	-2.906145573
C	-8.546373367	-3.347827673	-3.575050592
H	-9.504302979	-2.898631573	-5.441570759
H	-7.311645031	-3.771719694	-1.872429490
H	-9.472900391	-3.609695435	-3.069123745
C	-7.536586761	-2.246562719	-7.065959454
H	-8.606709480	-2.396739721	-7.268520832
C	-7.245765686	-0.778892457	-7.429396152
H	-6.183048248	-0.545013130	-7.329750061
H	-7.529036045	-0.595360458	-8.473802567
H	-7.819519997	-0.087485947	-6.800966740
C	-6.775554180	-3.195966005	-8.009765625
H	-5.716355801	-2.935525417	-8.058901787
H	-6.818302631	-4.227552414	-7.655175209
H	-7.198361397	-3.122673988	-9.020543098
C	-4.855537415	-3.293296814	-2.682312727
H	-5.232276440	-3.642577171	-1.710512519
C	-4.057203770	-2.011546135	-2.384523392
H	-3.275328398	-2.230772734	-1.646461010
H	-3.566221476	-1.627036572	-3.280922413
H	-4.694664001	-1.221094489	-1.971669078
C	-3.920874357	-4.398026943	-3.212560177
H	-4.488348007	-5.243443966	-3.604860544
H	-3.312031031	-4.028329372	-4.039946079
H	-3.252069950	-4.729693890	-2.407366276
C	-4.222876549	-4.317128658	-6.414497852
O	-5.272763252	-4.905117035	-5.929311275
C	-3.228600025	-5.166917324	-7.134262085
C	-3.361991882	-6.561217785	-6.948431015
C	-2.209335089	-4.732940674	-8.007658005
C	-2.498137951	-7.464094162	-7.557103157
C	-1.348423839	-5.640777588	-8.625025749
C	-1.474786043	-7.013818264	-8.400013924
H	-4.177688599	-6.886159420	-6.311273098
H	-2.105066538	-3.680652618	-8.246636391
H	-2.622969389	-8.531538010	-7.379299164
H	-0.577054560	-5.269471645	-9.298028946
H	-0.798439443	-7.718644142	-8.879187584

==============================

C

==============================

C	2.475999832	-3.692347527	-2.585282326
C	1.266426563	-2.960139275	-2.743808985
C	0.307311594	-2.904969692	-1.769054174
N	0.508563757	-3.530829430	-0.512230575
C	1.595774293	-4.443222523	-0.418952793
C	2.540782690	-4.494332790	-1.416368127
H	1.051431417	-2.448073149	-3.674878359
H	3.320038557	-5.242413521	-1.328535199
C	1.652932286	-5.350403786	0.739443421
C	0.482718498	-5.873446941	1.320204496
C	2.894777775	-5.727271080	1.286300421
C	0.558104277	-6.747226715	2.402652979
C	2.964720964	-6.606922626	2.362509012
C	1.795873642	-7.122498989	2.929555893
H	-0.480430156	-5.583908558	0.917879760
H	3.804453373	-5.300670147	0.876847863
H	-0.357746661	-7.141771317	2.834193468
H	3.934395790	-6.879487038	2.770337582

H	1.849954128	-7.800380707	3.776114702
C	3.543973446	-3.679174900	-3.584942341
C	4.873365879	-3.989642620	-3.222366333
C	3.303068399	-3.337858200	-4.934057713
C	5.902338505	-3.967454910	-4.158541203
C	4.335234642	-3.311756849	-5.866604805
C	5.643066883	-3.627549648	-5.488622189
H	5.105295658	-4.212480545	-2.185652256
H	2.292766809	-3.117511988	-5.264043808
H	6.915937424	-4.202850819	-3.844903469
H	4.116252422	-3.051386118	-6.898712158
H	6.447292328	-3.605441809	-6.218050480
C	-0.980458081	-2.225462914	-2.021154881
C	-2.197602272	-2.899138689	-1.825924158
C	-1.006656051	-0.915571630	-2.526823044
C	-3.406301498	-2.271018982	-2.116499662
C	-2.217531204	-0.289678156	-2.816749334
C	-3.422045231	-0.963131070	-2.608148575
H	-2.180629015	-3.915317774	-1.449287772
H	-0.068193287	-0.389095128	-2.676313639
H	-4.339766026	-2.808469296	-1.972749949
H	-2.220641613	0.727888763	-3.196971178
H	-4.366136074	-0.473868698	-2.828968525
O	-1.785742640	-1.504141808	1.331562281
O	-1.897098303	-3.739475012	1.052462578
C	-3.293775558	-3.664968967	1.387269974
H	-3.427898884	-3.228430033	2.380094051
H	-3.821389437	-3.049059868	0.654182017
H	-3.656891346	-4.692638397	1.363586187
C	0.229589075	-2.703084707	0.700599611
C	-1.256030440	-2.550218105	1.032370090
C	0.908683538	-1.322053075	0.664337039
H	0.677146912	-0.823090732	1.607945442
H	0.466208756	-0.716223001	-0.129709110
H	0.643281400	-3.260382652	1.544206977
C	2.425940990	-1.397458673	0.479754657
H	2.842135906	-0.383119762	0.429761469
H	2.711117268	-1.884504080	-0.455296457
C	3.110769987	-2.099206924	1.634956241
O	2.611720562	-2.306755781	2.722283125
O	4.374081135	-2.458187580	1.320302010
C	5.116451740	-3.066666126	2.393216610
H	5.250653267	-2.358171225	3.215391397
H	4.591448784	-3.947612286	2.770194292
H	6.080834389	-3.341404200	1.964909434

================================

G

================================

C	-5.472752571	0.164469257	-4.679598808
C	-4.596690178	-0.842066228	-5.380643845
C	-3.368340015	-0.612943351	-5.938375950
C	-4.669833183	1.277813554	-3.978872538
C	-2.626358509	0.689065933	-6.100075245
C	-4.177977562	2.392257452	-4.918924809
C	-3.571357012	1.898956895	-6.244894028
H	-6.176700115	0.604095936	-5.395430088
H	-1.944396973	0.833600342	-5.250956535
H	-3.821586132	0.821305215	-3.456373215
H	-3.437248230	2.993274689	-4.379008770
H	-6.089412212	-0.367571086	-3.951722860
H	-5.300245285	1.721114397	-3.201441288
H	-1.989338279	0.614513338	-6.988297939
H	-5.009661674	3.067729235	-5.151558876
H	-3.017910957	2.720478535	-6.710123062
H	-4.366909981	1.632041454	-6.951537132
N	-4.978532314	-2.183160543	-5.450768948
S	-2.661374092	-2.093740702	-6.538570404
C	-4.072838306	-3.015833616	-6.077963829
C	-6.224354744	-2.652478218	-4.868018150
C	-7.409679413	-2.598124981	-5.639955521
C	-6.195987701	-3.107401371	-3.519689798
C	-8.591187477	-3.015901089	-5.006969929
C	-7.417996883	-3.507401943	-2.964895010
C	-8.604177475	-3.457341671	-3.691554785
H	-9.517376900	-2.995690823	-5.573940277
H	-7.433577061	-3.865798712	-1.940362096
H	-9.535350800	-3.775449991	-3.233131886
C	-7.584680557	-2.147268772	-7.099284172
H	-8.371886253	-2.812443972	-7.480751514
C	-8.154994965	-0.711163044	-7.162383556
H	-7.391319275	0.030159306	-6.897680759
H	-8.490975380	-0.489206761	-8.179797173

H	-9.006868362	-0.579911351	-6.485046864
C	-6.409661770	-2.304847002	-8.078656197
H	-5.600605011	-1.591793776	-7.889587879
H	-6.002824783	-3.320168018	-8.069483757
H	-6.774321556	-2.111528397	-9.091765404
C	-4.959098339	-3.257960320	-2.620956898
H	-5.383309841	-3.434477091	-1.626642585
C	-4.054986477	-2.023538351	-2.471744299
H	-3.359884262	-2.191599607	-1.643595219
H	-3.450896978	-1.832702518	-3.361715555
H	-4.631547451	-1.124401927	-2.239094973
C	-4.121049404	-4.510640621	-2.954436541
H	-4.749491215	-5.394560337	-3.096735716
H	-3.513707638	-4.367993832	-3.851717710
H	-3.433221817	-4.717577457	-2.129349470
C	-4.224885941	-4.387461185	-6.333091736
O	-5.273344040	-5.099662304	-5.892101288
H	-5.850350857	-4.573457718	-5.309206009
C	-3.247123003	-5.200309277	-7.057160854
C	-2.991628170	-6.502714157	-6.582225800
C	-2.595480680	-4.751561642	-8.221961975
C	-2.069824934	-7.314064503	-7.231502056
C	-1.678792477	-5.573913097	-8.869345665
C	-1.406036019	-6.850217819	-8.371569633
H	-3.511507034	-6.857559681	-5.699428082
H	-2.841435909	-3.786626577	-8.651568413
H	-1.866652608	-8.309464455	-6.849579334
H	-1.190703869	-5.225187778	-9.773701668
H	-0.687896550	-7.487305164	-8.878091812

Table 3. Vibrational frequencies (in cm^{-1}) of the optimized structures

==============================

1a

==============================

7.38	16.53	31.73	41.77	46.75	48.21
55.63	57.68	71.43	77.80	84.91	96.48
102.38	109.50	116.22	128.74	130.57	143.95
160.01	169.28	199.49	219.81	229.43	240.22
246.41	253.67	266.74	280.28	297.10	303.67
324.66	355.22	376.59	398.47	412.34	415.41
419.46	424.33	452.66	494.43	499.03	511.53
528.20	566.48	597.26	618.28	625.54	627.85
630.43	634.94	648.34	655.83	668.31	669.91
677.14	706.83	715.26	724.07	751.01	764.46
778.37	784.82	791.33	798.34	835.55	851.66
857.31	867.12	871.76	875.64	900.60	913.83
918.98	938.37	950.96	956.63	959.43	987.43
988.41	993.35	998.00	1013.32	1014.98	1016.62
1017.15	1020.17	1020.85	1022.45	1024.57	1031.92
1048.78	1057.59	1059.12	1072.67	1093.41	1116.94
1119.42	1123.51	1126.68	1159.58	1172.55	1177.90
1179.83	1203.88	1204.22	1206.03	1206.87	1207.93
1216.67	1221.12	1224.67	1229.93	1238.43	1259.01
1274.46	1283.22	1301.18	1305.58	1329.11	1335.56
1338.36	1344.26	1366.66	1371.17	1375.02	1381.34
1400.80	1409.57	1429.46	1450.48	1455.32	1476.18
1480.50	1484.24	1487.20	1492.07	1493.88	1494.53
1497.71	1503.51	1506.22	1506.40	1536.87	1538.00
1545.18	1590.03	1631.60	1632.44	1634.04	1650.51
1656.56	1657.91	1668.05	1809.48	1844.07	3062.42
3068.67	3072.62	3097.59	3112.93	3122.67	3148.94
3157.78	3171.51	3184.78	3190.12	3191.06	3191.47
3192.84	3194.53	3200.00	3200.15	3203.36	3209.29
3209.34	3212.42	3219.58	3220.08	3220.98	3237.78
3239.61	3241.51	3243.08			

==============================

4a

==============================

56.26	130.61	183.24	195.92	277.69	340.59
391.01	400.74	482.98	500.54	510.05	562.52
609.85	626.35	705.51	716.76	759.21	770.03
784.23	834.93	879.03	891.01	913.51	924.89
960.32	966.20	972.37	995.33	1034.14	1049.46
1054.14	1158.33	1191.03	1198.63	1214.77	1252.34
1290.14	1302.78	1345.55	1391.45	1414.29	1424.45
1463.88	1486.25	1514.26	1558.50	1622.04	1656.94
1686.24	1709.68	3140.38	3163.40	3165.27	3171.79

3174.85	3177.34	3190.71	3204.01	3204.65	3246.53

==============================

SOMO1

==============================

32.79	47.83	55.01	82.81	133.95	137.89
148.29	168.96	195.05	213.55	260.29	316.74
346.96	432.60	504.92	550.55	596.69	667.86
711.69	752.48	795.29	899.55	909.19	987.25
1021.42	1054.76	1062.67	1127.01	1178.54	1179.13
1184.04	1207.90	1211.19	1228.59	1239.29	1294.72
1338.65	1395.93	1440.79	1477.79	1482.69	1485.30
1488.23	1493.47	1495.10	1508.51	1508.87	1718.48
1823.10	3051.75	3053.11	3058.93	3068.54	3103.86
3121.48	3126.37	3135.53	3165.45	3169.75	3213.04

==============================

SOMO2

==============================

6.85	20.98	27.34	34.71	41.22	51.43
69.31	93.91	97.32	127.45	133.60	139.32
146.06	185.44	192.31	221.96	236.81	245.64
282.64	301.65	304.94	336.15	363.80	402.45
415.86	438.01	469.07	479.16	488.96	518.90
521.19	562.33	630.84	632.45	646.71	670.11
700.01	716.48	749.60	759.68	772.54	779.06
779.73	786.60	829.14	848.97	854.09	869.83
884.99	913.64	942.33	954.33	957.19	978.21
989.07	1007.54	1025.54	1037.27	1051.34	1056.42
1075.55	1088.08	1114.13	1156.32	1174.39	1180.13
1180.41	1184.27	1190.60	1204.51	1211.29	1215.94
1226.74	1242.42	1259.38	1267.63	1281.01	1306.64
1307.12	1317.81	1352.33	1363.05	1378.65	1388.08
1399.59	1406.80	1430.31	1448.33	1478.64	1481.31
1481.84	1488.82	1494.95	1495.28	1495.38	1507.85
1508.56	1510.70	1513.80	1547.08	1591.29	1641.50
1658.27	1820.18	1833.69	3046.75	3054.64	3059.02
3059.56	3066.57	3069.50	3101.25	3108.13	3132.37
3135.76	3136.80	3169.49	3170.06	3170.81	3170.96
3174.85	3188.82	3190.46	3191.00	3196.25	3204.09

==============================

SOMO3

==============================

21.24	38.99	44.90	49.94	56.54	57.51
69.65	81.13	107.08	121.37	130.53	137.58
163.29	184.96	191.20	209.71	216.82	224.28
244.43	252.07	274.03	278.48	288.10	289.84
299.33	305.60	311.45	320.54	328.07	342.36
353.73	364.80	373.66	395.59	417.97	420.54
452.46	464.75	476.59	495.77	500.18	529.24
539.70	551.76	552.70	560.07	615.64	631.12
639.63	645.68	650.42	682.26	705.50	712.13
714.77	772.04	782.49	787.82	811.52	819.30
830.07	837.75	862.85	886.34	910.07	916.48
919.19	936.45	941.03	948.13	949.73	952.99
963.10	979.02	979.64	980.08	980.44	990.52
999.38	1007.37	1015.38	1051.52	1060.15	1093.18
1094.36	1112.87	1118.19	1122.07	1129.96	1131.67
1140.16	1144.20	1144.64	1178.67	1188.44	1195.21
1196.95	1212.69	1223.51	1225.97	1232.36	1255.77
1259.08	1271.48	1290.37	1300.87	1311.72	1317.28
1322.56	1341.95	1349.81	1350.72	1365.21	1375.44
1379.64	1384.73	1397.49	1405.00	1407.27	1416.00
1418.04	1418.99	1431.20	1437.93	1439.78	1482.69
1492.97	1494.95	1497.45	1497.79	1498.44	1499.88
1501.45	1512.80	1513.75	1515.94	1517.76	1518.45
1522.39	1531.95	1534.00	1534.49	1575.25	1636.17
1637.32	1639.66	1651.09	1657.05	3014.84	3029.20
3030.19	3030.72	3040.58	3042.25	3043.98	3049.86
3050.67	3052.13	3060.82	3063.32	3066.45	3085.72
3096.25	3106.40	3107.59	3125.52	3126.43	3126.71
3135.20	3140.92	3154.69	3157.84	3171.38	3173.60
3179.74	3183.33	3195.35	3198.81	3204.38	3213.69

==============================

NHC2

==============================

26.50	31.93	36.58	37.93	47.24	50.56
73.64	89.99	104.60	131.22	140.02	147.53
152.78	164.10	193.95	203.48	216.49	237.81
240.42	251.38	273.14	285.11	294.28	322.46
325.45	340.26	350.93	383.00	408.39	419.61
437.64	451.67	471.10	511.75	520.61	528.12
543.64	551.15	572.69	575.85	585.02	604.42
631.54	642.00	647.56	673.07	706.55	713.15
758.61	774.20	787.99	808.67	829.84	865.08
871.17	873.48	896.74	914.23	940.21	949.17
957.79	960.64	980.93	982.29	989.37	1001.57
1006.36	1015.47	1038.88	1045.49	1049.17	1059.19

1064.03 1065.77 1066.45 1072.54 1093.41 1113.01

1121.55 1123.57 1146.50 1185.92 1195.04 1206.04

1212.60 1224.29 1232.32 1255.40 1291.52 1293.36

1299.62 1309.15 1313.78 1339.95 1343.59 1350.87

1365.62 1376.12 1383.57 1396.58 1406.17 1408.49

1417.69 1427.47 1429.56 1433.45 1458.43 1482.92

1483.91 1488.00 1494.53 1497.08 1497.50 1497.83

1498.75 1510.40 1512.57 1517.93 1520.47 1533.74

1535.68 1577.49 1637.47 1642.16 1651.28 1656.91

1664.31 3015.82 3026.75 3029.19 3036.03 3039.81

3040.21 3042.55 3046.43 3062.52 3065.72 3083.54

3090.79 3093.26 3096.09 3106.28 3107.82 3122.59

3129.56 3132.04 3167.77 3169.63 3173.78 3183.40

3195.14 3204.02 3215.40

==============================

A

==============================

16.45 32.08 37.87 40.92 50.22 60.36

77.33 82.25 97.99 103.21 119.73 138.72

144.75 163.83 193.44 210.39 214.07 220.12

241.40 247.09 250.45 261.26 275.94 277.97

291.42 301.77 310.96 320.95 333.72 336.50

357.28 359.33 369.36 382.74 398.40 416.97

422.45 435.28 472.94 483.40 491.94 497.46

520.72 535.83 543.38 544.55 559.91 599.07

611.02 623.89 629.67 634.82 655.13 700.37

709.51 713.96 772.18 775.26 792.12 812.50

819.56 823.28 835.64 857.19 880.45 905.54

914.17 926.26 926.78 936.34 944.15 947.66

952.83 957.96 977.17 980.37 984.20 986.56

994.27 994.67 1007.77 1012.17 1049.08 1055.16

1070.89 1091.39 1097.39 1111.60 1116.32 1123.14

1128.57 1135.63 1138.74 1151.54 1174.58 1179.72

1184.72 1192.29 1195.44 1219.54 1224.54 1231.70

1243.76 1253.37 1256.72 1267.24 1277.95 1296.81

1308.05 1310.82 1326.27 1338.16 1340.91 1350.02

1367.39 1375.42 1380.77 1386.72 1388.28 1396.77

1405.21 1408.27 1412.92 1419.95 1422.49 1440.22

1443.96 1484.93 1487.38 1490.05 1495.99 1497.24

1497.66 1504.56 1505.48 1511.38 1514.05 1514.81

1518.35 1523.22 1524.90 1531.82 1533.40 1540.03

1623.54 1629.28 1635.09 1646.09 1667.61 1711.27

3006.27 3006.41 3026.36 3027.91 3033.72 3037.87

3045.69 3047.49 3048.02 3049.11 3051.92 3056.10

3062.02 3083.32 3093.85 3104.72 3106.53 3111.01

3112.12 3114.79 3116.04 3148.95 3153.06 3158.63

3170.98 3173.55 3180.21 3182.35 3197.74 3201.29

3215.88 3222.36 3791.88

==============================

B

==============================

25.16 33.21 47.79 52.18 57.54 60.18

68.42 84.49 91.32 109.87 134.14 151.19

162.72 182.81 203.75 208.41 213.78 220.57

243.96 245.53 254.02 262.93 270.67 285.82

299.12 302.12 311.92 321.34 323.56 332.60

343.31 357.08 368.93 390.32 411.64 422.81

427.80 462.14 472.12 496.77 499.13 517.70

523.74 536.77 554.52 555.56 611.58 620.32

630.73 634.36 645.70 652.25 699.37 703.81

706.29 765.69 767.38 775.43 807.52 812.80

830.07 836.93 848.58 879.95 898.41 899.05

910.08 913.62 940.48 942.62 947.99 950.96

952.22 957.82 959.89 980.23 981.99 984.76

988.42 1004.99 1006.32 1049.44 1052.80 1076.40

1091.10 1101.30 1104.27 1121.49 1130.26 1131.84

1133.11 1140.42 1149.13 1173.30 1177.05 1181.50

1189.92 1193.27 1211.94 1221.37 1229.47 1253.96

1255.98 1269.27 1289.46 1292.14 1308.59 1312.71

1328.22 1331.80 1346.78 1349.04 1352.53 1371.12

1373.42 1377.79 1388.19 1395.34 1403.24 1409.94

1412.44 1413.96 1416.78 1444.29 1446.67 1474.53

1487.36 1490.60 1493.75 1494.81 1495.73 1497.40

1498.77 1510.13 1512.39 1512.83 1516.32 1516.83

1519.33 1523.66 1533.00 1536.69 1553.19 1615.12

1630.47 1635.22 1649.23 1684.96 2960.88 2996.08

2997.84 3005.31 3015.04 3021.49 3023.83 3027.00

3029.07 3030.16 3034.92 3039.19 3047.02 3072.48

3082.28 3085.68 3090.91 3117.61 3122.96 3124.90

3134.15 3136.96 3143.02 3146.01 3147.19 3153.91

3155.74 3157.65 3170.91 3178.00 3202.69 3212.55

==============================

C

==============================

13.48 24.35 40.87 43.40 46.89 53.37

56.84 61.07 69.98 75.97 82.05 105.70

118.39 123.42 130.33 142.75 158.28 168.49

171.66 181.19 208.61 216.73 230.60 239.61

249.41 255.35 266.30 281.35 295.14 309.23

322.30 365.37 373.34 396.33 414.97 424.83

428.04 433.95 449.39 491.65 496.25 504.08

511.68 571.44 585.23 597.47 601.84 623.66

624.84 628.75 631.69 640.17 642.61 662.46

676.71 703.87 715.44 716.29 718.62 756.19

769.96 777.84 781.04 789.83 824.71 835.21

845.14 864.74 866.86 875.83 890.41 895.39

902.39 912.31 926.23 939.28 939.69 968.30

984.78 985.87 988.98 990.76 999.92 1000.81

1003.67 1007.72 1014.96 1017.79 1019.19 1026.27

1052.26 1055.52 1057.92 1067.11 1073.60 1106.15

1113.29 1116.05 1118.57 1130.52 1137.78 1183.00

1183.72 1195.34 1195.77 1196.13 1202.85 1211.67

1214.60 1217.76 1222.07 1224.40 1234.60 1245.66

1261.21 1271.99 1287.68 1312.31 1325.24 1331.50

1338.37 1340.11 1359.85 1364.58 1369.44 1371.23

1383.66 1404.44 1408.33 1422.67 1430.92 1476.62

1480.49 1483.05 1485.64 1487.82 1489.26 1493.41

1495.08 1496.36 1509.11 1513.01 1513.48 1535.82

1539.78 1542.58 1618.59 1620.86 1627.02 1635.61

1653.90 1656.68 1661.11 1814.49 1825.89 3054.49

3060.54 3061.74 3096.58 3111.30 3132.32 3140.23

3142.69 3146.70 3168.88 3170.48 3172.47 3175.63

3175.91 3179.14 3182.18 3183.97 3193.35 3193.67

3197.88 3200.95 3205.78 3205.97 3207.91 3213.10

3216.09 3222.15 3236.12

===============================

G

===============================

17.07 35.24 44.15 46.94 56.68 63.46

76.95 82.79 102.88 117.50 125.15 138.71

151.11 172.09 192.41 213.51 222.38 230.83

249.31 253.34 271.60 279.50 286.56 297.56

299.47 312.56 329.63 332.66 348.65 358.11

360.94 372.92 388.85 400.65 413.73 428.53

450.44 472.35 485.52 494.20 499.32 513.61

537.59 542.56 547.50 552.29 561.51 614.72

622.90 634.01 640.86 651.88 666.28 703.26

707.89 721.78 773.13 782.20 787.97 812.85

822.83 825.64 834.99 860.33 884.19 903.34

919.36 932.97 938.84 949.04 949.39 951.18

955.09 958.97 979.91 982.81 990.18 991.38

1006.84 1012.41 1014.96 1019.46 1047.45 1057.67

1088.57 1092.16 1110.05 1118.52 1119.94 1124.51

1126.32 1136.65 1138.28 1147.31 1182.32 1187.60

1189.60 1204.80 1214.57 1226.24 1227.94 1231.68

1255.40 1259.87 1268.15 1269.48 1290.41 1305.84

1311.81 1316.10 1332.64 1338.63 1346.73 1350.82

1373.02 1376.33 1378.82 1382.81 1394.51 1405.59

1407.92 1416.78 1420.71 1423.91 1431.24 1442.06

1442.62 1480.64 1484.46 1486.13 1488.35 1496.84

1499.58 1503.66 1505.00 1511.16 1514.52 1515.75

1518.44 1522.49 1526.62 1527.48 1529.81 1532.49

1542.28 1612.08 1625.50 1629.25 1634.44 1650.36

3022.79 3033.40 3037.67 3045.76 3048.87 3051.51

3055.98 3056.80 3057.57 3062.82 3065.20 3081.04

3086.28 3101.37 3106.68 3109.03 3119.50 3120.19

3124.52 3127.89 3130.48 3132.19 3141.06 3149.25

3190.09 3196.39 3197.97 3203.68 3211.97 3215.32

3220.08 3225.92 3631.51
